# Screening for esophageal adenocarcinoma and precancerous conditions (dysplasia and Barrett’s esophagus) in patients with chronic gastroesophageal reflux disease with or without other risk factors: two systematic reviews and one overview of reviews to inform a guideline of the Canadian Task Force on Preventive Health Care (CTFPHC)

**DOI:** 10.1186/s13643-020-1275-2

**Published:** 2020-01-29

**Authors:** Candyce Hamel, Nadera Ahmadzai, Andrew Beck, Micere Thuku, Becky Skidmore, Kusala Pussegoda, Lise Bjerre, Avijit Chatterjee, Kristopher Dennis, Lorenzo Ferri, Donna E. Maziak, Beverley J. Shea, Brian Hutton, Julian Little, David Moher, Adrienne Stevens

**Affiliations:** 1 Ottawa Hospital Research Institute, Knowledge Synthesis Group, 501 Smyth Road, Ottawa, ON Canada; 2 Department of Family Medicine, University of Ottawa, Ottawa, ON Canada; 3 Gastroenterology Department, Faculty of Medicine, Unveristy of Ottawa, Ottawa, ON Canada; 4 Ottawa Hospital Research Institute, Cancer Therapeutics Program, Ottawa, ON Canada; 5 Division of Thoracic and Upper Gastrointestinal Surgery, McGill University, Montreal, QC, Canada; 6 Department of Surgery and The Ottawa Hospital, Department of Thoracic Surgery, University of Ottawa, Ottawa, ON Canada; 7 School of Epidemiology and Public Health, University of Ottawa, Ottawa, ON Canada

**Keywords:** Esophageal adenocarcinoma, Gastroesophageal reflux disease, Barrett’s esophagus, Dysplasia, Screening, Patient values and preferences, Treatment, Systematic review, Overview of reviews

## Abstract

**Background:**

Two reviews and an overview were produced for the Canadian Task Force on Preventive Health Care guideline on screening for esophageal adenocarcinoma in patients with chronic gastroesophageal reflux disease (GERD) without alarm symptoms. The goal was to systematically review three key questions (KQs): (1) The effectiveness of screening for these conditions; (2) How adults with chronic GERD weigh the benefits and harms of screening, and what factors contribute to their preferences and decision to undergo screening; and (3) Treatment options for Barrett’s esophagus (BE), dysplasia or stage 1 EAC (overview of reviews).

**Methods:**

Bibliographic databases (e.g. Ovid MEDLINE®) were searched for each review in October 2018. We also searched for unpublished literature (e.g. relevant websites). The liberal accelerated approach was used for title and abstract screening. Two reviewers independently screened full-text articles. Data extraction and risk of bias assessments were completed by one reviewer and verified by another reviewer (KQ1 and 2). Quality assessments were completed by two reviewers independently in duplicate (KQ3). Disagreements were resolved through discussion. We used various risk of bias tools suitable for study design. The GRADE framework was used for rating the certainty of the evidence.

**Results:**

Ten studies evaluated the effectiveness of screening. One retrospective study reported no difference in long-term survival (approximately 6 to 12 years) between those who had a prior esophagogastroduodenoscopy and those who had not (adjusted HR 0.93, 95% confidence interval (CI) 0.58–1.50). Though there may be higher odds of a stage 1 diagnosis than a more advanced diagnosis (stage 2–4) if an EGD had been performed in the previous 5 years (OR 2.27, 95% CI 1.00–7.67). Seven studies compared different screening modalities, and showed little difference between modalities. Three studies reported on patients’ unwillingness to be screened (e.g. due to anxiety, fear of gagging). Eleven systematic reviews evaluated treatment modalities, providing some evidence of early treatment effect for some outcomes.

**Conclusions:**

Little evidence exists on the effectiveness of screening and values and preferences to screening. Many treatment modalities have been evaluated, but studies are small. Overall, there is uncertainty in understanding the effectiveness of screening and early treatments.

**Systematic review registrations:**

PROSPERO (CRD42017049993 [KQ1], CRD42017050014 [KQ2], CRD42018084825 [KQ3]).

## Introduction

There are two main types of esophageal cancer. These are, esophageal adenocarcinoma (EAC) where malignant cells form in the tissues of the lower third of the esophagus, primarily in glandular cells where Barrett’s Esophagus (BE) also develops [1], and esophageal squamous cell carcinoma (ESCC), where malignant cells form in the squamous cells of the esophagus. ESCC is the most prominent form of esophageal neoplasm worldwide, with 398,000 cases of ESCC compared to 52,000 cases of EAC in 2012 [2]. However, EAC is more common than ESCC in Canada and nearly 50% of the worldwide cases of EAC occur in Northwestern Europe and North America [3]. From 1986 to 2006, EAC incidence in Canada rose by 3.9% (1.8 to 3.5 per 100,000) in males and 3.6% (0.2 to 0.5 per 100,000) in females per year [3]. Rates in Canada, provided by the Canadian Cancer Society, report the overall rates of esophageal cancer (combined EAC and ESCC). In 2017, projected new cases of esophageal cancer were 2330 cases (1800 among men and 530 among women) with 2130 deaths from the disease (1650 among men and 480 among women). Although esophageal cancer has a lower incidence than other cancers (ranked 13th among men and 19th among women), it has a high mortality rate and a low 5-year survival rate (14%), the second lowest survival rate after pancreatic cancer [4]. About 20% of EAC cases are diagnosed at an early stage where treatment with surgery leads to a 5-year survival rate of 90% [5].

### Risk factors

Increases in incidence of EAC may be dependent on the increasing prevalence of related risk factors such as obesity and gastroesophageal reflux disease (GERD) [3]. Other risk factors for the development EAC are BE, age 50 years and older, male sex, European descent, current or past smoking, a family history of BE or EAC and a diet low in fruits and vegetables [1, 6–8].

The prevalence of GERD in Western countries has increased over the past few decades and is one of the most commonly encountered conditions in primary care practice with an estimated prevalence of between 18–27% in the USA and 9–26% in Europe [9]. Extrapolating these prevalence estimates to the Canadian population, since no Canadian incidence studies exist, would mean that 3.4–6.8 million persons in Canada experience GERD [10]. GERD is a chronic disease with varying definitions [10–13]. The Montreal definition has been adopted by clinicians and researchers, and defines GERD as “a condition which develops when the reflux of stomach contents causes troublesome symptoms (e.g., retrosternal burning (heartburn), regurgitation) and/or complications (e.g., esophagitis, esophageal stricture)” [14]. According to the American Society for Gastrointestinal Endoscopy, chronic, long-standing GERD is defined as frequent severe GERD symptoms for over 5 years and requiring regular acid suppression therapy [15]. However, experts differ in the definition of the duration of symptoms and whether acid suppression therapy is considered in defining chronic GERD [16–18].

The most common complications of GERD are esophagitis, esophageal stricture, BE and EAC [10]. Approximately 60% of people with EAC have experienced symptoms of GERD and there is an association between the frequency and severity of symptoms and increased risk of EAC [19, 20]. In BE, the tissue lining the esophagus transforms into tissue resembling the lining of the intestines. Generally, this transformation is called intestinal metaplasia, and in the esophagus, it is called BE. It is currently not known how the transformation occurs; however, it has been suggested that the acid regurgitation associated with GERD may assist changes at the cellular level [19]. BE is known to develop in around 6–14% of people with GERD, and among those with BE (with or without GERD), 0.2–0.5% develop EAC [21]. However, not all individuals with BE will experience chronic GERD symptoms, and it is still unclear why such a small percentage of people with GERD develop BE [22, 23]. Once an individual is diagnosed with BE, regular surveillance using endoscopy should be considered, as BE can progress over time from low- to high-grade dysplasia and into EAC [24, 25]. Patients who have EAC discovered as a result of endoscopic screening or as part of a surveillance program for BE are diagnosed with earlier-stage tumours, are less likely to have lymph node involvement, and have better short-term life expectancies than those who present with alarm symptoms such as dysphagia and weight loss [26]. It has also been found that the longer the length of BE (e.g. short segment vs. long segment), the higher the risk for EAC [27].

### Treatment

The goal of treatment for BE and/or low- or high-grade dysplasia is to slow or halt GERD symptoms, reduce mucosal inflammation, control dysplasia and prevent progression to adenocarcinoma [28]. The treatments for EAC depend on the stage of the disorder (0 to 4). For stage 0, the disease is considered precancerous and is synonymous with high-grade dysplasia. Endoscopic therapies (e.g. radiofrequency ablation (RFA) or endoscopic mucosal resection (EMR)) are typically performed, followed by endoscopic surveillance [29]. For stage 1, the disease is generally treated with mechanical methods to remove tissue (e.g. endoscopic mucosal resection) followed by an ablative technique to destroy any remaining abnormal areas in the esophagus lining [29].

There are four main categories for managing and/or treating the conditions of interest (i.e. stage 1 EAC, BE or dysplasia): (1) pharmacological therapies; (2) surveillance (endoscopic); (3) endoscopic or endoscopic-assisted therapies; and (4) surgery (see Additional file 1). These strategies may overlap with some of the conditions of interest. For example, proton pump inhibitor (PPI) therapy is not a treatment for EAC but may reduce the risk of developing dysplasia and EAC among people with BE. These therapies may also be used in combination (e.g. pharmacological therapy and surveillance procedures for BE) depending on the disease progression.

### Objectives

With Canada’s increasing senior population and longer life expectancy, there is an expected increase in the incidence rates of GERD and EAC, and, therefore, increased demand for gastrointestinal endoscopies [10, 30]. From the Canadian Institute for Health Information National Physician Database, between 2004 and 2008 the number of upper endoscopies performed in Canada has increased by approximately 16% [31]. However, the reason for the endoscopy was not detailed. In order to determine the effectiveness of screening for EAC among GERD patients, the following three key questions (KQs) (Table 1) were addressed through two systematic reviews (SRs) (KQ1 and KQ2) and one overview of reviews (KQ3).

**Table 1:** Key questions

Key question	Question
1a	In adults (≥ 18 years) with chronic gastroesophageal reflux disease (GERD)^a^ with or without other risk factors^b^, what is the effectiveness (benefits and harms) of screening for esophageal adenocarcinoma (EAC) and precancerous conditions (Barrett’s Esophagus (BE) and low- and high-grade dysplasia)? What are the effects in relevant subgroup populations?
1b	If there is evidence of effectiveness^c^, what is the optimal time to initiate and to end screening, and what is the optimal screening interval (includes single and multiple tests and ongoing ‘surveillance’)?
2	In adults with chronic GERD with or without other risk factors,^b^ who have been offered, received, or allocated to receive screening for EAC and precancerous conditions (BE and low- and high-grade dysplasia), how do they weigh the benefits and harms of screening, and what factors contribute to these preferences and to their decisions to undergo screening?
3	What is the effectiveness (benefits and harms) of treatment for stage 1 EAC and precancerous conditions (BE and low- and high-grade dysplasia) in adults?

^a^As defined by study authors

^b^Risk factors will be deemed so by included studies

^c^If there is evidence of at least moderate certainty of evidence of benefit, according to GRADE

## Methods

These SRs were developed, conducted and prepared according to the Canadian Task Force for Preventive Health Care (CTFPHC) Procedure Manual [32] or as methods were updated by the CTPHFC. The protocols for these SRs have been published with PROSPERO (CRD42017049993, CRD42017050014, CRD42018084825) and are available on the CTFPHC website (https://canadiantaskforce.ca/).

These reviews are reported according to the Preferred Reporting Items for Systematic Reviews and Meta-Analyses (PRISMA) statement [33] (Additional file 2) and includes a PRISMA flow diagram for each key question. We also used AMSTAR (A Measurement Tool to Assess the Methodological Quality of Systematic Reviews) for additional quality control [34]. Any amendments made to the protocols when conducting the reviews have been outlined in Additional file 3.

### Analytic frameworks

**Fig. 1:**
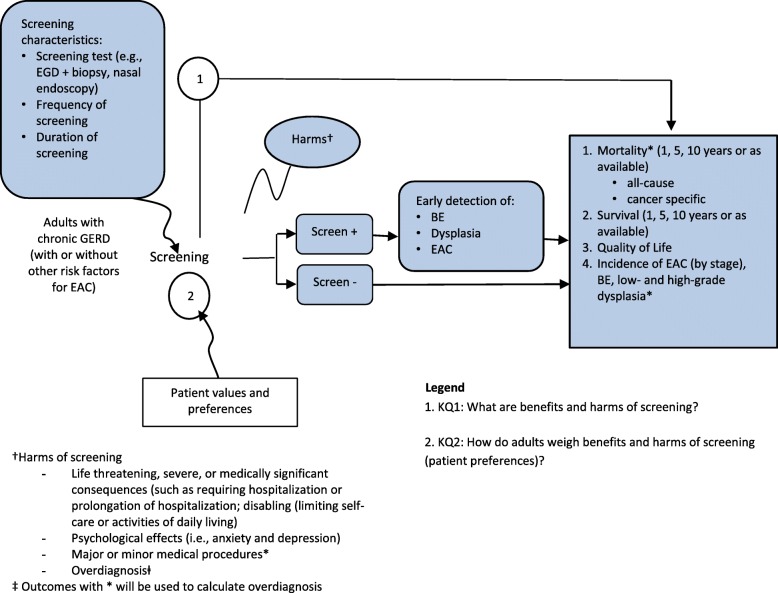
Guideline analytic framework

The analytic framework for these reviews is presented in Fig. 1.

### Inclusion and exclusion criteria

Table 2 presents the eligibility criteria for each KQ, using the PICOTS framework.

**Table 2:** Population, interventions, comparisons, outcomes, timeframe, study design (PICOTS)

		Key question 1	Key question 2	Key question 3
Population	Inclusion	Adults (≥ 18 years old)^a^ with chronic gastroesophageal reflux disease (GERD)^b^ with or without other risk factors^c^ for esophageal adenocarcinoma (EAC).	Adults (≥ 18 years old)^a^ with chronic GERD with or without other risk factors^c^ for EAC who have been offered, received, or allocated to receive screening, depending on the design of the study.	Adults (≥ 18 years old)^a^ with stage 1 EAC, Barrett’s Esophagus (BE) or low- or high-grade dysplasia, with or without chronic GERD as defined in the systematic reviews (SRs)^d^
	Exclusion	- Experiencing alarm symptoms for EAC: dysphagia, recurrent vomiting, anorexia, weight loss, gastrointestinal bleeding or other symptoms identified by authors as ‘alarm’. - Diagnosed with other gastro-esophageal conditions (e.g. gastric cancer, esophageal atresia, other life threatening esophageal conditions) or pre-existing disease (BE, dysplasia, or EAC).		Those diagnosed with other gastro-esophageal conditions (e.g. gastric cancer, esophageal atresia, and other life-threatening esophageal conditions).
Intervention /comparator	Inclusion	KQ1a: - Screening versus no screening - One screening modality versus another screening modality All screening modalities for BE, dysplasia or EAC will be included, such as esophagogastroduodenoscopy (EGD)^e,f^ EGD^f^ plus adjunct techniques^g^, transnasal endoscopy, cytologic examination KQ1b: - One screening modality vs. another screening modality - One interval of screening vs. another interval of screening - Timepoint at which to initiate screening vs. another timepoint - Timepoint at which to cease screening versus. another timepoint	Screening for EAC and other precancerous lesions with any screening modality Depending on study design, comparators may be: - No screening^h^ - Different screening modality - Different screening intervals - Different lengths/duration of screening - Offered screening but did not receive screening - No comparison	Management/ treatment for stage 1 EAC, low- or high-grade dysplasia or BE including: - Pharmacological therapies^i^ - Surveillance methods such as: EGD^e,f^ plus biopsy, EGD^f^ plus biopsy plus adjunct techniquesj - Endoscopic or Endoscopic Assisted therapies^k^ - Surgery, including fundoplication and esophagectomy Comparator: No management/treatment compared to another management/treatment regimen, or a combination of management/ treatment strategies.
	Exclusion	Any follow-up diagnostic tests, such as 24-h esophageal pH test or any test for staging purposes, such as computerized tomography and magnetic resonance imaging.		
Outcomes	Inclusion	Critical for decision-making 1. Mortality—all-cause and EAC-related (1, 5 and 10 year or as available)^l,m^ 2. Survival (1, 5 and 10 year or as available)^l^ 3. Life threatening, severe, or medically significant consequences (such as requiring hospitalization or prolongation of hospitalization; disabling (limiting self-care or activities of daily living) Important for decision-making 4. Incidence of EAC (by stage), BE, low- and high-grade dysplasia^m^ 5. Quality of life (validated scales only; e.g. SF-36, WHOQUAL) 6. Psychological effects (e.g. anxiety and depression) 7. Major or minor medical procedures^m^ 8. Overdiagnosis^n^	1. How patients weigh the benefits and harms of screening (e.g. ranking/rating of benefits and harms outcomes) 2. Willingness to be screened 3. Uptake of screening 4. Factors considered in decision to be screened: what components/outcomes of screening do patients place more value on when deciding whether to be screened or not (e.g. potential complications resulting from screening) 5. Intrusiveness of the screening modality	Critical for decision-making 1. Mortality—all-cause and EAC-related (1, 5 and 10 years, or as available)^l^ 2. Survival (1, 5 and 10 years, or as available)^l^ 3. Progression from non-dysplastic BE to BE with dysplasia, progression from low-grade to high-grade dysplasia, progression to EAC 4. Life threatening, severe, or medically significant consequences (such as requiring hospitalization or prolongation of hospitalization; disabling (limiting self-care or activities of daily living) Important for decision-making 5. Quality of life (validated scales only; e.g. SF-36, WHOQUAL) 6. Major or minor medical procedures 7. Psychological effects (e.g., anxiety, stress) 8. Overtreatment Post-hoc outcomes: 9. Complete eradication of: intestinal metaplasia/BE, dysplasia, high-grade dysplasia, neoplasia 10. Reduction/regression of BE: in length (cm), in area (%) 11. Treatment Failure (no ablation) 12. EAC recurrence
	Timing	No limits	No limits	No limits
	Setting	Settings were limited to primary care or settings in which a primary care physician could refer a patient for esophageal screening.	Primary care or other settings generalizable to primary care.	Any setting.
Study designs	Inclusion	Randomized controlled trials (RCTs), including cluster RCTs. If no or few RCTs (i.e. < 5 trials) are available: Non-RCT, controlled before-after, interrupted times series, cohort studies, case-control studies, limiting to higher levels of evidence depending on the nature and volume of specific study designs. If no or few RCTs are available for the overdiagnosis outcome, ecological and cohort studies will be considered for all outcomes used for the judgement of overdiagnosis.	Randomized controlled trials If insufficient data exists: Controlled clinical trials, controlled before-after, case-controls, cohort, interrupted time series (ITS), and cross-sectional (e.g. surveys) If insufficient data exists for the above: Qualitative studies and mixed-methods studies	Systematic reviews of RCTs^o^ To be defined as a SR, a review must have met all four of the following criteria: (1) searched at least one database; (2) reported its selection criteria; (3) conducted quality or risk of bias assessment on included studies; and (4) provided a list and synthesis of included studies. SRs that identified observational studies were included if results from RCTs were provided separately.
	Exclusion	Cross-sectional studies, case series, case reports, and other publication types (editorials, commentaries, notes, letter, opinions).	Commentaries, opinion, editorials and reviews	SRs that combine results from RCTs with non-RCTs, controlled before-after, interrupted times series, cohort studies, case-control studies, cross-sectional studies, case series, case reports and other publication types (editorials, commentaries, notes, letter, opinions) or SRs that only include non-RCT and observational studies.
	Language	No language restrictions in the search; however, only English and French articles will be included at full-text.		
	Databases	MEDLINE, Embase, Cochrane Library	MEDLINE, Embase, CINAHL, Cochrane Library	MEDLINE, Embase, Cochrane Library (CDSR, DARE, HTA)

^a^Studies addressing both adults and children, if data provided for adults are reported separately

^b^Chronic GERD, as defined by study authors

^c^Risk factors will be as deemed so by included studies

^d^We did not use a predefined method for diagnosis (e.g. histopathological exams, ICD code) and relied on how it was defined in the SRs

^e^Also known as panendoscopy and upper GI endoscopy

^f^With or without biopsy protocol

^g^For example, chromendoscopy and narrow-band imaging

^h^Although we will consider comparative studies that include a no screening arm, we understand that the outcomes of interest do not apply to people who do not receive or have not been offered screening. For such studies, we will only consider data for those who receive or are offered screening

^i^Such as PPI, H2 receptor antagonists, Cox-2 inhibitors, Prokinetics and antacids, NSAIDs

^j^Such as high-definition/high-resolution white light endoscopy, chromoendoscopy, electronic chromoendoscopy, autofluorescensce imaging, confocal laser endomicroscopy, light scattering spectroscopy, diffuse reflectance spectroscopy

^k^Such as ablative techniques (thermal or chemical), and mechanical methods (EMR, ESD or combined options)

^l^From the time of allocation to screening or control arm

^m^These outcomes will be used to judge the extent of overdiagnosis, which is defined as the diagnosis of disease which would never have become clinically apparent in a person's lifetime (i.e., causing neither symptoms nor death)

^n^As judged by the study author or will be judged by the CTFPHC working group using information provided by authors, where available

^o^Systematic reviews that combine RCT and non-RCTs will be included if results for RCTs are provided separately from non-RCT studies

### Literature search

All search strategies (Additional file 4) were developed and tested through an iterative process by an experienced medical information specialist in consultation with the review teams. In addition, the search strategy for the MEDLINE database was peer-reviewed by another experienced librarian using the Peer Review of Electronic Search Strategies (PRESS) checklist [35] (Additional file 5). Table 3 presents an overall description of the searching for each KQ.

**Table 3:** Searching for studies

	Key question 1	Key question 2	Key question 3
Searches^a,b^	Additional file 4. KQ1 searches	Additional file 4. KQ2 searches	Additional file 4. KQ3 searches
Databases	OVID MEDLINE® OVID MEDLINE® Epub Ahead of Print, In-Process and Other Non-Indexed Citations Embase Classic + Embase Cochrane Library on Wiley	Same as KQ1 plus CINAHL using the EBSCO platform	Same as KQ1
Date run	From the inception date on October 29–30, 2018.		
Controlled vocabulary examples^c^	Gastroesophageal reflux, esophageal neoplasms, endoscopy	Gastroesophageal reflux, patient acceptance of health care, informed consent	Barrett esophagus, esophageal neoplasms, meta analysis
Keywords examples^c^	GERD, esophageal cancer, esophagoscopy	GERD, patient perspective, informed decision-making	Barrett’s dysplasia, esophageal cancer, systematic review
Grey literature	CADTH Grey Matters, websites listed in Additional file 6, bibliographies of relevant systematic reviews and clinical practice guidelines identified from the search strategies and grey literature searching.		CADTH Grey Matters plus additional references listed in Additional file 6.

^a^When possible, animal-only and opinion-pieces were removed from the results

^b^The search strategies were peer-reviewed using PRESS 2015 [35] and can be found in Additional file 5

^c^Vocabulary and syntax adjusted across databases, as required

### Study selection

For each KQ, duplicates across searches were identified and removed using Reference Manager [36]. The remaining articles were uploaded into Distiller Systematic Review (DistillerSR) Software© [37] for title and abstract screening and full-text screening of the remaining potential relevant articles.

Reviewers performed a pilot testing phase of randomly selected title and abstracts (*n* = 50) and potentially relevant full-text articles (*n* = 25) prior to commencing broad screening. Screening forms can be found in Additional file 7. Titles and abstracts were independently screened for relevance by two reviewers, using the liberal accelerated method, which requires one user to include for further assessment at full-text and two reviewers to exclude [38]. References were reviewed in random order, with each reviewer unaware if the reference had already been assessed and excluded by the other reviewer. Subsequently, full-texts were retrieved and two reviewers independently assessed the article for relevancy. Conflicts at full-text were resolved by consensus or a third team member. Articles not available for download were ordered from the library through interlibrary loans. Those that were not received within 30 days were excluded and labelled accordingly. For articles with abstracts only, a search was performed to locate any full-text publications.

Where chronic GERD was not defined in a study (KQ1 and KQ2), we attempted to contact the study authors twice over 2 weeks by email to obtain more information. If authors did not respond, and the lack of definition for chronic GERD was the only reason for possible exclusion, we included the study. Reports in abstract form and protocols were coded as such and excluded, but a search was completed to see if the full-text was available. Those that were not available as full-texts were excluded and studies available only in abstract form are available in the list of excluded studies (Additional file 8).

### Data extraction and management

For all KQs, full data extraction was completed by one reviewer using a form developed a priori and 100% of these were verified by a second reviewer (Additional file 9). Any disagreements were resolved by consensus or if needed, with a third reviewer. For KQ1 and KQ2, where information was unclear or missing, authors were contacted by email twice over 2 weeks. If no response was received and the information affected the ability for quantitative analysis, the study was analyzed narratively. For KQ3, data were extracted as they were synthesized and/or reported in the included reviews. No additional information from the primary studies was extracted or assessed and quality control was not performed to verify the accuracy of the reviews’ data on the included studies.

### Risk of bias and quality assessment

For KQ1 and KQ2, all included studies were assessed for the risk of bias (RoB) by one reviewer, with verification completed by a second reviewer. The Cochrane RoB tool [39] was used to evaluate the RoB in RCTs and the Newcastle-Ottawa scale (NOS) [40] was used to evaluate the RoB in cohort studies. For KQ3, the quality of the included SRs was assessed using the AMSTAR measurement tool [41]. Two reviewers assessed the quality of each included SR independently. Any discrepancies were resolved through discussion and if needed, a third reviewer. We used the AMSTAR 2 [42] approach to determine the final assessments of quality of conduct, including consideration of four critical domains and categorized the quality as high, moderate, low or critically low, using the criteria described in Additional file 10. For all assessments, disagreements were resolved by consensus or third party adjudication.

### Analysis

For all KQs, characteristics of the included studies/reviews are presented in tables and summarised narratively. For KQ1, the results are presented in evidence sets 1 to 8 (Additional file 11), with associated forest plots, where applicable. For KQ2, due to the nature of the data, a meta-analysis of outcomes was not appropriate; however, narrative results are presented. For KQ3, the results presented in evidence sets 1-11 (Additional file 12) may omit some results due to overlap. In the case of overlap where outcome data was the same in multiple reviews, the review with the highest methodological quality or with the most complete outcome data was included; the additional reviews are listed in Additional file 12: Table 1 and mentioned in the Notes column within the evidence sets. For KQ3, odds ratios (OR) were commonly used in SRs and absolute risk differences (ARDs) were calculated accordingly. Where SR authors did not provide an OR, a relative risk (RR) was calculated based on the results and the ARD was calculated based on the RR. In instances where the RR did not approximate the OR reported in the SR, we inserted the RR in the notes column in the evidence set; however, the ARDs were calculated based on the OR. We determined the extent of overlap of evidence across reviews by outcome for each comparison using the corrected covered area (CCA) method [43].

#### Meta-analysis

For KQ1, raw data were extracted from all articles, when available. Raw data were entered into Review Manager Software version 5.3 [44] and hazard ratios (HR) were produced for the survival outcome and risk ratios (RR) were calculated for all other outcomes.

#### Subgroup analysis

A priori-defined subgroup analysis (KQ1) variables included age, sex, body mass index (BMI), smoking history, duration of chronic GERD, definition of chronic GERD, groupings of risk factors and various ethnic groups. Reporting did not allow for these to be undertaken.

#### Sensitivity analysis

Sensitivity analyses were planned to restrict to those studies as being low risk of bias (KQ1) based on the overall judgement, to address any decisions made regarding handling of data or to explore statistical heterogeneity (KQ1) and based on the timing of publication (KQ1 and KQ2). However, only two studies were considered low risk of bias and therefore sensitivity analysis was not undertaken.

#### Small study effects

For KQ1 and KQ2, to assess for small study effects, a combination of graphical aids (e.g. funnel plot) and/or statistical tests (e.g. Egger regression test, Hedges-Olkin) were planned if at least ten studies were available in any given analysis. This analysis was not undertaken.

### Rating the certainty of the evidence

For each critical and important outcome, the GRADE framework [32, 45] was used to assess the strength and certainty of the evidence. We followed the GRADE guidance for determining the extent of the risk of bias for the body of evidence [46]. The online software GRADEpro GDT (https://gradepro.org/) was used for the GRADE assessments. Assessment of each GRADE domain (study limitations (i.e. risk of bias), indirectness, inconsistency, imprecision and other considerations (i.e. publication bias and comprehensiveness of the search)) was presented, where possible, with the information provided in the studies. If there was missing information, a narrative description was provided. The certainty of the evidence for each outcome, in each study/review, was rated by one reviewer and verified by a second reviewer. Any discrepancies were resolved through consensus.

As KQ3 is an overview, and there are no published methods for performing GRADE for overviews of reviews, we have used the five domains listed above as a guide. As none of the included reviews used GRADE to evaluate the body of evidence, we performed these assessments using the reported information in the reviews and did not access the primary studies for any additional information, as was pre-specified in the protocol. When undertaking domain assessments, we considered an approach with sufficient face validity to align with GRADE guidance. We have elaborated on considerations and decisions in Additional file 13. As with existing GRADE guidance, each GRADE domain was judged as possessing no serious limitations (no rating down), serious limitations (rating down by one) or very serious limitations (rating down by two).

## Results

**Fig. 2:**
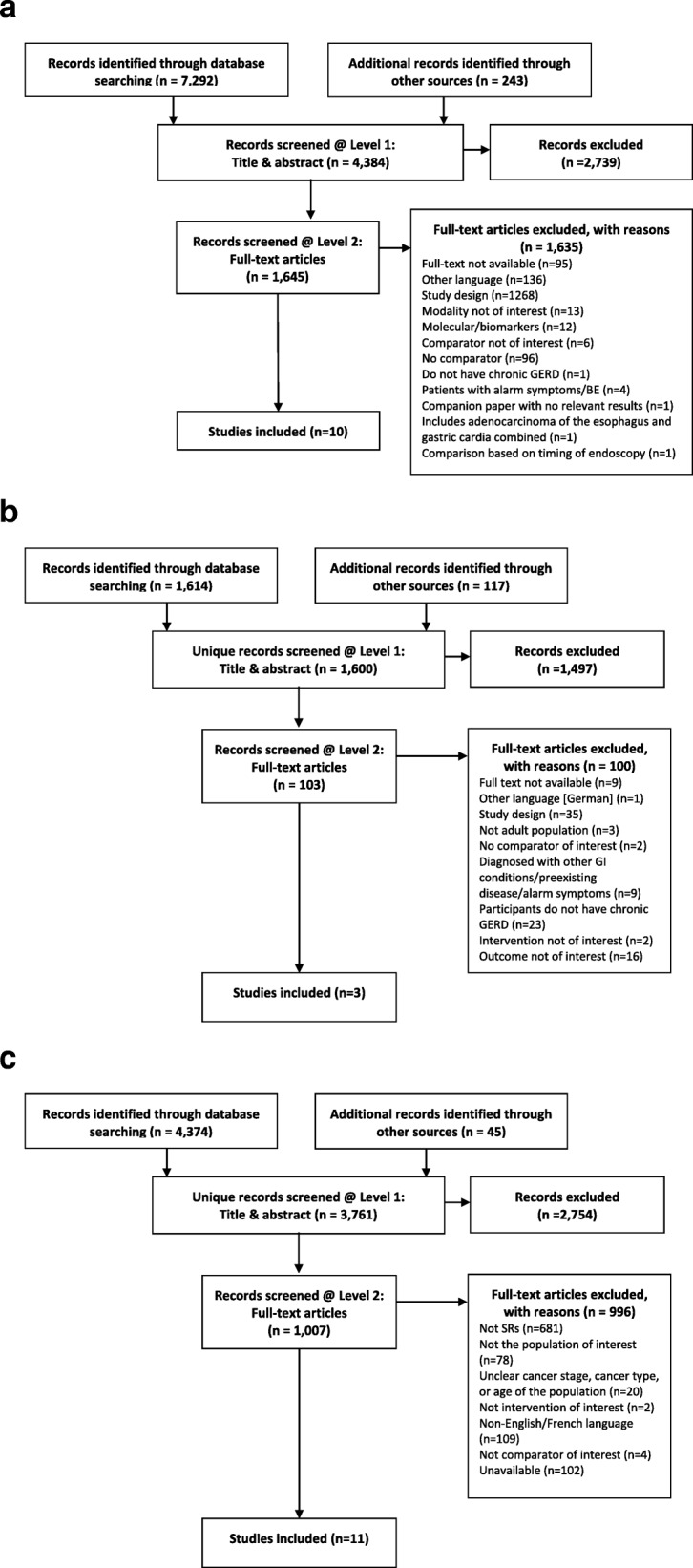
a PRISMA flow diagram for KQ 1. b PRISMA flow diagram for KQ 2. c PRISMA flow diagram for KQ 3

Table 4 provides a summary of the literature search results and Fig. 2a–c shows the PRISMA flow diagrams for each KQ. Study characteristics and population demographics for each key question are presented in Additional file 14 and overall RoB/quality assessment for included studies and reviews are presented in Additional file 15. Additional files 11, 16, 12 provide the evidence set results, narrative results, GRADE evidence profiles and GRADE summary of findings tables for KQ1, KQ2 and KQ3, respectively. The results presented herein provide a high level overview of the results. For additional details of the individual studies and reviews within each section, the full SRs can be found on the CTFPHC website (www.canadiantaskforce.ca). Additional file 8 provides a list of excluded studies at full-text, with reasons for each KQ. A list of ongoing studies for all KQs are provided in Additional file 17.

**Table 4:** Summary of studies/reviews

	Key question 1	Key question 2	Key question 3
Literature search (PRISMA flow diagrams in Fig. 2a–c)			
Initial search results	7,292	1,614	4,374
Deduplication, grey lit and suppl. searching^a^	4,384 (evaluated at title and abstract)	1,600	3,761
Evaluated at full-text	1645	103	1007
# of included studies	10 (6 RCTs, 1 randomized cross-over trial, 1 prospective cohort, 2 retrospective cohort)	3 (2 RCTs, 1 cohort)	11 SRs (10 reporting results) which included 25 articles reporting results of RCTs (1 to 16 per review) (Figs. 3 and 4)
Study characteristics (Full tables in Additional file 14: Tables S1-S3)			
Comparisons	- Screening in the last 5 years with EGD vs no screening [88, 90] - Screening modality (e.g. conventional EGD) vs. screening modality (transnasal esophagoscopy) –[80, 81, 83, 85–87] - Biopsy method (e.g. Four-quadrant random) vs biopsy method (chromoendoscopy) [82, 84]	- Transnasal esophagoscopy vs. Video capsule esophagoscopy - Transnasal-EGD vs. Peroral-EGD - Peroral-EGD and sedated EGD	- Celecoxib vs. Placebo - Omeprazole vs. Histamine Type 2 Receptor Antagonists - PDT + Omeprazole vs. Omeprazole - Anti-reflux surgery (Nissen fundoplication) + APC vs Anti-reflux surgery (Nissen fundoplication) +Surveillance (endoscopic) - Radiofrequency ablation + Proton pump inhibitor vs. Proton pump inhibitor - Anti-reflux surgery (Nissen fundoplication) vs. H2RA/Omeprazole - PDT using 5-ALA vs. PDT using Photofrin - PDT with different treatment parameters - RFA vs Surveillance (endoscopic) - APC + PPI vs. MPEC + PPI - PDT vs. APC + PPI - Endoscopic mucosal resection vs. RFA
Country of conduct	8 USA; 1 India; 1 Japan	3 USA	1 Brazil; 1 China; 5 UK; 4 USA
Years published	1999–2018	1998, 1999, 2014	2008–2018
Study size	- 20 to 92 participants per screening modality - 60–378 participants (RCTs) - 1580 participants (prospective cohort) - 153 and 155 participants (retrospective cohorts)	62, 105 and 1210 participants	- 9 to 208 participants across SRs (most with < 100 participants) - One SR reported on an ongoing RCT with no results [108]
Population demographics (Full tables in Additional file 14)			
Sex	Men: 42–99%	These were not reported in the included studies.	Many of these were not reported across reviews.
Ethnicity	White: 41–99%^b^		
Mean age	Mean age: 48–67 years old		
Smokers	43%, 80%		
PPI use	17%, 48%, 78%		
BMI	29.0 to 31.4^c^		
Outcomes not reported	- All-cause or cause-specific mortality - Quality of life - Major or minor medical procedures - Overdiagnosis	- How patients weigh the benefits and harms of screening - Factors considered in decision to be screened - Intrusiveness of the screening modality	- EAC-related mortality - Quality of life - Overtreatment
Overall RoB	- 2 studies were low RoB for histologically confirmed BE - Overall, outcomes across comparisons were at moderate or high RoB for both RCTs and observational studies (Additional file 15: Tables S1 and 2)	- 1 study rated as high RoB (Additional file 15: Table S3)	- 2 SRs were rates as low quality and 8 were critically low (Additional file 15: Table S4) - Multiple tools used to evaluate the primary RCTs included in the SRs, with the majority rated as unclear or high RoB (Additional file 15: Table S5)
Overall GRADE	All outcome for all comparison were rated as very low certainty.	GRADE was not performed for this KQ.	Evidence for most outcomes was rated as very low certainty or were given a range of ‘very low to low’ (description of ranges in Additional file 13).

APC: Argon Plasma Coagulation; Multipolar electrocoagulation; PDT: Photodynamic Therapy; PPI: Proton Pump Inhibitor; RoB: risk of bias

^a^ Bibliography search, search for full-text articles based on abstracts and protocols

^b^Reported in five studies

^c^Reported in four studies

^a^Including double counting

### Key question 1. Effectiveness of screening

Detailed characteristics tables for the ten included studies can be found in Additional 14: Table 1, and results are described herein. The certainty of the evidence to answer KQ1a was very low; therefore, KQ1b was not addressed.

#### EGD versus no prior EGD

Two retrospective cohort studies by Rubenstein 2008 [47] and Hammad 2019 [48] studied a group of individuals with EAC and evaluated their electronic medical records or the institutional cancer registry to examine if they had a standard sedated esophagogastroduodenoscopy (EGD) in the five years prior to cancer diagnosis or not (Additional file 11: Evidence Set 1). In Rubenstein 2008, survival data, reported using a Kaplan-Meier curve, showed no difference between survival rates at year 1 and 10 [47]. Authors report that there was no difference in long-term survival (approximately 6 to 12 years) between those who had received a prior EGD and those who had not (adjusted HR 0.93, 95% confidence interval (CI) 0.58 to 1.50) [*very low certainty*]. It was difficult to determine a range of effects across studies for survival analyses as the Hammad 2019 study only had one eligible patient with a prior EGD in the past 5 years.

Both Rubenstein et al. [47] and Hammad 2019 [48] reported information to evaluate whether an EGD in the previous five years influenced the incidence of EAC by stage of diagnosis at time of detection. It was difficult to determine a range of effects across studies for most stage-based analyses as one study only had one eligible patient with a prior EGD and the stage of diagnosis was unknown (author correspondence) [48]. Rubenstein et al. [47] reported that there may be a higher odds of a stage 1 diagnosis than a more advanced diagnosis (stages 2–4) (OR 2.77, 95% CI 1.00 to 7.67; *p* = 0.0497; *Forest Plot 1.1*) [*very low certainty*].

#### EGD versus TNE

Four studies evaluated EGD (sedated) compared to unsedated transnasal esophagoscopy (TNE) (RCTs by Chang 2011 [49] and Sami 2015 [50]; a randomised crossover study by Jobe 2006 [51]; one cohort study by Mori 2010 [52]) (Additional file 11: Evidence Set 2). Sami 2015 [50] evaluated safety, defined as serious adverse events (life-threatening, severe or medical significant consequences of screening), and reported no serious adverse events in either group [*very low certainty*].

Jobe et al. [51] reported on incidence of EAC only on those who were receiving initial screening (i.e. excluding those who were being followed with BE). There were no cases of EAC reported [*very low certainty*]. Three studies [49, 50, 52] defined incidence of endoscopically suspected BE differently. The RCTs showed no significant difference between screening modalities; RR 1.90, 95% CI 0.19 to 19.27 [49] and *p* = 0.37 [50] [*very low certainty*]. However, Mori 2010 [52] (cohort study) did show a significant difference, with those being screened with TNE having a higher incidence of suspected BE (RR 2.09, 95% CI 1.30 to 3.36; *Forest Plot 2.1*) [*very low certainty*]. Two studies reported no difference in incidence of histologically confirmed BE between screening modalities; *p* = 0.44 [50] and RR 0.89, 95% CI 0.59 to 1.33 [51] [*very low certainty*]. Incidence of dysplasia was low, with zero in Chang 2011 [49] and nine (EGD: 5; TNE: 4) in Jobe 2006 [51] showing no difference between screening modalities (RR 1.54, 95% CI 0.44 to 5.44; *Forest Plot 2.2*) [*very low certainty*].

Chang 2011 [49], Sami 2015 [50] and Jobe 2006 [51] used the same measurement tool to measure anxiety (psychological effects); however, there were differences in when the tool was utilized and the reporting of the outcomes were different (e.g. mean, median, level of severity). Therefore, no meta-analysis was performed. There was no difference in anxiety before the procedure (*p* = 0.084) [51] [*very low certainty*], less anxiety overall during the insertion (*p* = 0.0001) [51] [*very low certainty*] and during the procedure (*p* < 0.001 [50] and *p* = 0.0001 [51]) for those who received EGD compared to TNE [*very low certainty*].

#### EGD versus video capsule esophagoscopy

One RCT by Chang 2011 [49] evaluated three outcomes, all with *very low certainty* (Additional file 11: Evidence Set 3). There was no difference in the incidence of endoscopically suspected BE between screening modalities (RR 0.57, 95% CI 0.11 to 3.01; *Forest Plot 3.1*). Participants with suspected BE based on video capsule esophagoscopy (VCE) (swallowed device) were offered EGD and BE was confirmed through biopsy. Of the three participants with suspected BE who received VCE, none were histologically confirmed cases of BE. There was also no incidence of dysplasia among either group.

#### EGD versus transoral-EGD

One cohort study by Mori 2010 [52] allowed participants to choose between three screening modalities (sedated EGD, unsedated TNE and unsedated transoral-EGD presented here) (Additional file 11: Evidence Set 4). Overall, there was no difference in the frequency, distribution or severity in the incidence of endoscopically suspected BE between modalities in those with grade 2 or 3 BE (RR 1.30, 95% CI 0.83 to 2.03; *Forest Plot 4.1*) [*very low certainty*].

#### TNE versus VCE

Two studies, Chak 2014 [53] and Chang 2011 [49], provided data on four outcomes (Additional file 11: Evidence Set 5). There was no difference between screening modalities for incidence of endoscopically suspected BE (RR 0.86, 95% CI 0.29 to 2.56; *Forest Plot 5.1*) [*very low certainty*], [49, 53] or for those with histologically confirmed BE (RR 0.62, 95% CI 0.15 to 2.52) [*very low certainty*] [53]. Chang 2011 [49] reported that there were no incidences of dysplasia with either screening modality [*very low certainty*].

Those in the unsedated TNE group experienced more anxiety, nervousness or worry (psychological effects) before the procedure than those in the swallowed VCE group (RR 2.28, 95% CI 1.33 to 3.88; *Forest Plot 5.2*) [53] [*very low certainty*], and anxiety during the procedure (RR 2.14, 95% CI 1.22 to 3.77; *Forest Plot 5.3*) [53] [*very low certainty*].

#### Unsedated TNE versus unsedated transoral EGD

One RCT by Zaman 1999 [54] randomised participants with upper gastrointestinal (GI) symptoms. Mori 2010 [52] (cohort) included those who had previously been screened for upper intestinal tract disorders, and allowed participants to choose between three screening modalities (Additional file 11: Evidence Set 6). Only one complication (life-threatening, severe, or medically significant consequence) was reported (facial swelling followed by surgical exploration and full recovery), with no differences between screening modalities (RR 4.04, 95% CI 0.17 to 95.20; *Forest Plot 6.1*) [*very low certainty*] [54].

Zaman et al. [54] reported no difference between screening modalities in the incidence of endoscopically suspected BE (three cases total) (RR 0.68, 95% CI 0.07 to 7.09; *Forest Plot 6.2*) [*very low certainty*]. Mori et al. [52] reported a significant difference in the frequency of BE, with those screened with TNE less likely to have suspected BE (grade 2 or 3) compared to transoral EGD (RR 0.62, 95% CI 0.41 to 0.94; *Forest Plot 6.3*) [*very low certainty*].

Zaman et al. [54] evaluated the levels of anxiety before the procedure, during insertion, and during the procedure (psychological effects). Anxiety was assessed on a scale of 10 (higher score representing higher level of anxiety), with no significant difference between levels of anxiety at any time (*Forest Plots 6.4*-*6.6*) [*very low certainty*].

#### Random biopsy versus enhanced magnification-directed endoscopy biopsies (with acetic acid)

One RCT by Ferguson 2006 [55] included patients who received standard sedated EGD, with those with suspected BE randomised at that point to different biopsy methods (Additional file 11: Evidence Set 7). As all participants were evaluated on suspected BE through EGD, only incidence of histologically confirmed BE is reported. There was no difference in the incidence of histologically confirmed BE between different methods of biopsy. This was found in both those with pattern III and IV specialized intestinal metaplasia (RR 0.98, 95% CI 0.59 to 1.64; *Forest Plot 7.1*) [*very low certainty*] and among all specialized intestinal metaplasia pattern types (RR 1.14, 95% CI 0.71 to 1.82; *Forest Plot 7.2*) [*very low certainty*].

#### Random biopsy versus chromoendoscopy

One RCT by Wani 2014 [56] included participants who were given conventional EGD (*n* = 378) and those with suspected BE who were randomised to either random biopsy (*n* = 33) or chromoendoscopy (*n* = 23) (Additional file 11: Evidence Set 8). There was no difference in the number of participants with histologically confirmed BE between methods (RR 0.87; 95% CI 026–2.90; *Forest Plot 8.1*) [*very low certainty*].

### Key question 2. Patient values and preferences

Three studies (Chak 2014 [53], Zaman 1999 [54] and Zaman 1998 [57]) provided information on reasons why participants were unwilling to be part of the study or reasons for deciding against the uptake of screening once allocated [53]. Objectives of the included studies were to determine the acceptance and tolerability of different screening modalities and provide data on screening results. Studies reported on those who refused participation prior to study commencement (i.e. either prior to being screened or prior to randomisation), but did not provide participant characteristics on this patient subset. A narrative summary of the results is provided herein, with detailed results in Additional file 16. No studies provided results on how patients weight the benefits and harms of screening, factors considered in decision to be screened or intrusiveness of the screening modality.

#### Willingness to be screened

All three studies provided reasons on why those asked had refused to be screened/participate in the study. A large proportion of these individuals were in one study [53] with 1026 of the 1210 people asked not participating, and 184 who agreed to participate. Among those who did not participate during the invitation period, 627 (52%) did not return the phone call or respond to the letter, 385 (32%) refused to participate (with no reason provided), 12 (1%) were ineligible and two (0.2%) did not participate because of difficulty getting to the hospital. The other two studies by Zaman et al invited 105 outpatients in one study and 62 in the other. Zaman 1999 [54] reported 45 of 105 (43%) patients were unwilling to participate in the study comparing transnasal to peroral EGD. Zaman 1998 [57] reported 19 of 62 (31%) patients unwilling to participate in the study comparing peroral to sedated EGD.

The main reason unwillingness to be screened in both studies was due to anxiety, with 17% (18/105) [54] and 19% (12/62) [57] of all those asked to participate reporting this. Both studies also reported that a fear of gagging was the reason, with 10% (10/105) [54] and 5% (3/62) [57] reporting this as the reason. Lastly, not being interested in the study (10/105, 10%) [54], not wishing to undergo a transnasal procedure (7/105, 7%) [54] and unwillingness to be a study subject (4/62, 6%) were also reported [57].

#### Uptake of screening

Chak 2014 [53] reported seven individuals (of 184 randomised) who did not receive the allocated intervention after randomisation. Five people randomised to the TNE group did not receive the procedure because they wanted capsule instead. Two people randomised to the VCE group did not receive the procedure because they were worried about the capsule getting stuck. There was no statistically significant difference in uptake between intervention groups (*p* = 0.25).

### Key question 3. Treatment

**Fig. 3:**
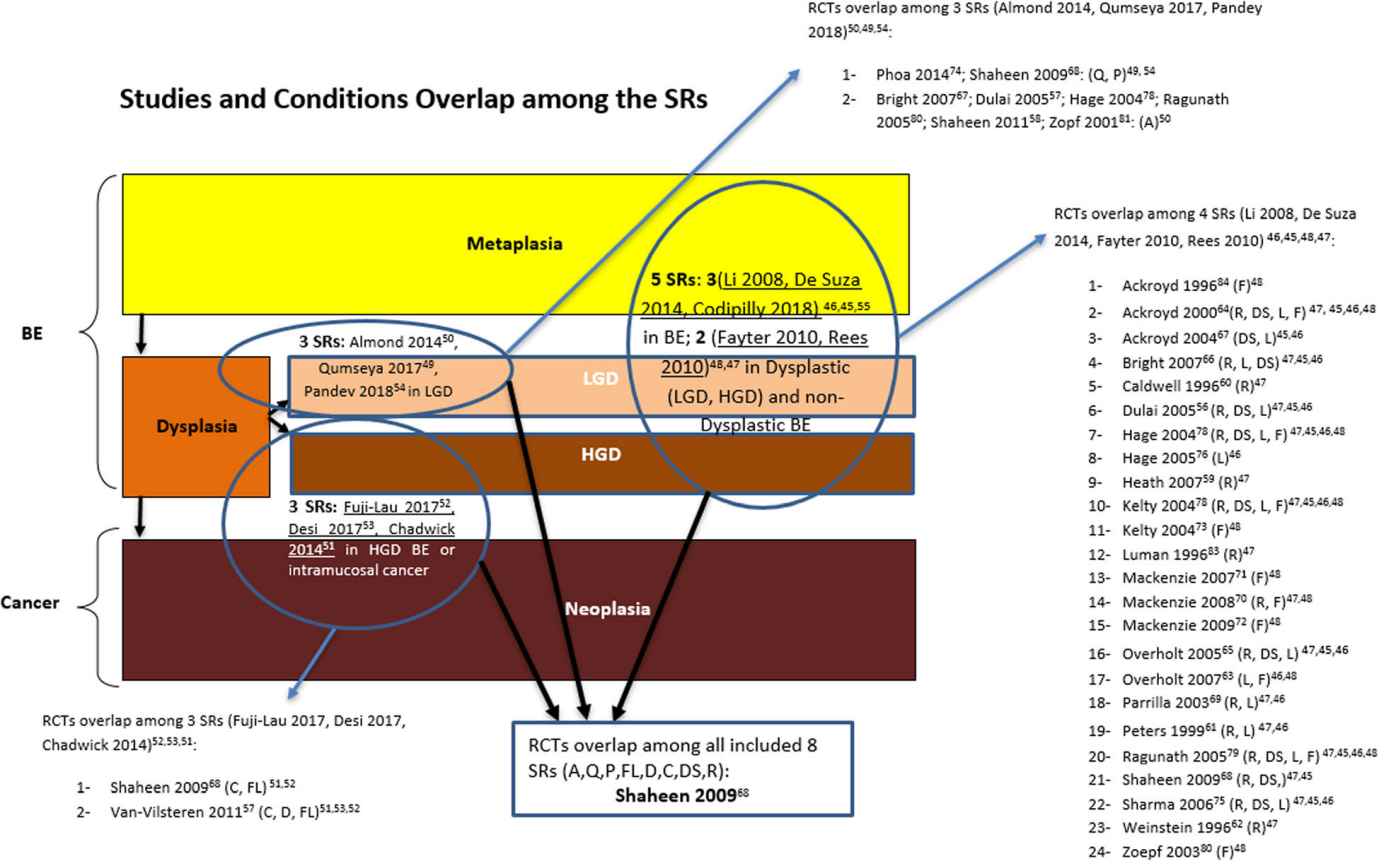
Primary studies and conditions overlap among the systematic reviews

**Fig. 4:**
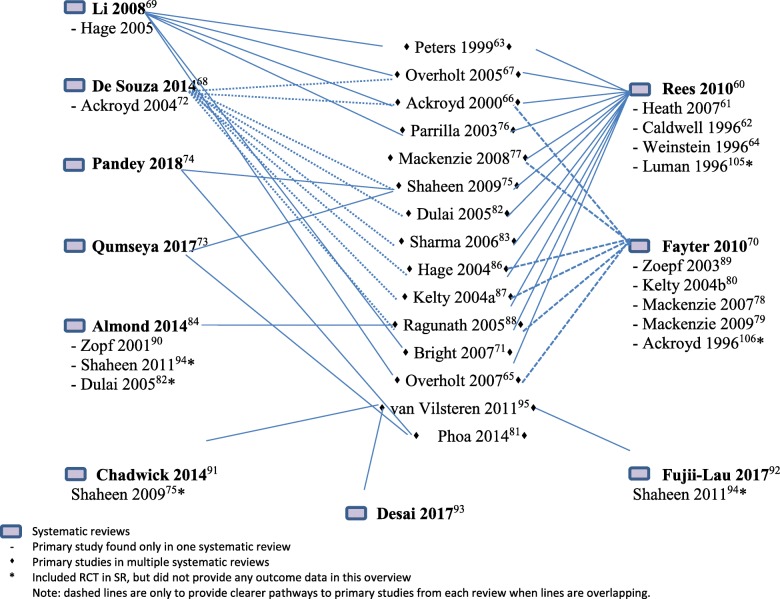
Map of Systematic Reviews and Primary RCTs

The review characteristics of the 11 included SRs are shown in Additional file 14: Table 3. Additional file 12: Table 1 provides additional details of all primary studies included in each SR, and which treatment comparisons provided results in each SR, respectively. Additional file 12: Evidence Sets 1-11 provides detailed results and GRADE tables. Some of the individual trials were represented in more than one review since the reviews did not have mutually exclusive eligibility criteria (Figs. 3 and 4). Twenty-two sets of comparisons had overlapping data across reviews (Additional file 18). In most cases, included studies overlapped completely, according to corrected covered area (CCA) calculations. In few cases, there was discordance among reviews. Throughout the Evidence Sets 1-11, the word “significance” refers to statistical significance unless stated otherwise.

#### Celecoxib versus placebo

Rees 2010 [58] included one primary RCT [59] and reported no difference between the groups for all-cause mortality [*low certainty*] and progression to adenocarcinoma at one year [*very low certainty*] (three cases per group) (Additional file 12: Evidence Set 1.1). For all-cause mortality, there is discordant reporting within the review, where the text reports two deaths in the trial, but the forest plot reports three deaths in each group. Not presented in the results table but presented narratively in the SR, review authors stated that the primary trial authors did not report any statistical difference for the following outcomes: the area of BE segment at 12 months, and in the reduction in the number of patients progressing from intestinal metaplasia to dysplasia between baseline and 1-year. In addition, review authors reported “no statistical difference in the number of patients” with complete eradication of dysplasia at 12 months, and with bleeding in each group.

#### Omeprazole versus histamine type 2 receptor antagonists

Rees 2010 [58] reported data from three primary studies [60–62], and one was only an abstract [60]. The three studies had differences with regards to drug dosage and regimens (Additional file 14: Table 4). Results and GRADE ratings are presented in Additional file 12: Evidence Set 2.1. There was no difference in the reduction in length (cm) of BE at 12 months between the compared groups, and the pooled effect estimates for both the overall and subgroups (*I*^2^ statistic = 62.6% and 60%, respectively) remained non-significant when the analysis was restricted to a subgroup who received a higher dose of omeprazole [*very low certainty*] [61, 62]. There was a small change in the reduction in area (%) of BE with omeprazole that was statistically significant at 12 months [*very low to low certainty*] [61, 62].

#### Photodynamic therapy + omeprazole versus omeprazole alone

Two unique [63, 64] trials (from three studies) [63–65] reported across four SRs [58, 66–68] reported on patients with BE. Overholt 2007 [63] provided 5-year follow-up data for progression to EAC, with Overholt 2005 [65] providing 2-year follow-up data for other outcomes for the same trial participants (Additional file 12: Evidence Set 3.1). Overholt 2005 [65] and Ackroyd 2000 [64] reported on all-cause mortality, using photodynamic therapy (PDT) with either 5-ALA or porfimer sodium, respectively. Overholt et al. reported no statistically significant difference between groups, but this was based on few observed events (*n* = 3) and Ackroyd et al. observed no deaths [*very low certainty*].

At both two- (OR 0.38, 95% CI 0.18 to 0.77) [65] and five- (RR 0.53, 95% CI 0.31 to 0.91) [63] years, there was a statistically lower progression to EAC with combined therapy than with omeprazole alone [*very low to low certainty*]. Progression from non-dysplastic to dysplastic BE was statistically lower with combined therapy (*n* = 0) compared to the omeprazole group (*n* = 12) [*very low certainty*] [64].

Both reviews show higher eradication of dysplasia with combined therapy [*very low to low certainty*]; however, there were some data discrepancies between reviews [58, 67] for both studies [64, 65]. Li 2008 [67] provided data among those with HGD from the same studies as the eradication of dysplasia outcome. It is unclear why more participants experienced eradication of HGD than dysplasia in general, as the denominators are the same. There was higher eradication with PDT combined with Omeprazole [*very low to low certainty*]. Overholt 2007 [63] reported that eradication of BE by 5 years was statistically greater with combined therapy (OR 14.18, 95% CI 5.38 to 37.37) [*very low to low certainty*].

One study with 36 participants (reported in three reviews) reported on reduction/regression of BE using various measures [58, 67, 68]. Statistically significant reductions in both length and area of BE were observed with combined therapy [64] in two reviews [*very low certainty*] [58, 67]. Fayter et al. [68] provided results of evidence of regression (not further described), with much higher percentage of those in the combined group experiencing regression (89% vs. 11%) [*very low certainty*].

There were fewer absolute treatment failures of BE with combined therapy [*very low certainty*] [64, 65].

Statistically significantly more strictures formed with combined therapy (49/138) compared to the omeprazole treatment group (0/70) in one study [*very low to low certainty*] [65].

#### Anti-reflux surgery + Argon plasma coagulation versus anti-reflux surgery + surveillance (endoscopic)

Three systematic reviews [58, 66, 67] reported data from a single trial with two publications [69, 70] on six outcomes (Additional file 12: Evidence Set 4.1). Nissen fundoplication was used for anti-reflux surgery. Ackroyd 2004 [70] was a short-term follow up of the patients, with longer-term follow up presented in Bright 2007 [69]. No patients progressed to cancer [*very low certainty*] [69]. Based on sparse events (two instances in the surveillance group) in Bright 2007 [69] (in Li 2008 [67]), no difference between the treatment effects was observed for progression to HGD (from LGD) [*very low certainty*]. Bright 2007 [69] provided 5-year follow-up data for progression from intestinal metaplasia to dysplasia, and reported no difference between the two groups (two cases of progression in the surveillance group) [*very low certainty*] [58, 69].

The effect estimate favoured Argon plasma coagulation (APC) [69] at 12 months for complete eradication of BE [*very low certainty*]. Note: the data presented in the forest plot differed from the data in the text [58, 69]. No difference was observed between the treatment groups for complete ablation (among those with histological change) [69] in Li 2008 [*very low certainty*]. Ackroyd 2004 [70] in De Souza 2014 [66] reported that no difference in treatment failure was observed between the compared groups [*very low certainty*].

#### Radiofrequency ablation + proton pump inhibitor versus PPI alone

Three systematic reviews [58, 71, 72] reported data from Shaheen 2009 [73] (Additional file 12: Evidence Set 5.1). Rees et al. [58] included patients with both low- and high-grade dysplasia; however, Qumseya 2017 [71] and Pandey 2018 [72] restricted their reporting to patients with low-grade dysplasia. Five participants progressed to EAC at 5 years or at the latest timepoint of follow-up (RFA + PPI: 1/84; PPI: 4/43) [58], resulting in no difference between the compared treatments [*low certainty*]. Among those with LGD, none progressed to EAC over the follow-up period [*low* (Rees 2010) *and very low certainty (*Qumseya 2017)] [58, 71].

Fewer patients progressed to higher grades of dysplasia with the radiofrequency ablation (RFA) treatment [*low certainty*] [58]. However, there is discrepancy in how this outcome is labelled in the review. The text says there was no data for those progressing from IM to dysplasia and labels it as progression to higher grades of dysplasia, but the forest plot is titled progression from IM to dysplasia. When the outcome was restricted to progression to HGD among patients with LGD, no difference was observed [*very low certainty*] [71, 72].

There was a statistically significant difference favouring RFA for complete clearance of intestinal metaplasia (RR 17.81, 95% CI 2.61–121.54) [*very low certainty*] [72], for complete clearance of dysplasia (OR 22.67, 95% CI 8.72 to 58.94) [58] [*low certainty*], which remained when restricted to patients with LGD (OR 0.03, 95% CI 0.01–0.13) [*very low certainty*] [72], and for complete eradication of BE (OR 143.53, 95% CI 18.53–1113.87) [*low certainty*] [58]. De Souza 2014 [66] showed higher rate of treatment failure in the proton pump inhibitor (PPI) treatment group compared to the RFA + PPI group (RFA + PPI: 19/84; PPI: 42/43) [*very low certainty*].

There was no difference between treatment effects for stricture formatio [58] [*very low certainty*]. There were no instances of perforation reported [72] [*very low certainty*], and only one study participant developed bleeding, but data was not presented per arm [72] [*very low certainty*].

#### Anti-reflux surgery (Nissen fundoplication) versus H2 receptor agonist/omeprazole

Two systematic reviews [58, 67] reported data from Parrilla 2003 [74] on five outcomes. Overall, the certainty of the evidence was *very low* for all outcomes (Additional file 12: Evidence Set 6.1). No deaths (all-cause mortality) were reported in either group [58].

Few participants progressed to EAC, with two in each group (not statistically significant) [58]. Rees 2010 [58] reported a significant difference in the incidence of progression to dysplasia from intestinal metaplasia, with less progression in the surgical treatment group compared with the pharmacological treatment group. Although Li et al. [67] included the same primary study, the incidence in the surgery group differed from Rees et al., and demonstrated no significant difference between the groups [58, 67]. Because different data were reported for the intervention groups, this led to discordant results between reviews.

Although some participants experienced eradication of dysplasia (surgery: 5/58, H2 receptor antagonist/omeprazole: 3/43) at 5-year follow-up, this was not statistically different between treatment groups [58]. None of the participants experienced complete eradication of BE at 5 years in either treatment group [58].

#### PDT with 5-aminolevulinic acid versus PDT with porfimer sodium

MacKenzie 2008 [75] in Rees 2010 [58] reported preliminary data only in abstract form and recruitment had not yet been completed. The certainty of evidence was *very low* for both outcomes (Additional file 12: Evidence Set 7.1). There was no statistically significant difference in eradication of HGD between the treatment groups (preliminary results included 14 patients in each treatment group) [75].

These preliminary results showed no difference between treatment groups in stricture formation.

#### Photodynamic therapy with different treatment parameters

A SR by Fayter 2010 [68] with three primary studies [76–78], one of which was an abstract [76], compared different parameters in the PDT treatment. The certainty of the evidence was *very low* for cancer risk, and ranged from very low to low for the remaining four outcomes (Additional file 12: Evidence Set 7.2). Generally, higher doses and red light had lower cancer risk and lower rates of adenocarcinoma [76]. These results were considered significant, but were taken from an abstract, so should be interpreted with caution.

#### Radiofrequency ablation versus surveillance (endoscopic)

Phoa 2014 [79] reported in two systematic reviews [71, 72], included patients with BE with low-grade dysplasia. These reviews also included another primary study by Shaheen et al. [73]; however, results from this study are presented in Evidence Set 5.1 as another review [58] states that both treatment groups also received pharmacological therapy (Additional file 12: Evidence Set 8.1). There were seven people with progression to EAC (RFA: 1/68, Surveillance: 6/68) [*very low certainty*]. Progression per patient-year is also presented [*very low certainty*]. Qumseya 2017 [71] reported data as cumulative progression from LGD to HGD [*very low certainty*] and progression per patient-year [*very low to low certainty*]. Few events were observed (RFA: 0, Surveillance: 12). Pandey 2018 [72] demonstrated a marginally statistically significant results favouring RFA (RR 0.03, 95% CI 0.00 to 0.44) [*very low to low certainty*] [72]. Although Pandey and Qumseya reported discrepant data for the surveillance group in the number of patients with progression to HGD, 18 and 12, respectively, effect estimates are similar between reviews.

RFA resulted in more patients with complete eradication of dysplasia (RR 3.52, 95% CI 2.40 to 5.17) [*very low to low certainty*] [72]. A favourable treatment effect was observed with RFA for complete eradication of intestinal metaplasia (RR 123.30, 95% CI 7.78 to 1954.10) [*very low to low certainty*] [72].

Eight strictures were formed among the study population; however, data was not reported per arm [*very low to low certainty*] [72]. None of the study patients developed perforations [*very low to low certainty*] [72], and only one study participant developed bleeding, but data was not reported per group [*very low to low certainty*] [72].

#### Argon plasma coagulation + PPI versus multipolar electrocoagulation + PPI

Rees 2010 [58] reported on two primary studies (Additional file 12: Evidence Set 9.1) [80, 81], with no instances of mortality (all-cause) reported [very low to low certainty] and one case of stricture formation in the Argon plasma coagulation (APC) + PPI group [*very low certainty*].

#### Multipolar electrocoagulation + PPI versus Argon plasma coagulation + PPI

Two SRs [66, 67] reported the same two primary studies as Evidence Set 9.1; however, the intervention and comparison groups are reversed (Additional file 12: Evidence Set 9.2) [80, 81]. Both outcomes are presented as one review provided the pooled OR (OR 2.01, 95% CI 0.77 to 5.23) [*very low certainty*] for histological complete ablation of BE [67] and the other provided the pooled risk difference (RD − 0.14, 95% CI − 0.33 to 0.05) [*very low certainty*] for treatment failure (the opposite of complete ablation). Both favour multipolar electrocoagulation (MPEC) + PPI [66].

#### Photodynamic therapy versus Argon plasma coagulation + PPI

Five systematic reviews [58, 66–68, 82] reported on six primary studies [83–88] of which some were abstracts (e.g. Zoepf 2003 [87]) (Additional file 12: Evidence Set 10.1). There were many differences between the SRs and the primary studies within the SRs in how comparison groups were reported, heterogeneity between therapy types (e.g. PDT with 5-ALA or Porfimer sodium), differences in their drug dosing and light delivery regimens [58] and differences in the participants who were included in the analyses (e.g. all levels of dysplasia or LGD only). Rees 2010 [58] reported on three studies [84–86], with a combined incidence of all-cause mortality of one in the PDT group and none in the APC + PPI group [*very low certainty*] [84].

Almond 2014 [82] reported on three studies [84, 86, 88] in participants with LDG. One incident case of EAC by 12 months in the PDT group was reported [*very low certainty*]. Almond et al. [82] reported no events of progression to high-grade dysplasia among 17 participants [*very low certainty*] [84, 86].

Rees 2010 [58] and Almond 2014 [82] show discrepant data for the PDT group in Ragunath et al. [86]. The number of patients experiencing complete eradication of dysplasia was reported as 10/13 in Rees 2010, and 8/11 in Almond 2014 [*very low certainty*]. As Almond et al. included only those with low-grade dysplasia, it might be that the two additional participants in Rees et al. had high-grade dysplasia, although this is not clearly reported. Five SRs [58, 66–68, 82] reported on PDT versus APC + PPI and how it affected BE in five primary studies [83–87]. These reviews reported the outcomes in several ways: complete ablation of BE, eradication of BE, reduction of BE (length, surface reduction) and treatment failure (no ablation). Overall, there was a high level of heterogeneity among studies and in the results with *very low certainty* in all of these outcomes except the reduction in length (cm) which was rated as *low certainty*. Determining concordance of results across reviews was difficult due to the differences in how information was reported. Almond 2014 [82] reports on Ragunath 2005 [86], reporting no difference between treatments in eradication of intestinal metaplasia (two participants in each group) [*very low certainty*].

Both Rees 2010 [58] and Almond 2014 [82] reported on stricture, with Rees 2010 including three primary studies [84–86] and Almond 2014 only including Ragunath 2005 [86]. Although there was discordance in the number of those experiencing stricture, neither review reported any difference between treatment groups [*very low certainty*].

#### Endoscopic mucosal resection versus radiofrequency ablation

Three SRs [89–91] included patients with BE and intramucosal neoplasia (i.e. early stage adenocarcinoma). Although both Fujii-Lau et al. [90] and Chadwick et al. [89] include Shaheen 2011 [92] as an included study, because only one of the treatment groups was considered relevant for those reviews, neither reported the results from the placebo group. Therefore, results from Shaheen 2011 [92] are not presented (Additional file 12: Evidence Set 11.1). All three reviews provided results for both treatment groups for the primary study of van Vilsteren 2011 [93], although all three reviews also label the treatment groups differently (e.g. stepwise EMR vs. focal EMR + RFA, EMR vs. RFA, complete EMR vs. RFA). Both endoscopic mucosal resection (EMR) and radiofrequency ablation (RFA) eradicated neoplasia (eradication of cancer) in most cases (EMR: 100%; RFA: 96%), with no difference between treatments [*very low certainty*] [91]. Eradication of dysplasia was completed in almost all participants at the end of the treatment and at follow-up. Only one participant in the RFA group did not have complete eradication at the end of treatment and follow-up [*very low certainty*] [89]. Almost all participants experienced complete eradication of intestinal metaplasia, although there was slight discordance among the percentages reported in the two reviews [*very low certainty*] [89, 91].

Only one participant in the EMR treatment group experienced recurrence of cancer [*very low certainty*] [90], no participant experienced recurrence of dysplasia [*low certainty*] [90] and two participants in each treatment group experienced recurrence of intestinal metaplasia [*very low certainty*] [90].

Two SRs [89, 91] reported on bleeding, with some data discrepancies, but overall concordant results. One SR [89] reported that among the 25 participants in the EMR group, only one participant experienced perforations. No one in the RFA group experienced this outcome. Most participants receiving EMR treatment experienced strictures (22 of 25, 88%) compared to only three of 22 (14%) in the RFA group. Review authors did not provide effect estimates, but a risk ratio of 6.45 (95% CI 2.23 to 18.66) for EMR compared to RFA was calculated using these data [91]. Almost all participants receiving EMR experienced stenosis requiring treatment (88%, 22/25), with only three of 21 (14%) experiencing stenosis in the RFA group [89]. This difference was statistically significant with a calculated risk ratio of 6.45 (95% CI 2.23–18.65) for EMR compared with RFA. All of these adverse events were rated as *very low certainty*.

## Discussion

Esophageal cancer, although lower in incidence relative to other cancers, has a higher mortality rate, partly due to a more advanced stage at diagnosis, when the cancer is widely spread to other vital organs and is incurable. This makes the consideration of whether to invest in screening services important. In 2012, a Cochrane systematic review by Yang et al. [94] set out to include only RCTs comparing screening versus no screening, and found no studies meeting their inclusion criteria. Five years later, this systematic review found no additional randomised controlled trials comparing screening to no screening. Among the few studies that have assessed the effectiveness of screening of individuals with chronic GERD, there exists several limitations (e.g. small sample sizes, one-time screening test with no follow-up). Although there may be higher odds of stage 1 diagnosis if an EGD had been performed in the previous 5 years, the study included a small number of cases, resulting in low precision [47]. Those diagnosed at earlier stages (T1 and T2) can be treated with potentially curable therapies, for example, esophagectomy in patients with high-grade dysplasia and stage T1a cancer has been associated with a greater survival; 89% at 1 year, 77% at five years and 68% at 10 years [95]. Comparatively, those with late stage cancer that cannot be cured by surgery receive chemotherapy/chemoradiation and have a 15% 5-year survival rate [2].

There was little difference in the incidence rates of EAC, BE and dysplasia using alternative screening methods. Although EGD with biopsy is considered the gold standard for the diagnosis and surveillance of BE [96, 97], the results from these studies may encourage increased usage of alternative methods of screening for BE and EAC. Conventional EGD uses sedation, which increases the cost of screening (e.g. monitoring patients post-procedure) and resources used (e.g. availability of a gastroenterologist, recovery room). Alternate methods do not require sedation, can be done in a primary care setting and require little monitoring post-procedure. In studies where participants who had experienced a previous screening and were allowed to then select which screening modality they wanted, there was a preference towards unsedated methods. Of the 1574 participants, 721 (46%) chose transnasal, 599 (38%) chose transoral and 254 (16%) chose EGD [52]. Further supporting patient choice of screening modality, RCTs reported higher levels of dropouts and anxiety among those randomised to TNE compared to other screening modalities, although not always significant. The perceived discomfort of the unsedated transnasal procedure could contribute to increased anxiety.

When considering patient values and preferences for screening, the data is also sparse. Three studies reported on the willingness, or in this case the unwillingness, to participate and be screened in a study on screening for EAC and precancerous conditions. One study also provided outcome information on uptake of screening, more specifically reasons why they did not uptake screening after allocation. No other outcomes of interest were addressed in these studies, overall providing little evidence to answer the KQ2. We are not aware of any other reviews that have been done in the area of upper GI screening in relation to how patients weigh the benefits and harms of screening and what factors contribute to these preferences and to their decision to undergo screening, so there is nothing to compare it to.

In our overview evaluating treatment for BE, with or without dysplasia, and early-stage adenocarcinoma (KQ3), 11 SRs were included. Treatment modalities covered pharmacological therapy, various ablative techniques, surgery and some combinations thereof, with a mix of statistically significant and non-significant results, meaning that treatment may show an effect on some outcomes and little to no effect on others. However, there were few studies, all with small sample sizes by outcome, and for many outcomes, only one study provided results, thereby providing little information with which to gauge the certainty of the evidence. In consultation with clinical experts, in addition to evidence from retrospective and prospective clinical series (e.g. AIM trial [92]), and registry data, certain treatments are currently considered as the standard of care. For example, BE with HGD should be treated with ablation and T1a esophageal cancer (EAC and ESCC) should be treated with endoscopic resection (either endoscopic mucosal resection or endoscopic submucosal resection).

## Limitations

Both reviews and the overview of reviews were developed using rigorous methodological standards, as detailed a priori in registered protocols. There may, however, still be some limitations. There is a risk of missing studies, although we minimized this risk by searching multiple databases and using several techniques to search for grey literature. We included only English and French language studies, and some studies were excluded because we could not get access to the full text (i.e. not available through open access journals or through interlibrary loans). There is a chance that some of these records may have met the inclusion criteria and provided additional results. In KQ3, most records (68%) were excluded during our screening phase due to not meeting the pre-defined SR definition [98]. Reason for exclusion were mainly lack of quality assessment of primary studies and not a study design of interest (either a narrative review or clinical practice guideline based on a non-systematic literature review). Consequently, there is a chance that our conclusions may not be reflective of the totality of relevant, existing evidence. Updating the evidence base is an important research agenda item. Among those that did meet our pre-defined definition, some were excluded because they only included observational studies, or did not separate results of RCTs from observational studies.

When evaluating the results for the effectiveness of screening (KQ1), given the very low certainty of the evidence, true effects may be substantially different or uncertain in light of limitations in the body of evidence. There were several important methodological limitations leading to a moderate or high risk of bias among all study outcomes. The few included studies, and generally small sample sizes leads to imprecise results that could not be assessed for consistency or publication bias. A trend that may continue in this area, as half of the potentially relevant ongoing trials are expecting sample populations of less than 200 participants (Additional file 17). Blinding of participants to screening modality was not possible in these studies. The inability to blind patients could affect psychological outcomes, as a patient might have a preference to one screening modality over another. When evaluating the results for patient values and preferences (KQ2), it was difficult to accurately assess RoB for these studies, as the primary purpose of the included studies was to evaluate acceptability after screening and effectiveness of the screening modality, a different lens to the context of our review. Most outcome data were collected before randomisation, and as there is no formal tool to assess RoB prior to randomisation, these outcomes were not assessed. Measurement bias may be present, as studies did not clearly state how this outcome data were collected. It is not clear how the data were collected among those who refused participation during the consent period, as there is no mention of questionnaires or if and how study personnel collected this information. Only the uptake of screening outcome in one study stated that a non-completion questionnaire was given to ascertain reasons for non-completion. It was difficult to assess the inconsistency among the included studies, mainly due to a lack of information among the studies contributing to outcome results. For example, the largest study invited 1210 participants, with 38% (385/1026) of those declining to participate not providing any information on why they refused. Poor reporting of patient information for those who contributed outcome data was seen in all studies. None reported on the age and sex of these participants, and indication for screening (as described above), making it difficult to understand how comparable these studies might have been. Similarly, the quality of the evidence for treating BE, dysplasia and early-stage cancer (KQ3) was low or very low across the comparisons and outcomes, indicating uncertainty that the observed effects would be representative of the true underlying effect. Poor reporting was a barrier in assessing all domains. Additionally, items within tools such as the Jadad score and Downs & Black do not directly translate to considerations that GRADE guidance suggests for assessing risk of bias. The current limited evidence originated in 11 poorly conducted reviews (two rated as low quality and nine rated as critically low quality), from small RCTs (published between 1996 and 2011 with one published in 2014) with unclear or high risk of bias with short follow-up times. Multicenter trials are needed to increase the power of the evidence base. The lack of a longer patient follow-up time to inform outcomes may be explained by patient retention issues or the cost of following patients long-term.

The lack of a definition of chronic GERD, or even how studies defined GERD, leads to a serious concern for the direct generalizability of the population represented in these studies to the target population of this review. Among studies that did provide a description on how GERD was defined, not all studies used a validated questionnaire to define GERD, while some defined GERD inclusion based on “typical symptoms”. Some studies did not define GERD at all. A standardized definition of chronic GERD would allow trialists to better identify the population of interest. Additionally, as more data accrue, this may lead to more certainty as to whom the evidence would apply (i.e. directness) and with greater precision of the estimate and better quality of conduct (and reporting).

Several outcomes of interest, including mortality, quality of life and overdiagnosis, were not reported in any of the included studies (KQ1). This is mostly because the study results were cross-sectional in nature and these outcomes would require follow-up. In the absence of the outcomes of interest to calculate overdiagnosis, we were unable to address this. The review on patient values and preferences (KQ2) did not provide any results on how patients weight the benefits and harms of screening, factors considered in decision to be screened and intrusiveness of the screening modality. When evaluating treatment options (KQ3), not all outcomes that were considered critical or important were reported. Only one review [58] reported on mortality, and five of ten reviews with usable data reported on progression to cancer, although at different time periods. Survival, quality of life, psychological effects and overtreatment were not reported in any of the included reviews. Additionally, outcomes that were reported have been reported using several different methods. For example, BE was reported as complete eradication, regression or reduction (e.g. regression in cm, regression in area), making it difficult to combine and compare results across studies. One review [66] reported the outcome as “treatment failure” which is the opposite of eradication but provides another opportunity for reporting core outcomes across reviews. A quick search by our research team on the Core Outcome Measures in Effectiveness Trials (COMET) web-based repository of core outcome sets (http://www.comet-initiative.org/studies/search) revealed none for BE; the one available for esophageal cancer was in relation to chemotherapy, radiation therapy and surgery, presumably for later-stage cancers. Although we developed our outcomes list a priori with review by various stakeholders, it would be a worthwhile endeavour to formally develop a core outcomes list to inform the conduct of future trials. The outcomes used in this review could be used to start discussions on developing core outcomes in this area. Due to the lack of a core outcome list, the pre-specified protocol missed some of the outcomes that were added post-hoc as we encountered them during screening and assessment of the reviews. Those core outcomes sets can help with consistency of outcome definition and terminology, an issue that was encountered in our reviews for all key questions.

Treatments have also changed over time. For instance, photodynamic therapy is used less frequently, according to clinical expert experience, and there were some treatment options listed in Additional file 1 that were not evaluated (e.g. cryotherapy, endoscopic submucosal resection). This might be because they are newer techniques (not yet included in published SRs) or considered less relevant options to include in a SR. Future SRs may consider conducting network meta-analyses of available treatment options, if there are additional primary studies in this area that have been conducted since the last search date of the included reviews.

Potentially relevant, unpublished trials were identified from our grey literature search and may prove informative for any subsequent updates of these reviews (Additional file 17). The ongoing BEST3 cluster randomised controlled trial in the UK involves 120 primary care practices with a planned sample of 9000 participants. The aims are to assess whether the Cytosponge test for patients with reflux symptoms will be effective in increasing the detection of BE in primary care compared to usual care, and to evaluate cost-effectiveness and patient acceptability. However, only the planned outcomes of the incidence of BE (KQ1), adverse events (KQ1) and patient acceptability (KQ2) may be relevant. Results are anticipated for late 2019.

## Implications for research

The current literature contains several methodologic flaws and issues around the certainty of the evidence, which limits our ability in considering the applicability of the evidence. The low quality of reporting in this area is of concern, as it influences the true effects of the interventions, and helps in understanding the similarity across studies and the applicability of the findings in SRs. It underscores the importance of proper, clear and transparent reporting for trials using the CONSORT statement [99] and for SRs using the PRISMA checklist [33]. Several journals have endorsed and incorporated the CONSORT statement in their instructions to authors. Specialty journals for gastroenterology should consider this approach in order to improve the quality of reporting of RCTs and SRs [100]. We also encourage authors of future SRs to perform GRADE and transparently report information for each domain. This is consistent with recommendations by the REWARD Alliance to reduce research waste and increase its value (http://rewardalliance.net/about/recommendations/), which states that “Investigators, funders, sponsors, regulators, research ethics committees, and journals should systematically develop and adopt standards for the content of study protocols and full study reports, and for data sharing practices”.

A consistent and transparent definition of “chronic GERD” should be developed, which would help update these SRs and guide medical professionals in which patients should be screened for EAC and precancerous lesions. A consistent classification of BE (e.g. Prague Classification) should also be used to allow for comparability between studies.

Most included studies compared one screening method versus another screening method. As EAC is a rare disease, to more accurately assess screening effectiveness, good quality, large multi-centre (including community hospitals and university centres) RCTs on screening versus no screening should be performed. These studies should perform follow-up of the participants over time, with clearly defined pathways, to evaluate EAC-related mortality, survival time and other critical outcomes better reported and defined, as discussed by the Core Outcome Measures in Effectiveness Trials (COMET) initiative [101].

Overdiagnosis has not been addressed in the current literature. de Gelder 2011 [102] outlines seven approaches to estimating overdiagnosis, depending on choice of numerator and denominator. Individual patient data in studies to test the seven approaches would be ideal. This could provide additional information to a study by Pohl 2005 where it was concluded that overdiagnosis in EAC should be excluded as an explanation for the rise in incidence [103].

Incorporating a systematic evaluation of patient values and preferences into the evidence considered in developing guidelines is important, and primary studies must contribute to this knowledge base. In addition, considering the gender perspective is important in recruitment strategies, should a screening program ever exist. Researchers can access national initiatives such as the Strategy for Patient-Oriented Research (SPOR) in Canada and the Patient Centered Outcomes Research Institute (PCORI) in the USA, who provide funding in research to integrate patient involvement, which is believed to lead to greater use and uptake of the research results. Additionally, INVOLVE, established in the UK in 1996, supports active public involvement in public and social care research.

## Conclusion

The evidence on the effectiveness (benefits and harms) of screening for EAC and precancerous conditions (BE and dysplasia) is sparse and is of very low certainty, making it difficult to conclude whether or not people with chronic GERD should be screened for EAC and precancerous lesions. More and better designed trials are needed and a definition of what is considered chronic GERD should be developed to help identify a patient group where screening can be better targeted to evaluate the effectiveness. Similarly, there is currently insufficient evidence to make firm conclusions on how adults with chronic GERD weigh the benefits and harms of screening, and what factors contribute to these preferences and to their decisions to undergo screening. As the importance of this area is well documented, there is a critical information gap that requires new primary research. Lastly, many treatment modalities for BE have been evaluated in the SR literature, but available evidence is of low or very low quality for most outcomes. Due to several limitations (poorly reported low-quality SRs, unclear or high risk of bias trials with small sample sizes, few studies per treatment modality), there is uncertainty in the effectiveness of these treatments. Large multicentre trials with longer follow-up are needed.

## Supplementary information

**Additional file 1:** List of treatment options.

**Additional file 2:** PRISMA checklist.

**Additional file 3:** Amendments to the protocol.

**Additional file 4:** Search strategies

**Additional file 5:** PRESS checklists.

**Additional file 6:** Grey literature searching.

**Additional file 7:** Screening forms.

**Additional file 8:** List of excluded studies.

**Additional file 9:** Data extraction variables.

**Additional file 10:** Additional details of RoB and Quality Assessment Methods.

**Additional file 11:** KQ1 evidence sets.

**Additional file 12:** KQ3 evidence sets.

**Additional file 13:** GRADE considerations and decisions for the overview of reviews (KQ3).

**Additional file 14:** Characteristics tables

**Additional file 15: **ROB and quality appraisal results

**Additional file 16:** KQ2 results.

**Additional file 17:** List of potentially relevant ongoing studies.

**Additional file 18:** Overlap and concordance.

## Data Availability

Not applicable.

## Abbreviations

ACG: American College of Gastroenterology
ACP: American College of Physicians
AGA: American Gastroenterological Association
AMSTAR: A Measurement Tool to Assess Systematic Reviews
APC: Argon plasma coagulation
ARD: Absolute risk difference
BE: Barrett’s esophagus
BMI: Body mass index
CADTH: Canadian Agency for Drugs and Technologies in Health
CAG: Canadian Association of Gastroenterology
CI: Confidence interval
COMET: Core Outcome Measures in Effectiveness Trials
CTFPHC: Canadian Task Force on Preventive Health Care
EAC: Esophageal adenocarcinoma
EGD: Esophagogastroduodenoscopy
EME: Enhanced magnification-directed endoscopy
EMR: Endoscopic mucosal resection
ESCC: Esophageal squamous cell carcinoma
GERD: Gastroesophageal reflux disease
GI: Gastrointestinal
GRADE: Grading of Recommendations, Assessment, Development and Evaluation
HGD: High-grade dysplasia
HR: Hazard ratio
LGD: Low-grade dysplasia
MD: Mean difference
MPEC: Multipolar electrocoagulation
NOS: Newcastle-Ottawa Scale
NR: Not reported
OR: Odds ratio
P-EGD: Peroral endoscopy
PDT: Photodynamic therapy
PICOS: Population, Interventions, Comparisons, Outcomes, Study design
PPI: Proton pump inhibitors
PRESS: Peer Review of Electronic Search Strategies
PRISMA: Preferred Reporting Items for Systematic Reviews and Meta-Analyses
RCT: Randomised controlled trial
RFA: Radiofrequency ablation
RoB: Risk of bias
RR: Risk ratio
SD: Standard deviation
SR: Systematic review
TNE: Transnasal esophagoscopy
Transoral-EGD: Transoral esophagoscopy
VCE: Video capsule esophagoscopy
