# Supplementary material for: Screening for esophageal adenocarcinoma and precancerous conditions (dysplasia and Barrett’s esophagus) in patients with chronic gastroesophageal reflux disease with or without other risk factors: two systematic reviews and one overview of reviews to inform a guideline of the Canadian Task Force on Preventive Health Care (CTFPHC)
Source: Syst Rev. 2020 Jan 29;9:20. doi: 10.1186/s13643-020-1275-2 (PMC6990541; doi:10.1186/s13643-020-1275-2)
Supplement: Supplementary file 1 — Additional file 1: List of treatment options. [file 13643_2020_1275_MOESM1_ESM.docx]

# Additional file 1. List of treatment options

**Pharmacological therapies**, such as:

- Proton pump inhibitors therapy
- H2 receptor antagonists
- Cyclo-oxygenase-2 inhibitors
- Prokinetics and antacids
- Non-steroidal anti-inflammatory drugs (NSAIDs)

**Surveillance** (primarily diagnostic procedures to enhance early detection):

- High-definition/high-resolution white light endoscopy
- Chromoendoscopy
- Electronic chromoendoscopy
- Autofluorescence imaging
- Confocal laser endomicroscopy
- Light scattering spectroscopy, diffuse reflectance spectroscopy

**Endoscopic or Endoscopic Assisted therapies**:

- Ablative techniques (eliminate all dysplastic mucosa)
- Thermal: Argon plasma coagulation (APC), Multipolar electrocoagulation (MPEC), Radiofrequency ablation (RFA), Cryotherapy/cryoablation, Laser ablation
- Chemical: Photodynamic therapy (PDT)
- Mechanical methods (remove targeted superficial tissue of the GI tract)
- Endoscopic mucosal resection (EMR)
- Endoscopic submucosal dissection (ESD)
- Combined options (i.e., EMR + PDT, PDT + PPI)

**Surgery**:

- Laparoscopic anti-reflux surgery (i.e., fundoplication)
- Esophagectomy
