# Supplementary material for: Screening for esophageal adenocarcinoma and precancerous conditions (dysplasia and Barrett’s esophagus) in patients with chronic gastroesophageal reflux disease with or without other risk factors: two systematic reviews and one overview of reviews to inform a guideline of the Canadian Task Force on Preventive Health Care (CTFPHC)
Source: Syst Rev. 2020 Jan 29;9:20. doi: 10.1186/s13643-020-1275-2 (PMC6990541; doi:10.1186/s13643-020-1275-2)
Supplement: Supplementary file 3 — Additional file 3: Amendments to the protocol. [file 13643_2020_1275_MOESM3_ESM.docx]

# Additional file 3. Amendments to the protocol

## Key Question 1

A predefined definition of chronic GERD described in the protocol was: (1) symptoms for ≥12 months, with no specific frequency; and/or (2) PPI (or other pharmacotherapy) use for GERD for ≥12 months. The timing of symptoms for ≥12 months which was used to be over-inclusive as a scoping exercise resulted in few studies that defined chronic GERD. Using the pre-defined definition of chronic GERD would have resulted in no included studies. The definition has been expanded to include what study authors considered chronic GERD and reflected this uncertainty in our interpretation of the findings (i.e., in the GRADE assessments).

We originally intended to include lower quality study designs only if there were five or fewer RCTs. Although there were six included RCTs, all relevant studies were included as there were few studies under each comparison and only two observational studies were located.

## Key Question 3

Data for additional relevant outcomes were extracted and included in this overview. Outcomes defined *a priori* only included progression; however, as the review is on treatment, other outcomes such as eradication/regression, reduction, and recurrence were considered relevant as well. It was stated in the protocol that AMSTAR assessments would be done by one reviewer, with verification by a second reviewer. However, these assessments were done independently, in duplicate, with conflicts resolved through discussion or with a third reviewer. We used the AMSTAR 2 approach, relating to the four critical domains, to come up with final categorization of the quality of conduct as noted in RoB and quality assessment section.
