# Supplementary material for: Screening for esophageal adenocarcinoma and precancerous conditions (dysplasia and Barrett’s esophagus) in patients with chronic gastroesophageal reflux disease with or without other risk factors: two systematic reviews and one overview of reviews to inform a guideline of the Canadian Task Force on Preventive Health Care (CTFPHC)
Source: Syst Rev. 2020 Jan 29;9:20. doi: 10.1186/s13643-020-1275-2 (PMC6990541; doi:10.1186/s13643-020-1275-2)
Supplement: Supplementary file 4 — Additional file 4: Search strategies [file 13643_2020_1275_MOESM4_ESM.docx]

# Additional file 4. Search strategies

## KQ1 Searches

Date Ran: 2018 Oct 29

Database: Embase Classic+Embase <1947 to 2018 October 26>, Ovid MEDLINE(R) ALL <1946 to October 25, 2018>

Search Strategy:

--------------------------------------------------------------------------------

1 exp Gastroesophageal Reflux/ (84749)

2 ((esophageal or gastric* or gastro-esophageal or gastro-oesophageal or gastroesophageal or gastrooesophageal or supraesophageal or supra-esophageal or supraoesophageal or supra-oesophageal) adj2 reflux*).tw,kw. (60380)

3 GERD.tw,kw. (23254)

4 GORD.tw,kw. (2189)

5 SEGR.tw,kw. (14)

6 (gastric adj2 regurgitat*).tw,kw. (524)

7 or/1-6 (101149)

8 Esophageal Neoplasms/ (54194)

9 exp Esophagus/ and exp Neoplasms/ (37262)

10 ((esophag* or oesophag* or pharynx-esophag*) adj3 (neoplas* or cancer* or tumour* or tumor* or carcinoma* or malignan* or metasta* or oncolog* or adenoma* or adenocarcinoma* or adeno-carcinoma* or carcinosarcoma* or carcino-sarcoma*)).tw,kw. (115398)

11 Barrett Esophagus/ (23026)

12 (Barrett* adj1 (esophag* or oesophag* or epitheli* or metaplasi* or syndrome?)).tw,kw. (22711)

13 (dysplasia* or dysplastic* or precancer* or pre-cancer* or premalignan* or pre-malignan*).tw,kw. (237615)

14 or/8-13 (381903)

15 7 and 14 [GERD AND ESOPHAGEAL CANCER] (14039)

16 exp Infant/ not (exp Adult/ and exp Infant/) (1653036)

17 exp Child/ not (exp Adult/ and exp Child/) (3188154)

18 15 not (16 or 17) [CHILD-ONLY REMOVED] (13587)

19 exp Animals/ not (exp Animals/ and Humans/) (16928885)

20 18 not 19 [ANIMAL-ONLY REMOVED] (9917)

21 (comment or editorial or interview or news).pt. (1857143)

22 (letter not (letter and randomized controlled trial)).pt. (2039050)

23 20 not (21 or 22) [OPINION PIECES REMOVED] (9328)

24 limit 23 to systematic reviews [Limit not valid in Embase; records were retained] (5438)

25 meta analysis.pt. (93528)

26 exp meta-analysis as topic/ (55716)

27 (meta-analy* or metanaly* or metaanaly* or met analy* or integrative research or integrative review* or integrative overview* or research integration or research overview* or collaborative review*).tw. (320935)

28 (systematic review* or systematic overview* or evidence-based review* or evidence-based overview* or (evidence adj3 (review* or overview*)) or meta-review* or meta-overview* or meta-synthes* or "review of reviews" or technology assessment* or HTA or HTAs).tw. (380695)

29 exp Technology assessment, biomedical/ (23572)

30 (cochrane or health technology assessment or evidence report).jw. (38344)

31 or/25-30 (663092)

32 23 and 31 (237)

33 24 or 32 [REVIEWS] (5478)

34 exp Guidelines as Topic/ (618496)

35 exp Clinical Protocols/ (244747)

36 Guideline.pt. (16000)

37 Practice Guideline.pt. (24370)

38 standards.fs. (661949)

39 Consensus Development Conference.pt. (10837)

40 (guidance* or guideline* or standards or recommendation*).ti. (311034)

41 (expert consensus or consensus statement* or consensus conference* or practice parameter* or position statement* or policy statement* or CPG or CPGs).tw. (111573)

42 or/34-41 (1639102)

43 23 and 42 [GUIDELINES] (344)

44 (controlled clinical trial or randomized controlled trial or pragmatic clinical trial).pt. (558557)

45 clinical trials as topic.sh. (185080)

46 (randomi#ed or randomly or RCT$1 or placebo*).tw. (2078317)

47 ((singl* or doubl* or trebl* or tripl*) adj (mask* or blind* or dumm*)).tw. (382260)

48 trial.ti. (451524)

49 or/44-48 (2552566)

50 23 and 49 [RCTS] (446)

51 controlled clinical trial.pt. (92722)

52 Controlled Clinical Trial/ or Controlled Clinical Trials as Topic/ (564652)

53 (control* adj2 trial*).tw. (536268)

54 Non-Randomized Controlled Trials as Topic/ (10101)

55 (nonrandom* or non-random* or quasi-random* or quasi-experiment*).tw. (110148)

56 (nRCT or nRCTs or non-RCT$1).tw. (1682)

57 Controlled Before-After Studies/ (212209)

58 (control* adj3 ("before and after" or "before after")).tw. (8951)

59 Interrupted Time Series Analysis/ (204342)

60 (time series adj3 interrupt*).tw. (5071)

61 (pre- adj3 post-).tw. (194013)

62 (pretest adj3 posttest).tw. (10024)

63 Historically Controlled Study/ (222590)

64 (control* adj2 stud$3).tw. (488554)

65 Control Groups/ (123900)

66 (control$ adj2 group$1).tw. (1094939)

67 trial.ti. (451524)

68 or/51-67 (3041588)

69 23 and 68 [NON-RCTS] (859)

70 exp Cohort Studies/ (2201726)

71 cohort$1.tw. (1281888)

72 Retrospective Studies/ (1057885)

73 (longitudinal or prospective or retrospective).tw. (2746922)

74 ((followup or follow-up) adj (study or studies)).tw. (111610)

75 Observational study.pt. (53767)

76 (observation$2 adj (study or studies)).tw. (221881)

77 ((population or population-based) adj (study or studies or analys#s)).tw. (36252)

78 ((multidimensional or multi-dimensional) adj (study or studies)).tw. (245)

79 Comparative Study.pt. (1812404)

80 ((comparative or comparison) adj (study or studies)).tw. (234286)

81 exp Case-Control Studies/ (1099012)

82 ((case-control* or case-based or case-comparison) adj (study or studies)).tw. (214189)

83 Cross-Sectional Studies/ (423299)

84 ((cross-sectional or frequency or prevalence) adj (analys#s or study or studies or survey$1)).tw. (426217)

85 or/70-84 (7259652)

86 23 and 85 [OBSERVATIONAL STUDIES] (2373)

87 33 or 43 or 50 or 69 or 86 [ALL STUDY DESIGNS] (7084)

88 87 use medall [MEDLINE RECORDS] (1803)

89 gastroesophageal reflux/ (76189)

90 ((esophageal or gastric* or gastro-esophageal or gastro-oesophageal or gastroesophageal or gastrooesophageal or supraesophageal or supra-esophageal or supraoesophageal or supra-oesophageal) adj2 reflux*).tw,kw. (60380)

91 GERD.tw,kw. (23254)

92 GORD.tw,kw. (2189)

93 SEGR.tw,kw. (14)

94 (gastric adj2 regurgitat*).tw,kw. (524)

95 or/89-94 (94213)

96 exp esophagus tumor/ (77710)

97 exp esophagus/ and exp neoplasm/ (37262)

98 ((esophag* or oesophag* or pharynx-esophag*) adj3 (neoplas* or cancer* or tumour* or tumor* or carcinoma* or malignan* or metasta* or oncolog* or adenoma* or adenocarcinoma* or adeno-carcinoma* or carcinosarcoma* or carcino-sarcoma*)).tw,kw. (115398)

99 Barrett Esophagus/ (23026)

100 (Barrett* adj1 (esophag* or oesophag* or epitheli* or metaplasi* or syndrome?)).tw,kw. (22711)

101 (dysplasia* or dysplastic* or precancer* or pre-cancer* or premalignan* or pre-malignan*).tw,kw. (237615)

102 or/96-101 (388236)

103 95 and 102 [GERD AND ESOPHAGEAL CANCER] (13204)

104 exp juvenile/ not (exp juvenile/ and exp adult/) (2337309)

105 exp Infant/ not (exp Adult/ and exp Infant/) (1653036)

106 exp Child/ not (exp Adult/ and exp Child/) (3188154)

107 or/104-106 (3908908)

108 103 not 107 [CHILD, 17 AND UNDER, REMOVED] (12752)

109 exp animal experimentation/ or exp models animal/ or exp animal experiment/ or nonhuman/ or exp vertebrate/ (47785712)

110 exp human/ or exp human experimentation/ or exp human experiment/ (37610583)

111 109 not 110 (10176834)

112 108 not 111 [ANIMAL-ONLY REMOVED] (12412)

113 editorial.pt. (1053946)

114 letter.pt. not (letter.pt. and randomized controlled trial/) (2034134)

115 112 not (113 or 114) [OPINION PIECES REMOVED] (11719)

116 meta-analysis/ (244188)

117 "systematic review"/ (181694)

118 "meta analysis (topic)"/ (38725)

119 (meta-analy* or metanaly* or metaanaly* or met analy* or integrative research or integrative review* or integrative overview* or research integration or research overview* or collaborative review*).tw. (320935)

120 (systematic review* or systematic overview* or evidence-based review* or evidence-based overview* or (evidence adj3 (review* or overview*)) or meta-review* or meta-overview* or meta-synthes* or "review of reviews" or technology assessment* or HTA or HTAs).tw. (380695)

121 biomedical technology assessment/ (22465)

122 (cochrane or health technology assessment or evidence report).jw. (38344)

123 or/116-122 (719814)

124 115 and 123 [REVIEWS] (466)

125 exp practice guideline/ (496379)

126 (guidance* or guideline* or standards or recommendation*).ti. (311034)

127 (expert consensus or consensus statement* or consensus conference* or practice parameter* or position statement* or policy statement* or CPG or CPGs).tw. (111573)

128 or/125-127 (807229)

129 115 and 128 [GUIDELINES] (406)

130 randomized controlled trial/ or controlled clinical trial/ (1261895)

131 exp "clinical trial (topic)"/ (278077)

132 (randomi#ed or randomly or RCT$1 or placebo*).tw. (2078317)

133 ((singl* or doubl* or trebl* or tripl*) adj (mask* or blind* or dumm*)).tw. (382260)

134 trial.ti. (451524)

135 or/130-134 (2842728)

136 115 and 135 [RCTS] (792)

137 controlled clinical trial/ (551272)

138 "controlled clinical trial (topic)"/ (9690)

139 (control* adj2 trial*).tw. (536268)

140 (nonrandom* or non-random* or quasi-random* or quasi-experiment*).tw. (110148)

141 (nRCT or nRCTs or non-RCT$1).tw. (1682)

142 (control* adj3 ("before and after" or "before after")).tw. (8951)

143 time series analysis/ (21591)

144 (time series adj3 interrupt*).tw. (5071)

145 pretest posttest control group design/ (353)

146 (pre- adj3 post-).tw. (194013)

147 (pretest adj3 posttest).tw. (10024)

148 controlled study/ (6234502)

149 (control* adj2 stud$3).tw. (488554)

150 control group/ (123900)

151 (control* adj2 group$1).tw. (1094939)

152 trial.ti. (451524)

153 or/137-152 (8064899)

154 115 and 153 [NON-RCTS] (2112)

155 cohort analysis/ (640633)

156 cohort$1.tw. (1281888)

157 retrospective study/ (1418098)

158 longitudinal study/ (236664)

159 prospective study/ (965640)

160 (longitudinal or prospective or retrospective).tw. (2746922)

161 follow up/ (1364204)

162 ((followup or follow-up) adj (study or studies)).tw. (111610)

163 observational study/ (205270)

164 (observation$2 adj (study or studies)).tw. (221881)

165 population research/ (95192)

166 ((population or population-based) adj (study or studies or analys#s)).tw. (36252)

167 ((multidimensional or multi-dimensional) adj (study or studies)).tw. (245)

168 exp comparative study/ (3125774)

169 ((comparative or comparison) adj (study or studies)).tw. (234286)

170 exp case control study/ (1099012)

171 ((case-control* or case-based or case-comparison) adj (study or studies)).tw. (214189)

172 cross-sectional study/ (550186)

173 ((cross-sectional or frequency or prevalence) adj (analys#s or study or studies or survey$1)).tw. (426217)

174 or/155-173 (9122540)

175 115 and 174 [OBSERVATIONAL STUDIES] (3847)

176 124 or 129 or 136 or 154 or 175 [ALL STUDY DESIGNS] (5431)

177 176 use emczd [EMBASE RECORDS] (3828)

178 88 or 177 [BOTH DATABASES] (5631)

179 (2016 11* or 2016 12* or 2017* or 2018*).dt. (2466286)

180 88 and 179 [MEDLINE UPDATE RECORDS] (140)

181 (201611* or 201612* or 2017* or 2018*).dc. (3493794)

182 177 and 181 [EMBASE UPDATE RECORDS] (545)

183 180 or 182 [BOTH DATABASES - UPDATE PERIOD] (685)

184 remove duplicates from 183 (573)

185 184 use medall [UNIQUE MEDLINE UPDATE RECORDS] (140)

186 184 use emczd [UNIQUE EMBASE UPDATE RECORDS] (433)

***************************

Cochrane

<https://www.cochranelibrary.com/advanced-search/search-manager?search=2220030>

Date Run: 30/10/2018 03:16:43

ID Search Hits

#1 MeSH descriptor: ["Gastroesophageal Reflux"] explode all trees 1740

#2 ((esophageal or gastric* or (gastro next esophageal) or (gastro next oesophageal) or supraesophageal or (supra next esophageal) or supraoesophageal or (supra next oesophageal)) near/2 reflux*):ti,ab,kw 1064

#3 GERD:ti,ab,kw 1185

#4 GORD:ti,ab,kw 148

#5 SEGR:ti,ab,kw 1

#6 (gastric near/2 regurgitat*):ti,ab,kw 67

#7 {or #1-#6} 2862

#8 MeSH descriptor: ["Esophageal Neoplasms"] explode all trees 1308

#9 MeSH descriptor: [Esophagus] explode all trees 267

#10 ((esophag* or oesophag* or (pharynx next esophag*)) near/3 (neoplas* or cancer* or tumour* or tumor* or carcinoma* or malignan* or metasta* or oncolog* or adenoma* or adenocarcinoma* or (adeno next carcinoma*) or carcinosarcoma* or (carcino next sarcoma*))):ti,ab,kw 3387

#11 MeSH descriptor: ["Barrett Esophagus"] explode all trees 207

#12 (Barrett* near/1 (esophag* or oesophag* or epitheli* or metaplasi* or syndrome*)):ti,ab,kw 471

#13 (dysplasia* or dysplastic* or precancer* or (pre next cancer*) or premalignan* or (pre next malignan*)):ti,ab,kw 3694

#14 {or #8-#13} 7045

#15 #7 and #14 133

#16 MeSH descriptor: [Infant] explode all trees 14928

#17 MeSH descriptor: [Child] explode all trees 1356

#18 #15 not (#16 or #17) with Cochrane Library publication date Between Oct 2016 and Oct 2018 35

DSR – 1

CENTRAL - 34

## KQ2 Searches

Database: Embase Classic+Embase <1947 to 2018 October 26>, Ovid MEDLINE(R) ALL <1946 to October 25, 2018>

Search Strategy:

--------------------------------------------------------------------------------

1 exp Gastroesophageal Reflux/ (84749)

2 ((esophageal or gastric* or gastro-esophageal or gastro-oesophageal or gastroesophageal or gastrooesophageal or supraesophageal or supra-esophageal or supraoesophageal or supra-oesophageal) adj2 reflux*).tw,kw. (60380)

3 GERD.tw,kw. (23254)

4 GORD.tw,kw. (2189)

5 SEGR.tw,kw. (14)

6 (gastric adj2 regurgitat*).tw,kw. (524)

7 or/1-6 [GERD] (101149)

8 Esophageal Neoplasms/ (54194)

9 exp Esophagus/ and exp Neoplasms/ (37262)

10 ((esophag* or oesophag* or pharynx-esophag*) adj3 (neoplas* or cancer* or tumour* or tumor* or carcinoma* or malignan* or metasta* or oncolog* or adenoma* or adenocarcinoma* or adeno-carcinoma* or carcinosarcoma* or carcino-sarcoma*)).tw,kw. (115398)

11 Barrett Esophagus/ (23026)

12 (Barrett* adj1 (esophag* or oesophag* or epitheli* or metaplasi* or syndrome?)).tw,kw. (22711)

13 (dysplasia* or dysplastic* or precancer* or pre-cancer* or premalignan* or pre-malignan*).tw,kw. (237615)

14 or/8-13 [ESOPHAGEAL CANCER] (381903)

15 7 and 14 [GERD AND ESOPHAGEAL CANCER] (14039)

16 exp Infant/ not (exp Adult/ and exp Infant/) (1653036)

17 exp Child/ not (exp Adult/ and exp Child/) (3188154)

18 15 not (16 or 17) [CHILD-ONLY REMOVED] (13587)

19 exp Animals/ not (exp Animals/ and Humans/) (16928885)

20 18 not 19 [ANIMAL-ONLY REMOVED] (9917)

21 (comment or editorial or news).pt. (1829540)

22 (letter not (letter and randomized controlled trial)).pt. (2039050)

23 20 not (21 or 22) [OPINION PIECES REMOVED] (9328)

24 exp Gastroesophageal Reflux/px (414)

25 Esophageal Neoplasms/px (212)

26 Barrett Esophagus/px (30)

27 Mass Screening/px (2080)

28 Early Detection of Cancer/px (915)

29 Diagnostic Tests, Routine/px (87)

30 Endoscopy/px (71)

31 Endoscopy, Gastrointestinal/px (80)

32 Esophagoscopy/px (17)

33 Gastroscopy/px (66)

34 or/24-33 [PSYCHOLOGICAL ASPECTS RE: DISEASE AND SCREENING TECHNIQUES] (3775)

35 exp Adaptation, Psychological/ (175170)

36 Attitude/ (105292)

37 Attitude to Death/ (25924)

38 exp Attitude to Health/ (482770)

39 Choice Behavior/ (207942)

40 Consumer Advocacy/ (6368)

41 *Consumer Behavior/ (10238)

42 exp Consumer Participation/ (85545)

43 Cooperative Behavior/ (75296)

44 Decision Making/ (290020)

45 Focus Groups/ (209410)

46 Health Care Surveys/ (39038)

47 Health Services Accessibility/ (198940)

48 Interviews as Topic/ (183260)

49 Life Change Events/ (46828)

50 Narration/ (20684)

51 Patient Acceptance of Health Care/ (93677)

52 Patient Advocacy/ (44216)

53 exp Patient-Centered Care/ (748675)

54 exp Patient Education as Topic/ (186333)

55 Patient Participation/ (47277)

56 Patient Preference/ (21341)

57 Patient Satisfaction/ (197656)

58 exp Patients/px (15527)

59 Personal Autonomy/ (28669)

60 *"Power (Psychology)"/ (66398)

61 Questionnaires/ (882190)

62 Quality of Life/px (22058)

63 exp Self Concept/ (289574)

64 Self Efficacy/ (66626)

65 exp Self-Help Groups/ (23022)

66 Social Values/ (96087)

67 ((accept* or anxiet* or anxious* or attitud* or consider* or choice? or choos* or chose? or concern* or decid* or decis* or dissatisf* or expect* or experienc* or fear* or feel* or felt or input* or opinion* or participat* or perceiv* or percepti* or perspective? or prefer* or respons* or satisf* or unsatisf* or value? or valuing or view* or worrie? or worry*) adj3 (citizen? or client? or consumer? or female? or male? or men or patient? or public or stake?holder* or user? or wom#n)).tw,kf. (1783010)

68 (advoca* adj3 (client? or consumer? or patient?)).tw,kf. (12512)

69 ((analys#s or valuation? or value? or valuing) adj3 (conjoint or contingent)).tw,kf. (3208)

70 (autonom* adj3 (personal* or self)).tw,kf. (4900)

71 (choice? adj1 (discrete or experiment*)).tw,kf. (6157)

72 ((client? or consumer? or patient?) adj (centered or centred or focus*)).tw,kf. (52284)

73 ((client? or consumer? or patient? or personal) adj narrati*).tw,kf. (2210)

74 empower*.tw,kf. (49166)

75 (focus group? or interview* or questionnaire? or survey*).tw,kf. (2689332)

76 (freedom? or libert*).tw,kf. (100795)

77 gambl*.tw,kf. (19958)

78 ((health or death) adj3 (anxiet* or anxious* or attitud* or concern* or fear* or feel? or feeling* or felt or perception* or perspective? or prefer* or view* or worrie? or worry*)).tw,kf. (161154)

79 health utilit*.tw,kf. (4626)

80 informed choice?.tw,kf. (5052)

81 (life adj3 (event? or experience?)).tw,kf. (63360)

82 (multi?attribute or multi?criteria).tw,kf. (2095)

83 (preference? adj1 (elicit* or scor* or stated)).tw,kf. (3368)

84 prospect theor*.tw,kf. (513)

85 (self adj2 (conceiv* or concept* or percepti* or perceiv*)).tw,kf. (45854)

86 (self adj (determin* or efficac* or help or manag* or support*)).tw,kf. (110203)

87 (social* adj1 valu*).tw,kf. (4014)

88 trade?off?.tw,kf. (11989)

89 (willing* adj2 pay*).tw,kf. (13209)

90 or/35-89 [COMBINED MeSH & TEXT WORDS FOR PATIENT PREFERENCES & VALUES] (6578882)

91 exp Communication/ (870722)

92 ((time$2 or timeliness) adj2 (communica* or info*)).tw,kf. (16287)

93 (miscommunicat* or mis-communicat*).tw,kf. (1807)

94 (misunderstand* or mis-understand*).tw,kf. (11841)

95 (misinform* or mis-inform*).tw,kf. (5330)

96 ((involv* or participat*) adj3 (client? or consumer? or patient?)).tw,kf. (212048)

97 exp Informed Consent/ (135722)

98 (informed adj (choice* or choos* or consent* or decision*)).tw,kf. (120770)

99 (choice? adj2 behavio?r*).tw,kf. (5292)

100 ((client? or consumer? or patient? or personal) adj3 consent*).tw,kf. (38447)

101 ((make or making or makes or made or shar* or support*) adj2 (choice? or choos* or decision*)).tw,kf. (364434)

102 Patient Reported Outcome Measures/ (11401)

103 patient reported outcome?.tw,kf. (34484)

104 (PROM or PROMs or ePREM or ePREMs).tw,kf. (8161)

105 or/91-104 [PATIENT COMMUNICATION / MISCOMMUNICATION / CONSENT / SUPPORT] (1646152)

106 90 or 105 (7475595)

107 34 or 106 [ALL PATIENT PREFERENCES & VALUES SETS] (7476065)

108 23 and 107 [GERD/ESOPHAGEAL CANCER - PATIENT PREFERENCES & VALUES] (1577)

109 108 use medall [MEDLINE RECORDS] (636)

110 gastroesophageal reflux/ (76189)

111 ((esophageal or gastric* or gastro-esophageal or gastro-oesophageal or gastroesophageal or gastrooesophageal or supraesophageal or supra-esophageal or supraoesophageal or supra-oesophageal) adj2 reflux*).tw,kw. (60380)

112 GERD.tw,kw. (23254)

113 GORD.tw,kw. (2189)

114 SEGR.tw,kw. (14)

115 (gastric adj2 regurgitat*).tw,kw. (524)

116 or/110-115 (94213)

117 exp esophagus tumor/ (77710)

118 exp esophagus/ and exp neoplasm/ (37262)

119 ((esophag* or oesophag* or pharynx-esophag*) adj3 (neoplas* or cancer* or tumour* or tumor* or carcinoma* or malignan* or metasta* or oncolog* or adenoma* or adenocarcinoma* or adeno-carcinoma* or carcinosarcoma* or carcino-sarcoma*)).tw,kw. (115398)

120 Barrett Esophagus/ (23026)

121 (Barrett* adj1 (esophag* or oesophag* or epitheli* or metaplasi* or syndrome?)).tw,kw. (22711)

122 (dysplasia* or dysplastic* or precancer* or pre-cancer* or premalignan* or pre-malignan*).tw,kw. (237615)

123 or/117-122 (388236)

124 116 and 123 [GERD AND ESOPHAGEAL CANCER] (13204)

125 exp juvenile/ not (exp juvenile/ and exp adult/) (2337309)

126 exp Infant/ not (exp Adult/ and exp Infant/) (1653036)

127 exp Child/ not (exp Adult/ and exp Child/) (3188154)

128 or/125-127 (3908908)

129 124 not 128 [CHILD, 17 AND UNDER, REMOVED] (12752)

130 exp animal experimentation/ or exp models animal/ or exp animal experiment/ or nonhuman/ or exp vertebrate/ (47785712)

131 exp human/ or exp human experimentation/ or exp human experiment/ (37610583)

132 130 not 131 (10176834)

133 129 not 132 [ANIMAL-ONLY REMOVED] (12412)

134 editorial.pt. (1053946)

135 letter.pt. not (letter.pt. and randomized controlled trial/) (2034134)

136 133 not (134 or 135) [OPINION PIECES REMOVED] (11719)

137 adaptive behavior/ (142109)

138 attitude/ (105292)

139 attitude to death/ (25924)

140 attitude to disability/ (316)

141 attitude to health/ (184789)

142 attitude to illness/ (4863)

143 attitude to life/ (642)

144 consumer advocacy/ (6368)

145 consumer attitude/ (3823)

146 cooperation/ (40714)

147 decision making/ (290020)

148 health care survey/ (44378)

149 exp interview/ (267434)

150 life event/ (27394)

151 patient advocacy/ (44216)

152 exp patient attitude/ (358813)

153 patient decision making/ (8997)

154 exp patient education/ (186333)

155 personal autonomy/ (28669)

156 psychological aspect/ (478485)

157 exp questionnaire/ (1538806)

158 exp self concept/ (289574)

159 self help/ (13262)

160 exp social psychology/ (924332)

161 ((accept* or anxiet* or anxious* or attitud* or consider* or choice? or choos* or chose? or concern* or decid* or decis* or dissatisf* or expect* or experienc* or fear* or feel* or felt or input* or opinion* or participat* or perceiv* or percepti* or perspective? or prefer* or respons* or satisf* or unsatisf* or value? or valuing or view* or worrie? or worry*) adj3 (citizen? or client? or consumer? or female? or male? or men or patient? or public or stake?holder* or user? or wom#n)).tw,kf. (1783010)

162 (advoca* adj3 (client? or consumer? or patient?)).tw,kf. (12512)

163 ((analys#s or valuation? or value? or valuing) adj3 (conjoint or contingent)).tw,kf. (3208)

164 (autonom* adj3 (personal* or self)).tw,kf. (4900)

165 (choice? adj1 (discrete or experiment*)).tw,kf. (6157)

166 ((client? or consumer? or patient?) adj (centered or centred or focus*)).tw,kf. (52284)

167 ((client? or consumer? or patient? or personal) adj narrati*).tw,kf. (2210)

168 empower*.tw,kf. (49166)

169 (focus group? or interview* or questionnaire? or survey*).tw,kf. (2689332)

170 (freedom? or libert*).tw,kf. (100795)

171 gambl*.tw,kf. (19958)

172 ((health or death) adj3 (anxiet* or anxious* or attitud* or concern* or fear* or feel? or feeling* or felt or perception* or perspective? or prefer* or view* or worrie? or worry*)).tw,kf. (161154)

173 health utilit*.tw,kf. (4626)

174 informed choice?.tw,kf. (5052)

175 (life adj3 (event? or experience?)).tw,kf. (63360)

176 (multi?attribute or multi?criteria).tw,kf. (2095)

177 (preference? adj1 (elicit* or scor* or stated)).tw,kf. (3368)

178 prospect theor*.tw,kf. (513)

179 (self adj2 (conceiv* or concept* or percepti* or perceiv*)).tw,kf. (45854)

180 (self adj (determin* or efficac* or help or manag* or support*)).tw,kf. (110203)

181 (social* adj1 valu*).tw,kf. (4014)

182 trade?off?.tw,kf. (11989)

183 (willing* adj2 pay*).tw,kf. (13209)

184 or/137-183 [Combined MeSH & text words for patient preferences & values] (6727797)

185 communication/ (197407)

186 exp verbal communication/ (291543)

187 ((time$2 or timeliness) adj2 (communica* or info*)).tw,kf. (16287)

188 (miscommunicat* or mis-communicat*).tw,kf. (1807)

189 (misunderstand* or mis-understand*).tw,kf. (11841)

190 (misinform* or mis-inform*).tw,kf. (5330)

191 ((involv* or participat*) adj3 (client? or consumer? or patient?)).tw,kf. (212048)

192 informed consent/ (131281)

193 (informed adj (choice* or choos* or consent* or decision*)).tw,kf. (120770)

194 (choice? adj2 behavio?r*).tw,kf. (5292)

195 ((client? or consumer? or patient? or personal) adj3 consent*).tw,kf. (38447)

196 ((make or making or makes or made or shar* or support*) adj2 (choice? or choos* or decision*)).tw,kf. (364434)

197 patient reported outcome?.tw,kf. (34484)

198 (PROM or PROMs or ePREM or ePREMs).tw,kf. (8161)

199 or/185-198 [Additional patient preference terms] (1271333)

200 184 or 199 [ALL PATIENT PREFERENCES & VALUES SETS] (7412212)

201 136 and 200 [GERD/ESOPHAGEAL CANCER - PATIENT PREFERENCES & VALUES] (2198)

202 201 use emczd [EMBASE RECORDS] (1377)

203 109 or 202 [BOTH DATABASES] (2013)

204 (2017 04* or 2017 05* or 2017 06* or 2017 07* or 2017 08* or 2017 09* or 2017 10* or 2017 11* or 2017 12* or 2018*).dt. (1988203)

205 109 and 204 [MEDLINE UPDATE RECORDS] (34)

206 (201704* or 201705* or 201706* or 201707* or 201708* or 201709* or 201710* or 201711* or 201712* or 2018*).dc. (2921967)

207 202 and 206 [EMBASE UPDATE RECORDS] (150)

208 205 or 207 [BOTH DATABASES - UPDATE PERIOD] (184)

209 remove duplicates from 208 (158)

210 209 use medall [MEDLINE UNIQUE UPDATE RECORDS] (33)

211 209 use emczd [EMBASE UNIQUE UPDATE RECORDS] (125)

***************************

Cochrane

<https://www.cochranelibrary.com/advanced-search/search-manager?search=2220026>

Date Run: 30/10/2018 02:50:02

ID Search Hits

#1 MeSH descriptor: ["Gastroesophageal Reflux"] explode all trees 1740

#2 ((esophageal or gastric* or (gastro next esophageal) or (gastro next oesophageal) or supraesophageal or (supra next esophageal) or supraoesophageal or (supra next oesophageal)) near/2 reflux*):ti,ab,kw 1064

#3 GERD:ti,ab,kw 1185

#4 GORD:ti,ab,kw 148

#5 SEGR:ti,ab,kw 1

#6 (gastric near/2 regurgitat*):ti,ab,kw 67

#7 {or #1-#6} 2862

#8 MeSH descriptor: ["Esophageal Neoplasms"] explode all trees 1308

#9 MeSH descriptor: [Esophagus] explode all trees 267

#10 ((esophag* or oesophag* or (pharynx next esophag*)) near/3 (neoplas* or cancer* or tumour* or tumor* or carcinoma* or malignan* or metasta* or oncolog* or adenoma* or adenocarcinoma* or (adeno next carcinoma*) or carcinosarcoma* or (carcino next sarcoma*))):ti,ab,kw 3387

#11 MeSH descriptor: ["Barrett Esophagus"] explode all trees 207

#12 (Barrett* near/1 (esophag* or oesophag* or epitheli* or metaplasi* or syndrome*)):ti,ab,kw 471

#13 (dysplasia* or dysplastic* or precancer* or (pre next cancer*) or premalignan* or (pre next malignan*)):ti,ab,kw 3694

#14 {or #8-#13} 7045

#15 #7 and #14 133

#16 MeSH descriptor: [Infant] explode all trees 14928

#17 MeSH descriptor: [Child] explode all trees 1356

#18 MeSH descriptor: [Juvenile] explode all trees 0

#19 #15 not (#16 or #17 or #18) 131

#20 MeSH descriptor: ["Gastroesophageal Reflux"] explode all trees and with qualifier(s): [PX - PX] 37

#21 MeSH descriptor: ["Esophageal Neoplasms"] explode all trees and with qualifier(s): [PX - PX] 18

#22 MeSH descriptor: ["Barrett Esophagus"] explode all trees and with qualifier(s): [PX - PX] 1

#23 MeSH descriptor: ["Mass Screening"] explode all trees and with qualifier(s): [PX - PX] 178

#24 MeSH descriptor: ["Early Detection of Cancer"] explode all trees and with qualifier(s): [PX - PX] 95

#25 MeSH descriptor: ["Diagnostic Tests, Routine"] explode all trees and with qualifier(s): [PX - PX] 2

#26 MeSH descriptor: [Endoscopy] explode all trees and with qualifier(s): [PX - PX] 190

#27 MeSH descriptor: ["Endoscopy, Gastrointestinal"] explode all trees and with qualifier(s): [PX - PX] 97

#28 MeSH descriptor: [Esophagoscopy] explode all trees and with qualifier(s): [PX - PX] 0

#29 MeSH descriptor: [Gastroscopy] explode all trees and with qualifier(s): [PX - PX] 10

#30 {or #20-#29} 478

#31 MeSH descriptor: ["Adaptation, Psychological"] explode all trees 4942

#32 MeSH descriptor: [Attitude] explode all trees 1009

#33 MeSH descriptor: ["Attitude to Death"] explode all trees 148

#34 MeSH descriptor: ["Attitude to Health"] explode all trees 32342

#35 MeSH descriptor: ["Choice Behavior"] explode all trees 1335

#36 MeSH descriptor: ["Consumer Advocacy"] explode all trees 14

#37 MeSH descriptor: ["Consumer Behavior"] explode all trees 56

#38 MeSH descriptor: ["Consumer Participation"] explode all trees 1402

#39 MeSH descriptor: ["Cooperative Behavior"] explode all trees 929

#40 MeSH descriptor: ["Decision Making"] explode all trees 2001

#41 MeSH descriptor: ["Focus Groups"] explode all trees 522

#42 MeSH descriptor: ["Health Care Surveys"] explode all trees 547

#43 MeSH descriptor: ["Health Services Accessibility"] explode all trees 606

#44 MeSH descriptor: ["Interviews as Topic"] explode all trees 1733

#45 MeSH descriptor: ["Life Change Events"] explode all trees 449

#46 MeSH descriptor: [Narration] explode all trees 171

#47 MeSH descriptor: ["Patient Acceptance of Health Care"] explode all trees 2601

#48 MeSH descriptor: ["Patient Advocacy"] explode all trees 72

#49 MeSH descriptor: ["Patient-Centered Care"] explode all trees 557

#50 MeSH descriptor: ["Patient Education as Topic"] explode all trees 8048

#51 MeSH descriptor: ["Patient Participation"] explode all trees 1156

#52 MeSH descriptor: ["Patient Preference"] explode all trees 637

#53 MeSH descriptor: ["Patient Satisfaction"] explode all trees 11067

#54 MeSH descriptor: [Patients] explode all trees and with qualifier(s): [PX - PX] 540

#55 MeSH descriptor: ["Personal Autonomy"] explode all trees 196

#56 MeSH descriptor: ["Power (Psychology)"] explode all trees 21

#57 Any MeSH descriptor 49716

#58 MeSH descriptor: ["Quality of Life"] explode all trees and with qualifier(s): [PX - PX] 1852

#59 MeSH descriptor: ["Self Concept"] explode all trees 6343

#60 MeSH descriptor: ["Self Efficacy"] explode all trees 2661

#61 MeSH descriptor: ["Self-Help Groups"] explode all trees 725

#62 MeSH descriptor: ["Social Values"] explode all trees 154

#63 ((accept* or anxiet* or anxious* or attitud* or consider* or choice* or choos* or chose* or concern* or decid* or decis* or dissatisf* or expect* or experienc* or fear* or feel* or felt or input* or opinion* or participat* or perceiv* or percepti* or perspective* or prefer* or respons* or satisf* or unsatisf* or value* or valuing or view* or worrie* or worry*) near/3 (citizen* or client* or consumer* or female* or male* or men or patient* or public or stake*holder* or user* or woman or women)):ti,ab,kw 116467

#64 (advoca* near/3 (client* or consumer* or patient*)):ti,ab,kw 339

#65 ((analys* or valuation* or value* or valuing) near/3 (conjoint or contingent)):ti,ab,kw 90

#66 (autonom* near/3 (personal* or self)):ti,ab,kw 358

#67 (choice* near/1 (discrete or experiment*)):ti,ab,kw 165

#68 ((client* or consumer* or patient*) next (centered or centred or focus*)):ti,ab,kw 2741

#69 ((client* or consumer* or patient* or personal) next narrati*):ti,ab,kw 55

#70 empower*:ti,ab,kw 1960

#71 ("focus group" or "focus groups" or interview* or questionnaire* or survey*):ti,ab,kw 109696

#72 (freedom* or libert*):ti,ab,kw 3073

#73 gambl*:ti,ab,kw 672

#74 ((health or death) near/3 (anxiet* or anxious* or attitud* or concern* or fear* or feel or feels or feeling* or felt or perception* or perspective* or prefer* or view* or worrie* or worry*)):ti,ab,kw 14714

#75 (health next utilit*):ti,ab,kw 431

#76 (informed next choice*):ti,ab,kw 248

#77 (life near/3 (event* or experience*)):ti,ab,kw 2140

#78 (multi*attribute or multi*criteria):ti,ab,kw 87

#79 (preference* near/1 (elicit* or scor* or stated)):ti,ab,kw 165

#80 (prospect next theor*):ti,ab,kw 27

#81 (self near/2 (conceiv* or concept* or percepti* or perceiv*)):ti,ab,kw 6382

#82 (self next (determin* or efficac* or help or manag* or support*)):ti,ab,kw 14580

#83 (social* near/1 valu*):ti,ab,kw 195

#84 trade*off*:ti,ab,kw 636

#85 (willing* near/2 pay*):ti,ab,kw 960

#86 MeSH descriptor: [Communication] explode all trees 8033

#87 (time* near/2 (communica* or info*)):ti,ab,kw 516

#88 mis*communicat*:ti,ab,kw 41

#89 mis*understand*:ti,ab,kw 129

#90 mis*inform*:ti,ab,kw 76

#91 ((involv* or participat*) near/3 (client* or consumer* or patient*)):ti,ab,kw 16155

#92 MeSH descriptor: ["Informed Consent"] explode all trees 657

#93 (informed next (choice* or choos* or consent* or decision*)):ti,ab,kw 13770

#94 (choice* near/2 behavio*):ti,ab,kw 1334

#95 ((client* or consumer* or patient* or personal) near/3 consent*):ti,ab,kw 5869

#96 ((make or making or makes or made or shar* or support*) near/2 (choice* or choos* or decision*)):ti,ab,kw 12710

#97 MeSH descriptor: ["Patient Reported Outcome Measures"] explode all trees 210

#98 ("patient reported" next outcome*):ti,ab,kw 4219

#99 (PROM or PROMS or ePREM or ePREMs):ti,ab,kw 565

#100 {or #31-#99} 275595

#101 #30 or #100 275631

#102 #19 and #101 with Cochrane Library publication date Between Apr 2017 and Oct 2018 9

CENTRAL – 9

## KQ3 Searches

Database: Embase Classic+Embase <1947 to 2018 October 26>, Ovid MEDLINE(R) ALL <1946 to October 25, 2018>

Search Strategy:

--------------------------------------------------------------------------------

1 Barrett Esophagus/ (23026)

2 (Barrett* adj1 (esophag* or oesophag* or epitheli* or metaplasi* or syndrome?)).tw,kf. (22348)

3 1 or 2 (27331)

4 ((Barrett* or esophag* or oesophag* or pharynx-esophag* or gastro-esophag* or gastro-oesophag*) adj3 (dysplasia* or dysplastic* or precancer* or pre-cancer* or premalignan* or pre-malignan*)).tw,kf. (5923)

5 3 or 4 (28574)

6 Esophageal Neoplasms/ (54194)

7 exp Esophagus/ and exp Neoplasms/ (37262)

8 ((esophag* or oesophag* or pharynx-esophag*) adj3 (neoplas* or cancer* or tumour* or tumor* or carcinoma* or malignan* or metasta* or oncolog* or adenoma* or adenocarcinoma* or adeno-carcinoma* or carcinosarcoma* or carcino-sarcoma*)).tw,kf. (115152)

9 or/6-8 (144916)

10 5 or 9 (156979)

11 exp Infant/ not (exp Adult/ and exp Infant/) (1653036)

12 exp Child/ not (exp Adult/ and exp Child/) (3188154)

13 10 not (11 or 12) (155770)

14 exp Animals/ not (exp Animals/ and Humans/) (16928885)

15 13 not 14 (118125)

16 (comment or editorial or interview or news).pt. (1857143)

17 (letter not (letter and randomized controlled trial)).pt. (2039050)

18 15 not (16 or 17) (112822)

19 limit 18 to systematic reviews [Limit not valid in Embase; records were retained] (53850)

20 meta analysis.pt. (93528)

21 exp meta-analysis as topic/ (55716)

22 (meta-analy* or metanaly* or metaanaly* or met analy* or integrative research or integrative review* or integrative overview* or research integration or research overview* or collaborative review*).tw,kf. (322057)

23 (systematic review* or systematic overview* or evidence-based review* or evidence-based overview* or (evidence adj3 (review* or overview*)) or meta-review* or meta-overview* or meta-synthes* or "review of reviews" or technology assessment* or HTA or HTAs).tw,kf. (382261)

24 exp Technology assessment, biomedical/ (23572)

25 (cochrane or health technology assessment or evidence report).jw. (38344)

26 (network adj (MA or MAs)).tw,kf. (16)

27 (NMA or NMAs).tw,kf. (4256)

28 indirect* compar*.tw,kf. (4481)

29 (indirect treatment* adj1 compar*).tw,kf. (605)

30 (mixed treatment* adj1 compar*).tw,kf. (1228)

31 (multiple treatment* adj1 compar*).tw,kf. (312)

32 (multi-treatment* adj1 compar*).tw,kf. (4)

33 simultaneous* compar*.tw,kf. (2157)

34 mixed comparison?.tw,kf. (53)

35 or/20-34 (671928)

36 18 and 35 (2302)

37 19 or 36 (54111)

38 37 use medall [MEDLINE RECORDS] (2016)

39 Barrett esophagus/ (23026)

40 (Barrett* adj1 (esophag* or oesophag* or epitheli* or metaplasi* or syndrome?)).tw,kw. (22711)

41 39 or 40 (27498)

42 esophagus dysplasia/ (864)

43 exp esophagus/ and dysplasia/ (1609)

44 ((Barrett* or esophag* or oesophag* or pharynx-esophag* or gastro-esophag* or gastro-oesophag*) adj3 (dysplasia* or dysplastic* or precancer* or pre-cancer* or premalignan* or pre-malignan*)).tw,kw. (6007)

45 or/42-44 (7478)

46 41 or 45 (29142)

47 exp esophagus tumor/ (77710)

48 exp esophagus/ and exp neoplasm/ (37262)

49 ((esophag* or oesophag* or pharynx-esophag*) adj3 (neoplas* or cancer* or tumour* or tumor* or carcinoma* or malignan* or metasta* or oncolog* or adenoma* or adenocarcinoma* or adeno-carcinoma* or carcinosarcoma* or carcino-sarcoma*)).tw,kw. (115398)

50 or/47-49 (152799)

51 46 or 50 (164217)

52 exp juvenile/ not (exp juvenile/ and exp adult/) (2337309)

53 exp Infant/ not (exp Adult/ and exp Infant/) (1653036)

54 exp Child/ not (exp Adult/ and exp Child/) (3188154)

55 or/52-54 (3908908)

56 51 not 55 (162843)

57 exp animal experimentation/ or exp models animal/ or exp animal experiment/ or nonhuman/ or exp vertebrate/ (47785712)

58 exp human/ or exp human experimentation/ or exp human experiment/ (37610583)

59 57 not 58 (10176834)

60 56 not 59 (159227)

61 editorial.pt. (1053946)

62 letter.pt. not (letter.pt. and randomized controlled trial/) (2034134)

63 60 not (61 or 62) (152899)

64 meta-analysis/ (244188)

65 "systematic review"/ (181694)

66 "meta analysis (topic)"/ (38725)

67 (meta-analy* or metanaly* or metaanaly* or met analy* or integrative research or integrative review* or integrative overview* or research integration or research overview* or collaborative review*).tw,kw. (324815)

68 (systematic review* or systematic overview* or evidence-based review* or evidence-based overview* or (evidence adj3 (review* or overview*)) or meta-review* or meta-overview* or meta-synthes* or "review of reviews" or technology assessment* or HTA or HTAs).tw,kw. (385401)

69 biomedical technology assessment/ (22465)

70 (cochrane or health technology assessment or evidence report).jw. (38344)

71 (network adj (MA or MAs)).tw,kw. (16)

72 (NMA or NMAs).tw,kw. (4274)

73 indirect* compar*.tw,kw. (4543)

74 (indirect treatment* adj1 compar*).tw,kw. (607)

75 (mixed treatment* adj1 compar*).tw,kw. (1251)

76 (multiple treatment* adj1 compar*).tw,kw. (317)

77 (multi-treatment* adj1 compar*).tw,kw. (4)

78 simultaneous* compar*.tw,kw. (2157)

79 mixed comparison?.tw,kw. (54)

80 or/64-79 (731158)

81 63 and 80 (4759)

82 81 use emczd [EMBASE RECORDS] (3269)

83 38 or 82 [BOTH DATABASES] (5285)

84 2018*.dt. (1068337)

85 38 and 84 [MEDLINE UPDATE RECORDS] (190)

86 2018*.dc. (1460954)

87 82 and 86 [EMBASE UPDATE RECORDS] (361)

88 85 or 87 [BOTH DATABASES - UPDATE RECORDS] (551)

89 remove duplicates from 88 (428)

90 89 use medall [MEDLINE UNIQUE UPDATE RECORDS] (184)

91 89 use emczd [EMBASE UNIQUE UPDATE RECORDS] (244)

***************************

Cochrane

Date Run: 30/10/2018 03:06:00

ID Search Hits

#1 MeSH descriptor: ["Barrett Esophagus"] explode all trees 207

#2 (Barrett* next (esophag* or oesophag* or epitheli* or metaplasi* or syndrome*)):ti,ab,kw 470

#3 #1 or #2 470

#4 ((Barrett* or esophag* or oesophag* or (pharynx next esophag*) or (gastro next esophag*) or (gastro next oesophag*)) near/3 (dysplasia* or dysplastic* or precancer* or (pre next cancer*) or premalignan* or (pre next malignan*))):ti,ab,kw 195

#5 #3 or #4 532

#6 MeSH descriptor: ["Esophageal Neoplasms"] explode all trees 1308

#7 MeSH descriptor: [Esophagus] explode all trees 267

#8 ((esophag* or oesophag* or (pharynx next esophag*)) near/3 (neoplas* or cancer* or tumour* or tumor* or carcinoma* or malignan* or metasta* or oncolog* or adenoma* or adenocarcinoma* or (adeno next carcinoma*) or carcinosarcoma* or (carcino next sarcoma*))):ti,ab,kw 3387

#9 {or #6-#8} 3457

#10 #5 or #9 3673

#11 MeSH descriptor: [Infant] explode all trees 14928

#12 MeSH descriptor: [Child] explode all trees 832

#13 #10 not (#11 or #12) with Cochrane Library publication date Between Jan 2018 and Oct 2018, in Cochrane Reviews, Cochrane Protocols 1
