# Supplementary material for: Screening for esophageal adenocarcinoma and precancerous conditions (dysplasia and Barrett’s esophagus) in patients with chronic gastroesophageal reflux disease with or without other risk factors: two systematic reviews and one overview of reviews to inform a guideline of the Canadian Task Force on Preventive Health Care (CTFPHC)
Source: Syst Rev. 2020 Jan 29;9:20. doi: 10.1186/s13643-020-1275-2 (PMC6990541; doi:10.1186/s13643-020-1275-2)
Supplement: Supplementary file 5 — Additional file 5: PRESS checklists. [file 13643_2020_1275_MOESM5_ESM.docx]

# Additional file 5. PRESS checklists

## KQ1. PRESS checklist

***PRESS Guideline 2015— Search Submission & Peer Review Assessment***

Reference: McGowan J, Sampson M, Salzwedel DM, Cogo E, Foerster V, Lefebvre C. PRESS Peer Review of Electronic Search Strategies: 2015 guideline statement. *J Clin Epidemiol* 2016;75:40-6. Available: <http://www.jclinepi.com/article/S0895-4356(16)00058-5/pdf>.

**Search submission: This section to be filled in by the searcher**

Searcher: Becky Skidmore Email: [bskidmore@rogers.com](mailto:bskidmore@rogers.com)

Date submitted: 2 Sep 2016 Date requested by: 7 Sep 2016 PM

| 1. **Systematic Review Title** |  |
| --- | --- |

Screening for esophageal adenocarcinoma and precancerous conditions (dysplasia and Barrett’s esophagus) in patients with chronic gastroesophageal reflux disease with or without other risk factors: a series of systematic reviews

| 1. **This search strategy is …** |
| --- |

| x | My PRIMARY (core) database strategy — First time submitting a strategy for search question and database |
| --- | --- |
|  | My PRIMARY (core) strategy — Follow-up review NOT the first time submitting a strategy for search question and database. If this is a response to peer review, itemize the changes made to the review suggestions |
|  | SECONDARY search strategy— First time submitting a strategy for search question and database |
|  | SECONDARY search strategy — NOT the first time submitting a strategy for search question and database. If  this is a response to peer review, itemize the changes made to the review suggestions |

| 1. **Database** (e.g., MEDLINE, CINAHL) *[mandatory]* |
| --- |

MEDLINE

| 1. **Interface** (e.g., Ovid, EbscoHost…) *[mandatory]* |
| --- |

Ovid

| 1. **Research Question** (Describe the purpose of the search)  *[mandatory]* |
| --- |

**KQ1a. In adults with chronic GERD with or without other risk factorsⱡ, what is the effectiveness (benefits and harms) of screening for EAC and precancerous conditions (Barrett’s esophagus and low- and high-grade dysplasia) with esophagogastroduodenoscopy (EGD) plus biopsy (if necessary) with or without adjunct techniques compared with no screening or another test? What are the effects in relevant subgroup populations?**

ⱡRisk factors will be deemed so by included studies

**KQ1b. If there is evidence of effectiveness†, what is the optimal time to initiate and to end screening, and the optimal screening interval (includes single and multiple tests and ongoing ‘surveillance’)?**

†If there is evidence of at least moderate quality of evidence, according to GRADE

| 1. **PICO Format** Outline the PICOs for your question — i.e., Patient, Intervention, Comparison, Outcome, and Study Design — as applicable |
| --- |

| **P** | Adults (≥18 years old) with chronic GERD with or without other risk factorsⱡ for EAC |
| --- | --- |
| **I** | *These are interventions and comparators:*  KQ1a  - Esophagogastroduodenoscopy (EGD)*† vs. no screening  EGD† plus adjunct techniques‡ vs. no screening  EGD† vs. EGD† plus adjunct techniques  *Also known as panendoscopy and upper GI endoscopy  † with or without biopsy protocol  ‡For example, chromendoscopy and narrow-band imaging  Screening for EAC, dysplasia, or Barrett’s esophagus  EGD could be used once or as a series of tests/surveillance  KQ1b   - One interval of screening* vs. another interval of screening - Timepoint at which to initiate screening* vs. another timepoint - Timepoint at which to cease screening* vs. another timepoint   *reflecting modalities shown above |
| **C** |  |
| **O** | Critical for decision-making   1. Mortality - all-cause (1, 5 and 10 year) 2. Mortality - cancer-related (1, 5 and 10 year) 3. Time to death 4. Proportion of survival 5. Quality of life (validated scales only)   Important for decision-making   1. Incidence of EAC, Barrett’s esophagus, low- and high-grade dysplasia 2. Stage of cancer (early vs. late stage diagnosis) 3. Life threatening, severe, or medically significant consequences (such as requiring hospitalization or prolongation of hospitalization; disabling; limiting self-care or activities of daily living) 4. Psychological effects (i.e., anxiety and depression) 5. Overdiagnosis*   *defined as the diagnosis of disease which would never have become clinically apparent in a person's lifetime (ie, causing neither symptoms nor death) |
| **S** | Randomized controlled trials (RCTs), including cluster RCTs.  If no or few randomized controlled trials are available: Non-randomized controlled clinical trials, controlled before-after, interrupted times series, cohort studies, case-control studies, limiting to higher levels of evidence depending on the nature and volume of specific study designs. |

| 1. **Inclusion Criteria** (List criteria such as age groups, study designs, etc., to be included) *[optional]*   **This search strategy is …** |
| --- |

| 1. **Exclusion Criteria** (List criteria such as study designs, date limits, etc., to be excluded) **[optional]** |
| --- |

| 1. **Was a search filter applied? Yes** |
| --- |

**If YES, which one(s) (e.g., Cochrane RCT filter, PubMed Clinical Queries filter)? Provide the source if this is a published filter.** *[mandatory if YES to previous question* — *textbox]*

*Various – amended Cochrane HSSS plus filters largely derived from CADTH*

| 1. **Notes or comments you feel would be useful for the peer reviewer**  *[optional]* |
| --- |

| 1. **Please copy and paste your search strategy here, exactly as run, including the number of hits per line. [mandatory]** |
| --- |

Database: Epub Ahead of Print, In-Process & Other Non-Indexed Citations, Ovid MEDLINE(R) Daily and Ovid MEDLINE(R) <1946 to Present>

Search Strategy:

--------------------------------------------------------------------------------

1 exp Gastroesophageal Reflux/ (23695)

2 ((esophageal or gastric* or gastro-esophageal or gastro-oesophageal or supraesophageal or supra-esophageal or supraoesophageal or supra-oesophageal) adj2 reflux*).tw,kw. (7037)

3 GERD.tw,kw. (6733)

4 GORD.tw,kw. (732)

5 SEGR.tw,kw. (5)

6 (gastric adj2 regurgitat*).tw,kw. (177)

7 or/1-6 (27723) [GERD]

8 Esophageal Neoplasms/di, pc (5750)

9 Barrett Esophagus/di, pc (1577)

10 7 and (8 or 9) (814) [SCREENING/DIAGNOSIS FOR ESOPHAGEAL CANCER IN GERD]

11 Esophageal Neoplasms/ (43184)

12 exp Esophagus/ and exp Neoplasms/ (9622)

13 ((esophag* or oesophag* or pharynx-esophag*) adj3 (neoplas* or cancer* or tumour* or tumor* or carcinoma* or malignan* or metasta* or oncolog* or adenoma* or adenocarcinoma* or adeno-carcinoma* or carcinosarcoma* or carcino-sarcoma* or hematoma* or lymphoma* or melanoma* or mesenchymoma* or sarcoma*)).tw,kw. (42075)

14 Barrett Esophagus/ (6913)

15 (Barrett* adj1 (esophag* or oesophag* or epitheli* or metaplasi* or syndrome?)).tw,kw. (7883)

16 (dysplasia* or dysplastic* or precancer* or pre-cancer* or premalignan* or pre-malignan*).tw,kw. (88918)

17 or/11-16 (144178) [ESOPHAGEAL CANCER]

18 Mass Screening/ (89803)

19 screen*.tw,kw. (573683)

20 Early Detection of Cancer/ (14445)

21 ((early or earlier or earliest) adj5 (detect* or diagnos* or identif* or recogni*)).tw,kw. (224191)

22 Diagnostic Tests, Routine/ (8890)

23 (routine* adj3 (test or tests or testing or check* or diagnos* or evaluat* or exam*)).tw,kw. (44813)

24 Preventive Health Services/ (11978)

25 Endoscopy/ (45653)

26 Endoscopy, Gastrointestinal/ (15889)

27 ((gastrointestin* or gastro-intestin*) adj5 endoscop*).tw,kw. (10558)

28 (gastroendoscop* or gastro-endoscop*).tw,kw. (227)

29 ((esophag* or oesophag* or gastro-esophag* or gastro-oesophag*) adj5 endoscop*).tw,kw. (9925)

30 Esophagoscopy/ (13034)

31 (esophagoscop* or oesophagoscop*).tw,kw. (2420)

32 Gastroscopy/ (15572)

33 gastroscop*.tw,kw. (6150)

34 (esophagogastroduodenoscop* or esophago-gastroduodenoscop* or oesophagogastroduodenoscop* or oesophago-gastroduodenoscop*).tw,kw. (2996)

35 (esophagogastro-duodenoscop* or esophago-gastro-duodenoscop* or oesophagogastro-duodenoscop* or oesophago-gastro-duodenoscop*).tw,kw. (242)

36 panendoscop*.tw,kw. (562)

37 (upper adj2 endoscop*).tw,kw. (7886)

38 or/18-37 (949047) [SCREENING TECHNIQUES]

39 17 and 38 (23107) [SCREENING FOR ESOPHAGEAL CANCER]

40 7 and 39 (1447) [SCREENING FOR ESOPHAGEAL CANCER IN GERD]

41 10 or 40 (1760) [SCREENING FOR ESOPHAGEAL CANCER IN GERD]

42 exp Infant/ not (exp Adult/ and exp Infant/) (761245)

43 exp Child/ not (exp Adult/ and exp Child/) (1080517)

44 41 not (42 or 43) (1716) [INFANT/CHILD-ONLY REMOVED]

45 exp Animals/ not (exp Animals/ and Humans/) (4305602)

46 44 not 45 (1691) [ANIMAL-ONLY REMOVED]

47 (comment or editorial or interview or news).pt. (1164687)

48 (letter not (letter and randomized controlled trial)).pt. (934364)

49 46 not (47 or 48) (1601) [OPINION PIECES REMOVED]

50 limit 49 to systematic reviews (63)

51 meta analysis.pt. (72900)

52 exp meta-analysis as topic/ (15336)

53 (meta-analy* or metanaly* or metaanaly* or met analy* or integrative research or integrative review* or integrative overview* or research integration or research overview* or collaborative review*).tw. (104706)

54 (systematic review* or systematic overview* or evidence-based review* or evidence-based overview* or (evidence adj3 (review* or overview*)) or meta-review* or meta-overview* or meta-synthes* or "review of reviews" or technology assessment* or HTA or HTAs).tw. (126749)

55 exp Technology assessment, biomedical/ (9804)

56 (cochrane or health technology assessment or evidence report).jw. (18257)

57 or/51-56 (229930)

58 49 and 57 (44)

59 50 or 58 (74) [REVIEWS]

60 exp Guidelines as Topic/ (132232)

61 exp Clinical Protocols/ (142547)

62 Guideline.pt. (15949)

63 Practice Guideline.pt. (21793)

64 standards.fs. (610725)

65 Consensus Development Conference.pt. (10140)

66 (guidance* or guideline* or standards or recommendation*).ti. (120654)

67 (expert consensus or consensus statement* or consensus conference* or practice parameter* or position statement* or policy statement* or CPG or CPGs).tw. (41231)

68 or/60-67 (933356)

69 49 and 68 (71) [CPGS]

70 (controlled clinical trial or randomized controlled trial or pragmatic clinical trial).pt. (516375)

71 clinical trials as topic.sh. (179187)

72 (randomi#ed or randomly or RCT$1 or placebo*).tw. (763805)

73 ((singl* or doubl* or trebl* or tripl*) adj (mask* or blind* or dumm*)).tw. (147654)

74 trial.ti. (161195)

75 or/70-74 (1110816)

76 49 and 75 (96) [RCTS]

77 controlled clinical trial.pt. (91628)

78 Controlled Clinical Trial/ or Controlled Clinical Trials as Topic/ (96847)

79 (control* adj2 trial*).tw. (193969)

80 Non-Randomized Controlled Trials as Topic/ (79)

81 (nonrandom* or non-random* or quasi-random* or quasi-experiment*).tw. (42913)

82 (nRCT or nRCTs or non-RCT$1).tw. (505)

83 Controlled Before-After Studies/ (182)

84 (control* adj3 ("before and after" or "before after")).tw. (3354)

85 Interrupted Time Series Analysis/ (213)

86 (time series adj3 interrupt*).tw. (1667)

87 (pre- adj3 post-).tw. (56788)

88 (pretest adj3 posttest).tw. (3870)

89 Historically Controlled Study/ (70)

90 (control* adj2 stud$3).tw. (187540)

91 Control Groups/ (1605)

92 (control$ adj2 group$1).tw. (390804)

93 trial.ti. (161195)

94 or/77-93 (953789)

95 49 and 94 (131) [NON-RCTS]

96 exp Cohort Studies/ (1586304)

97 cohort$1.tw. (386199)

98 Retrospective Studies/ (601864)

99 (longitudinal or prospective or retrospective).tw. (930590)

100 ((followup or follow-up) adj (study or studies)).tw. (43510)

101 Observational study.pt. (25625)

102 (observation$2 adj (study or studies)).tw. (66930)

103 ((population or population-based) adj (study or studies or analys#s)).tw. (13629)

104 ((multidimensional or multi-dimensional) adj (study or studies)).tw. (90)

105 Comparative Study.pt. (1765718)

106 ((comparative or comparison) adj (study or studies)).tw. (92030)

107 exp Case-Control Studies/ (810268)

108 ((case-control* or case-based or case-comparison) adj (study or studies)).tw. (83624)

109 Cross-Sectional Studies/ (225758)

110 ((cross-sectional or frequency or prevalence) adj (analys#s or study or studies or survey$1)).tw. (146913)

111 or/96-110 (3932126)

112 49 and 111 (652) [OBSERVATIONAL STUDIES]

113 59 or 69 or 76 or 95 or 112 (781) [ALL STUDY DESIGNS]

***************************

**Peer review assessment: this section to be filled in by the reviewer**

|  | Reviewer: Kaitryn Campbell | Email: kaitryn_chris@sympatico.ca | Date completed: 5 Sept. 2016 |
| --- | --- | --- | --- |
|  |  |  |  |

Do you wish to be acknowledged? (If yes, the review team will be advised to add an acknowledgement to any publications related to this work.) Yes No

The suggested acknowledgement is “We thank Xxxxx Yyyyyy, MLIS, AHIP (xxxxx Health Sciences Library, University of xxxxxx) for peer review of the MEDLINE search strategy.” [please edit to indicate your name, postnomials and institutional affiliation as you would like them presented].

|  | **1. TRANSLATION** |  |  | | |
| --- | --- | --- | --- | --- | --- |
| A -­‐No revisions | | X |  |  |  |
| B -­‐ Revision(s) suggested | |  |  |  |  |
| C -­‐ Revision(s) required | |  |  |  |  |

If “B” or “C,” please provide an explanation or example:

**2. BOOLEAN AND PROXIMITY OPERATORS**

| A -­‐No revisions | X |
| --- | --- |
| B -­‐ Revision(s) suggested |  |
| C -­‐ Revision(s) required |  |

If “B” or “C,” please provide an explanation or example:

**3. SUBJECT HEADINGS**

| A -­‐No revisions |  |
| --- | --- |
| B -­‐ Revision(s) suggested | X |
| C -­‐ Revision(s) required |  |

If “B” or “C,” please provide an explanation or example:

For “esophagogastroduodenoscopy” concept, suggest adding: Endoscopy, Digestive System/

**4. TEXT WORD SEARCHING**

| A -­‐No revisions |  |
| --- | --- |
| B -­‐ Revision(s)suggested | X |
| C -­‐ Revision(s) required |  |

If “B” or “C,” please provide an explanation or example:

Lines 2/29, suggest adding non-hyphenated versions of following: gastroesophageal or gastrooesophageal

Line 19, very low probability will yield any additional relevant hits but consider adding: ((mass or population-based or widespread) adj (test or tests or testing or tested)).tw,kw.

**5. SPELLING, SYNTAX, AND LINE NUMBERS**

| A -­‐No revisions | X |
| --- | --- |
| B -­‐ Revision(s)suggested |  |
| C -­‐ Revision(s) required |  |

If “B” or “C,” please provide an explanation or example:

**6. LIMITS AND FILTERS**

| A -­‐No revisions | X |
| --- | --- |
| B -­‐ Revision(s) suggested |  |
| C -­‐ Revision(s) required |  |

If “B” or “C,” please provide an explanation or example:

OVERALL EVALUATION (Note: If one or more “revision required” is noted above, the response below must be “revisions required”.)

| A -­‐No revisions |  |
| --- | --- |
| B -­‐ Revision(s) suggested | X |
| C -­‐ Revision(s) required |  |

Additional comments:

##

## KQ2. PRESS checklist

***PRESS Guideline* 2015— Search Submission & Peer Review Assessment**

Reference: McGowan J, Sampson M, Salzwedel DM, Cogo E, Foerster V, Lefebvre C. PRESS Peer Review of Electronic Search Strategies: 2015 guideline statement. *J Clin Epidemiol* 2016;75:40-6. Available: <http://www.jclinepi.com/article/S0895-4356(16)00058-5/pdf>.

**Search submission: This section to be filled in by the searcher**

Searcher: Becky Skidmore Email: [bskidmore@rogers.com](mailto:bskidmore@rogers.com)

Date submitted: 2 Sep 2016 Date requested by: 7 Sep 2016 PM

| 1. **Systematic Review Title** |  |
| --- | --- |

Screening for esophageal adenocarcinoma and precancerous conditions (dysplasia and Barrett’s esophagus) in patients with chronic gastroesophageal reflux disease with or without other risk factors: a series of systematic reviews

| 1. **This search strategy is …** |
| --- |

| x | My PRIMARY (core) database strategy — First time submitting a strategy for search question and database |
| --- | --- |
|  | My PRIMARY (core) strategy — Follow-up review NOT the first time submitting a strategy for search question and database. If this is a response to peer review, itemize the changes made to the review suggestions |
|  | SECONDARY search strategy— First time submitting a strategy for search question and database |
|  | SECONDARY search strategy — NOT the first time submitting a strategy for search question and database. If  this is a response to peer review, itemize the changes made to the review suggestions |

| 1. **Database** (e.g., MEDLINE, CINAHL) *[mandatory]* |
| --- |

MEDLINE

| 1. **Interface** (e.g., Ovid, EbscoHost…) *[mandatory]* |
| --- |

Ovid

| 1. **Research Question** (Describe the purpose of the search)  *[mandatory]* |
| --- |

KQ2. In adults with chronic GERD with or without other risk factors who have been offered, received, or allocated to receive screening, how do they weigh the benefits and harms of endoscopic screening, and what factors contribute to these preferences and to their decisions to undergo screening?

| 1. **PICO Format** Outline the PICOs for your question — i.e., Patient, Intervention, Comparison, Outcome, and Study Design — as applicable |
| --- |

| **P** | Adults (≥18 years old) with chronic GERD with or without other risk factorsⱡ for EAC who have been offered, received, or allocated to receive screening, depending on the design of the study |
| --- | --- |
| **I** | Screening for EAC and other precancerous lesions with esophagogastroduodenoscopy (EGD)†  †Patients may have also received biopsy and/or other adjunct techniques |
| **C** | Depending on study design, comparators may be:   - No screening* - Other screening tests - Different screening intervals - Different lengths/duration of screening - Offered screening but did not receive screening - No comparison |
| **O** | - Willingness to be screened - Uptake of screening - Factors considered in decision to be screened (i.e., what components of screening do patients place more value on when deciding whether to be screened or not [e.g., potential complications resulting from screening], intrusiveness of the screening technology) |
| **S** | Randomized controlled trials  If insufficient data exists:  Controlled clinical trials, controlled before-after, case-controls, cohort, interrupted time series (ITS), and cross-sectional (i.e., surveys)  If insufficient data exists for the above:  Qualitative studies and mixed-methods studies |

| 1. **Inclusion Criteria** (List criteria such as age groups, study designs, etc., to be included) *[optional]*   **This search strategy is …** |
| --- |

| 1. **Exclusion Criteria** (List criteria such as study designs, date limits, etc., to be excluded) **[optional]** |
| --- |

| 1. **Was a search filter applied? Yes** No   This i |
| --- |

**If YES, which one(s) (e.g., Cochrane RCT filter, PubMed Clinical Queries filter)? Provide the source if this is a published filter.** *[mandatory if YES to previous question* — *textbox]*

This is based on various filters but greatly expanded – see <https://sites.google.com/a/york.ac.uk/issg-search-filters-resource/filters-to-identify-studies-of-public-views-and-patient-issues>

| 1. **Notes or comments you feel would be useful for the peer reviewer**  *[optional]* |
| --- |

I removed “interviews” as an opinion concept due to the nature of this topic

| 1. **Please copy and paste your search strategy here, exactly as run, including the number of hits per line. [mandatory]** |
| --- |

Database: Epub Ahead of Print, In-Process & Other Non-Indexed Citations, Ovid MEDLINE(R) Daily and Ovid MEDLINE(R) <1946 to Present>

Search Strategy:

--------------------------------------------------------------------------------

1 exp Gastroesophageal Reflux/ (23695)

2 ((esophageal or gastric* or gastro-esophageal or gastro-oesophageal or supraesophageal or supra-esophageal or supraoesophageal or supra-oesophageal) adj2 reflux*).tw,kw. (7037)

3 GERD.tw,kw. (6733)

4 GORD.tw,kw. (732)

5 SEGR.tw,kw. (5)

6 (gastric adj2 regurgitat*).tw,kw. (177)

7 or/1-6 [GERD] (27723)

8 Esophageal Neoplasms/di, pc (5750)

9 Barrett Esophagus/di, pc (1577)

10 7 and (8 or 9) [SCREENING & DIAGNOSIS OF ESOPHAGEAL CANCER IN GERD] (814)

11 Esophageal Neoplasms/ (43184)

12 exp Esophagus/ and exp Neoplasms/ (9622)

13 ((esophag* or oesophag* or pharynx-esophag*) adj3 (neoplas* or cancer* or tumour* or tumor* or carcinoma* or malignan* or metasta* or oncolog* or adenoma* or adenocarcinoma* or adeno-carcinoma* or carcinosarcoma* or carcino-sarcoma* or hematoma* or lymphoma* or melanoma* or mesenchymoma* or sarcoma*)).tw,kw. (42075)

14 Barrett Esophagus/ (6913)

15 (Barrett* adj1 (esophag* or oesophag* or epitheli* or metaplasi* or syndrome?)).tw,kw. (7883)

16 (dysplasia* or dysplastic* or precancer* or pre-cancer* or premalignan* or pre-malignan*).tw,kw. (88918)

17 or/11-16 [ESOPHAGEAL CANCER] (144178)

18 Mass Screening/ (89803)

19 screen*.tw,kw. (573683)

20 Early Detection of Cancer/ (14445)

21 ((early or earlier or earliest) adj5 (detect* or diagnos* or identif* or recogni*)).tw,kw. (224191)

22 Diagnostic Tests, Routine/ (8890)

23 (routine* adj3 (test or tests or testing or check* or diagnos* or evaluat* or exam*)).tw,kw. (44813)

24 Preventive Health Services/ (11978)

25 Endoscopy/ (45653)

26 Endoscopy, Gastrointestinal/ (15889)

27 ((gastrointestin* or gastro-intestin*) adj5 endoscop*).tw,kw. (10558)

28 (gastroendoscop* or gastro-endoscop*).tw,kw. (227)

29 ((esophag* or oesophag* or gastro-esophag* or gastro-oesophag*) adj5 endoscop*).tw,kw. (9925)

30 Esophagoscopy/ (13034)

31 (esophagoscop* or oesophagoscop*).tw,kw. (2420)

32 Gastroscopy/ (15572)

33 gastroscop*.tw,kw. (6150)

34 (esophagogastroduodenoscop* or esophago-gastroduodenoscop* or oesophagogastroduodenoscop* or oesophago-gastroduodenoscop*).tw,kw. (2996)

35 (esophagogastro-duodenoscop* or esophago-gastro-duodenoscop* or oesophagogastro-duodenoscop* or oesophago-gastro-duodenoscop*).tw,kw. (242)

36 panendoscop*.tw,kw. (562)

37 (upper adj2 endoscop*).tw,kw. (7886)

38 or/18-37 [SCREENING] (949047)

39 17 and 38 [SCREENING FOR ESOPHAGEAL CANCER] (23107)

40 7 and 39 [SCREENING FOR ESOPHAGEAL CANCER IN GERD] (1447)

41 10 or 40 [SCREENING FOR ESOPHAGEAL CANCER IN GERD] (1760)

42 exp Infant/ not (exp Adult/ and exp Infant/) (761245)

43 exp Child/ not (exp Adult/ and exp Child/) (1080517)

44 41 not (42 or 43) [CHILD-ONLY REMOVED] (1716)

45 exp Animals/ not (exp Animals/ and Humans/) (4305602)

46 44 not 45 [ANIMAL-ONLY REMOVED] (1691)

47 (comment or editorial or news).pt. (1137877)

48 (letter not (letter and randomized controlled trial)).pt. (934364)

49 46 not (47 or 48) [OPINION PIECES (EXCLUDING INTERVIEWS) - REMOVED] (1601)

50 exp Gastroesophageal Reflux/px (375)

51 Esophageal Neoplasms/px (188)

52 Barrett Esophagus/px (26)

53 Mass Screening/px (1883)

54 Early Detection of Cancer/px (681)

55 Diagnostic Tests, Routine/px (73)

56 Endoscopy/px (69)

57 Endoscopy, Gastrointestinal/px (74)

58 Esophagoscopy/px (15)

59 Gastroscopy/px (64)

60 or/50-59 [PSYCHOLOGICAL ASPECTS RE: DISEASE AND SCREENING TECHNIQUES] (3312)

61 exp Adaptation, Psychological/ (111443)

62 Attitude/ (42511)

63 Attitude to Death/ (14540)

64 exp Attitude to Health/ (341700)

65 Choice Behavior/ (26278)

66 Consumer Advocacy/ (3181)

67 *Consumer Behavior/ (8632)

68 exp Consumer Participation/ (35436)

69 Cooperative Behavior/ (36723)

70 Decision Making/ (77376)

71 Focus Groups/ (20944)

72 Health Care Surveys/ (27805)

73 Health Services Accessibility/ (59428)

74 Interviews as Topic/ (48873)

75 Life Change Events/ (20875)

76 Narration/ (6219)

77 Patient Acceptance of Health Care/ (35926)

78 Patient Advocacy/ (22917)

79 exp Patient-Centered Care/ (14110)

80 exp Patient Education as Topic/ (76354)

81 Patient Participation/ (20532)

82 Patient Preference/ (4757)

83 Patient Satisfaction/ (67010)

84 exp Patients/px (13741)

85 Personal Autonomy/ (14623)

86 *"Power (Psychology)"/ (4697)

87 Questionnaires/ (355771)

88 Quality of Life/px (17617)

89 exp Self Concept/ (92312)

90 Self Efficacy/ (14860)

91 exp Self-Help Groups/ (9369)

92 Social Values/ (18848)

93 ((accept* or anxiet* or anxious* or attitud* or consider* or choice? or choos* or chose? or concern* or decid* or decis* or dissatisf* or expect* or experienc* or fear* or feel* or felt or input* or opinion* or participat* or perceiv* or percepti* or perspective? or prefer* or respons* or satisf* or unsatisf* or value? or valuing or view* or worrie? or worry*) adj3 (citizen? or client? or consumer? or female? or male? or men or patient? or public or stake?holder* or user? or wom#n)).tw,kf. (621879)

94 (advoca* adj3 (client? or consumer? or patient?)).tw,kf. (4466)

95 ((analys#s or valuation? or value? or valuing) adj3 (conjoint or contingent)).tw,kf. (1192)

96 (autonom* adj3 (personal* or self)).tw,kf. (1879)

97 (choice? adj1 (discrete or experiment*)).tw,kf. (2111)

98 ((client? or consumer? or patient?) adj (centered or centred or focus*)).tw,kf. (17302)

99 ((client? or consumer? or patient? or personal) adj narrati*).tw,kf. (816)

100 empower*.tw,kf. (17247)

101 (focus group? or interview* or questionnaire? or survey*).tw,kf. (995333)

102 (freedom? or libert*).tw,kf. (38334)

103 gambl*.tw,kf. (7312)

104 ((health or death) adj3 (anxiet* or anxious* or attitud* or concern* or fear* or feel? or feeling* or felt or perception* or perspective? or prefer* or view* or worrie? or worry*)).tw,kf. (59315)

105 health utilit*.tw,kf. (1477)

106 informed choice?.tw,kf. (1983)

107 (life adj3 (event? or experience?)).tw,kf. (23474)

108 (multi?attribute or multi?criteria).tw,kf. (780)

109 (preference? adj1 (elicit* or scor* or stated)).tw,kf. (1219)

110 prospect theor*.tw,kf. (227)

111 (self adj2 (conceiv* or concept* or percepti* or perceiv*)).tw,kf. (18129)

112 (self adj (determin* or efficac* or help or manag* or support*)).tw,kf. (39935)

113 (social* adj1 valu*).tw,kf. (1587)

114 trade?off?.tw,kf. (4753)

115 (willing* adj2 pay*).tw,kf. (4088)

116 or/61-115 [COMBINED MeSH & TEXT WORDS FOR PATIENT PREFERENCES & VALUES] (2238824)

117 exp Communication/ (415814)

118 ((time$2 or timeliness) adj2 (communica* or info*)).tw,kf. (5778)

119 (miscommunicat* or mis-communicat*).tw,kf. (592)

120 (misunderstand* or mis-understand*).tw,kf. (4339)

121 (misinform* or mis-inform*).tw,kf. (2110)

122 ((involv* or participat*) adj3 (client? or consumer? or patient?)).tw,kf. (71512)

123 exp Informed Consent/ (37640)

124 (informed adj (choice* or choos* or consent* or decision*)).tw,kf. (36122)

125 (choice? adj2 behavio?r*).tw,kw. (2168)

126 ((client? or consumer? or patient? or personal) adj3 consent*).tw,kf. (10426)

127 ((make or making or makes or made or shar* or support*) adj2 (choice? or choos* or decision*)).tw,kw. (126716)

128 or/117-127 [PATIENT COMMUNICATION / MISCOMMUNICATION / CONSENT / SUPPORT] (665069)

129 116 or 128 (2667690)

130 60 or 129 (2668124)

131 49 and 130 [PATIENT PREFERENCES / COMMUNICATION - GERD / ESOPHAGEAL CANCER] (304)

***************************

**Peer review assessment: this section to be filled in by the reviewer**

|  | Reviewer: Kaitryn Campbell | Email: kaitryn_chris@sympatico.ca | Date completed: 5 Sept. 2016 |
| --- | --- | --- | --- |
|  |  |  |  |

Do you wish to be acknowledged? (If yes, the review team will be advised to add an acknowledgement to any publications related to this work.) Yes No

The suggested acknowledgement is “We thank Xxxxx Yyyyyy, MLIS, AHIP (xxxxx Health Sciences Library, University of xxxxxx) for peer review of the MEDLINE search strategy.” [please edit to indicate your name, postnomials and institutional affiliation as you would like them presented].

|  | **1. TRANSLATION** |  |  | | |
| --- | --- | --- | --- | --- | --- |
| A -­‐No revisions | | X |  |  |  |
| B -­‐ Revision(s) suggested | |  |  |  |  |
| C -­‐ Revision(s) required | |  |  |  |  |

If “B” or “C,” please provide an explanation or example:

**2. BOOLEAN AND PROXIMITY OPERATORS**

| A -­‐No revisions | X |
| --- | --- |
| B -­‐ Revision(s) suggested |  |
| C -­‐ Revision(s) required |  |

If “B” or “C,” please provide an explanation or example:

**3. SUBJECT HEADINGS**

| A -­‐No revisions |  |
| --- | --- |
| B -­‐ Revision(s) suggested | X |
| C -­‐ Revision(s) required |  |

If “B” or “C,” please provide an explanation or example:

Please see comment from KQ1 PRESS review.

**4. TEXT WORD SEARCHING**

| A -­‐No revisions |  |
| --- | --- |
| B -­‐ Revision(s)suggested | X |
| C -­‐ Revision(s) required |  |

If “B” or “C,” please provide an explanation or example:

Please see comments from KQ1 PRESS review.

**5. SPELLING, SYNTAX, AND LINE NUMBERS**

| A -­‐No revisions | X |
| --- | --- |
| B -­‐ Revision(s)suggested |  |
| C -­‐ Revision(s) required |  |

If “B” or “C,” please provide an explanation or example:

**6. LIMITS AND FILTERS**

| A -­‐No revisions | X |
| --- | --- |
| B -­‐ Revision(s) suggested |  |
| C -­‐ Revision(s) required |  |

If “B” or “C,” please provide an explanation or example:

OVERALL EVALUATION (Note: If one or more “revision required” is noted above, the response below must be “revisions required”.)

| A -­‐No revisions |  |
| --- | --- |
| B -­‐ Revision(s) suggested | X |
| C -­‐ Revision(s) required |  |

Additional comments:

Fascinating filter—results to be presumably supplemented from KQ1 included studies?

## KQ3. PRESS checlist

***PRESS Guideline* 2015— Search Submission & Peer Review Assessment^83^**

**Searcher’s Name:** Becky Skidmore **E-mail:** bskidmore@rogers.com

**Date submitted:** 7 Mar 2017 **Date needed by:** ASAP (Mar 9, if possible)

***Note to peer reviewers – please enter your information in the Peer Review Assessment area***

Remember: this peer review only pertains to your MEDLINE search strategy.

***Search question*** (Describe the purpose of the search)

*Title: Benefits and Harms of Treatment for Barrett’s Esophagus: An Overview of Systematic Reviews*

*Question: What is the evidence for the benefits and harms of treatment for Barrett’s esophagus (BE) on reducing EAC, EAC related and all-cause mortality, and improving quality of life?*

***PICO format*** (Outline the PICO for your question, i.e., the Patient, Intervention, Comparison and Outcome)

***P:*** *Adults ≥18 years with Barrett’s Esophagus (BE)*

***I:*** *Treatment strategies for BE including: pharmacological therapies, surveillance methods and endoscopic therapies*

***C:*** *One treatment method vs. another treatment method*

***O****: Effectiveness of treatment for BE. Primary/critical outcomes are: all-cause mortality and cancer-related mortality (1,5,10 year as available), survival, incidence of EAC, low- and high-grade dysplasia, stage at diagnosis, life-threatening or medically-significant consequences*

*(see also PICO table in Protocol for more details)*

***Inclusion criteria*** (List any inclusion criteria, such as age groups, study designs, to be included)

Systematic reviews

***Exclusion criteria*** (List any exclusion criteria, such as study designs, to be excluded)

***Was a search filter applied?*** (Remember this pertains only to the MEDLINE strategy)

| Yes |  |  | No |  |  |  |  |  |
| --- | --- | --- | --- | --- | --- | --- | --- | --- |

***If yes, which one?***

Cochrane hedge: PUBMED clinical query:

Haynes/McKibbon et al: SIGN (Scottish):

CRD (UK): Robinson and Dickerson:

Other: *Modified CADTH, have added in network meta-analyses*

***MEDLINE search interface used***

| *EBSCO* |  | OVID |  | *PubMED* |  | *Other* | ___________________ |
| --- | --- | --- | --- | --- | --- | --- | --- |

***Has the search strategy been adapted (i.e., subject heading and terms reviewed) for other databases? Please check all that apply.***

| Ageline | |  | |
| --- | --- | --- | --- |
| AMED | |  | |
| C2-SPCTRE | |  | |
| CINAHL | |  | |
| *Cochrane Database of Systematic Reviews* (CDSR; Cochrane Reviews) | |  | |
| Cochrane Central Register of Controlled Trials (CENTRAL; Clinical Trials) | |  | |
| Cochrane Methodology Register (CMR; Methods Studies) | |  | |
| Cochrane Library (all databases) | |  | |
| Database of Abstracts of Reviews of Effects (DARE; Other Reviews) | |  | |
| Embase | |  | |
| ERIC | |  | |
| LILACS (Latin American and Caribbean Health Sciences Literature) | |  | |
| MEDLINE | |  | |
| PsycINFO | |  | |
| PreMEDLINE | |  | |
| Cochrane HTA | |  | |
| Other | |  | |
| Other | |  | |

***Other notes or comments that you feel would be useful for the peer reviewer?***

Group has decided to revert back to the original search that included both BE and EAC search terms

***Please paste your MEDLINE strategy here:***

Database: Ovid MEDLINE(R) Epub Ahead of Print, In-Process & Other Non-Indexed Citations, Ovid MEDLINE(R) Daily and Ovid MEDLINE(R) <1946 to Present>

Search Strategy:

--------------------------------------------------------------------------------

1 Barrett Esophagus/ (7075)

2 (Barrett* adj1 (esophag* or oesophag* or epitheli* or metaplasi* or syndrome?)).tw,kw. (8135)

3 1 or 2 (9350)

4 ((Barrett* or esophag* or oesophag* or pharynx-esophag* or gastro-esophag* or gastro-oesophag*) adj3 (dysplasia* or dysplastic* or precancer* or pre-cancer* or premalignan* or pre-malignan*)).tw,kw. (2138)

5 3 or 4 (9835)

6 Esophageal Neoplasms/ (43936)

7 exp Esophagus/ and exp Neoplasms/ (9850)

8 ((esophag* or oesophag* or pharynx-esophag*) adj3 (neoplas* or cancer* or tumour* or tumor* or carcinoma* or malignan* or metasta* or oncolog* or adenoma* or adenocarcinoma* or adeno-carcinoma* or carcinosarcoma* or carcino-sarcoma*)).tw,kw. (42666)

9 or/6-8 (57539)

10 5 or 9 (61194)

11 exp Infant/ not (exp Adult/ and exp Infant/) (761289)

12 exp Child/ not (exp Adult/ and exp Child/) (1081083)

13 10 not (11 or 12) (60742)

14 exp Animals/ not (exp Animals/ and Humans/) (4327457)

15 13 not 14 (59461)

16 (comment or editorial or interview or news).pt. (1171650)

17 (letter not (letter and randomized controlled trial)).pt. (950923)

18 15 not (16 or 17) (56366)

19 limit 18 to systematic reviews (1429)

20 meta analysis.pt. (75191)

21 exp meta-analysis as topic/ (15514)

22 (meta-analy* or metanaly* or metaanaly* or met analy* or integrative research or integrative review* or integrative overview* or research integration or research overview* or collaborative review*).tw,kw. (112084)

23 (systematic review* or systematic overview* or evidence-based review* or evidence-based overview* or (evidence adj3 (review* or overview*)) or meta-review* or meta-overview* or meta-synthes* or "review of reviews" or technology assessment* or HTA or HTAs).tw,kw. (136923)

24 exp Technology assessment, biomedical/ (9954)

25 (cochrane or health technology assessment or evidence report).jw. (14812)

26 (network adj (MA or MAs)).tw,kw. (2)

27 (NMA or NMAs).tw,kw. (1409)

28 indirect comparison?.tw,kw. (1208)

29 (indirect treatment* adj1 comparison?).tw,kw. (119)

30 (mixed treatment* adj1 comparison?).tw,kw. (349)

31 (multiple treatment* adj1 comparison?).tw,kw. (74)

32 (multi-treatment* adj1 comparison?).tw,kw. (0)

33 simultaneous comparison?.tw,kw. (396)

34 mixed comparison?.tw,kw. (13)

35 or/20-34 (243608)

36 18 and 35 (1247)

37 19 or 36 (1644)

**Peer Review Assessment**

**[For peer reviewers only]**

**Peer reviewer’s name:** Kaitryn Campbell

**Press #:** N/A

**E-mail:** kaitryn_chris@sympatico.ca

**Date completed:** 7 Mar 2017

Please select the one most appropriate answer for each element

|  | **Adequate** | **Adequate with revisions*** | **Needs revision*** |
| --- | --- | --- | --- |
| 1. Translation of the research question | X |  |  |
| 2. Boolean and proximity operators | X |  |  |
| 3. Subject headings | X |  |  |
| 4. Natural language / free-text | X |  |  |
| 5. Spelling, syntax and line numbers | X |  |  |
| 6. Limits and filters | X |  |  |
| 7. Search strategy adaptations | X |  |  |

*** Provide an explanation or example for “Adequate with revisions” and “needs revision”:

Other Comments (please limit to 3-5 sentences): Well done and straight forward. No changes or suggestions.
