# Supplementary material for: Screening for esophageal adenocarcinoma and precancerous conditions (dysplasia and Barrett’s esophagus) in patients with chronic gastroesophageal reflux disease with or without other risk factors: two systematic reviews and one overview of reviews to inform a guideline of the Canadian Task Force on Preventive Health Care (CTFPHC)
Source: Syst Rev. 2020 Jan 29;9:20. doi: 10.1186/s13643-020-1275-2 (PMC6990541; doi:10.1186/s13643-020-1275-2)
Supplement: Supplementary file 6 — Additional file 6: Grey literature searching. [file 13643_2020_1275_MOESM6_ESM.docx]

# Additional file 6. Grey literature searching

To search for unpublished literature (e.g., reports, theses, governmental publications) we used the CADTH Grey Matters checklist. The CADTH checklist includes national and international health technology assessment agencies, clinical practice guideline organizations, drug and device regulatory agencies, health economics resources, clinical trials registries, Canadian health prevalence and incidence databases, statistics, search engines, and databases. The clinical trial registries listed within the checklist included the Canadian Cancer Trials, ClinicalTrials.gov, WHO International Clinical Trials Registry Platform, ISRCTN, CenterWatch, and Clinical Trials Registry India.

**Key Question 1.** We searched the following websites: Canadian Association of Gastroenterology, Cancer Care Ontario, Canadian Cancer Society, Canadian Digestive Health Foundation, Ontario Association of Gastroenterology, American Society for Gastrointestinal Endoscopy, American College of Gastroenterology, American Gastroenterological Association, British Society of Gastroenterology, American College of Physicians, American Cancer Society, US Preventive Services Task Force, Agency for Healthcare Research and Quality, and the Centers for Disease Control and Prevention. We also scanned the bibliographies of relevant systematic reviews and clinical practice guidelines identified from the search strategies and grey literature searching.

**Key Question 2**. We also searched the following websites: The Esophageal Cancer Awareness Association, the Canadian Cancer Society, the American Cancer Society, the American Association for Cancer Research, the Oesophageal Patients Association, the Esophageal Cancer Education Foundation, Canadian Association of Gastroenterology, and the Esophageal Cancer Action Network. Bibliographies of included studies were also scanned based on title. We also scanned the bibliographies of relevant systematic reviews and clinical practice guidelines identified from the search strategies and grey literature searching.

**Key Question 3**. Additional references were sought through hand-searching the bibliographies of SRs and clinical practice guidelines.
