# Supplementary material for: Screening for esophageal adenocarcinoma and precancerous conditions (dysplasia and Barrett’s esophagus) in patients with chronic gastroesophageal reflux disease with or without other risk factors: two systematic reviews and one overview of reviews to inform a guideline of the Canadian Task Force on Preventive Health Care (CTFPHC)
Source: Syst Rev. 2020 Jan 29;9:20. doi: 10.1186/s13643-020-1275-2 (PMC6990541; doi:10.1186/s13643-020-1275-2)
Supplement: Supplementary file 7 — Additional file 7: Screening forms. [file 13643_2020_1275_MOESM7_ESM.docx]

# Additional file 7. Screening forms

## KQ1 Screening forms

### Title and Abstract screening form

1. Does this article discuss screening adults (without other gastroesophageal condition [e.g. gastric cancer] or pre-existing disease [e.g. BE*, dysplasia or EAC]) for esophageal adenocarcinoma, Barrett's Esophagus, and/or dysplasia? (exclude case studies)
   * BE may also be referred to as intestinal metaplasia, specialized intestinal metaplasia, gastric metaplasia, columnar-lined esophagus

🔿 Yes/unclear

🔿 No

**Comment:**

### Full-text screening form

1. **Full text not available:**

❒ Yes

1. **Language:**

🔿 English/French

🔿 Other

1. **What is the study design?**

1. **Does this study evaluate a screening modality/technique of interest?**(EGD, EGD plus biospy with/without adjunct techniques, capsule endoscopy, transnasal/transoral ultrathin endoscopy, barium swallow/barium radiology, cytologic examination (e.g., brush, balloon, sponge, liquid, flow cytometry), endoscopic ultrasonography (EUS), computed tomography (CT) scan, laser-induced fluorescence spectroscopy)

🔿 Yes

🔿 No

🔿 Unclear

🔿 Molecular (e.g., cells, genes) and other biomarkers (e.g., blood, stool, urine)

1. **Does this study evaluate a comparator of interest?**

🔿 Yes (e.g., no screening, different test, different number of tests, different intervals)

🔿 No (not of interest)

🔿 No comparator (e.g., all participants received the same test/number/interval)

🔿 Unclear

1. **Do the participants have chronic GERD?**

(defined as symptoms for ≥12 months, with no specific frequency, and/or proton pump inhibitor (PPI) (or other pharmacotherpay) use for GERD for ≥12 months)

🔿 Yes (meets our def’n)

🔿 Yes (does not meet our def’n)

🔿 No

🔿 Unclear

1. **How does the study define chronic GERD?**Copy and paste from article. This will help us when we contact authors for those that are unclear. It will also help us know why we said yes/no/unclear while we do conflict resolution.
2. **Do participants have alarm symptoms of EAC or are diagnosed with other gastroesophageal conditions or pre-existing disease?**
   Alarm symptoms: dysphagia, recurrent vomiting, anorexia, weight loss, gastrointestinal bleeding or other symptoms identified by authors as 'alarm'
   Other gastroesophageal conditions: for example gastric cancer, other life threatening esophageal conditions)
   Pre-existing disease: Barrett's esophagus, dysplasia, or esophageal cancer

🔿 Yes

🔿 No

🔿 Unclear

🔿 Case-control (case have disease, controls do not)

1. **How old are the participants?**

🔿 Adults (18 yrs +)

🔿 Children (<18 yrs)

🔿 Adults and children (data separated)

🔿 Adults and children (data not separated)

🔿 Unclear

**Comments:**

## KQ2 Screening forms

### Title and Abstract screening form

1. Does this study discuss any of the following:

- patients choosing to/choosing not to undergo screening for EAC (or BE, dysplasia); OR
- how they weighted the benefits and harms of screening; OR
- what factors contributed to these preferences and to their decision to undergo/not undergo screening

🔿 Yes/unclear

🔿 No

### Full-text screening form

1. **Full text not available:**

❒ Yes

1. **Language:**

🔿 English/French

🔿 Other

1. **Is the study design a commentary, opinion, editorial or review?**

🔿 Yes

🔿 No

🔿 Abstract or protocol

1. **Are included participants adults (≥18 years old)?**

🔿 Yes

🔿 No

🔿 Unclear

1. **Have participants been diagnosed with other gastro-esophageal conditions (e.g., gastric cancer, esophageal atresia, other life threatening esophageal conditions) or pre-existing disease (BE, dyplasia, or EAC) or did they have alarm symptoms (e.g., vomiting, dysphagia)?**

🔿 Yes

🔿 No

🔿 Unclear (enter why it is unclear)

1. **Do participants have "chronic GERD"?**

defined as: (1) symptoms for ≥12 months, with no specific frequency; and/or (2) proton pump inhibitor (PPI) (or other pharmacotherapy) use for GERD for ≥12 months)

🔿 Yes

🔿 No

🔿 Unclear (enter GERD definition)

1. **Does this article include an intervention of interest (any screening modality for EAC and other precancerous lesions)**

🔿 Yes

🔿 No

🔿 Unclear (describe)

1. **Does this article have a comparator of interest?**

- no screening - among those offered
- different screening modality
- different screening intervals
- different lengths
- duration of screening
- offered screening but did not receive screening
- no comparison

🔿 Yes

🔿 No

🔿 Unclear

**Comments:**

## KQ3 Screening forms

### Title and abstract screening form

1. **Is this record a review (addresses multiple studies within)? (exclude primary studies such as RCTs, cohort, case-control, cross-sectional, case series, case reports, and editorials/ commentaries/ opinion pieces, and protocols)**

Notes: Include clinical practice guidelines and scoping reviews of interest at this level.

🔿 Yes (include)

🔿 Unclear (include)

🔿 No (exclude)

1. **Does the review describe a management/treatment regimen for EAC and/or BE and/or low- or high-grade dysplasia? (i.e., pharmacological, surveillance, surgical/mechanical or chemotherapy/radiation, surgery)?**

Notes: If review is not directly on management/treatment (e.g., prognostic factors), please exclude it (also exclude reviews that only consider dietary intakes, physical activities, smoking etc.).

If an otherwise eligible review does not specify the type of esophageal cancer (EAC or ESCC), please include it under "unclear" at this level. If it is only on ESCC, exclude it.

Include reviews that are on cancers of esophagogastric junction (that is located at the borderline between esophagus and stomach.

If a review only focuses on chemotherapy/immunotherapy and/or radiation therapy, please exclude it.

🔿 Yes (include)

🔿 Unclear (include)

🔿 No (exclude)

1. **Does the review discuss adults (≥18 years)?**

🔿 Yes (include)

🔿 Unclear (include)

🔿 No (exclude)

Additional notes (optional)

|  |
| --- |

### Full-text screening form

1. **Is this record a systematic review of RCTs (or provide a separate analysis for RCTs)?**

Note: In order to fulfill the SR definition, the record must meet all of the following criteria:1) searched at least one database; 2) reported selection criteria; 3) reported quality appraisal; 4) provided a list and synthesis of included studies.

Notes: If a review claims assessing risk of bias, but it does not report some details of QA, it would not satisfy the “QA” condition for question 1. As such it should be excluded (please see #2 below for explanation of details).

If a review does not use a specific tool for assessing the quality of included primary studies but just “generic” assessment of risk of bias, please include it as long as it reports some details of QA results. If they just state "low risk of bias" for overall body of evidence for example, you can't consider that sufficient as you have no idea how they determined that. If they provide at least final rating per study (e.g., low risk, high risk, scoring etc.) +/- additional details, please include it.

If the record is a clinical practice guideline, please exclude it under Q1; however, if it is based on an SR, please check if they have referenced the original SR. If yes, please look the SR up and assess if it meets our eligibility criteria. If yes, please send the note to Nadera with the citation/reference for the SR. If you are unable to locate the full-text of the referenced SR, please request Raymond to locate it for you and keep Nadera copied.

🔿 Yes (include)

🔿 No (exclude)

🔿 Can't tell because abstract only

🔿 Full-text not available in English

🔿 Full-text not available (other reasons)

1. **Does the SR discuss adults with EAC (stage 1 only), BE, low or high-grade dysplasia?**

Note: If an SR has mix of children and adults with no separate analyses for adults, please exclude it but make a note of it in the "additional note" section.

Cancer type: If an SR included mix of esophageal cancer types [esophageal adenocarcinoma (EAC) and esophageal squamous cell carcinoma (ESCC or SCC)], with no separate analysis for EAC, please exclude under question 2 “No (exclude)” option.

Cancer stage: If an SR included mix EAC stages e.g., 0, I, II, III with no separate analysis for stages 0 and/or I. Please exclude it under question 2 under “No (exclude)”” option.

If an SR does not report the type of esophageal cancer (EAC or SCC), or EAC’s stage (0, I, II, III) and there is no other clue to know if it was EAC with stage 0 and/or I, please exclude it by choosing “unclear” option under question 2 and write the reason in the box.

🔿 Yes (include)

🔿 No (exclude)

🔿 Unclear (please specify what is unclear)

1. **Does the review describe a management/treatment regimen for EAC (stage 1) and/or BE and/or low- or high-grade dysplasia? (i.e., pharmacological, surveillance, surgical/mechanical or surgery)?**

Note: If it addresses another type of treatment/management not listed above, please consult Nadera.

We are not interested in: perioperative protocols before/ after surgery etc., Reconstruction after esophagectomy e.g., gastric tube vs whole stomach etc., Palliation given it is not provided in stage 1

Please do include: different techniques of the same intervention e.g., different surgery techniques/procedures of the esophagectomy

🔿 Yes (include)

🔿 No (exclude)

🔿 Unclear (please specify what is unclear)

1. **Does the SR compare one management/treatment strategy to another management/ treatment strategy or to no management/ treatment?**

🔿 Yes (include)

🔿 No (exclude)

1. **Is there any other reason to exclude this SR?**

🔿 Yes exclude (please specify in the box)

🔿 No, include

**Additional notes (optional)**

|  |
| --- |

Optional question for "eligible SRs that includes RCTs but with no sufficient separate data for RCTs". In order to conduct a separate synthesis for RCTs, one would need to go to the primary studies.

|  |
| --- |
