# Supplementary material for: Screening for esophageal adenocarcinoma and precancerous conditions (dysplasia and Barrett’s esophagus) in patients with chronic gastroesophageal reflux disease with or without other risk factors: two systematic reviews and one overview of reviews to inform a guideline of the Canadian Task Force on Preventive Health Care (CTFPHC)
Source: Syst Rev. 2020 Jan 29;9:20. doi: 10.1186/s13643-020-1275-2 (PMC6990541; doi:10.1186/s13643-020-1275-2)
Supplement: Supplementary file 8 — Additional file 8: List of excluded studies. [file 13643_2020_1275_MOESM8_ESM.docx]

# Additional file 8. List of excluded studies

## KQ1 List of excluded studies at full text

### Full text not available (n=95)

1. Pace, F., Manes, G., Conio, M., and Bianchi, Porro G. Pretreatment edoscopy - Pro & contra: Endoscopy is needed before treatment in all patients with gastroesophageal reflux disease. Endoscopy 2006; 38 (3): 271-275.
2. Beck, I. T., Champion, M. C., Lemire, S., Thomson, A. B., Anvari, M., Armstrong, D., Bailey, R. J., Barkun, A. N., Boivin, M., Bursey, R. F., Chaun, H., Chiba, N., Cockeram, A. W., Connon, J. J., Da Costa, L. R., Faloon, T. R., Fedorak, R. N., Gillies, R. R., Goeree, R., Hunt, R. H., Inculet, R. I., Klein, A., Leddin, D. J., Love, J. R., and Worobetz, L. J. The Second Canadian Consensus Conference on the Management of Patients with Gastroesophageal Reflux Disease. Canadian journal of gastroenterology 1997; 11 Suppl B: 7B-20B.
3. Lam, C., Liu, W. F., Bel, R. D., Chan, K., Miller, L., Brown, M. C., Chen, Z., Cheng, D., Patel, D., Xu, W., Darling, G. E., and Liu, G. Polymorphisms of the FOXF1 and MHC locus genes in individuals undergoing esophageal acid reflux assessments. Diseases of the Esophagus 2017 Feb; 30(2):1-7.
4. Piche, T. Dietary intake and risk of gastroesophageal reflux disease: A cross study of volunteers. Hepato-Gastro 2005; 12 (3): 229-230.
5. Garrido, Serrano A., Guerrero Igea, F. J., Lepe Jimenez, J. A., and Perianes, Hernandez C. Clinical features and endoscopic progression of gastroesophageal reflux disease. Revista Espanola de Enfermedades Digestivas 2003; 95 (10): 712-716.
6. Dias, Moretzsohn L., Diniz De Miranda, C. H., Barbosa, A. J. A., and Gonzaga Vaz, Coelho L. The presence of serum anti-Cag A antibodies of Helicobacter pylori may not represent a protective factor in the severe esophageal forms of GERD. Gastrenterologia Endoscopia Digestiva 2003; 22 (5): 175-180.
7. Tutuian, R. and Castell, D. O. Barrett's esophagus prevalence and epidemiology. Gastrointestinal Endoscopy Clinics of North America 2003; 13 (2): 227-232.
8. Dincer, D., Besisik, F., Sahin, E., Demir, K., Tuncer, I., Cevikbas, U., Mungan, Z., Kaymakoglu, S., Boztas, G., Ozdil, S., Cakaloglu, Y., and Okten, A. Intestinal metaplasia of the gastric cardia: A study from Turkey. Hepato-gastroenterology 2002; 49 (46): 1153-1156.
9. Andrews, J. Hiatus hernia: What the GP needs to know. Medicine Today 2002; 3 (1): 39-43.
10. Csendes, A., Smok, G., Cerda, G., Burdiles, P., Mazza, D., and Csendes, P. Prevalence of Helicobacter pylori infection in 190 control subjects and in 236 patients with gastroesophageal reflux, erosive esophagitis or Barrett's esophagus. Diseases of the Esophagus 1997; 10 (1): 38-42.
11. D'Onofrio, V., Bovero, E., and Iaquinto, G. Characterization of acid and alkaline reflux in patients with Barrett's esophagus. Diseases of the Esophagus 1997; 10 (1): 16-23.
12. Lunedei, V., Bazzoli, F., Pozzato, P., De Luca, L., Zagari, R. M., Fossi, S., Ricciardiello, L., Maltoni, S., and Roda, E. Endoscopic surveillance in Barrett's esophagus. Minerva Gastroenterologica e Dietologica 2002; 48 (2): 63-71.
13. De Backer, A. I., De Schepper, A. M., and Pelckmans, P. The value of medical imaging in uncomplicated and complicated Barrett's esophagus. Acta Gastro-Enterologica Belgica 2000; 63 (1): 22-28.
14. Peng, S., Cui, Y., Xiao, Y. L., Xiong, L. S., Hu, P. J., Li, C. J., and Chen, M. H. Prevalence of erosive esophagitis and Barrett's esophagus in the adult Chinese population. Endoscopy 2009; 41 (12): 1011-1017.
15. Elhak, N. Gad, Mostafa, M., Salah, T., and Haleem, M. Duodenogastroesophageal reflux: results of medical treatment and antireflux surgery. Hepato-gastroenterology 2008; 55 (81): 120-126.
16. Koslowsky, B., Jacob, H., Eliakim, R., and Adler, S. N. PillCam ESO in esophageal studies: improved diagnostic yield of 14 frames per second (fps) compared with 4 fps. Endoscopy 2006; 38 (1): 27-30.
17. Sharma, Prateek Barrett esophagus: will effective treatment prevent the risk of progression to esophageal adenocarcinoma? The American journal of medicine 2004; 117 Suppl 5A: 79S-85S.
18. Ofman, Joshua J. Decision making in gastroesophageal reflux disease. What are the critical issues? Gastroenterology Clinics of North America 2002; 31 (4 Suppl): S67-S76.
19. Isolauri, J., Luostarinen, M., Isolauri, E., Reinikainen, P., Viljakka, M., and Keyrilainen, O. Natural course of gastroesophageal reflux disease: 17-22 year follow-up of 60 patients. The American journal of gastroenterology 1997; 92 (1): 37-41.
20. Kalsbeek, H. L. and van der Wouden, A. Barrett-oesophagus with papillomatous tumour. Archivum chirurgicum Neerlandicum 1971; 23 (4): 287-296.
21. Waheed, T. and Hurwitz, A. L. Elective upper gastrointestinal endoscopy in an outpatient endoscopy center: Impact on patient management. Practical Gastroenterology 2013; 37 (4): 38-46.
22. Lisovsky, M. and Srivastava, A. Barrett esophagus: Evolving concepts in diagnosis and neoplastic progression. Surgical Pathology Clinics 2013; 6 (3): 475-496.
23. Smoking Is an Independent Risk Factor for Barrett Esophagus. Clinical Advances in Hematology and Oncology 2012; 10 (10): 10.
24. Barrett's oesophagus: A low cancer risk. Medicine Today 2003; 4 (11): 10.
25. Conio, M., Cameron, A., Spechler, S., and Peters, J. H. Can reflux prevention prevent esophageal adenocarcinoma? Gastrointestinal endoscopy 2002; 56 (3): 457-461.
26. Loffeld, R. J. L. F. and van der Putten, A. B. M. M. Hiatal hernia, reflux-oesophagitis, and Barrett's oesophagus. A retrospective endoscopic and clinical study. Diseases of the Esophagus 1993; 6 (4): 57-61.
27. Parker, C., Alexandridis, E., Plevris, J., O'Hara, J., and Panter, S. Transnasal endoscopy: No gagging no panic! Frontline Gastroenterology 2016; 7 (4): 246-256.
28. Nasseri, Moghaddam S., Islami, L., Kalantarian, S., and Malekzadeh, R. Long term proton pump Inhibitor (PPI) use and incidence of gastric (pre) malignant lesions. Cochrane Database of Systematic Reviews 2008; (2).
29. Testoni, P. A., Mazzoleni, G., Distefano, G., Testoni, S. G., Antonelli, M., Fanti, L., and Passaretti, S. Transoral anterior fundoplication (TAF) with medigus ultrasound surgical endostapler (MUSE) for the treatment of gastroesophageal reflux disease (GERD): 12-months results from a single-center prospective study. Gastrointestinal endoscopy 2018. 87 (6 Supplement 1) AB235.
30. Saller, J., Diffalha, S. A., Neill, K., Oliveri, C., Levine, H., Boulware, D., and Coppola, D. CDX2 protein expression in esophageal biopsies without histological evidence of intestinal metaplasia may predict the presence of undetected Barrett's esophagus. Laboratory Investigation 2018. 98 (Supplement 1) 300.
31. Agoston, A., Dunbar, K. B., Souza, R., Spechler, S. J., and Odze, R. Comparison of HIF-2alpha and NF-kB proteins during development of acute reflux esophagitis in patients with and without long-segment barrett's esophagus. Laboratory Investigation 2018. 98 (Supplement 1) 240.
32. Hu, Z.-W., Tian, S.-R., Wu, J.-M., Wang, Z.-G., Zhang, Y., Wang, F., Du, X., and Zhang, D. General gastroscopy of gastroesophageal reflux disease: Analysis of 4086 cases. Medical Journal of Chinese People's Liberation Army 2018. 43 (1) 38-44.
33. Ortiz, P. and Henriquez, F. Esophageal carcinoma: A 20-year cohort study. American Journal of Gastroenterology 2017. 112 (Supplement 1) S189.
34. Enweluzo, C., Canning, A., and Hewlett, A. Does the degree of dysplasia affect post-endoscopy care of patients with Barrett's esophagus? American Journal of Gastroenterology 2017. 112 (Supplement 1) S632-S633.
35. Hoversten, P., Otaki, F., and Katzka, D. A. Candida esophagitis: Epidemiology, risk factors and outcomes. American Journal of Gastroenterology 2017. 112 (Supplement 1) S186-S187.
36. Barker, D. F., Dulaney, D. T., Dion, G. R., Stratton, A. N., and Gancayco, J. Impact of 24-hour PH/impedance on clinical outcomes at a tertiary care hospital. American Journal of Gastroenterology 2017. 112 (Supplement 1) S208.
37. Takeshita, E., Sakata, Y., Kawamura, S., Matsunaga, T., Tsuruoka, N., Miyahara, K., Tominaga, N., Matsunaga, K., Shimoda, R., Iwakiri, R., Kusano, M., and Fujimoto, K. Frequent upper gastrointestinal symptoms in japanese females compared to males did not depend on endoscopic esophagitis. American Journal of Gastroenterology 2017. 112 (Supplement 1) S207.
38. Umar, S., Rana, A., and Kapetanos, A. Long-term proton pump inhibitor use in primary care internal medicine resident clinic. American Journal of Gastroenterology 2017. 112 (Supplement 1) S597.
39. Smith, M. and Clayton, S. Wats 3D brush biopsy variability compared to traditional forceps biopsy in the identification of barrett's esophagus and dysplasia diagnosis. American Journal of Gastroenterology 2017. 112 (Supplement 1) S177.
40. Robles-Medranda, C., Valero, M., Soria-Alcivar, M., Puga-Tejada, M., Ospina-Arboleda, J., Alvarado-Escobar, H., and Pitanga-Lukashok, H. Minimal esophageal lesions detected by digital chromoendoscopy using optical enhancement systemTM associated to high definition plus optical magnification in non-erosive reflux disease (NERD). American Journal of Gastroenterology 2017. 112 (Supplement 1) S176.
41. Reymunde, A. and Santiago, N. Fifteen plus years after stretta antireflux endoscopy procedure: Quality of life and PPI requirements. American Journal of Gastroenterology 2017. 112 (Supplement 1) S459-S460.
42. Sahebjam, F., Katoh, A., Knight, K., Grider, D., and Bern, M. J. Expression of MUC-2 and CDX-2 in mucosal brushings accurately predicts Barrett's esophagus (BE): 1 step closer to a non-invasive be screening test. American Journal of Gastroenterology 2017. 112 (Supplement 1) S225.
43. Lonie, J., Smith, S., Avramovic, J., and Baker, S. The incidence of pre-operative asymptomatic gastr-oesophageal reflux disease (gord) in a bariatric surgical population. integrated health/multidisciplinary care. Obesity surgery 2017. 27 (1 Supplement 1) 647.
44. Almontashery, A. Evidence of objective endoscopic gastroesophageal reflux post sleeve gastrectomy Post-operative complications. Obesity surgery 2017. 27 (1 Supplement 1) 155.
45. Genco, A., Ernesti, I., Soricelli, E., Casella, G., Leone, G., Petrucci, F. M., Monti, M., and Cipriano, M. Mid-term follow-up (3 years) of patients with barrett's esophagus after sleeve gastrectomy Post-operative complications. Obesity surgery 2017. 27 (1 Supplement 1) 758.
46. Itskoviz, D., Vilkin, A., Zvidi, I., Niv, Y., and Dickman, R. What is the clinical importance of an incidental irregular Z line? a long-term follow-up study. United European Gastroenterology Journal 2016. 4 (5 Supplement 1) A689.
47. Robles-Medranda, C., Valero, M., Soria, M., Puga, M., Ospina, J., Alvarado, H., and Pitanga, Lukashok H. Minimal esophageal lesions detected by digital chromoendoscopy using optical enhancement systemTM associated to high definition plus optical magnification in non erosive reflux disease (NERD). United European Gastroenterology Journal 2016. 4 (5 Supplement 1) A381-A382.
48. Carvalhana, S., Bernardo, S., Freitas, L., Ferreira, C., Ribeiro, L. C., and Velosa, J. Low yield repeat upper GI endoscopy for dyspepsia patients: Consecutive serie from a tertiary referral center. United European Gastroenterology Journal 2016. 4 (5 Supplement 1) A489.
49. Maddalo, G., Pilotto, V., Savarino, E., Orlando, C., Fassan, M., Basso, D., Rugge, M., and Farinati, F. Prevalence and pathophysiology of gastroesophageal reflux disease in patients with autoimmune gastritis. United European Gastroenterology Journal 2017. 5 (5 Supplement 1) A799.
50. Woodland, P., Ooi, J. L. S., Grassi, F., Lee, C., Evans, J., Koukias, N., Triantos, C., Mcdonald, S. A. C., Peiris, M., Aktar, R., Blackshaw, A., and Sifrim, D. The location of oesophageal mucosal afferent nerves are more superficial in patients with nerd than in healthy volunteers and patients with barrett's oesophagus. United European Gastroenterology Journal 2017. 5 (5 Supplement 1) A795-A796.
51. Castela, J., Vinha, Pereira D., Mao De, Ferro S., Casaca, R., Fonseca, R., Chaves, P., and Dias, Pereira A. Congenital or metaplastic: Evaluation of gastroesophageal neo-junctions to assess cardiac type epithelium origin. United European Gastroenterology Journal 2017. 5 (5 Supplement 1) A812.
52. Itskoviz, D., Tamary, H., Krasnov, T., Sahar, N., Zevit, N., Ben-Bassat, O., Leibovici, Wiseman Y., Boltin, D., Goldberg, Y., and Levi, Z. Esophageal reflux disease and esophageal squamous cell cancer in patients with fanconi anemia undergoing endoscopic surveillance. United European Gastroenterology Journal 2017. 5 (5 Supplement 1) A361.
53. Lin, E. C., Holub, J., Lieberman, D. A., and Hur, C. Esophagogastroduodenoscopy in the evaluation of gastroesophageal reflux disease without symptoms: improved endoscopic outcomes from 2003 to 2014. Gastrointestinal endoscopy 2017. 85 (5 Supplement 1) AB422.
54. Testoni, P. A., Testoni, S. G., Mazzoleni, G., Fanti, L., and Passaretti, S. Transoral anterior fundoplication (TAF) with medigus ultrasound surgical endostapler (MUSETM) for the treatment of gastroesophageal reflux disease (GERD): 6-month results from a single-center prospective study. Gastrointestinal endoscopy 2017. 85 (5 Supplement 1) AB562.
55. Noar, M. D., Squires, P., and Kahn, S. Spontaneous regression of barrett's mucosa occurs following sphincter targeted endoluminal gerd therapy. Gastrointestinal endoscopy 2017. 85 (5 Supplement 1) AB584-.
56. Testoni, P. A., Testoni, S. G. G., Mazzoleni, G., Fanti, L., and Passaretti, S. Transoral anterior fundoplication (TAF) with medigus ultrasound surgical endostapler (MUSETM) for the treatment of gastroesophageal reflux disease (GERD): 6-month results from a single-center prospective study. Digestive and Liver Disease 2017. 49 (Supplement 2) e86.
57. Nason, K. S., Levy, R. M., Sarkaria, I. S., Vaughan, T., Switzer, G. E., and Luketich, J. D. Current screening recommendations for Barrett's esophagus do not differentiate esophageal cancer patients from population controls. Gastroenterology 2017. 152 (5 Supplement 1) S1244.
58. Schlottmann, F., Andolfi, C., Herbella, F. A. M., and Patti, M. G. GERD and hiatal hernia: Presence and size infuence the clinical presentation, the esophageal function and reflux profile. Gastroenterology 2017. 152 (5 Supplement 1) S1215.
59. Smukalla, S., Pitman, M., Khan, A., Popov, V., and Thompson, C. C. Sleeve gastrectomy is a risk factor for Barrett's esophagus: A systematic review and meta-analysis. Gastroenterology 2017. 152 (5 Supplement 1) S457-S458.
60. Nwachokor, J., Gunewardena, S., Sharma, M., De, A., Sharma, P., Christenson, L., and Bansal, A. Use of molecular analysis to inform clinical management of black patients with Barrett's esophagus. Gastroenterology 2017. 152 (5 Supplement 1) S455-S456.
61. Woodland, P. J., Grassi, F., Peiris, M., Aktar, R., Ooi, J. L., Evans, J., Lee, C., McDonald, S. A., Blackshaw, L. A., and Sifrim, D. Innervation pattern of the distal mucosal squamous epithelium may underlie hyposensitivity to acid reflux in patients with Barrett's oesophagus. Gastroenterology 2017. 152 (5 Supplement 1) S236.
62. Lin, E. C., Holub, J., Lieberman, D. A., and Hur, C. Low risk of suspected long segment Barrett's esophagus in gastroesophageal reflux disease without alarm symptoms. Gastroenterology 2017. 152 (5 Supplement 1) S453-S454.
63. Hejazi, R., DeVault, K. R., and Francis, D. L. Case-control study of the prevalence of Barrett's esophagus in patients with a Schatzki ring. Gastroenterology 2017. 152 (5 Supplement 1) S453.
64. Saleh, M. A., Mansoor, E., Perry, Y., Lee, P. J., and Chak, A. Proportion of gastroesophageal reflux disease and Barrett's esophagus in laparoscopic sleeve gastrectomy vs. Roux-En-Y gastrojejunostomy: A population based study. Gastroenterology 2017. 152 (5 Supplement 1) S453.
65. Westerveld, D. R., Khullar, V., Mramba, L. K., Ayoub, F., Brar, T., Agarwal, M., Chakraborty, J., Riverso, M., Perbtani, Y. B., Draganov, P. V., and Yang, D. Adherence to quality indicators for the diagnosis and management of Barrett's esophagus: A single-center retrospective analysis. Gastroenterology 2017. 152 (5 Supplement 1) S448-S449.
66. Okusanya, O., Dhupar, R., Luketich, J. D., Apfel, A., Bertolet, M., Vaughan, T., Switzer, G. E., and Nason, K. S. Assessment of GERD frequency, severity and duration: patterns in esophageal adenocarcinomas cases do not differ from population controls. Gastroenterology 2017. 152 (5 Supplement 1) S1265.
67. Aguirre, A. P., Khoury, H., Rivas, H., and Morton, J. M. Prevalence of pre-operative endoscopic findings for sleeve gastrectomy patients. Gastroenterology 2017. 152 (5 Supplement 1) S1260.
68. Khalaf, M. H., Brock, A., and CASTELL, D. O. Sporadic fundic gland polyps and level of gastric acid suppression. Gastroenterology 2017. 152 (5 Supplement 1) S470.
69. Khasag, O., Boldbaatar, G., Namdag, B., and Nyamaa, B. Prevalence of gastroesophageal reflux disease among mongolian population. Gastroenterology 2017. 152 (5 Supplement 1) S660.
70. Jovani, M., Cao, Y., Feskanich, D., Hur, C., Jacobson, B. C., and Chan, A. T. Aspirin use is associated with lower risk of Barrett's esophagus in women. Gastroenterology 2017. 152 (5 Supplement 1) S105.
71. Kwak, Y. E., Saleh, A., Sanchez, M. J., and Masoud, A. Effectiveness of esophagogastroduodenoscopy in changing treatment measures in patients with refractory gastroesophageal reflux disease. Gastroenterology 2017. 152 (5 Supplement 1) S657.
72. Nwachokor, J., Gunewardena, S., De, A., Sharma, M., Sharma, P., Christenson, L., and Bansal, A. Cellular pathways are differentially activated in black versus white patients with gastroesophageal reflux disease: Implications for race-based disease pathogenesis. Gastroenterology 2017. 152 (5 Supplement 1) S663.
73. Zavala-Solares, M. R., Valdovinos, M. A., Fonseca-Camarillo, G., Grajales-Figueroa, G., Zamora-Nava, L. E., Aguilar-Olivos, N. E., Valdovinos-Garcia, L. R., and Yamamoto-Furusho, J. K. Expression of genes associated with inflammation in biopsies of esophageal mucosa of different phenotypes of gastroesophageal reflux disease. Gastroenterology 2017. 152 (5 Supplement 1) S237.
74. Kataria, R., Rosenfeld, B., Malik, Z. A., Harrison, M. J., Schey, R., Parkman, H. P., and Smith, M. S. Distal esophageal impedance measurements during high resolution esophageal manometry with impedance (HREMI) predicts presence and length of Barrett's esophagus (BE). Gastroenterology 2017. 152 (5 Supplement 1) S458.
75. Wang, K., Duan, L., Liu, Z., He, Z., Guo, C., He, Y., Zhang, L., Jin, Z., and Ke, Y. A population-based survey for the risk factors of GERD in an area with high esophageal cancer incidence of China. Journal of gastroenterology and hepatology 2017. 32 (Supplement 3) 32-33.
76. Zehetner, J., Rona, K. A., Yu, A., Bildzukewicz, N., Houghton, C., and Lipham, J. C. Hiatal hernia recurrence rate and durability after magnetic sphincter augmentation in patients with large hiatal hernias and gastroesophageal reflux disease. Hernia 2017. 21 (2 Supplement 1) S174.
77. Zhou, J., Shrestha, P., Ho, V., Turner, I., Teoh, W.-C., and Al-Sohaily, S. Stress proteins in oesophageal epithelium within the gastro-oesophageal diseases. Journal of Gastroenterology and Hepatology (Australia) 2017. 32 (Supplement 2) 63-64.
78. D'Alessandro, A., Esposito, G., Pesce, M., Cargiolli, M., Zito, F. P., Seguella, L., Gigli, S., De Palma, G. D., Cuomo, R., and Sarnelli, G. Role of S100beta protein in metaplasia-dysplasia-carcinoma sequence in Barrett's esophagus. Neurogastroenterology and Motility 2017. 29 (Supplement 1) 13.
79. Rona, K. A., Yu, A., Houghton, C., Bildzukewicz, N., and Lipham, J. C. Hiatal hernia recurrence rate and durability after magnetic sphincter augmentation in patients with large hiatal hernias and gastroesophageal reflux disease. Surgical Endoscopy and Other Interventional Techniques 2017. 31 (Supplement 1) S62.
80. Aguirre-Mar, D., Vazquez, G., Giasi, E., and Rickman, M. T. Initial experience of electro stimulation device (Endostim) therapy on patients with recurrent gerd after fundoplication failure. Preliminary report. Surgical Endoscopy and Other Interventional Techniques 2017. 31 (Supplement 1) S257.
81. Bell, R., Tripp Buckley, F. P., Freeman, K., Heidrick, R., and Doggett, S. Magnetic sphincter augmentation during repair of paraesophageal and large hiatal hernias results in favorable outcomes. Surgical Endoscopy and Other Interventional Techniques 2017. 31 (Supplement 1) S55.
82. Silva, L. E., Ruy, Jorge C., Ribeiro, P. C. P., Alves, M. M., El Ajouz, T. K., and Silva, R. A. Simple, safe and effective. the MGB added to nissen surgery (MGBN): 15-Year outcomes of 1452 cases from a single surgeon. Surgical Endoscopy and Other Interventional Techniques 2017. 31 (Supplement 1) S286.
83. Chen, G. L., Eisenberg, D., and Kubat, E. Laparoscopic Roux-En-Y gastric bypass in veterans with barrett's esophagus. Surgical Endoscopy and Other Interventional Techniques 2017. 31 (Supplement 1) S132.
84. Shirai, S., Takeshita, E., Kawakubo, H., Sakata, Y., Tominaga, N., Miyahara, K., Morisaki, T., Iwakiri, R., Kusano, M., and Fujimoto, K. Medical health checkups indicated high frequency of reflux and dyspeptic symptoms in females than males regardless of endoscopic esophagitis. Diseases of the Esophagus 2016. 29 (Supplement 1) 56A.
85. Yoshida, T., Nishino, T., Inoue, S., Matsumoto, D., Takasugi, H., Inui, T., Morimoto, M., Okumura, K., Takechi, H., and Tangoku, A. Implications for improvement of gastroesophageal reflux disease after esophagectomy. Diseases of the Esophagus 2016. 29 (Supplement 1) 62A.
86. Offman, J., Pesola, F., Fitzgerald, R., Hamilton, W., and Sasieni, P. Incidence and progression of Barrett's oesophagus in clinical practice: A cohort analysis for planning of the Barrett's ESophagus Trial 3 (BEST3) comparing the Cytosponge-TFF3 test with usual care. European Journal of Surgical Oncology 2016. 42 (11) S252-S253.
87. Haboubi, H. N., Williams, L., Manson, J., Al-Mossawi, N., Rees, B., Lawrence, R., Bodger, O., Griffiths, P., Thornton, C., and Jenkins, G. J. Blood-based biomarkers in the oesophageal cancer model - Results from the piga mutant phenotype study. Gut 2016. 65 (Supplement 1) A31-A32.
88. Preedy, R., Phillpotts, S., El, Menabawey T., and Besherdas, K. What is the value of performing an endoscopy in patients under the age of 50 with symptoms of gastro-oesophageal reflux disease. Gut 2016. 65 (Supplement 1) A67.
89. Wang, K., Duan, L., Zhang, L., Liu, Z., He, Z., and Ke, Y. The prevalence of GERD subtypes in the natural population with high esophageal cancer incidence of China. Journal of Gastroenterology and Hepatology (Australia) 2016. 31 (Supplement 3) 23.
90. Takalkar, U. V., Kulkarni, U., and Reddy, D. N. Diagnostic yield of upper gastrointestinal endoscopy with reference to Barrett's esophagus in a tertiary care center from India. Journal of Gastroenterology and Hepatology (Australia) 2016. 31 (Supplement 3) 26.
91. Yin, C., Zhang, J., and Wang, J. Prevalence and risk factors of Barrett's esophagusin Northwest China. Journal of Gastroenterology and Hepatology (Australia) 2016. 31 (Supplement 3) 24.
92. Boldbaatar, G., Tegshee, T., Khasag, O., Duger, D., Adiyasuren, B., Tomohisa, U., Subsomwong, P., Takeshi, M., Tsogt-Ochir, B., and Yoshio, Y. Validation of serum markers of precancerous condition in Mongolia, where burdens high gastric cancer. Journal of Gastroenterology and Hepatology (Australia) 2016. 31 (Supplement 3) 91.
93. Cooper, S. C., Prew, S., Podmore, L., and Trudgill, N. J. Gastro-oesophageal reflux symptoms and the development of oesophageal adenocarcinoma: Comparison with community and reflux oesophagitis controls from moses (midlands oesophageal adenocarcinoma epidemiology study). Gut 2010. 59 (Supplement 1) A114.
94. Powell, N., Russo, E. A., Hoare, J., Teare, J., Negus, R., Thomas, H., and Orchard, T. R. Longitudinal analysis of erosive and non-erosive gastro-oesophageal reflux disease. Gut 2010. 59 (Supplement 1) A25-A26.
95. NCT01288612. Comparative Effectiveness of Endoscopic Assessment of Gastroesophageal Reflux and Barretts Esophagus. Https://clinicaltrials.gov/show/nct01288612. 2011.

### Other language (n=136)

1. Pech, Oliver [Gastroesophageal reflux disease and Barrett's dysplasia]. Deutsche medizinische Wochenschrift (1946) 2015; 140 (20): 1533-1536.
2. Rodriguez-DJesus, A., Gordillo, J., Uchima, H., Araujo, I., Saperas, E., Elizalde, I., and Fernandez-Esparrach, G. Prevalence and epidemiology of Barrett's esophagus in the province of Barcelona. Gastroenterologia y hepatologia 2014; 37 (7): 397-401.
3. Corvinus, F. M., Grimminger, P. P., and Lang, H. Multimodal treatment of early esophagial neoplasms. Internistische Praxis 2016; 56 (2): 253-262.
4. Kroupa, R. Barrett's esophagus, risk factors and management. Interni Medicina pro Praxi 2012; 14 (3): 104-106.
5. Marzo, M., Carrillo, R., Mascort, J. J., Alonso, P., Mearin, F., Ponce, J., Ferrandiz, J., Fernandez, M., Martinez, G., Saez, M., Bonfill, X., and Pique, J. M. Management of patients with GERD. Clinical practice guideline. 2008 update. Clinical Practice Guideline Working Group on GERD. Gastroenterologia y hepatologia 2009; 32 (6): 431-464.
6. Lubbers, H., Mahlke, R., Lankisch, P. G., and Stolte, M. Follow-up endoscopy in gastroenterology: When is it helpful? Deutsches Arzteblatt 2010; 107 (3): 30-.
7. Lukas, K., Bures, J., Drahonovsky, V., Jirasek, V., Martinek, J., Richter, P., Strosova, A., Hep, A., Mandys, V., Dite, P., and Svab, J. Gastroesophageal reflux disease updated guidelines of Czech gastroenterology society 2009. Ceska a Slovenska Gastroenterologie a Hepatologie 2009; 63 (2): 76-85.
8. Lin, S., Xu, G., and Hu, P. Chinese consensus on gastroesophageal reflux disease. Chinese Journal of Gastroenterology 2007; 12 (4): 233-239.
9. Messmann, H., Ell, C., Fein, M., Kiesslich, R., Ortner, M., Porschen, R., and Stolte, M. Consensus Conference on the DGVS on Gastroesophageal reflux. Subject group VI: Barrett's esophagus. Zeitschrift fur Gastroenterologie 2005; 43 (2): 184-190.
10. Seifert, B., Vojtiskova, J., Charvatova, E., and Koudelka, T. Management of gastroesophageal reflux disease (GERD) in primary care. Ceska a Slovenska Gastroenterologie a Hepatologie 2006; 60 (4): 157-162.
11. Sharma, P. Barrett's esophagus: From reflux disease to cancer. Therapeutic Research 2005; 26 (4): 701-713.
12. Fibbe, C., Keller, J., and Layer, P. Short practice guideline for the management of gastroesophageal reflux disease. Deutsche Medizinische Wochenschrift 2005; 130 (34-35): 1970-1973.
13. Endlicher, E., Knuchel, R., and Messmann, H. Surveillance of patients with Barrett's esophagus. Zeitschrift fur Gastroenterologie 2001; 39 (8): 593-600.
14. Zhang, Q.-Z., Yang, Q., Feng, J., Zhang, S.-X., and Wang, X.-R. Digital chrome endoscopy for diagnosis of Barrett's esophagus. World Chinese Journal of Digestology 2014; 22 (18): 2578-2582.
15. Aust, D. E. and Baretton, G. B. Barrett's esophagus: Indicators for cancer progression. Gastroenterologe 2013; 8 (6): 487-494.
16. Calvet, X. Gastroesophageal reflux disease and Barrett's esophagus: Epidemiology, diagnosis and treatment. Gastroenterologia y hepatologia 2011; 34 (SUPPL. 1): 28-34.
17. Wallner, G., Chibowski, D., Misiuna, P., Sory, A., Abramowicz, K., and Skomra, D. Comparative value of esophagoscopy and biopsy after antireflux procedure in patients operated on due to gastroesophageal reflux disease. Gastroenterologia Polska 1997; 4 (2): 125-134.
18. Kashin, S. V. and Ivanikov, I. O. [Barrett esophagus: modern diagnosis, drug therapy and reduce risk of cancer] [Russian]. Eksperimental'Naia I Klinicheskaia Gastroenterologiia 2009; (2): 90-98.
19. Tsukanov, V. V., Kasparov, E. V., Onuchina, E. V., Vasyutin, A. V., Butorin, N. N., Amelchugova, O. S., and Tonkikh, Yu L. [The frequency and clinical aspects of extraesophageal syndromes in elderly patients with gastroesophageal reflux disease]. Terapevticheskii arkhiv 2016; 88 (2): 28-32.
20. Kicinski, Przemyslaw, Mokrowiecka, Anna, Czkwianianc, Elzbieta, Kolasa-Kicinska, Marzena, Wozniakowska-Gesicka, Teresa, and Malecka-Panas, Ewa [The role of selected risk factors in Barrett's esophagus development]. Polski merkuriusz lekarski: organ Polskiego Towarzystwa Lekarskiego 2009; 26 (155): 390-394.
21. Demura, T. A., Kogan, E. A., Sklianskaia, O. A., and Mol', R. [Role of tight junction claudins in the morphogenesis of adenocarcinoma in the presence of Barrett's esophagus]. Arkhiv patologii 2008; 70 (5): 20-24.
22. Martinez de Haro, Luisa F., Munitiz, Vicente, Ortiz, Angeles, Ruiz de Angulo, David, Navarro, M. Dolores, and Parrilla, Pascual [Outpatient monitoring of oesophageal pH with a catheter-free pH-meter (Bravo System). A Study of tolerance, safety and efficacy]. Cirugia espanola 2008; 84 (4): 201-209.
23. Kim, Byung Chang, Yoon, Young Hoon, Jyung, Hyun Soo, Chung, Jae Bock, Chon, Chae Yun, Lee, Sang In, and Lee, Yong Chan [Clinical characteristics of gastroesophageal reflux diseases and association with Helicobacter pylori infection]. The Korean journal of gastroenterology = Taehan Sohwagi Hakhoe chi 2006; 47 (5): 363-369.
24. Carmona-Sanchez, Ramon and Navarro-Cano, Gregorio [Prevalence of Helicobacter pylori infection in patients with reflux esophagitis. A case-control study]. Revista de gastroenterologia de Mexico 2003; 68 (1): 23-28.
25. Lagergren, J. [Increased incidence of adenocarcinoma of the esophagus and cardia. Reflux and obesity are strong and independent risk factors according to the SECC study]. Lakartidningen 2000; 97 (16): 1950-1953.
26. Fahrlander, H. [Pathophysiology, clinical aspects and therapy of gastro-esophageal reflux disease]. Schweizerische medizinische Wochenschrift 1981; 111 (16): 550-555.
27. Wienbeck, M. [Benign diseases of the esophagus. An internist's view]. Zeitschrift fur Gastroenterologie 1976; 14 Suppl: 138-147.
28. Inda, Vargas, V, Torices, Escalante E., Dominguez, Camacho L., and Botello, Hernandez Z. Endoscopic and histopathological diagnosis of Barrett's esophagus compared, for the regional hospital 1.degree de Octubre (Instituto de Seguridad y Servicios Sociales de los Trabajadores del Estado) from may 2011 to may 2012. Endoscopia 2014; 26 (3): 83-86.
29. Yin, X., Xu, Y.-L., Zhou, J., and Chang, Y. Relationship among symptoms, endoscopic classification and pathological characteristics of Barrett esophagus. Journal of Shanghai Jiaotong University (Medical Science) 2013; 33 (1): 50-55.
30. Helman, L., Biccas, B. N., Lemme, E. M. O., Novais, P., and Fittipaldi, V. Esophageal manometry findings and degree of acid exposure in short and long Barrett's esophagus. Arquivos de Gastroenterologia 2012; 49 (1): 64-68.
31. He, S., Chen, Y., Xu, J., Tang, Y., Yao, Y., Sun, Y., and Ding, X. Analysis of esophageal motility in patients with nonerosive reflux disease. Chinese Journal of Gastroenterology 2011; 16 (8): 487-489.
32. Shi, X., Hao, H., Wang, L., Zhang, Y., and Ma, C. Study on reflux esophagitis, Barrett's esophagus and esophageal adenocarcinoma. Chinese Journal of Gastroenterology 2010; 15 (4): 233-236.
33. Kunsch, S., Neesse, A., Linhart, T., Steinkamp, M., Fensterer, H., Adler, G., Gress, T. M., and Ellenrieder, V. Impact of pantoprazole on duodeno-gastro-esophageal reflux (DGER). Zeitschrift fur Gastroenterologie 2009; 47 (3): 277-282.
34. Zhang, L.-Y., Lan, Y., and Wang, Q. Etiological differences for different types of gastroesophageal reflux disease. World Chinese Journal of Digestology 2009; 17 (8): 829-833.
35. Kunsch, S., Neesse, A., Huth, J., Steinkamp, M., Klaus, J., Adler, G., Gress, T. M., and Ellenrieder, V. Increased duodeno-gastro-esophageal reflux (DGER) in symptomatic GERD patients with a history of cholecystectomy. Zeitschrift fur Gastroenterologie 2009; 47 (8): 744-748.
36. De Freitas, M. C., Moretzsohn, L. D., and Coelho, L. G. V. Prevalence of Barrett's esophagus in individuals without typical symptoms of gastroesophageal reflux disease. Arquivos de Gastroenterologia 2008; 45 (1): 46-49.
37. Ringhofer, C., Lenglinger, J., Izay, B., Kolarik, K., Zacherl, J., Eisler, M., Wrba, F., Chandrasoma, P. T., Cosentini, E. P., Prager, G., and Riegler, M. Histopathology of the endoscopic esophagogastric junction in patients with gastroesophageal reflux disease. Wiener Klinische Wochenschrift 2008; 120 (11-12): 350-359.
38. Kula, Z. and Welshof, A. The prevalence of Barrett's oesophagus in own material of 6326 endoscopies. Gastroenterologia Polska 2007; 14 (2): 85-89.
39. Lenglinger, J., Ringhofer, C., Eisler, M., Sedivy, R., Wrba, F., Zacherl, J., Cosentini, E. P., Prager, G., Haefner, M., and Riegler, M. Histopathology of columnar-lined esophagus in patients with gastroesophageal reflux disease. Wiener Klinische Wochenschrift 2007; 119 (13-14): 405-411.
40. Svoboda, P., Ehrmann, J., Klvana, P., Machytka, E., Rydlo, M., and Hrabovsky, V. Endoscopic findings in upper gastrointestinal tract in patients with liver cirrhosis. Vnitrni Lekarstvi 2007; 53 (9): 968-971.
41. Pulanic, R. Gastroesophageal reflux disease. Medicus 2006; 15 (1): 25-37.
42. Hartmann, D., Kudis, V., Jakobs, R., Riemann, J. F., and Schilling, D. Course of erosive gastroesophageal reflux disease (ERD) - A prospective examination under therapeutic daily life conditions with a mean follow-up of 6.5 years. Zeitschrift fur Gastroenterologie 2006; 44 (10): 1039-1042.
43. Bajbouj, M., Reichenberger, J., Neu, B., Prinz, C., Schmid, R. M., Rosch, T., and Meining, A. A prospective multicenter clinical and endoscopic follow-up study of patients with gastroesophageal reflux disease. Zeitschrift fur Gastroenterologie 2005; 43 (12): 1303-1307.
44. Chen, X., Ouyang, Q., Zhang, W.-Y., Li, X.-S., and Liang, H.-L. The clinical, pathological features and expression of Ki-67 and COX-2 in severe reflux esophagitis and Barrett's esophagus. Journal of Sichuan University (Medical Science Edition) 2005; 36 (2): 207-209.
45. Huppe, D., Felten, G., and Gillessen, A. Gastroesophageal reflux disease and esophageal carcinoma. Is there an increase of Barrett's carcinomas? Verdauungskrankheiten 2002; 20 (3): 91-95.
46. D'Addazio, G., Conio, M., Aste, H., Cestari, R., Ravelli, P., and Missale, G. Gastroesophageal reflux and Barrett's esophagus: Correlations among endoscopy, histological and clinical evidence. Giornale Italiano di Endoscopia Digestiva 2000; 23 (1): 33-40.
47. De, Salvo L., Arezzo, A., Percivale, A., and Gianiorio, F. Personal experience in Barrett's esophagus treatment. Giornale Italiano di Endoscopia Digestiva 1997; 20 (2): 71-75.
48. Santo, Marco Aurelio, Quintanilha, Sylvia Regina, Mietti, Cesar Augusto, Kawamoto, Flavio Masato, Marson, Allan Garms, and de Cleva, Roberto. Endoscopic Changes Related to Gastroesophageal Reflux Disease: Comparative Study Among Bariatric Surgery Patients. Arquivos brasileiros de cirurgia digestiva: Brazilian archives of digestive surgery 2015; 28 Suppl 1: 36-38.
49. Rodriguez-D'Jesus, Antonio, Gordillo, Jordi, Uchima, Hugo, Araujo, Isis, Saperas, Esteban, Elizalde, Ignasi, and Fernandez-Esparrach, Gloria [Prevalence and epidemiology of Barrett's esophagus in the province of Barcelona]. Gastroenterologia y hepatologia 2014; 37 (7): 397-401.
50. Zsolt, Simonka, Paszt, Attila, Geczi, Tibor, Abraham, Szabolcs, Toth, Illes, Horvath, Zoltan, Pieler, Jozsef, Tajti, Janos, Varga, Akos, Tiszlavicz, Laszlo, Nemeth, Istvan, Izbeki, Ferenc, Rosztoczy, Andras, Wittmann, Tibor, and Lazar, Gyorgy [Comparison of surgical patients with gastroesophageal reflux disease and Barrett's esophagus]. Magyar sebeszet 2014; 67 (5): 287-296.
51. Riegler, M., Asari, R., Cosentini, E. P., Wrba, F., and Schoppmann, S. F. [Critical assessment of a new endoscopic anatomic concept for the so-called cardia in the sense of the notions of Parmenides and Martin Heidegger]. Zeitschrift fur Gastroenterologie 2014; 52 (4): 367-373.
52. Kirova, M. V. [Confocal laser endo-microscopy and confirming endoscopic procedures in the diagnosis of Barrett's esophagus]. Eksperimental'naia i klinicheskaia gastroenterologiia = Experimental & clinical gastroenterology 2012; (4): 98-103.
53. Choi, Cheul Young, Suh, Seungchul, Park, Jae Serk, Lee, Hyun Jeong, Lee, Jong Sup, Choi, Hyo Sun, Park, Hyun Sung, and Hong, Seung Goun [The prevalence of Barrett's esophagus and the comparison of Barrett's esophagus with cardiac intestinal metaplasia in the health screening at a secondary care hospital]. The Korean journal of gastroenterology 2012; 60 (4): 219-223.
54. de Faria, Plinio Conte Jr, Andreollo, Nelson Adami, Trevisan, Miriam Aparecida da Silva, and Lopes, Luiz Roberto [Relationship of the sialomucins (Tn and Stn antigens) with adenocarcinoma in Barrett's esophagus]. Revista da Associacao Medica Brasileira (1992) 2007; 53 (4): 360-364.
55. Lopes, L. R., Brandalise, N. A., Andreollo, N. A., and Leonardi, L. S. [Videolaparoscopic surgical treatment of gastroesophageal reflux disease: modified Nissen technique - clinical and functional results]. Revista da Associacao Medica Brasileira (1992) 2001; 47 (2): 141-148.
56. Kroshchuk, V. V., Miasoiedov, S. D., Andreieshchev, S. A., Buryi, O. M., Fediuchek, A. S., Umanets', M. S., Petunin, Iu I., and Oleksiuk, O. V. [Remote results of the treatment of patients for Barrett's esophagus]. Klinichna khirurhiia / Ministerstvo okhorony zdorov'ia Ukrainy, Naukove tovarystvo khirurhiv Ukrainy 2010; (5): 5-10.
57. Stiefelhagen, Peter [Barrett esophagus. Overkill for a killer?] MMW Fortschritte der Medizin 2008; 150 (45): 20.
58. Darvin, V. V. and Funygin, D. V. [Endoscopic chromoscopy in diagnosing and treatment of complicated gastroesophageal reflux disease]. Vestnik khirurgii imeni I.I.Grekova 2008; 167 (4): 30-31.
59. Hoara, P., Birla, R., Gindea, C., Constantin, A., Panaitescu, G., Iordan, N., and Constantinoiu, S. [The role of esophageal manometry in the management of the patients with gastro-esophageal reflux disease and Barrett's esophagus]. Chirurgia (Bucharest, Romania: 1990) 2008; 103 (4): 407-412.
60. Yoo, Seung Suk, Lee, Won Hyun, Ha, Jong, Choi, Sun Pil, Kim, Hyun Jin, Kim, Tae Hyo, and Lee, Ok Jae [The prevalence of esophageal disorders in the subjects examined for health screening]. The Korean journal of gastroenterology 2007; 50 (5): 306-312.
61. Blaszak, Antoni, Wojtun, Stanislaw, Gil, Jerzy, and Wojtkowiak, Marek [The analysis of hiatal hernia occurrence in connection with GERD]. Polski merkuriusz lekarski: organ Polskiego Towarzystwa Lekarskiego 2007; 22 (131): 357-361.
62. Lee, Sang Ho, Ryu, Chang Beom, Jang, Jae Young, and Cho, Joo Young [Magnifying endoscopy in upper gastrointestinal tract]. The Korean journal of gastroenterology 2006; 48 (3): 145-155.
63. Grater, H. [Gastroesophageal reflux disease]. Praxis 2005; 94 (48): 1899-1906.
64. Hanai, Masaaki, Kusano, Motoyasu, Kawamura, Osamu, Shimoyama, Yasuyuki, and Maeda, Masaki [Epidemiology of Barrett's esophagus--comparison of Japan and the West]. Nihon rinsho. Japanese journal of clinical medicine 2005; 63 (8): 1333-1339.
65. Holscher, A. H., Bollschweiler, E., Gutschow, Ch, and Malfertheiner, P. [Correct diagnosis for indication in gastroesophageal reflux disease]. Der Chirurg; Zeitschrift fur alle Gebiete der operativen Medizen 2005; 76 (4): 345-352.
66. Leers, J., Bollschweiler, E., and Holscher, A. H. [Symptoms in patients with adenocarcinoma of the esophagus]. Zeitschrift fur Gastroenterologie 2005; 43 (3): 275-280.
67. Neuhaus, H. [Barrett's esophagus]. Praxis 2004; 93 (47): 1951-1957.
68. Fiorentino, Eugenio, Cabibi, Daniela, Barbiera, Filippo, Pantuso, Gianni, Buscemi, Giuseppe, Latteri, Federica, Mastrosimone, Achille, and Valenti, Antonio [Hiatal hernia, gastro-oesophageal reflux and oesophagitis: videofluorographic, endoscopic and histopathological correlation]. Chirurgia italiana 2004; 56 (4): 483-488.
69. Csendes, Attila, Carcamo, Carlos, and Henriquez, Ana [Nutcracker esophagus. Analysis of 80 patients]. Revista Medica de Chile 2004; 132 (2): 160-164.
70. Fuessl, Hermann Sebastian [Heartburn--a symptom or an illness?] Medizinische Klinik (Munich, Germany : 1983) 2004; 99 (5): 237-250.
71. Endzinas, Z., Mickevicius, A., and Kiudelis, M. [The influence of Barrett's esophagus on the clinical signs and postoperative results of GERD]. Zentralblatt fur Chirurgie 2004; 129 (2): 99-103.
72. Caum, Leiber C., Bizinelli, Sergio L., Pisani, Julio Cesar, Amarantes, Heda Maria Barska dos Santos, Ioshii, Sergio O., and Carmes, Eliane R. [Specialized intestinal metaplasia of the distal esophagus in gastroesophageal reflux disease: prevalence and clinico-demographic features]. Arquivos de Gastroenterologia 2003; 40 (4): 220-226.
73. Zakharash, M. P., Ioffe, A. Iu, Zavernyi, D. G., Bekmuradov, A. R., Vasil'ev, A. V., and Kalashnikov, A. A. [Gastroesophageal reflux disease as a surgical problem]. Likars'ka sprava / Ministerstvo okhorony zdorov'ia Ukrainy 2003; (8): 90-94.
74. Stiefelhagen, Peter [Gastroesophageal reflux. Endoscopic control is mostly unnecessary]. MMW Fortschritte der Medizin 2003; 145 (50): 16.
75. Labenz, J. [Erosions, strictures, Barrett's esophagus? Investigate reflux thoroughly]. MMW Fortschritte der Medizin 2003; 145 (42): 69-70.
76. Fuessl, H. S. [Heartburn without esophagitis. Symptoms more important than the finding?]. MMW Fortschritte der Medizin 2003; 145 (39): 4-6.
77. Tagle Arrospide, M., Aguinaga Meza, M., and Vasquez Rubio, G. [Hiatal hernia as a risk factor for erosive esphagitis: experience and endoscopic findings of a Peruvian population with heartburn]. Revista de gastroenterologia del Peru: organo oficial de la Sociedad de Gastroenterologia del Peru 2003; 23 (1): 36-40.
78. Zaninotto, G., Costantini, M., Molena, D., Rizzetto, C., Ekser, B., and Ancona, E. [Barrett's esophagus. Prevalence, risk of adenocarcinoma, role of endoscopic surveillance]. Minerva chirurgica 2002; 57 (6): 819-836.
79. Allescher, H. D. [Diagnosis of gastroesophageal reflux]. Praxis 2002; 91 (18): 779-790.
80. Hollenz, M., Stolte, M., and Labenz, J. [Prevalence of gastro-oesophageal reflux disease in general practice]. Deutsche medizinische Wochenschrift (1946) 2002; 127 (19): 1007-1012.
81. von Schrenck, T. [Diagnosis of gastroesophageal reflux and Barrett esophagus]. Zentralblatt fur Chirurgie 2000; 125 (5): 414-423.
82. Hoshihara, Y. [Complications of gastroesophageal reflux disease. 2. Endoscopic diagnosis of Barrett esophagus--can Barrett esophagus be diagnosed by endoscopic observation alone?]. Nihon Naika Gakkai zasshi.The Journal of the Japanese Society of Internal Medicine 2000; 89 (1): 85-90.
83. Jaspersen, D., Diehl, K. L., Geyer, P., Martens, E., and Arps, H. [Benign proximal esophageal stenosis--mostly a complication of gastroesophageal reflux disease]. Deutsche medizinische Wochenschrift (1946) 1999; 124 (8): 205-208.
84. Schmidt, C. and Baumeister, B. [Gastroesophageal reflux disease]. Deutsche medizinische Wochenschrift (1946) 1999; 124 (3): 56-61.
85. Csendes, A., Smok, G., Christensen, H., Rojas, J., Burdiles, P., and Korn, O. [Prevalence of cardial or fundic mucosa and Helicobacter pylori in the squamous-columnar mucosa in patients with chronic patological gastroesophageal reflux without intestinal metaplasia comparated with controls]. Revista Medica de Chile 1999; 127 (12): 1439-1446.
86. Csendes, A., Burdiles, P., Smok, G., Rojas, J., Flores, N., Domic, S., Quiroz, J., and Henriquez, A. [Clinical and endoscopic findings and magnitude of gastric and duodenal reflux in patients with cardial intestinal metaplasia, short Barrett esophagus, compared with controls]. Revista Medica de Chile 1999; 127 (11): 1321-1328.
87. Csendes, A., Smok, G., Sagastume, H., and Rojas, J. [Biopsy and endoscopic prospective study of the prevalence of intestinal metaplasia in the gastroesophageal junction in controls and in patients with gastroesophageal reflux]. Revista Medica de Chile 1998; 126 (2): 155-161.
88. Wallner, G., Misiuna, P., Chibowski, D., Polkowski, W., Abramowicz, K., and Sory, A. [Postoperative evaluation of esophageal mucosal change dynamics in patients with reflux diseases]. Wiadomosci lekarskie (Warsaw, Poland : 1960) 1997; 50 Suppl 1 Pt 1: 273-281.
89. Csendes, A., Smok, G., Alvarez, F., Braghetto, I., and Blanco, C. [Patients with pathologic gastroesophageal reflux without erosive esophagitis: correlation of the endoscopic and histological aspect of the esophagus]. Revista Medica de Chile 1994; 122 (2): 159-163.
90. Pinero, R., Salomon, A., Gonzalez, R., Olavarria, R., and Poleo, J. R. [Barrett esophagus. Frequency, endoscopic, histologic, and clinical characteristics]. G.E.N 1990; 44 (1): 46-48.
91. Braghetto, I., Csendes, A., Diaz, J. C., Maluenda, F., and Henriquez, A. [Clinical and laboratory correlation of severe esophagitis compared to mild or moderate esophagitis]. Acta gastroenterologica Latinoamericana 1989; 19 (2): 75-82.
92. de Rezende, J. M., Rosa, H., Vaz, M. da, Andrade-Sa, N., Porto, J. D., Neves Neto, J., and Ximenes, J. A. [Endoscopy in megaesophagus. Prospective study of 600 cases]. Arquivos de Gastroenterologia 1985; 22 (2): 53-62.
93. Siewert, R., Koch, A., Stuhler, T., and Jennewein, H. M. [Cardia function and gastroesophageal reflux after distal gastric resection]. Zeitschrift fur Gastroenterologie 1974; 12 (8): 583-590.
94. Calvet, X. Oesophageal diseases: Gastroesophageal reflux disease, Barrett's disease, Achalasia and eosinophilic oesophagitis. Gastroenterologia y hepatologia 2015; 38: 49-55.
95. Karaca, A. S., Capar, M., and Ali, R. Laparoscopic nissen fundoplication, single center experience. Journal of Clinical and Analytical Medicine 2015; 6 (2): 205-207.
96. Oral. Journal of Gastroenterology and Hepatology (Australia) 2015; 30: 1-27.
97. Sharifi, A., Dolatshahi, S., Rezaeifar, A., and Ramim, T. Sensitivity and specificity of endoscopy in diagnosis for Barrett's esophagus. Tehran University Medical Journal 2014; 72 (6): 396-403.
98. Calvet, X. and Villoria, A. Esophageal diseases: GERD, barrett, achalasia and eosinophilic esophagitis. Gastroenterologia y hepatologia 2014; 37 (S3): 53-61.
99. Kroupa, R. Premalignant conditions of the esophagus. Klinicka Onkologie 2013; 26 (SUPPL): S17-S21.
100. Weiss, W. Treatment of gastroesophageal reflux disease (GERD) in Austria: LIFE observational program - Commentary. Journal fur Gastroenterologische und Hepatologische Erkrankungen 2009; 7 (4): 23-24.
101. Yang, H.-Y., Ge, Z.-Z., Dai, J., Li, X.-B., and Gao, Y.-J. Positive rate comparison between immunological and chemical methods of fecal occult blood test in upper digestive tract bleeding. World Chinese Journal of Digestology 2008; 16 (9): 946-950.
102. Gomez, Senent S., Raposo, C. G., and Cabral, J. M. S. Esophageal cancer. Medicina Clinica 2008; 130 (11): 423-428.
103. Kment, M. Barrett's esophagus - An ideal study model of carcinogenesis? Ceska a Slovenska Gastroenterologie a Hepatologie 2007; 61 (1): 47-54.
104. Belleri, G., Comini, L., Ramponi, G. P., Tabaglio, E., Archetti, G., Astori, P., Baldini, S., Baronchelli, M., Beltrami, G., Benini, F., Bertolotti, B., Bettoncelli, G., Bonardelli, E., Bovini, L., Chiappi, A., Ferrari, A., Guarnera, L., Mauro, N., Multineddu, M., Mutti, E., Palini, S., Palumbo, M., Pascarella, A., Pellizzari, R., Regazzoli, T., Sidari, G., Terranova, F. R., Zadra, A., and Zavanella, D. Peer review about gastro-esophageal reflux disease in primary care: Epidemiology, diagnostic and therapeutic management. Recenti Progressi in Medicina 2006; 97 (2): 74-78.
105. Hansmann, J. and Grenacher, L. Radiological imaging of the upper gastrointestinal tract. Part 1. The esophagus. Radiologe 2006; 46 (12): 1077-1088.
106. Trillo, Sallan E., Lopez Fananas, M. S., Villaverde Royo, M. V., and Isanta, Pomar C. Study of the gastroscopies requested at a health centre. Atencion Primaria 2005; 35 (7): 375-377.
107. Al-Tashi, M., Bures, J., Rejchrt, S., Kopacova, M., Siroky, M., Papik, Z., Repak, R., Tycova, V., Nozicka, J., Dedic, K., Langr, F., Bukac, J., Douda, T., Kupkova, B., Fejfar, T., Tacheci, I., Slezak, L., and Hulek, P. Barrett's esophagus. The occurrence and complications during 1994-2003. Ceska a Slovenska Gastroenterologie a Hepatologie 2005; 59 (2): 62-65.
108. Martinek, J., Hucl, T., and Spicak, J. The Prevalence of Helicobacter pylori Infection in Some Disease of the Esophagus, Stomach and Duodenum - A Retrospective Analysis. Ceska a Slovenska Gastroenterologie a Hepatologie 2003; 57 (6): 228-232.
109. Endlicher, E., Timmer, A., and Messmann, H. Does surveillance of patients with Barrett's esophagus improve survival? Zeitschrift fur Gastroenterologie 2003; 41 (7): 675-678.
110. Jaspersen, D., Diehl, K.-L., Geyer, P., Martens, E., and Arps, H. Benign stenosis of the proximal oesophagus, most often a complication of gastro-oesophageal reflux. Deutsche Medizinische Wochenschrift 1999; 124 (8): 205-208.
111. Rosch, W. Gastroesophageal reflux disease and Barrett's esophagus surveillance and therapy. Praxis 1994; 83 (25-26): 783-787.
112. Rosch, W. Esophagoscopy: indications. Fortschritte der Medizin 1974; 92 (5): 193-197.
113. Caroline, D. F. and Laufer, I. Double contrast examination of the esophagus. Revista Interamericana de Radiologia 1982; 7 (4): 119-125.
114. Lenglinger, Johannes, See, Stephanie Fischer, Beller, Lukas, Cosentini, Enrico P., Asari, Reza, Wrba, Fritz, Riegler, Martin, and Schoppmann, Sebastian F. Review on novel concepts of columnar lined esophagus. Wiener klinische Wochenschrift 2013; 125 (19-20): 577-590.
115. Bauerfeind, P., Mullhaupt, B., Schofl, R., Rosch, T., Schwizer, W., Wirth, H. P., Kullak-Ublick, G. A., and Fried, M. [Highlights in gastroenterology 2006]. Praxis 2006; 95 (46): 1793-1804.
116. Schepp, W., Allescher, H. D., Frieling, T., Katschinski, M., Malfertheiner, P., Pehl, C., Peitz, U., Rosch, W., and Hotz, J. [Topic complex I: Definitions, epidemiology and natural course]. Zeitschrift fur Gastroenterologie 2005; 43 (2): 165-168.
117. Holscher, A. H. and Blum, A. L. [Surgical and medical therapy for gastroesophageal reflux disease. Consensus of pro/contra clinical debate]. Zeitschrift fur Gastroenterologie 2003; 41 (8): 729-732.
118. Wang, Y.-K., Hu, H.-M., Hsu, W.-H., Wu, D.-C., and Kuo, C.-H. From gastroesophageal reflux disease to Barrett's esophagus. Journal of Internal Medicine of Taiwan 2012; 23 (4): 254-266.
119. Liu, F.-X., Wang, W.-H., and Shuai, X.-W. Prevalence of helicobacter pylori in patients with Barrett's esophagus: A meta-analysis. Chinese Journal of Evidence-Based Medicine 2008; 8 (12): 1086-1093.
120. Eickhoff, A. and Riemann, J. F. Clinical advances in gastroenterology. Deutsche Medizinische Wochenschrift 2006; 131 (25-26): 1452-1455.
121. Lutz, L. and Werner, M. [Barrett's esophagus and carcinoma: Recommendations of the S2k guideline 2014 and the S3 guideline 2015]. Der Pathologe 2016; 37 (2): 193-200.
122. Kinoshita, Yoshikazu, Ishimura, Norihisa, and Ishihara, Shunji [Revision process and present task: evidence-based clinical practice guidelines for gastroesophageal reflux disease]. Nihon rinsho. Japanese journal of clinical medicine 2015; 73 (7): 1190-1194.
123. Labenz, J. Barrett's esophagus. Internist 2016; 57 (11): 1079-1092.
124. Hongo, M. GERD Guideline Workshop Report 2002. Therapeutic Research 2003; 24 (5): 830-835.
125. Chen, Y., Liu, J., and Chen, S. Effect of proton pump inhibitor on gastric mucosal histological changes in patients with gastroesophageal reflux disease. Chinese Journal of Gastroenterology 2015; 20 (12): 717-721.
126. Vargas Cardenas, Gloria [Barrett's esophagus: prevalence and risk factors in the National Hospital Arzobispo Loayza in Lima-Peru]. Revista de gastroenterologia del Peru 2010; 30 (4): 284-304.
127. Rehfeld, J. F. and Gotze, J. P. Barrett's metaplasia and esophageal cancer. Ugeskrift for Laeger 2003; 165 (24): 2498.
128. Halm, U., Schoppmeyer, K., and Mossner, J. Pathogenesis of Barrett's esophagus and Barrett's carcinoma. Chirurgische Gastroenterologie Interdisziplinar 2001; 17 (1): 51-56.
129. Estevez-Fernandez, Sergio, Sanchez-Santos, Raquel, Marino-Padin, Esther, Gonzalez-Fernandez, Sonia, and Turnes-Vazquez, Juan Esophagogastric pathology in morbid obese patient: Preoperative diagnosis, influence in the selection of surgical technique. Revista espanola de enfermedades digestivas 2015; 107 (7): 408-412.
130. Nassif, Paulo Afonso Nunes, Pedri, Lucas Eduardo, Martins, Priscila Reis, Foauni, Marcelo Morikuni, Justen, Marcel da Silva, Varaschim, Michelle, Bopp, Denise Serpa, and Malafaia, Osvaldo Incidence and predisponent factors for the migration of the fundoplication by Nissen-Rossetti technique in the surgical treatment of GERD. Arquivos brasileiros de cirurgia digestiva: Brazilian archives of digestive surgery 2012; 25 (2): 75-80.
131. Herbella, F. A., Del Grande, J. C., Lourenco, L. G., Mansur, N. S., and Haddad, C. M. [Late results of Heller operation and fundoplication for the treatment of the megaesophagus: analysis of 83 cases]. Revista da Associacao Medica Brasileira (1992) 1999; 45 (4): 317-322.
132. Parini, U., Fosson, A., Murix, E. L., and Liotta, G. [Laparoscopic treatment of gastroesophageal reflux]. Chirurgia italiana 1999; 51 (2): 121-126.
133. Rotterdam, H. Pathology of the gastric cardia. Verhandlungen der Deutschen Gesellschaft fur Pathologie 1999; 83: 37-42.
134. Spechler, S. J. [Pathogenesis and epidemiology of Barrett esophagus]. Der Chirurg; Zeitschrift fur alle Gebiete der operativen Medizen 1994; 65 (2): 84-87.
135. Gomez, Senent S., Raposo, C. G., and Cabral, J. M. S. Esophageal cancer. Medicina clinica 2008; 130 (11): 423-428.
136. Morales-Fuentes, G. A., Zarate-Osorno, A., Quinonez-Urrego, E. E., Antonio-Manrique, M., Martinez-Garcia, C. L., Figueroa-Barojas, P., Zamorano-Orozco, Y., Leal-Osuna, S. E., Martinez-Camacho, C., Mejia-Cuan, L. A., Rivera-Nava, C. A., Sanchez-Chavez, X., and Ramirez-Ramirez, M. A. [p53 expression in the gastric mucosa of patients infected with Helicobacter pylori]. Revista de gastroenterologia de Mexico 2013; 78 (1): 12-20.

### Study design: Systematic review/Meta-analysis (n=13)

1. Song, Huan, Zhu, Jianwei, Lu, DongHao, and Cochrane Database of Systematic Reviews Long-term proton pump inhibitor (PPI) use and the development of gastric pre-malignant lesions. 2014; (12).
2. Curvers, W. L., Broek, F. J., Reitsma, J. B., Dekker, E., and Bergman, J. J. Systematic review of narrow-band imaging for the detection and differentiation of abnormalities in the esophagus and stomach (with video) (DARE structured abstract). Gastrointestinal endoscopy 2009; 69: 307-317.
3. Zagari, Rocco Maurizio, Eusebi, Leonardo Henry, Rabitti, Stefano, Cristoferi, Laura, Vestito, Amanda, Pagano, Nico, and Bazzoli, Franco Prevalence of upper gastrointestinal endoscopic findings in the community: A systematic review of studies in unselected samples of subjects. Journal of gastroenterology and hepatology 2016; 31 (9): 1527-1538.
4. Mallick, R., Patnaik, S. K., Wani, S., and Bansal, A. A Systematic Review of Esophageal MicroRNA Markers for Diagnosis and Monitoring of Barrett's Esophagus. Digestive diseases and sciences 2016; 61 (4): 1039-1050.
5. Astin, M. P., Martins, T., Welton, N., Neal, R. D., Rose, P. W., and Hamilton, W. Diagnostic value of symptoms of oesophagogastric cancers in primary care: A systematic review and meta-analysis. British Journal of General Practice 2015; 65 (639): e677-e691.
6. Thosani N, Dayyeh BK, Sharma P, Aslanian HR, Enestvedt BK, Komanduri S, Manfredi M, Navaneethan U, Maple JT, Pannala R, Parsi MA. ASGE Technology Committee systematic review and meta-analysis assessing the ASGE Preservation and Incorporation of Valuable Endoscopic Innovations thresholds for adopting real-time imaging–assisted endoscopic targeted biopsy during endoscopic surveillance of Barrett’s esophagus. Gastrointestinal endoscopy. 2016 Apr 30; 83 (4):684-98.
7. Ip S, Chung M, Moorthy D, Yu WW, Lee J, Chan JA, Bonis PA, Lau J. Comparative effectiveness of management strategies for gastroesophageal reflux disease: update.
8. Peeters M, Lerut T, Vlayen J, Mambourg F, Ectors N, Deprez P, et al. Guideline pour la prise en charge du cancer oesophagien et gastrique : éléments scientifiques à destination du Collège d’Oncologie. Bruxelles: Centre fédéral d'expertise des soins de santé (KCE); 2008. KCE Reports 75B (D/2008/10.273/17)
9. National Institute for Health and Care Excellence. Dyspepsia and gastrooesophageal reflux disease: investigation and management of dyspepsia, symptoms suggestive of gastro-oesophageal reflux disease, or both. London: NICE, September 2014.
10. Yang S, Wu S, Huang Y, Shao Y, Chen XY, Xian L, Zheng J, Wen Y, Chen X, Li H, Yang C. Screening for oesophageal cancer. Cochrane Database of Systematic Reviews 2012, Issue 12. Art. No.: CD007883. DOI: 10.1002/14651858.CD007883.pub2
11. Swedish Council on Health Technology Assessment. Dyspepsia and Gastro-oesophageal Reflux: A Systematic Review [Internet]. SBU Systematic Review Summaries. 2007.
12. Iqbal, Umair, Siddique, Osama, Ovalle, Anais, Anwar, Hafsa, and Moss, Steven F. Safety and efficacy of a minimally invasive cell sampling device ('Cytosponge') in the diagnosis of esophageal pathology: a systematic review. European journal of gastroenterology & hepatology 2018. 30 (11) 1261-1269.
13. Song, H., Zhu, J., and Lu, D. Long-term proton pump inhibitor (PPI) use and the development of gastric pre-malignant lesions. Cochrane Database of Systematic Reviews 2014. 2014 (12) CD010623.

### Study design: Protocol (n=30)

1. Kramer, J. R., Arney, J., Chen, J., Richardson, P., Duan, Z., Street, Jr, Hinojosa-Lindsey, M., Naik, A. D., and El-Serag, H. B. Patient-centered, comparative effectiveness of esophageal cancer screening: protocol for a comparative effectiveness research study to inform guidelines for evidence-based approach to screening and surveillance endoscopy. BMC health services research 2012; 12: 288.
2. Current Controlled Trials [Internet]. London: BioMed Central. [date unknown]. ISRCTN13256080, Barretts Oesophagus Screening Trial in a case-control study; 2011 June 16 [cited 2007 Feb 27]; Available from: <http://www.controlledtrials.com/ISRCTN13256080>
3. ISRCTN registry. Barrett’s oesophagus screening trial in a case control study. ISRCTN12730505 DOI 10.1186/ISRCTN12730505. Available from: <http://www.isrctn.com/ISRCTN12730505>
4. ISRCTN registry. Cancer of the oesophagus or gastricus: new assessment of the technology of endosonography. ISRCTN01444215 DOI 10.1186/ISRCTN01444215
5. ISRCTN registry. Randomised controlled trial of surveillance and no surveillance for patients with Barrett's oesophagus. SRCTN54190466 DOI 10.1186/ISRCTN54190466. Available from: <http://www.isrctn.com/ISRCTN54190466>
6. ISRCTN registry. Endoscopic Tri-Modal Imaging for the detection of early neoplasia in patients with Barrett's oesophagus in tertiary referral Centres: a randomised cross-over multicentre study. ISRCTN68328077 DOI 10.1186/ISRCTN68328077. Available from: <http://www.isrctn.com/ISRCTN68328077?q=&filters=recruitmentCountry:Netherlands,conditionCategory:Cancer&sort=&offset=67&totalResults=205&page=1&pageSize=100&searchType=basic-search>
7. Endoscopic Tri­Modal Imaging versus standard video endoscopy for the detection of early neoplasia in patients with low­grade dysplasia in a Barrett's oesophagus a multicentre randomised cross­over controlled study. ISRCTN91816824 DOI 10.1186/ISRCTN91816824. Available from: <http://www.isrctn.com/ISRCTN91816824?q=&filters=conditionCategory:Digestive%20System,trialStatus:Completed,recruitmentCountry:Netherlands&sort=&offset=28&totalResults=67&page=1&pageSize=50&searchType=basic-search>
8. Clinical Trials Registry-India.Possible beneficial role of CT scan combined with virtual endoscopy in cases of esophageal tumor. CTRI/2013/10/004043 [Registered on: 03/10/2013]. Available from: <http://www.ctri.nic.in/Clinicaltrials/pdf_generate.php?trialid=7448&EncHid=&modid=&compid=%27,%277448det%27>
9. Clinical Trials. Evaluation of Efficiency of Esophageal Capsule Endoscopy in the Screening of Patients with Gastroesophageal Reflux Disease or Dyspepsia as Compared to Upper Endoscopy. NCT00217347. Available from: <https://clinicaltrials.gov/ct2/show/NCT00217347>
10. Clinical Trials. Endoscopic Tri-Modal Imagine to Distinguish Functional Dyspepsia From Reflux Disease. NCT02685150. Available from: <https://clinicaltrials.gov/ct2/show/NCT02685150>
11. Clinical Trials. Endoscopic Findings in Patients with Typical Gastroesophageal Reflux Disease (GERD) Symptoms. NCT00730106. Available from <https://clinicaltrials.gov/ct2/show/NCT00730106>
12. Clinical Trials. Barrett's Esophagus & Gastroesophageal Reflux Disease. NCT00513331. Available from: <https://clinicaltrials.gov/ct2/show/NCT00513331>
13. Clinical Trials .Demographics and Findings of Upper Endoscopy Patients. NCT00576992. Available from <https://clinicaltrials.gov/ct2/show/NCT00576992>
14. Analysis of Biopsies From the Upper Gastrointestinal Tract (histoGERD). NCT01576289. Available from <https://clinicaltrials.gov/ct2/show/NCT01576289>
15. Clinical Trials.Study of Endoscopic Barrett's Esophagus Diagnosis. NCT0059146. Available from: <https://clinicaltrials.gov/ct2/show/results/NCT00591461>
16. Clinical Trials. Narrow­band Imaging, Autofluorescence Imaging and Gastroesophageal Reflux Disease. NCT01504971. Available from: <https://www.clinicaltrials.gov/ct2/show/NCT01504971>
17. Clinical Trials. Pilot Study to Image the Esophagus Using a SECM Tethered Endoscopic Capsule (SECM). NCT02445014. Available from: <https://clinicaltrials.gov/ct2/show/NCT02445014>
18. Clinical Trials. Imaging Enhanced Endoscopy for the Screening of Barrett's Esophagus. NCT01976351. Available from <https://clinicaltrials.gov/ct2/show/NCT02445014>
19. Clinical Trials. Cytosponge Adequacy Study Evaluation II (CASEII). NCT02395471. Available from <https://clinicaltrials.gov/ct2/show/NCT02395471>
20. Clinical Trials. The Role of Chromoendoscopy in the Early Detection of Esophageal Cancer in Patients With Prior Head and Neck Cancers. NCT02435602. Available from: <https://clinicaltrials.gov/ct2/show/NCT02435602>
21. Clinical Trials. Esophageal Cytology With FISH in Detecting Esophageal Cancer in Participants Undergoing EsophagoGastro­Duodenoscopy or Upper Endoscopy. NCT02100189 Available from: <https://clinicaltrials.gov/ct2/show/NCT02100189>
22. Clinical Trials. Endoscopy Every 2 Years or Only as Needed in Monitoring Patients With Barrett Esophagus. NCT00987857. Available from: <https://clinicaltrials.gov/ct2/show/NCT00987857>
23. Clinical Trials. Tethered Capsule Endoscope in Screening Participants for Barrett Esophagus. NCT00903136. Available from: <https://clinicaltrials.gov/ct2/show/NCT00903136>
24. Pronase Improves Efficacy of Chromoendoscopy Screening on Esophageal Cancer.NCT02030769. Available from: <https://clinicaltrials.gov/ct2/show/NCT02030769>
25. Gastrointestinal Biomarkers in Tissue and Biological Fluid Samples From Patients and Healthy Participants Undergoing Colonoscopy, Endoscopy, or Surgery. NCT00899626. Available from <https://clinicaltrials.gov/ct2/show/NCT00899626>
26. Early Detection of Esophageal Cancer. NCT00341523. Available from: <https://clinicaltrials.gov/ct2/show/NCT00341523>
27. Endoscopy Screening for Esophageal Cancer. NCT00927446. Available from: <https://clinicaltrials.gov/ct2/show/NCT00927446>
28. UMIN-CTR Clinical Trial Registry. A prospective randomized study to compare the tolerability of the magnifyi ng narrow‒band imaging endoscopy with lugol chromoendoscopy for esophageal cancer screening. UMIN000012097 . Available from: <https://upload.umin.ac.jp/cgi-open-bin/ctr_e/ctr_view.cgi?recptno=R000014135>
29. Jiankun Wang, Lili Zhao, Zhining Fan, Li Liu, Xiang Wang, Min Wang. Diagnostic yield of capsule endoscopy versus conventional gastroscopy for gastric diseases: a systematic review and meta-analysis. PROSPERO International prospective register of systematic reviews.
30. Offman, J., Muldrew, B., O'Donovan, M., Debiram-Beecham, I., Pesola, F., Kaimi, I., Smith, S. G., Wilson, A., Khan, Z., Lao-Sirieix, P., Aigret, B., Walter, F. M., Rubin, G., Morris, S., Jackson, C., Sasieni, P., and Fitzgerald, R. C. Barrett's oESophagus trial 3 (BEST3): Study protocol for a randomised controlled trial comparing the Cytosponge-TFF3 test with usual care to facilitate the diagnosis of oesophageal pre-cancer in primary care patients with chronic acid reflux. BMC Cancer 2018. 18 (1) 784.

### Study design: Abstract (n=230)

1. Tsukanov, V. V., Onuchina, E. V., Vasyutin, A. V., Amelchugova, O. S., and Tonkikh, J. L. H. Pylori eradication does not affect the GERD course in elderly patients: The results of a 5-year prospective study. Gastroenterology 2016; 150 (4 SUPPL. 1): S471-S472.
2. Park, C. and Portenier, D. Reflux, Barrett's esophagus & bariatric surgery: Working towards a clinical pathway for the management of a pre-cancerous condition in bariatric surgery patients. Surgery for Obesity and Related Diseases 2015; 11 (6 SUPPL. 1): S118-.
3. Cai, J. X., Campbell, E. J., and Richter, J. M. Evaluation of discordant upper endoscopy in outpatients with gastroesophageal reflux disorder. Gastroenterology 2015; 148 (4 SUPPL. 1): S210-.
4. Kramer, J. R., Shakhatreh, M. H., Naik, A. D., Duan, Z., and El-Serag, H. B. Use and yield of endoscopy in patients with uncomplicated gastroesophageal reflux disorder. JAMA Internal Medicine 2014; 174 (3): 462-465.
5. Khandwalla, H., Kramer, J. R., Ramsey, D. J., Duong, N. B., Green, L. K., and El-Serag, H. Barrett's esophagus at endoscopy but no intestinal metaplasia on biopsy, what's next?. Gastroenterology 2013; 144 (5 SUPPL. 1): S688-S689.
6. Tang, T. Q., Lee, C. T., Tai, C. M., and Chang, C. Y. Health-related quality of life in patients with Barrett's esophagus. Journal of Gastroenterology and Hepatology (Australia) 2015; 30: 301-302.
7. Tan, G. and Gandhi, M. Outcomes of open access endoscopy in dyspepsia/GERD patients without alarm features in a community medical center. American Journal of Gastroenterology 2015; 110: S641-S642.
8. Liu, D., Lv, J., Zhang, X., Ma, S., Zhang, L., Zou, B., Gong, J., and Zhang, J. Detection of Barrett's esophagus in columnar-lined esophagus by using narrow band imaging with magnifying endoscopy. Gastrointestinal endoscopy 2015; 81 (5 SUPPL. 1): AB392-AB393.
9. Crews, N. R., Dunagan, K. T., Johnson, M. L., Enders, F., Schleck, C. D., Wong Kee Song, L. M., Wang, K. K., Katzka, D. A., and Iyer, P. G. Increased number of risk factors predicts esophageal injury and metaplasia: Results from a large prospective population-based study. Gastroenterology 2015; 148 (4 SUPPL. 1): S215-.
10. Evsyutina, Y., Truhmanov, A., Lyamina, S., Malyshev, I., and Ivashkin, V. The circulating level of cytokines in patients with different forms of gastroesophageal reflux disease: Non-erosive reflux disease, erosive esophagitis and barrett's esophagus. United European Gastroenterology Journal 2014; 2 (1 SUPPL. 1): A263-A264.
11. Russo, E., Cereatti, F., Bruschini, P., Trentino, P., De, Giacomo T., D'Amati, G., Venuta, F., and Francioni, F. Barrett's esophagus and esophageal adenocarcinoma. A single center experience and changes of management. Diseases of the Esophagus 2014; 27: 154A-.
12. Crews, N. R., Dunagan, K. T., Johnson, M. L., Devanna, S., Wong Kee Song, L. M., Katzka, D. A., and Iyer, P. G. Prevalence and characteristics of esophagitis and barrett's esophagus in population subjects without gastroesophageal reflux symptoms: Results from a large randomized controlled study. Gastroenterology 2014; 146 (5 SUPPL. 1): S28-S29.
13. Alashkar, B., Faulx, A. L., Isenberg, G. A., Greer, K. B., Pulice, R., Hepner, A., Falck-Ytter, Y., and Chak, A. Comparative acceptance of transnasal esophagoscopy vs. Esophageal capsule endoscopy for barrett's esophagus screening. Gastroenterology 2013; 144 (5 SUPPL. 1): S689-S690.
14. Egginton, J., Dunagan, K. T., Shah, N. D., Blevins, C., Ragunathan, K., Leggett, C. L., and Iyer, P. G. Patient preferences for endoscopic assessment of gastroesophageal reflux and barrett's esophagus. Gastroenterology 2013; 144 (5 SUPPL. 1): S689-.
15. El-Serag, H., Hashmi, A. A., Garcia, J. M., Richardson, P., Alsarraj, A., Fitzgerald, S. J., Vela, M. F., Shaib, Y. H., Abraham, N., Velez, M. E., Cole, R. A., Rodriguez, M., Anand, B., Graham, D. Y., and Kramer, J. R. Visceral abdominal obesity measured by CT scan is associated with an increased risk of barrett's esophagus: A case-control study. Gastroenterology 2013; 144 (5 SUPPL. 1): S380-.
16. Rubenstein, J. H., Morgenstern, H., McConnell, D. S., Scheiman, J. M., Schoenfeld, P. S., Appelman, H. D., Metko, V., and Inadomi, J. M. Association of serum leptin with barrett's esophagus. Gastroenterology 2013; 144 (5 SUPPL. 1): S351-.
17. Lamzabi, I., Jain, R., and Jakate, S. Helicobacter carditis and its association with symptoms, egd findings and coexistent helicobacter gastritis, helicobacter duodenitis, gastroesophageal reflux and barrett's esophagus. Laboratory Investigation 2013; 93: 163A-.
18. Blevins, C., Sharma, A., Johnson, M., Dunagan, K., Gupta, M., and Iyer, P. Influence of reflux and central adiposity on intercellular space in squamous esophageal epithelium acg obesity award. American Journal of Gastroenterology 2013; 108: S7-.
19. Leggett, C., Gorospe, E. C., Dunagan, K. T., Katzka, D. A., Clemens, M. A., and Prasad, G. A. Influence of central obesity on esophageal injury: A population based study. Gastroenterology 2012; 142 (5 SUPPL. 1): S759-.
20. Wu, Y., Tseng, P., Wang, S., and Yang, W. 18Fluoro-deoxyglucose positron emission tomography in gastroesophageal reflux disease. European Journal of Nuclear Medicine and Molecular Imaging 2012; 39: S236-.
21. Neumann, H., GuNther, C., Vieth, M., and Neurath, M. F. 3-dimensional endoscopy (3De) improves the characterization of non-dysplastic and dysplastic barrett's esophagus. Gastrointestinal endoscopy 2012; 75 (4 SUPPL. 1): AB481-.
22. Chang, C.-Y., Lee, C.-T., Lo, J., Hwang, J.-C., Ou, T., and Lin, J.-T. Health-related quality of life in Barrett's esophagus. Journal of gastroenterology and hepatology 2011; 26: 212-.
23. Sugimoto, M., Nishino, M., Kodaira, C., Yamade, M., Ikuma, M., and Furuta, T. Esophageal mucosal injury with low-dose aspirin and its prevention by rabeprazole. Gastroenterology 2009; 136 (5 SUPPL. 1): A447-.
24. Sutheesuntorntham, B., Leelakusolvong, S., and Suthiwana, C. Quality of life in non-erosive reflux disease (NERD) before and after treatment with low dose and standard dose esomeprazole in thai patient. Digestion 2009; 79 (3 SUPPL. 1): 69-.
25. Ende, A. R., Higa, J. T., Singla, A., Choi, A. Y., Lee, A. B., Whang, S. G., Gravelle, K., D'Andrea, S., Bang, S. J., Schmidt, R. A., Yeh, M. M., and Hwang, J. H. Gastric intestinal metaplasia, dysplasia, and gastric cancer in a U.S. tertiary care population-who's at risk?. Gastrointestinal endoscopy 2016; 83 (5 SUPPL. 1): AB459-.
26. Cook, M. B., Drahos, J., Wood, S., Enewold, L., Parsons, R., Freedman, N. D., Taylor, P. R., Ricker, W., and Abnet, C. C. Pathogenesis and progression of esophageal adenocarcinoma by prior diagnosis of Barrett's esophagus. Cancer Research 2015; 75 (15 SUPPL. 1): no-.
27. Li, X., Chen, Y., Chandler, J., Girman, C. J., and Sturmer, T. Similarities regarding diagnostic workup utilization in two data systems. Pharmacoepidemiology and Drug Safety 2015; 24: 351-.
28. Kryvy, V., Klyarytska, I., Semenichina, E., Rabotyagova, Y., Ratan, G., and Tsapyak, T. 13C-octanoic acid breath test in diagnosis of gastroesophageal reflux disease in obese patients with type 2 diabetes mellitus. Neurogastroenterology and Motility 2015; 27: 77-78.
29. Genco, A., Soricelli, E., Maselli, R., Casella, G., Cipriano, M., Baglio, G., Leone, G., Basso, N., and Redler, A. Barrett's esophagus after sleeve gastrectomy for morbid obesity: Preliminary results. Gastrointestinal endoscopy 2015; 81 (5 SUPPL. 1): AB470-.
30. Yamamoto, S., Komori, M., Nishiyama, M., Fukuoka, M., Kudo, S., Maesaka, K., Shirai, K., Kimura, S.-Y., Okahara, T., Okuda, Y., Konoshita, M., Hirao, M., Hosui, A., and Yoshihara, H. Features of upper gastrointestinal abnormalities in non-alcoholic steatohepatitis (NASH) patients. United European Gastroenterology Journal 2014; 2 (1 SUPPL. 1): A450-.
31. Thomas, S. and Corley, D. Associations between leptin, ghrelin, and barrett's esophagus: A case-control study with stratified analyses. American Journal of Gastroenterology 2014; 109: S5-.
32. Genco, A., Maselli, R., Scucchi, L., Casella, G., Cipriano, M., Leone, G., Lorenzo, M., Baglio, G., Basso, N., and Redler, A. Barrett esophagus: A possible long-term complication after sleeve gastrectomy. Obesity Surgery 2014; 24 (8): 1145-1146.
33. Nguyen, T., Khalaf, N., Ramsey, D. J., and El-Serag, H. Statin use may decrease the risk of barrett's esophagus: A case-control study of U.S. Veterans. Gastroenterology 2014; 146 (5 SUPPL. 1): S-561.
34. Cui, R., Zhang, H., Lu, J., Xue, Y., Wang, Y., and Zhou, L. Diagnostic value of combination of intercellular space measurement and histopathologic score for gastroesophageal reflux disease. Gastroenterology 2014; 146 (5 SUPPL. 1): S-116.
35. Cheung, D., Menon, S., and Trudgill, N. How commonly is oesophageal cancer missed at endoscopy (a UK primary care based study)? Gut 2013; 62: A5-.
36. Rubenstein, J. H., Morgenstern, H., Appelman, H. D., Scheiman, J. M., Schoenfeld, P. S., McMahon, L. F., Metko, V., Near, E., Kellenberg, J. E., Kalish, T., and Inadomi, J. M. Risk factors for barrett's esophagus among men without gerd symptoms: Results from the newly diagnosed barrett's esophagus study. Gastroenterology 2012; 142 (5 SUPPL. 1): S72-.
37. Walia, R., Hodges, T. N., Huang, J. L., Varsch, K. A., Saggar, R., Naik, P. M., Kuo, E. Y., Bremner, R. M., and Smith, M. A. Incidence and clinical course of barrett's esophagus pre-and post-lung transplantation. Journal of Heart and Lung Transplantation 2012; 31 (4 SUPPL. 1): S180-S181.
38. Kumar, U., Cappell, M., Desai, T., and Batke, M. Barrett's esophagus in eosinophilic esophagitis, a coincidence or correlation? - A retrospective case-control study of 365 patients. American Journal of Gastroenterology 2011; 106: S8-.
39. Trapasso, R. and Genta, R. Helicobacter pylori gastritis is inversely correlated to dysplasia in patients with Barrett's esophagus ACG governors award for excellence in clinical research. American Journal of Gastroenterology 2011; 106: S1-S2.
40. Pacheco, Bastidas F., Alvarez, Uslar R., Molina, Zapata H., Alarcon, Mendez A., and Valdebenito, Quiroz M. Erosive esophagitis after laparoscopic sleeve gastrectomy. Obesity Surgery 2011; 21 (8): 1007-.
41. Cadman, L., Nelsen, E. M., Tian, J., Schleck, C., Zinsmeister, A. R., Locke, G. R., Talley, N. J., Wang, K. K., Dunagan, K. T., and Prasad, G. A. Metabolic syndrome is a risk factor for Barrett's esophagus : A population based case control study. Gastroenterology 2011; 140 (5 SUPPL. 1): S178-.
42. Tabbaa, M. G., Fernandez, R., and Muala, S. Acute upper gastro-intertinal bleeding (AUGIB) is a serious complication of GERD with different clinical features from bleeding peptic ulcers (PUD). Gastroenterology 2009; 136 (5 SUPPL. 1): A738-A739.
43. Singh, M., Wani, S. B., Rastogi, A., Hall, S. B., Higbee, A. D., Singh, V., Bansal, A., and Sharma, P. Is proton pump inhibitor (PPI) use associated with a lower prevalence of Barrett's esophagus (BE) in patients with chronic GERD. Gastroenterology 2009; 136 (5 SUPPL. 1): A595-.
44. Lord, R. V. N., Freeman, A., Botelho, N. K., Wettstein, A. R., El-Hammuri, N., Levert-Mignon, A., and Lord, S. J. Effect of barryx halo radiofrequency ablation treatment on mRNA gene expression in Barrett's esophagus with dysplasia. Gastroenterology 2009; 136 (5 SUPPL. 1): A593-.
45. Entezari, O., Nouri, Z., Dowlati, E., Scott, V. F., Frederick, W., Lee, E. L., Smoot, D. T., Hardy, A., Ibrahim, A. Z. A., and Ashktorab, H. Helicobacter pylori may protect African Americans from reflux esophagitis a hospital based study. Gastroenterology 2009; 136 (5 SUPPL. 1): A552-
46. Chen, M., Snyder, C., Hatcher, L., Lynch, H. T., Watson, P., and Gatalica, Z. Barrett's esophagus in the patients with familial adenomatous polyposis. Laboratory Investigation 2009; 89: 126A-.
47. Menon, S., Nightingale, P., Butterworth, J., and Trudgill, N. Severe oesophagitis and its complications are more common in women after the menopause. Gut 2009; 58: A145-.
48. Rajendra, S., Snow, E. T., Lai, H. J., Ball, M. J., and Robertson, I. K. Human papilloma virus and Barrett's oesophagus. Journal of gastroenterology and hepatology 2009; 24: A338-.
49. Carton, Mulligan, Keeling, Tanner, McDonald, and Reynolds Specialized intestinal metaplasia: analysis of prevalence, risk factors and association with gastro-oesophageal reflux disease. The British journal of surgery 2000; 87 (3): 362-373.
50. Felsenreich, D. M., Langer, F., Kefurt, R., Panhofer, P., Schermann, M., Beckerhinn, P., Sperker, C., Lenglinger, J., Kristo, I., Schoppmann, S., and Prager, G. Weight loss, reflux and reoperations: Our first 100 patients treated with lap. Sleeve Gastrectomy. Obesity Facts 2016; 9: 311-312.
51. Heberle, C. R., Kong, C. Y., and Hur, C. Cost effectiveness of a minimally invasive cell sampling device to screen for Barrett's esophagus in patients with Gerd symptoms. Value in Health 2016; 19 (3): A304-.
52. Parasa, S., Vennelaganti, S., Vennalaganti, P., Brown, J., Kanakadandi, V., Alsop, B., Titi, M. A., Kennedy, K., Kohli, K., Gachpaz, B., Vittal, A., Duvvuri, A., Bansal, A., Gupta, N., and Sharma, P. Epidemiology and risk factors for the presence of large hiatal hernias in GERD patients. Gastrointestinal endoscopy 2016; 83 (5 SUPPL. 1): AB588-.
53. Kanakadandi, V., Vennelaganti, S., Vennalaganti, P., Brown, J., Parasa, S., Alsop, B., Titi, M. A., Bansal, A., Kennedy, K., Kohli, K., Duvvuri, A., Gachpaz, B., Vittal, A., Gupta, N., and Sharma, P. Declining trends in the prevalence of barrett's esophagus among patients with gastroesophageal reflux disease (GERD). Gastrointestinal endoscopy 2016; 83 (5 SUPPL. 1): AB553-.
54. Dova, G., Caro, L. E., Brasesco, O., Paleari, J., Borlle, G. L., Durand, L., Bauer, I., Gajardo, C., Bolino, C., Dumonceau, J. M., and Cerisoli, C. L. Effects of gastric bypass in obese patients with barrett's esophagus. Gastrointestinal endoscopy 2016; 83 (5 SUPPL. 1): AB551-.
55. Preedy, R., El, Menabawey T., Phillpotts, S., and Besherdas, K. Is there any value of performing an endoscopy in GERD patients under 50? Gastrointestinal endoscopy 2016; 83 (5 SUPPL. 1): AB546-.
56. Solomon, A. and Besherdas, K. Is there any value of endoscopy for gastro-oesophageal reflux disease in patients under 45?. Gastrointestinal endoscopy 2016; 83 (5 SUPPL. 1): AB533-.
57. Daoud, D., Therrien, A., Soucy, G., and Bouin, M. Is the lymphocytic esophagitis a new clinical entity? Canadian Journal of Gastroenterology and Hepatology 2016.
58. Milne, K., Kathol, B., Swain, M., Johnstone, C., Kwan, J., Schoombee, W., Andrews, C., and Novak, K. Endoscopy utilization and outcome for the GI nurse navigator pathway: A quality improvement project for chronic dyspepsia, heartburn & irritable bowel syndrome. Canadian Journal of Gastroenterology and Hepatology 2016.
59. Chen, W-C., Hemminger, L. L., Bowers, S. P., and Wolfsen, H. C. Development of Barrett's esophagus after esophagectomy: Experience at a single tertiary center. Gastroenterology 2016; 150 (4 SUPPL. 1): S1225-.
60. Zackria, S., Thota, P. N., and Lopez, R. Prevalence of abnormal endoscopic findings in patients with laryngopharyngeal reflux (LPR). Gastroenterology 2016; 150 (4 SUPPL. 1): S861-.
61. Rubenstein, J. H., McConnell, D., Beer, D. G., Chak, A., Metko, V., and Clines, G. Association of proton pump inhibitor use with hyperparathyroidism. Gastroenterology 2016; 150 (4 SUPPL. 1): S835-.
62. Wright, M. R., Higginbotham, T., Slaughter, J. C., Ates, F., Yuksel, E. S., and Vaezi, M. Mucosal impedance in Barrett's Esophagus: Can it assess compliance with medication?. Gastroenterology 2016; 150 (4 SUPPL. 1): S260-.
63. Fock, K. M., Ang, T. L., Li, W. J., Poh, C. H., Law, N. M., and Ang, D. Refractory gastro-esophageal reflux symptoms in a multiracial Asian cohort: Roles of advanced imaging and functional testing in diagnosis. Gastroenterology 2016; 150 (4 SUPPL. 1): S35-.
64. Schlottmann, F., Reino, R., Spano, M., Galvarini, M., Gallesio, J. A., and Buxhoeveden, R. Preoperative endoscopy in bariatric patients may change surgical strategy. Surgical Endoscopy and Other Interventional Techniques 2016; 30: S261-.
65. Boys, J. A., Azadgoli, B., Martinez, M., Oh, D. S., Hagen, J. A., and DeMeester, S. R. Is EGD reporting adequate: A review of reports from 100 referring gastroenterologists. Surgical Endoscopy and Other Interventional Techniques 2016; 30: S214-.
66. Maddalo, G., Marafatto, F., Fassan, M., Cardin, R., Piciocchi, M., Pozzan, C., Zaninotto, G., Ruol, A., Castoro, C., Rugge, M., and Farinati, F. Serum determination of squamous cellular carcinoma antigen as a biomarker of Barrett-s esophagus and esophageal cancer: A phase iii study. Digestive and Liver Disease 2016; 48: e70-e71.
67. Onuchina, E., Tsukanov, V., Kasparov, E., Vasyutin, A., and Amelchugova, O. Monitoring of Barrett's esophagus in elderly patients: Results of five-year prospective study. Journal of Gastroenterology and Hepatology (Australia) 2015; 30: 58-59.
68. James, L. W., Kwongming, F., Tiingleong, A., Choohean, P., Ngai, Moh L., and Daphne, A. Refractory gastro-esophageal reflux disease in an Asian population: Roles of advanced imaging and functional testing in diagnosis and management. Journal of Gastroenterology and Hepatology (Australia) 2015; 30: 7-.
69. Horsley-Silva, J. L., Amer, S., Kommineni, V. T., Crowell, M. D., Lam-Himlin, D., and Nguyen, C. C. Gastric intestinal metaplasia: Defining the natural history. American Journal of Gastroenterology 2015; 110: S1021-S1022.
70. Higa, J. T., Ende, A. R., Choi, A. Y., Bang, S. J., Gravelle, K., Kang, S., Whang, S. G., Lee, A. B., D'Andrea, S., Schmidt, R. A., Yeh, M. M., and Hwang, J. H. Gastric intestinal metaplasia in a U.S. Tertiary care population. American Journal of Gastroenterology 2015; 110: S1016-.
71. Villa, N. A., Lam-Himlin, D., Pannala, R., Fleischer, D. E., Ramirez, F. C., and Faigel, D. Pancreatic acinar metaplasia at the gastroesophageal junction: A single institution experience. American Journal of Gastroenterology 2015; 110: S735-.
72. Tabbaa, M., Dizechi, S., and Newman, A. Nsaid use in patients with barrett's esophagus increased the severity of reflux esophagitis. American Journal of Gastroenterology 2015; 110: S724-.
73. Tang, Z., Akinyeye, S., Suarez, M. G., Berzosa, M., Ilyas, J., Zarrin-Khameh, N., and Vela, M. F. Yield of upper endoscopy and impedance-PH monitoring in PPI-refractory GERD patients with typical symptoms. American Journal of Gastroenterology 2015; 110: S699-.
74. Anapaz, V., Carvalho, R., Reis, J., Branco, J., Lourenco, L., Santos, L., Cardoso, M., and Oliveira, A. Impact of endoscopic surveillance in Barrett 'S esophagus without dysplasia-a retrospective study. United European Gastroenterology Journal 2015; 3 (5 SUPPL. 1): A657-A658.
75. Katopodi, K., Viazis, N., Karamanolis, G., Anastasiou, J., Denaxas, K., Giakoumis, M., Koukouratos, T., Kamberoglou, D., Christidou, A., Ladas, S. D., and Karamanolis, D. G. Proton pump inhibitor and selective serotonin reuptake inhibitor therapy for the management of non cardiac chest pain. United European Gastroenterology Journal 2015; 3 (5 SUPPL. 1): A476-A477.
76. Solomon, A. S. and Besherdas, K. Is there any value of endoscopy for gord in patients under 45? United European Gastroenterology Journal 2015; 3 (5 SUPPL. 1): A361-.
77. Evsyutina, Y., Trukhmanov, A., Storonova, O., Ivashkin, V., Lyamina, S., and Malyshev, I. Association between the form of gastroesophageal reflux disease, caracteristics of esophageal pH-impedance monitoring and cytokines expression. United European Gastroenterology Journal 2015; 3 (5 SUPPL. 1): A293-.
78. Sang, Kil L. and Lee, S. K. Sedation during upper endoscopy can decrease the detection of minimal change esophagitis and hiatal hernia. Neurogastroenterology and Motility 2015; 27: 97-.
79. Ding, Y., Cavichiolo, F., Ponnuswamy, S., Moattar, H., Alghamry, A., Croese, J., Hodgson, R., Appleyard, M., Howlett, M., Vandeleur, A., and Rahman, T. Service and quality improvement-Eosinophilic oesophagitis and food bolus impaction-an emergent emergency? Journal of Gastroenterology and Hepatology (Australia) 2015; 30: 179-180.
80. Wark, G., Turner, I., and Al-Sohaily, S. What is the utility of performing gastroscopy in patients with positive FOBT without upper GI symptoms, iron deficiency or anaemia? Journal of Gastroenterology and Hepatology (Australia) 2015; 30: 24-.
81. Kommineni, V. T., Pannala, R., Crowell, M. D., Burdick, G. E., Vela, M. F., Faigel, D. O., Fleischer, D. E., and Ramirez, F. C. Outcomes of endoscopic therapy for high-grade dysplasia(HGD) and esophageal adenocarcinoma(EAC) in long and short segment Barrett's esophagus. Gastrointestinal endoscopy 2015; 81 (5 SUPPL. 1): AB513-AB514.
82. Subramaniam, S., Goodchild, G., and Besherdas, K. The value of follow up endoscopy in oesophagitis patients: A UK district general hospital experience. Gastrointestinal endoscopy 2015; 81 (5 SUPPL. 1): AB237-.
83. Chang, C.-Y., Lee, C.-T., Tai, C.-M., and Lin, J.-T. Lower dysplastic progression of Barrett's Esophagus in asian population- an ethnic chinese prospective cohort study. Gastrointestinal endoscopy 2015; 81 (5 SUPPL. 1): AB500-.
84. Bibbo, S., Ianiro, G., Petruzziello, L., Spada, C., Larghi, A., Riccioni, M. E., Gasbarrini, A., Costamagna, G., and Cammarota, G. Esophageal posterior and right wall are the most common localizations of Barrett's esophagus. Gastrointestinal endoscopy 2015; 81 (5 SUPPL. 1): AB499-.
85. Bernal-Mendez, A. R., Tellez-Avila, F. I., Briseno, F. D., Romano, A. F., Barreto-Zuniga, R., Martinez-Lozano, J. A., Ramierz Polo, A. I., and Valdovinos-Andraca, F. Clinical and endoscopic differences between patients with barrett's esophagus without dysplasia and dysplasia/esophageal adenocarcinoma in Latin American population. Gastroenterology 2015; 148 (4 SUPPL. 1): S348-S349.
86. Rebecchi, F., Allaix, M., Giaccone, C., Merlo, P., and Morino, M. Laparoscopic roux-en-y-gastric bypass increases esophageal exposure to weakly acidic reflux. Surgical Endoscopy and Other Interventional Techniques 2015; 29: S50-.
87. Subramaniam, S., Goodchild, G., and Besherdas, K. Odynophagia and its yield on upper GI endoscopy-a symptom worth asking about. United European Gastroenterology Journal 2014; 2 (1 SUPPL. 1): A479-.
88. Subramaniam, S., Goodchild, G., and Besherdas, K. Limited value for follow up endoscopy in severe oesophagitis: Findings from a large london district general hospital. United European Gastroenterology Journal 2014; 2 (1 SUPPL. 1): A479-.
89. Robles-Medranda, C., Del, Valle R., Soria, M., Bravo, G., Lukashok, H., and Robles-Jara, C. Pentax i-scantm with magnification for the identification of underdiagnosis organic esophageal lesions (barret esophagus and esophagitis) in patients with functional dyspepsia: A prospective study. United European Gastroenterology Journal 2014; 2 (1 SUPPL. 1): A163-A164.
90. Gaikwad, N., Samarth, S. G. A., and Sawalakhe, N. Endoscopic profile of dysphagia-A prospective study. Indian Journal of Gastroenterology 2014; 33 (1 SUPPL. 1): A30-.
91. Gross, S., Kaul, V., and Smith, M. Increased detection of barrett's esophagus and dysplasia in community gastroenterology practices resulting from the addition of computer-assisted transepithelial brush biopsy to forceps biopsy. American Journal of Gastroenterology 2014; 109: S39-.
92. Alkaddour, A., McGaw, C., Hritani, R., Palacio, C., Munoz, J., and Vega, K. Clinical factors do not explain why barrett's esophagus primarily occurs among non-hispanic whites in North Florida. American Journal of Gastroenterology 2014; 109: S34-.
93. Toppino, M., Rebecchi, F., Allaix, M. E., Giaccone, C., Merlo, P., and Morino, M. Increased esophageal exposure to weakly acidic reflux after laparoscopic Roux-en-Y-gastric bypass for morbid obesity. Obesity Surgery 2014; 24 (7): 976-.
94. Sadek, R. W. and Wassef, A. M. Viabliltiy of simultaneous diaphragmatic hernia repair and longitudinal sleeve gastrectomy paired procedures. Obesity Surgery 2014; 24 (8): 1349-.
95. Cayado-Lopez, R., Bornschein, J., Zeki, S., Udarbe, M., and Di, Pietro M. Clinical utility of Endofaster in patients on chronic ppi therapy undergoing upper GI endoscopy. Gut 2014; 63: A117-.
96. Subramaniam, S., Goodchild, G., and Besherdas, K. Odynophagia - A symptom worth asking about? Gut 2014; 63: A60-A61.
97. Butt, S. K. and Besherdas, K. The value of gastroscopy in the investigation of non cardiac chest pain. Gut 2014; 63: A60-.
98. Buda, A., De, Bona M., Bellumat, A., Valiante, F., Piselli, P., Della, Libera D., Cian, E., Sturniolo, G. C., and De, Boni M. Predictors of barrett oesophagus in patients undergoing first time gastroscopy in a single centre: Potential implications for the screening dilemma. Digestive and Liver Disease 2014; 46: S90-.
99. DelRosso, L. M., Hoque, R., and Harper, M. Prevalence of obstructive sleep apnea and Barrett's esophagus in patients referred for esophagogastroduodenoscopy due to reflux symptoms. Sleep 2014; 37: A261-A262.
100. Chaudhry, U. I., Mikami, D. J., Needleman, B. J., Melvin, W. S., and Noria, S. F. Routine preoperative esophagogastroduodenoscopy in patients undergoing bariatric surgery. Surgical Endoscopy and Other Interventional Techniques 2014; 28: 398-.
101. Sormaz, I. C., Tunca, F., Iscan, Y. A., Meric, S., and Avtan, L. Laparoscopic nissen-rossetti fundoplication in gastro-oesophageal reflux disease; experience in 205 consecutive patients. Surgical Endoscopy and Other Interventional Techniques 2014; 28: S157-.
102. Bradley, D. D., Louie, B. E., Aye, R. W., McMahon, R., Chen, J., and Farivar, A. S. The effect of concurrent esophageal pathology on bariatric surgical planning. Gastroenterology 2014; 146 (5 SUPPL. 1): S-1026.
103. Patel, D. A., Ates, F., Slaughter, J. C., Higginbotham, T., and Vaezi, M. F. Esophageal acid exposure consistency as a measure of pH reliability: Should we abandon lower normative cutoffs? Gastroenterology 2014; 146 (5 SUPPL. 1): S751-S752.
104. Aberra, F., Ates, F., Li, Z., Slaughter, J. C., Higginbotham, T., and Vaezi, M. F. Appropriateness of upper endoscopy referrals in patients with GERD by specialty. Gastroenterology 2014; 146 (5 SUPPL. 1): S749-S750.
105. Onuchina, E. V., Amelchugova, O. S., and Tsukanov, V. V. The frequency of high-grade dysplasia in elderly patients with barrett's esophagus: Results of five-year prospective study. Gastroenterology 2014; 146 (5 SUPPL. 1): S306-S307.
106. Al, Kaddour A., McGaw, C., Hritani, R., Palacio, C., Munoz, J. C., and Vega, K. J. African Americans do not develop barrett's esophagus following erosive esophagitis. Gastroenterology 2014; 146 (5 SUPPL. 1): S302-S303.
107. Zaidi, A. H., Gopalakrishnan, V., Kasi, P. M., Malhotra, U., Balasubramanian, J., Visweswaran, S., Zeng, X., Sun, M., Bergman, J. J., Bigbee, W. L., and Jobe, B. A. Evaluation of a four-protein biomarker panel (biglycan, annexin-A6, myeloperoxidase and protein S100-A9; B-AMP©) for detection of esophageal adenocarcinoma. Gastroenterology 2014; 146 (5 SUPPL. 1): S-161.
108. Ketwaroo, G. A., Vivian, C., Makda, M., Magge, S., Feuerstein, J., Patil, D. T., Najarian, R. M., and Lembo, A. Long-term follow-up and incidence of HPV in the esophageal squamous papilloma. Gastrointestinal endoscopy 2014; 79 (5 SUPPL. 1): AB393-.
109. Bolino, C., Cerisoli, C. L., Durand, L., Avagnina, A., Elsner, B., De, Elizalde S., Domenichini, E., and Caro, L. E. Long term follow up in patients with columnar lined esophagus: A retrospective analysis of 12 years. Gastrointestinal endoscopy 2014; 79 (5 SUPPL. 1): AB387-AB388.
110. Saxena, A., Parikh, K., Lu, C., Chak, A., Greer, K. B., Cooper, G. S., Alashkar, B., and Faulx, A. L. Late stage diagnosis of esophageal adenocarcinoma (EAC) in veterans-could screening be effective?. Gastrointestinal endoscopy 2014; 79 (5 SUPPL. 1): AB385-.
111. Schneider, N., Plieschnegger, W., Schmack, B., Bordel, H., Hofler, B., Eherer, A., Wolf, E.-M., Rehak, P., Vieth, M., and Langner, C. Cardiac mucosa at the gastroesophageal junction: Indicator of gastroesophageal reflux disease? United European Gastroenterology Journal 2013; 1 (1 SUPPL. 1): A568-.
112. Lahcene, M., Oumnia, N., Chiali, N., Saadaoui, Y., Boudjella, M., and Tebaibia, A. Characteristics of esophageal involvement in scleroderma. United European Gastroenterology Journal 2013; 1 (1 SUPPL. 1): A256-.
113. Mukundan, S., Muthukumaran, K., Ramkumar, G., Ganesh, R. B., and Kumar, S. J. Methylene blue chromoendoscopy for early diagnosis of Barrett's metaplasia, dysplasia and early esophageal adenocarcinoma: A pilot study. Indian Journal of Gastroenterology 2013; 32 (1 SUPPL. 1): A10-.
114. Sugumaran, A. and Rasheed, A. Gastro-oesophageal reflux disease (GORD) symptomatology is not a reliable predictor of oesophageal adenocarcinoma. Gut 2013; 62: A194-.
115. Liu, X., Wong, A., Kadri, S. R., O'Donovan, M., Lao-Sirieix, P., Burnham, R., and Fitzgerald, R. Gord symptoms and demographic factors as a pre-screening tool for barrett's oesophagus. Gut 2013; 62: A119-.
116. Timratana, P., Lada, M. J., Nieman, D. R., Han, M. S., Peyre, C. G., Jones, C. E., Watson, T. J., and Peters, J. H. The clinical spectrum of esophagogastric junction outflow obstruction identified via high resolution manometry. Gastroenterology 2013; 144 (5 SUPPL. 1): S1099-.
117. Onuchina, E. V., Tsukanov, V. V., and Amelchugova, O. S. Changing of gerd structure in elderly patients: Results of five-year prospective study. Gastroenterology 2013; 144 (5 SUPPL. 1): S861-.
118. Kanakadandi, V., Giacchino, M., Gaddam, S., Bansal, A., Rastogi, A., Higbee, A. D., Gupta, N., and Sharma, P. Has there been a change in the prevalence of be or patient characteristics that may be contributing to the increasing EAC incidence?. Gastroenterology 2013; 144 (5 SUPPL. 1): S690-.
119. Bansal, A., Hong, X., Lee, I.-H., House, J., Mathur, S. C., Rastogi, A., Sharma, P., and Christenson, L. K. Serum exosomal microrna expression can be a novel non-invasive strategy for the screening of barrett's esophagus. Gastroenterology 2013; 144 (5 SUPPL. 1): S684-.
120. Bansal, A., Hong, X., Lee, I.-H., House, J., Mathur, S. C., Rastogi, A., Sharma, P., and Christenson, L. K. Evaluation of tissue microrna expression could increase the accuracy of non-endoscopic esophageal sampling devices based testing for the diagnosis of barrett's esophagus. Gastroenterology 2013; 144 (5 SUPPL. 1): S674-S675.
121. Taggart, M. W., Ross, W. A., Rashid, A., and Abraham, S. Esophageal squamous dysplasia-clinical associations and follow-up. Gastroenterology 2013; 144 (5 SUPPL. 1): S517-.
122. Tsuji, N., Okumura, N., Taniike, S., Takaba, T., Matsumoto, N., Kono, M., Maruyama, Y., and Kudo, M. Verrucous antral gastritis is not related to H. Pylori-positive chronic gastritis, but is related to a high BMI and barrett's esophagus. Gastroenterology 2013; 144 (5 SUPPL. 1): S340-.
123. Rubenstein, J. H., Morgenstern, H., McConnell, D. S., Scheiman, J. M., Schoenfeld, P. S., Appelman, H. D., Metko, V., and Inadomi, J. M. Associations of hyperinsulinemia and diabetes mellitus with barrett's esophagus. Gastroenterology 2013; 144 (5 SUPPL. 1): S28-S29.
124. Gemignani, L., Savarino, E., Corbo, M., Dulbecco, P., Giacchino, M., Giambruno, E., Mastracci, L., Sarocchi, F., Fiocca, R., Repici, A., and Savarino, V. The diagnostic value of narrow-band imagingwith magnifying endoscopy in the detection of gastric intestinal metaplasia: A prospective study in an unselected population. Digestive and Liver Disease 2013; 45: S206-.
125. Bhattacharya, B. and Samaha, S. Granular cell tumors of the gastrointestinal tract: Clinicopathologic associations in a large series. Laboratory Investigation 2013; 93: 145A-.
126. Gashi, Z., Shabani, R., and Haziri, A. The histopathological correlation with clinical and endoscopic evidence in patients with Barrett's esophagus. Journal of Clinical Gastroenterology 2013; 47 (1): 93-.
127. Gonsalkorala, E. S., Roche, E., and Fairley, S. The incidence of oesophageal heterotopic gastric mucosa (O-HGM) in an Australian population. Journal of gastroenterology and hepatology 2013; 28: 40-.
128. Gupta, M., Sharma, A., Buttar, N., Geno, D., Katzka, D., Harmsen, W., Enders, F., Dunagan, K., and Iyer, P. Influence of central obesity and reflux on esophageal injury: A prospective study. American Journal of Gastroenterology 2013; 108: S23-.
129. Al-Subee, O., Al-Khawam, A., Allababidi, R., and Subei, I. Prevalence of barrett's esophagus in a community hospital in Saudi Arabia. American Journal of Gastroenterology 2013; 108: S5-.
130. Satarasinghe, R., Rathnayake, J., Ambawatte, S., Jayasinghe, N., Wijesinghe, R., De, Silva P., and Rasendran, N. Endoscopic outcome in a cohort of adult Sri Lankan dysphagics admitted to a tertiary care hospital. Journal of gastroenterology and hepatology 2013; 28: 722-.
131. Wang, Z. G., Hu, Z. W., Wu, J. M., Liu, J. J., Tian, S. R., Ji, F., and Li, Z. T. The interventional treatment on extra-esophageal symptoms: Preliminary experience on 2016 patients. Journal of gastroenterology and hepatology 2013; 28: 305-.
132. Radha, M., Kini, R., Mohammed, K. S., Kumar, K. P., Pugazhendhi, T., and Ali, M. Clinical significance and differential diagnosis of nodular lesions in the gastrointestinal tract by clinicopathological correlation in a tertiary care hospital in Southern India. Indian Journal of Gastroenterology 2012; 31 (1 SUPPL. 1): A109-.
133. Kumar, G. R., Kini, R., Kani, M. S., Kumar, P., Pugazhendi, T., and Ali, M. Barrett's esophagus-changing trends in incidence-a cross sectional study. Indian Journal of Gastroenterology 2012; 31 (1 SUPPL. 1): A16-A17.
134. Martin, L., Stavrou, M., El, Madani F., and Gupta, S. Six years of laparoscopic Nissen's fundoplication, was it worth it? An audit of 100 patients. Gut 2012; 61: A265-.
135. Hoppo, T., Komatsu, Y., and Jobe, B. A. Short-term outcome of antireflux surgery on patients with chronic cough and abnormal proximal exposure as measured by hypopharyngeal multichannel intraluminal impedance. Gastroenterology 2012; 142 (5 SUPPL. 1): S1075-.
136. Alsop, B. R., Gupta, N., Balasubramanian, G., Wani, S., Gaddam, S., Higbee, A. D., Shipe, T., Singh, M., Yandrapu, H., Giacchino, M., Rastogi, A., Bansal, A., and Sharma, P. Racial differences in prevalence of hiatal hernia and esophageal mucosal injury: Results from a large prospective GERD cohort. Gastroenterology 2012; 142 (5 SUPPL. 1): S759-.
137. Rubenstein, J. H., Morgenstern, H., Chey, W. D., Murray, J., Scheiman, J. M., Schoenfeld, P. S., Appelman, H. D., McMahon, L. F., Metko, V., Near, E., and Inadomi, J. M. Acid reflux, erosive esophagitis, and barrett's esophagus are associated with different measures of abdominal obesity in men. Gastroenterology 2012; 142 (5 SUPPL. 1): S754-.
138. Fasanella, K. E., Bista, R. K., Staton, K. D., Brand, R., McGrath, K., and Liu, Y. Assessment of gastric cardia to monitor the response to endoscopic therapy in patients with esophageal dysplasia and adenocarcinoma. Gastroenterology 2012; 142 (5 SUPPL. 1): S749-S750.
139. El-Serag, H., Hinojosa-Lindsey, M., Duan, Z., Hou, J., Naik, A. D., Street, R. L., Chen, G. J., and Kramer, J. R. Timing and diagnostic yield of endoscopy among patients with gerd. Gastroenterology 2012; 142 (5 SUPPL. 1): S576-S577.
140. Morales-Fuentes, G. A., Barojas, P. F., Antonio-Manrique, M., Clara Luz, M. G., Zamorano, Y., Zarate-Osorno, A., Quinonez-Urrego, E. E., Mejia-Cuan, L. A., Martinez-Camacho, C., Rivera-Nava, C. A., Sanchez-Chavez, X., Rojas-Macuil, P., Montenegro-Molina, W., al Osuna, S. E., and Ramirez-Ramirez, M. A. P53 expression in gastric mucosa of patients infected with helicobacter pylori. Gastroenterology 2012; 142 (5 SUPPL. 1): S476-.
141. Kidambi, T., Toto, E., Ho, N., Taft, T., and Hirano, I. Etiologies of dysphagia over the past decade-emergence of eosinophilic esophagitis. Gastroenterology 2012; 142 (5 SUPPL. 1): S430-S431.
142. Asaoka, D., Nagahara, A., Sasaki, H., Hojo, M., and Watanabe, S. The clinical characteristics of laryngopharyngeal reflux disease by using reflux finding score. Journal of gastroenterology and hepatology 2012; 27: 321-.
143. Lawrence, Ho K. Y. Barrett's esophagus: Situation in Asia-Pacific. Journal of gastroenterology and hepatology 2012; 27: 26-27.
144. Angeles, P.-A., Rosales Zabal, J. M., Lopez Vega, M. C., Fernandez, Cano F., Romero Ordonez, M. A., Gonzalez, Barcenas M., and Rivas, Ruiz F. Impact of the manometric esophageal alterations and the exposure to acid in the incidence of Barrett's Esophagus. Neurogastroenterology and Motility 2012; 24: 96-.
145. Lubwama, R. N., Chandler, J., and Kou, T. D. Impact of gastroesophageal reflux disease (GERD) definition on incidence rate of endoscopies among osteoporotic patients. Pharmacoepidemiology and Drug Safety 2012; 21: 421-422.
146. Gilani, S. N. S., Bass, G., Staunton, P., Kilc, S., Chrysostomou, K., Himy, N., Downes, M. R., Caffrey, J. F., Tobbia, I., and Walsh, T. N. Bile reflux: the link between cholecystectomy, Barrett's and oesophageal adenocarcinoma. Irish Journal of Medical Science 2012; 181: S14-S15.
147. Miranda, A., Romano, M., Ricciolino, S., Iadevaia, M., Mango, C., Gravina, A. G., Federico, A., and Loguercio, C. Does gender influence upper gastrointestinal symptoms and endoscopic diagnosis? Digestive and Liver Disease 2012; 44: S93-.
148. Parham, K. Evaluation of office-based transnasal esophagoscopy tolerance, safety and findings in the geriatric population. Journal of the American Geriatrics Society 2011; 59: S148-.
149. Tammana, V., Karpurapu, H., Chintala, R., Chava, S., Giday, S., Momodu, J., Sanderson, A., Smoot, D., Ali, E., and Sealy, P. Association of bmi and Barrett's esophagus/esophageal adenocarcinoma in african american population from an inner city hospital ACG IBD award. American Journal of Gastroenterology 2011; 106: S11-.
150. McIntire, M. Clinical and pathologic characteristics of patients with a diagnosis of intestinal metaplasia in biopsies of the gastroesophageal junction. American Journal of Gastroenterology 2011; 106: S10-.
151. Tyberg, A. M., Sundararajan, S., Zeffren, N., Ando, Y., Aden, B., Jacobson, I. M., and Gambarin-Gelwan, M. Cirrhosis and barrett's esophagus (BE): Diagnosis and prevalence. Hepatology 2011; 54: 1270A-.
152. Sanchez-Santos, R., Tome, Espineira C., Estevez, Fernandez S., Gonzalez, Fernandez S., Vazquez, Astray E., Turnes, J., Ulla, J. L., Brox, A., Marino, E., and Pinon, Cimadevila M. Esophago-gastric pathology in morbid obese: Preoperative diagnosis and influence in technique selection. Obesity Surgery 2011; 21 (8): 962-.
153. Rege, T. A., Goldblum, J. R., Falk, G., Kuo, F., and Odze, R. D. Does histology of gastroesophageal junction (GEJ) biopsies predict future development of Barrett's Esophagus (BE). Laboratory Investigation 2011; 91: 165A-.
154. Rebay, M. J., Durand, L., Cerisoli, C. L., Rodriguez, P. C., Bolino, C., De, Elizalde S., Elsner, B., Avagnina, A., and Caro, L. E. Prevalence of Barrett's esophagus, dysplasia and esophageal adenocarcinoma in a single centre in argentina. Gastrointestinal endoscopy 2011; 73 (4 SUPPL. 1): AB279-.
155. O'Leary, D. A., O'Connor, A., and O'Morain, C. A. Barrett's metaplasia: Underdiagnosed at endoscopy. Gastrointestinal endoscopy 2011; 73 (4 SUPPL. 1): AB210-AB211.
156. Manabe, N., Haruma, K., Hamada, H., Yamauchi, R., Teramen, K., Inoue, K., and Hata, J. Is the course of gastroesophageal reflux disease progressive? A 10-year follow-up of 200 patients. Gastroenterology 2011; 140 (5 SUPPL. 1): S581-S582.
157. Belhocine, K., Letessier, E., Coron, E., Boulanger, G., Galmiche, J. P., and Des Varannes, S. B. Laparoscopic sleeve gastrectomy (LSG) for obesity: Consequences on gastroesophageal reflux disease (GERD) symptoms, characteristics of reflux events and esophageal motility. Gastroenterology 2011; 140 (5 SUPPL. 1): S398-.
158. Altawil, J., Irwin, B., Jinjuvadia, R., Torrazza-Perez, E. G., and Antaki, F. Chemoprevention of Barrett's esophagus in patients with acid reflux. Gastroenterology 2011; 140 (5 SUPPL. 1): S258-.
159. Bashir, S., Doran, A. E., and Borum, M. L. Esophageal inlet patch: Experience in a tertiary care center. Gastroenterology 2011; 140 (5 SUPPL. 1): S233-.
160. Omer, Z., Liang, C., Nattinger, K. J., Yachimski, P. S., and Hur, C. Risk stratification for Barrett's esophagus: Interim results of a logistic regression analysis. Gastroenterology 2011; 140 (5 SUPPL. 1): S218-S219.
161. Bulsiewicz, W. J., Pasricha, S., Dellon, E. S., Madanick, R. D., Spacek, M., Orlando, R. C., and Shaheen, N. J. Efficacy and predictors of stricture development following radiofrequency ablation for Barrett's esophagus at a tertiary referral center. Gastroenterology 2011; 140 (5 SUPPL. 1): S217-.
162. Bulsiewicz, W. J., Pasricha, S., Dellon, E. S., Madanick, R. D., and Shaheen, N. J. Predictors of difficulty eradicating Barrett's esophagus with radiofrequency ablation. Gastroenterology 2011; 140 (5 SUPPL. 1): S216-.
163. Garman, K. S., Peery, A. F., Daugherty, N., Hoppo, T., Bream, S., Sanz, A. F., Spacek, M., Connors, D., Faulx, A. L., Chak, A., Luketich, J. D., Jobe, B. A., and Shaheen, J. Yield of transnasal endoscopy in an unselected general medical population. Gastroenterology 2011; 140 (5 SUPPL. 1): S211-.
164. Bulsiewicz, W. J., Pasricha, S., Dellon, E. S., Madanick, R. D., and Shaheen, N. J. The effect of operator experience on treatment of dysplastic Barrett's esophagus With radiofrequency ablation. Gastroenterology 2011; 140 (5 SUPPL. 1): S211-.
165. Yates, M., Luben, R., Cheong, E., Igali, L., Fitzgerald, R., Khaw, K.-T., and Hart, A. Dietary fat intake in the aetiology of Barrett's oesophagus and oesophageal adenocarcinoma - Data from a prospective cohort study (EPIC-Norfolk) using 7-day food diary data. Gastroenterology 2011; 140 (5 SUPPL. 1): S80-.
166. Wischin, C., Lenglinger, J., Wrba, F., Riegler, F. M., and Miholic, J. Reliability of reflux symptoms to detect recurrent gastro-esophageal reflux following fundoplication in patients with long segment Barrett's oesophagus. Journal of Clinical Gastroenterology 2011; 45 (2): 197-.
167. Zhang, M., Zou, X. P., Zhang, X. Q. I., Yu, C. G., Zhuge, Y. Z., Zhang, L. H., and Fan, X. S. Prevalence of Barrett's esophagus in Chinese mainland & #65,306; four years prospective study. Journal of Clinical Gastroenterology 2011; 45 (2): 184-.
168. Raman, S. R., Kella, V., Garber, S., Holover, S., and Cosgrove, J. M. Antireflux transoral incisionless fundoplication using esophyx: The New York evidence. Surgical Endoscopy and Other Interventional Techniques 2010; 24 (1 SUPPL. 1): S484-.
169. Bhattacharya, B. Granular cell tumors of the gastrointestinal tract: Clinicopathologic study of 63 cases. Histopathology 2010; 57: 89-.
170. Ngo, C., Mann, S., and Leung, J. Length of barrett's esophagus predicts progression. American Journal of Gastroenterology 2010; 105: S398-.
171. Lujan, G. and Genta, R. The inlet patch revisited: A clinicopathologic study of 569 patients with heterotopic gastric mucosa in the proximal esophagus. American Journal of Gastroenterology 2010; 105: S4-.
172. Dal-Paz, K., Navarro-Rodriguez, T., Natan, Eisig J., Correa, Barbuti R., Chinzon, D., and Prado Moraes-Filho, J. Patients with gastroesophageal reflux disease (GERD) treated with protonpump inhibitors have low adherence to outpatients-based treatment. Diseases of the Esophagus 2010; 23: 71A-72A.
173. Heimall, J., Arora, M., Holland, S. M., Heller, T., and Freeman, A. Gastrointestinal manifestations of autosomal dominant hyper IgE syndrome. Clinical Immunology 2010; 135 (2): 317-.
174. Gaddam, S., Rastogi, A., Gupta, N., Wani, S. B., Bansal, A., Singh, M., Singh, V., Reddymasu, S., Moloney, B., and Sharma, P. Prediction of Barrett's esophagus (BE) in patients with gastroesophageal reflux disease (GERD) using logistic regression model (LRM) and artificial neural network (ANN). Gastrointestinal endoscopy 2010; 71 (5): AB245-.
175. Singh, M., Gaddam, S., Singh, V., Gupta, N., Wani, S. B., Moloney, B., Higbee, A. D., Bansal, A., Rastogi, A., and Sharma, P. High prevalence of columnar lined esophagus in patients with chronic gastroesophageal reflux disease: Implications for disease definitions. Gastrointestinal endoscopy 2010; 71 (5): AB175-AB176.
176. Russo, E., Jonathan, H., Negus, R. P., Orchard, T., Teare, J. P., Thomas, H. J., and Powell, N. Longitudinal analysis of erosive and non-erosive GERD. Gastroenterology 2010; 138 (5 SUPPL. 1): S724-.
177. Vemulapalli, R., Genta, R. M., Souza, R. F., and Spechler, S. J. PPI-resistant heartburn in veteran patients: Underlying conditions and response to alterations in the dosage and type of PPI prescribed. Gastroenterology 2010; 138 (5 SUPPL. 1): S656-.
178. Ronkainen, J., Talley, N., Storskrubb, T., Vieth, M., Lind, T., Agreus, L., and Aro, P. Natural history of erosive esophagitis, the kalixanda study, a population-based study. Gastroenterology 2010; 138 (5 SUPPL. 1): S630-.
179. Gaddam, S., Wani, S. B., Gupta, N., Singh, M., Singh, V., Moloney, B., Rastogi, A., Bansal, A., and Sharma, P. Presence of hiatus hernia (HH) contributes to nocturnal reflux: Results from a large prospective cohort of patients with chronic gastroesophageal reflux disease (GERD). Gastroenterology 2010; 138 (5 SUPPL. 1): S558-.
180. Balbinot, S. S., Sakai, P., Ribeiro, U., Balbinot, R. A., Safatle-Ribeiro, A. V., Navarro-Rodriguez, T., Alves, V. A., Soares, I. C., and Carrilho, F. J. Comparative study of immunoexpression of junctional proteins (claudin 1, 3 and 4), cyclooxygenase-2 and inflammatory infiltrate in patients with erosive and non-erosive gastroesophageal reflux disease. Gastroenterology 2010; 138 (5 SUPPL. 1): S491-.
181. Galindo, G., Vassalle, J., Marcus, S. N., and Triadafilopoulos, G. Multi-modality evaluation of patients with GERD symptoms who have failed empiric PPI therapy. Gastroenterology 2010; 138 (5 SUPPL. 1): S487-.
182. Bardhan, K. D., Royston, C., Hoeroldt, B., Willemse, P. J., Lambertz, M. M., Srinivas, M., and Basumani, P. The natural history of Barrett's esophagus (BE): The view from a uk district general hospital (DGH). Gastroenterology 2010; 138 (5 SUPPL. 1): S334-.
183. Dann, M. D., Pickett-Blakely, O. E., Reynon, M. A., Walton, K. A., Furlong, M., Ebert, E. C., Shen, E., Manoukian, A. V., Michael, H., Ben-Menachem, T., and Das, K. M. Detection of early metaplasia at the esophagogastric junction (EGJ) in the absence of histological Barrett's epithelium using a monoclonal antibody. Gastroenterology 2010; 138 (5 SUPPL. 1): S332-.
184. Martinek, J., Strosova, A., Kostalova, K., Inna, T., Hrabal, P., Zavada, F., Suchanek, S., Stefanova, M., and Zavoral, M. Significant esophageal eosinophilia and typical endoscopic features are highly but not exclusively specific for eosinofilic esophagitis. Gastroenterology 2010; 138 (5 SUPPL. 1): S177-S178.
185. Rubenstein, J. H., Mattek, N., and Eisen, G. M. Assessment of risk for Barrett's esophagus by demographic features. Gastroenterology 2009; 136 (5 SUPPL. 1): A491-.
186. Tsibouris, P., Goula, K., Kalantzis, C., Apostolopoulos, P., Tsironis, C., Alexandrakis, G., Karameris, A., and Kalantzis, N. Carditis can partially regress after H. pylori eradication but not proton pump inhibitor treatment. Helicobacter 2009; 14 (4): 364-.
187. Tsibouris, P., Goula, K., Kalantzis, C., Liatsos, C., Tsironis, C., Kalafatis, E., Karameris, A., and Kalantzis, N. Intestinal metaplasia of the gastric cardia is more frequent in GERD patients with family history of GERD. Helicobacter 2009; 14 (4): 337-.
188. Cariello, M., Carrillo, P. A., Abecia, V. H., Talamazzi, A. R., Pogorelsky, V., Gomez, C. A. M., Davolos, J., and Paula, J. A. Epidemiology of Barrett esophagus in a university hospital health maintenance organization. Gastrointestinal endoscopy 2009; 69 (5): AB361-.
189. Poh, C. H., Gasiorowska, A., Navarro-Rodriguez, T., Willis, M. R., Noelck, N. J., Wendel, C., and Fass, R. Prevalence of upper gastrointestinal tract findings in patients with heartburn who failed PPI treatment versus those off antireflux treatment. Gastrointestinal endoscopy 2009; 69 (5): AB358-.
190. Chaurand-Lara, M., Sobrino-Cossio, S. R., Hernandez-Guerrero, A., Alonso-Larraga, J. O., Barranco, B. F., Alvaro, J., and Mora-Levy, J.-G. Narrow band imaging to identify short & ultra-short segment Barrett's esophagus in the presence of an irregular squamocolumnar junction. Gastrointestinal endoscopy 2009; 69 (5): AB354-.
191. Marek, T. A., Mularczyk, A., Szczepanska, A. J., Palen, P., Dziurkowska-Marek, A., Kajor, M., and Hartleb, M. Increasing prevalence of Barrett's esophagus with relatively low risk of neoplasia - Three-year prospective cohort study from central European country. Gastrointestinal endoscopy 2009; 69 (5): AB351-AB352.
192. Wu, W., Song, D.-D., Yuan, Y.-Z., Wu, Y.-L., and Zhu, Q. Esophageal adenocarcinoma in Shanghai China: A retrospective study. Digestion 2009; 79 (3 SUPPL. 1): 74-.
193. Lee, S. W., Jung, S. W., Ahn, J. H., Kim, S. Y., Park, S. M., Kim, J. N., Kim, D. I., Koo, J. S., Yim, H. J., Lee, H. S., and Choi, J. H. Risk factor of erosive esophagitis for adults with normal Z-line in Korea. Digestion 2009; 79 (3 SUPPL. 1): 73-.
194. Son, B. K., Baek, D. H., Jung, J. Y., Jun, D. W., Kim, S. H., Jo, Y. J., and Park, Y. S. Role of upper endoscopic evaluation in atypical chest pain patients with normal coronary angiographic fnding. Journal of gastroenterology and hepatology 2009; 24: A98-.
195. Kearney, D. J., Crump, C., Maynard, C., and Fisher, D. Exposure to EGD and mortality in a VA population with GERD. Evidence-Based Gastroenterology 2003; 4 (4): 120-121.
196. Gerson, L. B., Shetler, K., Triadafilopoulos, G., and Shaheen, N. J. What is the prevalence of Barrett's esophagus in asymptomatic populations? Evidence-Based Gastroenterology 2002; 3 (4): 110-111.
197. Shiota, S., Singh, S., Anshasi, A., and El-Serag, H. B. The prevalence of Barrett's esophagus in Asian countries: A systematic review and meta-analysis. Gastroenterology 2015; 148 (4 SUPPL. 1): S403-.
198. Gerson, L. B., Rutenberg, M., and Scott, R. Incremental benefit of computer-assisted brush-biopsy (WATS3D) compared to standard endoscopic biopsy for detection of barrett's esophagus (BE) and dysplasia: Systematic review and meta-analysis. Gastroenterology 2014; 146 (5 SUPPL. 1): S-307.
199. Miller, M. C., Brenner, A., Kroep, S., Inadomi, J. M., and Hur, C. Systematic review: Is there a secular trend in barrett's esophagus prevalence and incidence? Gastroenterology 2014; 146 (5 SUPPL. 1): S-185.
200. Pace, F. GERD natural history. Diseases of the Esophagus 2012; 25: 12A-13A.
201. Taylor, J. B. and Rubenstein, J. H. Meta-analysis of the association of gastroesophageal reflux disease with Barrett's esophagus: No association with short segment Barrett's esophagus. Gastroenterology 2010; 138 (5 SUPPL. 1): S330-.
202. Arastu, S., Henry, C. H., Infantolino, A., and Quirk, D. The frequency with which gastroenterologists follow guidelines in surveillance of barrett's esophagus. American Journal of Gastroenterology 2016; 111: S201-.
203. Wang, K., Duan, L., Zhang, L., Liu, Z., He, Z., and Ke, Y. The prevalence of GERD subtypes in the natural population with high esophageal cancer incidence of China. Journal of Gastroenterology and Hepatology (Australia) 2016; 31: 23-.
204. Takeshita, E., Sakata, Y., Hara, M., Tsuruoka, N., Sakata, N., Akutagawa, K., Matsunaga, K., Kusano, M., Iwakiri, R., and Fujimoto, K. Reflux symptoms and acid-related dyspepsia are more common in females than males with or without endoscopic esophagitis: Analysis of 3,505 Japanese healthy subjects undergoing medical health checkups. Journal of Gastroenterology and Hepatology (Australia) 2016; 31: 301-.
205. Rubenstein, J., McConnell, D., Beer, D., Chak, A., Metko, V., and Clines, G. Associations of vitamin d deficiency and hyperparathyroidism with gastroesophageal reflux disease and its sequelae. American Journal of Gastroenterology 2016; 111: S203-S204.
206. Okada, T. and Adkins, G. Light endoscopic findings of okadaella gastrococcus like bacterium positive reactive gastropathy (OGLBP-RG). American Journal of Gastroenterology 2016; 111 (): S482-.
207. Chamarthy, P., Razjouyan, H., Chokhavatia, S., and Wang, X. Higher prevalence of non-erosive reflux disease (NERD) than erosive reflux disease (ERD) in type 2 diabetes mellitus (T2DM) patients. American Journal of Gastroenterology 2016; 111 (): S1249-S1250.
208. Yin, C., Zhang, J., and Wang, J. Prevalence and risk factors of Barrett's esophagusin Northwest China. Journal of Gastroenterology and Hepatology (Australia) 2016; 31: 24-.
209. David, W. J., Qumseya, B. J., Bartel, M. J., Badillo, R. J., Bhalla, R., Brahmbhatt, B., Melendez-Rosado, J., Moris, M., Kroner, T., Wolfsen, H. C., and Wallace, M. B. Interobserver agreement for differentiating normal vs. barrett's esophagus and erosive vs. non erosive esophagitis using volumetric laser endomicroscopy imaging technique. Gastroenterology 2014; 146 (5 SUPPL. 1): S-219.
210. Bond, A., Wilson, D., Hastings, A., Veeramootoo, D., and Singh, K. K. Barrett's oesophagus infers a higher rate of reflux following fundoplication. Diseases of the Esophagus 2012; 25:74A-.
211. Rebecchi, F., Giaccone, C., Festa, F., and Morino, M. Disappointing results of laparoscopic total fundoplication in the control of non acid reflux. Surgical Endoscopy and Other Interventional Techniques 2011; 25: S42-.
212. Dellon, E. S., Bower, J. J., Keku, T. O., Chen, X. L., Miller, C. R., Woosley, J. T., Orlando, R. C., and Shaheen, N. J. Markers of tyrosine kinase activity for diagnosis and treatment response in eosinophilic esophagitis: A pilot study of pERK 1/2 and pSTAT5. Gastroenterology 2011; 140 (5 SUPPL. 1): S187-.
213. Kahn, A., Al-Qaisi, M., Callaway, J., Burdick, G., Crowell, M. D., Vela, M., and Ramirez, F. C. The clinical course of women with barrett's esophagus. American Journal of Gastroenterology 2016; 111: S226-S227.
214. Takalkar, U. V., Kulkarni, U., and Reddy, D. N. Diagnostic yield of upper gastrointestinal endoscopy with reference to Barrett's esophagus in a tertiary care center from India. Journal of Gastroenterology and Hepatology (Australia) 2016; 31: 26-.
215. Arroyo-Martinez, Q., Rodriguez-Tellez, M., Garcia-Escudero, A., Brugal-Molina, J., Gonzalez-Campora, R., and Caunedo-Alvarez, A. Stem cells from submucosal glands as a possible origin for Barretts esophagus epithelium. Gastroenterology 2016; 150 (4 SUPPL. 1): S851-S852.
216. Gorodner, M. V., Clemente, G., and Grigaites, A. Barrett's esophagus after roux-en-y gastric bypass: Does regression occur? Surgical Endoscopy and Other Interventional Techniques 2016; 30: S255-.
217. Reynolds, J. L., Doggett, S., Mguyen, V., Bildzukewicz, N. A., Buckley III, F. P., and Lipham, J. C. Predicting magnetic sphincter augmentation failure. Surgical Endoscopy and Other Interventional Techniques 2016; 30: S237-. Najarian, R., Ketwaroo, G., Patil, D., and Lembo, A. Esophageal squamous papillomas: Incidentaloma or harbinger of human papilloma virus infection? Laboratory Investigation 2016; 96: 188A-.
218. Wang, Z., Lee, H.-C., Giacomelli, M. G., Liang, K., Ahsen, O. O., Figueiredo, M., Huang, Q., Fujimoto, J. G., and Mashimo, H. Automated optical coherence tomography image processing for three dimensional detection and visualization of subsurface glands in barrett's esophagus with dysplasia. Gastroenterology 2015; 148 (4 SUPPL. 1): S580-S581.
219. Siddiki, H. A., Lam-Himlin, D., Kahn, A., Alhalabi, K., Burdick, G. E., Crowell, M. D., De, Petris G., Pannala, R., Ramirez, F. C., and Fleischer, D. E. Intestinal metaplasia (IM) of the cardia: Incidence in patients with barrett's esophagus (BE) and those undergoing EGD for other indications. Gastroenterology 2015; 148 (4 SUPPL. 1): S219-.
220. Salem, A., Matthews, S., Mori, Y., Ibrahim, S., Meltzer, S., and Roland, B. C. Prevalence and associated risk factors of helicobacter pylori-negative gastritis. American Journal of Gastroenterology 2014; 109: S40-.
221. Wang, W., Uedo, N., Yang, Y., Peng, L., Wang, J., Li, H., and Wang, X. Autofluorescence imaging videoendoscopy predicts acid reflux in patients with gastroesophageal reflux disease. Gastroenterology 2013; 144 (5 SUPPL. 1): S848-.
222. Goldberg, A., Gerkin, R., and Young, M. A. Medical prevention of barrett's esophagus: Effects of statins and aspirin/NSAIDs. Gastroenterology 2013; 144 (5 SUPPL. 1): S701-.
223. Onuchina, E. and Tsukanov, V. The progressive course of gastroesophageal reflux disease in the elderly and its risk factors. Journal of gastroenterology and hepatology 2013; 28: 63-.
224. Abdalla, Y., Oshima, K., Howard, A., and Hachem, C. Practice patterns and outcomes in barrett's esophagus indeterminate for dysplasia. Gastrointestinal endoscopy 2012; 75 (4 SUPPL. 1): AB455-AB456.
225. Bandla, S., Thoms, K., Litle, V., Watson, T., Peters, J., Song, K., Godfrey, T. E., and Zhou, Z. Genomic analysis of esophageal columnar cell metaplasia reveals less frequent changes in non-goblet cell metaplasia than intestinal metaplasia. Laboratory Investigation 2012; 92: 155A-.
226. Cheung, C. K. Y., Wu, J., Chan, Y., Ching, J., Chan, F. K. L., and Sung, J. J. Increasing severity of reflux esophagitis (RE) among young patients with gastroesophageal reflux disease (GERD) in Chinese population: A 10-year prospective study. Gastroenterology 2009; 136 (5 SUPPL. 1): A737-.
227. Transnasal Oesophagoscopy (TNE). Health Technology Assessment Report, MOH/P/PAK/231.12 (TR), Issue 2011/111. Retrieved from: <http://www.moh.gov.my/v/hta>.
228. Endoscopic Ultrasound vs. Traditional Endoscopy for the Identification of Common Biliary and Gastroesophagael Problems: Clinical and Cost-Effectiveness.February 4, 2008. Retrieved from: <https://v2disprod.evidencepartners.com/Generic/getAttachment2.php?id=1076>
229. Gralnek IM, Adler SN, Yassin K, Koslowsky B, Metzger Y, Eliakim R. Detecting esophageal disease with second-generation capsule endoscopy: initial evaluation of the PillCam ESO 2. Endoscopy. 2008 Apr;40(04):275-9.
230. Neu B, Wettschureck E, Rösch T. Is esophageal capsule endoscopy feasible? Results of a pilot. Endoscopy. 2003 Nov;35(11):957-61.

### Study design: Narrative review/report (n=349)

1. Vaezi, Michael F., Brill, Joel V., Mills, Michael R., Bernstein, Brett B., Ness, Reid M., Richards, William O., Brillstein, Lili, Leibowitz, Rebecca, Strople, Ken, Montgomery, Elizabeth A., and Patel, Kavita An Episode Payment Framework for Gastroesophageal Reflux Disease. Gastroenterology 2016; 150 (4): 1019-1025.
2. Vaezi, Michael F., Brill, Joel V., Mills, Michael R., Bernstein, Brett B., Ness, Reid M., Richards, William O., Brillstein, Lili, Leibowitz, Rebecca, Strople, Ken, Montgomery, Elizabeth A., and Patel, Kavita An Episode Payment Framework for Gastroesophageal Reflux Disease: Symptomatic Gastroesophageal Reflux Disease, Dysplastic and Nondysplastic Barrett's Esophagus, and Anti-Reflux Surgical and Endoscopic Interventions. Gastroenterology 2016; 150 (4): 1009-1018.
3. Leggit, Jeffrey C. Evaluation and treatment of GERD and upper GI complaints in athletes. Current sports medicine reports 2011; 10 (2): 109-114.
4. Scholl, Shannon, Dellon, Evan S., and Shaheen, Nicholas J. Treatment of GERD and proton pump inhibitor use in the elderly: practical approaches and frequently asked questions. The American journal of gastroenterology 2011; 106 (3): 386-392.
5. Morganstern, Bradley and Anandasabapathy, Sharmila GERD and Barrett's esophagus: diagnostic and management strategies in the geriatric population. Geriatrics 2009; 64 (7): 9-12.
6. Shaheen, Nicholas J. and Palmer, Lena B. Improving screening practices for Barrett's esophagus. Surgical oncology clinics of North America 2009; 18 (3): 423-437.
7. Ricer, Rick E. and Li, Christina Capsule endoscopy. What can we expect? The Journal of family practice 2005; Suppl: 15-18.
8. Piterman, Leon, Nelson, Mark, and Dent, John Gastro-oesophageal reflux disease--current concepts in management. Australian family physician 2004; 33 (12): 987-991.
9. Shalauta, Mark D. and Saad, Richard Barrett's esophagus. American family physician 2004; 69 (9): 2113-2118.
10. Spechler, S. J. and Barr, H. Review article: screening and surveillance of Barrett's oesophagus: what is a cost-effective framework? Alimentary pharmacology & therapeutics 2004; 19 Suppl 1: 49-53.
11. Spechler, Stuart Jon Barrett's esophagus and esophageal adenocarcinoma: pathogenesis, diagnosis, and therapy. The Medical clinics of North America 2002; 86 (6): 1423-1vii.
12. Delforge, M., Plomteux, O., Delfosse, V., Fontaine, F., Louis, E., and Societe Liegeoise de Gastro-Enterologie [Barrett's esophagus: overview]. Revue medicale de Liege 2002; 57 (8): 535-545.
13. Spechler, Stuart Jon Clinical practice. Barrett's Esophagus. The New England journal of medicine 2002; 346 (11): 836-842.
14. Axon, A. T. Chronic dyspepsia: who needs endoscopy? Gastroenterology 1997; 112 (4): 1376-1380.
15. Fock, K. M., Talley, N., Goh, K. L., Sugano, K., Katelaris, P., Holtmann, G., Pandolfino, J. E., Sharma, P., Ang, T. L., Hongo, M., Wu, J., Chen, M., Choi, M.-G., Law, N. M., Sheu, B.-S., Zhang, J., Ho, K. Y., Sollano, J., Rani, A. A., Kositchaiwat, C., and Bhatia, S. Asia-Pacific consensus on the management of gastro-oesophageal reflux disease: An update focusing on refractory reflux disease and Barrett's oesophagus. Gut 2016; 65 (9): 1402-1415.
16. Rao, V. L., Micic, D., and Kim, K. E. Primary Care Evaluation and Management of Gastroenterologic Issues in Women. Obstetrics and Gynecology Clinics of North America 2016; 43 (2): 347-366.
17. Kapoor, H., Agrawal, D. K., and Mittal, S. K. Barrett's esophagus: Recent insights into pathogenesis and cellular ontogeny. Translational Research 2015; 166 (1): 28-40.
18. Sheu, B.-S., Chiu, C.-T., Lee, Y.-C., Chang, C.-Y., Wu, D.-C., Liou, J.-M., Wu, M.-S., Chang, W.-L., Wu, C.-Y., and Lin, J.-T. Consensus of gastroesophageal reflux disease in Taiwan with endoscopy-based approach covered by National Health Insurance. Advances in Digestive Medicine 2015; 2 (3): 85-94.
19. Sharma, P., Katzka, D. A., Gupta, N., Ajani, J., Buttar, N., Chak, A., Corley, D., El-Serag, H., Falk, G. W., Fitzgerald, R., Goldblum, J., Gress, F., Ilson, D. H., Inadomi, J. M., Kuipers, E. J., Lynch, J. P., McKeon, F., Metz, D., Pasricha, P. J., Pech, O., Peek, R., Peters, J. H., Repici, A., Seewald, S., Shaheen, N. J., Souza, R. F., Spechler, S. J., Vennalaganti, P., and Wang, K. Quality Indicators for the Management of Barrett's Esophagus, Dysplasia, and Esophageal Adenocarcinoma: International Consensus Recommendations from the American Gastroenterological Association Symposium. Gastroenterology 2015; 149 (6): 1599-1606.
20. Gupta, M. and Iyer, P. G. Screening for Barrett's Esophagus. Gastroenterology Clinics of North America 2015; 44 (2): 265-283.
21. Rubenstein, J. H. and Thrift, A. P. Risk factors and populations at risk: Selection of patients for screening for Barrett's oesophagus. Best Practice and Research: Clinical Gastroenterology 2015; 29 (1): 41-50.
22. Ro, T. H., Mathew, M. A., and Misra, S. Value of screening endoscopy in evaluation of esophageal, gastric and colon cancers 2015 Advances in Gastrointestinal Endoscopy. World journal of gastroenterology 2015; 21 (33): 9693-9706.
23. Hosoe, N., Naganuma, M., and Ogata, H. Current status of capsule endoscopy through a whole digestive tract. Digestive Endoscopy 2015; 27 (2): 205-215.
24. Ramzan, Z., Nassri, A. B., and Huerta, S. The use of imaging and biomarkers in diagnosing Barrett's esophagus and predicting the risk of neoplastic progression. Expert Review of Molecular Diagnostics 2014; 14 (5): 575-591.
25. Wood, R. K. Endoscopic aspects in diagnosis of gastroesophageal reflux disease and motility disorders: Bravo, capsule, and functional lumen imaging probe. Techniques in Gastrointestinal Endoscopy 2014; 16 (1): 2-9.
26. Loughrey, M. B. and Johnston, B. T. Guidance on the effective use of upper gastrointestinal histopathology. Frontline Gastroenterology 2014; 5 (2): 88-95.
27. Pauli, E. M. and Ponsky, J. L. A modern history of the surgeon-endoscopist. Techniques in Gastrointestinal Endoscopy 2013; 15 (4): 166-172.
28. Chokhavatia, S., Alli-Akintade, L., Harpaz, N., and Stern, R. Esophageal Pathology: A Brief Guide and Atlas. Otolaryngologic Clinics of North America 2013; 46 (6): 1043-1057.
29. Lagergren, J. and Lagergren, P. Recent developments in esophageal adenocarcinoma. CA Cancer Journal for Clinicians 2013; 63 (4): 232-248.
30. Katz, P. O., Gerson, L. B., and Vela, M. F. Guidelines for the diagnosis and management of gastroesophageal reflux disease. American Journal of Gastroenterology 2013; 108 (3): 308-328.
31. Varghese, Jr, Hofstetter, W. L., Rizk, N. P., Low, D. E., Darling, G. E., Watson, T. J., Mitchell, J. D., and Krasna, M. J. The society of thoracic surgeons guidelines on the diagnosis and staging of patients with esophageal cancer. Annals of Thoracic Surgery 2013; 96 (1): 346-356.
32. Illig, R., Klieser, E., Kiesslich, T., and Neureiter, D. GERD - Barrett - Adenocarcinoma: Do we have suitable prognostic and predictive molecular markers? Gastroenterology Research and Practice 2013 Article ID 643084, 14 pages
33. Krugmann, J., Neumann, H., Vieth, M., and Armstrong, D. What is the role of endoscopy and oesophageal biopsies in the management of GERD? Best Practice and Research: Clinical Gastroenterology 2013; 27 (3): 373-385.
34. Kheraj, R., Tewani, S. K., Ketwaroo, G., and Leffler, D. A. Quality Improvement in Gastroenterology Clinical Practice. Clinical Gastroenterology and Hepatology 2012; 10 (12): 1305-1314.
35. Appelman, H. D., Umar, A., Orlando, R. C., Sontag, S. J., Nandurkar, S., El-Zimaity, H., Lanas, A., Parise, P., Lambert, R., and Shields, H. M. Barrett's esophagus: Natural history. Ann New York Acad.Sci 2011; 1232 (1): 292-308.
36. Kadri, S., Lao-Sirieix, P., and Fitzgerald, R. C. Developing a nonendoscopic screening test for Barrett's esophagus. Biomarkers in Medicine 2011; 5 (3): 397-404.
37. Cash, B. D., Banerjee, S., Anderson, M. A., Ben-Menachem, T., Decker, G. A., Fanelli, R. D., Fukami, N., Ikenberry, S. O., Jain, R., Jue, T. L., Khad, K. M., Krinsky, M. L., Malpas, P. M., Maple, J. T., Sharaf, R., and Dominitz, J. A. Ethnic issues in endoscopy. Gastrointestinal endoscopy 2010; 71 (7): 1108-1112.
38. Ciriza-de-los-Rios, C. Barrett's esophagus - a review. Revista Espanola de Enfermedades Digestivas 2010; 102 (4): 257-269.
39. Garud, S. S., Keilin, S., Qiang, Cai, and Willingham, F. F. Review: Diagnosis and management of Barrett's esophagus for the endoscopist. Therapeutic Advances in Gastroenterology 2010; 3 (4): 227-238.
40. Spechler, S. J., Fitzgerald, R. C., Prasad, G. A., and Wang, K. K. History, Molecular Mechanisms, and Endoscopic Treatment of Barrett's Esophagus. Gastroenterology 2010; 138 (3): 854-869.
41. Lambert, R. and Halnaut, P. Esophageal cancer: Cases and causes (Part I). Endoscopy 2007; 39 (6): 550-555.
42. Schuchert, M. J. and Luketich, J. D. Management of Barrett's esophagus. Oncology 2007; 21 (11): 1382-1392.
43. Obesity. Annals of Internal Medicine 2008; 149 (7): ITC41-ITC416.
44. Wang, K. K. and Sampliner, R. E. Updated guidelines 2008 for the diagnosis, surveillance and therapy of Barrett's esophagus. American Journal of Gastroenterology 2008; 103 (3): 788-797.
45. Barritt IV, A. S. and Shaheen, N. J. Should patients with Barrett's oesophagus be kept under surveillance? The case against. Best Practice and Research: Clinical Gastroenterology 2008; 22 (4): 741-750.
46. Lichtenstein, D. R., Cash, B. D., Davila, R., Baron, T. H., Adler, D. G., Anderson, M. A., Dominitz, J. A., Gan, S.-I., Harrison III, M. E., Ikenberry, S. O., Qureshi, W. A., Rajan, E., Shen, B., Zuckerman, M. J., Fanelli, R. D., and VanGuilder, T. Role of endoscopy in the management of GERD. Gastrointestinal endoscopy 2007; 66 (2): 219-224.
47. Tse, E. and Holloway, R. H. Update on gastro-oesophageal reflux disease. Medicine Today 2007; 8 (4): 14-22.
48. Armstrong, D., Marshall, J. K., Chiba, N., Enns, R., Fallone, C. A., Fass, R., Hollingworth, R., Hunt, R. H., Kahrilas, P. J., Mayrand, S., Moayyedi, P., Paterson, W. G., Sadowski, D., and Veldhuyzen van Zanten, S. J. O. Canadian Concensus Conference on the management of gastroesophageal reflux disease in adults - Update 2004. Canadian Journal of Gastroenterology 2005; 19 (1): 15-35.
49. Aviv, J. E. Transnasal esophagoscopy: State of the art. Otolaryngology - Head and Neck Surgery 2006; 135 (4): 616-619.
50. Williams, L. J., Guernsey, D. L., and Casson, A. G. Biomarkers in the molecular pathogenesis of esophageal (Barrett) adenocarcinoma. Current Oncology 2006; 13 (1): 33.
51. Pennathur, A., Landreneau, R. J., and Luketich, J. D. Surgical aspects of the patient with high-grade dysplasia. Seminars in Thoracic and Cardiovascular Surgery 2006; 17 (4): 326-332.
52. Hirota, W. K., Zuckerman, M. J., Adler, D. G., Davila, R. E., Egan, J., Leighton, J. A., Qureshi, W. A., Rajan, E., Fanelli, R., Wheeler-Harbaugh, J., Baron, T. H., and Faigel, D. O. ASGE guideline: The role of endoscopy in the surveillance of premalignant conditions of the upper GI tract. Gastrointestinal endoscopy 2006; 63 (4): 570-580.
53. Sharma, V. K., Eliakim, R., Sharma, P., and Faigel, D. ICCE consensus for esophageal capsule endoscopy. Endoscopy 2005; 37 (10): 1060-1064.
54. Smith, L. and Coughlin, L. Updated ACG guidelines for diagnosis and treatment of GERD. American family physician 2005; 71 (12): 2376-2383.
55. Sidorenko, E. I. and Sharma, P. High-resolution chromoendoscopy in the esophagus. Gastrointestinal Endoscopy Clinics of North America 2004; 14 (3): 437-451.
56. Spechler, S. J. Review article: What I do now to manage adenocarcinoma risk, and what I may be doing in 10 years' time. Alimentary Pharmacology and Therapeutics, Supplement 2004; 20 (5): 105-110.
57. Katelaris, P., Holloway, R., Talley, N., Gotley, D., Williams, S., and Dent, J. Gastro-oesophageal reflux disease in adults: Guidelines for clinicians. Journal of Gastroenterology and Hepatology (Australia) 2002; 17 (8): 825-833.
58. Spechler, S. J. Barrett's esophagus. New England Journal of Medicine 2002; 346 (11): 836-842.
59. Basu, K. K. and De Caestecker, J. S. Surveillance in Barrett's oesophagus: A personal view. Postgraduate Medical Journal 2002; 78 (919): 263-268.
60. Spechler, S. J. Acid suppression therapy for Barrett's esophagus. European Journal of Surgery, Supplement 2001; 167 (586): 78-81.
61. Freston, J. W. Motion - All patients with GERD should be offered once in a lifetime endoscopy: Arguments against the motion. Canadian Journal of Gastroenterology 2002; 16 (8): 555-558.
62. Falk, G. W. Barrett's esophagus. Clinical Perspectives in Gastroenterology 2001; 4 (5): 267-275.
63. Spechler, S. J. Barrett's oesophagus: Diagnosis and management. Bailliere's Best Practice and Research in Clinical Gastroenterology 2000; 14 (5): 857-879.
64. Falk, G. W. Reflux disease and Barrett's esophagus. Endoscopy 1999; 31 (1): 9-16.
65. Lambert, R. The role of endoscopy in the prevention of esophagogastric cancer. Endoscopy 1999; 31 (2): 180-199.
66. Moss, S. F. Consensus statement for management of gastroesophageal reflux disease: Result of workshop meeting at Yale University School of Medicine, Department of Surgery, November 16 and 17, 1997. Journal of Clinical Gastroenterology 1998; 27 (1): 6-12.
67. Bhardwaj, A., Hollenbeak, C. S., Pooran, N., and Mathew, A. A meta-analysis of the diagnostic accuracy of esophageal capsule endoscopy for Barrett's esophagus in patients with gastroesophageal reflux disease (DARE structured abstract). American Journal of Gastroenterology 2009; 104: 1533-1539.
68. Singh, Rajvinder, Ragunath, Krish, and Jankowski, Janusz Barrett's Esophagus: Diagnosis, Screening, Surveillance, and Controversies. Gut and liver 2007; 1 (2): 93-100.
69. Barr, H., Kendall, C., and Stone, N. The light solution for Barrett's oesophagus Photodiagnosis and photodynamic therapy for columnar-lined oesophagus. Photodiagnosis and photodynamic therapy 2004; 1 (1): 75-84.
70. Vaira, Dino, Gatta, Luigi, Ricci, Chiara, Castelli, Valentina, Fiorini, Giulia, Kajo, Enkeleda, and Lanzini, Alberto Gastroesophageal reflux disease and Barrett's esophagus. Internal and emergency medicine 2011; 6 (4): 299-306.
71. Louis, H. Reflux disease and Barrett's esophagus. Endoscopy 2007; 39 (11): 969-973.
72. Eisen, Glenn M. Capsule endoscopy. New applications. The Journal of Family Practice 2005; 54(12): S9-
73. Kyrgidis, Athanassios, Kountouras, Jannis, Zavos, Christos, and Chatzopoulos, Dimitrios New molecular concepts of Barrett's esophagus: clinical implications and biomarkers. The Journal of surgical research 2005; 125 (2): 189-212.
74. Bergman, J. J. G. H. Gastroesophageal reflux disease and Barrett's esophagus. Endoscopy 2005; 37 (1): 8-18.
75. Peters, J. H. and Wang, K. K. How should Barrett's ulceration be treated? Surgical endoscopy 2004; 18 (2): 338-344.
76. DeVault, K. R. and Castell, D. O. Current diagnosis and treatment of gastroesophageal reflux disease. Mayo Clinic proceedings 1994; 69 (9): 867-876.
77. Pophali, P. and Halland, M. Barrett's oesophagus: Diagnosis and management. BMJ (Online) 2016; 353:i2373.
78. Lin, J., Kligerman, S., Goel, R., Sajedi, P., Suntharalingam, M., and Chuong, M. D. State-of-the-art molecular imaging in esophageal cancer management: Implications for diagnosis, prognosis, and treatment. Journal of Gastrointestinal Oncology 2015; 6 (1): 3-19.
79. Pech, O. Reflux and Barrett's disease. Endoscopy 2014; 46 (4): 306-309.
80. Pech, O. Esophageal disease. Gastrointestinal endoscopy 2013; 78 (3): 405-409.
81. De Palma, G. D. Management strategies of Barrett's esophagus. World journal of gastroenterology 2012; 18 (43): 6216-6225.
82. Anand, O., Wani, S., and Sharma, P. Gastroesophageal reflux disease and Barrett's esophagus. Endoscopy 2008; 40 (2): 126-130.
83. Sayana, H., Wani, S., and Sharma, P. Esophageal adenocarcinoma and Barrett's esophagus. Minerva Gastroenterologica e Dietologica 2007; 53 (2): 157-169.
84. Musana, A. K., Yale, S. H., and Lang, K. A. Managing dyspepsia in a primary care setting. Clinical Medicine and Research 2006; 4 (4): 337-342.
85. Barr, H., Stone, N., and Rembacken, B. Endoscopic therapy for Barrett's oesophagus. Gut 2005; 54 (6): 875-884.
86. Moayyedi, P. and Ford, A. Recent developments in gastroenterology. British Medical Journal 2002; 325 (7377): 1399-1402.
87. Bass, B. L. What's new in general surgery: Gastrointestinal conditions. Journal of the American College of Surgeons 2002; 195 (6): 835-854.
88. Koop, H. Reflux disease and Barrett's esophagus. Endoscopy 2000; 32 (2): 101-107.
89. Chang, Chi Yang, Cook, Michael B., Lee, Yi Chia, Lin, Jaw Town, Ando, Takafumi, Bhatia, Shobna, Chow, Wong Ho, El-Omar, Emad M., Goto, Hidemi, Li, Yang Qing, McColl, Kenneth, Reddy, Nageshwar, Rhee, Poong Lyul, Sharma, Prateek, Sung, Joseph J. Y., Ghoshal, Uday, Wong, Jennie Y. Y., Wu, Justin C. Y., Zhang, Jun, Ho, Khek Yu, and Asian Barrett's Consortium Current status of Barrett's esophagus research in Asia. Journal of gastroenterology and hepatology 2011; 26 (2): 240-246.
90. Schuchert, Matthew J., McGrath, Kevin, and Buenaventura, Percival O. Barrett's esophagus: diagnostic approaches and surveillance. Seminars in Thoracic and Cardiovascular Surgery 2005; 17 (4): 301-312.
91. Sharma, P. and Sidorenko, E. I. Are screening and surveillance for Barrett's oesophagus really worthwhile? Gut 2005; 54 Suppl 1: i27-i32.
92. Chen, X., Zhu, L.-R., and Hou, K.-H. The characteristics of Barrett's esophagus: An analysis of 4120 cases in China. Diseases of the Esophagus 2009; 22 (4): 348-353.
93. Quera, R., O'Sullivan, K., and Quigley, E. M. M. Surveillance in barrett's oesophagus: Will a strategy focused on a high-risk group reduce mortality from oesophageal adenocarcinoma? Endoscopy 2006; 38 (2): 162-169.
94. Sharma, P. Review article: Prevalence of Barrett's oesophagus and metaplasia at the gastro-oesophageal junction. Alimentary Pharmacology and Therapeutics, Supplement 2004; 20 (5): 48-54.
95. Assimakopoulos, D. and Patrikakos, G. The role of gastroesophageal reflux in the pathogenesis of laryngeal carcinoma. American Journal of Otolaryngology - Head and Neck Medicine and Surgery 2002; 23 (6): 351-357.
96. Spechler, S. J. Screening for Barrett's esophagus. Reviews in gastroenterological disorders 2002; 2 (SUPPL. 2): S25-S29.
97. Gerson, L. B. and Triadafilopoulos, G. Screening for esophageal adenocarcinoma: An evidence-based approach. American Journal of Medicine 2002; 113 (6): 499-505.
98. Pech, O., Gossner, L., May, A., and Ell, C. Management of Barrett's oesophagus, dysplasia and early adenocarcinoma. Best Practice and Research: Clinical Gastroenterology 2001; 15 (2): 267-284.
99. Sontag, S. J. Preventing death of Barrett's cancer: Does frequent surveillance endoscopy do it? American Journal of Medicine 2001; 111 (8 SUPPL. 1): 137S-141S.
100. Riddell, R. H. Early detection of neoplasia of the esophagus and gastroesophageal junction. American Journal of Gastroenterology 1996; 91 (5): 853-863.
101. Cooper, G. S. Indications and contraindications for upper gastrointestinal endoscopy. Gastrointestinal Endoscopy Clinics of North America 1994; 4 (3): 439-454.
102. Hetzel, D. P. and McCallum, R. W. The natural history of reflux esophagitis. Acta Endoscopica 1993; 23 (2): 73-81.
103. Whiteman, David C. and Kendall, Bradley J. Barrett's oesophagus: epidemiology, diagnosis and clinical management. The Medical journal of Australia 2016; 205 (7): 317-324.
104. Fry, Lucia C., Monkemuller, Klaus, and Malfertheiner, Peter Functional heartburn, nonerosive reflux disease, and reflux esophagitis are all distinct conditions--a debate: con. Current treatment options in gastroenterology 2007; 10 (4): 305-311.
105. di Pietro, Massimiliano and Fitzgerald, Rebecca C. Screening and risk stratification for Barrett's esophagus: how to limit the clinical impact of the increasing incidence of esophageal adenocarcinoma. Gastroenterology Clinics of North America 2013; 42 (1): 155-173.
106. Buxbaum, J. L. and Eloubeidi, M. A. Endoscopic evaluation and treatment of esophageal cance. Minerva Gastroenterologica e Dietologica 2009; 55 (4): 455-469.
107. di Pietro, Massimiliano, Peters, Christopher J., and Fitzgerald, Rebecca C. Clinical puzzle: Barrett's oesophagus. Disease models & mechanisms 2008; 1 (1): 26-31.
108. Sabate, Jean Marc, Jouet, Pauline, and Coffin, Benoit [Gastroesophageal reflux in adults. Hiatal hernia]. La Revue du praticien 2006; 56 (14): 1591-1596.
109. Goldblum, John R. Barrett's esophagus and Barrett's-related dysplasia. Modern pathology 2003; 16 (4): 316-324.
110. Katzka, David A. Barrett's esophagus: surveillance and treatment. Gastroenterology Clinics of North America 2002; 31 (2): 481-497.
111. Kleeff, J., Friess, H., Liao, Q., and Buchler, M. W. Immunohistochemical presentation in non-malignant and malignant Barrett's epithelium. Diseases of the esophagus 2002; 15 (1): 10-15.
112. Spechler, S. J. Screening and surveillance for complications related to gastroesophageal reflux disease. The American journal of medicine 2001; 111 Suppl 8A: 130S-136S.
113. Gopal, D. V. Another look at Barrett's esophagus. Current thinking on screening and surveillance strategies. Postgraduate medicine 2001; 110 (3): 57-58.
114. Bammer, T., Hinder, R. A., Klaus, A., Trastek, V. F., and Achem, S. R. Rationale for surgical therapy of Barrett esophagus. Mayo Clinic proceedings 2001; 76 (3): 335-342.
115. Katzka, D. A. and Rustgi, A. K. Gastroesophageal reflux disease and Barrett's esophagus. The Medical clinics of North America 2000; 84 (5): 1137-1161.
116. Boyer, J. [Barrett's esophagus]. La Revue du praticien 1999; 49 (11): 1159-1165.
117. Kuipers, E. J. Review article: exploring the link between Helicobacter pylori and gastric cancer. Alimentary pharmacology & therapeutics 1999; 13 Suppl 1 (): 3-11.
118. Spivak, H., Lelcuk, S., and Hunter, J. G. Laparoscopic surgery of the gastroesophageal junction. World Journal of Surgery 1999; 23 (4): 356-367.
119. Mabrut, J. Y. and Baulieux, J. [Management of Barrett's esophagus]. Journal de chirurgie 1999; 136 (6): 301-308.
120. Bartlesman, J. F., Hameeteman, W., and Tytgat, G. N. Barrett's oesophagus. European journal of cancer prevention 1992; 1 (4): 323-325.
121. Tepes, B. Population based Helicobacter pylori screening and eradication: Advances versus side effects. Current Pharmaceutical Design 2014; 20 (28): 4501-4509.
122. De Jonge, P. J. F., Van, Blankenstein M., Grady, W. M., and Kuipers, E. J. Barrett's oesophagus: Epidemiology, cancer risk and implications for management. Gut 2014; 63 (1): 191-202.
123. Sharma, V. K. Role of endoscopy in GERD. Gastroenterology Clinics of North America 2014; 43 (1): 39-46.
124. Bivin, W. W., Finkelstein, S. D., and Silverman, J. F. Molecular occurrences in the neoplastic progression of barrett esophagus: Can molecular analysis play a role in risk stratification? Pathology Case Reviews 2014; 19 (1): 28-35.
125. Parker, C. E., Spada, C., Mcalindon, M., Davison, C., and Panter, S. Capsule endoscopy-not just for the small bowel: A review. Expert Review of Gastroenterology and Hepatology 2014; 9 (1): 79-89.
126. Ishimura, N., Okada, M., Mikami, H., Okimoto, E., Fukuda, N., Uno, G., Aimi, M., Oshima, N., Ishihara, S., and Kinoshita, Y. Pathophysiology of barrett's esophagus-associated neoplasia: Circumferential spatial predilection. Digestion 2014; 89 (4): 291-298.
127. Schneider, A., Michaud, L., and Gottrand, F. Esophageal atresia: Metaplasia, Barrett. Diseases of the Esophagus 2013; 26 (4): 425-427.
128. Bush, C. M. and Postma, G. N. Transnasal Esophagoscopy. Otolaryngologic Clinics of North America 2013; 46 (1): 41-52.
129. Tiwari, A. K., Laird-Fick, H. S., Wali, R. K., and Roy, H. K. Surveillance for gastrointestinal malignancies. World journal of gastroenterology 2012; 18 (33): 4507-4516.
130. Huang, Q., Fang, D. C., Fang, J. Y., Chen, M. H., Zhang, J., Lin, L., Dai, N., Yu, C. G., and Zhang, H. J. How to diagnose and manage patients with Barrett's esophagus in China. Journal of Digestive Diseases 2012; 13 (3): 123-132.
131. Fisichella, P. M., Carter, S. R., and Robles, L. Y. Presentation, diagnosis, and treatment of oesophageal motility disorders. Digestive and Liver Disease 2012; 44 (1): 1-7.
132. Li, Z. and Rice, T. W. Diagnosis and Staging of Cancer of the Esophagus and Esophagogastric Junction. Surgical Clinics of North America 2012; 92 (5): 1105-1126.
133. Goh, K. Gastroesophageal Reflux Disease in Asia: A historical perspective and present challenges. Journal of Gastroenterology and Hepatology (Australia) 2011; 26 (SUPPL. 1): 2-10.
134. Fock, K. M. and Ang, T. L. Global epidemiology of Barrett's esophagus. Expert Review of Gastroenterology and Hepatology 2011; 5 (1): 123-130.
135. Fang, D. C., Lin, S. R., Huang, Q., Yu, Z. L., Yuan, Y. Z., Chen, M. H., Bai, W. Y., Chen, X. X., Zhang, J., Li, Y. Q., Zhou, L. Y., Ke, M. Y., Fang, X. C., and Lan, Y. Chinese National Consensus on diagnosis and management of Barrett's esophagus (BE): Revised edition, June 2011, Chongqing, China. Journal of Digestive Diseases 2011; 12 (6): 415-419.
136. Di, Pietro M. Barretts esophagus and reflux disease. Endoscopy 2010; 42 (11): 910-915.
137. Bisschops, R. and Demedts, I. Reflux and Barrett's disease. Endoscopy 2009; 41 (1): 42-45.
138. Niv, Y. Capsule endoscopy: No longer limited to the small bowel. Israel Medical Association Journal 2010; 12 (3): 178-180.
139. Madani, A., Sowerby, L., Gregor, J. C., Wong, E., and Fung, K. Detecting the other reflux disease. Journal of Family Practice 2010; 59 (2): 102-107.
140. Muto, M., Horimatsu, T., Ezoe, Y., Hori, K., Yukawa, Y., Morita, S., Miyamoto, S., and Chiba, T. Narrow-band imaging of the gastrointestinal tract. Journal of gastroenterology 2009; 44 (1): 13-25.
141. Herszenyi, L., Pregun, I., and Tulassay, Z. Diagnosis and recognition of early esophageal neoplasia. Digestive Diseases 2009; 27 (1): 24-30.
142. Lenglinger, J., Izay, B., Eisler, M., Wrba, F., Zacherl, J., Prager, G., and Riegler, F. M. Barrett's esophagus: Size of the problem and diagnostic value of a novel histopathology classification. European Surgery - Acta Chirurgica Austriaca 2009; 41 (1): 26-39. \
143. Westerhof, J., Koornstra, J. J., and Weersma, R. K. Capsule endoscopy: A review from the clinician's perspectives. Minerva Gastroenterologica e Dietologica 2008; 54 (2): 189-207.
144. Reddymasu, S. C. and Sharma, P. Advances in Endoscopic Imaging of the Esophagus. Gastroenterology Clinics of North America 2008; 37 (4): 763-774.
145. Mathus-Vliegen, E. M. H. The role of endoscopy in bariatric surgery. Best Practice and Research: Clinical Gastroenterology 2008; 22 (5): 839-864.
146. Nakamura, T. and Terano, A. Capsule endoscopy: Past, present, and future. Journal of gastroenterology 2008; 43 (2): 93-99.
147. Bittinger, M. and Messmann, H. Gastroesophageal reflux disease and Barrett's esophagus. Endoscopy 2007; 39 (2): 118-123.
148. Shapiro, M., Moore, A., and Fass, R. Refractory gastroesophageal reflux disease - What next?. Journal of Respiratory Diseases 2007; 28 (10): 427-435.
149. Ponsot, P. Barrett's oesophagus: Endoscopic diagnosis and follow-up. Annales de chirurgie 2006; 131 (1): 3-6.
150. Canon, C. L., Morgan, D. E., Einstein, D. M., Herts, B. R., Hawn, M. T., and Johnson, L. F. Surgical approach to gastroesophageal reflux disease: What the radiologist needs to know. Radiographics 2005; 25 (6): 1485-1499.
151. Saadi, A. and Fitzgerald, R. C. Mechanisms underlying the progression of Barrett's oesophagus. Drug Discovery Today: Disease Mechanisms 2006; 3 (4): 447-456.
152. Camilleri, M. Gastroenterology and Hepatology Clinical Research Update: 2005-2006. Clinical Gastroenterology and Hepatology 2006; 4 (12): 1428-1433.
153. Kiesslich, R. and Neurath, M. F. Magnifying chromoendoscopy for the detection of premalignant gastrointestinal lesions. Best Practice and Research: Clinical Gastroenterology 2006; 20 (1): 59-78.
154. Bergman, J. J. G. H. Latest developments in the endoscopic management of gastroesophageal reflux disease and Barrett's esophagus: An overview of the year's literature. Endoscopy 2006; 38 (2): 122-132.
155. Lambert, R. Upper gastrointestinal tumors. Endoscopy 2006; 38 (2): 133-136.
156. Takubo, K., Arai, T., Sawabe, M., Iwakiri, K., and Vieth, M. Columnar-lined esophagus, Barrett's esophagus and adenocarcinoma: Differences between east and west. Digestive Endoscopy 2006; 18 (SUPPL. 1): S16-S20.
157. Hongo, M. Review article: Barrett's oesophagus and carcinoma in Japan. Alimentary Pharmacology and Therapeutics, Supplement 2004; 20 (8): 50-54.
158. Mariani, G., Boni, G., Barreca, M., Bellini, M., Fattori, B., Alsharif, A., Grosso, M., Stasi, C., Costa, F., Anselmino, M., Marchi, S., Rubello, D., and Strauss, H. W. Radionuclide gastroesophageal motor studies. Journal of Nuclear Medicine 2004; 45 (6): 1004-1028.
159. Conio, M., Lapertosa, G., Blanchi, S., and Filiberti, R. Barrett's esophagus: An update. Critical Reviews in Oncology/Hematology 2003; 46 (2): 187-206.
160. Rosch, T. DDW Reports 2003 Orlando: Reflux disease and Barrett's esophagus. Endoscopy 2003; 35 (10): 809-815.
161. Koop, H. Gastroesophageal reflux disease and Barrett's esophagus. Endoscopy 2002; 34 (2): 97-103.
162. Cameron, A. J. Epidemiology of Barrett's esophagus and adenocarcinoma. Diseases of the Esophagus 2002; 15 (2): 106-108.
163. Moreto, M. Diagnosis of esophagogastric tumors. Endoscopy 2001; 33 (1): 1-7.
164. DeMeester, S. R. and DeMeester, T. R. Columnar mucosa and intestinal metaplasia of the esophagus: Fifty years of controversy. Annals of Surgery 2000; 231 (3): 303-321.
165. Rosch, T. Gastroesophageal reflux disease and Barrett's esophagus. Endoscopy 2000; 32 (11): 826-835.
166. Cittadini, G., Sardanelli, F., and De, Cicco E. State-of-the-art barium examination of gastroesophageal reflux disease. Gastroenterology International 1997; 10 (SUPPL. 2): 18-24.
167. Katzka, D. A. and Castell, D. O. Barrett's esophagus: Management and surveillance. Article seven in the series. Practical Gastroenterology 1996; 20 (1): 24-35.
168. Jung, Hye Kyung Epidemiology of gastroesophageal reflux disease in Asia: a systematic review. Journal of neurogastroenterology and motility 2011; 17 (1): 14-27.
169. ASGE Standards of Practice Committee, Muthusamy, V. Raman, Lightdale, Jenifer R., Acosta, Ruben D., Chandrasekhara, Vinay, Chathadi, Krishnavel V., Eloubeidi, Mohamad A., Fanelli, Robert D., Fonkalsrud, Lisa, Faulx, Ashley L., Khashab, Mouen A., Saltzman, John R., Shaukat, Aasma, Wang, Amy, Cash, Brooks, and DeWitt, John M. The role of endoscopy in the management of GERD. Gastrointestinal endoscopy 2015; 81 (6): 1305-1310.
170. De Palma, Giovanni D. and Forestieri, Pietro Role of endoscopy in the bariatric surgery of patients. World journal of gastroenterology 2014; 20 (24): 7777-7784.
171. Spechler, Stuart Jon Barrett esophagus and risk of esophageal cancer: a clinical review. JAMA 2013; 310 (6): 627-636.
172. ASGE Standards of Practice Committee, Sharaf, Ravi N., Shergill, Amandeep K., Odze, Robert D., Krinsky, Mary L., Fukami, Norio, Jain, Rajeev, Appalaneni, Vasundhara, Anderson, Michelle A., Ben-Menachem, Tamir, Chandrasekhara, Vinay, Chathadi, Krishnavel, Decker, G. Anton, Early, Dana, Evans, John A., Fanelli, Robert D., Fisher, Deborah A., Fisher, Laurel R., Foley, Kimberly Q., Hwang, Joo Ha, Jue, Terry L., Ikenberry, Steven O., Khan, Khalid M., Lightdale, Jennifer, Malpas, Phyllis M., Maple, John T., Pasha, Shabana, Saltzman, John, Dominitz, Jason A., and Cash, Brooks D. Endoscopic mucosal tissue sampling. Gastrointestinal endoscopy 2013; 78 (2): 216-224.
173. Shaheen, Nicholas J., Weinberg, David S., Denberg, Thomas D., Chou, Roger, Qaseem, Amir, Shekelle, Paul, and Clinical Guidelines Committee of the American College of Physicians Upper endoscopy for gastroesophageal reflux disease: best practice advice from the clinical guidelines committee of the American College of Physicians. Annals of internal medicine 2012; 157 (11): 808-816.
174. Taylor, Justin B. and Rubenstein, Joel H. Meta-analyses of the effect of symptoms of gastroesophageal reflux on the risk of Barrett's esophagus. The American journal of gastroenterology 2010; 105 (8): 1729-1738.
175. Shaheen, Nicholas and Ransohoff, David F. Gastroesophageal reflux, barrett esophagus, and esophageal cancer: scientific review. JAMA 2002; 287 (15): 1972-1981.
176. Fiocca, Roberto, Mastracci, Luca, Milione, Massimo, Parente, Paola, Savarino, Vincenzo, Gruppo Italiano Patologi Apparato Digerente (GIPAD), and Societa Italiana di Anatomia Patologica e Citopatologia Diagnostica/International Academy of Pathology, Italian division SIAPEC IAP Microscopic esophagitis and Barrett's esophagus: the histology report. Digestive and liver disease 2011; 43 Suppl 4: S319-S330.
177. Barbiere, Josephine M. and Lyratzopoulos, Georgios Cost-effectiveness of endoscopic screening followed by surveillance for Barrett's esophagus: a review. Gastroenterology 2009; 137 (6): 1869-1876.
178. Lin, San Ren, Xu, Guo Ming, Hu, Pin Jin, Zhou, Li Ya, Chen, Min Hu, Ke, Mei Yun, Yuan, Yao Zong, Fang, Dian Chun, Xiao, Shu Dong, and Chinese National Consensus Workshop on GERD Chinese consensus on gastroesophageal reflux disease (GERD): October 2006, Sanya, Hainan Province, China. Journal of digestive diseases 2007; 8 (3): 162-169.
179. Fullard, M., Kang, J. Y., Neild, P., Poullis, A., and Maxwell, J. D. Systematic review: does gastro-oesophageal reflux disease progress? Alimentary pharmacology & therapeutics 2006; 24 (1): 33-45.
180. Kang, J. Y. Systematic review: geographical and ethnic differences in gastro-oesophageal reflux disease. Alimentary pharmacology & therapeutics 2004; 20 (7): 705-717.
181. Sampliner, Richard E. and Practice Parameters Committee of the American College of Gastroenterology Updated guidelines for the diagnosis, surveillance, and therapy of Barrett's esophagus. The American journal of gastroenterology 2002; 97 (8): 1888-1895.
182. Shaheen, Nicholas J., Provenzale, Dawn, and Sandler, Robert S. Upper endoscopy as a screening and surveillance tool in esophageal adenocarcinoma: a review of the evidence. The American journal of gastroenterology 2002; 97 (6): 1319-1327.
183. Moraes-Filho, JoaquimPradoP, Cecconello, Ivan, Gama-Rodrigues, Joaquim, Castro, LuizdePaula, Henry, Maria Aparecida, Meneghelli, Ulisses G., Quigley, Eamonn, and Brazilian Consensus Group Brazilian consensus on gastroesophageal reflux disease: proposals for assessment, classification, and management. The American journal of gastroenterology 2002; 97 (2): 241-248.
184. Ofman, J. J., Shaheen, N. J., Desai, A. A., Moody, B., Bozymski, E. M., and Weinstein, W. M. The quality of care in Barrett's esophagus: endoscopist and pathologist practices. The American journal of gastroenterology 2001; 96 (3): 876-881.
185. Sampliner, R. E. Practice guidelines on the diagnosis, surveillance, and therapy of Barrett's esophagus. The Practice Parameters Committee of the American College of Gastroenterology. The American journal of gastroenterology 1998; 93 (7): 1028-1032.
186. Anderson, W. D., Strayer, S. M., and Mull, S. R. Common questions about the management of gastroesophageal reflux disease. American Family Physician 2015; 91 (10): 692-697.
187. Bouchard, S., Ibrahim, M., and Van, Gossum A. Video capsule endoscopy: Perspectives of a revolutionary technique. World journal of gastroenterology 2014; 20 (46): 17330-17344.
188. Yang, X.-B. and Yu, L.-F. Clinical outcomes and endoscopic surveillance of gastroesophageal reflux disease: A review. International Journal of Clinical and Experimental Medicine 2016; 9 (2): 682-691.
189. Rai, T., Vennalaganti, P., and Sharma, P. Role of endoscopy in gastroesophageal reflux disease. Journal of Digestive Endoscopy 2015; 6 (3): 89-95.
190. Dunbar, K. B. and Spechler, S. J. Controversies in Barrett esophagus. Mayo Clinic Proceedings 2014; 89 (7): 973-984.
191. Hawkshaw, M. J., Sataloff, J. B., and Sataloff, R. T. New concepts in vocal fold imaging: A review. Journal of Voice 2013; 27 (6): 738-743.
192. Di, Pietro M. and Fitzgerald, R. C. Research advances in esophageal diseases: Bench to bedside. F1000Prime Reports 2013; 5:44 (doi:10.12703/P5-44).
193. Parasa, S. and Sharma, P. Complications of gastro-oesophageal reflux disease. Best Practice and Research: Clinical Gastroenterology 2013; 27 (3): 433-442.
194. Dent, J., Becher, A., Sung, J., Zou, D., Agreus, L., and Bazzoli, F. Systematic Review: Patterns of Reflux-Induced Symptoms and Esophageal Endoscopic Findings in Large-Scale Surveys. Clinical Gastroenterology and Hepatology 2012; 10 (8): 863-873.
195. Choi, S. E. and Hur, C. Screening and surveillance for Barrett's esophagus: Current issues and future directions. Current opinion in gastroenterology 2012; 28 (4): 377-381.
196. Qumseya, B. J., Wolfsen, C. L., and Wolfsen, H. C. Reflux disease and Barretts esophagus. Endoscopy 2011; 43 (11): 962-965.
197. Fedeli, P., Gasbarrini, A., and Cammarota, G. Spectral endoscopic imaging: The multiband system for enhancing the endoscopic surface visualization. Journal of clinical gastroenterology 2011; 45 (1): 6-15.
198. Odze, R. D. Update on the diagnosis and treatment of Barrett esophagus and related neoplastic precursor lesions. Archives of Pathology and Laboratory Medicine 2008; 132 (10): 1577-1585.
199. Dilemmas in managing Barrett's oesophagus. Drug and Therapeutics Bulletin 2006; 44 (9): 69-72.
200. Katz, P. O. Gastroesophageal reflux disease symptoms on antisecretory therapy: Acid, non-acid, or no GERD. Reviews in gastroenterological disorders 2006; 6 (3): 136-145.
201. Rice, T. W., Mendelin, J. E., and Goldblum, J. R. Barrett's esophagus: Pathologic considerations and implications for treatment. Seminars in Thoracic and Cardiovascular Surgery 2006; 17 (4): 292-300.
202. Schneider, H. R. CPD: Is a proton pump inhibitor (PPI) the GP's gastroscopy?. South African Family Practice 2005; 47 (2): 24-29.
203. Sampliner, R. E. Epidemiology, pathophysiology, and treatment of Barrett's esophagus: Reducing mortality from esophageal adenocarcinoma. Medical Clinics of North America 2005; 89 (2): 293-312.
204. Koop, H. Gastroesophageal reflux disease and Barrett's esophagus. Endoscopy 2004; 36 (2): 103-109.
205. Bonino, J. A. and Sharma, P. Barrett esophagus. Current opinion in gastroenterology 2004; 20 (4): 375-380.
206. Pisegna, J., Holtmann, G., Howden, C. W., Katelaris, P. H., Sharma, P., Spechler, S., Triadafilopoulos, G., and Tytgat, G. Review article: Oesophageal complications and consequences of persistent gastro-oesophageal reflux disease. Alimentary Pharmacology and Therapeutics, Supplement 2004; 20 (9): 47-56.
207. Devault, K. R. and Castell, D. O. Updated guidelines for the diagnosis and treatment of gastroesophageal reflux disease. American Journal of Gastroenterology 1999; 94 (6): 1434-1442.
208. Freston, J. W., Malagelada, J. R., Petersen, H., and McCloy, R. F. Critical issues in the management of gastroesophageal reflux disease. European Journal of Gastroenterology and Hepatology 1995; 7 (6): 577-586.
209. Rosch, T. and Allescher, H. D. Congress report on the Digestive Diseases Week 1994 in New Orleans: Endoscopic abstracts. Endoscopy 1994; 26 (7): 635-658.
210. Yerian, Lisa Histology of metaplasia and dysplasia in Barrett's esophagus. Surgical oncology clinics of North America 2009; 18 (3): 411-422.
211. Golger, D., Probst, A., and Messmann, H. Barrett's esophagus: Lessons from recent clinical trials. Annals of Gastroenterology 2016; 29 (4): 417-423.
212. Recommendations for clinical practice. Gastroesophageal reflux in adults: Diagnosis and treatment. Journal Medical Libanais 1999; 47 (4): 238-242.
213. Fennerty, M. B., Castell, D., Fendrick, A. M., Halpern, M., Johnson, D., Kahrilas, P. J., Lieberman, D., Richter, J. E., and Sampliner, R. E. The diagnosis and treatment of gastroesophageal reflux disease in a managed care environment: Suggested disease management guidelines. Archives of Internal Medicine 1996; 156 (5): 477-484.
214. Carlson, D. A., Hinchcliff, M., and Pandolfino, J. E. Advances in the Evaluation and Management of Esophageal Disease of Systemic Sclerosis. Current Rheumatology Reports 2015; 17(1): 475.
215. Alsop, B. R. and Sharma, P. Esophageal Cancer. Gastroenterology Clinics of North America 2016; 45 (3): 399-412.
216. Galmiche, J.-P. and Des Varannes, S. B. Symptoms and disease severity in gastro-oesophageal reflux disease. Scandinavian Journal of Gastroenterology, Supplement 1994; 29 (201): 62-68.
217. Segal, Fabio and Breyer, Helenice Pankowski Diagnosis and management of Barrett's metaplasia: What's new. World journal of gastrointestinal endoscopy 2012; 4 (9): 379-386.
218. Davis-Yadley, Ashley H., Neill, Kevin G., Malafa, Mokenge P., and Pena, Luis R. Advances in the Endoscopic Diagnosis of Barrett Esophagus. Cancer control : journal of the Moffitt Cancer Center 2016; 23 (1): 67-77.
219. Raiser, F., Hinder, R. A., McBride, P. J., Katada, N., and Filipi, C. J. The technique of laparoscopic Nissen fundoplication. Chest surgery clinics of North America 1995; 5 (3): 437-448.
220. van Baal, J. W. P. M. and Krishnadath, K. K. High throughput techniques for characterizing the expression profile of Barrett's esophagus. Diseases of the Esophagus 2008; 21 (7): 634-640.
221. Cockeram A. Canadian Association of Gastroenterology Practice Guideline for clinical competence in diagnostic and therapeutic endoscopic retrograde cholangiopancreatography. Canadian Journal of Gastroenterology and Hepatology. 1997;11(6):535-8.
222. Springer J, Enns R, Romagnuolo J, Ponich T, Barkun AN, Armstrong D. Canadian credentialing guidelines for endoscopic retrograde cholangiopancreatography. Canadian Journal of Gastroenterology and Hepatology. 2008;22(6):547-51.
223. Armstrong D, Barkun A, Bridges R, Carter R, De Gara C, Dubé C, Enns R, Hollingworth R, MacIntosh D, Borgaonkar M, Forget S. Canadian Association of Gastroenterology consensus guidelines on safety and quality indicators in endoscopy. Canadian Journal of Gastroenterology and Hepatology. 2012;26(1):17-31.
224. Ponich T, Enns R, Romagnuolo J, Springer J, Armstrong D, Barkun AN. Canadian credentialing guidelines for esophagogastroduodenoscopy. Canadian Journal of Gastroenterology and Hepatology. 2008;22(4):349-54.
225. Cockeram AW. Canadian association of gastroenterology practice guidelines: evaluation of dysphagia. Canadian Journal of Gastroenterology and Hepatology. 1998;12(6):409-13.
226. Armstrong D, Marshall JK, Chiba N, Enns R, Fallone CA, Fass R, Hollingworth R, Hunt RH, Kahrilas PJ, Mayrand S, Moayyedi P. Canadian Consensus Conference on the management of gastroesophageal reflux disease in adults–update 2004. Canadian Journal of Gastroenterology and Hepatology. 2005;19(1):15-35.
227. Thomson AB, Chiba N, Armstrong D, Tougas G, Hunt RH. The second Canadian gastroesophageal reflux disease consensus: moving forward to new concepts. Canadian Journal of Gastroenterology and Hepatology. 1998;12(8):551-6.
228. Petersen BT, Chennat J, Cohen J, Cotton PB, Greenwald DA, Kowalski TE, Krinsky ML, Park WG, Pike IM, Romagnuolo J, Rutala WA. Multisociety guideline on reprocessing flexible gastrointestinal endoscopes: 2011. Gastrointestinal endoscopy. 2011 Jun 1;73(6):1075-84.
229. Shergill AK, Ben-Menachem T, Chandrasekhara V, Chathadi K, Decker GA, Evans JA, Early DS, Fanelli RD, Fisher DA, Foley KQ, Fukami N. Guidelines for endoscopy in pregnant and lactating women. Gastrointestinal endoscopy. 2012 Jul 1;76(1):18-24.
230. Early DS, Acosta RD, Chandrasekhara V, Chathadi KV, Decker GA, Evans JA, Fanelli RD, Fisher DA, Foley KQ, Fonkalsrud L, Hwang JH. Modifications in endoscopic practice for the elderly. Gastrointestinal endoscopy. 2013 Jul 1;78(1):1-7.
231. Muthusamy VR, Lightdale JR, Acosta RD, Chandrasekhara V, Chathadi KV, Eloubeidi MA, Fanelli RD, Fonkalsrud L, Faulx AL, Khashab MA, Saltzman JR. The role of endoscopy in the management of GERD. Gastrointestinal endoscopy. 2015 Jun 1;81(6):1305-10.
232. Pasha SF, Acosta RD, Chandrasekhara V, Chathadi KV, Decker GA, Early DS, Evans JA, Fanelli RD, Fisher DA, Foley KQ, Fonkalsrud L. The role of endoscopy in the evaluation and management of dysphagia. Gastrointestinal endoscopy. 2014 Feb 1;79(2):191-201.
233. Evans JA, Early DS, Chandraskhara V, Chathadi KV, Fanelli RD, Fisher DA, Foley KQ, Hwang JH, Jue TL, Pasha SF, Sharaf R. The role of endoscopy in the assessment and treatment of esophageal cancer. Gastrointestinal endoscopy. 2013 Mar 1;77(3):328-34
234. Evans JA, Early DS, Fukami N, Ben-Menachem T, Chandrasekhara V, Chathadi KV, Decker GA, Fanelli RD, Fisher DA, Foley KQ, Hwang JH. The role of endoscopy in Barrett's esophagus and other premalignant conditions of the esophagus. Gastrointestinal endoscopy. 2012 Dec 1;76(6):1087-94.
235. Ben-Menachem T, Decker GA, Early DS, Evans J, Fanelli RD, Fisher DA, Fisher L, Fukami N, Hwang JH, Ikenberry SO, Jain R. Adverse events of upper GI endoscopy. Gastrointestinal endoscopy. 2012 Oct 1;76(4):707-18.
236. Early DS, Ben-Menachem T, Decker GA, Evans JA, Fanelli RD, Fisher DA, Fukami N, Hwang JH, Jain R, Jue TL, Khan KM. Appropriate use of GI endoscopy. Gastrointestinal endoscopy. 2012 Jun 1;75(6):1127-31.
237. Parsi MA, Sullivan SA, Goodman A, Manfredi M, Navaneethan U, Pannala R, Smith ZL, Thosani N, Banerjee S, Maple JT. Automated endoscope reprocessors. Gastrointestinal endoscopy. 2016 Dec 1;84(6):885-92.
238. Lo SK, Fujii-Lau LL, Enestvedt BK, Hwang JH, Konda V, Manfredi MA, Maple JT, Murad FM, Pannala R, Woods KL, Banerjee S. The use of carbon dioxide in gastrointestinal endoscopy. Gastrointestinal endoscopy. 2016 May 1;83(5):857-65.
239. Varadarajulu S, Banerjee S, Barth BA, Desilets DJ, Kaul V, Kethu SR, Pedrosa MC, Pfau PR, Tokar JL, Wang A, Song LM. GI endoscopes. Gastrointestinal endoscopy. 2011 Jul 31;74(1):1-6.
240. Nelson DB, Barkun AN, Block KP, Burdick JS, Ginsberg GG, Greenwald DA, Kelsey PB, Nakao NL, Slivka A, Smith P, Vakil N. Transmission of infection by gastrointestional endoscopy. Gastrointestinal endoscopy. 2001 Dec 1;54(6):824-8.
241. Nelson DB, Barkun AN, Block KP, Burdick JS, Ginsberg GG, Greenwald DA, Kelsey PB, Nakao NL, Slivka A, Smith P, Vakil N. Transmission of infection by gastrointestional endoscopy. Gastrointestinal endoscopy. 2001 Dec 1;54(6):824-8.
242. Gottlieb KT, Banerjee S, Barth BA, Bhat YM, Desilets DJ, Maple JT, Pfau PR, Pleskow DK, Siddiqui UD, Tokar JL, Wang A. Monitoring equipment for endoscopy. Gastrointestinal endoscopy. 2013 Feb 1;77(2):175-80.
243. Rodriguez SA, Banerjee S, Desilets D, Diehl DL, Farraye FA, Kaul V, Kwon RS, Mamula P, Pedrosa MC, Varadarajulu S, Song LM. Ultrathin endoscopes. Gastrointestinal endoscopy. 2010 May 1;71(6):893-8.
244. Tierney WM, Adler DG, Conway JD, Diehl DL, Farraye FA, Kantsevoy SV, Kaul V, Kethu SR, Kwon RS, Mamula P, Pedrosa MC. Overtube use in gastrointestinal endoscopy. Gastrointestinal endoscopy. 2009 Nov 1;70(5):828-34.
245. Somogyi L, Chuttani R, Croffie J, DiSario J, Liu J, Mishkin D, Shah R, Tierney W, Song LM, Petersen BT. Guidewires for use in GI endoscopy. Gastrointestinal endoscopy. 2007 Apr 1;65(4):571-6.
246. Manfredi MA, Dayyeh BK, Bhat YM, Chauhan SS, Gottlieb KT, Hwang JH, Komanduri S, Konda V, Lo SK, Maple JT, Murad FM. Electronic chromoendoscopy. Gastrointestinal endoscopy. 2015 Feb 1;81(2):249-61.
247. Song LM, Adler DG, Chand B, Conway JD, Croffie JM, DiSario JA, Mishkin DS, Shah RJ, Somogyi L, Tierney WM, Petersen BT. Chromoendoscopy. Gastrointestinal endoscopy. 2007 Oct 1;66(4):639-49.
248. Sharma P, Savides TJ, Canto MI, Corley DA, Falk GW, Goldblum JR, Wang KK, Wallace MB, Wolfsen HC. The American Society for Gastrointestinal Endoscopy PIVI (preservation and incorporation of valuable endoscopic innovations) on imaging in Barrett's esophagus (Long form). Retrieved from <http://old.asge.org/assets/0/71542/76654/08c53485-c9ef-4e19-9275-60cd045a0870.pdf>
249. Sharma P, Savides TJ, Canto MI, Corley DA, Falk GW, Goldblum JR, Wang KK, Wallace MB, Wolfsen HC. The American Society for Gastrointestinal Endoscopy PIVI (preservation and incorporation of valuable endoscopic innovations) on imaging in Barrett's esophagus. Gastrointestinal endoscopy. 2012 Aug 1;76(2):252-4.
250. Shaheen NJ, Falk GW, Iyer PG, Gerson LB. ACG clinical guideline: diagnosis and management of Barrett's esophagus. The American journal of gastroenterology. 2016 Jan 1;111(1):30.
251. DeVault KR, Castell DO. Updated guidelines for the diagnosis and treatment of gastroesophageal reflux disease. The American journal of gastroenterology. 2005 Jan 1;100(1):190.
252. American Gastroenterological Association. (2011). American Gastroenterological Association medical position statement on the management of Barrett's esophagus. Gastroenterology, 140(3), 1084-1091.
253. Spechler SJ, Sharma P, Souza RF, Inadomi JM, Shaheen NJ. American Gastroenterological Association technical review on the management of Barrett's esophagus. Gastroenterology. 2011 Mar;140(3):e18.
254. Kahrilas PJ, Shaheen NJ, Vaezi MF. American Gastroenterological Association Medical Position Statement on the management of gastroesophageal reflux disease. Gastroenterology. 2008 Oct 31;135(4):1383-91.
255. Kahrilas, P. J., Shaheen, N. J., & Vaezi, M. F. (2008). American Gastroenterological Association Institute technical review on the management of gastroesophageal reflux disease. Gastroenterology, 135(4), 1392-1413.
256. Yang YX, Brill J, Krishnan P, Leontiadis G, Adams MA, Dorn SD, Dudley-Brown SL, Flamm SL, Gellad ZF, Gruss CB, Kosinski LR. American Gastroenterological Association Institute guideline on the role of upper gastrointestinal biopsy to evaluate dyspepsia in the adult patient in the absence of visible mucosal lesions. Gastroenterology. 2015 Oct 1;149(4):1082-7.
257. Allen JI, Katzka D, Robert M, Leontiadis GI. American Gastroenterological Association Institute technical review on the role of upper gastrointestinal biopsy to evaluate dyspepsia in the adult patient in the absence of visible mucosal lesions. Gastroenterology. 2015 Oct 1;149(4):1088-118.
258. Forgacs I, Ashton R, Allum W, Bowley T, Brown H, Coleman MP, Fitzgerald R, Glynn M, Hiom S, Jones R, Machesney M. Conference report: improving outcomes for gastrointestinal cancer in the UK. Frontline Gastroenterology. 2016 Jun 14:flgastro-2016.
259. Allum WH, Blazeby JM, Griffin SM, Cunningham D, Jankowski JA, Wong R. Guidelines for the management of oesophageal and gastric cancer. Gut. 2011 Jan 1:gut-2010.
260. National Collaborating Centre for Women's and Children's Health. UK. Gastro-Oesophageal Reflux Disease: Recognition, Diagnosis and Management in Children and Young People. London: National Institute for Health and Care Excellence (UK); 2015.  <https://www.ncbi.nlm.nih.gov/pubmed/25950074>
261. Fitzgerald RC, di Pietro M, Ragunath K, Ang Y, Kang JY, Watson P, Trudgill N, Patel P, Kaye PV, Sanders S, O'donovan M. British Society of Gastroenterology guidelines on the diagnosis and management of Barrett's oesophagus. Gut. 2013 Oct 26:gutjnl-2013.
262. Bennett C, Vakil N, Bergman J, Harrison R, Odze R, Vieth M, Sanders S, Gay L, Pech O, Longcroft–Wheaton G, Romero Y. Consensus statements for management of Barrett's dysplasia and early-stage esophageal adenocarcinoma, based on a Delphi process. Gastroenterology. 2012 Aug 31;143(2):336-46.
263. Green J. Complications of gastrointestinal endoscopy. BSG Guidelines in Gastroenterology. 2006:1-30.
264. National Institute for Health and Care Excellence. Dyspepsia and gastro-oesophageal reflux disease in adults. Quality standard Published: 23 July 2015. <http://www/nice.org.uk/guidance/qs96>
265. National Institute for Health and Care Excellence. Gastro-oesophageal reflux in children and young people. Quality standard Published: Published: 28 January 2016 <http://www.nice.org.uk/guidance/qs112>
266. National OesophagoGastric Cancer Audit . An audit of the care received by people with Oesophago-Gastric Cancer in England and Wales 2016 Annual Report. Retrieved from <http://content.digital.nhs.uk/og>
267. Palser T, Cromwell D, Meulen van der J, Hardwick RH, Riley S, Greenaway K, Dean S: The National Oesophago-Gastric Cancer Audit. An audit of the care received by people with Oesophago-gastric Cancer in England and Wales. First Annual Report 2008. 2008, London: NHS Information Centre Retrieved from <http://content.digital.nhs.uk/og>
268. Shaheen NJ, Weinberg DS, Denberg TD, Chou R, Qaseem A, Shekelle P. Upper endoscopy for gastroesophageal reflux disease: best practice advice from the clinical guidelines committee of the American College of Physicians. Annals of internal medicine. 2012 Dec 4;157(11):808-16.
269. Recommendation AC. Upper Endoscopy for Gastroesophageal Reflux: Review of the Performance Measures by the Performance Measurement Committee of the American College of Physicians. Retrieved from, <https://www.acponline.org/system/files/documents/clinical_information/performance_measurement/measures/pmc_gerd_review.pdf>
270. Gilbert R, Devries-Aboud M, Winquist E, Waldron J, McQuestion M. The management of head and neck cancer in Ontario. Cancer Care Ontario; 2009 Dec 15. Retrieved from <https://www.cancercare.on.ca/common/pages/UserFile.aspx?fileId=58592>
271. Canadian Agency for Drugs and Technologies in Health (CADTH). Prioritization of Patients Requiring Endoscopy Procedures: Clinical Evidence and Guidelines. Rapid response report: Summary of Abstracts. Prioritization of Patients Requiring Endoscopy Procedures, 2011. Retrieved from <https://cadth.ca/sites/default/files/pdf/htis/dec-2011/RB0454-000%20Endoscopy%20Prioritization.pdf>
272. Hailey D. Endoscope-based treatments for gastroesophageal reflux disease. Issues in emerging health technologies. 2004 Mar(54):1-4.
273. Topfer, LA.The Cytosponge: an alternative to endoscopy in detecting Barrett esophagus. Ottawa: CADTH; 2015 Oct. (CADTH Issues in Emerging Health Technologies; Issue 144)
274. Lynn M. Brodsky. Wireless Capsule Endoscopy. Issues in Emerging Health Technologies, Issue 53 — December 2003. Retrieved from <https://www.cadth.ca/wireless-capsule-endoscopy-0>
275. Medical Advisory Secretariat. Wireless capsule endoscopy: an evidence­based analysis. Ontario Health Technology Assessment Series 2003;3(2).
276. Julie Tranchemontagne. Summary: Initial Staging of Esophageal Cancer: Systematic review of the performance of diagnostic methods. Extract from the report: Initial Staging of Esophageal Cancer: Systematic Review of the Performance of Diagnotic Methods. prepared for AETMIS. Retrieved from <https://www.inesss.qc.ca/fileadmin/doc/AETMIS/Rapports/Cancer/2009_06_res_en.pdf>
277. Lerut T, Stordeur S, Verleye L, Vlayen J, Boterberg T, De Hertogh G, De Mey J, Deprez P, Flamen P, Pattyn P, Van Laethem J-L, Peeters M. Actualisation des recommandations cliniques pour le cancer de l’œsophage et de l’estomac. Good Clinical Practice (GCP). Bruxelles: Centre Fédéral d’Expertise des Soins de Santé (KCE). 2012. KCE Report 179B. D/2012/10.273/33. Retrieved from, <http://kce.fgov.be/fr/content/a-propos-du-copyright-des-rapports-kce>.
278. Vlayen J, De Gendt C, Stordeur S, Schillemans V, Camberlin C, Vrijens F, Van Eyck indicators for the management of upper gastrointestinal cancer. Good Clinical Practice (GCP) Health Care Knowledge Centre (KCE). 2013. KCE Reports 200. D/2013/10.273/15. Retrieved from, <http://kce.fgov.be/content/about-copyrights-for-kce-reports>
279. Indication à viseé diagnostique de l'endoscopie digestive haute en pathologie œso-gastro-duodénale de l'adulte à l'exclusion de l'échoendoscopie et l'entéroscopie, Retrieved from: https://www.has-sante.fr/portail/upload/docs/application/pdf/endoscopdigrap.pdf
280. National Collaborating Centre for Cancer (UK. Suspected cancer: recognition and referral. London: National Institute for Health and Care Excellence (UK); 2015 Jun.
281. National Collaborating Centre for Women's and Children's Health (UK. Gastro-Oesophageal Reflux Disease: Recognition, Diagnosis and Management in Children and Young People. London: National Institute for Health and Care Excellence (UK); 2015 Jan.Retrieved from, <https://www.ncbi.nlm.nih.gov/pubmed/25950074>
282. Liu, R., Kriz, H., Thielke, A., Vandegriff, S., & King, V. Upper Endoscopy for Gastroesophageal Reflux Disease (GERD) and Upper Gastrointestinal (GI) Symptoms. Portland, OR: Center for Evidence-based Policy, Oregon Health and Science University: 2012
283. Guidelines and Protocols Advisory committee. Gastroesophageal Reflux Disease – Clinical Approach in Adults. 2009
284. Alberta Health Services. Management of patients with early esophageal cancer, dysplastic and non-dysplastic Barrett’s Esophagus. Clinical Practice guideline GI-011; Version 2: 2014, Retrieved from <http://www.albertahealthservices.ca/assets/info/hp/cancer/if-hp-cancer-guide-gi011-barretts-esophagus.pdf>
285. Alberta Health Services. Gastric Cancer. Clinical Practice guideline GI-008; Version 4: 2016. Retrieved from <http://www.albertahealthservices.ca/assets/info/hp/cancer/if-hp-cancer-guide-gi008-gastric.pdf>
286. Family Practice Oncology Network. Upper Gastrointestinal Cancer – Part 1. BC Cancer Agency Care and Research, 2016. Retrieved from <http://www.bccancer.bc.ca/family-oncology-network-site/Documents/UpperGICancer-Part1-%20PublishCopy%20-%20April%202016.pdf>
287. Provincial Esophageal Cancer and Gastro-esophageal junction Cancer Treatment Guidelines. Saskatchewan Cancer Agency, 2011. Retrieved from <http://www.saskcancer.ca/Esophageal%20Guidelines>
288. Alberta Health Services. Esophageal Cancer. Clinical Practice guideline GI-009; Version 4. Retrieved from <http://www.albertahealthservices.ca/assets/info/hp/cancer/if-hp-cancer-guide-gi009-esophageal.pdf>
289. Stahl M, Budach W, Meyer HJ, Cervantes A, ESMO Guidelines Working Group. Esophageal cancer: Clinical Practice Guidelines for diagnosis, treatment and follow-up. Annals of Oncology. 2010 May 1;21(suppl_5):v46-9.
290. Jacobson BC, Hirota W, Baron TH, Leighton JA, Faigel DO, Standards of Practice Committee. The role of endoscopy in the assessment and treatment of esophageal cancer. Gastrointestinal endoscopy. 2003 Jun 30;57 (7):817-22.
291. AETNA. Oral and Esophageal Brush Biopsy. Policy No.0686. Retrieved from <http://www.aetna.com/cpb/medical/data/600_699/0686.html>
292. AETNA. Endoscopic Ultrasonography. Policy No.0446. Retrieved from <http://www.aetna.com/cpb/medical/data/400_499/0446.html>
293. AETNA. Virtual Gastrointestinal Endoscopy. Policy No.0535. Retrieved from <http://www.aetna.com/cpb/medical/data/500_599/0535.html>
294. AETNA. Upper Gastrointestinal Endoscopy. Policy No.0738. Retrieved from <http://www.aetna.com/cpb/medical/data/700_799/0738.html>
295. AETNA. Capsule Endoscopy. Policy No.0588. Retrieved from <http://www.aetna.com/cpb/medical/data/500_599/0588.html>
296. Sturgeon CM, Diamandis EP. Use of tumor markers in liver, bladder, cervical, and gastric cancers. Laboratory medicine practice guidelines. Washington: National Academy of Clinical Biochemistry. 2010. Retrieved from <https://www.aacc.org/science-and-practice/practice-guidelines/liver-tumor-markers>
297. French National Agency for Accreditation and Evaluation in Healthcare. Indications for lower gastrointestinal endoscopy (excluding population screening). Clinical practice guidelines, 2004. Retrieved from <https://www.has-sante.fr/portail/upload/docs/application/pdf/Endoscopy_guidelines.pdf>
298. French National Agency for Accreditation and Evaluation in Healthcare. . anaesdiagnostic indications for upper gastrointestinal endoscopy in oesophageal and gastroduodenal disease in adults, excluding endoscopic ultrasonography and enteroscopy. Guidelines department, 2001. Retrieved from <https://www.has-sante.fr/portail/upload/docs/application/pdf/gastro_endoscopy.pdf>
299. Heidelbaugh JJ, Harrison RV, McQuillan MA, Nostrant TT. Gastroesophageal Reflux Disease (GERD). Guidelines for Clinical Care Ambulatory, Updated 2012. Retrieved from <https://www.med.umich.edu/1info/FHP/practiceguides/gerd/gerd.12.pdf>
300. Katz PO, Gerson LB, Vela MF. Corrigendum: guidelines for the diagnosis and management of gastroesophageal reflux disease. The American journal of gastroenterology. 2013 Oct 1;108(10):1672.
301. Whiteman DC, Appleyard M, Bahin FF, Bobryshev YV, Bourke MJ, Brown I, Chung A, Clouston A, Dickins E, Emery J, Eslick GD. Australian clinical practice guidelines for the diagnosis and management of Barrett's esophagus and early esophageal adenocarcinoma. Journal of gastroenterology and hepatology. 2015 May 1;30(5):804-20.
302. Liu R, Kriz H, Thielke A, Vandegriff S, & King V. Upper endoscopy for gastroesophagealreflux disease (GERD) and upper gastrointestinal (GI) symptoms. Olympia: Washington State Health Authority Health Technology Assessment Program, 2013. Retrieved from <http://www.hta.hca.wa.gov/gerd.html>
303. Bechara R, Inoue H. Recent advancement of therapeutic endoscopy in the esophageal benign diseases. World journal of gastrointestinal endoscopy. 2015 May 16;7(5):481.
304. Ladas SD, Triantafyllou K, Spada CR, Riccioni ME, Rey JF, Niv Y, Delvaux M, De Franchis R, Costamagna G. European Society of Gastrointestinal Endoscopy (ESGE): recommendations (2009) on clinical use of video capsule endoscopy to investigate small-bowel, esophageal and colonic diseases. Endoscopy. 2010 Mar;42(03):220-7.
305. Cohen J, Pike IM. Defining and measuring quality in endoscopy. Gastrointestinal endoscopy. 2015 Jan 1;81(1):1-2.
306. Paterson WG, Depew WT, Paré P, Petrunia D, Switzer C, van Zanten SJ, Daniels S, Canadian Association of Gastroenterology Wait Time Consensus Group. Canadian consensus on medically acceptable wait times for digestive health care. Canadian Journal of Gastroenterology and Hepatology. 2006;20(6):411-23.
307. Enns R, Romagnuolo J, Ponich T, Springer J, Armstrong D, Barkun AN. Canadian credentialing guidelines for flexible sigmoidoscopy. Canadian Journal of Gastroenterology and Hepatology. 2008;22(2):115-9.
308. Enns RA, Hookey L, Armstrong D, Bernstein CN, Heitman SJ, Teshima C, Leontiadis GI, Tse F, Sadowski D. Clinical Practice Guidelines for the Use of Video Capsule Endoscopy. Gastroenterology. 2017 Feb 28;152(3):497-514.
309. Devlin TB. Canadian Association of Gastroenterology practice guidelines: Antibiotic prophylaxis for gastrointestinal endoscopy. Canadian Journal of Gastroenterology and Hepatology. 1999;13(10):819-21.
310. Beaulieu D, Barkun AN, Dubé C, Tinmouth J, Hallé P, Martel M. Endoscopy reporting standards. Canadian Journal of Gastroenterology and Hepatology. 2013;27(5):286-92.
311. Borgaonkar MR, Hookey L, Hollingworth R, Kuipers EJ, Forster A, Armstrong D, Barkun A, Bridges R, Carter R, De Gara C, Dube C. Indicators of safety compromise in gastrointestinal endoscopy. Canadian Journal of Gastroenterology and Hepatology. 2012;26(2):71-8.
312. Rizk MK, Sawhney MS, Cohen J, Pike IM, Adler DG, Dominitz JA, Lieb JG, Lieberman DA, Park WG, Shaheen NJ, Wani S. Quality indicators common to all GI endoscopic procedures. Gastrointestinal endoscopy. 2015 Jan 1;81(1):3-16.
313. Park WG, Shaheen NJ, Cohen J, Pike IM, Adler DG, Inadomi JM, Laine LA, Lieb JG, Rizk MK, Sawhney MS, Wani S. Quality indicators for EGD. Gastrointestinal endoscopy. 2015 Jan 1;81(1):17-30.
314. Adler DG, Lieb JG, Cohen J, Pike IM, Park WG, Rizk MK, Sawhney MS, Scheiman JM, Shaheen NJ, Sherman S, Wani S. Quality indicators for ERCP. Gastrointestinal endoscopy. 2015 Jan 1;81(1):54-66.
315. Wani S, Wallace MB, Cohen J, Pike IM, Adler DG, Kochman ML, Lieb JG, Park WG, Rizk MK, Sawhney MS, Shaheen NJ. Quality indicators for EUS. Gastrointestinal endoscopy. 2015 Jan 1;81(1):67-80.
316. Faigel DO, Pike IM, Baron TH, Chak A, Cohen J, Deal SE, Hoffman B, Jacobson BC, Mergener K, Petersen BT, Petrini JL. Quality indicators for gastrointestinal endoscopic procedures: an introduction. Gastrointestinal endoscopy. 2006 Apr 1;63(4):S3-9.
317. Cohen J, Safdi MA, Deal SE, Baron TH, Chak A, Hoffman B, Jacobson BC, Mergener K, Petersen BT, Petrini JL, Rex DK. Quality indicators for esophagogastroduodenoscopy. Gastrointestinal endoscopy. 2006 Apr 1;63(4):S10-5.
318. Baron TH, Petersen BT, Mergener K, Chak A, Cohen J, Deal SE, Hoffman B, Jacobson BC, Petrini JL, Safdi MA, Faigel DO. Quality indicators for endoscopic retrograde cholangiopancreatography. Gastrointestinal endoscopy. 2006 Apr 1;63(4):S29-34.
319. Jacobson BC, Chak A, Hoffman B, Baron TH, Cohen J, Deal SE, Mergener K, Petersen BT, Petrini JL, Safdi MA, Faigel DO. Quality indicators for endoscopic ultrasonography. Gastrointestinal endoscopy. 2006 Apr 1;63(4):S35-8.
320. Faigel DO, Baron TH, Lewis B, Petersen B, Petrini J. Ensuring competence in endoscopy. Prepared by the ASGE taskforce on ensuring competence in endoscopy and American College of Gastroenterology executive and practice management committees. ASGE policy and procedures manual for gastrointestinal endoscopy: guidelines for training and practice on CD-ROM. ASGE. 2005;136
321. Sedlack RE, Coyle WJ, Obstein KL, Al-Haddad MA, Bakis G, Christie JA, Davila RE, DeGregorio B, DiMaio CJ, Enestvedt BK, Jorgensen J. ASGE’s assessment of competency in endoscopy evaluation tools for colonoscopy and EGD. Gastrointestinal endoscopy 2014 Jan 1;79(1):1-7.
322. Murad FM, Komanduri S, Dayyeh BK, Chauhan SS, Enestvedt BK, Fujii-Lau LL, Konda V, Maple JT, Pannala R, Thosani NC, Banerjee S. Echoendoscopes. Gastrointestinal Endoscopy 2015 Aug 1;82(2):189-202.
323. Shaukat A, Wang A, Acosta RD, Bruining DH, Chandrasekhara V, Chathadi KV, Eloubeidi MA, Fanelli RD, Faulx AL, Fonkalsrud L, Gurudu SR. The role of endoscopy in dyspepsia. Gastrointestinal endoscopy. 2015 Aug 1;82(2):227-32.
324. van Vliet, E. P., Eijkemans, M. J., Kuipers, E. J., Poley, J. W., Steyerberg, E. W., and Siersema, P. D. Publication bias does not play a role in the reporting of the results of endoscopic ultrasound staging of upper gastrointestinal cancers. Endoscopy 2007; 39 (4): 325-332.
325. Yang, Dennis and Draganov, Peter V. Expanding Role of Third Space Endoscopy in the Management of Esophageal Diseases. Current treatment options in gastroenterology 2018. 16 (1) 41-57.
326. Munoz-Largacha, Juan A. and Litle, Virginia R. Endoscopic mucosal ablation and resection of Barrett's esophagus and related diseases. Journal of visualized surgery 2017. 3 () 128.
327. Mansour, Nabil M., El-Serag, Hashem B., and Anandasabapathy, Sharmila. Barrett's esophagus: best practices for treatment and post-treatment surveillance. Annals of cardiothoracic surgery 2017. 6 (2) 75-87.
328. Mendes-Filho, Antonio Moreira, Godoy, Eduardo Savio Nascimento, Alhinho, Helga Cristina Almeida Wahnon, Galvao-Neto, Manoel Dos Passos, Ramos, Almino Cardoso, Ferraz, Alvaro Antonio Bandeira, and Campos, Josemberg Marins. FUNDOPLICATION CONVERSION IN ROUX-EN-Y GASTRIC BYPASS FOR CONTROL OF OBESITY AND GASTROESOPHAGEAL REFLUX: SYSTEMATIC REVIEW. Arquivos brasileiros de cirurgia digestiva (Brazilian archives of digestive surgery) 2017. 30 (4) 279-282.
329. Bresalier, Robert S. Chemoprevention of Barrett's Esophagus and Esophageal Adenocarcinoma. Digestive diseases and sciences 2018. 63 (8) 2155-2162.
330. Spechler, Stuart J. Cardiac Metaplasia: Follow, Treat, or Ignore? Digestive diseases and sciences 2018. 63 (8) 2052-2058.
331. Clermont, Michelle and Falk, Gary W. Clinical Guidelines Update on the Diagnosis and Management of Barrett's Esophagus. Digestive diseases and sciences 2018. 63 (8) 2122-2128.
332. Akin, Hakan and Aydin, Yucel. How should we describe, diagnose and observe the Barrett's esophagus? The Turkish journal of gastroenterology 2017. 28 (Suppl 1) S26-S30.
333. Philpott, Hamish, Garg, Mayur, Tomic, Dunya, Balasubramanian, Smrithya, and Sweis, Rami. Dysphagia: Thinking outside the box. World journal of gastroenterology 2017. 23 (38) 6942-6951.
334. Amadi, Chidi and Gatenby, Piers. Barrett's oesophagus: Current controversies. World journal of gastroenterology 2017. 23 (28) 5051-5067.
335. Sjomina, Olga, Heluwaert, Frederic, Moussata, Driffa, and Leja, Marcis. Helicobacter pylori infection and nonmalignant diseases. Helicobacter 2017. 22 Suppl 1.
336. Schlottmann, Francisco and Patti, Marco G. Current Concepts in Treatment of Barrett's Esophagus With and Without Dysplasia. Journal of gastrointestinal surgery 2017. 21 (8) 1354-1360.
337. Schlottmann, Francisco, Patti, Marco G., and Shaheen, Nicholas J. From Heartburn to Barrett's Esophagus, and Beyond. World journal of surgery 2017. 41 (7) 1698-1704.
338. Nejat Pish-Kenari, F., Qujeq, D., and Maghsoudi, H. Some of the effective factors in the pathogenesis of gastro-oesophageal reflux disease. Journal of Cellular and Molecular Medicine 2018.
339. Mkarimi, M. and Mashimo, H. Advanced Imaging for Barrett's Esophagus and Early Neoplasia: Surface and Subsurface Imaging for Diagnosis and Management. Current Gastroenterology Reports 2018. 20 (12) 54.
340. Kav, T. To whom and when the upper gastrointestinal endoscopy is indicated in gastroesophageal reflux disease? What is the role of routine esophageal biopsy? Which endoscopic esophagitis classification should be used? Turkish Journal of Gastroenterology 2017. 28 (Supplement 1) S22-S25.
341. Inadomi, J., Alastal, H., Bonavina, L., Gross, S., Hunt, R. H., Mashimo, H., di, Pietro M., Rhee, H., Shah, M., Tolone, S., Wang, D. H., and Xie, S.-H. Recent advances in Barrett's esophagus. Annals of the New York Academy of Sciences 2018.
342. Otaki, F. and Iyer, P. G. Point-Counterpoint: Screening and Surveillance for Barrett's Esophagus, Is It Worthwhile? Digestive diseases and sciences 2018. 63 (8) 2081-2093.
343. Sackmann, M. Barrett's esophagus: Prevention and screening. Journal of Gastrointestinal and Liver Diseases 2018. 27 (Supplement 1) 13-14.
344. Michopoulos, S. Critical appraisal of guidelines for screening and surveillance of Barrett's esophagus. Annals of Translational Medicine 2018. 6 (13) 259.
345. Zakko, L., Visrodia, K., Leggett, C., Lutzke, L., and Wang, K. K. Screening patients for Barrett esophagus: Why, who, and how. Techniques in Gastrointestinal Endoscopy 2018. 20 (2) 55-61.
346. Savarino, E., Ottonello, A., Tolone, S., Bartolo, O., Baeg, M. K., Farjah, F., Kuribayashi, S., Shetler, K. P., Lottrup, C., and Stein, E. Novel insights into esophageal diagnostic procedures. Annals of the New York Academy of Sciences 2016. 1380 (1) 162-177.
347. Bisschops, R. Reflux and Barrett's diseaseCan we stop surveillance after 2011? Endoscopy 2012. 44 (4) 362-365.
348. Hatta, W., Tong, D., Lee, Y. Y., Ichihara, S., Uedo, N., and Gotoda, T. Different time trend and management of esophagogastric junction adenocarcinoma in three Asian countries. Digestive Endoscopy 2017. 29 (Supplement 2) 18-25.
349. Schoofs, N., Bisschops, R., and Prenen, H. Progression of barrett's esophagus toward esophageal adenocarcinoma: An overview. Annals of gastroenterology 2017. 30 (1) 1-6.

### Study design: Qualitative study (n=1)

1. Freeman, M., Offman, J., Walter, F. M., Sasieni, P., and Smith, S. G. Acceptability of the Cytosponge procedure for detecting Barrett's oesophagus: A qualitative study. BMJ open 2017. 7 (3) e013901.

### Study design: Single-arm cohort (non-comparative) (n=193)

1. Abdalla, Adil A., Petersen, Bret T., Ott, Beverly J., Fredericksen, Mary, Schleck, Cathy D., Zinsmeister, Alan R., Grunewald, Kassandra M. J., Zais, Teresa, and Romero, Yvonne. Impact of feedback and didactic sessions on the reporting behavior of upper endoscopic findings by physicians and nurses. Clinical gastroenterology and hepatology 2007; 5 (3): 326-330.
2. Blustein, P. K., Beck, P. L., Meddings, J. B., Van Rosendaal, G. M., Bailey, R. J., Lalor, E., Thomson, A. B., Verhoef, M. J., and Sutherland, L. R. The utility of endoscopy in the management of patients with gastroesophageal reflux symptoms. The American journal of gastroenterology 1998; 93 (12): 2508-2512.
3. Rossi, M., Barreca, M., De, Bortoli N., Renzi, C., Santi, S., Gennai, A., Bellini, M., Costa, F., Conio, M., and Marchi, S. Efficacy of Nissen fundoplication versus medical therapy in the regression of low-grade dysplasia in patients with Barrett esophagus: A prospective study. Annals of Surgery 2006; 243 (1): 58-63.
4. Melleney, E. M. A., Subhani, J. M., and Willoughby, C. P. Dysphagia referrals to a district general hospital gastroenterology unit: Hard to swallow. Dysphagia 2004; 19 (2): 78-82.
5. Saruc, Murat, Aksoy, Elif Ayanoglu, Vardereli, Eser, Karaaslan, Mehmet, Cicek, Bahattin, Ince, Umit, Oz, Ferhan, and Tozun, Nurdan Risk factors for laryngopharyngeal reflux. European archives of oto-rhino-laryngology 2012; 269 (4): 1189-1194.
6. Kauttu, Tuuli M. E., Rantanen, Tuomo K., Sihvo, Eero I., Rasanen, Jari V., Puolakkainen, Pauli, and Salo, Jarmo A. Esophageal adenocarcinoma arising after antireflux surgery: a population-based analysis. European journal of cardio-thoracic surgery 2011; 40 (6): 1450-1454.
7. Ronkainen, Jukka, Talley, Nicholas J., Storskrubb, Tom, Johansson, Sven Erik, Lind, Tore, Vieth, Michael, Agreus, Lars, and Aro, Pertti Erosive esophagitis is a risk factor for Barrett's esophagus: a community-based endoscopic follow-up study. The American journal of gastroenterology 2011; 106 (11): 1946-1952.
8. Tosato, F., Marano, S., Luongo, B., Paltrinieri, G., Mattacchione, S., and Bezzi, M. Total fundoplication without division of the short gastric vessels: functional evaluation at one year and review of literature. Minerva chirurgica 2011; 66 (2): 95-100.
9. Collen, M. J. and Strong, R. M. Comparison of omeprazole and ranitidine in treatment of refractory gastroesophageal reflux disease in patients with gastric acid hypersecretion. Digestive Diseases and Sciences 1992; 37 (6): 897-903.
10. Boreiri, M., Samadi, F., Etemadi, A., Babaei, M., Ahmadi, E., Houshang, Sharifi A., Nikmanesh, A., Houshiar, A., Pourfarzi, F., Yazdanbod, A., Alimohammadian, M., and Sotoudeh, M. Gastric cancer mortality in a high incidence area: Long- term follow-up of helicobacter pylori-related precancerous lesions in the general population. Archives of Iranian Medicine 2013; 16 (6): 343-347.
11. Malfertheiner, P., Lind, T., Willich, S., Vieth, M., Jaspersen, D., Labenz, J., Meyer-Sabellek, W., Junghard, O., and Stolte, M. Prognostic influence of Barrett's oesophagus and Helicobacter pylori infection on healing of erosive gastro-oesophageal reflux disease (GORD) and symptom resolution in non-erosive GORD: Report from the ProGORD study. Gut 2005; 54 (6): 746-751.
12. Kiesslich, R., Kanzler, S., Vieth, M., Moehler, M., Neidig, J., Thanka Nadar, B. J., Schilling, D., Burg, J., Nafe, B., Neurath, M. F., and Galle, P. R. Minimal change esophagitis: Prospective comparison of endoscopic and histological markers between patients with non-erosive reflux disease and normal controls using magnifying endoscopy. Digestive Diseases 2004; 22 (2): 221-227.
13. Kulig, M., Nocon, M., Vieth, M., Leodolter, A., Jaspersen, D., Labenz, J., Meyer-Sabellek, W., Stolte, M., Lind, T., Malfertheiner, P., and Willich, S. N. Risk factors of gastroesophageal reflux disease: Methodology and first epidemiological results of the ProGERD study. Journal of Clinical Epidemiology 2004; 57 (6): 580-589.
14. Wu, J. C. Y., Chan, F. K. L., Ching, J. Y. L., Leung, W.-K., Lee, Y.-T., and Sung, J. J. Y. Empirical treatment based on "typical" reflux symptoms is inappropriate in a population with a high prevalence of Helicobacter pylori infection. Gastrointestinal endoscopy 2002; 55 (4): 461-465.
15. Johansson, J., Johnsson, F., Walther, B., Willen, R., Von Holstein, C. S., and Zilling, T. Adenocarcinoma in the distal esophagus with and without Barrett esophagus: Differences in symptoms and survival rates. Archives of Surgery 1996; 131 (7): 708-713.
16. Collen, M. J., Johnson, D. A., and Sheridan, M. J. Basal acid output and gastric acid hypersecretion in gastroesophageal reflux disease. Correlation with ranitidine therapy. Digestive Diseases and Sciences 1994; 39 (2): 410-417.
17. Gorodner, Veronica, Buxhoeveden, Rudolf, Clemente, Gaston, Sanchez, Christian, Caro, Luis, and Grigaites, Alejandro Barrett's esophagus after Roux-en-Y gastric bypass: does regression occur? Surgical Endoscopy 2017 Apr 1;31(4):1849-54.
18. Promberger, Regina, Lenglinger, Johannes, Riedl, Otto, Seebacher, Gernot, Eilenberg, Wolf, Ott, Johannes, Riegler, Franz, Gadenstatter, Michael, and Neumayer, Christoph Gastro-oesophageal reflux disease in type 2 diabetics: symptom load and pathophysiologic aspects - a retro-pro study. BMC gastroenterology 2013; 13: 132-.
19. Ponce, Julio, Ortiz, Vicente, Maroto, Nuria, Ponce, Marta, Bustamante, Marco, and Garrigues, Vicente High prevalence of heartburn and low acid sensitivity in patients with idiopathic achalasia. Digestive Diseases and Sciences 2011; 56 (3): 773-776.
20. Sanduleanu, S., Stridsberg, M., Jonkers, D., Hameeteman, W., Biemond, I., Lundqvist, G., Lamers, C., and Stockbrugger, R. W. Serum gastrin and chromogranin A during medium- and long-term acid suppressive therapy: a case-control study. Alimentary pharmacology & therapeutics 1999; 13 (2): 145-153.
21. Topart, R., Deschamps, C., Taillefer, R., and Duranceau, A. [Scleroderma and esophageal reflux, surgical monitoring]. Annales de chirurgie 1992; 46 (9): 794-799.
22. Praveenraj, P., Gomes, R. M., Kumar, S., Senthilnathan, P., Parathasarathi, R., Rajapandian, S., and Palanivelu, C. Diagnostic yield and clinical implications of preoperative upper gastrointestinal endoscopy in morbidly obese patients undergoing bariatric surgery. Journal of Laparoendoscopic and Advanced Surgical Techniques 2015; 25 (6): 465-469.
23. Khandwalla, H. E., Graham, D. Y., Kramer, J. R., Ramsey, D. J., Duong, N., Green, L. K., and El-Serag, H. B. Barrett's Esophagus suspected at endoscopy but no specialized intestinal metaplasia on biopsy, what's next. American Journal of Gastroenterology 2014; 109 (2): 178-182.
24. Leeuwenburgh, I., Scholten, P., Calje, T. J., Vaessen, R. J., Tilanus, H. W., Hansen, B. E., and Kuipers, E. J. Barrett's esophagus and esophageal adenocarcinoma are common after treatment for achalasia. Digestive Diseases and Sciences 2013; 58 (1): 244-252.
25. Leodolter, A., Nocon, M., Vieth, M., Lind, T., Jaspersen, D., Richter, K., Willich, S., Stolte, M., Malfertheiner, P., and Labenz, J. Progression of specialized intestinal metaplasia at the cardia to macroscopically evident Barrett's esophagus: An entity of concern in the ProGERD study. Scandinavian Journal of Gastroenterology 2012; 47 (12): 1429-1435.
26. Dias, Pereira A. and Chaves, P. Columnar-lined oesophagus without intestinal metaplasia: Results from a cohort with a mean follow-up of 7 years. Alimentary Pharmacology and Therapeutics 2012; 36 (3): 282-289.
27. Malfertheiner, P., Nocon, M., Vieth, M., Stolte, M., Jaspersen, D., Koelz, H. R., Labenz, J., Leodolter, A., Lind, T., Richter, K., and Willich, S. N. Evolution of gastro-oesophageal reflux disease over 5 years under routine medical care - The ProGERD study. Alimentary Pharmacology and Therapeutics 2012; 35 (1): 154-164.
28. Rice, T. W., Goldblum, J. R., Rybicki, L. A., Rajeswaran, J., Murthy, S. C., Mason, D. P., and Blackstone, E. H. Fate of the esophagogastric anastomosis. Journal of Thoracic and Cardiovascular Surgery 2011; 141 (4): 875-.
29. Rantanen, T., Oksala, N., Honkanen, T., Rasanen, J., Sihvo, E., Mattila, J., Paimela, H., Paavonen, T., and Salo, J. The effect of fundoplication on proliferative and anti-apoptotic activity of esophageal mucosa in gastroesophageal reflux disease: 4-year follow-up study. Journal of Digestive Diseases 2011; 12 (4): 263-271.
30. Jacobson, B. C., Giovannucci, E. L., and Fuchs, C. S. Smoking and Barrett's esophagus in women who undergo upper endoscopy. Digestive Diseases and Sciences 2011; 56 (6): 1707-1717.
31. Hashemi, N., Loren, D., Dimarino, A. J., and Cohen, S. Presentation and prognosis of esophageal adenocarcinoma in patients below age 50. Digestive Diseases and Sciences 2009; 54 (8): 1708-1712.
32. Flameling, R. D., Numans, M. E., Ter, Linde J., De Wit, N. J., and Siersema, P. D. Different characteristics of patients with gastro-oesophageal reflux disease on their path through healthcare: A population follow-up study. European Journal of Gastroenterology and Hepatology 2010; 22 (5): 578-582.
33. Johansson, J., Hakansson, H.-O., Mellblom, L., Kempas, A., Kjellen, G., Brudin, L., Granath, F., Johansson, K.-E., and Nyren, O. Pancreatic acinar metaplasia in the distal oesophagus and the gastric cardia: Prevalence, predictors and relation to GORD. Journal of gastroenterology 2010; 45 (3): 291-299.
34. Yuasa, N., Abe, T., Sasaki, E., Fukaya, M., Nimura, Y., and Miyahara, R. Comparison of gastroesophageal reflux in 100 patients with or without prior gastroesophageal surgery. Journal of gastroenterology 2009; 44 (7): 650-658.
35. Jacobson, B. C., Chan, A. T., Giovannucci, E. L., and Fuchs, C. S. Body mass index and Barrett's oesophagus in women. Gut 2009; 58 (11): 1460-1466.
36. Williams, J. F., Sontag, S. J., Schnell, T., and Leya, J. Non-cardiac chest pain: The long-term natural history and comparison with gastroesophageal reflux disease. American Journal of Gastroenterology 2009; 104 (9): 2145-2152.
37. Falkenback, D., Oberg, S., Johnsson, F., and Johansson, J. Is the course of gastroesophageal reflux disease progressive? A 21-year follow-up. Scandinavian Journal of Gastroenterology 2009; 44 (11): 1277-1287.
38. Moriyama, N., Amano, Y., Mishima, Y., Okita, K., Takahashi, Y., Yuki, T., Ishimura, N., Ishihara, S., and Kinoshita, Y. What is the clinical significance of stromal angiogenesis in Barrett's esophagus? Journal of Gastroenterology and Hepatology (Australia) 2008; 23 (SUPPL. 2): S210-S215.
39. Yamagishi, H., Koike, T., Ohara, S., Kobayashi, S., Ariizumi, K., Abe, Y., Iijima, K., Imatani, A., Inomata, Y., Kato, K., Shibuya, D., Aida, S., and Shimosegawa, T. Tongue-like Barrett's esophagus is associated with gastroesophageal reflux disease. World journal of gastroenterology 2008; 14 (26): 4196-4203.
40. Okita, K., Amano, Y., Takahashi, Y., Mishima, Y., Moriyama, N., Ishimura, N., Ishihara, S., and Kinoshita, Y. Barrett's esophagus in Japanese patients: Its prevalence, form, and elongation. Journal of gastroenterology 2008; 43 (12): 928-934.
41. Akiyama, T., Inamori, M., Akimoto, K., Iida, H., Mawatari, H., Endo, H., Nozaki, Y., Yoneda, K., Ikeda, T., Sakamoto, Y., Fujita, K., Yoneda, M., Takahashi, H., Goto, A., Hirokawa, S., Abe, Y., Kirikoshi, H., Kobayashi, N., Kubota, K., Saito, S., Rino, Y., and Nakajima, A. Gastric surgery is not a risk factor for the development or progression of Barrett's epithelium. Hepato-gastroenterology 2008; 55 (86-87): 1899-1904.
42. Bozikas, A., Marsman, W. A., Rosmolen, W. D., van Baal, J. W. P. M., Kulik, W., Ten Kate, F. J. W., Krishnadath, K. K., and Bergman, J. J. G. H. The effect of oral administration of ursodeoxycholic acid and high-dose proton pump inhibitors on the histology of Barrett's esophagus. Diseases of the Esophagus 2008; 21 (4): 346-354.
43. Ogiya, K., Kawano, T., Ito, E., Nakajima, Y., Kawada, K., Nishikage, T., and Nagai, K. Lower esophageal palisade vessels and the definition of Barrett's esophagus. Diseases of the Esophagus 2008; 21 (7): 645-649.
44. Zagari, R. M., Fuccio, L., Wallander, M.-A., Johansson, S., Fiocca, R., Casanova, S., Farahmand, B. Y., Winchester, C. C., Roda, E., and Bazzoli, F. Gastro-oesophageal reflux symptoms, oesophagitis and barrett's oesophagus in the general population: The Loiano-Monghidoro study. Gut 2008; 57 (10): 1354-1359.
45. Bax, D., Siersema, P. D., Moons, L. M. G., Dekken, H. V., Tilanus, H. W., Kusters, J. G., and Kuipers, E. J. CDX2 expression in columnar metaplasia of the remnant esophagus in patients who underwent esophagectomy. Journal of Clinical Gastroenterology 2007; 41 (4): 375-379.
46. Oh, D. S., DeMeester, S. R., Vallbohmer, D., Mori, R., Kuramochi, H., Hagen, J. A., Lipham, J., Danenberg, K. D., Danenberg, P. V., Chandrasoma, P., and DeMeester, T. R. Reduction of interleukin 8 gene expression in reflux esophagitis and Barrett's esophagus with antireflux surgery. Archives of Surgery 2007; 142 (6): 554-559.
47. Stoltey, J., Reeba, H., Ullah, N., Sabhaie, P., and Gerson, L. Does Barrett's oesophagus develop over time in patients with chronic gastro-oesophageal reflux disease?. Alimentary Pharmacology and Therapeutics 2007; 25 (1): 83-91.
48. Ward, E. M., Wolfsen, H. C., Achem, S. R., Loeb, D. S., Krishna, M., Hemminger, L. L., and DeVault, K. R. Barrett's esophagus is common in older men and women undergoing screening colonoscopy regardless of reflux symptoms. American Journal of Gastroenterology 2006; 101 (1): 12-17.
49. Seltman, A. K., Kahrilas, P. J., Chang, E. Y., Mori, M., Hunter, J. G., and Jobe, B. A. Endoscopic measurement of cardia circumference as an indicator of GERD. Gastrointestinal endoscopy 2006; 63 (1): 22-31.
50. Sontag, S. J., Sonnenberg, A., Schnell, T. G., Leya, J., and Metz, A. The long-term natural history of gastroesophageal reflux disease. Journal of Clinical Gastroenterology 2006; 40 (5): 398-404.
51. Oksala, N. K. J., Atalay, M., and Rantanen, T. K. Antireflux surgery and esophageal mucosal DNA damage. Pathophysiology 2006; 13 (1): 23-27.
52. Labenz, J., Nocon, M., Lind, T., Leodolter, A., Jaspersen, D., Meyer-Sabellek, W., Stolte, M., Vieth, M., Willich, S. N., and Malfertheiner, P. Prospective follow-up data from the ProGERD study suggest that GERD is not a categorial disease. American Journal of Gastroenterology 2006; 101 (11): 2457-2462.
53. Sundar, N., Muraleedharan, V., Pandit, J., Green, J. T., Crimmins, R., and Swift, G. L. Does endoscopy diagnose early gastrointestinal cancer in patients with uncomplicated dyspepsia?. Postgraduate Medical Journal 2006; 82 (963): 52-54.
54. Wipff, J., Allanore, Y., Soussi, F., Terris, B., Abitbol, V., Raymond, J., Chaussade, S., and Kahan, A. Prevalence of Barrett's esophagus in systemic sclerosis. Arthritis and Rheumatism 2005; 52 (9): 2882-2888.
55. Stein, D. J., El-Serag, H. B., Kuczynski, J., Kramer, J. R., and Sampliner, R. E. The association of body mass index with Barrett's oesophagus. Alimentary Pharmacology and Therapeutics 2005; 22 (10): 1005-1010.
56. Wo, J. M., Mendez, C., Harrell, S., Joubran, R., Bressoud, P. F., and McKinney, W. P. Clinical impact of upper endoscopy in the management of patients with gastroesophageal reflux disease. American Journal of Gastroenterology 2004; 99 (12): 2311-2316.
57. Labenz, J., Jaspersen, D., Kulig, M., Leodolter, A., Lind, T., Meyer-Sabellek, W., Stolte, M., Vieth, M., Willich, S., and Malfertheiner, P. Risk factors for erosive esophagitis: A multivariate analysis based on the proGERD study initiative. American Journal of Gastroenterology 2004; 99 (9): 1652-1656.
58. El-Serag, H. B., Aguirre, T., Kuebeler, M., and Sampliner, R. E. The length of newly diagnosed Barrett's oesophagus and prior use of acid suppressive therapy. Alimentary Pharmacology and Therapeutics 2004; 19 (12): 1255-1260.
59. Chak, A., Faulx, A., Kinnard, M., Brock, W., Willis, J., Wiesner, G. L., Parrado, A. R., and Goddard, K. A. B. Identification of Barrett's esophagus in relatives by endoscopic screening. American Journal of Gastroenterology 2004; 99 (11): 2107-2114.
60. Desai, K. M., Soper, N. J., Frisella, M. M., Quasebarth, M. A., Dunnegan, D. L., and Brunt, L. M. Efficacy of laparoscopic antireflux surgery in patients with Barrett's esophagus. American journal of surgery 2003; 186 (6): 652-659.
61. Dresner, S. M., Griffin, S. M., Wayman, J., Bennett, M. K., Hayes, N., and Raimes, S. A. Human model of duodenogastro-oesophageal reflux in the development of Barrett's metaplasia. British Journal of Surgery 2003; 90 (9): 1120-1128.
62. Cameron, A. J. and Arora, A. S. Barrett's esophagus and reflux esophagitis: Is there a missing link? American Journal of Gastroenterology 2002; 97 (2): 273-278.
63. Wetscher, G. J., Gadenstaetter, M., Klingler, P. J., Weiss, H., Obrist, P., Wykypiel, H., Klaus, A., and Profanter, C. Efficacy of medical therapy and antireflux surgery to prevent Barrett's metaplasia in patients with gastroesophageal reflux disease. Annals of Surgery 2001; 234 (5): 627-632.
64. Levine, D. S., Blount, P. L., Rudolph, R. E., and Reid, B. J. Safety of a systematic endoscopic biopsy protocol in patients with Barrett's esophagus. American Journal of Gastroenterology 2000; 95 (5): 1152-1157.
65. Mittal, S. K., Awad, Z. T., Tasset, M., Filipi, C. J., Dickason, T. J., Shinno, Y., Marsh, R. E., Tomonaga, T. J., and Lerner, C. The preoperative predictability of the short esophagus in patients with stricture or paraesophageal hernia. Surgical endoscopy 2000; 14 (5): 464-468.
66. Rantanen, T. K., Halme, T. V., Luostarinen, M. E., Karhumaki, L. M., Kononen, E. O., and Isolauri, J. O. The long term results of open antireflux surgery in a community-based health care center. American Journal of Gastroenterology 1999; 94 (7): 1777-1781.
67. Farrell, T. M., Smith, C. D., Metreveli, R. E., Johnson, A. B., Galloway, K. D., and Hunter, J. G. Fundoplication provides effective and durable symptom relief in patients with Barrett's esophagus. American journal of surgery 1999; 178 (1): 18-21.
68. Schenk, B. E., Kuipers, E. J., Klinkenberg-Knol, E. C., Eskes, S. A., and Meuwissen, S. G. M. Helicobacter pylori and the efficacy of omeprazole therapy for gastroesophageal reflux disease. American Journal of Gastroenterology 1999; 94 (4): 884-887.
69. Hackelsberger, A., Gunther, T., Schultze, V., Manes, G., Dominguez-Munoz, J.-E., Roessner, A., and Malfertheiner, P. Intestinal metaplasia at the gastro-oesophageal junction: Helicobacter pylori gastritis or gastro-oesophageal reflux disease? Gut 1998; 43 (1): 17-21.
70. Somppi, E., Tammela, O., Ruuska, T., Rahnasto, J., Laitinen, J., Turjanmaa, V., and Jarnberg, J. Outcome of patients operated on for esophageal atresia: 30 years' experience. Journal of Pediatric Surgery 1998; 33 (9): 1341-1346.
71. Bersentes, K., Fass, R., Padda, S., Johnson, C., and Sampliner, R. E. Prevalence of Barrett's esophagus in Hispanics is similar to Caucasians. Digestive Diseases and Sciences 1998; 43 (5): 1038-1041.
72. Kiviluoto, T., Siren, J., Farkkila, M., Luukkonen, P., Salo, J., and Kivilaakso, E. Laparoscopic nissen fundoplication: A prospective analysis of 200 consecutive patients. Surgical Laparoscopy and Endoscopy 1998; 8 (6): 429-434.
73. Newton, M., Bryan, R., Burnham, W. R., and Kamm, M. A. Evaluation of Helicobacter pylori in reflux oesophagitis and Barrett's oesophagus. Gut 1997; 40 (1): 9-13.
74. Hunter, J. G., Trus, T. L., Branum, G. D., Waring, J. P., and Wood, W. C. A physiologic approach to laparoscopic fundoplication for gastroesophageal reflux disease. Annals of Surgery 1996; 223 (6): 673-687.
75. Johnston, B. T., Carre, I. J., Thomas, P. S., and Collins, B. J. Twenty to 40 year follow up of infantile hiatal hernia. Gut 1995; 36 (6): 809-812.
76. Sontag, S. J., Schnell, T. G., Miller, T. Q., Khandelwal, S., O'Connell, S., Chejfec, G., Greenlee, H., Seidel, U. J., and Brand, L. Prevalence of oesophagitis in asthmatics. Gut 1992; 33 (7): 872-876.
77. Shimoyama, Shouji, Ogawa, Toshihisa, and Toma, Toshiyuki Trajectories of endoscopic Barrett esophagus: Chronological changes in a community-based cohort. World journal of gastroenterology 2016; 22 (35): 8060-8066.
78. Alkaddour, Ahmad, McGaw, Camille, Hritani, Rama, Palacio, Carlos, Nakshabendi, Rahman, Munoz, Juan Carlos, and Vega, Kenneth J. African American ethnicity is not associated with development of Barrett's oesophagus after erosive oesophagitis. Digestive and liver disease 2015; 47 (10): 853-856.
79. Dutta, Sudhir K., Agrawal, Kireet, Girotra, Mohit, Fleisher, A. Steven, Motevalli, Mahnaz, Mah'moud, Mitchell A., and Nair, Padmanabhan P. Barrett's esophagus and beta-carotene therapy: symptomatic improvement in GERD and enhanced HSP70 expression in esophageal mucosa. Asian Pacific journal of cancer prevention: APJCP 2012; 13 (12): 6011-6016.
80. Faintuch, Jacob Jehuda, Silva, Fernando Marcuz, Navarro-Rodriguez, Tomas, Barbuti, Ricardo Correa, Hashimoto, Claudio Lyoiti, Rossini, Alessandra Rita Asayama Lopes, Diniz, Marcio Augusto, and Eisig, Jaime Natan Endoscopic findings in uninvestigated dyspepsia. BMC gastroenterology 2014; 14: 19-.
81. Galindo, G., Vassalle, J., Marcus, S. N., and Triadafilopoulos, G. Multimodality evaluation of patients with gastroesophageal reflux disease symptoms who have failed empiric proton pump inhibitor therapy. Diseases of the esophagus 2013; 26 (5): 443-450.
82. Qureshi, Alia P., Aye, Ralph W., Buduhan, Gordon, Knight, Ariel, Orlina, Jeraldine, Farivar, Alexander S., Wagner, Oliver J., McHugh, Sean, and Louie, Brian E. The laparoscopic Nissen-Hill hybrid: pilot study of a combined antireflux procedure. Surgical endoscopy 2013; 27 (6): 1945-1952.
83. Kuo, Chia Jung, Lin, Cheng Hui, Liu, Nai Jen, Wu, Ren Chin, Tang, Jui Hsiang, and Cheng, Chi Liang Frequency and risk factors for Barrett's esophagus in Taiwanese patients: a prospective study in a tertiary referral center. Digestive Diseases and Sciences 2010; 55 (5): 1337-1343.
84. Lord, Reginald V. N., Demeester, Steven R., Peters, Jeffrey H., Hagen, Jeffrey A., Elyssnia, Dino, Sheth, Corinne T., and Demeester, Tom R. Hiatal hernia, lower esophageal sphincter incompetence, and effectiveness of Nissen fundoplication in the spectrum of gastroesophageal reflux disease. Journal of gastrointestinal surgery 2009; 13 (4): 602-610.
85. Lee, Sang Kuon and Kim, Eung Kook Laparoscopic Nissen fundoplication in Korean patients with gastroesophageal reflux disease. Yonsei medical journal 2009; 50 (1): 89-94.
86. Anandasabapathy, Sharmila, Jhamb, Jagriti, Davila, Marta, Wei, Caimiao, Morris, Jeffrey, and Bresalier, Robert Clinical and endoscopic factors predict higher pathologic grades of Barrett dysplasia. Cancer 2007; 109 (4): 668-674.
87. Mullin, J. M., Valenzano, M. C., Trembeth, S., Allegretti, P. D., Verrecchio, J. J., Schmidt, J. D., Jain, V., Meddings, J. B., Mercogliano, G., and Thornton, J. J. Transepithelial leak in Barrett's esophagus. Digestive Diseases and Sciences 2006; 51 (12): 2326-2336.
88. Frazzoni, M., Manno, M., De Micheli, E., and Savarino, V. Pathophysiological characteristics of the various forms of gastro-oesophageal reflux disease. Spectrum disease or distinct phenotypic presentations? Digestive and liver disease 2006; 38 (9): 643-648.
89. Vallbohmer, Daniel, Demeester, Steven R., Oh, Daniel S., Banki, Farzaneh, Kuramochi, Hidekazu, Shimizu, Daisuke, Hagen, Jeffrey A., Danenberg, Kathleen D., Danenberg, Peter V., Chandrasoma, Parakrama T., Peters, Jeffrey H., and Demeester, Tom R. Antireflux surgery normalizes cyclooxygenase-2 expression in squamous epithelium of the distal esophagus. The American journal of gastroenterology 2006; 101 (7): 1458-1466.
90. Hanna, Sameh, Rastogi, Amit, Weston, Allan P., Totta, Frank, Schmitz, Robert, Mathur, Sharad, McGregor, Douglas, Cherian, Rachel, and Sharma, Prateek Detection of Barrett's esophagus after endoscopic healing of erosive esophagitis. The American journal of gastroenterology 2006; 101 (7): 1416-1420.
91. Leeuwenburgh, I., Van Dekken, H., Scholten, P., Hansen, B. E., Haringsma, J., Siersema, P. D., and Kuipers, E. J. Oesophagitis is common in patients with achalasia after pneumatic dilatation. Alimentary pharmacology & therapeutics 2006; 23 (8): 1197-1203.
92. Bhat, Yasser M. and Bielefeldt, Klaus Capsaicin receptor (TRPV1) and non-erosive reflux disease. European journal of gastroenterology & hepatology 2006; 18 (3): 263-270.
93. Velanovich, Vic and Mohlberg, Nathan The split-stomach fundoplication after esophagogastrectomy. Journal of gastrointestinal surgery 2006; 10 (2): 178-5.
94. Ozmen, V., Oran, E Sen, Gorgun, E., Asoglu, O., Igci, A., Kecer, M., and Dizdaroglu, F. Histologic and clinical outcome after laparoscopic Nissen fundoplication for gastroesophageal reflux disease and Barrett's esophagus. Surgical endoscopy 2006; 20 (2): 226-229.
95. Eliakim, R., Yassin, K., Shlomi, I., Suissa, A., and Eisen, G. M. A novel diagnostic tool for detecting oesophageal pathology: the PillCam oesophageal video capsule. Alimentary pharmacology & therapeutics 2004; 20 (10): 1083-1089.
96. Lin, Edward, Swafford, Vickie, Chadalavada, Rajagopal, Ramshaw, Bruce J., and Smith, C. Daniel Disparity between symptomatic and physiologic outcomes following esophageal lengthening procedures for antireflux surgery. Journal of gastrointestinal surgery 2004; 8 (1): 31-39.
97. Papasavas, P. K., Keenan, R. J., Yeaney, W. W., Caushaj, P. F., Gagne, D. J., and Landreneau, R. J. Effectiveness of laparoscopic fundoplication in relieving the symptoms of gastroesophageal reflux disease (GERD) and eliminating antireflux medical therapy. Surgical endoscopy 2003; 17 (8): 1200-1205.
98. Kulig, M., Leodolter, A., Vieth, M., Schulte, E., Jaspersen, D., Labenz, J., Lind, T., Meyer-Sabellek, W., Malfertheiner, P., Stolte, M., and Willich, S. N. Quality of life in relation to symptoms in patients with gastro-oesophageal reflux disease-- an analysis based on the ProGERD initiative. Alimentary pharmacology & therapeutics 2003; 18 (8): 767-776.
99. Richards, William O., Houston, Hugh L., Torquati, Alfonso, Khaitan, Leena, Holzman, Michael D., and Sharp, Kenneth W. Paradigm shift in the management of gastroesophageal reflux disease. Annals of Surgery 2003; 237 (5): 638-639.
100. Nasseri-Moghaddam, Siavosh, Malekzadeh, Reza, Sotoudeh, Masoud, Tavangar, Mohammad, Azimi, Kourosh, Sohrabpour, Amir Ali, Mostadjabi, Pardis, Fathi, Hosnieh, and Minapoor, Mina Lower esophagus in dyspeptic Iranian patients: a prospective study. Journal of gastroenterology and hepatology 2003; 18 (3): 315-321.
101. Wong, W. M., Lam, S. K., Hui, W. M., Lai, K. C., Chan, C. K., Hu, W. H. C., Xia, H. H. X., Hui, C. K., Yuen, M. F., Chan, A. O. O., and Wong, B. C. Y. Long-term prospective follow-up of endoscopic oesophagitis in southern Chinese--prevalence and spectrum of the disease. Alimentary pharmacology & therapeutics 2002; 16 (12): 2037-2042.
102. Donahue, Philip E., Horgan, Santiago, Liu, Katherine J. M., and Madura, James A. Floppy Dor fundoplication after esophagocardiomyotomy for achalasia. Surgery (United Kingdom) 2002; 132 (4): 716-3.
103. Lindstrom, Dean R., Wallace, James, Loehrl, Todd A., Merati, Albert L., and Toohill, Robert J. Nissen fundoplication surgery for extraesophageal manifestations of gastroesophageal reflux (EER). The Laryngoscope 2002; 112 (10): 1762-1765.
104. Todd, J. A., Johnston, D. A., and Dillon, J. F. The changing spectrum of gastroesophageal reflux disease. European journal of cancer prevention 2002; 11 (3): 215-219.
105. Kamolz, T., Bammer, T., Granderath, F. A., and Pointner, R. Comorbidity of aerophagia in GERD patients: outcome of laparoscopic antireflux surgery. Scandinavian Journal of Gastroenterology 2002; 37 (2): 138-143.
106. Awad, Z. T., Mittal, S. K., Roth, T. A., Anderson, P. I., Jr, W. A., and Filipi, C. J. Esophageal shortening during the era of laparoscopic surgery. World Journal of Surgery 2001; 25 (5): 558-561.
107. Csendes, A., Smok, G., Flores, N., Rojas, J., Quiroz, J., and Henriquez, A. Comparison of clinical, endoscopic and functional findings in patients with intestinal metaplasia at the cardia, carditis and short-segment columnar epithelium of the distal esophagus with and without intestinal metaplasia. Diseases of the esophagus 2000; 13 (1): 61-68.
108. Braghetto, I., Csendes, A., Burdiles, P., and Korn, O. Antireflux surgery, highly selective vagotomy and duodenal switch procedure: post-operative evaluation in patients with complicated and non-complicated Barrett's esophagus. Diseases of the esophagus 2000; 13 (1): 12-17.
109. Reid, B. J., Levine, D. S., Longton, G., Blount, P. L., and Rabinovitch, P. S. Predictors of progression to cancer in Barrett's esophagus: baseline histology and flow cytometry identify low- and high-risk patient subsets. The American journal of gastroenterology 2000; 95 (7): 1669-1676.
110. Yau, P., Watson, D. I., Devitt, P. G., Game, P. A., and Jamieson, G. G. Laparoscopic antireflux surgery in the treatment of gastroesophageal reflux in patients with Barrett esophagus. Archives of surgery (Chicago, Ill.: 1960) 2000; 135 (7): 801-805.
111. Patti, M. G., Arcerito, M., Feo, C. V., Worth, S., De Pinto, M., Gibbs, V. C., Gantert, W., Tyrrell, D., Ferrell, L. F., and Way, L. W. Barrett's esophagus: a surgical disease. Journal of gastrointestinal surgery 1999; 3 (4): 397-4.
112. Shai, S. E., Chen, C. Y., Hsu, C. P., Hsia, J. Y., and Yang, S. S. Transthoracic oesophagomyotomy in the treatment of achalasia--a 15-year experience. Scandinavian cardiovascular journal: SCJ 1999; 33 (6): 333-336.
113. Horvath, K. D., Jobe, B. A., Herron, D. M., and Swanstrom, L. L. Laparoscopic Toupet fundoplication is an inadequate procedure for patients with severe reflux disease. Journal of gastrointestinal surgery 1999; 3 (6): 583-591.
114. Trastek, V. F., Deschamps, C., Allen, M. S., Miller, D. L., Pairolero, P. C., and Thompson, A. M. Uncut Collis-Nissen fundoplication: learning curve and long-term results. The Annals of thoracic surgery 1998; 66 (5): 1739-1744.
115. McDougall, N. I., Johnston, B. T., Collins, J. S., McFarland, R. J., and Love, A. H. Disease progression in gastro-oesophageal reflux disease as determined by repeat oesophageal pH monitoring and endoscopy 3 to 4.5 years after diagnosis. European journal of gastroenterology & hepatology 1997; 9 (12): 1161-1167.
116. Ritter, D. W., Vanderpool, D., and Westmoreland, M. Laparoscopic Nissen fundoplication for gastroesophageal reflux disease. American journal of surgery 1997; 174 (6): 715-718.
117. Wetscher, G. J., Profanter, C., Gadenstatter, M., Perdikis, G., Glaser, K., and Hinder, R. A. Medical treatment of gastroesophageal reflux disease does not prevent the development of Barrett's metaplasia and poor esophageal body motility. Langenbecks Archiv fur Chirurgie 1997; 382 (2): 95-99.
118. Isolauri, J., Luostarinen, M., Viljakka, M., Isolauri, E., Keyrilainen, O., and Karvonen, A. L. Long-term comparison of antireflux surgery versus conservative therapy for reflux esophagitis. Annals of Surgery 1997; 225 (3): 295-299.
119. McDougall, N. I., Johnston, B. T., Kee, F., Collins, J. S., McFarland, R. J., and Love, A. H. Natural history of reflux oesophagitis: a 10 year follow up of its effect on patient symptomatology and quality of life. Gut 1996; 38 (4): 481-486.
120. Jones, R., Canal, D. F., Inman, M. M., and Rescorla, F. J. Laparoscopic fundoplication: a three-year review. The American surgeon 1996; 62 (8): 632-636.
121. Vanamo, K., Rintala, R. J., Lindahl, H., and Louhimo, I. Long-term gastrointestinal morbidity in patients with congenital diaphragmatic defects. Journal of Pediatric Surgery 1996; 31 (4): 551-554.
122. Spechler, S. J., Zeroogian, J. M., Antonioli, D. A., Wang, H. H., and Goyal, R. K. Prevalence of metaplasia at the gastro-oesophageal junction. Lancet (London, England) 1994; 344 (8936): 1533-1536.
123. Carr, N. J., Monihan, J. M., and Sobin, L. H. Squamous cell papilloma of the esophagus: a clinicopathologic and follow-up study of 25 cases. The American journal of gastroenterology 1994; 89 (2): 245-248.
124. Jaakkola, A., Reinikainen, P., Ovaska, J., and Isolauri, J. Barrett's esophagus after cardiomyotomy for esophageal achalasia. The American journal of gastroenterology 1994; 89 (2): 165-169.
125. Luostarinen, M., Isolauri, J., Laitinen, J., Koskinen, M., Keyrilainen, O., Markkula, H., Lehtinen, E., and Uusitalo, A. Fate of Nissen fundoplication after 20 years. A clinical, endoscopical, and functional analysis. Gut 1993; 34 (8): 1015-1020.
126. Johansson, J., Johnsson, F., Joelsson, B., Floren, C. H., and Walther, B. Outcome 5 years after 360 degree fundoplication for gastro-oesophageal reflux disease. The British journal of surgery 1993; 80 (1): 46-49.
127. Hendel, L., Hage, E., Hendel, J., and Stentoft, P. Omeprazole in the long-term treatment of severe gastro-oesophageal reflux disease in patients with systemic sclerosis. Alimentary pharmacology & therapeutics 1992; 6 (5): 565-577.
128. Collen, M. J., Lewis, J. H., and Benjamin, S. B. Gastric acid hypersecretion in refractory gastroesophageal reflux disease. Gastroenterology 1990; 98 (3): 654-661.
129. Perniceni, T., Leymarios, J., Molas, G., and Fekete, F. [Does Barrett esophagus regress after total duodenal diversion?]. Gastroenterologie clinique et biologique 1988; 12 (10): 709-712.
130. Lee, F. I. and Isaacs, P. E. Barrett's ulcer: response to standard dose ranitidine, high dose ranitidine, and omeprazole. The American journal of gastroenterology 1988; 83 (9): 914-916.
131. Ransom, J. M., Patel, G. K., Clift, S. A., Womble, N. E., and Read, R. C. Extended and limited types of Barrett's esophagus in the adult. The Annals of thoracic surgery 1982; 33 (1): 19-27.
132. Mosimann, R., Walder, J., Buchheim, G., Ollyo, J. B., Loup, P., and Fasel, J. [Gastroesophageal reflux: long-term results of surgical management]. Helvetica chirurgica acta 1981; 47 (6): 749-757.
133. Pohl, H., Robertson, D., and Welch, H. G. Repeated Upper Endoscopy in the Medicare Population A Retrospective Analysis. Annals of Internal Medicine 2014; 160 (3): 154-160.
134. Nkuize, M., De, Wit S., Muls, V., Pirenne, C., and Buset, M. The role of upper gastrointestinal endoscopy in the era of modern antiretroviral therapy. European Journal of Gastroenterology and Hepatology 2015; 27 (12): 1459-1465.
135. Royston, C., Caygill, C., Charlett, A., and Bardhan, K. D. The evolution and outcome of surveillance of Barrett's oesophagus over four decades in a UK District General Hospital. European Journal of Gastroenterology and Hepatology 2016; 28(12):1365-73.
136. Braghetto, I. and Csendes, A. Prevalence of Barrett's Esophagus in Bariatric Patients Undergoing Sleeve Gastrectomy. Obesity Surgery 2016; 26 (4): 710-714.
137. Picardo, S. L., O'Brien, M. P., Feighery, R., O'Toole, D., Ravi, N., O'Farrell, N. J., O'Sullivan, J. N., and Reynolds, J. V. A Barrett's esophagus registry of over 1000 patients from a specialist center highlights greater risk of progression than population-based registries and high risk of low grade dysplasia. Diseases of the Esophagus 2015; 28 (2): 121-126.
138. Dworkin, J. P., Dowdall, J. R., Kubik, M., Thottam, P. J., and Folbe, A. The Role of the Modified Barium Swallow Study and Esophagram in Patients with Globus Sensation. Dysphagia 2015; 30 (5): 506-510.
139. Kim, A., Park, W.-Y., Shin, N., Lee, H. J., Kim, Y. K., Lee, S. J., Hwang, C.-S., Park, D. Y., Kim, G. H., Lee, B. E., and Jo, H.-J. Cardiac mucosa at the gastroesophageal junction: An Eastern perspective. World journal of gastroenterology 2015; 21 (30): 9126-9133.
140. Greene, C. L., DeMeester, S. R., Augustin, F., Worrell, S. G., Oh, D. S., Hagen, J. A., and DeMeester, T. R. Long-term quality of life and alimentary satisfaction after esophagectomy with colon interposition. Annals of Thoracic Surgery 2014; 98 (5): 1713-1719.
141. Zehetner, J., DeMeester, S. R., Ayazi, S., Kilday, P., Alicuben, E. T., and DeMeester, T. R. Laparoscopic wedge fundectomy for collis gastroplasty creation in patients with a foreshortened esophagus. Annals of Surgery 2014; 260 (6): 1030-1033.
142. Alexander, P., Ramya, S., Solomon, R., Raja, S., Priyadarshini, M., Geetha, R., Srinivasan, V., and Jayanthi, V. Effects of long-term acid suppressants with ranitidine and omeprazole on gastric mucosa. Journal of Digestive Endoscopy 2013; 4 (1): 1-5.
143. Cade, R. J., Fox, A. M., Fahy, E. T., and Hii, M. W. Prevention of Barrett's metaplasia in a human model of duodenogastro-oesophageal reflux. Gastroenterology Insights 2013; 5 (1): 12-15.
144. Becker, V., Bobardt, J., Ott, R., Rosch, T., and Meining, A. Long-Term Follow-Up in Patients with Indeterminate Barrett Esophagus. Digestion 2013; 88 (3): 161-164.
145. Zschau, N. B., Andrews, J. M., Holloway, R. H., Schoeman, M. N., Lange, K., Tam, W. C. E., and Holtmann, G. J. Gastroesophageal reflux disease after diagnostic endoscopy in the clinical setting. World journal of gastroenterology 2013; 19 (16): 2514-2520.
146. Yachimski, P. Use of narrow band imaging in a group endoscopy practice. Endoscopy International Open 2013; 1 (1): E8-E11.
147. Zhang, M., Fan, X.-S., and Zou, X.-P. The prevalence of Barrett's esophagus remains low in Eastern China: Single-center 7-year descriptive study. Saudi Medical Journal 2012; 33 (12): 1324-1329.
148. Durand, L., De, Anton R., Caracoche, M., Covian, E., Gimenez, M., Ferraina, P., and Swanstrom, L. Short esophagus: Selection of patients for surgery and long-term results. Surgical Endoscopy and Other Interventional Techniques 2012; 26 (3): 704-713.
149. Tosato, F., Marano, S., Mattacchione, S., Luongo, B., Mingarelli, V., and Campagna, G. Quality of life after nissen-rossetti fundoplication. Surgical Laparoscopy, Endoscopy and Percutaneous Techniques 2012; 22 (3): 205-209.
150. Jung, K. W., Talley, N. J., Romero, Y., Katzka, D. A., Schleck, C. D., Zinsmeister, A. R., Dunagan, K. T., Lutzke, L. S., Wu, T.-T., Wang, K. K., Frederickson, M., Geno, D. M., Locke, G. R., and Prasad, G. A. Epidemiology and natural history of intestinal metaplasia of the gastroesophageal junction and barrett's esophagus: A population-based study. American Journal of Gastroenterology 2011; 106 (8): 1447-1455.
151. Braghetto, I., Korn, O., Cardemil, G., Coddou, E., Valladares, H., and Henriquez, A. Inversed Y cardioplasty plus a truncal vagotomy-antrectomy and a Roux-en-Y gastrojejunostomy performed in patients with stricture of the esophagogastric junction after a failed cardiomyotomy or endoscopic procedure in patients with achalasia of the esophagus. Diseases of the Esophagus 2010; 23 (3): 208-215.
152. Smithers, B. M., Fahey, P. P., Corish, T., Gotley, D. C., Falk, G. L., Smith, G. S., Kiroff, G. K., Clouston, A. D., Watson, D. I., and Whiteman, D. C. Symptoms, investigations and management of patients with cancer of the oesophagus and gastro-oesophageal junction in Australia. Medical Journal of Australia 2010; 193 (10): 572-577.
153. Modiano, N. and Gerson, L. B. Risk factors for the detection of Barrett's esophagus in patients with erosive esophagitis. Gastrointestinal endoscopy 2009; 69 (6): 1014-1020.
154. Nayyar, A. K., Royston, C., and Bardhan, K. D. Oesophageal acid-peptic strictures in the histamine H2 receptor antagonist and proton pump inhibitor era. Digestive and Liver Disease 2003; 35 (3): 143-150.
155. Markus, P. M., Horstmann, O., Kley, C., Neufang, T., and Becker, H. Laparoscopic fundoplication: Is there a correlation between pH studies and the patient's quality of life? Surgical Endoscopy and Other Interventional Techniques 2002; 16 (1): 48-53.
156. Bowers, S. P., Mattar, S. G., Smith, C. D., Waring, J. P., and Hunter, J. G. Clinical and histologic follow-up after antireflux surgery for Barrett's esophagus. Journal of gastrointestinal surgery 2002; 6 (4): 532-539.
157. Huang, M.-T., Lai, I.-R., Wei, P.-L., Wu, C.-C., and Lee, W.-J. Laparoscopic nissen fundoplication for reflux esophagitis: The initial experience. Formosan Journal of Surgery 2000; 33 (2): 66-71.
158. Teodori, L., Gohde, W., Persiani, M., Ferrario, F., Danesi, D. T., Scarpignato, C., Di, Tondo U., Alo, P., and Capurso, L. DNA/protein flow cytometry as a predictive marker of malignancy in dysplasia-free barrett's esophagus: Thirteen-year follow-up study on a cohort of patients. Communications in Clinical Cytometry 1998; 34 (6): 257-263.
159. Drewitz, D. J., Sampliner, R. E., and Garewal, H. S. The incidence of adenocarcinoma in Barrett's esophagus: a prospective study of 170 patients followed 4.8 years. The American journal of gastroenterology 1997; 92 (2): 212-215.
160. Weston, A. P., Sharma, P., Mathur, S., Banerjee, S., Jafri, A. K., Cherian, R., McGregor, D., Hassanein, R. S., and Hall, M. Risk stratification of Barrett's esophagus: Updated prospective multivariate analysis. American Journal of Gastroenterology 2004; 99 (9): 1657-1666.
161. Steevens, Jessie, Schouten, Leo J., Driessen, Ann L. C., Huysentruyt, Clement J. R., Keulemans, Yolande C. A., Goldbohm, R. Alexandra, and van den Brandt, Piet A. A prospective cohort study on overweight, smoking, alcohol consumption, and risk of Barrett's esophagus. Cancer epidemiology, biomarkers & prevention: a publication of the American Association for Cancer Research, cosponsored by the American Society of Preventive Oncology 2011; 20 (2): 345-358.
162. Lamberts, R., Brunner, G., and Solcia, E. Effects of very long (up to 10 years) proton pump blockade on human gastric mucosa. Digestion 2001; 64 (4): 205-213.
163. Miholic, Johannes, Hafez, Joumanah, Lenglinger, Johannes, Wrba, Fritz, Wischin, Christiane, Schutz, Katrin, and Hudec, Marcus Hiatal hernia, Barrett's esophagus, and long-term symptom control after laparoscopic fundoplication for gastroesophageal reflux. Surgical endoscopy 2012; 26 (11): 3225-3231.
164. Tekin, Koray, Toydemir, Toygar, and Yerdel, Mehmet Ali Is laparoscopic antireflux surgery safe and effective in obese patients? Surgical endoscopy 2012; 26 (1): 86-95.
165. Nocon, Marc, Labenz, Joachim, Jaspersen, Daniel, Leodolter, Andreas, Meyer-Sabellek, Wolfgang, Stolte, Manfred, Vieth, Michael, Lind, Tore, Malfertheiner, Peter, and Willich, Stefan N. Nighttime heartburn in patients with gastroesophageal reflux disease under routine care. Digestion 2008; 77 (2): 69-72.
166. Marcotullio, Dario, Magliulo, Giuseppe, and Pezone, Tiziana Reinke's edema and risk factors: clinical and histopathologic aspects. American journal of otolaryngology 2002; 23 (2): 81-84.
167. Ye, W., Chow, W. H., Lagergren, J., Yin, L., and Nyren, O. Risk of adenocarcinomas of the esophagus and gastric cardia in patients with gastroesophageal reflux diseases and after antireflux surgery. Gastroenterology 2001; 121 (6): 1286-1293.
168. Ye, W., Chow, W. H., Lagergren, J., Boffetta, P., Boman, G., Adami, H. O., and Nyren, O. Risk of adenocarcinomas of the oesophagus and gastric cardia in patients hospitalized for asthma. British Journal of Cancer 2001; 85 (9): 1317-1321.
169. Balsiger, B. M., Murr, M. M., Mai, J., and Sarr, M. G. Gastroesophageal reflux after intact vertical banded gastroplasty: correction by conversion to Roux-en-Y gastric bypass. Journal of gastrointestinal surgery 2000; 4 (3): 276-281.
170. Di Simone, M. P., Felice, V., D'Errico, A., Bassi, F., D'Ovidio, F., Brusori, S., and Mattioli, S. Onset timing of delayed complications and criteria of follow-up after operation for esophageal achalasia. The Annals of thoracic surgery 1996; 61 (4): 1106-1.
171. Caygill, C. P., Hill, M. J., Kirkham, J. S., and Northfield, T. C. Oesophageal cancer in gastric surgery patients. The Italian journal of gastroenterology 1993; 25 (4): 168-170.
172. Moghissi, K., Sharpe, D. A., and Pender, D. Adenocarcinoma and Barrett's oesophagus. A clinico-pathological study. European journal of cardio-thoracic surgery 1993; 7 (3): 126-131.
173. Fekete, F., Gayet, B., Deslandes, M., and Dubertret, M. [Reoperations for failure of gastroesophageal reflux surgery. Apropos of fifty reoperations]. Annales de chirurgie 1992; 46 (1): 44-50.
174. Pearson, F. G., Cooper, J. D., Patterson, G. A., Ramirez, J., and Todd, T. R. Gastroplasty and fundoplication for complex reflux problems. Long-term results. Annals of surgery 1987; 206 (4): 473-481.
175. Januszewicz, W., Kaminski, M. F., Wieszczy, P., Wronska, E., Bielasik, A., Wojciechowska, U., Didkowska, J., Orlowska, J., and Regula, J. Adenocarcinoma risk in patients registered with polish barrett's oesophagus registry. Diseases of the Esophagus 2017; 30(1):1-6.
176. Khan, A. A., Shah, S. W. H., Alam, A., Butt, A. K., and Shafqat, F. Sixteen years follow up of achalasia: A prospective study of graded dilatation using Rigiflex ballon. Diseases of the Esophagus 2005; 18 (1): 41-45.
177. Singh, P., Indaram, A., Greenberg, R., Visvalingam, V., and Bank, S. Long term omeprazole therapy for reflux esophagitis: Follow-up in serum gastrin levels, EC cell hyperplasia and neoplasia. World journal of gastroenterology 2000; 6 (6): 789-792.
178. Rebecchi, F., Allaix, M. E., Ugliono, E., Giaccone, C., Toppino, M., and Morino, M. Increased Esophageal Exposure to Weakly Acidic Reflux 5 Years After Laparoscopic Roux-en-Y Gastric Bypass. Annals of surgery 2016; 264 (5): 871-877.
179. Bradley, D. D., Louie, B. E., Chen, J., Aye, R. W., McMahon, R., and Farivar, A. S. The effect of concurrent esophageal pathology on bariatric surgical planning. Journal of gastrointestinal surgery 2015; 19 (1): 111-115.
180. Gorodner, V., Buxhoeveden, R., Clemente, G., Sanchez, C., Caro, L., and Grigaites, A. Barrett's esophagus after Roux-en-Y gastric bypass: does regression occur? Surgical endoscopy 2017; 31 (4): 1849-1854.
181. Sharma, P., Morales, T. G., Bhattacharyya, A., Garewal, H. S., and Sampliner, R. E. Dysplasia in short-segment Barrett's esophagus: a prospective 3-year follow-up. The American journal of gastroenterology 1997; 92 (11): 2012-2016.
182. Graham, D. Y., Schwartz, J. T., Cain, G. D., and Gyorkey, F. Prospective evaluation of biopsy number in the diagnosis of esophageal and gastric carcinoma. Gastroenterology 1982; 82 (2): 228-231.
183. Mortensen, M. B., Fristrup, C. W., Ainsworth, A. P., Pless, T., Nielsen, H. O., and Hovendal, C. Combined preoperative endoscopic and laparoscopic ultrasonography for prediction of R0 resection in upper gastrointestinal tract cancer. The British journal of surgery 2006; 93 (6): 720-725.
184. Mariette, C., Balon, J. M., Maunoury, V., Taillier, G., Van, Seuningen, I, and Triboulet, J. P. Value of endoscopic ultrasonography as a predictor of long-term survival in oesophageal carcinoma. The British journal of surgery 2003; 90 (11): 1367-1372.
185. Menon, K. V. and Dehn, T. C. Multiport staging laparoscopy in esophageal and cardiac carcinoma. Diseases of the esophagus 2003; 16 (4): 295-300.
186. Lin, Emery C., Holub, Jennifer, Lieberman, David, and Hur, Chin. Low Prevalence of Suspected Barrett's Esophagus in Patients with Gastroesophageal Reflux Disease Without Alarm Symptoms. Clinical gastroenterology and hepatology 2018.
187. Ahmed Osman, Heba, Aly, Sanaa S., Mahmoud, Hasan S., Ahmed, Eman H., Salah Eldin, Eman M., Abdelrahim, Eman A., El Masry, Muhammad A., Herdan, Rania A., and Hassan, Mohammed H. Effect of Acid Suppression on Peripheral T-Lymphocyte Subsets and Immunohistochemical Esophageal Mucosal Changes in Patients With Gastroesophageal Reflux Disease. Journal of clinical gastroenterology 2018.
188. Stasek, Martin, Aujesky, Rene, Vrba, Radek, Lovecek, Martin, Chudacek, Josef, Janda, Petr, Gregorik, Michal, Vomackova, Katherine, Neoral, Cestmir, and Klos, Dusan. Indications and benefits of intraoperative esophagogastroduodenoscopy. Wideochirurgia i inne techniki maloinwazyjne = Videosurgery and other miniinvasive techniques 2018. 13 (2) 164-175.
189. Stasyshyn, Andriy R. Gastroesophageal reflux disease: the results of videolaparoscopic fundoplication at five years after surgery. Wiadomosci lekarskie (Warsaw, Poland: 1960) 2017. 70 (4) 751-753.
190. Epstein, Jeremy A., Cosby, Hilary, Falk, Gary W., Khashab, Mouen A., Kiesslich, Ralf, Montgomery, Elizabeth A., Wang, Jean S., and Canto, Marcia Irene. Columnar islands in Barrett's esophagus: Do they impact Prague C&M criteria and dysplasia grade? Journal of gastroenterology and hepatology 2017. 32 (9) 1598-1603.
191. Wolter, Stefan, Dupree, Anna, Miro, Jameel, Schroeder, Cornelia, Jansen, Marie Isabelle, Schulze-Zur-Wiesch, Clarissa, Groth, Stefan, Izbicki, Jakob, Mann, Oliver, and Busch, Philipp. Upper Gastrointestinal Endoscopy prior to Bariatric Surgery-Mandatory or Expendable? An Analysis of 801 Cases. Obesity surgery 2017. 27 (8) 1938-1943.
192. Abou, Hussein B., Khammas, A., Shokr, M., Majid, M., Sandal, M., Awadhi, S. A., Mazrouei, A. A., and Badri, F. Role of routine upper endoscopy before bariatric surgery in the Middle East population: A review of 1278 patients. Endoscopy International Open 2018. 6 (10) E1171-E1176.
193. Soricelli, E., Casella, G., Baglio, G., Maselli, R., Ernesti, I., and Genco, A. Lack of correlation between gastroesophageal reflux disease symptoms and esophageal lesions after sleeve gastrectomy. Surgery for Obesity and Related Diseases 2018. 14 (6) 751-756.

### Study design: Cross-sectional (including diagnostic test studies) (n=316)

1. Realdon, Stefano, Antonello, Alessandro, Arcidiacono, Diletta, Dassie, Elisa, Cavallin, Francesco, Fassan, Matteo, Nardi, Maria Teresa, Alberti, Alfredo, Rugge, Massimo, and Battaglia, Giorgio Adherence to WCRF/AICR lifestyle recommendations for cancer prevention and the risk of Barrett's esophagus onset and evolution to esophageal adenocarcinoma: results from a pilot study in a high-risk population. European journal of nutrition 2016; 55 (4): 1563-1571.
2. Menezes, A., Tierney, A., Yang, Y. X., Forde, K. A., Bewtra, M., Metz, D., Ginsberg, G. G., and Falk, G. W. Adherence to the 2011 American Gastroenterological Association medical position statement for the diagnosis and management of Barrett's esophagus. Diseases of the esophagus 2015; 28 (6): 538-546.
3. Jacobson, B. C. and Gerson, L. B. The inaccuracy of ICD-9-CM Code 530.2 for identifying patients with Barrett's esophagus. Diseases of the esophagus 2008; 21 (5): 452-456.
4. Chandrasoma, Parakrama T., Der, Roger, Ma, Yanling, Peters, Jeffrey, and Demeester, Tom Histologic classification of patients based on mapping biopsies of the gastroesophageal junction. The American journal of surgical pathology 2003; 27 (7): 929-936.
5. Rossi, Angelo, Bersani, Gianluca, Ricci, Giorgio, Defabritiis, Giovanni, Pollino, Valeria, Suzzi, Alessandra, Gorini, Beatrice, and Alvisi, Vittorio ASGE guidelines for the appropriate use of upper endoscopy: association with endoscopic findings. Gastrointestinal endoscopy 2002; 56 (5): 714-719.
6. Westbrook, J. I., McIntosh, J. H., and Duggan, J. M. Accuracy of provisional diagnoses of dyspepsia in patients undergoing first endoscopy. Gastrointestinal endoscopy 2001; 53 (3): 283-288.
7. Desilets, D. J., Nathanson, B. H., and Navab, F. Barrett's esophagus in practice: Gender and screening issues. Journal of Men's Health 2014; 11 (4): 177-182.
8. Langner, C., Wolf, E.-M., Plieschnegger, W., Geppert, M., Wigginghaus, B., Hoss, G. M., Eherer, A., Schneider, N. I., Rehak, P., and Vieth, M. Multilayered epithelium at the gastroesophageal junction is a marker of gastroesophageal reflux disease: Data from a prospective Central European multicenter study (histoGERD trial). Virchows Archiv 2014; 464 (4): 409-417.
9. Johanson, J. F., Frakes, J., and Eisen, D. Computer-assisted analysis of abrasive transepithelial brush biopsies increases the effectiveness of esophageal screening: A multicenter prospective clinical trial by the endocdx collaborative group. Digestive Diseases and Sciences 2011; 56 (3): 767-772.
10. Salem, S. B., Kushner, Y., Marcus, V., Mayrand, S., Fallone, C. A., and Barkun, A. N. The potential impact of contemporary developments in the management of patients with gastroesophageal reflux disease undergoing an initial gastroscopy. Canadian Journal of Gastroenterology 2009; 23 (2): 99-104.
11. De Jonge, P. J. F., Van Eijck, B. C., Geldof, H., Bekkering, F. C., Essink-Bot, M.-L., Polinder, S., Kuipers, E. J., and Siersema, P. D. Capsule endoscopy for the detection of oesophageal mucosal disorders: A comparison of two different ingestion protocols. Scandinavian Journal of Gastroenterology 2008; 43 (7): 870-877.
12. Arts, J., Eisendrath, P., Deviere, J., and Tack, J. Empirical therapy for symptomatic gastroesophageal reflux disease in primary care: Determinants of efficacy. Digestion 2007; 76 (3-4): 207-214.
13. Anagnostopoulos, G. K., Pick, B., Cunliffe, R., Fortun, P., Kaye, P., and Ragunath, K. Barrett's esophagus specialist clinic: What difference can it make? Diseases of the Esophagus 2006; 19 (2): 84-87.
14. Tack, J., Koek, G., Demedts, I., Sifrim, D., and Janssens, J. Gastroesophageal reflux disease poorly responsive to single-dose proton pump inhibitors in patients without Barrett's esophagus: Acid reflux, bile reflux, or both? American Journal of Gastroenterology 2004; 99 (6): 981-988.
15. Peghini, M., Rajaonarison, P., Pecarrere, J.-L., Razafindramboa, H., Andriantsoavina, H., Rakotomalala, M., and Ramarokoto, N. Madagascar: Esophago-gastro-duodenoscopy. Analysis of 12000 procedures and problems in tropical areas. Medecine Tropicale 1996; 56 (1): 89-94.
16. Chavalitdhamrong, D., Chen, G. C., Roth, B. E., Goltzer, O., Sul, J., and Jutabha, R. Esophageal capsule endoscopy for evaluation of patients with chronic gastroesophageal reflux symptoms: findings and its image quality. Diseases of the esophagus 2011; 24 (5): 295-298.
17. Ma, Xiuqiang, Xu, Qin, Zheng, Yanling, Zhao, Yanfang, Lu, Jian, Wang, Rui, Li, Zhaoshen, Zou, Duowu, and He, Jia Prevalence of esophageal eosinophilia and eosinophilic esophagitis in adults: a population-based endoscopic study in Shanghai, China. Digestive Diseases and Sciences 2015; 60 (6): 1716-1723.
18. Langner, Cord, Schneider, Nora I., Plieschnegger, Wolfgang, Schmack, Bertram, Bordel, Hartmut, Hofler, Bernd, Eherer, Andreas J., Wolf, Eva Maria, Rehak, Peter, and Vieth, Michael Cardiac mucosa at the gastro-oesophageal junction: indicator of gastro-oesophageal reflux disease? Data from a prospective central European multicentre study on histological and endoscopic diagnosis of oesophagitis (histoGERD trial). Histopathology 2014; 65 (1): 81-89.
19. Schneider, Nora I., Plieschnegger, Wolfgang, Geppert, Michael, Wigginghaus, Bernd, Hoss, Gabriele M., Eherer, Andreas, Wolf, Eva Maria, Rehak, Peter, Vieth, Michael, and Langner, Cord Pancreatic acinar cells--a normal finding at the gastroesophageal junction? Data from a prospective Central European multicenter study. Virchows Archiv: an international journal of pathology 2013; 463 (5): 643-650.
20. Ludvigsson, Jonas F., Aro, Pertti, Walker, Marjorie M., Vieth, Michael, Agreus, Lars, Talley, Nicholas J., Murray, Joseph A., and Ronkainen, Jukka Celiac disease, eosinophilic esophagitis and gastroesophageal reflux disease, an adult population-based study. Scandinavian Journal of Gastroenterology 2013; 48 (7): 808-814.
21. Galmiche, Jean Paul, Sacher-Huvelin, Sylvie, Coron, Emmanuel, Cholet, Franck, Soussan, Emmanuel Ben, Sebille, Veronique, Filoche, Bernard, d'Abrigeon, Gilles, Antonietti, Michel, Robaszkiewicz, Michel, Le Rhun, Marc, and Ducrotte, Philippe Screening for esophagitis and Barrett's esophagus with wireless esophageal capsule endoscopy: a multicenter prospective trial in patients with reflux symptoms. The American journal of gastroenterology 2008; 103 (3): 538-545.
22. Delvaux, M., Papanikolaou, I. S., Fassler, I., Pohl, H., Voderholzer, W., Rosch, T., and Gay, G. Esophageal capsule endoscopy in patients with suspected esophageal disease: double blinded comparison with esophagogastroduodenoscopy and assessment of interobserver variability. Endoscopy 2008; 40 (1): 16-22.
23. Bohmer, C. J., Niezen-de Boer, M. C., Klinkenberg-Knol, E. C., Deville, W. L., Nadorp, J. H., and Meuwissen, S. G. The prevalence of gastroesophageal reflux disease in institutionalized intellectually disabled individuals. The American journal of gastroenterology 1999; 94 (3): 804-810.
24. Kim, S. L., Wo, J. M., Hunter, J. G., Davis, L. P., and Waring, J. P. The prevalence of intestinal metaplasia in patients with and without peptic strictures. The American journal of gastroenterology 1998; 93 (1): 53-55.
25. Trudgill, N. J., Suvarna, S. K., Kapur, K. C., and Riley, S. A. Intestinal metaplasia at the squamocolumnar junction in patients attending for diagnostic gastroscopy. Gut 1997; 41 (5): 585-589.
26. Takeshita, E., Sakata, Y., Hara, M., Akutagawa, K., Sakata, N., Endo, H., Ohyama, T., Matsunaga, K., Yoshioka, T., Kawakubo, H., Tanaka, Y., Shirai, S., Ito, Y., Tsuruoka, N., Iwakiri, R., Kusano, M., and Fujimoto, K. Higher Frequency of Reflux Symptoms and Acid-Related Dyspepsia in Women than Men Regardless of Endoscopic Esophagitis: Analysis of 3,505 Japanese Subjects Undergoing Medical Health Checkups. Digestion 2016; 93 (4): 266-271.
27. Roshandel, G., Khoshnia, M., Sotoudeh, M., Merat, S., Etemadi, A., Nickmanesh, A., Norouzi, A., Pourshams, A., Poustchi, H., Semnani, S., Ghasemi-Kebria, F., Noorbakhsh, R., Abnet, C., Dawsey, S. M., and Malekzadeh, R. Endoscopic screening for precancerous lesions of the esophagus in a high risk area in northern Iran. Archives of Iranian Medicine 2014; 17 (4): 246-252.
28. Liu, N., Ando, T., Ishiguro, K., Maeda, O., Watanabe, O., Funasaka, K., Nakamura, M., Miyahara, R., Ohmiya, N., and Goto, H. Characterization of bacterial biota in the distal esophagus of Japanese patients with reflux esophagitis and Barrett's esophagus. BMC Infectious Diseases 2013; 13(1): 130
29. Siwiec, R. M., Dua, K., Surapaneni, S. N., Hafeezullah, M., Massey, B., and Shaker, R. Unsedated transnasal endoscopy with ultrathin endoscope as a screening tool for research studies. Laryngoscope 2012; 122 (8): 1719-1723.
30. Rosztoczy, A., Izbeki, F., Roka, R., Nemeth, I., Gecse, K., Vadaszi, K., Kadar, J., Vetro, E., Tiszlavicz, L., and Wittmann, T. The evaluation of oesophageal function in patients with different types of oesophageal metaplasia. Digestion 2011; 84 (4): 273-280.
31. Lin, O. S., Schembre, D. B., Mergener, K., Spaulding, W., Lomah, N., Ayub, K., Brandabur, J. J., Bredfeldt, J., Drennan, F., Gluck, M., Jiranek, G. C., McCormick, S. E., Patterson, D., and Kozarek, R. A. Blinded comparison of esophageal capsule endoscopy versus conventional endoscopy for a diagnosis of Barrett's esophagus in patients with chronic gastroesophageal reflux. Gastrointestinal endoscopy 2007; 65 (4): 577-583.
32. Eisen, G. M., Eliakim, R., Zaman, A., Schwartz, J., Faigel, D., Rondonotti, E., Villa, F., Weizman, E., Yassin, K., and deFranchis, R. The accuracy of PillCam ESO capsule endoscopy versus conventional upper endoscopy for the diagnosis of esophageal varices: A prospective three-center pilot study. Endoscopy 2006; 38 (1): 31-35.
33. Ronkainen, J., Aro, P., Storskrubb, T., Johansson, S.-E., Lind, T., Bolling-Sternevald, E., Vieth, M., Stolte, M., Talley, N. J., and Agreus, L. Prevalence of Barrett's esophagus in the general population: An endoscopic study. Gastroenterology 2005; 129 (6): 1825-1831.
34. Bolling-Sternevald, E., Carlsson, R., Aalykke, C., Wilson, B. V. L., Junghard, O., Glise, H., and Lauritsen, K. Self-administered symptom questionnaires in patients with dyspepsia and their yield in discriminating between endoscopic diagnoses. Digestive Diseases 2002; 20 (2): 191-198.
35. Van Sandick, J. W., Van Lanschot, J. J. B., Van, Felius L., Haringsma, J., Tytgat, G. N. J., Dekker, W., Drillenburg, P., Offerhaus, G. J. A., and Ten Kate, F. J. W. Intestinal metaplasia of the esophagus or esophagogastric junction: Evidence of distinct clinical, pathologic, and histochemical staining features. American Journal of Clinical Pathology 2002; 117 (1): 117-125.
36. Smythe, A., Bird, N. C., Troy, G. P., Globe, J., and Johnson, A. G. Effect of cisapride on oesophageal motility and duodenogastro-oesophageal reflux in patients with Barrett's oesophagus. European Journal of Gastroenterology and Hepatology 1997; 9 (12): 1149-1153.
37. Chernin, M. M., Amberg, J. R., and Kogan, F. J. Efficacy of radiologic studies in the detection of Barrett's esophagus. American Journal of Roentgenology 1986; 147 (2): 257-260.
38. Cui, R., Zhang, H., Zhou, L., Lu, J., Xue, Y., Wang, Y., Yan, X., Lin, L., and Lin, S. Diagnostic value of dilated intercellular space and histopathologic scores in gastroesophageal reflux disease. Diseases of the esophagus 2015; 28 (6): 530-537.
39. Nguyen, Theresa H., Thrift, Aaron P., Ramsey, David, Green, Linda, Shaib, Yasser H., Graham, David Y., and El-Serag, Hashem B. Risk factors for Barrett's esophagus compared between African Americans and non-Hispanic Whites. The American journal of gastroenterology 2014; 109 (12): 1870-1880.
40. Murray, Iain A., Palmer, Joanne, Waters, Carolyn, and Dalton, Harry R. Predictive value of symptoms and demographics in diagnosing malignancy or peptic stricture. World journal of gastroenterology 2012; 18 (32): 4357-4362.
41. Mazzadi, Sergio Angel, Garcia, Alfredo Omar, Salis, Graciela Beatriz, and Chiocca, Juan Carlos Peptic esophageal stricture: a report from Argentina. Diseases of the esophagus 2004; 17 (1): 63-66.
42. Vieth, M., Masoud, B., Meining, A., and Stolte, M. Helicobacter pylori infection: protection against Barrett's mucosa and neoplasia? Digestion 2000; 62 (4): 225-231.
43. Teriaky, A., Alnasser, A., Mclean, C., Gregor, J., and Yan, B. The Utility of Endoscopic Biopsies in Patients with Normal Upper Endoscopy. Canadian Journal of Gastroenterology and Hepatology 2016.
44. Dore, M. P., Pes, G. M., Bassotti, G., Farina, M. A., Marras, G., and Graham, D. Y. Risk factors for erosive and non-erosive gastroesophageal reflux disease and Barrett's esophagus in Nothern Sardinia. Scandinavian Journal of Gastroenterology 2016;51(11):1281-7.
45. Bhatt, A., Parsi, M. A., Stevens, T., Gabbard, S., Kumaravel, A., Jang, S., Grove, D., Lopez, R., Murthy, S., Vargo, J. J., and Dweik, R. Volatile organic compounds in plasma for the diagnosis of esophageal adenocarcinoma: A pilot study. Gastrointestinal endoscopy 2016; 84(4): 597-603.
46. Shiota, S., El-Serag, H. B., and Thrift, A. P. Premature Birth and Large for Gestational Age Are Associated with Risk of Barrett's Esophagus in Adults. Digestive Diseases and Sciences 2016; 61 (4): 1139-1147.
47. Gyedu, A. and Yorke, J. Upper gastrointestinal endoscopy in the patient population of Kumasi, Ghana: Indications and findings. Pan African Medical Journal 2014; 18: 327
48. Olmez, S., Aslan, M., Erten, R., Sayar, S., and Bayram, I. The prevalence of gastric intestinal metaplasia and distribution of helicobacter pylori infection, atrophy, dysplasia, and cancer in its subtypes. Gastroenterology Research and Practice 2015.
49. Gado, A., Ebeid, B., Abdelmohsen, A., and Axon, A. Prevalence of reflux esophagitis among patients undergoing endoscopy in a secondary referral hospital in Giza, Egypt. Alexandria Journal of Medicine 2015; 51 (2): 89-94.
50. Pascarenco, O. D., Boeriu, A., Mocan, S., Pascarenco, G., Drasoveanu, S., Galeanu, M., and Dobru, D. Barrett's esophagus and intestinal metaplasia of gastric cardia: Prevalence, clinical, endoscopic and histological features. Journal of Gastrointestinal and Liver Diseases 2014; 23 (1): 19-25.
51. Vela, M. F., Kramer, J. R., Richardson, P. A., Dodge, R., and El-Serag, H. B. Poor sleep quality and obstructive sleep apnea in patients with GERD and Barrett's esophagus. Neurogastroenterology and Motility 2014; 26 (3): 346-352.
52. Rubenstein, J. H., Morgenstern, H., Appelman, H., Scheiman, J., Schoenfeld, P., McMahon, L. F., Metko, V., Near, E., Kellenberg, J., Kalish, T., and Inadomi, J. M. Prediction of barrett's esophagus among men. American Journal of Gastroenterology 2013; 108 (3): 353-362.
53. Keyashian, K., Hua, V., Narsinh, K., Kline, M., Chandrasoma, P. T., and Kim, J. J. Barrett's esophagus in Latinos undergoing endoscopy for gastroesophageal reflux disease symptoms. Diseases of the Esophagus 2013; 26 (1): 44-49.
54. Khamechian, T., Alizargar, J., and Mazoochi, T. The prevalence of Barrett's esophagus in outpatients with dyspepsia in Shaheed Beheshti Hospital of Kashan. Iranian Journal of Medical Sciences 2013; 38 (3): 263-266.
55. Mussetto, A., Manno, M., Fuccio, L., and Conigliaro, R. Screening for Barrett's oesophagus with oesophageal capsule endoscopy in first-degree relatives of patients affected by Barrett's oesophagus: Results of a pilot study. Arab Journal of Gastroenterology 2013; 14 (2): 51-54.
56. Coban, S., Ormeci, N., Savas, B., Ekiz, F., Ensari, A., Kuzu, I., and Palabiyikoglu, M. Evaluation of Barrett's esophagus with CK7, CK20, p53, Ki67, and COX2 expressions using chromoendoscopical examination. Diseases of the Esophagus 2013; 26 (2): 189-196.
57. Balasubramanian, G., Gupta, N., Giacchino, M., Singh, M., Kanakadandi, V., Gaddam, S., Wani, S. B., Higbee, A. D., Rastogi, A., Bansal, A., and Sharma, P. Cigarette smoking is a modifiable risk factor for Barrett's oesophagus. United European Gastroenterology Journal 2013; 1 (6): 430-437.
58. Balasubramanian, G., Singh, M., Gupta, N., Gaddam, S., Giacchino, M., Wani, S. B., Moloney, B., Higbee, A. D., Rastogi, A., Bansal, A., and Sharma, P. Prevalence and predictors of columnar lined esophagus in Gastroesophageal Reflux Disease (GERD) patients undergoing upper endoscopy. American Journal of Gastroenterology 2012; 107 (11): 1655-1661.
59. Mathew, P., Joshi, A. S., Shukla, A., and Bhatia, S. J. Risk factors for Barrett's esophagus in Indian patients with gastroesophageal reflux disease. Journal of Gastroenterology and Hepatology (Australia) 2011; 26 (7): 1151-1156.
60. Nakos, A., Kouklakis, G., Pitiakoudis, M., Zezos, P., Efraimidou, E., Giatromanolaki, A., Polychronidis, A., Liratzopoulos, N., Sivridis, E., and Simopoulos, K. The histological and immunohistochemical aspects of bile reflux in patients with gastroesophageal reflux disease. Gastroenterology Research and Practice 2011.
61. Bamanikar, A. A., Diwan, A. G., Benoj, D. E., and Bamanikar, S. A. Barrett's metaplasia in indian obese male patients with gastroesophageal reflux disease. Journal of the Indian Medical Association 2011; 109 (7): 483-484.
62. Savarino, E., Gemignani, L., Pohl, D., Zentilin, P., Dulbecco, P., Assandri, L., Marabotto, E., Bonfanti, D., Inferrera, S., Fazio, V., Malesci, A., Tutuian, R., and Savarino, V. Oesophageal motility and bolus transit abnormalities increase in parallel with the severity of gastro-oesophageal reflux disease. Alimentary Pharmacology and Therapeutics 2011; 34 (4): 476-486.
63. Menon, S., Jayasena, H., Nightingale, P., and Trudgill, N. J. Influence of age and sex on endoscopic findings of gastrooesophageal reflux disease: An endoscopy database study. European Journal of Gastroenterology and Hepatology 2011; 23 (5): 389-395.
64. Chen, Z., Thompson, S. K., Jamieson, G. G., Devitt, P. G., and Watson, D. I. Effect of sex on symptoms associated with gastroesophageal reflux. Archives of Surgery 2011; 146 (10): 1164-1169.
65. Navarathne, N. M. M., Abeysuriya, V., Ileperuma, A., and Thoufeek, U. L. Endoscopic observations around the gastroesophageal junction in patients with symptomatic gastroesophageal reflux disease in South Asia. Indian Journal of Gastroenterology 2010; 29 (5): 184-186.
66. Alvaro-Villegas, J. C., Sobrino-Cossio, S., Hernandez-Guerrero, A., Alonso-Larraga, J. O., de-la-Mora-Levy, J. G., Molina-Cruz, A., Dominguez-Malagon, H. R., and Herrera-Esquivel, J. J. Dilated intercellular spaces in subtypes of gastroesophagic reflux disease. Revista Espanola de Enfermedades Digestivas 2010; 102 (5): 302-307.
67. Berndt, U., Philipsen, L., Bartsch, S., Hu, Y., Rocken, C., Bertram, W., Hammerle, M., Rosch, T., and Sturm, A. Comparative Multi-Epitope-Ligand-Cartography reveals essential immunological alterations in Barrett's metaplasia and esophageal adenocarcinoma. Molecular Cancer 2010; 9(1): 177
68. Park, J.-J., Kim, H. J., Chung, M. G., Park, S. M., Baik, G. H., Nah, B. K., Nam, S. Y., Seo, K. S., Ko, B. S., Jang, J.-Y., Kim, B. G., Kim, J. W., Choi, Y. S., Joo, M. K., Kim, J. I., Cho, M.-Y., Kim, N., Park, S.-H., Jung, H. C., and Chung, I.-S. The prevalence of and risk factors for barrett's esophagus in a Korean population: A nationwide multicenter prospective study. Journal of Clinical Gastroenterology 2009; 43 (10): 907-914.
69. Glickman, J. N., Spechler, S. J., Souza, R. F., Lunsford, T., Lee, E., and Odze, R. D. Multilayered epithelium in mucosal biopsy specimens from the gastroesophageal junction region is a histologic marker of gastroesophageal reflux disease. American Journal of Surgical Pathology 2009; 33 (6): 818-825.
70. Chang, C.-Y., Lee, Y.-C., Lee, C.-T., Tu, C.-H., Hwang, J.-C., Chiang, H., Tai, C.-M., Chiang, T.-H., Wu, M.-S., and Lin, J.-T. The application of prague C and M criteria in the diagnosis of barrett's esophagus in an ethnic chinese population. American Journal of Gastroenterology 2009; 104 (1): 13-20.
71. Fouad, Y. M., Makhlouf, M. M., Tawfik, H. M., El, Amin H., Ghany, W. A., and El-Khayat, H. R. Barrett's esophagus: Prevalence and risk factors in patients with chronic GERD in Upper Egypt. World journal of gastroenterology 2009; 15 (28): 3511-3515.
72. Dickman, R., Levi, Z., Vilkin, A., Zvidi, I., and Niv, Y. Predictors of specialized intestinal metaplasia in patients with an incidental irregular Z line. European Journal of Gastroenterology and Hepatology 2010; 22 (2): 135-138.
73. Rubenstein, J. H., Mattek, N., and Eisen, G. Age- and sex-specific yield of Barrett's esophagus by endoscopy indication. Gastrointestinal endoscopy 2010; 71 (1): 21-27.
74. Chang, Y., Liu, B., Liu, G.-S., Wang, T., and Gong, J. Short-segment Barrett's esophagus and cardia intestinal metaplasia: A comparative analysis. World journal of gastroenterology 2010; 16 (48): 6151-6154.
75. Binato, M., Fagundes, R., Gurski, R., Meurer, L., and Edelweiss, M. I. Immunohistochemical overexpression of the p53 protein and Ki-67 (MIB-1) antigen in patients with GERD and chronic esophagitis. Applied Immunohistochemistry and Molecular Morphology 2010; 18 (3): 236-243.
76. Vega, K. J., Chisolm, S., and Jamal, M. M. Comparison of reflux esophagitis and its complications between African Americans and non-Hispanic whites. World journal of gastroenterology 2009; 15 (23): 2878-2881.
77. Gerson, L. B. and Banerjee, S. Screening for Barrett's esophagus in asymptomatic women. Gastrointestinal endoscopy 2009; 70 (5): 867-873.
78. Allameh, A., Rasmi, Y., Nasseri-Moghaddam, S., Tavangar, S. M., Sharifi, R., and Sadreddini, M. Immunohistochemical analysis of selected molecular markers in esophagus precancerous, adenocarcinoma and squamous cell carcinoma in Iranian subjects. Cancer Epidemiology 2009; 33 (1): 79-84.
79. Akagi, T., Ito, T., Kato, M., Jin, Z., Cheng, Y., Kan, T., Yamamoto, G., Olaru, A., Kawamata, N., Boult, J., Soukiasian, H. J., Miller, C. W., Ogawa, S., Meltzer, S. J., and Koeffler, H. P. Chromosomal abnormalities and novel disease-related regions in progression from Barrett's esophagus to esophageal adenocarcinoma. International Journal of Cancer 2009; 125 (10): 2349-2359.
80. El-Serag, H. B., Fitzgerald, S., and Richardson, P. The extent and determinants of prescribing and adherence with acid-reducing medications: A national claims database study. American Journal of Gastroenterology 2009; 104 (9): 2161-2167.
81. Boghratian, A. H., Hashemi, M. H., and Kabir, A. Gender-related differences in upper gastrointestinal endoscopic findings: An assessment of 4,700 cases from Iran. Journal of Gastrointestinal Cancer 2009; 40 (3-4): 83-90.
82. Breton, J., Gage, M. C., Hay, A. W., Keen, J. N., Wild, C. P., Donnellan, C., Findlay, J. B. C., and Hardie, L. J. Proteomic screening of a cell line model of esophageal carcinogenesis identifies cathepsin D and aldo-keto reductase 1C2 and 1B10 dysregulation in barrett's esophagus and esophageal adenocarcinoma. Journal of Proteome Research 2008; 7 (5): 1953-1962.
83. Hui, Y. Z., Zhang, X., Chen, X., Thomas, D., Hormi-Carver, K., Elder, F., Spechler, S. J., and Souza, R. F. Differences in activity and phosphorylation of MAPK enzymes in esophageal squamous cells of GERD patients with and without Barrett's esophagus. American Journal of Physiology - Gastrointestinal and Liver Physiology 2008; 295 (3): G470-G478.
84. Stairs, D. B., Nakagawa, H., Klein-Szanto, A., Mitchell, S. D., Silberg, D. G., Tobias, J. W., Lynch, J. P., and Rustgi, A. K. Cdx1 and c-Myc foster the initiation of transdifferentiation of the normal esophageal squamous epithelium toward Barrett's esophagus. PloS one 2008; 3 (10): e3534.
85. Gutschow, C. A., Bludau, M., Vallbohmer, D., Schroder, W., Bollschweiler, E., and Holscher, A. H. NERD, GERD, and Barrett's esophagus: Role of acid and non-acid reflux revisited with combined pH-impedance monitoring. Digestive Diseases and Sciences 2008; 53 (12): 3076-3081.
86. Akiyama, T., Inamori, M., Iida, H., Mawatari, H., Endo, H., Hosono, K., Yoneda, K., Fujita, K., Yoneda, M., Takahashi, H., Goto, A., Abe, Y., Kobayashi, N., Kubota, K., Saito, S., and Nakajima, A. Alcohol consumption is associated with an increased risk of erosive esophagitis and Barrett's epithelium in Japanese men. BMC Gastroenterology 2008; 8 (1):58
87. Tseng, P.-H., Lee, Y.-C., Chiu, H.-M., Huang, S.-P., Liao, W.-C., Chen, C.-C., Wang, H.-P., Wu, M.-S., and Lin, J.-T. Prevalence and clinical characteristics of Barrett's esophagus in a Chinese general population. Journal of Clinical Gastroenterology 2008; 42 (10): 1074-1079.
88. Kerkhof, M., Steyerberg, E. W., Kusters, J. G., Kulpers, E. J., and Slersema, P. D. Predicting presence of intestinal metaplasia and dysplasia in columnar-lined esophagus: A multivariate analysis. Endoscopy 2007; 39 (9): 772-778.
89. Sollano, J. D., Wong, S. N., Andal-Gamutan, T., Chan, M. M., Carpio, R. E., Tady, C. S., Ismael, A. E., Judan-Ruiz, E. A., Ang, V. N., Go, J. T., Lim, V. Y., Perez, J. Y., and Alvarez, S. Z. Erosive esophagitis in the Philippines: A comparison between two time periods. Journal of Gastroenterology and Hepatology (Australia) 2007; 22 (10): 1650-1655.
90. Agnese, V., Cabibi, D., Calcara, D., Terrasi, M., Pantuso, G., Fiorentino, E., Intrivici, C., Colucci, G., Aragona, F., Gebbia, N., Bazan, V., and Russo, A. Aurora-A overexpression as an early marker of reflux-related columnar mucosa and Barrett's oesophagus. Annals of Oncology 2007; 18 (SUPPL. 6): vi110-vi115.
91. Yuksel, I., Uskudar, O., Koklu, S., Basar, O., Gultuna, S., Unverdi, S., Ozturk, Z. A., Sengul, D., Turker, Arikok A., Yuksel, O., and Coban, S. Inlet patch: Associations with endoscopic findings in the upper gastrointestinal system. Scandinavian Journal of Gastroenterology 2008; 43 (8): 910-914.
92. Brillantino, A., Monaco, L., Schettino, M., Torelli, F., Izzo, G., Cosenza, A., Marano, L., and Di, Martino N. Prevalence of pathological duodenogastric reflux and the relationship between duodenogastric and duodenogastrooesophageal reflux in chronic gastrooesophageal reflux disease. European Journal of Gastroenterology and Hepatology 2008; 20 (12): 1136-1143.
93. Ruigomez, A., Rodriguez, L. A. G., Wallander, M.-A., Johansson, S., and Dent, J. Endoscopic findings in a cohort of newly diagnosed gastroesophageal reflux disease patients registered in a UK primary care database. Diseases of the Esophagus 2008; 21 (3): 251-256.
94. Sharma, P., Wani, S., Rastogi, A., Bansal, A., Higbee, A., Mathur, S., Esquivel, R., Camargo, L., and Sampliner, R. E. The diagnostic accuracy of esophageal capsule endoscopy in patients with gastroesophageal reflux disease and Barrett's esophagus: A blinded, prospective study. American Journal of Gastroenterology 2008; 103 (3): 525-532.
95. Pech, O., Petrone, M. C., Manner, H., Rabenstein, T., May, A., Pohl, J., and Ell, C. One-step chromoendoscopy and structure enhancement using balsamic vinegar for screening of Barrett's esophagus. Acta Gastro-Enterologica Belgica 2008; 71 (2): 243-245.
96. White, N., Gabril, M., Ejeckam, G., Mathews, M., Fardy, J., Kamel, F., Dore, J., and Yousef, G. M. Barrett's esophagus and cardiac intestinal metaplasia: Two conditions within the same spectrum. Canadian Journal of Gastroenterology 2008; 22 (4): 369-375.
97. Gilani, N., Gerkin, R. D., Ramirez, F. C., Hakim, S., and Randolph, A. C. Prevalence of Barrett's esophagus in patients with moderate to severe erosive esophagitis. World journal of gastroenterology 2008; 14 (22): 3518-3522.
98. Murray, L. J., Tully, O., Rudolph, D. S., Whitby, M., Valenzano, M. C., Mercogliano, G., Thornton, J. J., and Mullin, J. M. Absence of Na+/sugar cotransport activity in Barrett's metaplasia. World journal of gastroenterology 2008; 14 (9): 1365-1369.
99. Bayrakci, B., Kasap, E., Kitapcioglu, G., and Bor, S. Low prevalence of erosive esophagitis and Barrett esophagus in a tertiary referral center in Turkey. Turkish Journal of Gastroenterology 2008; 19 (3): 145-151.
100. Lao-Sirieix, P., Corovic, A., Jankowski, J., Lowe, A., Triadafilopoulos, G., and Fitzgerald, R. C. Physiological and molecular analysis of acid loading mechanisms in squamous and columnar-lined esophagus. Diseases of the Esophagus 2008; 21 (6): 529-538.
101. Nozu, T. and Komiyama, H. Clinical characteristics of asymptomatic esophagitis. Journal of gastroenterology 2008; 43 (1): 27-31.
102. Dickman, R., Mattek, N., Holub, J., Peters, D., and Fass, R. Prevalence of upper gastrointestinal tract findings in patients with noncardiac chest pain versus those with gastroesophageal reflux disease (GERD)-related symptoms: Results from a National Endoscopic Database. American Journal of Gastroenterology 2007; 102 (6): 1173-1179.
103. Johansson, J., Hakansson, H.-O., Mellblom, L., Kempas, A., Granath, F., Johansson, K.-E., and Nyren, O. Diagnosing Barrett's oesophagus: Factors related to agreement between endoscopy and histology. European Journal of Gastroenterology and Hepatology 2007; 19 (10): 870-877.
104. Chatzopoulos, D., Kyrgidis, A., Kountouras, J., Zavos, C., Molyvas, E., and Venizelos, I. Bax upregulation may provide a rationale for the low incidence of esophageal adenocarcinoma in a Greek cohort of patients with Barrett's esophagus. Hepato-gastroenterology 2007; 54 (75): 705-709.
105. Rezailashkajani, M., Roshandel, D., Shafaee, S., and Zali, M. R. High prevalence of reflux oesophagitis among upper endoscopies of Iranian patients. European Journal of Gastroenterology and Hepatology 2007; 19 (6): 499-506.
106. Halum, S. L., Postma, G. N., Bates, D. D., and Koufman, J. A. Incongruence between histologic and endoscopic diagnoses of Barrett's esophagus using transnasal esophagoscopy. Laryngoscope 2006; 116 (2): 303-306.
107. Vasavi, M., Ponnala, S., Gujjari, K., Boddu, P., Bharatula, R. S., Prasad, R., Ahuja, Y. R., and Hasan, Q. DNA methylation in esophageal diseases including cancer: Special reference to hMLH1 GENE promoter status. Tumori 2006; 92 (2): 155-162.
108. Lenglinger, J., Eisler, M., Ringhofer, C., Sedivy, R., Wrba, F., Zacherl, J., Cosentini, E. P., Prager, G., Haefuer, M., and Riegler, M. Video-endoscopy for evaluation of columnar lined esophagus in patients with gastroesophageal reflux disease. European Surgery - Acta Chirurgica Austriaca 2006; 38 (4): 293-301.
109. Lastraioli, E., Taddei, A., Messerini, L., Comin, C. E., Festini, M., Giannelli, M., Tomezzoli, A., Paglierani, M., Mugnai, G., De, Manzoni G., Bechi, P., and Arcangeli, A. hERG1 channels in human esophagus: Evidence for their aberrant expression in the malignant progression of Barrett's esophagus. Journal of Cellular Physiology 2006; 209 (2): 398-404.
110. Ali, I., Rafiee, P., Hogan, W. J., Jacob, H. J., Komorowski, R. A., Haasler, G. B., and Shaker, R. Dickkopf homologs in squamous mucosa of esophagitis patients are overexpressed compared with Barrett's patients and healthy controls. American Journal of Gastroenterology 2006; 101 (7): 1437-1448.
111. Grassi, A., Giannarelli, D., Iacopini, F., Paoluzi, P., Iannetti, A., Giovannelli, L., Efrati, C., Barberani, F., Giovannone, M., and Tosoni, M. Prevalence of intestinal metaplasia in the distal esophagus in patients endoscopically suspected for short Barrett's esophagus. Journal of Experimental and Clinical Cancer Research 2006; 25 (3): 297-302.
112. Pilotto, A., Franceschi, M., Leandro, G., Scarcelli, C., D'Ambrosio, L. P., Seripa, D., Perri, F., Niro, V., Paris, F., Andriulli, A., and Di, Mario F. Clinical features of reflux esophagitis in older people: A study of 840 consecutive patients. Journal of the American Geriatrics Society 2006; 54 (10): 1537-1542.
113. Kim, G. H., Kang, D. H., Song, G. A., Kim, T. O., Heo, J., Cho, M., Kim, J. S., Lee, B. J., and Wang, S. G. Gastroesophageal flap valve is associated with gastroesophageal and gastropharyngeal reflux. Journal of gastroenterology 2006; 41 (7): 654-661.
114. Shapiro, M., Green, C., Faybush, E. M., Esquivel, R. F., and Fass, R. The extent of oesophageal acid exposure overlap among the different gastro-oesophageal reflux disease groups. Alimentary Pharmacology and Therapeutics 2006; 23 (2): 321-329.
115. van Oijen, M. G. H., Josemanders, D. F. G. M., Laheij, R. J. F., van Rossum, L. G. M., Tan, A. C. I. T., and Jansen, J. B. M. J. Gastrointestinal disorders and symptoms: Does body mass index matter? Netherlands Journal of Medicine 2006; 64 (2): 45-49.
116. Marie, I., Ducrotte, P., Denis, P., Hellot, M. F., and Levesque, H. Oesophageal mucosal involvement in patients with systemic sclerosis receiving proton pump inhibitor therapy. Alimentary Pharmacology and Therapeutics 2006; 24 (11-12): 1593-1601.
117. Ang, T.-L., Fock, K.-M., Ng, T.-M., Teo, E.-K., Chua, T.-S., and Tan, J. A comparison of the clinical, demographic and psychiatric profiles among patients with erosive and non-erosive reflux disease in a multi-ethnic Asian country. World journal of gastroenterology 2005; 11 (23): 3558-3561.
118. Bretagne, J. F., Rey, J.-F., Caekaert, A., and Barthelemy, P. Routine management of gastro-oesophageal reflux disease by gastroenterologists in France: A prospective observational study. Digestive and Liver Disease 2005; 37 (8): 566-570.
119. Voutilainen, M., Mantynen, T., Mauranen, K., Kunnamo, I., and Juhola, M. Is it possible to reduce endoscopy workload using age, alarm symptoms and H. pylori as predictors of peptic ulcer and oesophagogastric cancers? Digestive and Liver Disease 2005; 37 (7): 526-532.
120. Cantu, P., Savojardo, D., Carmagnola, S., and Penagini, R. Impact of referral for gastro-oesophageal reflux disease on the workload of an academic Gastroenterology Unit. Digestive and Liver Disease 2005; 37 (10): 735-740.
121. Bafandeh, Y., Esmaili, H., and Aharizad, S. Endoscopic and histologic findings in Iranian patients with heartburn. Indian Journal of Gastroenterology 2005; 24 (6): 236-238.
122. Westhoff, B., Brotze, S., Weston, A., McElhinney, C., Cherian, R., Mayo, M. S., Smith, H. J., and Sharma, P. The frequency of Barrett's esophagus in high-risk patients with chronic GERD. Gastrointestinal endoscopy 2005; 61 (2): 226-231.
123. Postma, G. N., Cohen, J. T., Belafsky, P. C., Halum, S. L., Gupta, S. K., Bach, K. K., and Koufman, J. A. Transnasal esophagoscopy: Revisited (over 700 consecutive cases). Laryngoscope 2005; 115 (2): 321-323.
124. Takubo, K., Honma, N., Aryal, G., Sawabe, M., Arai, T., Tanaka, Y., Mafune, K.-I., and Iwakiri, K. Is there a set of histologic changes that are invariably reflux associated? Archives of Pathology and Laboratory Medicine 2005; 129 (2): 159-163.
125. Olvera, M., Wickramasinghe, K., Brynes, R., Bu, X., Ma, Y., and Chandrasoma, P. Ki67 expression in different epithelial types in columnar lined oesophagus indicates varying levels of expanded and aberrant proliferative patterns. Histopathology 2005; 47 (2): 132-140.
126. Shen, B., Porter, E. M., Reynoso, E., Shen, C., Ghosh, D., Connor, J. T., Drazba, J., Rho, H. K., Gramlich, T. L., Li, R., Ormsby, A. H., Sy, M.-S., Ganz, T., and Bevins, C. L. Human defensin 5 expression in intestinal metaplasia of the upper gastrointestinal tract. Journal of Clinical Pathology 2005; 58 (7): 687-694.
127. Gulmann, C., Rathore, O., Grace, A., Hegarty, H., O'Grady, A., Leader, M., Patchett, S., and Kay, E. 'Cardiac-type' (mucinous) mucosa and carditis are both associated with Helicobacter pylori-related gastritis. European Journal of Gastroenterology and Hepatology 2004; 16 (1): 69-74.
128. Toruner M, Soykan I, Ensari A, Kuzu I, Yurdaydin C, Ozden A. Barrett's esophagus: Prevalence and its relationship with dyspeptic symptoms. Journal of Gastroenterology and Hepatology (Australia) 2004; 19 (5): 535-540.
129. Balaji NS, DeMeester SR, Wickramasinghe KS, Hagen JA, Peters JH, DeMeester TR. Etiology of intestinal metaplasia at the gastroesophageal junction: Reflux, H. pylori infection, or both? Surgical Endoscopy and Other Interventional Techniques 2003; 17 (1): 43-48.
130. El-Serag, H. B., Petersen, N. J., Carter, J., Graham, D. Y., Richardson, P., Genta, R. M., and Rabeneck, L. Gastroesophageal reflux among different racial groups in the United States. Gastroenterology 2004; 126 (7): 1692-1699.
131. Zhang, J., Chen, X.-L., Wang, K.-M., Guo, X.-D., Zuo, A.-L., and Gong, J. Barrett's esophagus and its correlation with gastroesophageal reflux in Chinese. World journal of gastroenterology 2004; 10 (7): 1065-1068.
132. Rajendra, S., Kutty, K., and Karim, N. Ethnic differences in the prevalence of endoscopic esophagitis and Barrett's esophagus: The long and short of it all. Digestive Diseases and Sciences 2004; 49 (2): 237-242.
133. Wildi, S. M., Glenn, T. F., Woolson, R. F., Wang, W., Hawes, R. H., and Wallace, M. B. Is esophagoscopy alone sufficient for patients with reflux symptoms? Gastrointestinal endoscopy 2004; 59 (3): 349-354.
134. Loffeld, R. J. L. F. and van der Putten, A. B. M. M. Helicobacter pylori and gastro-oesophageal reflux disease: A cross-sectional epidemiological study. Netherlands Journal of Medicine 2004; 62 (6): 188-191.
135. Panter, S. J., O'Flanagan, H., Bramble, M. G., and Hungin, A. P. S. Empirical use of antisecretory drug therapy delays diagnosis of upper gastrointestinal adenocarcinoma but does not effect outcome. Alimentary Pharmacology and Therapeutics 2004; 19 (9): 981-988.
136. Lin, M., Gerson, L. B., Lascar, R., Davila, M., and Triadafilopoulos, G. Features of gastroesophageal reflux disease in women. American Journal of Gastroenterology 2004; 99 (8): 1442-1447.
137. Van, Blankenstein M., Bohmer, C. J. M., and Hop, W. C. J. The incidence of adenocarcinoma in Barrett's esophagus in an institutionalized population. European Journal of Gastroenterology and Hepatology 2004; 16 (9): 903-909.
138. Rosaida, M. S. and Goh, K.-L. Gastro-oesophageal reflux disease, reflux oesophagitis and non-erosive reflux disease in a multiracial Asian population: A prospective, endoscopy based study. European Journal of Gastroenterology and Hepatology 2004; 16 (5): 495-501.
139. Eda, A., Osawa, H., Satoh, K., Yanaka, I., Kihira, K., Ishino, Y., Mutoh, H., and Sugano, K. Aberrant expression of CDX2 in Barrett's epithelium and inflammatory esophageal mucosa. Journal of gastroenterology 2003; 38 (1): 14-22.
140. Lee, J. I., Park, H., Jung, H.-Y., Rhee, P.-L., Song, C. W., and Choi, M. G. Prevalence of Barrett's esophagus in an urban Korean population: A multicenter study. Journal of gastroenterology 2003; 38 (1): 23-27.
141. Dietz, J., Meurer, L., Maffazzoni, D. R., Furtado, A. D., and Prolla, J. C. Intestinal metaplasia in the distal esophagus and correlation with symptoms of gastroesphageal reflux disease. Diseases of the Esophagus 2003; 16 (1): 29-32.
142. Csendes, A., Smok, G., Burdiles, P., Korn, O., Gradiz, M., Rojas, J., and Recio, M. Prevalence of intestinal metaplasia according to the length of the specialized columnar epithelium lining the distal esophagus in patients with gastroesophageal reflux. Diseases of the Esophagus 2003; 16 (1): 24-28.
143. Khalaf, A. S. The efficacy of endoscopy: Analysis of its use in the evaluation of dyspeptic patients in Qatar. Emirates Medical Journal 2003; 21 (2): 134-138.
144. Fujiwara, Y., Higuchi, K., Shiba, M., Watanabe, T., Tominaga, K., Oshitani, N., Matsumoto, T., and Arakawa, T. Association between gastroesophageal flap valve, reflux esophagitis, Barrett's epithelium, and atrophic gastritis assessed by endoscopy in Japanese patients. Journal of gastroenterology 2003; 38 (6): 533-539.
145. Ackermark, P., Kuipers, E. J., Wolf, C., Breumelhof, R., Seldenrijk, C. A., Timmer, R., Segeren, K. C. A., Kusters, J. G., and Smout, A. J. P. M. Colonization with cagA-positive Helicobacter pylori strains in intestinal metaplasia of the esophagus and the esophagogastric junction. American Journal of Gastroenterology 2003; 98 (8): 1719-1724.
146. Fitzgerald, R. C., Onwuegbusi, B. A., Bajaj-Elliott, M., Saeed, I. T., Burnham, W. R., and Farthing, M. J. G. Diversity in the oesophageal phenotypic response to gastro-oesophageal reflux: Immunological determinants. Gut 2002; 50 (4): 451-459.
147. Mantynen, T., Farkkila, M., Kunnamo, I., Mecklin, J.-P., Juhola, M., and Voutilainen, M. The impact of upper GI endoscopy referral volume on the diagnosis of gastroesophageal reflux disease and its complications: A 1-year cross-sectional study in a referral area with 260,000 inhabitants. American Journal of Gastroenterology 2002; 97 (10): 2524-2529.
148. Jones, T. F., Sharma, P., Daaboul, B., Cherian, R., Mayo, M., Topalovski, M., and Weston, A. P. Yield of intestinal metaplasia in patients with suspected short-segment Barrett's esophagus (SSBE) on repeat endoscopy. Digestive Diseases and Sciences 2002; 47 (9): 2108-2111.
149. Loffeld, R. J. L. F. Young patients with Barrett's oesophagus experience less reflux complaints. Digestion 2001; 64 (3): 151-154.
150. Kongara, K., Varilek, G., and Soffer, E. E. Salivary growth factors and cytokines are not deficient in patients with gastroesophageal reflux disease or Barrett's esophagus. Digestive Diseases and Sciences 2001; 46 (3): 606-609.
151. Schowengerdt, C. G. Standard acid reflux testing revisited. Digestive Diseases and Sciences 2001; 46 (3): 603-605.
152. Gerson, L. B., Edson, R., Lavori, P. W., and Triadafilopoulos, G. Use of a simple symptom questionnaire to predict Barrett's esophagus in patients with symptoms of gastroesophageal reflux. American Journal of Gastroenterology 2001; 96 (7): 2005-2012.
153. Fireman, Z., Wagner, G., Weissman, J., Kopelman, Y., Wagner, Y., Groissman, G., and Sternberg, A. Prevalence of short-segment Barrett's epithelium. Digestive and Liver Disease 2001; 33 (4): 322-325.
154. Couvelard, A., Cauvin, J.-M., Goldfain, D., Rotenberg, A., Robaszkiewicz, M., Flejou, J.-F., Croue, A., Volant, A., Diebold, M.-D., Vissuzaine, C., Sagan, C., Boyer, J., Cadiot, G., Mignon, M., Aparicio, T., Galmiche, J.-P., and Rhun, M. L. Cytokeratin immunoreactivity of intestinal metaplasia at normal oesophagogastric junction indicates its aetiology. Gut 2001; 49 (6): 761-766.
155. Sabel, M. S., Pastore, K., Toon, H., and Smith, J. L. Adenocarcinoma of the esophagus with and without Barrett mucosa. Archives of Surgery 2000; 135 (7): 831-835.
156. Voutilainen, M., Farkkila, M., Mecklin, J.-P., Juhola, M., and Sipponen, P. Classical Barrett esophagus contrasted with Barrett-type epithelium at normal-appearing esophagogastric junction: Comparison of demographic, endoscopic, and histologic features. Scandinavian Journal of Gastroenterology 2000; 35 (1): 2-9.
157. Wallner, B., Sylvan, A., Stenling, R., and Janunger, K.-G. The esophageal Z-line appearance correlates to the prevalence of intestinal metaplasia. Scandinavian Journal of Gastroenterology 2000; 35 (1): 17-22.
158. Tack, J. and Sifrim, D. New techniques for the detection of gastro-oesophageal reflux. Digestive and Liver Disease 2000; 32 (SUPPL. 3): S245-S248.
159. Shah, N. H., Shah, M. S., Khan, I., and Hameed, K. An audit of diagnostic upper GI endoscopy and comparison of booked versus open access cases. Journal of the College of Physicians and Surgeons Pakistan 1999; 9 (4): 174-176.
160. Parrilla, P., Liron, R., Martinez de Haro, L. F., Ortiz, A., Molina, J., and De, Andres B. Gastric surgery does not increase the risk of developing Barrett's esophagus. American Journal of Gastroenterology 1997; 92 (6): 960-963.
161. Triadafilopoulos, G. and Sharma, R. Features of symptomatic gastroesophageal reflux disease in elderly patients. American Journal of Gastroenterology 1997; 92 (11): 2007-2011.
162. Triadafilopoulos, G., Kaczynska, M., and Iwane, M. Esophageal mucosal eicosanoids in gastroesophageal reflux disease and Barrett's esophagus. American Journal of Gastroenterology 1996; 91 (1): 65-74.
163. Katsinelos, P., Lazaraki, G., Kountouras, J., Chatzimavroudis, G., Zavos, C., Terzoudis, S., Tsiaousi, E., Gkagkalis, S., Trakatelli, C., Bellou, A., and Vasiliadis, T. Prevalence of Barrett's esophagus in Northern Greece: A Prospective Study (Barrett's esophagus). Hippokratia 2013; 17 (1): 27-33.
164. Livovsky, Dan M., Pappo, Orit, Skarzhinsky, Galina, Peretz, Asaf, Turvall, Elliot, and Ackerman, Zvi Gastric Polyp Growth during Endoscopic Surveillance for Esophageal Varices or Barrett's Esophagus. The Israel Medical Association journal 2016; 18 (5): 267-271.
165. Mohiuddin, Mohammed Khaliq, Chowdavaram, Suman, Bogadi, Varun, Prabhakar, Boddu, Rao, Kondadasula Pandu Ranga, Devi, Suneetha, and Mohan, Vasavi Epidemic Trends of Upper Gastrointestinal Tract Abnormalities: Hospital-based study on Endoscopic Data Evaluation. Asian Pacific journal of cancer prevention 2015; 16 (14): 5741-5747.
166. Lee, Hyun Jik, Kim, Bun, Kim, Dong Wook, Park, Jun Chul, Shin, Sung Kwan, Lee, Yong Chan, and Lee, Sang Kil Does Sedation Affect Examination of Esophagogastric Junction during Upper Endoscopy? Yonsei medical journal 2015; 56 (6): 1566-1571.
167. Iijima, Katsunori, Koike, Tomoyuki, Abe, Yasuhiko, Ohara, Shuichi, Nakaya, Naoki, and Shimosegawa, Tooru Time series analysis of gastric acid secretion over a 20-year period in normal Japanese men. Journal of gastroenterology 2015; 50 (8): 853-861.
168. Arif, Tasleem, Masood, Qazi, Singh, Jaswinder, and Hassan, Iffat Assessment of esophageal involvement in systemic sclerosis and morphea (localized scleroderma) by clinical, endoscopic, manometric and pH metric features: a prospective comparative hospital based study. BMC gastroenterology 2015; 15: 24-.
169. Gibbs, John F., Rajput, Ashwani, Chadha, Krishdeep S., Douglas, Wade G., Hill, Hank, Nwogu, Chukwumere, Nava, Hector R., and Sabel, Michael S. The changing profile of esophageal cancer presentation and its implication for diagnosis. Journal of the National Medical Association 2007; 99 (6): 620-626.
170. Watari, Jiro, Hori, Kazutoshi, Toyoshima, Fumihiko, Kamiya, Noriko, Yamasaki, Takahisa, Okugawa, Takuya, Asano, Haruki, Li, Zhao Liang, Kondo, Takashi, Ikehara, Hisatomo, Sakurai, Jun, Tomita, Toshihiko, Oshima, Tadayuki, Fukui, Hirokazu, and Miwa, Hiroto Association between obesity and Barrett's esophagus in a Japanese population: a hospital-based, cross-sectional study. BMC gastroenterology 2013; 13: 143-.
171. Nason, Katie S., Murphy, Thomas, Schindler, Joshua, Schipper, Paul H., Hoppo, Toshitaka, Diggs, Brian S., Sauer, David A., Shaheen, Nicholas J., Morris, Cynthia D., Jobe, Blair A., and Barrett's Esophagus Risk Consortium (BERC) A cross-sectional analysis of the prevalence of Barrett esophagus in otolaryngology patients with laryngeal symptoms. Journal of Clinical Gastroenterology 2013; 47 (9): 762-768.
172. Savarino, Edoardo, Corbo, Marina, Dulbecco, Pietro, Gemignani, Lorenzo, Giambruno, Elisa, Mastracci, Luca, Grillo, Federica, and Savarino, Vincenzo Narrow-band imaging with magnifying endoscopy is accurate for detecting gastric intestinal metaplasia. World journal of gastroenterology 2013; 19 (17): 2668-2675.
173. Niu, Chun Yan, Zhou, Yong Li, Yan, Rong, Mu, Ni La, Gao, Bao Hua, Wu, Fang Xiong, and Luo, Jin Yan Incidence of gastroesophageal reflux disease in Uygur and Han Chinese adults in Urumqi. World journal of gastroenterology 2012; 18 (48): 7333-7340.
174. Cardin, Fabrizio, Andreotti, Alessandra, Zorzi, Manuel, Terranova, Claudio, Martella, Bruno, Amato, Bruno, and Militello, Carmelo Usefulness of a fast track list for anxious patients in a upper GI endoscopy. BMC surgery 2012; 12 Suppl 1: S11-.
175. Gerson, L. B., McLaughlin, T., Balu, S., Jackson, J., and Lunacsek, O. Variation of health-care resource utilization according to GERD-associated complications. Diseases of the esophagus 2012; 25 (8): 694-701.
176. Kidambi, Trilokesh, Toto, Erin, Ho, Nancy, Taft, Tiffany, and Hirano, Ikuo Temporal trends in the relative prevalence of dysphagia etiologies from 1999-2009. World journal of gastroenterology 2012; 18 (32): 4335-4341.
177. Erichsen, Rune, Robertson, Douglas, Farkas, Dora K., Pedersen, Lars, Pohl, Heiko, Baron, John A., and Sorensen, Henrik T. Erosive reflux disease increases risk for esophageal adenocarcinoma, compared with nonerosive reflux. Clinical gastroenterology and hepatology 2012; 10 (5): 475-480.
178. Khoury, Joe E., Chisholm, Sian, Jamal, M. Mazen, Palacio, Carlos, Pudhota, Sunitha, and Vega, Kenneth J. African Americans with Barrett's esophagus are less likely to have dysplasia at biopsy. Digestive Diseases and Sciences 2012; 57 (2): 419-423.
179. Fletcher, Kenneth C., Goutte, Marion, Slaughter, James C., Garrett, C. Gaelyn, and Vaezi, Michael F. Significance and degree of reflux in patients with primary extraesophageal symptoms. The Laryngoscope 2011; 121 (12): 2561-2565.
180. Irvanloo, Giti, Fallahi, Behnaz, Ensani, Fereshte, Azmi, Mehrdad, and Morteza, Afsaneh Endoscopic versus histological diagnosis of Barrett's esophagus: a cross-sectional survey. Polish journal of pathology 2011; 62 (3): 152-156.
181. Juhasz, Arpad, Sundaram, Abhishek, Hoshino, Masato, Lee, Tommy H., Filipi, Charles J., and Mittal, Sumeet K. Endoscopic assessment of failed fundoplication: a case for standardization. Surgical endoscopy 2011; 25 (12): 3761-3766.
182. Roorda, Andrew K., Marcus, Samuel N., and Triadafilopoulos, George Algorithmic approach to patients presenting with heartburn and epigastric pain refractory to empiric proton pump inhibitor therapy. Digestive Diseases and Sciences 2011; 56 (10): 2871-2878.
183. Gaddam, Srinivas, Maddur, Haripriya, Wani, Sachin, Gupta, Neil, Singh, Mandeep, Singh, Vikas, Moloney, Brian, Puli, Srinivas R., Rastogi, Amit, Bansal, Ajay, and Sharma, Prateek Risk factors for nocturnal reflux in a large GERD cohort. Journal of Clinical Gastroenterology 2011; 45 (9): 764-768.
184. Nason, Katie S., Wichienkuer, Promporn Paula, Awais, Omar, Schuchert, Matthew J., Luketich, James D., O'Rourke, Robert W., Hunter, John G., Morris, Cynthia D., and Jobe, Blair A. Gastroesophageal reflux disease symptom severity, proton pump inhibitor use, and esophageal carcinogenesis. Archives of surgery (Chicago, Ill.: 1960) 2011; 146 (7): 851-858.
185. Masci, Enzo, Viaggi, Paolo, Mangiavillano, Benedetto, Di Pietro, Salvatore, Micheletto, Giancarlo, Di Prisco, Franco, Paganelli, Michele, Pontiroli, Antonio Ettore, Pontiroli, Ettore Antonio, Laneri, Marco, Testoni, Sabrina, and Testoni, Pier Alberto No increase in prevalence of Barrett's oesophagus in a surgical series of obese patients referred for laparoscopic gastric banding. Digestive and liver disease 2011; 43 (8): 613-615.
186. Chandrasoma, Parakrama, Wijetunge, Sulochana, Demeester, Steven R., Hagen, Jeffrey, and Demeester, Tom R. The histologic squamo-oxyntic gap: an accurate and reproducible diagnostic marker of gastroesophageal reflux disease. The American journal of surgical pathology 2010; 34 (11): 1574-1581.
187. Chen, M. J., Lee, Y. C., Chiu, H. M., Wu, M. S., Wang, H. P., and Lin, J. T. Time trends of endoscopic and pathological diagnoses related to gastroesophageal reflux disease in a Chinese population: eight years single institution experience. Diseases of the esophagus 2010; 23 (3): 201-207.
188. Xiong, Li Shou, Cui, Yi, Wang, Jin Ping, Wang, Jin Hui, Xue, Ling, Hu, Pin Jin, and Chen, Min Hu Prevalence and risk factors of Barrett's esophagus in patients undergoing endoscopy for upper gastrointestinal symptoms. Journal of Digestive Diseases 2010; 11 (2): 83-87.
189. Beech, T. J., Trotter, M. I., McDermott, A. L., Mandal, W., and Batch, A. J. Is there a role for flexible oesophagogastroscopy in upper aerodigestive tract squamous cell carcinoma? The Journal of laryngology and otology 2010; 124 (4): 417-419.
190. Zagari, Rocco Maurizio, Law, Graham Richard, Fuccio, Lorenzo, Pozzato, Paolo, Forman, David, and Bazzoli, Franco Dyspeptic symptoms and endoscopic findings in the community: the Loiano-Monghidoro study. The American journal of gastroenterology 2010; 105 (3): 565-571.
191. Odemis, B., Cicek, B., Zengin, N. I., Arhan, M., Kacar, S., Cengiz, C., and Yuksel, O. Barrett's esophagus and endoscopically assessed esophagogastric junction integrity in 1000 consecutive Turkish patients undergoing endoscopy: a prospective study. Diseases of the esophagus 2009; 22 (8): 649-655.
192. Binato, M., Gurski, R. R., Fagundes, R. B., Meurer, L., and Edelweiss, M. I. P53 and Ki-67 overexpression in gastroesophageal reflux disease--Barrett's esophagus and adenocarcinoma sequence. Diseases of the esophagus 2009; 22 (7): 588-595.
193. Vakil, Nimish, Talley, Nicholas, van Zanten, Sander Veldhuyzen, Flook, Nigel, Persson, Tore, Bjorck, Ewa, Lind, Tore, Bolling-Sternevald, Elisabeth, and STARS, I. Study Group Cost of detecting malignant lesions by endoscopy in 2741 primary care dyspeptic patients without alarm symptoms. Clinical gastroenterology and hepatology 2009; 7 (7): 756-761.
194. Smith, Jeanette G., Li, Wenjie, and Rosson, Robert S. Prevalence, clinical and endoscopic predictors of Helicobacter pylori infection in an urban population. Connecticut medicine 2009; 73 (3): 133-137.
195. Quispel, R., van Boxel, O. S., Schipper, M. E., Sigurdsson, V., Canninga-van Dijk, M. R., Kerckhoffs, A., Smout, A. J., Samsom, M., and Schwartz, M. P. High prevalence of esophageal involvement in lichen planus: a study using magnification chromoendoscopy. Endoscopy 2009; 41 (3): 187-193.
196. Perry, Kyle A., Enestvedt, C. Kristian, Lorenzo, Cedric S. F., Schipper, Paul, Schindler, Joshua, Morris, Cynthia D., Nason, Katie, Luketich, James D., Hunter, John G., and Jobe, Blair A. The integrity of esophagogastric junction anatomy in patients with isolated laryngopharyngeal reflux symptoms. Journal of gastrointestinal surgery 2008; 12 (11): 1880-1887.
197. Lee, Jun Haeng, Kim, Nayoung, Chung, Il Kwun, Jo, Yun Ju, Seo, Geom Seog, Kim, Sang Wook, Im, Eui Hyeog, Kim, Hye Rang, Park, Soo Hyun, Lee, So Young, Cha, Hyun Min, Lee, Kyoung Soo, Hyun, Dong Hyo, Kim, Hyun Young, Kim, Sun Mi, Shin, Jeong Eun, Park, Soo Heon, Chung, Hyun Chae, Chung, In Sik, and .pylori and GERD Study Group of Korean College of Helicobacter and Upper Gastrointestinal Research Clinical significance of minimal change lesions of the esophagus in a healthy Korean population: a nationwide multi-center prospective study. Journal of gastroenterology and hepatology 2008; 23 (7 Pt 1): 1153-1157.
198. Martinek, Jan, Benes, Marek, Hucl, Tomas, Drastich, Pavel, Stirand, Petr, and Spicak, Julius Non-erosive and erosive gastroesophageal reflux diseases: No difference with regard to reflux pattern and motility abnormalities. Scandinavian Journal of Gastroenterology 2008; 43 (7): 794-800.
199. Ramirez, Francisco C., Akins, Rodney, and Shaukat, Masud Screening of Barrett's esophagus with string-capsule endoscopy: a prospective blinded study of 100 consecutive patients using histology as the criterion standard. Gastrointestinal endoscopy 2008; 68 (1): 25-31.
200. Abrams, Julian A., Fields, Sydney, Lightdale, Charles J., and Neugut, Alfred I. Racial and ethnic disparities in the prevalence of Barrett's esophagus among patients who undergo upper endoscopy. Clinical gastroenterology and hepatology 2008; 6 (1): 30-34.
201. Francois, F., Roper, J., Goodman, A. J., Pei, Z., Ghumman, M., Mourad, M., de Perez, A. Z. O., Perez-Perez, G. I., Tseng, C. H., and Blaser, M. J. The association of gastric leptin with oesophageal inflammation and metaplasia. Gut 2008; 57 (1): 16-24.
202. Diarra, M., Konate, A., Traore, C. B., Drabo, M., Soukho, A. espouse Diarra, Kalle, A., Dembele, M., Traore, H. A., and Maiga, M. Y. [Gastritis associated with duodeno-gastric reflux]. Le Mali medical 2007; 22 (4): 47-51.
203. Reichel, O. and Issing, W. J. Should patients with pH-documented laryngopharyngeal reflux routinely undergo oesophagogastroduodenoscopy? A retrospective analysis. The Journal of laryngology and otology 2007; 121 (12): 1165-1169.
204. Kim, Jeong Hwan, Rhee, Poong Lyul, Lee, Jun Haeng, Lee, Hyuk, Choi, Yong Sung, Son, Hee Jung, Kim, Jae J., and Rhee, Jong Chul Prevalence and risk factors of Barrett's esophagus in Korea. Journal of gastroenterology and hepatology 2007; 22 (6): 908-912.
205. Evans, John A., Bouma, Brett E., Bressner, Jason, Shishkov, Milen, Lauwers, Gregory Y., Mino-Kenudson, Mari, Nishioka, Norman S., and Tearney, Guillermo J. Identifying intestinal metaplasia at the squamocolumnar junction by using optical coherence tomography. Gastrointestinal endoscopy 2007; 65 (1): 50-56.
206. Punia, R. S., Arya, Savita, Mohan, H., Duseja, A., and Bal, A. Spectrum of clinico-pathological changes in Barrett oesophagus. The Journal of the Association of Physicians of India 2006; 54 (): 187-189.
207. Lassen, Annmarie, Hallas, Jesper, and de Muckadell, Ove B. S. Esophagitis: incidence and risk of esophageal adenocarcinoma--a population-based cohort study. The American journal of gastroenterology 2006; 101 (6): 1193-1199.
208. Veldhuyzen van Zanten, S. J. O., Thomson, A. B. R., Barkun, A. N., Armstrong, D., Chiba, N., White, R. J., Escobedo, S., and Sinclair, P. The prevalence of Barrett's oesophagus in a cohort of 1040 Canadian primary care patients with uninvestigated dyspepsia undergoing prompt endoscopy. Alimentary pharmacology & therapeutics 2006; 23 (5): 595-599.
209. Alonso, Oscar, Hernandez, Diego, Moreno, Enrique, Manrique, Alejandro, Moreno, Almudena, Garcia-Sesma, Alvaro, and Calvo, Jorge The real value of lower esophageal sphincter measurement for predicting acid gastroesophageal reflux or Barrett's esophagus. Journal of gastrointestinal surgery 2005; 9 (7): 973-979.
210. Rouev, Petar, Chakarski, Ivan, Doskov, Dosjo, Dimov, Gospodin, and Staykova, Elena Laryngopharyngeal symptoms and gastroesophageal reflux disease. Journal of voice 2005; 19 (3): 476-480.
211. Andrus, Jennifer G., Dolan, Robert W., and Anderson, Timothy D. Transnasal esophagoscopy: a high-yield diagnostic tool. The Laryngoscope 2005; 115 (6): 993-996.
212. Wilkins, Thad and Gillies, Ralph A. Office-based unsedated ultrathin esophagoscopy in a primary care setting. Annals of family medicine 2005; 3 (2): 126-130.
213. Kanthan, R., Torkian, B., and Kanthan, S. C. Pathological validity of esophageal endoscopy. How real is what we see? Myth or reality?. Diseases of the esophagus 2004; 17 (4): 304-306.
214. Brandt, Michael G., Darling, Gail E., and Miller, Linda Symptoms, acid exposure and motility in patients with Barrett's esophagus. Canadian journal of surgery. Journal canadien de chirurgie 2004; 47 (1): 47-51.
215. Loffeld, R. J. L. F. and van der Putten, A. B. M. M. Rising incidence of reflux oesophagitis in patients undergoing upper gastrointestinal endoscopy. Digestion 2003; 68 (2-3): 141-144.
216. Mokhashi, Mahesh S., Wildi, Stephan M., Glenn, Tammy F., Wallace, Michael B., Jost, Christian, Gumustop, Bora, Kim, Christopher Y., Cotton, Peter B., and Hawes, Robert H. A prospective, blinded study of diagnostic esophagoscopy with a superthin, stand-alone, battery-powered esophagoscope. The American journal of gastroenterology 2003; 98 (11): 2383-2389.
217. Koufman, James A., Belafsky, Peter C., Bach, Kevin K., Daniel, Elena, and Postma, Gregory N. Prevalence of esophagitis in patients with pH-documented laryngopharyngeal reflux. The Laryngoscope 2002; 112 (9): 1606-1609.
218. El-Serag, Hashem B., Bailey, Nadine R., Gilger, Mark, and Rabeneck, Linda Endoscopic manifestations of gastroesophageal reflux disease in patients between 18 months and 25 years without neurological deficits. The American journal of gastroenterology 2002; 97 (7): 1635-1639.
219. Laheij, R. J. F., van Rossum, L. G. M., De Boer, W. A., and Jansen, J. B. M. J. Corpus gastritis in patients with endoscopic diagnosis of reflux oesophagitis and Barrett's oesophagus. Alimentary pharmacology & therapeutics 2002; 16 (5): 887-891.
220. Eloubeidi, M. A. and Provenzale, D. Clinical and demographic predictors of Barrett's esophagus among patients with gastroesophageal reflux disease: a multivariable analysis in veterans. Journal of Clinical Gastroenterology 2001; 33 (4): 306-309.
221. Dhawan, P. S., Alvares, J. F., Vora, I. M., Joseph, T. K., Bhatia, S. J., Amarapurkar, A. D., Parikh, S. S., Kulkarni, S. G., and Kalro, R. H. Prevalence of short segments of specialized columnar epithelium in distal esophagus: association with gastroesophageal reflux. Indian journal of gastroenterology 2001; 20 (4): 144-147.
222. Lanzafame, S., Torrisi, A., Favara, C., Russo, V., and Emmanuele, C. Correlation between intestinal metaplasia of the gastric cardia and gastroesophageal reflux disease. Hepato-gastroenterology 2001; 48 (40): 1007-1010.
223. Fass, R., Hell, R. W., Garewal, H. S., Martinez, P., Pulliam, G., Wendel, C., and Sampliner, R. E. Correlation of oesophageal acid exposure with Barrett's oesophagus length. Gut 2001; 48 (3): 310-313.
224. Vieth, M. and Stolte, M. Barrett's mucosa, Barrett's dysplasia and Barrett's carcinoma: diagnostic endoscopy without biopsy-taking does not suffice. Diseases of the esophagus 2000; 13 (1): 23-27.
225. Voutilainen, M., Sipponen, P., Mecklin, J. P., Juhola, M., and Farkkila, M. Gastroesophageal reflux disease: prevalence, clinical, endoscopic and histopathological findings in 1,128 consecutive patients referred for endoscopy due to dyspeptic and reflux symptoms. Digestion 2000; 61 (1): 6-13.
226. Carton, E., Caldwell, M. T., McDonald, G., Rama, D., Tanner, W. A., and Reynolds, J. V. Specialized intestinal metaplasia in patients with gastro-oesophageal reflux disease. The British journal of surgery 2000; 87 (1): 116-121.
227. Oberg, S., Peters, J. H., Nigro, J. J., Theisen, J., Hagen, J. A., DeMeester, S. R., Bremner, C. G., and DeMeester, T. R. Helicobacter pylori is not associated with the manifestations of gastroesophageal reflux disease. Archives of surgery (Chicago, Ill.: 1960) 1999; 134 (7): 722-726.
228. El-Serag, H. B., Sonnenberg, A., Jamal, M. M., Kunkel, D., Crooks, L., and Feddersen, R. M. Characteristics of intestinal metaplasia in the gastric cardia. The American journal of gastroenterology 1999; 94 (3): 622-627.
229. Gadour, M. O. and Ayoola, E. A. Barrett's oesophagus and oesophageal cancer in Saudi Arabia. Tropical gastroenterology 1999; 20 (3): 111-115.
230. Aste, H., Bonelli, L., Ferraris, R., Conio, M., and Lapertosa, G. Gastroesophageal reflux disease: relationship between clinical and histological features. GOSPE. Gruppo Operativo per lo Studio delle Precancerosi dell'Esofago. Digestive Diseases and Sciences 1999; 44 (12): 2412-2418.
231. Loughney, T., Maydonovitch, C. L., and Wong, R. K. Esophageal manometry and ambulatory 24-hour pH monitoring in patients with short and long segment Barrett's esophagus. The American journal of gastroenterology 1998; 93 (6): 916-919.
232. Ellis, K. K., Oehlke, M., Helfand, M., and Lieberman, D. Management of symptoms of gastroesophageal reflux disease: does endoscopy influence medical management?. The American journal of gastroenterology 1997; 92 (9): 1472-1474.
233. Lieberman, D. A., Oehlke, M., and Helfand, M. Risk factors for Barrett's esophagus in community-based practice. GORGE consortium. Gastroenterology Outcomes Research Group in Endoscopy. The American journal of gastroenterology 1997; 92 (8): 1293-1297.
234. Weston, A. P., Krmpotich, P. T., Cherian, R., Dixon, A., and Topalovski, M. Prospective evaluation of intestinal metaplasia and dysplasia within the cardia of patients with Barrett's esophagus. Digestive Diseases and Sciences 1997; 42 (3): 597-602.
235. Morales, T. G., Sampliner, R. E., and Bhattacharyya, A. Intestinal metaplasia of the gastric cardia. The American journal of gastroenterology 1997; 92 (3): 414-418.
236. Johnston, M. H., Hammond, A. S., Laskin, W., and Jones, D. M. The prevalence and clinical characteristics of short segments of specialized intestinal metaplasia in the distal esophagus on routine endoscopy. The American journal of gastroenterology 1996; 91 (8): 1507-1511.
237. Weston, A. P., Krmpotich, P., Makdisi, W. F., Cherian, R., Dixon, A., McGregor, D. H., and Banerjee, S. K. Short segment Barrett's esophagus: clinical and histological features, associated endoscopic findings, and association with gastric intestinal metaplasia. The American journal of gastroenterology 1996; 91 (5): 981-986.
238. Collen, M. J., Abdulian, J. D., and Chen, Y. K. Gastroesophageal reflux disease in the elderly: more severe disease that requires aggressive therapy. The American journal of gastroenterology 1995; 90 (7): 1053-1057.
239. Borhan-Manesh, F. and Farnum, J. B. Incidence of heterotopic gastric mucosa in the upper oesophagus. Gut 1991; 32 (9): 968-972.
240. Chobanian, S. J., Cattau, E. L. J., Winters, C. Jr, Johnson, D. A., Van Ness, M. M., Miremadi, A., Horwitz, S. L., and Colcher, H. In vivo staining with toluidine blue as an adjunct to the endoscopic detection of Barrett's esophagus. Gastrointestinal endoscopy 1987; 33 (2): 99-101.
241. Savarino, E., Tolone, S., Caccaro, R., Bartolo, O., Galeazzi, F., Nicoletti, L., Morbin, T., Zanatta, L., Salvador, R., and Costantini, M. Clinical, endoscopic, histological and radiological characteristics of Italian patients with eosinophilic oesophagitis. Digestive and Liver Disease 2015; 47 (12): 1033-1038.
242. Carabotti, M., Avallone, M., Cereatti, F., Paganini, A., Greco, F., Scirocco, A., Severi, C., and Silecchia, G. Usefulness of Upper Gastrointestinal Symptoms as a Driver to Prescribe Gastroscopy in Obese Patients Candidate to Bariatric Surgery. A Prospective Study. Obesity Surgery 2016; 26 (5): 1075-1080.
243. Ayana, S. M., Swai, B., Maro, V. P., and Kibiki, G. S. Upper gastrointestinal endoscopic findings and prevalence of helicobacter pylori infection among adult patients with dyspepsia in northern Tanzania. Tanzania Journal of Health Research 2014; 16 (1): no-.
244. Cao, H.-L., Song, S.-L., Yang, B.-L., Zhang, Z.-H., Zhang, H., Qu, R., and Wang, B.-M. Typical endoscopic appearance accurately predicts sporadic fundic gland polyps: A retrospective study of 47 771 endoscopies. Chinese Medical Journal 2013; 126 (15): 2984-2986.
245. Jeje, E., Olajide, T., and Akande, B. Upper gastrointestinal endoscopy - Our findings, our experience in Lagoon hospital, Lagos, Nigeria. Macedonian Journal of Medical Sciences 2013; 6 (2).
246. Lim, S. W., Lee, J. H., Kim, J.-H., Kim, J. H., Kim, H. U., and Jeon, S. W. Management of asymptomatic erosive esophagitis: An E-mail survey of physician's opinions. Gut and liver 2013; 7 (3): 290-294.
247. Kao, S.-S., Chen, W.-C., Hsu, P.-I., Chuah, S.-K., Lu, C.-L., Lai, K.-H., Tsai, F.-W., Chang, C.-C., and Tai, W.-C. The frequencies of gastroesophageal and extragastroesophageal symptoms in patients with mild erosive esophagitis, severe erosive esophagitis, and barrett's esophagus in Taiwan. Gastroenterology Research and Practice 2013.
248. Khan, O. U. and Rasheed, A. Frequency of Barrett esophagus in patients with symptoms of gastroesophageal reflux disease. Rawal Medical Journal 2008; 33 (2): 205-207.
249. Khalid, K., Al-Salamah, S. M., Al-Teimi, I., and Al-Dossary, N. F. Gastrointestinal tract bleeding in intellectually disabled adults. Southern Medical Journal 2008; 101 (1): 29-34.
250. Takwoingi, Y. M., Kale U.S., and Morgan, D. W. Rigid endoscopy in globus pharyngeus: How valuable is it? Journal of Laryngology and Otology 2006; 120 (1): 42-46.
251. Banki, F., DeMeester, S. R., Mason, R. J., Campos, G., Hagen, J. A., Peters, J. H., Bremner, C. G., and DeMeester, T. R. Barrett's esophagus in females: A comparative analysis of risk factors in females and males. American Journal of Gastroenterology 2005; 100 (3): 560-567.
252. Kiltz, U., Pfaffenbach, B., Schmidt, W. E., and Adamek, R. J. The lack of influence of CagA positive Helicobacter pylori strains on gastro-oesophageal reflux disease. European Journal of Gastroenterology and Hepatology 2002; 14 (9): 979-984.
253. Wallner, B., Sylvan, A., Stenling, R., and Janunger, K.-G. A postfundoplication study on Z-line appearance and intestinal metaplasia in the gastroesophageal junction. Surgical Laparoscopy, Endoscopy and Percutaneous Techniques 2001; 11 (4): 235-241.
254. Campos, G. M. R., DeMeester, S. R., Peters, J. H., Oberg, S., Crookes, P. F., Hagen, J. A., Bremner, C. G., Sillin III, L. F., Mason, R. J., and DeMeester, T. R. Predictive factors of Barrett esophagus: Multivariate analysis of 502 patients with gastroesophageal reflux disease. Archives of Surgery 2001; 136 (11): 1267-1273.
255. Peck-Radosavljevic, M., Puspok, A., Potzi, R., and Oberhuber, G. Histological findings after routine biopsy at the gastro-oesophagealjunction. European Journal of Gastroenterology and Hepatology 1999; 11 (11): 1265-1270.
256. Conio, M., Bonelli, L., Munizzi, F., Carbone, A., Volpe, R., and Aste, H. Prevalence of Barrett's esophagus in patients with gastroesophageal reflux disease. Acta Endoscopica 1988; 18 (5): 339-346.
257. Bohmer, C. J., Klinkenberg-Knol, E. C., Niezen-de Boer, R. C., and Meuwissen, S. G. The age-related incidences of oesophageal carcinoma in intellectually disabled individuals in institutes in The Netherlands. European journal of gastroenterology & hepatology 1997; 9 (6): 589-592.
258. Wang, A., Mattek, N. C., Holub, J. L., Lieberman, D. A., and Eisen, G. M. Prevalence of complicated gastroesophageal reflux disease and Barrett's esophagus among racial groups in a multi-center consortium. Digestive diseases and sciences 2009; 54 (5): 964-971.
259. Gerson, L. B., Ullah, N., Fass, R., Green, C., Shetler, K., and Singh, G. Does body mass index differ between patients with Barrett's oesophagus and patients with chronic gastro-oesophageal reflux disease? Alimentary Pharmacology and Therapeutics 2007; 25 (9): 1079-1086.
260. Savarino, V., Mela, G. S., Zentilin, P., Mele, M. R., Mansi, C., Remagnino, A. C., Vigneri, S., Malesci, A., Belicchi, M., Lapertosa, G., and Celle, G. Time pattern of gastric acidity in Barrett's esophagus. Digestive diseases and sciences 1996; 41 (7): 1379-1383.
261. Nishimura, K., Tanaka, T., Tsubuku, T., Matono, S., Nagano, T., Murata, K., Aoyama, Y., Yanagawa, T., Shirouzu, K., and Fujita, H. Reflux esophagitis after esophagectomy: impact of duodenogastroesophageal reflux. Diseases of the esophagus 2012; 25 (5): 381-385.
262. Lee, In Seok, Choi, Suck Chei, Shim, Ki Nam, Jee, Sam Ryong, Huh, Kyu Chan, Lee, Jun Haeng, Lee, Kwang Jae, Park, Hyung Seok, Lee, Yong Chan, Jung, Hoon Yong, and Park, Hyo Jin Prevalence of Barrett's esophagus remains low in the Korean population: nationwide cross-sectional prospective multicenter study. Digestive diseases and sciences 2010; 55 (7): 1932-1939.
263. Wickramasinghe, Kumari S., Chandrasoma, Parakrama T., and Chandraratna, P. Anthony Detection of Barrett's epithelium by acoustic microscopy. Ultrasound in medicine & biology 2002; 28 (2): 203-207.
264. Eloubeidi, M. A. and Provenzale, D. Health-related quality of life and severity of symptoms in patients with Barrett's esophagus and gastroesophageal reflux disease patients without Barrett's esophagus. The American journal of gastroenterology 2000; 95 (8): 1881-1887.
265. O'Connor, H. J. and Cunnane, K. Helicobacter pylori and gastro-oesophageal reflux disease--a prospective study. Irish journal of medical science 1994; 163 (8): 369-373.
266. Katzka, D. A., Reynolds, J. C., Saul, S. H., Plotkin, A., Lang, C. A., Ouyang, A., Jimenez, S., and Cohen, S. Barrett's metaplasia and adenocarcinoma of the esophagus in scleroderma. The American journal of medicine 1987; 82 (1): 46-52.
267. Roberts, I. M., Curtis, R. L., and Madara, J. L. Gastroesophageal reflux and Barrett's esophagus in developmentally disabled patients. The American journal of gastroenterology 1986; 81 (7): 519-523.
268. Messian, R. A., Hermos, J. A., Robbins, A. H., Friedlander, D. M., and Schimmel, E. M. Barrett's esophagus. Clinical review of 26 cases. The American journal of gastroenterology 1978; 69 (4): 458-466.
269. Barros, R., Pereira, D., Calle, C., Camilo, V., Cunha, A. I., David, L., Almeida, R., Dias-Pereira, A., and Chaves, P. Dynamics of SOX2 and CDX2 Expression in Barrett's Mucosa. Disease Markers 2016.
270. Ho, K.-Y., Tay, H.-H., and Kang, J.-Y. A prospective study of the clinical features, manometric findings, incidence and prevalence of achalasia in Singapore. Journal of Gastroenterology and Hepatology (Australia) 1999; 14 (8): 791-795.
271. Zaidi, A. H., Gopalakrishnan, V., Kasi, P. M., Zeng, X., Malhotra, U., Balasubramanian, J., Visweswaran, S., Sun, M., Flint, M. S., Davison, J. M., Hood, B. L., Conrads, T. P., Bergman, J. J., Bigbee, W. L., and Jobe, B. A. Evaluation of a 4-protein serum biomarker panel-biglycan, annexin-A6, myeloperoxidase, and protein S100-A9 (B-AMP)-for the detection of esophageal adenocarcinoma. Cancer 15-12-2014; 120 (24): 3902-3913.
272. Langner, C., Schneider, N. I., Plieschnegger, W., Schmack, B., Bordel, H., Hofler, B., Eherer, A. J., Wolf, E. M., Rehak, P., and Vieth, M. Cardiac mucosa at the gastro-oesophageal junction: indicator of gastro-oesophageal reflux disease? Data from a prospective central European multicentre study on histological and endoscopic diagnosis of oesophagitis (histoGERD trial). Histopathology 2014; 65 (1): 81-89.
273. Wang, W., Uedo, N., Yang, Y., Peng, L., Bai, D., Lu, Z., Fan, K., Wang, J., Wang, X., Zhao, Y., and Yu, Z. Autofluorescence imaging endoscopy for predicting acid reflux in patients with gastroesophageal reflux disease. Journal of gastroenterology and hepatology 2014; 29 (7): 1442-1448.
274. The NHS Information Centre. An audit of the care received by people with Oesophago-Gastric Cancer in England and Wales Second Annual Report. 2009, Document reference: IC23090209. Retrieved from <https://digital.nhs.uk/catalogue/PUB02752>
275. A Diagnostic Study of the Advanced Endoscopy to Detect Early Esophageal Cancer. Last updated: July 7, 2014 ClinicalTrials.gov Identifier: NCT02182804
276. Prueksapanich P, Pittayanon R, Rerknimitr R, Wisedopas N, Kullavanijaya P. Value of probe-based confocal laser endomicroscopy (pCLE) and dual focus narrow-band imaging (dNBI) in diagnosing early squamous cell neoplasms in esophageal Lugol’s voiding lesions. Endoscopy international open. 2015 Aug;3(04):E281-8.
277. Chadwick G, Groene O, Hoare J, Hardwick RH, Riley S, Crosby TD, Hanna GB, Cromwell DA. A population-based, retrospective, cohort study of esophageal cancer missed at endoscopy. Endoscopy. 2014 Jul;46(07):553-60.
278. Lieberman, D. A., Oehlke, M., and Helfand, M. Risk factors for Barrett's esophagus in community-based practice. GORGE consortium. Gastroenterology Outcomes Research Group in Endoscopy. The American journal of gastroenterology 1997; 92 (8): 1293-1297.
279. Hardwick, R. H., Morgan, R. J., Warren, B. F., Lott, M., and Alderson, D. Brush cytology in the diagnosis of neoplasia in Barrett's esophagus. Diseases of the esophagus 1997; 10 (4): 233-237.
280. Caygill, C. P., Reed, P. I., Johnston, B. J., Hill, M. J., Ali, M. H., and Levi, S. A single centre's 20 years' experience of columnar-lined (Barrett's) oesophagus diagnosis. European journal of gastroenterology & hepatology 1999; 11 (12): 1355-1358.
281. Nandurkar, S., Talley, N. J., Martin, C. J., Ng, T. H., and Adams, S. Short segment Barrett's oesophagus: prevalence, diagnosis and associations. Gut 1997; 40 (6): 710-715.
282. May, A., Gunter, E., Roth, F., Gossner, L., Stolte, M., Vieth, M., and Ell, C. Accuracy of staging in early oesophageal cancer using high resolution endoscopy and high resolution endosonography: a comparative, prospective, and blinded trial. Gut 2004; 53 (5): 634-640.
283. Cameron, A. J. and Lomboy, C. T. Barrett's esophagus: age, prevalence, and extent of columnar epithelium. Gastroenterology 1992; 103 (4): 1241-1245.
284. Shakhatreh MH, Duan Z, Avila N, Naik A, Kramer JR, Hinojosa-Lindsey M, Chen J, El-Serag HB. Risk of Upper Gastrointestinal Cancers in Patients with Gastroesophageal Reflux Disease After a Negative Screening Endoscopy. *Clinical Gastroenterology and Hepatology* 2015; 13:280-286.
285. Lopez-Colombo, A., Jimenez-Toxqui, M., Gogeascoechea-Guillen, P. D., Melendez-Mena, D., Morales-Hernandez, E. R., Montiel-Jarquin, A. J., and Amaro-Balderas, E. Prevalence of esophageal inlet patch and clinical characteristics of the patients. Revista de gastroenterologia de Mexico 2018.
286. Wasielica-Berger, Justyna, Kemona, Andrzej, Kisluk, Joanna, Swidnicka-Siergiejko, Agnieszka, Rogalski, Pawel, Chwiesko, Adam, Kostrzewska, Maja, and Dabrowski, Andrzej. The added value of magnifying endoscopy in diagnosing patients with certain gastroesophageal reflux disease. Advances in medical sciences 2018. 63 (2) 359-366.
287. Smadi, Y., Deb, C., Bornstein, J., Safder, S., Horvath, K., and Mehta, D. Blind esophageal brushing offers a safe and accurate method to monitor inflammation in children and young adults with eosinophilic esophagitis. Diseases of the esophagus 2018.
288. Herrera Elizondo, J. L., Monreal Robles, R., Garcia Compean, D., Gonzalez Moreno, E. I., Borjas Almaguer, O. D., Maldonado Garza, H. J., and Gonzalez Gonzalez, J. A. Prevalence of Barrett's esophagus: An observational study from a gastroenterology clinic. Revista de gastroenterologia de Mexico 2017. 82 (4) 296-300.
289. Nwokediuko, Sylvester Chuks, Ijoma, Uchenna, and Okafor, Okechukwu. Esophageal Intraepithelial Neutrophil Infiltration is Common in Nigerian Patients With Non-Erosive Reflux Disease. Gastroenterology research 2011. 4 (1) 20-25.
290. Hutopila, Ionut, Constantin, A., and Copaescu, Catalin. Gastroesophageal Reflux Before Metabolic Surgery. Chirurgia (Bucharest, Romania: 1990) 2018. 113 (1) 101-107.
291. Heimgartner, Benjamin, Herzig, Marcus, Borbely, Yves, Kroll, Dino, Nett, Philipp, and Tutuian, Radu. Symptoms, endoscopic findings and reflux monitoring results in candidates for bariatric surgery. Digestive and liver disease 2017. 49 (7) 750-756.
292. Weijenborg, P. W., Smout, A. J. P. M., Krishnadath, K. K., Bergman, J. G. H. M., Verheij, J., and Bredenoord, A. J. Esophageal sensitivity to acid in patients with Barrett's esophagus is not related to preserved esophageal mucosal integrity. Neurogastroenterology and motility 2017. 29 (7).
293. Sekiguchi, Masau, Terauchi, Takashi, Kakugawa, Yasuo, Shimada, Naoki, Saito, Yutaka, and Matsuda, Takahisa. Performance of 18-fluoro-2-deoxyglucose positron emission tomography for esophageal cancer screening. World journal of gastroenterology 2017. 23 (15) 2743-2749.
294. Royston, Christine and Bardhan, Karna D. Adam, Eve and the reflux enigma: age and sex differences across the gastro-oesophageal reflux spectrum. European journal of gastroenterology & hepatology 2017. 29 (6) 634-639.
295. Woodland, Philip, Shen Ooi, Joanne Li, Grassi, Federica, Nikaki, Kornilia, Lee, Chung, Evans, James A., Koukias, Nikolaos, Triantos, Christos, McDonald, Stuart A., Peiris, Madusha, Aktar, Rubina, Blackshaw, L. Ashley, and Sifrim, Daniel. Superficial Esophageal Mucosal Afferent Nerves May Contribute to Reflux Hypersensitivity in Nonerosive Reflux Disease. Gastroenterology 2017. 153 (5) 1230-1239.
296. Tan, Mimi C., Murrey-Ittmann, Jackson, Nguyen, Theresa, Ketwaroo, Gyanprakash A., El-Serag, Hashem B., and Thrift, Aaron P. Risk Profiles for Barrett's Esophagus Differ between New and Prevalent, and Long- and Short-Segment Cases. PloS one 2016. 11 (12) e0169250.
297. Alkaddour, A., McGaw, C., Hritani, R., Palacio, C., Munoz, J. C., and Vega, K. J. Protective Propensity of Race or Environmental Features in the Development of Barrett's Esophagus in African Americans - A Single Center Pilot Study. Journal of the National Medical Association 2018.
298. Stephens, J. A., Fisher, J. L., Krok-Schoen, J. L., Baltic, R. D., Sobotka, H. L., and Paskett, E. D. Esophageal Adenocarcinoma: Opportunities for Targeted Prevention in Ohio. Clinical Medicine Insights: Gastroenterology 2018. 11.
299. Deng, P., Min, M., Dong, T., Bi, Y., Tang, A., and Liu, Y. Linked color imaging improves detection of minimal change esophagitis in non-erosive reflux esophagitis patients. Endoscopy International Open 2018. 6 (10) E1177-E1183.
300. Takeuchi, M., Goda, K., Oyama, T., Fujisaki, J., Ishihara, R., Takahashi, A., Takaki, Y., Hirasawa, D., Momma, K., Amano, Y., Yagi, K., Watanabe, G., and Ohkura, Y. The Japan esophageal society classification system to identify superficial neoplasms in Barrett's esophagus using magnifying endoscopy. Gastrointestinal endoscopy 2018. 87 (6 Supplement 1) AB277-AB278.
301. Lind, A., Siersema, P. D., Kusters, J. G., Konijn, T., Mebius, R. E., and Koenderman, L. The microenvironment in Barrett's esophagus tissue is characterized by high FOXP3 and RALDH2 levels. Frontiers in Immunology 2018. 9 (JUN) 1375.
302. Saarinen, T., Kettunen, U., Pietilainen, K. H., and Juuti, A. Is preoperative gastroscopy necessary before sleeve gastrectomy and Roux-en-Y gastric bypass? Surgery for Obesity and Related Diseases 2018. 14 (6) 757-762.
303. Cal, P., Deluca, L., Jakob, T., Lonardi, D., and Fernandez, E. Presurgical gerd evaluation of the bariatric patient. a cross sectional study. Surgical Endoscopy and Other Interventional Techniques 2018. 32 (1 Supplement 1) S313.
304. Quach, D. T., Nguyen, T. T., and Hiyama, T. Abnormal gastroesophageal flap valve is associated with high gastresophageal reflux disease questionnaire score and the severity of gastroesophageal reflux disease in vietnamese patients with upper gastrointestinal symptoms. Journal of neurogastroenterology and motility 2018. 24 (2) 226-232.
305. Nucci, D., Arcidiacono, D., Fassan, M., Antonello, A., Rugge, M., Morbin, T., Nardi, M., Maddalo, G., Agostini, M., Farinati, F., Alberti, A., and Realdon, S. Is the adherence to WCRF/AICR recommendations involved in barrett's esophagus onset and its progression to EAC? A retrospective analysis in a high-risk population. Digestive and Liver Disease 2018. 50 (2 Supplement 1) e103.
306. Lenoci, N., Amato, A., Andrealli, A., Paggi, S., Mandelli, G., Terreni, N., Mogavero, G., Rondonotti, E., Spinzi, G., Imperiali, G., and Radaelli, F. Olga-based staging and dysplasia relevance in 50-75 year old patients in open access endoscopy. Digestive and Liver Disease 2018. 50 (2 Supplement 1) e79.
307. Anuk, T., Kahramanca, S., and Kaya, O. Predictive parameters for barrett's esophagus: Percent body fat (PBF) and Visceral fat area (VFA) are more valuable than body mass index (BMI). Kuwait Medical Journal 2018. 50 (1) 72-76.
308. Barros, R., Pereira, D., Calle, C., Camilo, V., Cunha, A. I., David, L., Almeida, R., Dias-Pereira, A., and Chaves, P. Dynamics of SOX2 and CDX2 Expression in Barrett's Mucosa. Disease Markers 2016. 2016 () 1532791.
309. Offman, J. and Fitzgerald, R. C. Alternatives to Traditional Per-Oral Endoscopy for Screening. Gastrointestinal Endoscopy Clinics of North America 2017. 27 (3) 379-396.
310. Ojuka, D., Dindi, K., and Awori, M. Prevalence of esophageal adenocarcinoma. Annals of African Surgery 2017. 14 (2).
311. Schlottmann, F., Sadava, E. E., Reino, R., Galvarini, M., and Buxhoeveden, R. Preoperative endoscopy in bariatric patients may change surgical strategy. Acta Gastroenterologica Latinoamericana 2017. 47 (2) 117-121.
312. Paterson, A. L., Lao-Sirieix, P., O'Donovan, M., Debiram-Beecham, I., di, Pietro M., Miremadi, A., Attwood, S. E., Walter, F. M., Sasieni, P. D., and Fitzgerald, R. C. Range of pathologies diagnosed using a minimally invasive capsule sponge to evaluate patients with reflux symptoms. Histopathology 2017. 70 (2) 203-210.
313. Buas, M. F., Gu, H., Djukovic, D., Zhu, J., Onstad, L., Reid, B. J., Raftery, D., and Vaughan, T. L. Candidate serum metabolite biomarkers for differentiating gastroesophageal reflux disease, Barrett's esophagus, and high-grade dysplasia/esophageal adenocarcinoma. Metabolomics 2017. 13 (3) 23.
314. Kaplan, M., Tanoglu, A., Sakin, Y. S., Akyol, T., Oncu, K., Kara, M., and Yazgan, Y. Landmark reading alterations in patients with gastro-oesophageal reflux symptoms undergoing diagnostic gastroscopy. Arab Journal of Gastroenterology 2016. 17 (4) 176-180.
315. Soucy, G., Onstad, L., Vaughan, T. L., and Odze, R. D. Histologic Features Associated With Columnar-lined Esophagus in Distal Esophageal and Gastroesophageal Junction (GEJ) Biopsies From GERD Patients: A Community-based Population Study. The American journal of surgical pathology 2016. 40 (6) 827-835.
316. Makanga, W. and Nyaoncha, A. Upper gastrointestinal disease in Nairobi and Nakuru counties, Kenya; a two year comparative endoscopy study. Annals of African Surgery 2014. 11 (2) 35-39.

### Study design: Case-series/case report (n=9)

1. Shaheen, N. and Ransohoff, D. F. Gastroesophageal reflux, Barrett esophagus, and esophageal cancer: Clinical applications. Journal of the American Medical Association 2002; 287 (15): 1982-1986.
2. Fan, X., Nath, S., Gomez, G., and Raju, G. S. Fellows' corner. Practical Gastroenterology 2007; 31 (4): 103-.
3. El-Serag, H. B. Temporal trends in new and recurrent esophageal strictures in Department of Veterans Affairs. American Journal of Gastroenterology 2006; 101 (8): 1727-1733.
4. Clark, G. W., Smyrk, T. C., Burdiles, P., Hoeft, S. F., Peters, J. H., Kiyabu, M., Hinder, R. A., Bremner, C. G., and DeMeester, T. R. Is Barrett's metaplasia the source of adenocarcinomas of the cardia? Archives of surgery (Chicago, Ill.: 1960) 1994; 129 (6): 609-614.
5. Diop, P. S., Ndoye, J. M., Ndiaye, D., Ka, I., Dangou, J. M., and Fall, B. Oesophagus cancer in Senegal: Review of a series of 72 cases. Journal Africain d'Hepato-Gastroenterologie 2009; 3 (4): 190-194.
6. Peitz, U., Vieth, M., Ebert, M., Kahl, S., Schulz, H.-U., Roessner, A., and Malfertheiner, P. Small-bowel metaplasia arising in the remnant esophagus after esophagogastrostomy - A prospective study in patients with a history of total gastrectomy. American Journal of Gastroenterology 2005; 100 (9): 2062-2070.
7. Agha, F. P. and Keren, D. F. Barrett's esophagus complicating achalasia after esophagomyotomy. A clinical, radiologic, and pathologic study of 70 patients with achalasia and related motor disorders. Journal of clinical gastroenterology 1987; 9 (2): 232-237.
8. Vergouwe, Floor Wt, Gottrand, Madeleine, Wijnhoven, Bas Pl, IJsselstijn, Hanneke, Piessen, Guillaume, Bruno, Marco J., Wijnen, Rene Mh, and Spaander, Manon Cw. Four cancer cases after esophageal atresia repair: Time to start screening the upper gastrointestinal tract. World journal of gastroenterology 2018. 24 (9) 1056-1062.
9. Gamaletsou, Maria N. and Denning, David W. Gastroesophageal Reflux Disease and Pulmonary Diseases Associated with Aspergillosis: Is There a Connection? Mycopathologia 2017. 182 (11-12) 1125-1129.

### Study design: Cost-effectiveness/Markov model (n=12)

1. Gerson, L. B., Groeneveld, P. W., and Triadafilopoulos, G. Cost-effectiveness model of endoscopic screening and surveillance in patients with gastroesophageal reflux disease. Clinical Gastroenterology and Hepatology 2004; 2 (10): 868-879.
2. Inadomi, J. M., Sampliner, R., Lagergren, J., Lieberman, D., Fendrick, A. M., and Vakil, N. Screening and surveillance for Barrett esophagus in high-risk groups: A cost-utility analysis. Annals of Internal Medicine 2003; 138 (3): 176-186.
3. Stern, M. A., Fendrick, A. M., McDonnell, W. M., Gunaratnam, N., Moseley, R., and Chey, W. D. Screening for high-grade dysplasia in gastroesophageal reflux disease: Is it cost-effective? American Journal of Gastroenterology 2000; 95 (8): 2086-2093.
4. Benaglia, Tatiana, Sharples, Linda D., Fitzgerald, Rebecca C., and Lyratzopoulos, Georgios Health benefits and cost effectiveness of endoscopic and nonendoscopic cytosponge screening for Barrett's esophagus. Gastroenterology 2013; 144 (1): 62-73.
5. Raman, Anoop, Sternbach, Joel, Babajide, Azeesat, Sheth, Ketan, and Schwaitzberg, Steven D. When does testing for GERD become cost effective in an integrated health network? Surgical endoscopy 2010; 24 (6): 1245-1249.
6. Gerson, Lauren and Lin, Otto S. Cost-benefit analysis of capsule endoscopy compared with standard upper endoscopy for the detection of Barrett's esophagus. Clinical gastroenterology and hepatology 2007; 5 (3): 319-325.
7. Rubenstein, Joel H., Inadomi, John M., Brill, Joel V., and Eisen, Glenn M. Cost utility of screening for Barrett's esophagus with esophageal capsule endoscopy versus conventional upper endoscopy. Clinical gastroenterology and hepatology 2007; 5 (3): 312-318.
8. Esfandyari, Tuba, Potter, Jon W., and Vaezi, Michael F. Dysphagia: a cost analysis of the diagnostic approach. The American journal of gastroenterology 2002; 97 (11): 2733-2737.
9. Rubenstein, J. H. and Inadomi, J. M. Defining a clinically significant adverse impact of diagnosing Barrett's esophagus. Journal of Clinical Gastroenterology 2006; 40 (2): 109-115.
10. Ende, A. R., Higa, J. T., Singla, A., Choi, A. Y., Lee, A. B., Whang, S. G., Gravelle, K., D'Andrea, S., Bang, S. J., Schmidt, R. A., Yeh, M. M., and Hwang, J. H. Gastric intestinal metaplasia, dysplasia, and gastric cancer in a U.S. tertiary care population-who's at risk?. Gastrointestinal endoscopy 2016; 83 (5 SUPPL. 1): AB459-.
11. Nietert, P. J., Silverstein, M. D., Mokhashi, M. S., Kim, C. Y., Glenn, T. F., Marsi, V. A., Hawes, R. H., and Wallace, M. B. Cost effectiveness of screening a population with chronic gastroesophageal reflux. Gastrointestinal endoscopy 2003; 57 (3): 311-.
12. Heberle, Curtis R., Omidvari, Amir Houshang, Ali, Ayman, Kroep, Sonja, Kong, Chung Yin, Inadomi, John M., Rubenstein, Joel H., Tramontano, Angela C., Dowling, Emily C., Hazelton, William D., Luebeck, E Georg, Lansdorp-Vogelaar, Iris, and Hur, Chin. Cost Effectiveness of Screening Patients With Gastroesophageal Reflux Disease for Barrett's Esophagus With a Minimally Invasive Cell Sampling Device. Clinical gastroenterology and hepatology 2017. 15 (9) 1397-1404.

### Study design: Other (e.g., editorial, commentary, notes, letters, opinions) (n=102)

1. Danis, P. and Chumley, H. Clinical inquiries. Which patients with gastroesophageal reflux disease (GERD) should have esophagogastroduoudenoscopy (EGD)? The Journal of family practice 2001; 50 (8): 658-659.
2. Spechler, S. J. Guidelines for managing short-segment Barrett's esophagus. The American journal of managed care 2000; 6 (16 Suppl): S891-S894.
3. Falk, G. W. Updated guidelines for diagnosing and managing Barrett Esophagus. Gastroenterology and Hepatology 2016; 12 (7): 449-451.
4. Chan, C. and Chen, H. Barrett's oesophagus Does it matter? Medicine Today 2015; 16 (10): 49-51.
5. Talley, N. J. and Napthali, K. E. Endoscopy in symptomatic gastroesophageal reflux disease: Scoping out whomto target. JAMA Internal Medicine 2014; 174 (3): 465-466.
6. Rubenstein, J. H. Clinical prediction and screening for Barrett esophagus. Gastroenterology and Hepatology 2014; 10 (3): 187-189.
7. Armstrong, C. ACP publishes recommendations on upper endoscopy for gastroesophageal reflux disease. American family physician 2013; 88 (12): 865-.
8. Fitzgerald, R. C. and Rubenstein, J. H. Oracular guidance on clinical management of early neoplastic barrett's esophagus. Gastroenterology 2012; 143 (2): 282-284.
9. Raju, G. S. Advances in GERD: Esophageal perforations. Gastroenterology and Hepatology 2012; 8 (8): 548-551.
10. Klibansky, D. A., Gordon, S. R., and Gardner, T. B. Overutilization of endoscopic surveillance in nondysplastic Barrett's: Too much of a good thing? Gastroenterology 2012; 142 (7): 1614-1616.
11. Gerson, L. B. Are we ready for gender-based guidelines for Barrett's esophagus screening?. Gastroenterology 2011; 141 (6): 2271-2273.
12. Shaheen, N. J. Editorial: Should women with heartburn undergo screening upper endoscopy for prevention of cancer. American Journal of Gastroenterology 2011; 106 (2): 261-263.
13. Fass, R. Challenges associated with the treatment of patients with gastroparesis and GERD. Gastroenterology and Hepatology 2009; 5 (10): 7-8.
14. SSAT patient care guidelines: Management of Barrett's esophagus. Journal of Gastrointestinal Surgery 2007; 11 (9): 1213-1215.
15. Inadomi, J. M. Fear of Loss, Not Promise of Gain, Drives Practice Patterns in Barrett's Esophagus. Gastroenterology 2008; 134 (4): 1258-1260.
16. Spechler, S. J. A balancing view: To screen or not to screen: Scoping out the issues. American Journal of Gastroenterology 2004; 99 (12): 2295-2296.
17. Sampliner, R. E. Should patients with GERD be screened once at least for Barrett's epithelium? PRO: The need to screen GERD patients for Barrett's esophagus - A greater yield than surveillance. American Journal of Gastroenterology 2004; 99 (12): 2291-2293.
18. Moayyedi, P. Should patients with GERD be screened once at least for Barrett's epithelium? CON: Patients with GERD should be screened once at least for Barrett's esophagus - Medicine or magic?. American Journal of Gastroenterology 2004; 99 (12): 2293-2295.
19. Vij, R. and Triadafilopoulos, G. GERD: Could your patient be at risk for esophageal cancer? Journal of Respiratory Diseases 2003; 24 (1): 6-8.
20. Armstrong, D. Motion - All patients with GERD should be offered once in a lifetime endoscopy: Arguments for the motion. Canadian Journal of Gastroenterology 2002; 16 (8): 549-551.
21. Gastroesophageal reflux disease: Diagnostic and management approaches. Consultant 1999; 39 (11): 3122-3124.
22. Howden, C. W. Summary and recommendations. Gastroenterology International 1997; 10 (3): 131-132.
23. Ishaq, S. and Jankowski, J. A. Barrett's metaplasia: clinical implications. World journal of gastroenterology 2001; 7 (4): 563-565.
24. Sharma, P. and Katz, P. Endoscopic therapies for gastroesophageal reflux disease: Back in the game?. Gastroenterology 2015; 148 (2): 280-285.
25. Kim, G. H. Columnar-lined esophagus: Its prevalence and predictors in patients with gastroesophageal reflux disease. Journal of Neurogastroenterology and Motility 2013; 19 (2): 261-263.
26. Miller, M. Barrett's esophagus: Major issues uncertain and unsolved. Journal of the National Cancer Institute 2001; 93 (9): 674-675.
27. Prescott, L. M. Highlights of digestive disease week 2000. P and T 2000; 25 (8): 401-409.
28. Cuomo, R. Diagnosing gastro-oesophageal reflux: Endoscopy, pH-metry or empirical trial? Digestive and Liver Disease 2000; 32 (SUPPL. 3): S239-S241.
29. Cooper, G. S., Kou, T. D., and Chak, A. Receipt of previous diagnoses and endoscopy and outcome from esophageal adenocarcinoma: A population-based study with temporal trends. American Journal of Gastroenterology 2009; 104 (6): 1356-1362.
30. Gerson, L. B. Unsedated endoscopy: Ready for prime time? Commentary. Evidence-Based Gastroenterology 2007; 8 (3): 51-53.
31. Katz, P. O. Esophageal cancer in GERD patients. Reviews in gastroenterological disorders 2002; 2 (2): 87-89.
32. Odze, R. Cytokeratin 7/20 immunostaining: Barrett's oesophagus or gastric intestinal metaplasia? Lancet 2002; 359 (9319): 1711-1713.
33. Sampliner, R. E. Definitional confusion: When Barrett's esophagus is not Barrett's and not Barrett's is Barrett's. Prevalence of metaplasia at the gastro-oesophageal junction. American Journal of Gastroenterology 1995; 90 (7): 1181-1182.
34. Caldwell, M. T. P., Lawlor, P., Byrne, P. J., Walsh, T. N., and Hennessy, T. P. J. Ambulatory oesophageal bile reflux monitoring in Barrett's oesophagus. British Journal of Surgery 1995; 82 (5): 657-660.
35. Fardoun, T., Peyronnet, B., Pery, C., Sulpice, L., Boudjema, K., and Meunier, B. Gastrointestinal stromal tumor of the esophagus. Diseases of the esophagus 2013; 26 (3): 336-337.
36. Bresalier, Robert Management of Barrett's esophagus: cases and questions. Seminars in oncology 2005; 32 (6 Suppl 9): S2-S5.
37. Cooper, Gregory S., Yuan, Zhong, Chak, Amitabh, and Rimm, Alfred A. Association of prediagnosis endoscopy with stage and survival in adenocarcinoma of the esophagus and gastric cardia. Cancer 2002; 95 (1): 32-38.
38. Bapaye, A., Dubale, N., Pujari, R., Kulkarni, A., Jajoo, Naval R., Vyas, V., and Mahadik, M. Peroral endoscopic pyloromyotomy for delayed postoperative gastroparesis. Endoscopy 2015; 47 (): E581-E582.
39. Zalar, A., Haddouche, B., Antonietti, M., Alhameedi, R., Iwanicki-Caron, I., Lecleire, S., and Ducrotte, P. Lack of correlation between morbid obesity and severe gastroesophageal reflux disease in candidates for bariatric surgery: Results of a large prospective study. Obesity Surgery 2013; 23 (11): 1939-1941.
40. Mergener, K. The impact of the Affordable Care Act on the future of endoscopy practice. Gastroenterology and Hepatology 2013; 9 (11): 749-751.
41. Triadifilopoulos, G. Cytosponge for Barrett's esophagus screening: When smart science matches simplicity. Gastroenterology 2011; 141 (2): 766-768.
42. Kuipers, E. J. Barrett Esophagus and life expectancy: Implications for screening? Gastroenterology and Hepatology 2011; 7 (10): 689-691.
43. Triadafilopoulos, G., Lombard, C. M., and Jobe, B. A. "War and peace" with Barrett's esophagus. Digestive Diseases and Sciences 2011; 56 (4): 988-992.
44. Wong, R. K. H. and Altekruse, S. F. Editorial: Where are you and how do we find you the dilemma of identifying Barrett's epithelium before adenocarcinoma of the esophagus. American Journal of Gastroenterology 2009; 104 (6): 1363-1365.
45. Atkinson, M. and Chak, A. Unsedated small-caliber endoscopy - A new screening and surveillance tool for Barrett's esophagus? Commentary. Nature Clinical Practice Gastroenterology and Hepatology 2007; 4 (8): 426-427.
46. Gerson, L. B., Groeneveld, P. W., Triadafilopoulos, G., and Inadomi, J. M. Barrett esophagus: More questions than answers. Evidence-Based Gastroenterology 2005; 6 (1): 4-6.
47. Wetscher, G. J. Selected Commentary to: "Ineffective oesophageal motility does not affect the clinical outcome of open Nissen fundoplication" (Munitiz V, Ortiz A, Martinez de Haro LF, Molina J, Parrilla P; Br J Surg 91: 1010-1014, 2004). European Surgery - Acta Chirurgica Austriaca 2004; 36 (6): 388-390.
48. Adami, H.-O. and Trichopoulos, D. Obesity and mortality from cancer. New England Journal of Medicine 2003; 348 (17): 1623-1624.
49. Wei, J. T., Shaheen, N. J., Inadomi, J., and Vakil, N. Dollars and sense in preventing esophageal cancer. Gastroenterology 2003; 125 (4): 1268-1270.
50. Mui, A. C. W. Barrett's oesophagus. Hong Kong Practitioner 2002; 24 (12): 594-603.
51. Oberg, S. and Mason, R. J. Is there a surgical nirvana for Barett's esophagus? American Journal of Gastroenterology 1996; 91 (12): 2630-2631.
52. Al Dulaimi, David Recent advances in oesophageal diseases. Gastroenterology and hepatology from bed to bench 2014; 7 (3): 186-189.
53. Buscaglia, J. M. Issue Highlights. Clinical Gastroenterology and Hepatology 2015; 13 (13): 2205-2208.
54. Jo, Y. New consensus on the management of Barrett's dysplasia and early stage esophageal adenocarcinoma: Limited evidence, but best available guidance. Journal of neurogastroenterology and motility 2012; 18 (4): 455-456.
55. Fock, K. M. GERD, NERD and Barrett's esophagus. Digestion 2009; 79 (3 SUPPL. 1): 59-.
56. Greenberger, N. J. and Sharma, P. Update in gastroenterology and hepatology. Annals of internal medicine 2006; 145 (4): 294-298.
57. Freston, J., Orlando, R., Galmiche, J. P., Scheiman, J., Asaka, M., and Chan, F. Discussion. Drugs 2006; 66 (SUPPL. 1): 29-33.
58. Marchal, F. ORL. Medecine et Hygiene 2004; 62 (2465): 121-128.
59. Henteleff, H. J. A., Darling, G., and McKenzie, M. Canadian Association of General Surgeons Evidence Based Reviews in Surgery. 6. "GERD" as a risk factor for esophageal cancer. Canadian Journal of Surgery 2003; 46 (3): 208-210.
60. Vakil, N., Talley, N., Malfertheiner, P., Fass, R., Tytgat, G. N. J., Laine, L. A., Jones, R., Hawkey, C. J., Armstrong, D., Lagergren, J., Dent, J., Lundell, L., and Kahrilas, P. J. Session 4: Long-term medical management. European Journal of Gastroenterology and Hepatology 2001; 13 (SUPPL. 3): S67-S89.
61. De Jonge, P. J. F. and Hvid-Jensen, F. Barrett's oesophagus: Size does matter. Gut 2016; 65 (2): 189-190.
62. Corley, D. Current surveillance and therapeutic options for barrett esophagus. Gastroenterology and Hepatology 2008; 4 (3): 183-186.
63. The role of endoscopy in the management of GERD. Gastrointestinal endoscopy 1999; 49 (6): 834-835.
64. Lagergren, J. Oesophageal cancer in 2014: Advances in curatively intended treatment. Nature Reviews Gastroenterology and Hepatology 2015; 12 (2): 74-75.
65. Jankowski, J. A. and Satsangi, J. Barrett's esophagus: Evolutionary insights from genomics. Gastroenterology 2013; 144 (4): 667-669.
66. Cadiot, G. On-demand therapy for gastroesophageal reflux disease: Cons. Hepato-Gastro 2003; 10 (5): 388-391.
67. Lord, R. V., Bowrey, D. J., and Blom, D. Barrett's esophagus: a surgical disease? The American journal of gastroenterology 2000; 95 (11): 3302-3305.
68. Odze, Robert D. Unraveling the mystery of the gastroesophageal junction: a pathologist's perspective. The American journal of gastroenterology 2005; 100 (8): 1853-1867.
69. Schaffner, J. A., Litin, S. C., and Bundrick, J. B. Clinical pearls in gastroenterology (2013). Disease-a-Month 2014; 60 (7): 345-353.
70. Aro, P. and Ronkainen, J. Evolution of GERD over 5 years. Alimentary Pharmacology and Therapeutics 2012; 35 (3): 393-394.
71. Pope, C. E. Ruminations. Gullet 1992; 2 (4): 171-172.
72. The Canadian Association of Gastroenterology (CAG) and the Canadian Digestive Health Foundation (CDHF) Annual Scientific Conference: Canadian Digestive Diseases Week (CDDW) February 27 – March 1, 2004 Banff, Alberta. Retrieved from <https://www.cag-acg.org/images/news/capsule_endoscopy_cddw_2004.pdf>
73. Capsule Endoscopy. Retrieved from <https://www.cag-acg.org/images/news/march_21_2006_capsule_endoscopy_medical_post.pdf>
74. Letter from the canadian association of gastroenterology. Retrieved from <https://www.cag-acg.org/images/news/compus_gerd_feedback_17april2006.pdf>
75. Updates in Cancer screening. Retrieved from <https://www.cag-acg.org/images/news/june_2006_updates_in_cancer_screening_medical_post.pdf>
76. David Hodges. CDDW: Rural FP able to provide endoscopic services. The Medical Post, March 2007. Retrieved from <https://www.cag-acg.org/images/news/march_13_2007_rural_fp_endoscopic_services_medical_post.pdf>
77. David Hodges. CDDW: Rural general surgeons' endoscopy burden points to training disconnec Endoscopy- Medical post. Retrieved from <https://www.cag-acg.org/images/news/march_13_2007_rural_general_surgeons_endoscopy_disconnect_medical_post.pdf>
78. Armstrong D. Promoting access and quality in endoscopic services. Qmentum Quarterly 2011; 3(4):24-8.
79. Bhat YM, Dayyeh BK, Chauhan SS, Gottlieb KT, Hwang JH, Komanduri S, Konda V, Lo SK, Manfredi MA, Maple JT, Murad FM. High-definition and high-magnification endoscopes. Gastrointestinal endoscopy. 2014 Dec 1;80(6):919-27.
80. American Society for Gastrointestinal Endoscopy. Endoscopic techniques offer hope for throat cancer patients . December 2015. Retrieved from <https://medicalxpress.com/news/2015-12-endoscopic-techniques-throat-cancer-patients.html>
81. American Society for Gastrointestinal Endoscopy. Radiofrequency ablation and complete endoscopic resection equally effective for dysplastic barrett’s esophagus. 21 May, 2014. Retrieved from <https://www.eurekalert.org/pub_releases/2014-05/asfg-raa052114.php>
82. American Society for Gastrointestinal Endoscopy. Screening for esophageal disease with unsedated transnasal endoscopy is safe and feasible.10 May, 2012. Retrieved from <https://www.eurekalert.org/pub_releases/2012-05/asfg-sfe051012.php>
83. American Society for Gastrointestinal Endoscopy. Study compares narrow band imaging to chromoendoscopy for the detection of dysplasia in IBD patients. October 12, 2011. Retrieved from <https://www.sciencedaily.com/releases/2011/10/111012161304.htm>
84. Adam Harris. Guidance on the indications for diagnostic upper gi endoscopy, flexible sigmoidoscopy and colonoscopy. British Society of Gastroenterology, 2013. Retrieved from <http://www.bsg.org.uk/clinical-guidance/endoscopy/guidance-on-the-indications-for-diagnostic-upper-gi-endoscopy-flexible-sigmoidoscopy-and-colonoscopy.html>
85. British Society of Gastroenterology.Tables for the bsg commissioning Guide. Retrieved from <http://www.bsg.org.uk/clinical/general/commissioning-report.html>
86. British Society of Gastroenterology. IV Barrets Oesophagus.
87. Shaheen NJ, Weinberg DS. Upper endoscopy for gastroesophageal reflux disease. Annals of Internal Medicine. 2013 Mar 19;158(6):503-4.
88. Recommendation AC. Upper Endoscopy for Gastroesophageal Reflux: Review of the Performance Measures by the Performance Measurement Committee of the American College of Physicians.
89. Canadian Cancer Society, Esophageal Cancer. Understanding your diagnosis. Retrieved from <http://www.cancer.ca/en/support-and-services/resources/publications/?region=on>
90. Canadian Agency for Drugs and Technologies in Health (CADTH). Endoscopic Ultrasound for the Diagnosis of Disease and Staging of Cancers in Adult Patients with Gastroenterological or Oncological Disease: Guidelines. 26 February, 2014. Retrieved from <https://www.cadth.ca/media/pdf/htis/mar-2014/RB0653%20Endoscopic%20Ultrasound%20Final.pdf>
91. Hailey D. Endoscope-based treatments for gastroesophageal reflux disease. Issues in emerging health technologies. 2004 Mar; (54):1-4.
92. MSAC, Medical Services Advisory Committee. Endoscopic Ultrasound for Evaluating Pancreatic, Gastric, Esophageal and Hepatobiliary Neoplasms. MSAC 1072 Assessment report. ISBN 1-74186-199-3. Issue 2008-048. Retrieved from <http://www.msac.gov.au/internet/msac/publishing.nsf/Content/DAFBB85D163DAB7ACA25801000123B51/$File/1072-Assessment-Report.pdf>
93. HAS, French National Authority for Health (Haute Autorité de Santé-HAS) . Assessment of real-time optical endomicroscopy performed during mapping of Barrett's esophagus (BE) (for diagnosis of BE-associated early neoplasia). ISBN number: 978-2-11-138124-7. Retrieved from <http://www.has-sante.fr/portail/jcms/c_1731777/fr/evaluationde-l-endomicroscopie-optique-realisee-lors-de-la-cartographie-d-un-endo-brachy-oesophage-aide-au-diagnosticprecoce-du-cancer-superficiel-de-l-oesophage-rapport-d-evaluation>
94. Saskatchewan Cancer Agency. Provincial Esophageal Cancer and Gastro-esophageal junction Cancer Treatment Guidelines. March 12, 2011. Retrieved from <https://www.cma.ca/En/Pages/clinical-practice-guidelines.aspx>
95. Stahl M, Oliveira J. Esophageal cancer: ESMO clinical recommendations for diagnosis, treatment and follow-up. Annals of oncology. 2008 May 1; 19(suppl_2):ii21-2.
96. Vakil N. Endoscopy in GERD: Boondoggle, diagnostic test, or risk management tool? The American journal of gastroenterology. 2008 Feb 1; 103(2):276.
97. Canadian Associaton of Gastroenterology. Five Things Physicians and Patients Should Question. Choosing Wisely Canada. Retrieved from: <https://choosingwiselycanada.org/wp-content/uploads/2017/02/Gastroenterology.pdf>
98. Dubé C. Use of the endoscopy global Rating scale by endoscopy services in Canada. Canadian Journal of Gastroenterology and Hepatology 2013; 27(12):684-5.
99. Hollingworth R, Dubé C. The canadian association of gastroenterology endoscopy Quality Initiative: Leading the wave. Canadian Journal of Gastroenterology and Hepatology. 2009;23(7):506-7.
100. Canadian Digestive Helath Foundation. Understanding Barrett’s Esophagus. Retrieved from <http://cdhf.ca/bank/document_en/3understanding-barretts-esophagus-.pdf#zoom=100>
101. Bjorkman DJ, Popp Jr JW. Measuring the quality of endoscopy. The American journal of gastroenterology. 2006 Apr 1;101(4):864.
102. American Society for Gastrointestinal Endoscopy, American College of Gastroenterology and the American Gastroenterological Association. Statement- Universal adoption of capnography for moderate sedation in adults undergoing upper endoscopy and colonoscopy has not been shown to improve patient safety or clinical outcomes and significantly increases costs for moderate sedation. Retrieved from <https://www.asge.org/docs/default-source/education/practice_guidelines/doc-90dc9b63-593d-48a9-bec1-9f0ab3ce946a.pdf?sfvrsn=6>

### Does not evaluate a screening modality/technique of interest (n=13)

1. Orbelo, Diana M., Enders, Felicity T., Romero, Yvonne, Francis, Dawn L., Achem, Sami R., Dabade, Tushar S., Crowell, Michael D., Geno, Debra M., DeJesus, Ramona S., Namasivayam, Vikneswaran, Adamson, Steven C., Arora, Amindra S., Majka, Andrew J., Alexander, Jeffrey A., Murray, Joseph A., Lohse, Matthew, Diehl, Nancy N., Fredericksen, Mary, Jung, Kee Wook, Houston, Margaret S., O'Neil, Angela E., and Katzka, David A. Once-daily omeprazole/sodium bicarbonate heals severe refractory reflux esophagitis with morning or nighttime dosing. Digestive Diseases and Sciences 2015; 60 (1): 146-162.
2. Pilger, Diogo Andre, Lopez, Patricia Luciana da Costa, Segal, Fabio, and Leistner-Segal, Sandra Analysis of R213R and 13494 g--> a polymorphisms of the p53 gene in individuals with esophagitis, intestinal metaplasia of the cardia and Barrett's Esophagus compared with a control group. Genomic medicine 2007; 1 (1-2): 57-63.
3. Drahos, J., Ricker, W., Pfiffer, R. M., Warren, J. L., and Cook, M. B. Metabolic syndrome is associated with an increased risk of Barrett's esophagus in those without symptomatic reflux. Cancer Research 2013; 73 (8 SUPPL. 1): no-.
4. Stein, H. J., Hoeft, S., DeMeester, T. R., and Mathisen, D. G. Functional foregut abnormalities in Barrett's esophagus. Journal of Thoracic and Cardiovascular Surgery 1993; 105 (1): 107-111.
5. Sugimoto, M., Nishino, M., Kodaira, C., Yamade, M., Ikuma, M., Tanaka, T., Sugimura, H., Hishida, A., and Furuta, T. Esophageal mucosal injury with low-dose aspirin and its prevention by rabeprazole. J Clin Pharmacol 2010; 50 (3): 320-330.
6. Russell IT, Edwards RT, Gliddon AE, Ingledew DK, Russell D, Whitaker R, Yeo ST, Attwood SE, Barr H, Nanthakumaran S, Park KG. cancer of oesophagus or gastricus–new assessment of technology of endosonography (cognate): report of pragmatic randomised trial.
7. Ireland, C. J., Fielder, A. L., Thompson, S. K., Laws, T. A., Watson, D. I., and Esterman, A. Development of a risk prediction model for Barrett's esophagus in an Australian population. Diseases of the esophagus 2017. 30 (11): 1-8.
8. Ness-Jensen, Eivind, Gottlieb-Vedi, Eivind, Wahlin, Karl, and Lagergren, Jesper. All-cause and cancer-specific mortality in GORD in a population-based cohort study (the HUNT study). Gut 2018. 67 (2) 209-215.
9. Maret-Ouda, J., Wahlin, K., Artama, M., Brusselaers, N., Farkkila, M., Lynge, E., Mattsson, F., Pukkala, E., Romundstad, P., Tryggvadottir, L., Von Euler-Chelpin, M., and Lagergren, J. Risk of Esophageal Adenocarcinoma after Antireflux Surgery in Patients with Gastroesophageal Reflux Disease in the Nordic Countries. JAMA Oncology 2018.
10. Filiberti, R. A., Fontana, V., De, Ceglie A., Blanchi, S., Grossi, E., Della, Casa D., Lacchin, T., De, Matthaeis M., Ignomirelli, O., Cappiello, R., Rosa, A., Foti, M., Laterza, F., D'Onofrio, V., Iaquinto, G., and Conio, M. Alcohol consumption pattern and risk of Barrett's oesophagus and erosive oesophagitis: An Italian case-control study. British Journal of Nutrition 2017. 117 (8) 1151-1161.
11. Thrift, A. P., Vaughan, T., Anderson, L. A., Whiteman, D., and El-Serag, H. B. External validation of the Michigan Barrett's esophagus prediction tool (M-BERET). Gastroenterology 2017. 152 (5 Supplement 1) S453.
12. Winzer, B. M., Paratz, J. D., Whitehead, J. P., Whiteman, D. C., and Reeves, M. M. The feasibility of an exercise intervention in males at risk of oesophageal adenocarcinoma: a randomized controlled trial. PloS one 2015. 10 (2) e0117922.
13. Prevalence and Predictors of Gastroesophageal Reflux Complications in Community Subjects. Digestive diseases and sciences. (pp 1-8), 2016.Date of publication: 10.aug 2016. 2016.

### Evaluates molecular (e.g., cells, genes) and other biomarkers (e.g., blood, stool, urine) (n=12)

1. Bansal, A., Hong, X., Lee, I.-H., Krishnadath, K. K., Mathur, S. C., Gunewardena, S., Rastogi, A., Sharma, P., and Christenson, L. K. MicroRNA expression can be a promising strategy for the detection of barrett's esophagus: A pilot study. Clinical and Translational Gastroenterology 2014: 5(12):e65.
2. Nancarrow, D. J., Clouston, A. D., Smithers, B. M., Gotley, D. C., Drew, P. A., Watson, D. I., Tyagi, S., Hayward, N. K., and Whiteman, D. C. Whole genome expression array profiling highlights differences in mucosal defense genes in barrett's esophagus and esophageal adenocarcinoma. PloS One 2011; 6 (7): e22513.
3. Cabibi, D., Fiorentino, E., Pantuso, G., Mastrosimone, A., Callari, C., Cacciatore, M., Campione, M., and Aragona, F. Keratin 7 expression as an early marker of reflux-related columnar mucosa without intestinal metaplasia in the esophagus. Medical Science Monitor 2009; 15 (5): CR203-CR210.
4. Chang, C.-L., Lao-Sirieix, P., Save, V., Mendez, G. D. L. C., Laskey, R., and Fitzgerald, R. C. Retinoic acid-induced glandular differentiation of the oesophagus. Gut 2007; 56 (7): 906-917.
5. Liu, G.-S., Gong, J., Cheng, P., Zhang, J., Chang, Y., and Qiang, L. Distinction between short-segment Barrett's esophageal and cardiac intestinal metaplasia. World journal of gastroenterology 2005; 11 (40): 6360-6365.
6. Salo, J. A., Kivilaakso, E. O., Klviluoto, T. A., and Virtanen, I. O. Cytokeratin profile suggests metaplastic epithelial transformation in Barrett's oesophagus. Annals of Medicine 1996; 28 (4): 305-309.
7. Buas, Matthew F., Onstad, Lynn, Levine, David M., Risch, Harvey A., Chow, Wong Ho, Liu, Geoffrey, Fitzgerald, Rebecca C., Bernstein, Leslie, Ye, Weimin, Bird, Nigel C., Romero, Yvonne, Casson, Alan G., Corley, Douglas A., Shaheen, Nicholas J., Wu, Anna H., Gammon, Marilie D., Reid, Brian J., Hardie, Laura J., Peters, Ulrike, Whiteman, David C., and Vaughan, Thomas L. MiRNA-Related SNPs and Risk of Esophageal Adenocarcinoma and Barrett's Esophagus: Post Genome-Wide Association Analysis in the BEACON Consortium. PloS one 2015; 10 (6): e0128617-.
8. Gaj, Pawel, Mikula, Michal, Wyrwicz, Lucjan S., Regula, Jaroslaw, and Ostrowski, Jerzy Barrett's esophagus associates with a variant of IL23R gene. Acta biochimica Polonica 2008; 55 (2): 365-369.
9. Maddalo, Gemma, Fassan, Matteo, Cardin, Romilda, Piciocchi, Marika, Marafatto, Filippo, Rugge, Massimo, Zaninotto, Giovanni, Pozzan, Caterina, Castoro, Carlo, Ruol, Alberto, Biasiolo, Alessandra, and Farinati, Fabio. Squamous Cellular Carcinoma Antigen Serum Determination as a Biomarker of Barrett Esophagus and Esophageal Cancer: A Phase III Study. Journal of clinical gastroenterology 2018. 52 (5) 401-406.
10. Rafat, M. N., Younus, H. A. E., EL-Shorpagy, M. S., Hemida, M. H., EL Shahawy, M. S., and El Sayed Atiia, A. A. E. A. Adiponectin level changes among Egyptians with gastroesophageal reflux disease. JGH Open 2018. 2 (1) 21-27.
11. Petrick, J. L., Falk, R. T., Hyland, P. L., Caron, P., Pfeiffer, R. M., Wood, S. N., Dawsey, S. M., Abnet, C. C., Taylor, P. R., Guillemette, C., Murray, L. J., Anderson, L. A., and Cook, M. B. Association between circulating levels of sex steroid hormones and esophageal adenocarcinoma in the FINBAR Study. PloS one 2018. 13 (1) e0190325.
12. Kauttu, T., Mustonen, H., Vainionp+. Disintegrin and metalloproteinases (ADAMs) expression in gastroesophageal reflux disease and in esophageal adenocarcinoma. Clinical & translational oncology 2017. 19 (1) 58G.

### Does not evaluate of comparator of interest (n=6)

1. Fang, C., Huang, Q., Lu, L., Shi, J., Sun, Q., Xu, G. F., Gold, J., Mashimo, H., and Zou, X. P. Risk factors of early proximal gastric carcinoma in Chinese diagnosed using WHO criteria. Journal of Digestive Diseases 2015; 16 (6): 327-336.
2. Khalaf, N., Nguyen, T., Ramsey, D., and El-Serag, H. B. Nonsteroidal Anti-inflammatory Drugs and the Risk of Barrett's Esophagus. Clinical Gastroenterology and Hepatology 2014; 12 (11): 1832-1839.
3. Iyer, P. G., Borah, B. J., Heien, H. C., Das, A., Cooper, G. S., and Chak, A. Association of Barrett's Esophagus With Type II Diabetes Mellitus: Results From a Large Population-based Case-Control Study. Clinical Gastroenterology and Hepatology 2013; 11 (9): 1108-1114.
4. Emken, Birgitte Elise, Lundell, Lars R., Wallin, Lene, Myrvold, Helge E., Engstrom, Cecilia, Montgomery, Madeleine, Malm, Anders R., Lind, Tore, Hatlebakk, Jan G., and SOPRAN Study Group. Effects of omeprazole or anti-reflux surgery on lower oesophageal sphincter characteristics and oesophageal acid exposure over 10 years. Scandinavian Journal of Gastroenterology 2016: 1-7.
5. Haider, Syed H., Kwon, Sophia, Lam, Rachel, Lee, Audrey K., Caraher, Erin J., Crowley, George, Zhang, Liqun, Schwartz, Theresa M., Zeig-Owens, Rachel, Liu, Mengling, Prezant, David J., and Nolan, Anna. Predictive Biomarkers of Gastroesophageal Reflux Disease and Barrett's Esophagus in World Trade Center Exposed Firefighters: a 15 Year Longitudinal Study. Scientific reports 2018. 8 (1) 3106.
6. Guerrero Garcia Hall, Mats, Wenner, Jorgen, and Oberg, Stefan. The normal squamocolumnar junction is circumferentially even and minimal irregularities are manifestations of gastroesophageal acid reflux. Scandinavian journal of gastroenterology 2017. 52 (3) 270-275.

### No comparator (e.g., all participants received the same test/number/interval) (n=96)

1. Leggett, Cadman L., Gorospe, Emmanuel C., Calvin, Andrew D., Harmsen, William S., Zinsmeister, Alan R., Caples, Sean, Somers, Virend K., Dunagan, Kelly, Lutzke, Lori, Wang, Kenneth K., and Iyer, Prasad G. Obstructive sleep apnea is a risk factor for Barrett's esophagus. Clinical gastroenterology and hepatology 2014; 12 (4): 583-588.
2. Rubenstein, Joel H., Inadomi, John M., Scheiman, James, Schoenfeld, Philip, Appelman, Henry, Zhang, Min, Metko, Val, and Kao, John Y. Association between Helicobacter pylori and Barrett's esophagus, erosive esophagitis, and gastroesophageal reflux symptoms. Clinical gastroenterology and hepatology 2014; 12 (2): 239-245.
3. McIntire, Maria G., Soucy, Genevieve, Vaughan, Thomas L., Shahsafaei, Aliakbar, and Odze, Robert D. MUC2 is a highly specific marker of goblet cell metaplasia in the distal esophagus and gastroesophageal junction. The American journal of surgical pathology 2011; 35 (7): 1007-1013.
4. Tsimogiannis, Konstantinos E., Pappas-Gogos, George K., Benetatos, Nikolaos, Tsironis, Demitrios, Farantos, Charalampos, and Tsimoyiannis, Evangelos C. Laparoscopic Nissen fundoplication combined with posterior gastropexy in surgical treatment of GERD. Surgical endoscopy 2010; 24 (6): 1303-1309.
5. Csendes, A., Burdiles, P., Korn, O., Braghetto, I., Huertas, C., and Rojas, J. Late results of a randomized clinical trial comparing total fundoplication versus calibration of the cardia with posterior gastropexy. The British journal of surgery 2000; 87 (3): 289-297.
6. Csendes, A., Smok, G., Burdiles, P., Sagastume, H., Rojas, J., Puente, G., Quezada, F., and Korn, O. 'Carditis': an objective histological marker for pathologic gastroesophageal reflux disease. Diseases of the esophagus 1998; 11 (2): 101-105.
7. Filiberti, R., Blanchi, S., Ravelli, P., Conio, M., Munizzi, F., Aste, H., Giacosa, A., Ferraris, R., Fracchia, M., Pera, A., Marchi, S., Costa, F., Rivelli, P., Missale, G., Cestari, R., Lapertosa, G., Fiocca, R., D'Onofrio, V., Iaquinto, G., Sablich, R., Lacchin, T., Benedetti, G., and Gusmaroli, R. Risk factors for Barrett's esophagus: A case-control study. International Journal of Cancer 2002; 97 (2): 225-229.
8. Kim, S. L., Waring, J. P., Spechler, S. J., Sampliner, R. E., Doos, W. G., Krol, W. F., and Williford, W. O. Diagnostic inconsistencies in Barrett's esophagus. Gastroenterology 1994; 107 (4): 945-949.
9. Tseng, Ping Huei, Yang, Wei Shiung, Liou, Jyh Ming, Lee, Yi Chia, Wang, Hsiu Po, Lin, Jaw Town, and Wu, Ming Shiang Associations of Circulating Gut Hormone and Adipocytokine Levels with the Spectrum of Gastroesophageal Reflux Disease. PloS one 2015; 10 (10): e0141410-.
10. Khalaf, N., Ramsey, D., Kramer, J. R., and El-Serag, H. B. Personal and family history of cancer and the risk of Barrett's esophagus in men. Diseases of the esophagus 2015; 28 (3): 283-290.
11. Goldberg, Aaron, Gerkin, Richard D., and Young, Michele Medical Prevention of Barrett's Esophagus: Effects of Statins, Aspirin, Non-aspirin NSAIDs, Calcium, and Multivitamins. Digestive Diseases and Sciences 2015; 60 (7): 2058-2062.
12. Shinkai, Hirohiko, Iijima, Katsunori, Koike, Tomoyuki, Abe, Yasuhiko, Dairaku, Naohiro, Inomata, Yoshifumi, Kayaba, Syoichi, Ishiyama, Fumitake, Oikawa, Tomoyuki, Ohyauchi, Motoki, Ito, Hirotaka, Asonuma, Sho, Hoshi, Tatsuya, Kato, Katsuaki, Ohara, Shuichi, and Shimosegawa, Tooru Association between the body mass index and the risk of Barrett's esophagus in Japan. Digestion 2014; 90 (1): 1-9.
13. Rubenstein, Joel H., Morgenstern, Hal, McConell, Daniel, Scheiman, James M., Schoenfeld, Philip, Appelman, Henry, McMahon, Laurence F. J., Kao, John Y., Metko, Val, Zhang, Min, and Inadomi, John M. Associations of diabetes mellitus, insulin, leptin, and ghrelin with gastroesophageal reflux and Barrett's esophagus. Gastroenterology 2013; 145 (6): 1237-5.
14. Lin, Derek, Kramer, Jennifer R., Ramsey, David, Alsarraj, Abeer, Verstovsek, Gordana, Rugge, Massimo, Parente, Paola, Graham, David Y., and El-Serag, Hashem B. Oral bisphosphonates and the risk of Barrett's esophagus: case-control analysis of US veterans. The American journal of gastroenterology 2013; 108 (10): 1576-1583.
15. Cummings, Linda C., Shah, Ninad, Maimone, Santo, Salah, Wajeeh, Khiani, Vijay, and Chak, Amitabh Barrett's esophagus and the risk of obstructive sleep apnea: a case-control study. BMC gastroenterology 2013; 13: 82-.
16. Kramer, Jennifer R., Fischbach, Lori A., Richardson, Peter, Alsarraj, Abeer, Fitzgerald, Stephanie, Shaib, Yasser, Abraham, Neena S., Velez, Maria, Cole, Rhonda, Anand, Bhupinderjit, Verstovsek, Gordana, Rugge, Massimo, Parente, Paola, Graham, David Y., and El-Serag
17. Hashem B. Waist-to-hip ratio, but not body mass index, is associated with an increased risk of Barrett's esophagus in white men. Clinical gastroenterology and hepatology 2013; 11 (4): 373-381.
18. Omer, Zehra B., Ananthakrishnan, Ashwin N., Nattinger, Kevin J., Cole, Elisabeth B., Lin, Jesse J., Kong, Chung Yin, and Hur, Chin Aspirin protects against Barrett's esophagus in a multivariate logistic regression analysis. Clinical gastroenterology and hepatology 2012; 10 (7): 722-727.
19. Fisichella, P. Marco, Davis, Christopher S., Gagermeier, James, Dilling, Daniel, Alex, Charles G., Dorfmeister, Jennifer A., Kovacs, Elizabeth J., Love, Robert B., and Gamelli, Richard L. Laparoscopic antireflux surgery for gastroesophageal reflux disease after lung transplantation. The Journal of surgical research 2011; 170 (2): e279-e286.
20. Matsuzaki, Juntaro, Suzuki, Hidekazu, Asakura, Keiko, Saito, Yoshimasa, Hirata, Kenro, Takebayashi, Toru, and Hibi, Toshifumi Etiological difference between ultrashort- and short-segment Barrett's esophagus. Journal of gastroenterology 2011; 46 (3): 332-338.
21. Akiyama, Tomoyuki, Inamori, Masahiko, Akimoto, Keiko, Iida, Hiroshi, Endo, Hiroki, Hosono, Kunihiro, Ikeda, Tamon, Sakamoto, Yasunari, Fujita, Koji, Yoneda, Masato, Koide, Tomoko, Takahashi, Hirokazu, Tokoro, Chikako, Goto, Ayumu, Abe, Yasunobu, Kobayashi, Noritoshi, Kubota, Kensuke, Saito, Satoru, Moriya, Akihiko, Rino, Yasushi, Imada, Toshio, and Nakajima, Atsushi Gastric surgery is not a risk factor for erosive esophagitis or Barrett's esophagus. Scandinavian Journal of Gastroenterology 2010; 45 (4): 403-408.
22. Abdul-Razzak, Khalid K. and Bani-Hani, Kamal E. Increased prevalence of Helicobacter pylori infection in gastric cardia of patients with reflux esophagitis: a study from Jordan. Journal of Digestive Diseases 2007; 8 (4): 203-206.
23. Nason, Katie S., Farrow, Diana C., Haigh, Geoffrey, Lee, Sum P., Bronner, Mary P., Rosen, Sheldon N., and Vaughan, Thomas L. Gastric fluid bile concentrations and risk of Barrett's esophagus. Interactive cardiovascular and thoracic surgery 2007; 6 (3): 304-307.
24. Miwa, H., Minoo, T., Hojo, M., Yaginuma, R., Nagahara, A., Kawabe, M., Ohkawa, A., Asaoka, D., Kurosawa, A., Ohkusa, T., and Sato, N. Oesophageal hypersensitivity in Japanese patients with non-erosive gastro-oesophageal reflux diseases. Alimentary pharmacology & therapeutics 2004; 20 Suppl 1: 112-117.
25. Mitre, Marcia C., Katzka, David A., Brensinger, Colleen M., Lewis, James D., Mitre, Ricardo J., and Ginsberg, Gregory G. Schatzki ring and Barrett's esophagus: do they occur together? Digestive Diseases and Sciences 2004; 49 (5): 770-773.
26. Avidan, Benjamin, Sonnenberg, Amnon, Schnell, Thomas G., and Sontag, Stephen J. Hiatal hernia and acid reflux frequency predict presence and length of Barrett's esophagus. Digestive Diseases and Sciences 2002; 47 (2): 256-264.
27. Vaezi, M. F., Falk, G. W., Peek, R. M., Vicari, J. J., Goldblum, J. R., Perez-Perez, G. I., Rice, T. W., Blaser, M. J., and Richter, J. E. CagA-positive strains of Helicobacter pylori may protect against Barrett's esophagus. The American journal of gastroenterology 2000; 95 (9): 2206-2211.
28. Lord, R. V., Frommer, D. J., Inder, S., Tran, D., and Ward, R. L. Prevalence of Helicobacter pylori infection in 160 patients with Barrett's oesophagus or Barrett's adenocarcinoma. The Australian and New Zealand journal of surgery 2000; 70 (1): 26-33.
29. Hackelsberger, A., Schultze, V., Gunther, T., von Arnim, U., Manes, G., and Malfertheiner, P. The prevalence of Helicobacter pylori gastritis in patients with reflux oesophagitis: a case-control study. European journal of gastroenterology & hepatology 1998; 10 (6): 465-468.
30. Eisen, G. M., Sandler, R. S., Murray, S., and Gottfried, M. The relationship between gastroesophageal reflux disease and its complications with Barrett's esophagus. The American journal of gastroenterology 1997; 92 (1): 27-31.
31. Grande, L., Monforte, R., Ros, E., Toledo-Pimentel, V., Estruch, R., Lacima, G., Urbano-Marquez, A., and Pera, C. High amplitude contractions in the middle third of the oesophagus: a manometric marker of chronic alcoholism?. Gut 1996; 38 (5): 655-662.
32. Hilal, J., El-Serag, H. B., Ramsey, D., Ngyuen, T., and Kramer, J. R. Physical activity and the risk of Barrett's esophagus. Diseases of the Esophagus 2016; 29 (3): 248-254.
33. Krawczyk, M., Scierski, W., Ryszkiel, I., Namyslowski, G., Grzegorzek, S., and Misiolek, M. Endoscopic evidence of reflux disease in the larynx. Acta Oto-Laryngologica 2014; 134 (8): 831-837.
34. Thrift, A. P., Kramer, J. R., Qureshi, Z., Richardson, P. A., and El-Serag, H. B. Age at onset of GERD symptoms predicts risk of barrett's esophagus. American Journal of Gastroenterology 2013; 108 (6): 915-922.
35. Lv, J., Liu, D., Ma, S.-Y., and Zhang, J. Investigation of relationships among gastroesophageal reflux disease subtypes using narrow band imaging magnifying endoscopy. World journal of gastroenterology 2013; 19 (45): 8391-8397.
36. Sonnenberg, A. and Genta, R. M. Barrett's metaplasia and colonic neoplasms: A significant association in a 203,534-patient study. Digestive Diseases and Sciences 2013; 58 (7): 2046-2051.
37. Jiao, L., Kramer, J. R., Rugge, M., Parente, P., Verstovsek, G., Alsarraj, A., and El-Serag, H. B. Dietary intake of vegetables, folate, and antioxidants and the risk of Barrett's esophagus. Cancer Causes and Control 2013; 24 (5): 1005-1014.
38. Rosztoczy, A., Izbeki, F., Nemeth, I. B., Dulic, S., Vadaszi, K., Roka, R., Gecse, K., Gyokeres, T., Lazar, G., Tiszlavicz, L., and Wittmann, T. Detailed esophageal function and morphological analysis shows high prevalence of gastroesophageal reflux disease and Barrett's esophagus in patients with cervical inlet patch. Diseases of the Esophagus 2012; 25 (6): 498-504.
39. Mesteri, I., Lenglinger, J., Beller, L., Fischer-See, S., Schoppmann, S. F., Wrba, F., Riegler, F. M., and Zacherl, J. Assessment of columnar-lined esophagus in controls and patients with gastroesophageal reflux disease with and without proton-pump inhibitor therapy. European Surgery - Acta Chirurgica Austriaca 2012; 44 (5): 304-313.
40. Sistonen, S. J., Koivusalo, A., Nieminen, U., Lindahl, H., Lohi, J., Kero, M., Karkkainen, P. A., Farkkila, M. A., Sarna, S., Rintala, R. J., and Pakarinen, M. P. Esophageal morbidity and function in adults with repaired esophageal atresia with tracheoesophageal fistula: A population-based long-term follow-up. Annals of Surgery 2010; 251 (6): 1167-1173.
41. Ghanem, Y., Al-Rabeei, N. A., and Dallak, A. Gastro-oesophageal reflux disease among patients attending an endoscopic clinic in Yemen. Arab Journal of Gastroenterology 2009; 10 (3): 109-111.
42. Hahn, H. P., Blount, P. L., Ayub, K., Das, K. M., Souza, R., Spechler, S., and Odze, R. D. Intestinal differentiation in metaplastic, nongoblet columnar epithelium in the esophagus. American Journal of Surgical Pathology 2009; 33 (7): 1006-1015.
43. Smith, K. J., O'Brien, S. M., Green, A. C., Webb, P. M., and Whiteman, D. C. Current and Past Smoking Significantly Increase Risk for Barrett's Esophagus. Clinical Gastroenterology and Hepatology 2009; 7 (8): 840-848.
44. Lippmann, Q. K., Crockett, S. D., Dellon, E. S., and Shaheen, N. J. Quality of life in GERD and Barrett's esophagus is related to gender and manifestation of disease. American Journal of Gastroenterology 2009; 104 (11): 2695-2703.
45. Corley, D. A., Kubo, A., Levin, T. R., Habel, L., Zhao, W., Leighton, P., Rumore, G., Quesenberry, C., Buffler, P., and Block, G. Iron intake and body iron stores as risk factors for Barrett's esophagus: A community-based study. American Journal of Gastroenterology 2008; 103 (12): 2997-3004.
46. Ramus, J. R., Gatenby, P. A. C., Caygill, C. P. J., and Watson, A. Helicobacter pylori infection and severity of reflux-induced esophageal disease in a cohort of patients with columnar-lined esophagus. Digestive Diseases and Sciences 2007; 52 (10): 2821-2825.
47. Rajendra, S., Ackroyd, R., Robertson, I. K., Ho, J. J., Karim, N., and Kutty, K. M. Helicobacter pylori, ethnicity, and the gastroesophageal reflux disease spectrum: A study from the East. Helicobacter 2007; 12 (2): 177-183.
48. Corley, D. A., Kubo, A., Levin, T. R., Block, G., Habel, L., Zhao, W., Leighton, P., Quesenberry, C., Rumore, G. J., and Buffler, P. A. Abdominal Obesity and Body Mass Index as Risk Factors for Barrett's Esophagus. Gastroenterology 2007; 133 (1): 34-41.
49. Johansson, J., Hakansson, H.-O., Mellblom, L., Kempas, A., Johansson, K.-E., Granath, F., and Nyren, O. Risk factors for Barrett's oesophagus: A population-based approach. Scandinavian Journal of Gastroenterology 2007; 42 (2): 148-156.
50. Maieron, R., Elli, L., Marino, M., Floriani, I., Minerva, F., Avellini, C., Falconieri, G., Pizzolitto, S., and Zilli, M. Celiac disease and intestinal metaplasia of the esophagus (Barrett's esophagus). Digestive Diseases and Sciences 2005; 50 (1): 126-129.
51. Rajendra, S., Ackroyd, R., Murad, S., Mohan, C., Ho, J. J., Goh, K. L., Azrena, A., and Too, C. L. Human leucocyte antigen determinants of susceptibility to Barrett's oesophagus in Asians - A preliminary study. Alimentary Pharmacology and Therapeutics 2005; 21 (11): 1377-1383.
52. Felix, V. N. and Viebig, R. G. Simultaneous bilimetry and pHmetry in GERD and Barrett's patients. Hepato-gastroenterology 2005; 52 (65): 1452-1455.
53. Poelmans, J., Feenstra, L., Demedts, I., Rutgeerts, P., and Tack, J. The yield of upper gastrointestinal endoscopy in patients with suspected reflux-related chronic ear, nose, and throat symptoms. American Journal of Gastroenterology 2004; 99 (8): 1419-1426.
54. Chrysos, E., Prokopakis, G., Athanasakis, E., Pechlivanides, G., Tsiaoussis, J., Mantides, A., and Xynos, E. Factors affecting esophageal motility in gastroesophageal reflux disease. Archives of Surgery 2003; 138 (3): 241-246.
55. Romero, Y., Cameron, A. J., Schaid, D. J., McDonnell, S. K., Burgart, L. J., Hardtke, C. L., Murray, J. A., and Locke III, G. R. Barrett's esophagus: Prevalence in symptomatic relatives. American Journal of Gastroenterology 2002; 97 (5): 1127-1132.
56. Zaninotto, G., Portale, G., Parenti, A., Lanza, C., Costantini, M., Molena, D., Ruol, A., Battaglia, G., Costantino, M., Epifani, M., and Nicoletti, L. Role of acid and bile reflux in development of specialised intestinal metaplasia in distal oesophagus. Digestive and Liver Disease 2002; 34 (4): 251-257.
57. Zentilin, P., Conio, M., Mele, M. R., Mansi, C., Pandolfo, N., Dulbecco, P., Gambaro, C., Tessieri, L., Iiritano, E., Bilardi, C., Biagini, R., Vigneri, S., and Savarino, V. Comparison of the main oesophageal pathophysiological characteristics between short- and long-segment Barrett's oesophagus. Alimentary Pharmacology and Therapeutics 2002; 16 (5): 893-898.
58. Csendes, A., Smok, G., Burdiles, P., Quesada, F., Huertas, C., Rojas, J., and Korn, O. Prevalence of Barrett's esophagus by endoscopy and histologic studies: A prospective evaluation of 306 control subjects and 376 patients with symptoms of gastroesophageal reflux. Diseases of the Esophagus 2000; 13 (1): 5-11.
59. Incarbone, R., Bonavina, L., Szachnowicz, S., Saino, G., and Peracchia, A. Rising incidence of esophageal adenocarcinoma in Western countries: Is it possible to identify a population at risk? Diseases of the Esophagus 2000; 13 (4): 275-278.
60. Coenraad, M., Masclee, A. A. M., Straathof, J. W. A., Ganesh, S., Griffioen, G., and Lamers, C. B. H. W. Is Barrett's esophagus characterized by more pronounced acid reflux than severe esophagitis. American Journal of Gastroenterology 1998; 93 (7): 1068-1072.
61. Goldblum, J. R., Vicari, J. J., Falk, G. W., Rice, T. W., Peek, R. M., Easley, K., and Richter, J. E. Inflammation and intestinal metaplasia of the gastric cardia: The role of gastroesophageal reflux and H. pylori infection. Gastroenterology 1998; 114 (4 I): 633-639.
62. Champion, G., Richter, J. E., Vaezi, M. F., Singh, S., and Alexander, R. Duodenogastroesophageal reflux: Relationship to pH and importance in Barrett's esophagus. Gastroenterology 1994; 107 (3): 747-754.
63. Gotley, D. C., Morgan, A. P., Ball, D., Owen, R. W., and Cooper, M. J. Composition of gastro-oesophageal refluxate. Gut 1991; 32 (10): 1093-1099.
64. Pohl, Heiko, Wrobel, Katharina, Bojarski, Christian, Voderholzer, Winfried, Sonnenberg, Amnon, Rosch, Thomas, and Baumgart, Daniel C. Risk factors in the development of esophageal adenocarcinoma. The American journal of gastroenterology 2013; 108 (2): 200-207.
65. Takahashi, Yoshiko, Amano, Yuji, Yuki, Takafumi, Mishima, Yuko, Tamagawa, Yuji, Uno, Goichi, Ishimura, Norihisa, Sato, Shuichi, Ishihara, Shunji, and Kinoshita, Yoshikazu Impact of the composition of gastric reflux bile acids on Barrett's oesophagus. Digestive and liver disease 2011; 43 (9): 692-697.
66. Groome, Maximillian, Lindsay, Jamie, Ross, Peter E., Cotton, James P., Hupp, Ted R., and Dillon, John F. Use of oesophageal stress response proteins as potential biomarkers in the screening for Barrett's oesophagus. European journal of gastroenterology & hepatology 2008; 20 (10): 961-965.
67. Sgouros, Spiros N., Mpakos, Dimitrios, Rodias, Miltiadis, Vassiliades, Kostas, Karakoidas, Christos, Andrikopoulos, Evangelos, Stefanidis, Gerasimos, and Mantides, Apostolos Prevalence and axial length of hiatus hernia in patients, with nonerosive reflux disease: a prospective study. Journal of Clinical Gastroenterology 2007; 41 (9): 814-818.
68. Lurje, Georg, Vallbohmer, Daniel, Collet, Peter H., Xi, Huan, Baldus, Stephan E., Brabender, Jan, Metzger, Ralf, Heitmann, Michaela, Neiss, Susanne, Drebber, Ute, Holscher, Arnulf H., and Schneider, Paul M. COX-2 mRNA expression is significantly increased in acid-exposed compared to nonexposed squamous epithelium in gastroesophageal reflux disease. Journal of gastrointestinal surgery 2007; 11 (9): 1105-1111.
69. Wolfgarten, Eva, Putz, Benito, Holscher, Arnulf H., and Bollschweiler, Elfriede Duodeno-gastric-esophageal reflux--what is pathologic? Comparison of patients with Barrett's esophagus and age-matched volunteers. Journal of gastrointestinal surgery 2007; 11 (4): 479-486.
70. Pereira, A. D., Suspiro, A., Chaves, P., Saraiva, A., Gloria, L., de Almeida, J. C., Leitao, C. N., Soares, J., and Mira, F. C. Short segments of Barrett's epithelium and intestinal metaplasia in normal appearing oesophagogastric junctions: the same or two different entities? Gut 1998; 42 (5): 659-662.
71. Csendes, A., Maluenda, F., Braghetto, I., Csendes, P., Henriquez, A., and Quesada, M. S. Location of the lower oesophageal sphincter and the squamous columnar mucosal junction in 109 healthy controls and 778 patients with different degrees of endoscopic oesophagitis. Gut 1993; 34 (1): 21-27.
72. Winters, C. Jr, Spurling, T. J., Chobanian, S. J., Curtis, D. J., Esposito, R. L., Hacker, J. F., Johnson, D. A., Cruess, D. F., Cotelingam, J. D., and Gurney, M. S. Barrett's esophagus. A prevalent, occult complication of gastroesophageal reflux disease. Gastroenterology 1987; 92 (1): 118-124.
73. Vardar, R., Vardar, E., and Bor, S. Is the prevalence of intestinal metaplasia at the squamocolumnar junction different in patients with progressive sytemic sclerosis? Turkish Journal of Gastroenterology 2010; 21 (3): 251-256.
74. Moriichi, K., Watari, J., Das, K. M., Tanabe, H., Fujiya, M., Nata, T., Nomura, Y., Ueno, N., Ishikawa, C., Inaba, Y., Ito, T., Sato, R., Okamoto, K., Mizukami, Y., and Kohgo, Y. Effects of helicobacter pylori infection on aberrant CpG Island methylation status and cellular phenotype in columnar-lined esophagus in a Japanese population. Gastroenterology 2009; 136 (5 SUPPL. 1): A599-.
75. Cook, M. B., Drahos, J., Wood, S., Enewold, L., Parsons, R., Freedman, N. D., Taylor, P. R., Ricker, W., and Abnet, C. C. Pathogenesis and progression of oesophageal adenocarcinoma varies by prior diagnosis of Barrett's oesophagus. Br J Cancer 22-11-2016; 115 (11): 1383-1390.
76. Gatalica, Z., Chen, M., Snyder, C., Mittal, S., and Lynch, H. T. Barrett's esophagus in the patients with familial adenomatous polyposis. Fam Cancer 2014; 13 (2): 213-217.
77. Goldberg, A., Gerkin, R. D., and Young, M. Medical Prevention of Barrett's Esophagus: Effects of Statins, Aspirin, Non-aspirin NSAIDs, Calcium, and Multivitamins. Digestive diseases and sciences 2015; 60 (7): 2058-2062.
78. Conio, M., Filiberti, R., Blanchi, S., Ferraris, R., Marchi, S., Ravelli, P., Lapertosa, G., Iaquinto, G., Sablich, R., Gusmaroli, R., Aste, H., and Giacosa, A. Risk factors for Barrett's esophagus: a case-control study. International journal of cancer. Journal international du cancer 10-1-2002; 97 (2): 225-229.
79. Alicuben, Evan T., Tatum, James M., Bildzukewicz, Nikolai, Samakar, Kamran, Samaan, Jamil S., Silverstein, Einav N., Sandhu, Kulmeet, Houghton, Caitlin C., and Lipham, John C. Regression of intestinal metaplasia following magnetic sphincter augmentation device placement. Surgical endoscopy 2018.
80. Sonnenberg, A., Turner, K. O., and Genta, R. M. Decreased risk for microscopic colitis and inflammatory bowel disease among patients with reflux disease. Colorectal disease 2018. 20 (9) 813-820.
81. Bazin, Camille, Benezech, Alban, Alessandrini, Marine, Grimaud, Jean Charles, and Vitton, Veronique. Esophageal Motor Disorders Are a Strong and Independant Associated Factor of Barrett's Esophagus. Journal of neurogastroenterology and motility 2018. 24 (2) 216-225.
82. Vergouwe, Floor W. T., IJsselstijn, Hanneke, Biermann, Katharina, Erler, Nicole S., Wijnen, Rene M. H., Bruno, Marco J., and Spaander, Manon C. W. High Prevalence of Barrett's Esophagus and Esophageal Squamous Cell Carcinoma After Repair of Esophageal Atresia. Clinical gastroenterology and hepatology 2018. 16 (4) 513-521.
83. Triadafilopoulos, George, Tandon, Apurva, Shetler, Katerina P., and Clarke, John. Clinical and pH study characteristics in reflux patients with and without ineffective oesophageal motility (IEM). BMJ open gastroenterology 2016. 3 (1) e000126.
84. Schlottmann, Francisco, Andolfi, Ciro, Herbella, Fernando A., Rebecchi, Fabrizio, Allaix, Marco E., and Patti, Marco G. GERD: Presence and Size of Hiatal Hernia Influence Clinical Presentation, Esophageal Function, Reflux Profile, and Degree of Mucosal Injury. The American surgeon 2018. 84 (6) 978-982.
85. Itskoviz, David, Levi, Zohar, Boltin, Doron, Vilkin, Alex, Snir, Yifat, Gingold-Belfer, Rachel, Niv, Yaron, Dotan, Iris, and Dickman, Ram. Risk of Neoplastic Progression Among Patients with an Irregular Z Line on Long-Term Follow-Up. Digestive diseases and sciences 2018. 63 (6) 1513-1517.
86. Baik, D., Sheng, J., Schlaffer, K., Friedenberg, F. K., Smith, M. S., and Ehrlich, A. C. Abdominal diameter index is a stronger predictor of prevalent Barrett's esophagus than BMI or waist-to-hip ratio. Diseases of the esophagus 2017. 30 (9) 1-6.
87. Benjamin, Tanmayee, Zackria, Shamiq, Lopez, Rocio, Richter, Joel, and Thota, Prashanthi N. Upper esophageal sphincter abnormalities and high-resolution esophageal manometry findings in patients with laryngopharyngeal reflux. Scandinavian journal of gastroenterology 2017. 52 (8) 816-821.
88. Koivusalo, Antti I., Sistonen, Saara J., Lindahl, Harry G., Rintala, Risto J., and Pakarinen, Mikko P. Long-term outcomes of oesophageal atresia without or with proximal tracheooesophageal fistula - Gross types A and B. Journal of pediatric surgery 2017. 52 (10) 1571-1575.
89. Genco, Alfredo, Soricelli, Emanuele, Casella, Giovanni, Maselli, Roberta, Castagneto-Gissey, Lidia, Di Lorenzo, Nicola, and Basso, Nicola. Gastroesophageal reflux disease and Barrett's esophagus after laparoscopic sleeve gastrectomy: a possible, underestimated long-term complication. Surgery for obesity and related diseases 2017. 13 (4) 568-574.
90. Matsueda, K., Manabe, N., Toshikuni, N., Sato, Y., Watanabe, T., Yamamoto, H., and Haruma, K. Clinical characteristics and associated factors of Japanese patients with adenocarcinoma of the esophagogastric junction: a multicenter clinicoepidemiological study. Diseases of the esophagus 2017. 30 (6) 1-6.
91. Sharma, N., Hui, T., Wong, H. C., Srivastava, S., Teh, M., Yeoh, K. G., and Ho, K. Y. Risk stratifying the screening of Barrett's esophagus: An Asian perspective. JGH Open 2017. 1 (2) 68-73.
92. Rangan, V., Chan, W., Lo, W.-K., and Feldman, N. Postreflux swallow- induced peristaltic wave index and mean nocturnal baseline impedance are decreased in GERD patients with Barrett ' s esophagus. Neurogastroenterology and Motility 2018. 30 (Supplement 1).
93. Yan, J., Strong, A. T., Sharma, G., Gabbard, S., Thota, P., Rodriguez, J., and Kroh, M. Surgical management of gastroesophageal reflux disease in patients with systemic sclerosis. Surgical Endoscopy and Other Interventional Techniques 2018. 32 (9) 3855-3860.
94. Royston, C., Caygill, C., Charlett, A., and Bardhan, K. D. The evolution and outcome of surveillance of Barrett's oesophagus over four decades in a UK District General Hospital. European Journal of Gastroenterology and Hepatology 2016. 28 (12) 1365-1373.
95. Chang, C.-Y., Lee, L. J. H., Wang, J.-D., Lee, C.-T., Tai, C.-M., Tang, T.-Q., and Lin, J.-T. Health-related quality of life in patients with Barrett's esophagus. Health and Quality of Life Outcomes 2016. 14 (1) 158.
96. Teixeira, A. C., Herbella, F. A., Bonadiman, A., Farah, J. F., and Del Grande, J. C. Predictive factors for short gastric vessels division during laparoscopic total fundoplication. Revista do Colegio Brasileiro de Cirurgioes 2015. 42 (3) 154-158.

### Participants did not have chronic GERD (n=1)

1. Cammarota, G., Galli, J., Agostino, S., De, Corso E., Rigante, M., Cianci, R., Cesaro, P., Nista, E. C., Candelli, M., Gasbarrini, A., and Gasbarrini, G. Accuracy of laryngeal examination during upper gastrointestinal endoscopy for premalignancy screening: Prospective study in patients with and without reflux symptoms. Endoscopy 2006; 38 (4): 376-381.

### Participants had alarm symptoms of EAC or are diagnosed with other gastroesophageal conditions or pre-existing disease (n=4)

1. Sami SS, Dunagan KT, Johnson ML, Schleck CD, Shah ND, Zinsmeister AR, Wongkeesong LM, Wang KK, Katzka DA, Ragunath K, Iyer PG. A randomized comparative effectiveness trial of novel endoscopic techniques and approaches for Barrett's esophagus screening in the community. The American journal of gastroenterology. 2015 Jan;110(1):148.
2. Yang S, Wu S, Huang Y, Shao Y, Chen XY, Xian L, Zheng J, Wen Y, Chen X, Li H, Yang C. Screening for oesophageal cancer. Cochrane Database of Systematic Reviews 2012, Issue 12. Art. No.: CD007883. DOI: 10.1002/14651858.CD007883.pub2
3. Ragunath, K., Krasner, N., Raman, V. S., Haqqani, M. T., and Cheung, W. Y. A randomized, prospective cross-over trial comparing methylene blue-directed biopsy and conventional random biopsy for detecting intestinal metaplasia and dysplasia in Barrett's esophagus. Endoscopy 2003; 35 (12): 998-1003.
4. Sharriff MK, Varghese S, O’Donovan M, Abdullahi Z, Liu X, Fitzgeral RC, Di Pietro M. Pilot randomized crossover study comparing the efficacy of transnasal disposable endosheath with standard endoscopy to detect Barrett’s esophagus. *Endoscopy* 2016; 48:110-116.

### Companion paper with no relevant results (n=1)

1. Crews NR, Johnson ML, Schleck CD, Enders FT, Wongkeesong L-M, Wang KK, Katzka DA, Iyer PG. Prevalence and Predictors of Gastroesophageal Reflux Complications in Community Subjects. *Dig Dis Sci* 2016; 61:3221-3228.

### Includes adenocarcinoma of the esophagus or gastric gardia combined (n=1)

1. Kearney DJ, Crump C, Maynard C, Boyko EJ. A case-control study of endoscopy and mortality from adenocarcinoma of the esophagus or gastric cardia in persons with GERD. *Gastrointestinal Endoscopy* 2003; 57(7):823-829.

### Comparison based on timing (intervals) of endoscopy (n=1)

van Soest EM, Dieleman JP, Sturkenboom MCJM, Siersema PD, Kuipers EJ. Gastro-oesophageal reflux, medical resource utilization and upper gastrointestinal endoscopy in patients at risk of oesophageal adenocarcinoma. *Aliment Pharmacol Ther* 2008; 28:137-143.

### Evaluates prevalence of BE on repeat exam (n=1)

1. Rodriguez S, Mattek N, Lieberman D, Fennerty B, Eisen G. Barrett’s Esophagus on Repeat Endoscopy: Should We Look More Than Once? *Am J Gastroenterol* 2008; 103:1892-1897

##

## KQ2 List of excluded studies at full text

###

### Full text not available (n=9)

1. Craig A, Shoeman M, Dent J. A comparison of narrow bore transnasal and transoral endoscopy in usedated patients [abstract]. Gastrointest Endosc 1998;47:AB28.
2. Mulcahy HE, Alstead EM, McKenzie C, Riches A, Kiely M, Farthing MJG, Fairclough PD.A randomized trial of a 5.5 mm vs 9.5 mm diameter videogastroscope in unsedated upper GI endoscopy [abstract]. Gastrointest Endosc 1997;45:AB54
3. Mulcahy HE, Kelly P, Banks M, Farthing MJG, Fairclough PD, Kumar P. Factors associated with tolerance to unsedated upper gastrointestinal endoscopy [abstract]. Gastrointest Endosc 1998;47:AB56.
4. Lewis, Liane, Marcu, Afrodita, Whitaker, Katriina, and Maguire, Roma. Patient factors influencing symptom appraisal and subsequent adjustment to oesophageal cancer: A qualitative interview study. European journal of cancer care 2018; 27 (1).
5. Stasyshyn, Andriy. Diagnosis and treatment of gastroesophageal reflux disease complicated by Barrett's esophagus. Polski przeglad chirurgiczny 2017; 89 (4) 29-32.
6. Gehlot, V., Mahant, S., Das, K., and Das, R. Risk or lifestyle factors associated with Gastroesophageal Reflux Disease (GERD) in north India. Helicobacter 2016; 21 (Supplement 1) 164.
7. Sakin, Y. S., Vardar, R., Sezgin, B., Cetin, Z. E., Alev, Y., Yildirim, E., Kirazli, T., and Bor, S. The diagnostic value of 24-hour ambulatory intraesophageal PH-impedance in patients with laryngopharyngeal reflux symptoms compared to those with typical symptoms. United European Gastroenterology Journal 2016; 4 (5 Supplement 1) A684-A685.
8. Jovani, M., Cao, Y., Feskanich, D., Hur, C., Jacobson, B. C., and Chan, A. T. Aspirin use is associated with lower risk of Barrett's esophagus in women. Gastroenterology 2017; 152 (5 Supplement 1) S105.
9. Ward, M. A., Dunst, C. M., Robinson, B., Teitelbaum, E. N., Sharata, A. M., DeMeester, S. R., Reavis, K. M., and Swanstrom, L. L. 20 Year outcomes: Laparoscopic heller myotomy stands the test of time. Surgical Endoscopy and Other Interventional Techniques 2017; 31 (Supplement 1) S234.

### Other language (n=1)

1. Dohmen W, Fuchs W. Rapidity of pain relief, medication requirement and patient satisfaction with reflux treatment in the physician's office. *MMW-Fortschritte der Medizin* 2005; 147(9): 39. [German]

### Study design (i.e., commentary, opinion, editorial, review, abstract or protocol) (n=35)

1. Munoz-Largacha JA, Fernando HC, Litle VR. Optimizing the diagnosis and therapy of Barrett's esophagus. *Journal of Thoracic Disease* 2017; 9: S146-S153.
2. Parker CE, Spada C, Mcalindon M, Davison C, Panter S. Capsule endoscopy-not just for the small bowel: A review. *Expert Review of Gastroenterology and Hepatology* 2014; 9(1): 79-89.
3. Estores D, Velanovich V. Barrett esophagus: Epidemiology, pathogenesis, diagnosis, and management. *Current Problems in Surgery* 2013; 50(5): 192-226.
4. Farnbacher MJ, Keles M, Meier M, Hagel A, Schneider T. Capsule endoscopy in a network cooperation: Assessment of the experience in 822 patients. *Scandinavian Journal of Gastroenterology* 2013; 48(9): 1088-1094.
5. Shaheen N. Barrett esophagus: Disease management and patient perceptions. *Gastroenterology and Hepatology* 2006; 2(7): 468-470.
6. Barr H. Endoscopic surveillance of patients with Barrett's oesophagus. *Gut* 2002; 51(3): 313-314.
7. Kamolz T, Velanovich V. Psychological and emotional aspects of gastroesophageal reflux disease. *Diseases of the Esophagus* 2002; 15(3): 199-203.
8. Tierney M, Bevan R, Rees CJ, Trebble TM. What do patients want from their endoscopy experience? The importance of measuring and understanding patient attitudes to their care. *Frontline Gastroenterol* 2016; 7(3): 191-198.
9. Ofman JJ, Rabeneck L. The effectiveness of endoscopy in the management of dyspepsia: a qualitative systematic review. *American Journal of Medicine* 1999; 106(3): 335-346.
10. Hinojosa-Lindsey M, Arney J, Heberlig S, Kramer JR, Street RL Jr, El-Serag HB, Naik AD. Patients' intuitive judgments about surveillance endoscopy in Barrett's esophagus: a review and application to models of decision-making. *Dis Esophagus* 2013; 26(7): 682-689.
11. Sorbi D, Chak A. Unsedated EGD. *Gastrointest Endosc* 2003; 58:102-10
12. Atkinson M, Chak A. Unsedated small-caliber endoscopyda new screening and surveillance tool for Barrett’s esophagus. *Gastroenterology & Hepatology* 2017 Aug; 4(8):426-427.
13. Eisen GM, Baron TH, Dominitz JA, Faigel DO, Goldstein JL, Johanson JF, Mallery JS, Raddawi HM, Vargo JJ II, Waring JP, Fanelli RD, Wheeler-Harbough J. Complications of upper GI endoscopy. *Gastrointest Endosc* 2002; 55: 784-793.
14. Ross WA. Premedication for upper gastrointestinal endoscopy. *Gastrointest Endosc* 1989; 35(2):120-126.
15. Saeian K. Unsedated transnasal endoscopy: a safe and less costly alternative. *Curr Gastroenterol Rep* 2002; 4:213-7.
16. Waterman M, Gralnek IM. Capsule endoscopy of the esophagus. *J Clin Gastroenterol* 2009; 43:605-12.
17. Waye JD. Worldwide use of sedation and analgesia for upper intestinal endoscopy. *Gastrointest Endosc* 1999; 50:888-91.
18. Kramer JR, Arney J, Chen J, Richardson P, Duan Z, Street RLJ, Hinojosa-Lindsey M, Naik AD, El-Serag HB. Patient-centered, comparative effectiveness of esophageal cancer screening: protocol for a comparative effectiveness research study to inform guidelines for evidence-based approach to screening and surveillance endoscopy. *BMC Health Services Research* 2012; 12: 288-.
19. Tan G, Gandhi M. Outcomes of open access endoscopy in dyspepsia/GERD patients without alarm features in a community medical center. *American Journal of Gastroenterology* 2015; 110: S641-S642.
20. Nason K, Romero Y, Shaheen N, Vaughan T, Switzer G, Chang J, Zickmund S, Luketich J. Lack of symptom reporting and self-medication are prevalent and modifiable barriers to early diagnosis in esophageal cancer. *Diseases of the Esophagus* 2014; 27: 61A-
21. Crews NR, Dunagan KT, Johnson ML, Devanna S, Wong Kee Song LM, Katzka DA, Iyer PG. Prevalence and characteristics of esophagitis and barrett's esophagus in population subjects without gastroesophageal reflux symptoms: Results from a large randomized controlled study. *Gastroenterology* 2014; 146(5 SUPPL. 1): S28-S29.
22. Alashkar B, Faulx AL, Isenberg GA, Greer KB, Pulice R, Hepner A, Falck-Ytter Y, Chak A. Comparative acceptance of transnasal esophagoscopy vs. Esophageal capsule endoscopy for barrett's esophagus screening. *Gastroenterology* 2013; 144(5 SUPPL. 1): S689-S690.
23. Egginton J, Dunagan KT, Shah ND, Blevins C, Ragunathan K, Leggett CL, Iyer PG. Patient preferences for endoscopic assessment of gastroesophageal reflux and barrett's esophagus. *Gastroenterology* 2013; 144(5 SUPPL. 1): S689-.
24. Hinojosa-Lindsey M, El-Serag H, Arney J, Kramer JR, Street RL, Naik AD. Patients' and gastroenterologists' perspectives on adherence to surveillance endoscopy for barrett's esophagus. *Gastroenterology* 2012; 142(5 SUPPL. 1): S399-S400.
25. Dominitz JA, Seibel EJ. Mo1526 Tethered Capsule Endoscope (TCE) Versus Standard EGD for Screening for Barrett's Esophagus (BE): Preliminary Results From a Blinded Pilot Study. *Gastrointestinal Endoscopy* 2011; 73(4): AB375-.
26. Elfant AB, Scheider DM, Bourke MJ, Alhalel R, Peikin SR, Haber GB, et al. Prospective controlled trial of transnasal endoscopy (T-EGD) vs per-oral endoscopy (P-EGD) [abstract]. *Gastrointest Endosc* 1996;43: 311.
27. Jackson FW. Office endoscopy without sedation [Abstract]. *Gastrointest Endosc* 1981; 27(2):123.
28. Rey JF, Duforest D, Marek TA. The feasibility of upper GI endoscopy performed through the nose using thin videoendoscope [abstract]. *Gastrointest Endosc* 1996;43: 302.
29. Iqbal, Umair, Siddique, Osama, Ovalle, Anais, Anwar, Hafsa, and Moss, Steven F. Safety and efficacy of a minimally invasive cell sampling device ('Cytosponge') in the diagnosis of esophageal pathology: a systematic review. European journal of gastroenterology & hepatology 2018. 30 (11) 1261-1269.
30. Munoz-Largacha, Juan A., Fernando, Hiran C., and Litle, Virginia R. Optimizing the diagnosis and therapy of Barrett's esophagus. Journal of thoracic disease 2017. 9 (Suppl 2) S146-S153.
31. Britton, James, Keld, Richard, Prasad, Neeraj, Hamdy, Shaheen, McLaughlin, John, and Ang, Yeng. Effect of diagnosis, surveillance, and treatment of Barrett's oesophagus on health-related quality of life. The lancet. Gastroenterology & hepatology 2018. 3 (1) 57-65.
32. Wani, Sachin, Muthusamy, V. Raman, Shaheen, Nicholas J., Yadlapati, Rena, Wilson, Robert, Abrams, Julian A., Bergman, Jacques, Chak, Amitabh, Chang, Kenneth, Das, Ananya, Dumot, John, Edmundowicz, Steven A., Eisen, Glenn, Falk, Gary W., Fennerty, M. Brian, Gerson, Lauren, Ginsberg, Gregory G., Grande, David, Hall, Matt, Harnke, Ben, Inadomi, John, Jankowski, Janusz, Lightdale, Charles J., Makker, Jitin, Odze, Robert D., Pech, Oliver, Sampliner, Richard E., Spechler, Stuart, Triadafilopoulos, George, Wallace, Michael B., Wang, Kenneth, Waxman, Irving, and Komanduri, Srinadh. Development of quality indicators for endoscopic eradication therapies in Barrett's esophagus: the TREAT-BE (Treatment with Resection and Endoscopic Ablation Techniques for Barrett's Esophagus) Consortium. Gastrointestinal endoscopy 2017. 86 (1) 1-17.
33. Zakko, L., Visrodia, K., Leggett, C., Lutzke, L., and Wang, K. K. Screening patients for Barrett esophagus: Why, who, and how. Techniques in Gastrointestinal Endoscopy 2018. 20 (2) 55-61.
34. Schlottmann, F. and Patti, M. G. Current Concepts in Treatment of Barrett's Esophagus With and Without Dysplasia. Journal of gastrointestinal surgery 2017. 21 (8) 1354-1360.
35. Offman, J., Muldrew, B., O'Donovan, M., Debiram-Beecham, I., Pesola, F., Kaimi, I., Smith, S. G., Wilson, A., Khan, Z., Lao-Sirieix, P., Aigret, B., Walter, F. M., Rubin, G., Morris, S., Jackson, C., Sasieni, P., and Fitzgerald, R. C. Barrett's oESophagus trial 3 (BEST3): Study protocol for a randomised controlled trial comparing the Cytosponge-TFF3 test with usual care to facilitate the diagnosis of oesophageal pre-cancer in primary care patients with chronic acid reflux. BMC Cancer 2018. 18 (1) 784.
[truncated: 241,404 more chars]
