# Supplementary material for: Screening for esophageal adenocarcinoma and precancerous conditions (dysplasia and Barrett’s esophagus) in patients with chronic gastroesophageal reflux disease with or without other risk factors: two systematic reviews and one overview of reviews to inform a guideline of the Canadian Task Force on Preventive Health Care (CTFPHC)
Source: Syst Rev. 2020 Jan 29;9:20. doi: 10.1186/s13643-020-1275-2 (PMC6990541; doi:10.1186/s13643-020-1275-2)
Supplement: Supplementary file 9 — Additional file 9: Data extraction variables. [file 13643_2020_1275_MOESM9_ESM.docx]

# Additional file 9. Data extraction variables

## KQ1

- Author
- Year published
- Country of conduct
- Funding
- Study design
- Sample size (overall and per group)
- Timing of data collection
- Follow-up
- GERD definition
- Intervention
- Comparator
- Setting
- GERD definition
- Inclusion and exclusion criteria
- Patient characteristics: age, sex, ethnicity, PPI therapy, smokers, BMI
- Outcomes
- Results

## KQ2

- Author
- Year published
- Country of conduct
- Funding
- Study design
- Sample size (overall and per group)
- Timing of data collection
- Follow-up
- GERD definition
- Intervention
- Comparator
- Setting
- GERD definition
- Inclusion and exclusion criteria
- Patient characteristics
- Outcomes
- Results

## KQ3

- Systematic review information
  - Author
  - Year published
  - Country of conduct
  - Funding
  - Conflict of interest
  - Date of last search and databases searched
  - List of included studies
  - Total population of SR
  - Primary studies included
  - Comparisons and number of trials
  - Outcomes
- Primary studies
  - Author
  - Year published
  - Country of conduct
  - Intervention and comparator including the number of participants in each group
  - Total number of participants and level of disease (e.g., low grade dysplasia)
  - Participant characteristics: sex, age, race, and if they had other gastroesophageal conditions
