# Supplementary material for: Screening for esophageal adenocarcinoma and precancerous conditions (dysplasia and Barrett’s esophagus) in patients with chronic gastroesophageal reflux disease with or without other risk factors: two systematic reviews and one overview of reviews to inform a guideline of the Canadian Task Force on Preventive Health Care (CTFPHC)
Source: Syst Rev. 2020 Jan 29;9:20. doi: 10.1186/s13643-020-1275-2 (PMC6990541; doi:10.1186/s13643-020-1275-2)
Supplement: Supplementary file 10 — Additional file 10: Additional details of RoB and Quality Assessment Methods. [file 13643_2020_1275_MOESM10_ESM.docx]

# Additional file 10. Additional details of RoB and Quality Assessment Methods

## KQ1

For the RCTs identified in KQ1, as stated in the Cochrane Handbook (Higgins & Green, 2008), outcome-specific domains (i.e., blinding of participants/personnel, blinding of outcome assessors, and incomplete outcome data) were assessed at the outcome level. The overall RoB for the body of evidence involved a judgement of the relative importance of domains, guided by known empirical evidence of bias, the likely direction of bias, and the likely magnitude of bias (Higgins & Green, 2008), and all outcomes (or outcome classes) were given a summary judgement within each trial. These assessments were considered in the Grading of Recommendations Assessment, Development and Evaluation (GRADE) domain of study limitations.

## KQ2

For KQ 2, the remaining evidence that was located addressed the ‘willingness to participate/be screened’ outcome, which would have been collected only during the consent period of the studies. This, therefore, would have related to all potentially eligible participants in the study, but not determined separately for the groups being compared. In these cases, a formal RoB assessment was not performed, as relevant tools would apply to study conduct as of allocation onward.

## KQ3

We used the AMSTAR 2 (Shea et al., 2017) approach to come up with final assessments of quality of conduct, including consideration of four critical domains (i.e., 1. Was an a priori design provided? 2. Was a comprehensive literature search performed? 3. Was a list of studies provided? 4. Was the likelihood of publication bias assessed? A senior reviewer categorized the quality as high, moderate, low, or critically low, using the criteria below, with another senior reviewer verifying these categorizations: (i) High quality: ≤1 non-critical weakness; (ii) Moderate quality: >1 non-critical weakness and no critical flaw; (iii) Low: one critical flaw; or (iv) Critically low: >1 critical flaws.

# References

Higgins, J., & Green, S. (2008). *Cochrane handbook for systematic reviews of interventions*. Wiley Online Library.

Shea, B. J., Reeves, B. C., Wells, G., Thuku, M., Hamel, C., Moran, J., … Kristjansson, E. (2017). AMSTAR 2: A critical appraisal tool for systematic reviews that include randomised or non-randomised studies of healthcare interventions, or both. *Bmj*, *358*, j4008.
