# Supplementary material for: Screening for esophageal adenocarcinoma and precancerous conditions (dysplasia and Barrett’s esophagus) in patients with chronic gastroesophageal reflux disease with or without other risk factors: two systematic reviews and one overview of reviews to inform a guideline of the Canadian Task Force on Preventive Health Care (CTFPHC)
Source: Syst Rev. 2020 Jan 29;9:20. doi: 10.1186/s13643-020-1275-2 (PMC6990541; doi:10.1186/s13643-020-1275-2)
Supplement: Supplementary file 11 — Additional file 11: KQ1 evidence sets. [file 13643_2020_1275_MOESM11_ESM.docx]

**Additional file 11. KQ1 Evidence Sets**

Contents

[Table 1. Evidence Set comparisons, outcomes and studies 1](#_Toc19792263)

[Evidence Set 1: Esophagogastroduodenoscopy (EGD) versus no prior EGD 2](#_Toc19792264)

[*Evidence Set 1 - Results table* 2](#_Toc19792265)

[*Evidence Set 1 - Forest Plots* 3](#_Toc19792266)

[*Evidence Set 1 - GRADE evidence profile table* 4](#_Toc19792267)

[*Evidence Set 1 - Summary of Findings Table* 5](#_Toc19792268)

[Evidence Set 2: Esophagogastroduodenoscopy (EGD) versus Transnasal esophagoscopy (TNE) 6](#_Toc19792269)

[*Evidence Set 2 - Results table* 6](#_Toc19792270)

[*Evidence Set 2 - Forest Plots* 8](#_Toc19792271)

[*Evidence Set 2 - GRADE evidence profile table* 9](#_Toc19792272)

[*Evidence Set 2 - Summary of Findings Table* 12](#_Toc19792273)

[Evidence Set 3: Esophagogastroduodenoscopy (EGD) versus Video capsule esophagoscopy (VCE) 14](#_Toc19792274)

[*Evidence Set 3 - Results table* 14](#_Toc19792275)

[*Evidence Set 3 - Forest Plot* 15](#_Toc19792276)

[*Evidence Set 3 - GRADE evidence profile table* 16](#_Toc19792277)

[*Evidence Set 3 - Summary of Findings Table* 17](#_Toc19792278)

[Evidence Set 4: Esophagogastroduodenoscopy (EGD) versus Transoral-EGD 18](#_Toc19792279)

[*Evidence Set 4 - Results table* 18](#_Toc19792280)

[*Evidence Set 4 - Forest Plot* 19](#_Toc19792281)

[*Evidence Set 4 - GRADE evidence profile table* 20](#_Toc19792282)

[*Evidence Set 4 - Summary of Findings Table* 21](#_Toc19792283)

[Evidence Set 5: Transnasal esophagoscopy (TNE) versus Video capsule esophagoscopy (VCE) 22](#_Toc19792284)

[*Evidence Set 5 - Results table* 22](#_Toc19792285)

[*Evidence Set 5 - Forest Plots* 23](#_Toc19792286)

[*Evidence Set 5 - GRADE evidence profile table* 24](#_Toc19792287)

[*Evidence Set 5 - Summary of Findings Table* 26](#_Toc19792288)

[Evidence Set 6: Transnasal esophagoscopy (TNE) versus Transoral EGD 28](#_Toc19792289)

[*Evidence Set 6 - Results table* 28](#_Toc19792290)

[*Evidence Set 6 - Forest Plots* 29](#_Toc19792291)

[*Evidence Set 6 - GRADE evidence profile table* 30](#_Toc19792292)

[*Evidence Set 6 - Summary of Findings table* 32](#_Toc19792293)

[Evidence Set 7: EGD with random biopsy versus Enhanced magnification-directed endoscopy (EME) biopsies 33](#_Toc19792294)

[*Evidence Set 7 - Results table* 33](#_Toc19792295)

[*Evidence Set 7 - Forest Plots* 34](#_Toc19792296)

[*Evidence Set 7 - GRADE evidence profile table* 35](#_Toc19792297)

[*Evidence Set 7 - Summary of Findings Table* 36](#_Toc19792298)

[Evidence Set 8: EGD with random biopsy versus chromoendoscopy 37](#_Toc19792299)

[*Evidence Set 8 - Results table* 37](#_Toc19792300)

[*Evidence Set 8 - Forest Plot* 38](#_Toc19792301)

[*Evidence Set 8 - GRADE evidence profile table* 39](#_Toc19792302)

[*Evidence Set 8 - Summary of Findings Table* 40](#_Toc19792303)

**Table 1. Evidence Set comparisons, outcomes and studies**

The table below presents the outcomes of interest, the comparisons in the included studies, and which studies report on these comparisons and outcomes. If the cell is blank, no study reported this outcome for that comparator, and there are no results presented in the results tables or any results in the GRADE tables.

|  | **Evidence Set 1** | **Evidence Set 2** | **Evidence Set 3** | **Evidence Set 4** | **Evidence Set 5** | **Evidence Set 6** | **Evidence Set 7** | **Evidence Set 8** |
| --- | --- | --- | --- | --- | --- | --- | --- | --- |
|  | **EGD vs no prior EGD** | **EGD vs TNE** | **EGD vs VCE** | **EGD vs TNE** | **TNE vs VCE** | **TNE vs TNE** | **Random biopsy vs EM-DE** | **Random biopsy vs chromoendoscopy** |
| Mortality |  |  |  |  |  |  |  |  |
| Survival | Rubenstein 2008 |  |  |  |  |  |  |  |
| Serious adverse events* |  | Sami 2015† |  |  |  | Zaman 1999 |  |  |
| Esophageal adenocarcinoma | Rubenstein 2008  Hammad 2019 | Jobe 2006 |  |  |  |  |  |  |
| Suspected Barrett’s Esophagus |  | Chang 2011;  Sami 2015†;  Mori 2010 | Chang 2011 | Mori 2010 | Chak 2014;  Chang 2011 | Zaman 1999  Mori 2010 |  |  |
| Confirmed Barrett’s Esophagus |  | Sami 2015†;  Jobe 2006 | Chang 2011 |  | Chak 2014;  Chang 2011 |  | Ferguson 2006 | Wani 2014 |
| Dysplasia |  | Chang 2011;  Jobe 2006 | Chang 2011 |  | Chang 2011 |  |  |  |
| QoL |  |  |  |  |  |  |  |  |
| Psychological effects |  | Chang 2011;  Sami 2015†;  Jobe 2006 |  |  | Chak 2014 | Zaman 1999 |  |  |
| Medical procedures |  |  |  |  |  |  |  |  |
| Overdiagnosis |  |  |  |  |  |  |  |  |

EGD: esophagogastroduodenoscopy; EM-DE: Enhanced magnification-directed endoscopy; TNE: Transnasal esophagoscopy; VCE: Video capsule endoscopy

* Life threatening, severe or medically significant consequences

† Evaluates transnasal esophagoscopy in the hospital and in a mobile van.

**Evidence Set 1: Esophagogastroduodenoscopy (EGD) versus no prior EGD**

***Evidence Set 1 - Results table***

| **Author Year**  **Study design** | **Outcome(s) description** | **Results** | | **ROB assessment** | **Notes** |
| --- | --- | --- | --- | --- | --- |
|  |  | **EGD** | **no prior EGD** |  |  |
| **CRITICAL OUTCOME 2: Long-term survival** | | | | | |
| Rubenstein 2008^49^  Retrospective cohort | Long-term survival^^[[1]](#footnote-1)^^:  1 year after diagnosis of cancer  5 years after diagnosis of cancer  10 years after diagnosis of cancer | 51.6% | 50.2% | Moderate | Study authors report that there was no difference in long-term survival between those who had received a prior EGD and those who had not (HR 0.82 [95%CI 0.52-1.29]). Adjusting for age, comorbidities, and year of diagnosis yielded similar results (HR 0.93 [95% CI, 0.58-1.50]). |
|  |  | 21.9% | 10.3% |  |  |
|  |  | 6.1% | 6.1% |  |  |
| **IMPORTANT OUTCOME 4: Incidence of EAC (by stage), BE, and low- and high-grade dysplasia** | | | | | |
| **EAC** | | | | | |
| Rubenstein 2008^49^  Retrospective cohort | EAC stage at diagnosis^^[[2]](#footnote-2)^^: |  | | Moderate | It was difficult to determine a range of effects across studies due to very low sample size in the EGD group of the Hammad 2019 study.  Of the 155 participants with EAC, 25 had previous EGD in the past 1-5 years, and 130 had not (Rubenstein).  Among the 153 EAC patients with no previously known BE, 1 had a previous EGD in the last 5 years and 152 had not (Hammad). |
|  | Stage 1 | 7/25 (28%) | 16/130 (12%) |  |  |
|  | Stage 2 | 11/25 (44%) | 47/130 (36%) |  |  |
|  | Stage 3 | 3/25 (12%) | 31/130 (24%) |  |  |
|  | Stage 4 | 4/25 (16%) | 36/130 (28%) |  |  |
| Hammad 2019^50^  Retrospective cohort | EAC stage at diagnosis: |  | | Moderate |  |
|  | Stage 0 | 0/1 (0%) | 2/152 (1%) |  |  |
|  | Stage 1 | 0/1 (0%) | 8/152 (5%) |  |  |
|  | Stage 2 | 0/1 (0%) | 37/152 (24%) |  |  |
|  | Stage 3 | 0/1 (0%) | 31/152 (20%) |  |  |
|  | Stage 4 | 0/1 (0%) | 70/152 (46%) |  |  |
|  | Unknown | 1/1 (100%) | 4/152 (3%) |  |  |

***Evidence Set 1 - Forest Plots***

*Forest Plot 1.1: EAC Stage 1 at diagnosis*


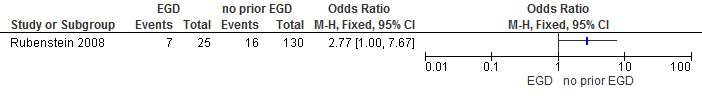


***Evidence Set 1 - GRADE evidence profile table***

| **Certainty assessment** | | | | | | | **№ of patients** | | | **Effect** | | | **Certainty** | **Importance** | |
| --- | --- | --- | --- | --- | --- | --- | --- | --- | --- | --- | --- | --- | --- | --- | --- |
| **№ of studies** | **Study design** | **Risk of bias** | **Inconsistency** | **Indirectness** | **Imprecision** | **Other considerations** | **EGD** | **no EGD** | | **Relative (95% CI)** | | **Absolute (95% CI)** |  |  |  |
| Survival | | | | | | | | | | | | | | | |
| 1 | observational studies | serious ^a^ | not serious | serious ^b^ | serious ^c^ | none | Study authors report that there was no difference in long-term survival between those who had received a prior EGD and those who had not (HR 0.82 [95%CI 0.52-1.29]). Adjusting for age, comorbidities, and year of diagnosis yielded similar results (HR 0.93 [95% CI, 0.58-1.50]). | | | | | | ⨁◯◯◯ VERY LOW | CRITICAL | |
| EAC stage 1 at diagnosis | | | | | | | | | | | | | | | |
| 1 | observational studies | serious ^a^ | not serious | serious ^b^ | serious ^c^ | none | 7/25 (28.0%) | | 16/130 (12.3%) | | **RR 2.27** (1.04 to 4.95) | **156 more per 1,000** (from 5 more to 486 more) | ⨁◯◯◯ VERY LOW | | IMPORTANT |
| EAC stage at diagnosis | | | | | | | | | | | | | | | |
| 1 | observational studies | serious ^d^ | not serious | serious ^e^ | serious ^c^ | none | One out of 153 patients, not under surveillance for BE, had received an EGD in the previous five years. An additional 15 had received an EGD more than five years ago, with no additional details on timing. For the purposes of this review, these patients were grouped with those with no prior EGD. This one patient was diagnosed with "unknown stage" of EAC. | | | | | | ⨁◯◯◯ VERY LOW | | IMPORTANT |

**CI:** Confidence interval; **EGD**: Esophagogastroduodenoscopy; **RR:** Risk ratio

**Explanations**

a. The study consisted of a group of veterans, and there was a significant difference between groups on comorbidities.

b. GER identified by ICDs codes 530.10-530.12, 530.81, or 787.1

c. Too few participants.

d. This study consists of patients diagnosed with EAC at the VA Medical Centre. The authors do not provide a comparison for the participants of interest for this review, as their larger population included 29 patients undergoing surveillance for BE. These participants were excluded from our results. This left one patient not under surveillance for BE who received an EGD in the previous five years.

e. GERD was not defined and only two-thirds of the participants included in this review had GERD diagnosis.

***Evidence Set 1 - Summary of Findings Table***

| **EGD compared to no prior EGD for screening for EAC and precancerous conditions (BE and dysplasia)** | | | | | | |
| --- | --- | --- | --- | --- | --- | --- |
| **Setting**: Hospital-based  **Intervention**: EGD  **Comparison**: no prior EGD | | | | | | |
| Outcomes | **Anticipated absolute effects^*^** (95% CI) | | Relative effect (95% CI) | № of participants  (studies) | Certainty of the evidence (GRADE) | Comments |
|  | **Risk with no prior EGD** | **Risk with EGD** |  |  |  |  |
| Survival | Study authors report that there was no difference in long-term survival between those who had received a prior EGD and those who had not (HR 0.82 [95%CI 0.52-1.29]). Adjusting for age, comorbidities, and year of diagnosis yielded similar results (HR 0.93 [95% CI, 0.58-1.50]). | |  | (1 observational study) | ⨁◯◯◯ VERY LOW ^a,b,c^ |  |
| EAC stage 1 at diagnosis | 123 per 1,000 | **279 per 1,000** (128 to 609) | **RR 2.27** (1.04 to 4.95) | 155 (1 observational study) | ⨁◯◯◯ VERY LOW ^a,b,c^ |  |
| EAC stage at diagnosis | One out of 153 patients, not under surveillance for BE, had received an EGD in the previous five years. An additional 15 had received an EGD more than five years ago, with no additional details on timing. For the purposes of this review, these patients were grouped with those with no prior EGD. This one patient was diagnosed with "unknown stage" of EAC. | |  | 153  (1 observational study) | ⨁◯◯◯ VERY LOW ^c,d,e^ |  |
| ***The risk in the intervention group** (and its 95% confidence interval) is based on the assumed risk in the comparison group and the **relative effect** of the intervention (and its 95% CI).   **CI:** Confidence interval; **EGD**: Esophagogastroduodenoscopy **RR:** Risk ratio | | | | | | |
| **GRADE Working Group grades of evidence** **High certainty:** We are very confident that the true effect lies close to that of the estimate of the effect **Moderate certainty:** We are moderately confident in the effect estimate: The true effect is likely to be close to the estimate of the effect, but there is a possibility that it is substantially different **Low certainty:** Our confidence in the effect estimate is limited: The true effect may be substantially different from the estimate of the effect **Very low certainty:** We have very little confidence in the effect estimate: The true effect is likely to be substantially different from the estimate of effect | | | | | | |

**Explanations**

a. The study consisted of a group of veterans, and there was a significant difference between groups on comorbidities.

b. GER identified by ICDs codes 530.10-530.12, 530.81, or 787.1

c. Too few participants.

d. This study consists of patients diagnosed with EAC at the VA Medical Centre. The authors do not provide a comparison for the participants of interest for this review, as their larger population included 29 patients undergoing surveillance for BE. These participants were excluded from our results. This left one patient not under surveillance for BE who received an EGD in the previous five years.

e. GERD was not defined and only two-thirds of the participants included in this review had GERD diagnosis.

**Evidence Set 2: Esophagogastroduodenoscopy (EGD) versus Transnasal esophagoscopy (TNE)**

***Evidence Set 2 - Results table***

| **Author Year**  **Study design** | **Outcome(s) description** | | **Results** | | **ROB assessment** | **Notes** |
| --- | --- | --- | --- | --- | --- | --- |
|  |  |  | **EGD** | **TNE** |  |  |
| **CRITICAL OUTCOME 3: Life threatening, severe, or medically significant consequences** | | | | | | |
| Sami 2015^52^  RCT | Serious adverse events (e.g., bleeding, perforation, hospitalization) | | 0/61 | hospital TNE 0/72  mobile TNE 0/76 | High | Assessed 1 and 30 days after the procedure. |
| **IMPORTANT OUTCOME 4: Incidence of EAC (by stage), BE, and low- and high-grade dysplasia** | | | | | | |
| EAC | | | | | | |
| Jobe 2006^53^ Randomized crossover | EAC | | 0/43 | 0/54 | Moderate | Excludes those undergoing surveillance endoscopy (analysis). |
| **Suspected BE (endoscopically)** | | | | | | |
| Chang 2011^51^  RCT | Classified as endoscopic presence of 1 cm or more of columnar-lined distal esophagus above the gastroesophageal junction (z-line appearance (ZAP) classification). | | 2/20 (10%) | 1/19 (5%) | High |  |
| Sami 2015^52^  RCT | Defined as the presence of columnar mucosa at least 1 cm length in the tubular esophagus (Prague Classification) | | There was no difference in procedure yield between the study arms with regards to suspected (p=0.37) BE. | | High |  |
| Mori 2010^54^  Cohort study | Measurement of the mucosa between the esophagogastric junction and squamocolumnar junction. | Grade 1^^[[3]](#footnote-3)^^  Grade 2^^[[4]](#footnote-4)^^  Grade 3^^[[5]](#footnote-5)^^ | 61/254 (24%) | 188/727 (25.9%) | High | Patients were asked to select between screening options. There was no significant difference in the frequency distribution of the severity of BE. |
|  |  |  | 26/254 (10.2%) | 31/727 (4.3%) |  |  |
|  |  |  | 1/254 (0.39%) | 6/727 (0.8%) |  |  |
| **Confirmed BE (histologically)** | | | | | | |
| Sami 2015^52^  RCT | Defined as the presence of intestinal metaplasia with goblet cells in biopsies. | | There was no difference in procedure yield between the study arms with regards to confirmed (p=0.44) BE. | | High | Participants in the TNE groups were offered “confirmatory” EGD with histology in 2 weeks’ time. |
| Jobe 2006^53^ Randomized crossover | Intestinal metaplasia required the unequivocal presence of goblet cells within columnar epithelium. | | 32/121 (26%) | 36/121 (30%) | Moderate | Investigators performing TNE were unaware of whether the subject was undergoing a screening or surveillance procedure (28% (37/134)). |
|  |  |  | p-value=0.503 | |  |  |
| **Dysplasia** | | | | | | |
| Chang 2011^51^ RCT | Dysplasia (not defined) | | 0/20 | 0/20 | Moderate |  |
| Jobe 2006^53^  Randomized crossover | Dysplasia  Low-grade  High-grade | | 4 /52 | 4/64 | Moderate | Each modality detected 4 cases of low-grade dysplasia with concordance on only one case. |
|  |  |  | 1/52 | 0/64 |  |  |
| **IMPORTANT OUTCOME 6: Psychological effects** | | | | | | |
| Chang 2011^51^  RCT | Anxiety during the procedure (median score (range)) | | Not reported | 1.5 (0-7) | High | Study only gave those in the TNE group the tolerability questionnaire. |
| Sami 2015^52^  RCT | Anxiety during the procedure (Mean (SD)) (scale of 0-10, where 10 is severe) | | 0.8 (1.5) | hospital TNE 2.3 (2.2)  mobile TNE 2.8 (2.8) | High | p<0.001 between modalities |
| Jobe 2006^53^  Randomized crossover | Anxiety during procedure  None  Mild  Moderate  Severe | | 87/116  11/116  11/116  7/116 | 62/116  38/116  12/116  4/116 | High | The largest differences in comfort level between procedure type occurred primarily within the “none” to “mild” range; the “moderate” to “severe” categories were statistically similar between endoscopic approaches in all questions. |
|  |  |  | p=0.0001 | |  |  |
| Jobe 2006^53^  Randomized crossover | Anxiety before procedure  None  Mild  Moderate  Severe  Anxiety during insertion  None  Mild  Moderate  Severe | | 64/116  26/116  16/116  10/116 | 59/116  39/116  13/116  5/116 |  |  |
|  |  |  | p=0.084 | |  |  |
|  |  |  | 83/116  15/116  7/116  11/116 | 45/116  43/116  19/116  9/116 |  |  |
|  |  |  | p=0.0001 | |  |  |

***Evidence Set 2 - Forest Plots***

*Forest Plot 2.1: Incidence of endoscopically suspected BE (Grade 2 and 3) [observational]*


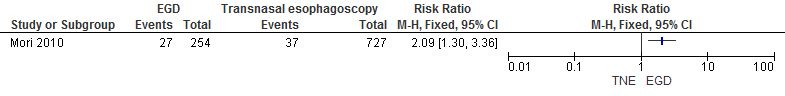


Includes those with Barrett’s Esophagus grade 2 and 3

*Forest Plot 2.2: Incidence of dysplasia*


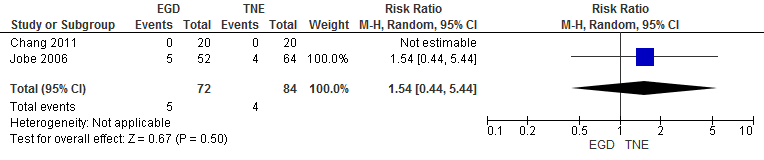


***Evidence Set 2 - GRADE evidence profile table***

| **Certainty assessment** | | | | | | | **№ of patients** | | **Effect** | | **Certainty** | **Importance** |
| --- | --- | --- | --- | --- | --- | --- | --- | --- | --- | --- | --- | --- |
| **№ of studies** | **Study design** | **Risk of bias** | **Inconsistency** | **Indirectness** | **Imprecision** | **Other considerations** | **EGD** | **TNE** | **Relative (95% CI)** | **Absolute (95% CI)** |  |  |
| Life threatening, severe, or medically significant consequences | | | | | | | | | | | | |
| 1 ^a^ | randomized trials | very serious ^b^ | not serious | serious ^c^ | serious ^d^ | none | Serious adverse events were assessed 1 and 30 days after the procedure. No serious adverse events were reported in any of the study arms. Hospital-based TNE and mobile-based TNE were combined for this outcome under TNE. | | | | ⨁◯◯◯ VERY LOW | CRITICAL |
| Incidence of EAC | | | | | | | | | | | | |
| 1 ^e^ | randomized trials | serious ^f^ | not serious | serious ^g^ | serious ^d^ | none | Among the participants who were receiving their initial screening (i.e., not those undergoing surveillance endoscopy), 0/43 and 0/54 were found to have EAC when randomized to be screened first with EGD or TNE, respectively. | | | | ⨁◯◯◯ VERY LOW | IMPORTANT |
| Incidence of suspected BE | | | | | | | | | | | | |
| 2 | randomized trials | very serious ^h^ | not serious | serious ^i^ | serious ^d^ | none | Sami 2015 reported that there was no difference in procedure yield between study arms with regards to suspected BE (p=0.37) [this considers all three study arms]. Chang 2011 reported 2/20 participants in the EGD and 1/19 participants in the TNE group having suspected BE. | | | | ⨁◯◯◯ VERY LOW | IMPORTANT |
| Incidence of suspected BE | | | | | | | | | | | | |
| 1 | observational studies | serious ^j^ | not serious | serious ^k^ | serious ^d^ | none | 27/254 (10.6%) | 37/727 (5.1%) | **RR 2.09** (1.30 to 3.36) | **55 more per 1,000** (from 15 fewer to 120 more) | ⨁◯◯◯ VERY LOW | IMPORTANT |
| Incidence of confirmed BE | | | | | | | | | | | | |
| 2 ^e^ | randomized trials | serious ^l^ | not serious | serious ^c,g^ | serious ^d^ | none | Sami 2015 reported no difference in procedure yield between study arms (p=0.44) [this considers all three study arms]. Jobe 2006 reported 32/121 (26%) of those randomized first to EGD had confirmed BE and 36/121 (30%) randomized to TNE first had confirmed BE (p=0.503). | | | | ⨁◯◯◯ VERY LOW | IMPORTANT |
| Incidence of dysplasia | | | | | | | | | | | | |
| 2 ^e^ | randomized trials | serious ^f^ | not serious | serious ^g,i^ | serious ^d^ | none | 5/72 (6.9%) | 4/84 (4.8%) | **RR 1.54** (0.44 to 5.44) | **26 more per 1,000** (from 27 fewer to 211 more) | ⨁◯◯◯ VERY LOW | IMPORTANT |
| Psychological effects (anxiety before the procedure) | | | | | | | | | | | | |
| 1 ^e^ | randomized trials | very serious ^m^ | not serious | serious ^g^ | serious ^d^ | none | Authors report those who experienced no anxiety, and mild, moderate and severe anxiety. There was no difference between screening modalities (p=0.084) | | | | ⨁◯◯◯ VERY LOW | IMPORTANT |
| Psychological effects (anxiety during insertion) | | | | | | | | | | | | |
| 1 ^e^ | randomized trials | very serious ^m^ | not serious | serious ^g^ | serious ^d^ | none | Authors report those who experienced no anxiety, and mild, moderate and severe anxiety. There was a statistically significant difference between modalities (p=0.0001), with those randomized to TNE experiencing more anxiety during insertion. | | | | ⨁◯◯◯ VERY LOW | IMPORTANT |
| Psychological effects (anxiety during procedure) | | | | | | | | | | | | |
| 3 ^e^ | randomized trials | very serious ^m^ | not serious | serious ^c,g,i^ | serious ^d^ | none | Chang 2011 appears to only have given the questionnaire to the TNE group and reports the results using median score and the range, Sami 2015 reports the results using mean (SD) on a scale of 0-10, and Jobe 2006 reports the results using the number of participants who selected the level of anxiety as "none", "mild", "moderate", and "severe". Both Sami and Jobe report a statistically significant differences between modalities with those randomized to TNE experiencing more anxiety during the procedure, p<0.001 and p=0.0001, respectively. | | | | ⨁◯◯◯ VERY LOW | IMPORTANT |

**CI:** Confidence interval; **EGD**: Esophagogastroduodenoscopy; **RR:** Risk ratio; **TNE**: Transnasal esophagoscopy

**Explanations**

a. Defined in Sami 2015 as safety (adverse events including pain, abdominal discomfort, bleeding, perforation, or need for hospitalization)

b. Many domains were judged as high risk of bias (e.g., allocation concealment, blinding of participants, personnel and outcome assessors)

c. Defined as "heartburn or acid regurgitation >1 week, <1 week, or none" using a GERQ questionnaire

d. Too few participants.

e. One study is a randomized crossover design (Jobe 2006)

f. Many domains were judged as unclear (e.g., sequence generation, allocation concealment, blinding of participants and personnel, etc); as such the overall ROB was considered moderate risk.

g. GERD defined as "heartburn, regurgitation or dysphagia"

h. Many domains were judged as high risk of bias (e.g., blinding of participants, personnel and outcome assessors, etc).

i. Symptoms obtained through questionnaires and were not clearly defined

j. GERD was not defined in the cohort.

k. GERD was not defined and the assessment of the outcome could be influenced by the personnel's knowledge and possible bias to the screening modality.

l. No description of allocation concealment in Sami 2015, and some selective outcome reporting.

m. Participants were aware of what screening modality they were being given and this could influence the level of anxiety.

***Evidence Set 2 - Summary of Findings Table***

| **EGD compared to transnasal esophagoscopy (TNE) for screening for EAC and precancerous conditions (BE and dysplasia)** | | | | | | |
| --- | --- | --- | --- | --- | --- | --- |
| **Setting**: Hospital- and office-based (depending on modality)  **Intervention**: EGD  **Comparison**: TNE | | | | | | |
| Outcomes | **Anticipated absolute effects^*^** (95% CI) | | Relative effect (95% CI) | № of participants  (studies) | Certainty of the evidence (GRADE) | Comments |
|  | **Risk with TNE** | **Risk with EGD** |  |  |  |  |
| Life threatening, severe, or medically significant consequences | Serious adverse events were assessed 1 and 30 days after the procedure. No serious adverse events were reported in any of the study arms. Hospital-based TNE and mobile-based TNE were combined for this outcome under TNE. | |  | 209 (1 RCT) ^a^ | ⨁◯◯◯ VERY LOW ^b,c,d^ |  |
| Incidence of EAC | Among the participants who were receiving their initial screening (i.e., not those undergoing surveillance endoscopy), 0/43 and 0/54 were found to have EAC when randomized to be screened first with EGD or TNE, respectively. | |  | 97 (1 RCT) ^e^ | ⨁◯◯◯ VERY LOW ^d,f,g^ |  |
| Incidence of suspected BE | Sami 2015 reported that there was no difference in procedure yield between study arms with regards to suspected BE (p=0.37) [this considers all three study arms]. Chang 2011 reported 2/20 participants in the EGD and 1/19 participants in the TNE group having suspected BE. | |  | (2 RCTs) | ⨁◯◯◯ VERY LOW ^d,h,i^ |  |
| Incidence of suspected BE | 51 per 1,000 | **106 per 1,000** (66 to 171) | **RR 2.09** (1.30 to 3.36) | 981 (1 observational study) | ⨁◯◯◯ VERY LOW ^d,j,k^ | Includes those with Grade 2 and 3, as those with Grade 1 would not have been considered as BE in Chang 2011 and Sami 2015. |
| Incidence of confirmed BE | Sami 2015 reported no difference in procedure yield between study arms (p=0.44) [this considers all three study arms]. Jobe 2006 reported 32/121 (26%) of those randomized first to EGD had confirmed BE and 36/121 (30%) randomized to TNE first had confirmed BE (p=0.503). | |  | (2 RCTs) ^e^ | ⨁◯◯◯ VERY LOW ^c,d,g,l^ |  |
| Incidence of dysplasia | 48 per 1,000 | **73 per 1,000** (21 to 259) | **RR 1.54** (0.44 to 5.44) | 156 (2 RCTs) ^e^ | ⨁◯◯◯ VERY LOW ^d,f,g,i^ |  |
| Psychological effects (anxiety before the procedure) | Authors report those who experienced no anxiety, and mild, moderate and severe anxiety. There was no difference between screening modalities (p=0.084) | |  | (1 RCT) ^e^ | ⨁◯◯◯ VERY LOW ^d,g,m^ |  |
| Psychological effects (anxiety during insertion) | Authors report those who experienced no anxiety, and mild, moderate and severe anxiety. There was a statistically significant difference between modalities (p=0.0001), with those randomized to TNE experiencing more anxiety during insertion. | |  | (1 RCT) ^e^ | ⨁◯◯◯ VERY LOW ^d,g,m^ |  |
| Psychological effects (anxiety during procedure) | Chang 2011 appears to only have given the questionnaire to the TNE group and reports the results using median score and the range, Sami 2015 reports the results using mean (Standard Deviation) on a scale of 0-10, and Jobe 2006 reports the results using the number of participants who selected the level of anxiety as "none", "mild", "moderate", and "severe". Both Sami and Jobe report a statistically significant differences between modalities with those randomized to TNE experiencing more anxiety during the procedure, p<0.001 and p=0.0001, respectively. | |  | (3 RCTs) ^e^ | ⨁◯◯◯ VERY LOW ^c,d,g,i,m^ |  |
| ***The risk in the intervention group** (and its 95% confidence interval) is based on the assumed risk in the comparison group and the **relative effect** of the intervention (and its 95% CI).   **CI:** Confidence interval; **RR:** Risk ratio | | | | | | |
| **GRADE Working Group grades of evidence** **High certainty:** We are very confident that the true effect lies close to that of the estimate of the effect **Moderate certainty:** We are moderately confident in the effect estimate: The true effect is likely to be close to the estimate of the effect, but there is a possibility that it is substantially different **Low certainty:** Our confidence in the effect estimate is limited: The true effect may be substantially different from the estimate of the effect **Very low certainty:** We have very little confidence in the effect estimate: The true effect is likely to be substantially different from the estimate of effect | | | | | | |

**Explanations**

a. Defined in Sami 2015 as safety (adverse events including pain, abdominal discomfort, bleeding, perforation, or need for hospitalization)

b. Many domains were judged as high risk of bias (e.g., allocation concealment, blinding of participants, personnel and outcome assessors)

c. Defined as "heartburn or acid regurgitation >1 week, <1 week, or none" using a GERQ questionnaire

d. Too few participants.

e. One study is a randomized crossover design (Jobe 2006)

f. Many domains were judged as unclear (e.g., sequence generation, allocation concealment, blinding of participants and personnel, etc); as such the overall ROB was considered moderate risk.

g. GERD defined as "heartburn, regurgitation or dysphagia"

h. Many domains were judged as high risk of bias (e.g., blinding of participants, personnel and outcome assessors, etc).

i. Symptoms obtained through questionnaires and were not clearly defined

j. GERD was not defined in the cohort.

k. GERD was not defined and the assessment of the outcome could be influenced by the personnel's knowledge and possible bias to the screening modality.

l. No description of allocation concealment in Sami 2015, and some selective outcome reporting.

m. Participants were aware of what screening modality they were being given and this could influence the level of anxiety.

**Evidence Set 3: Esophagogastroduodenoscopy (EGD) versus Video capsule esophagoscopy (VCE)**

***Evidence Set 3 - Results table***

| **Author Year**  **Study design** | **Outcome(s) description** | **Results** | | **ROB assessment** | **Notes** |
| --- | --- | --- | --- | --- | --- |
|  |  | **EGD** | **VCE** |  |  |
| **IMPORTANT OUTCOME 4: Incidence of EAC (by stage), BE, and low- and high-grade dysplasia** | | | | | |
| **Suspected BE (endoscopically)** | | | | | |
| Chang 2011^51^  RCT | Endoscopically suspected BE, classified as endoscopic presence of 1 cm or more of columnar-lined distal esophagus above the gastroesophageal junction (with EGD) and as ZAP grade 2 or 3 (with VCE). | 2/20 (10%) | 3/17 (18%) | High | Three VCE patients had suspected BE and were offered EGD. |
| **Confirmed BE (histologically)** | | | | | |
| Chang 2011^51^  RCT | Confirmed BE. Patients suspected of BE on VCE were offered EGD and BE was confirmed through biopsy. | Not reported | 0/3 (0%) | High | The three patients in the were those who were identified as having suspected BE based on VCE. BE was histologically confirmated with EGD. All cases of BE were short-segment. |
| **Dysplasia** | | | | | |
| Chang 2011^51^  RCT | Dysplasia (not defined) | 0/20 (0%) | 0/20 (0%) | Moderate |  |

***Evidence Set 3 - Forest Plot***

*Forest Plot 3.1: Incidence of endoscopically suspected BE*


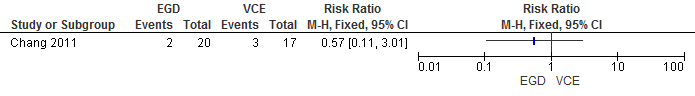


***Evidence Set 3 - GRADE evidence profile table***

| **Certainty assessment** | | | | | | | **№ of patients** | | **Effect** | | **Certainty** | **Importance** |
| --- | --- | --- | --- | --- | --- | --- | --- | --- | --- | --- | --- | --- |
| **№ of studies** | **Study design** | **Risk of bias** | **Inconsistency** | **Indirectness** | **Imprecision** | **Other considerations** | **EGD** | **VCE** | **Relative (95% CI)** | **Absolute (95% CI)** |  |  |
| Incidence of suspected BE | | | | | | | | | | | | |
| 1 | randomized trials | very serious ^a^ | not serious | serious ^b^ | serious ^c^ | none | 2/20 (10.0%) | 3/17 (17.6%) | **RR 0.57** (0.11 to 3.01) | **76 fewer per 1,000** (from 157 fewer to 355 more) | ⨁◯◯◯ VERY LOW | IMPORTANT |
| Incidence of confirmed BE | | | | | | | | | | | | |
| 1 | randomized trials | serious ^d^ | not serious | serious ^b^ | serious ^c^ | none | Authors do not report on the incidence of confirmed BE in the EGD group and 0 of 3 had confirmed BE in the VCE group. | | | | ⨁◯◯◯ VERY LOW | IMPORTANT |
| Incidence of dysplasia | | | | | | | | | | | | |
| 1 | randomized trials | serious ^d^ | not serious | serious ^b^ | serious ^c^ | none | There were no cases of dysplasia in either group. | | | | ⨁◯◯◯ VERY LOW | IMPORTANT |

**CI:** Confidence interval; **EGD**: Esophagogastroduodenoscopy; **RR:** Risk ratio; **VCE**: video capsule esophagoscopy

**Explanations**

a. The lack of blinding of the study personnel could influence this outcome. There were also many other domains that were judged as unclear risk (e.g., sequence randomization, allocation concealment, blinding of outcome assessors, etc).

b. Chronic GERD was not defined. Symptoms were obtained through validated questionnaires.

c. Too few participants.

d. There were many domains that were unclear risk of bias, due to lack of reporting (e.g., sequence generation, allocation concealment, blinding of outcome assessors, etc); as such, it has been judged as moderate risk of bias.

***Evidence Set 3 - Summary of Findings Table***

| **Sedated EGD compared to esophageal video capsule esophagoscopy (VCE) for screening for EAC and precancerous conditions (BE and dysplasia)** | | | | | | |
| --- | --- | --- | --- | --- | --- | --- |
| **Setting**: Clinical Research Centre  **Intervention**: EGD  **Comparison**: Video capsule esophagoscopy (VCE) | | | | | | |
| Outcomes | **Anticipated absolute effects^*^** (95% CI) | | Relative effect (95% CI) | № of participants  (studies) | Certainty of the evidence (GRADE) | Comments |
|  | **Risk with VCE** | **Risk with EGD** |  |  |  |  |
| Incidence of suspected BE | 176 per 1,000 | **101 per 1,000** (19 to 531) | **RR 0.57** (0.11 to 3.01) | 37 (1 RCT) | ⨁◯◯◯ VERY LOW ^a,b,c^ |  |
| Incidence of confirmed BE | Authors do not report on the incidence of confirmed BE in the EGD group and 0 of 3 had confirmed BE in the VCE group. | |  | 17 (1 RCT) | ⨁◯◯◯ VERY LOW ^b,c,d^ |  |
| Incidence of dysplasia | There were no cases of dysplasia in either group. | |  | 40 (1 RCT) | ⨁◯◯◯ VERY LOW ^b,c,d^ |  |
| ***The risk in the intervention group** (and its 95% confidence interval) is based on the assumed risk in the comparison group and the **relative effect** of the intervention (and its 95% CI).   **CI:** Confidence interval; **EGD**: Esophagogastroduodenoscopy; **RR:** Risk ratio; **VCE**: video capsule esophagoscopy | | | | | | |
| **GRADE Working Group grades of evidence** **High certainty:** We are very confident that the true effect lies close to that of the estimate of the effect **Moderate certainty:** We are moderately confident in the effect estimate: The true effect is likely to be close to the estimate of the effect, but there is a possibility that it is substantially different **Low certainty:** Our confidence in the effect estimate is limited: The true effect may be substantially different from the estimate of the effect **Very low certainty:** We have very little confidence in the effect estimate: The true effect is likely to be substantially different from the estimate of effect | | | | | | |

**Explanations**

a. The lack of blinding of the study personnel could influence this outcome. There were also many other domains that were judged as unclear risk (e.g., sequence randomization, allocation concealment, blinding of outcome assessors, etc).

b. Chronic GERD was not defined. Symptoms were obtained through validated questionnaires.

c. Too few participants.

d. There were many domains that were unclear risk of bias, due to lack of reporting (e.g., sequence generation, allocation concealment, blinding of outcome assessors, etc); as such, it has been judged as moderate risk of bias.

**Evidence Set 4: Esophagogastroduodenoscopy (EGD) versus Transoral-EGD**

***Evidence Set 4 - Results table***

| **Author Year**  **Study design** | **Outcome(s) description** | **Results** | | **ROB assessment** | **Notes** |
| --- | --- | --- | --- | --- | --- |
|  |  | **EGD** | **Transoral-EGD** |  |  |
| **IMPORTANT OUTCOME 4: Incidence of EAC (by stage), BE, and low- and high-grade dysplasia** | | | | | |
| **Suspected BE (endoscopically)** | | | | | |
| Mori 2010^54^  Cohort study | Measurment of the mucosa between the esophagogastric junction and squamocolumnar junction  Grade 1^23^  Grade 2^24^  Grade 3^25^ | 61/254 (24%) | 150/599 (25%) | High | Patients were asked to select between screening options. There was no significant difference in the frequency distribution of the severity of BE among EGDs. |
|  |  | 26/254 (10.2%) | 46/599 (7.7%) |  |  |
|  |  | 1/254 (0.39%) | 3/599 (0.5%) |  |  |

^23^ BE grade 1 (circumferential (C) or tongue (T) type, longest BE <1cm)

^24^ BE grade 2 (C type, 3cm > longest BE ≥1cm or T type, longest BE ≥1cm)

^25^ BE grade 3 (C type, shortest BE ≥3cm)

***Evidence Set 4 - Forest Plot***

*Forest Plot 4.1: Incidence of suspected BE (grade 2 and 3)*


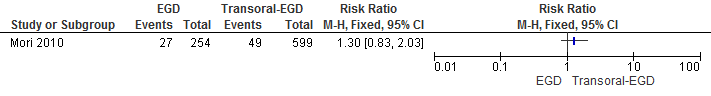


***Evidence Set 4 - GRADE evidence profile table***

| **Certainty assessment** | | | | | | | **№ of patients** | | **Effect** | | **Certainty** | **Importance** |
| --- | --- | --- | --- | --- | --- | --- | --- | --- | --- | --- | --- | --- |
| **№ of studies** | **Study design** | **Risk of bias** | **Inconsistency** | **Indirectness** | **Imprecision** | **Other considerations** | **EGD** | **Transoral EGD** | **Relative (95% CI)** | **Absolute (95% CI)** |  |  |
| Incidence of suspected BE | | | | | | | | | | | | |
| 1 | observational studies | very serious ^a^ | not serious | serious ^b^ | serious ^c^ | none | 27/254 (10.6%) | 49/599 (8.2%) | **RR 1.30** (0.83 to 2.03) | **25 more per 1,000** (from 14 fewer to 84 more) | ⨁◯◯◯ VERY LOW | IMPORTANT |

**CI:** Confidence interval; **RR:** Risk ratio

**Explanations**

a. A cohort who had received prior EGD who were allowed to select which screening modality they were exposed to. There is no description on how the outcome was assessed.

b. One of the main objectives of the study was to diagnose GERD. Patients who had previous EGD for screening upper intestinal tract disorders were enrolled in the present study.

c. Too few participants.

***Evidence Set 4 - Summary of Findings Table***

| **EGD compared to transoral EGD for screening for EAC and precancerous conditions (BE and dysplasia)** | | | | | | |
| --- | --- | --- | --- | --- | --- | --- |
| **Setting**: Hospital  **Intervention**: EGD  **Comparison**: Transoral EGD | | | | | | |
| Outcomes | **Anticipated absolute effects^*^** (95% CI) | | Relative effect (95% CI) | № of participants  (studies) | Certainty of the evidence (GRADE) | Comments |
|  | **Risk with transoral EGD** | **Risk with EGD** |  |  |  |  |
| Incidence of suspected BE | 82 per 1,000 | **106 per 1,000** (68 to 166) | **RR 1.30** (0.83 to 2.03) | 853 (1 observational study) | ⨁◯◯◯ VERY LOW ^a,b,c^ | Includes those with Grade 2 and 3, as those with Grade 1 would not normally be considered as having BE based on other included studies (Chang 2011 and Sami 2015). |
| ***The risk in the intervention group** (and its 95% confidence interval) is based on the assumed risk in the comparison group and the **relative effect** of the intervention (and its 95% CI).   **CI:** Confidence interval; **RR:** Risk ratio | | | | | | |
| **GRADE Working Group grades of evidence** **High certainty:** We are very confident that the true effect lies close to that of the estimate of the effect **Moderate certainty:** We are moderately confident in the effect estimate: The true effect is likely to be close to the estimate of the effect, but there is a possibility that it is substantially different **Low certainty:** Our confidence in the effect estimate is limited: The true effect may be substantially different from the estimate of the effect **Very low certainty:** We have very little confidence in the effect estimate: The true effect is likely to be substantially different from the estimate of effect | | | | | | |

**Explanations**

a. A cohort who had received prior EGD who were allowed to select which screening modality they were exposed to. There is no description on how the outcome was assessed.

b. One of the main objectives of the study was to diagnose GERD. Patients who had previous EGD for screening upper intestinal tract disorders were enrolled in the present study.

c. Too few participants.

**Evidence Set 5: Transnasal esophagoscopy (TNE) versus Video capsule esophagoscopy (VCE)**

***Evidence Set 5 - Results table***

| **Author Year**  **Study design** | **Outcome(s) description** | **Results** | | **ROB assessment** | **Notes** |
| --- | --- | --- | --- | --- | --- |
|  |  | **TNE** | **VCE** |  |  |
| **IMPORTANT OUTCOME 4: Incidence of EAC (by stage), BE, and low- and high-grade dysplasia** | | | | | |
| **Suspected BE (endoscopically)** | | | | | |
| Chak 2014^55^  RCT | Suspected BE (ZAP classification) determined the need for biopsy. | 10/87 | 9/90 | High | Subjects with suspected BE (ZAP grade 2 or higher) or other findings (e.g., mass) were referred for EGD. |
| Chang 2011^51^  RCT | Suspected BE, classified as endoscopic presence of 1 cm or more of columnar-lined distal esophagus above the gastroesophageal junction (for TNE) or as ZAP grade 2 or 3, and patients were offered EGD for confirmation (for VCE). | 1/19 (5%) | 3/17 (18%) | High | All cases of BE were short-segment.  Three VCE patients had suspected BE and were offered EGD. Results of exam and biopsy were normal. |
| **Confirmed BE (histologically)** | | | | | |
| Chak 2014^55^  RCT | BE (Prague classification) confirmed through sedated EGD and histologic examination of biopsy. | 3/87 | 5/90 | Low | Subjects with suspected BE (ZAP grade 2 or higher) or other findings (e.g., mass) were referred for EGD. |
|  |  | p-value=0.49 | |  |  |
| Chang 2011^51^  RCT | Confirmed BE by sedated EGD exam and biopsy | Not reported | 0/3 (0%) | High |  |
| **Dysplasia** | | | | | |
| Chang 2011^51^  RCT | Dysplasia (not defined) | 0/20 | 0/20 | Moderate |  |
| **IMPORTANT OUTCOME 6: Psychological effects** | | | | | |
| Chak 2014^55^  RCT | Anxiety, nervousness, or worry before the procedure | 33/87 (38%) | 15/90 (17%) | High |  |
|  |  | p-value=0.001 | |  |  |
| Chak 2014^55^  RCT | Anxiety, nervousness, or worry during the procedure | 29/87 (33%) | 14/90 (16%) |  |  |
|  |  | p-value=0.006 | |  |  |

***Evidence Set 5 - Forest Plots***

*Forest Plot 5.1: Incidence of endoscopically suspected BE*


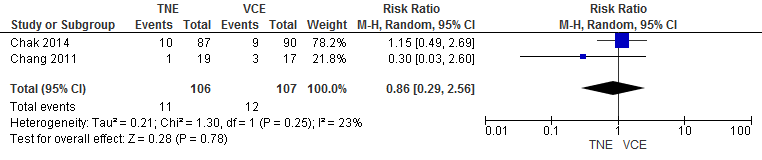


*Forest Plot 5.2: Anxiety before the procedure*


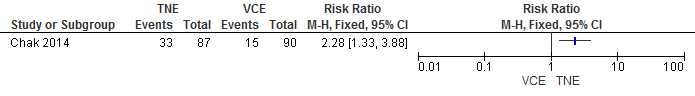


*Forest Plot 5.3: Anxiety during the procedure*


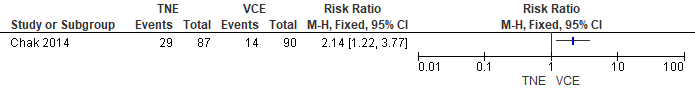


***Evidence Set 5 - GRADE evidence profile table***

| **Certainty assessment** | | | | | | | **№ of patients** | | **Effect** | | **Certainty** | | **Importance** |
| --- | --- | --- | --- | --- | --- | --- | --- | --- | --- | --- | --- | --- | --- |
| **№ of studies** | **Study design** | **Risk of bias** | **Inconsistency** | **Indirectness** | **Imprecision** | **Other considerations** | **TNE** | **VCE** | **Relative (95% CI)** | **Absolute (95% CI)** |  |  |  |
| Incidence of suspected BE | | | | | | | | | | | | | |
| 2 | randomized trials | very serious ^a^ | not serious | serious ^b,c^ | serious ^d^ | none | 11/106 (10.4%) | 12/107 (11.2%) | **RR 0.86** (0.29 to 2.56) | **16 fewer per 1,000** (from 80 fewer to 175 more) | ⨁◯◯◯ VERY LOW | | IMPORTANT |
| Incidence of confirmed BE | | | | | | | | | | | | | |
| 2 | randomized trials | serious ^e^ | not serious | serious ^b,c^ | serious ^d^ | none | Chak 2014 reports 3/87 and 5/90 (p=0.49) cases of confirmed BE from TNE and VCE, respectively. Chang 2011 does not report how many cases of confirmed BE were in the TNE group, and reports that 0/3 of those with suspected BE were confirmed. | | | | | ⨁◯◯◯ VERY LOW | IMPORTANT |
| Incidence of dysplasia | | | | | | | | | | | | | |
| 1 | randomized trials | serious ^f^ | not serious | serious ^c^ | serious ^d^ | none | There were no cases of dysplasia in either group. | | | | | ⨁◯◯◯ VERY LOW | IMPORTANT |
| Psychological effects (anxiety, nervousness, or worry before the procedure) | | | | | | | | | | | | | |
| 1 | randomized trials | very serious ^g^ | not serious | serious ^b^ | serious ^d^ | none | 33/87 (37.9%) | 15/90 (16.7%) | **RR 2.28** (1.33 to 3.88) | **213 more per 1,000** (from 55 more to 480 more) | ⨁◯◯◯ VERY LOW | | IMPORTANT |
| Psychological effects (anxiety during the procedure) | | | | | | | | | | | | | |
| 1 | randomized trials | very serious ^g^ | not serious | serious ^b,c^ | serious ^d^ | none | 29/87 | 14/90 | **RR 2.14**  (1.22 to 3.77) | **177 more per 1,000**  (from 34 more to 431 more) | ⨁◯◯◯ VERY LOW | | IMPORTANT |

**CI:** Confidence interval; **RR:** Risk ratio; **TNE**: Transnasal esophagoscopy; **VCE**: video capsule esophagoscopy

**Explanations**

a. Personnel and outcome assessors were aware of screening modality and could be influenced by this knowledge.

b. Chak 2014 defined GERD based on symptoms of GERD (from questionnaire) or use of acid suppression medicine (within 7 days of screening).

c. Chang 2011 defined GERD based on symptoms obtained through validated questionnaires.

d. Too few participants.

e. Chak 2014 was considered low risk but contributed a greater amount of data to the outcome. Chang 2011 was considered high risk, but only contributed 20 participants to each comparison.

f. Many ROB domains were unclear due to lack of reporting for this study.

g. Participants were aware of screening modality and could be influenced by this knowledge. Personnel could also influence the level of anxiety by knowledge of the screening modality.

***Evidence Set 5 - Summary of Findings Table***

| **Transnasal esophagoscopy (TNE) compared to esophageal video capsule esophagoscopy (VCE) for screening for EAC and precancerous conditions (BE and dysplasia)** | | | | | | |
| --- | --- | --- | --- | --- | --- | --- |
| **Setting**: Outpatient clinic and Clinical Research Centre (depending on study)  **Intervention**: TNE  **Comparison**: VCE | | | | | | |
| Outcomes | **Anticipated absolute effects^*^** (95% CI) | | Relative effect (95% CI) | № of participants  (studies) | Certainty of the evidence (GRADE) | Comments |
|  | **Risk with VCE** | **Risk with TNE** |  |  |  |  |
| Incidence of suspected BE | 112 per 1,000 | **96 per 1,000** (33 to 287) | **RR 0.86** (0.29 to 2.56) | 213 (2 RCTs) | ⨁◯◯◯ VERY LOW ^a,b,c,d^ |  |
| Incidence of confirmed BE | Chak 2014 reports 3/87 and 5/90 (p=0.49) cases of confirmed BE from TNE and VCE, respectively. Chang 2011 does not report how many cases of confirmed BE were in the TNE group, and reports that 0/3 of those with suspected BE were confirmed. | |  | 93 (2 RCTs) | ⨁◯◯◯ VERY LOW ^b,c,d,e^ |  |
| Incidence of dysplasia | There were no cases of dysplasia in either group. | |  | 40 (1 RCT) | ⨁◯◯◯ VERY LOW ^c,d,f^ |  |
| Psychological effects (anxiety, nervousness, or worry before the procedure) | 167 per 1,000 | **380 per 1,000** (222 to 647) | **RR 2.28** (1.33 to 3.88) | 177 (1 RCT) | ⨁◯◯◯ VERY LOW ^b,d,g^ |  |
| Psychological effects (anxiety during the procedure) | 156 per 1,000 | **333 per 1,000**  (190 to 586) | **RR 2.14**  (1.22 to 3.77) | 177 (1 RCTs) | ⨁◯◯◯ VERY LOW ^b,c,d,g^ |  |
| ***The risk in the intervention group** (and its 95% confidence interval) is based on the assumed risk in the comparison group and the **relative effect** of the intervention (and its 95% CI).   **CI:** Confidence interval; **RR:** Risk ratio; **TNE**: Transnasal esophagoscopy; **VCE**: video capsule esophagoscopy | | | | | | |
| **GRADE Working Group grades of evidence** **High certainty:** We are very confident that the true effect lies close to that of the estimate of the effect **Moderate certainty:** We are moderately confident in the effect estimate: The true effect is likely to be close to the estimate of the effect, but there is a possibility that it is substantially different **Low certainty:** Our confidence in the effect estimate is limited: The true effect may be substantially different from the estimate of the effect **Very low certainty:** We have very little confidence in the effect estimate: The true effect is likely to be substantially different from the estimate of effect | | | | | | |

**Explanations**

a. Personnel and outcome assessors were aware of screening modality and could be influenced by this knowledge.

b. Chak 2014 defined GERD based on symptoms of GERD (from questionnaire) or use of acid suppression medicine (within 7 days of screening).

c. Chang 2011 defined GERD based on symptoms obtained through validated questionnaires.

d. Too few participants.

e. Chak 2014 was considered low risk but contributed a greater amount of data to the outcome. Chang 2011 was considered high risk, but only contributed 20 participants to each comparison.

f. Many ROB domains were unclear due to lack of reporting for this study.

g. Participants were aware of screening modality and could be influenced by this knowledge. Personnel could also influence the level of anxiety by knowledge of the screening modality.

**Evidence Set 6: Transnasal esophagoscopy (TNE) versus Transoral EGD**

***Evidence Set 6 - Results table***

| **Author Year**  **Study design** | **Outcome(s) description** | **Results** | | | **ROB assessment** | | **Notes** | |
| --- | --- | --- | --- | --- | --- | --- | --- | --- |
|  |  | **TNE** | | **Transoral-EGD** |  |  |  |  |
| **CRITICAL OUTCOME 3: Life threatening, severe, or medically significant consequenes** | | | | | | | | |
| Zaman 1999^56^  RCT | Several hours after discharge, facial swelling developed, and eventually a small proximal esophageal performation was diagnosed by means of an x-ray swallowing series using water-soluable contrast. Surgical exploration of the neck did not reveal a perforation. | 1/25 | 0/34 | | | Moderate | | A woman undergoing endoscopy for abdominal pain and early satiety. |
| **IMPORTANT OUTCOME 4: Incidence of EAC (by stage), BE, and low- and high-grade dysplasia** | | | | | | | | |
| **Suspected BE (endoscopically)** | | | | | | | | |
| Zaman 1999^56^  RCT | Not defined | 1/25 | 2/34 | | | High | | Patients were self-selected, as 43% of those approached declined to participate. |
| Mori 2010^54^  Cohort study | Measurement of the mucosa between the esophagogastric junction and squamocolumnar junction  Grade 1^23^  Grade 2^24^  Grade 3^25^ | 188/727 (25.9%) | 150/599 (25%) | | | High | | Patients were asked to select between screening options. There was no significant difference in the frequency distribution of the severity of BE among EGDs. |
|  |  | 31/727 (4.3%) | 46/599 (7.7%) | | |  |  |  |
|  |  | 6/727 (0.8%) | 3/599 (0.5%) | | |  |  |  |
| **IMPORTANT OUTCOME 6: Psychological effects** | | | | | | | | |
| Zaman 1999^56^  RCT | Anxiety before the procedure (mean ± SE) | 3.0 ± 0.6 | 3.6 ± 0.5 | | | High | |  |
|  |  | p=0.39 | | | |  |  |  |
| Zaman 1999^56^  RCT | Anxiety during insertion (mean ± SE) | 4.4 ± 0.6 | 4.7 ± 0.5 | | |  |  |  |
|  |  | p=0.63 | | | |  |  |  |
| Zaman 1999^56^  RCT | Anxiety during the procedure (mean ± SE) | 3.3 ± 0.7 | 3.3 ± 0.5 | | |  |  |  |
|  |  | p=0.99 | | | |  |  |  |

^23^ BE grade 1 (circumferential (C) or tongue (T) type, longest BE <1cm)

^24^ BE grade 2 (C type, 3cm > longest BE ≥1cm or T type, longest BE ≥1cm)

^25^ BE grade 3 (C type, shortest BE ≥3cm)

***Evidence Set 6 - Forest Plots***

*Forest Plot 6.1: Life threatening, severe, or medically significant consequences*


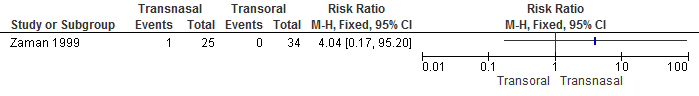


*Forest Plot 6.2: Incidence of suspected BE (RCT)*


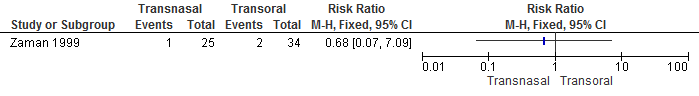


*Forest Plot 6.3: Incidence of suspected BE (grade 2 and 3) (observational)*


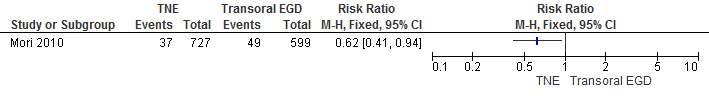


*Forest Plot 6.4: Anxiety before the procedure*


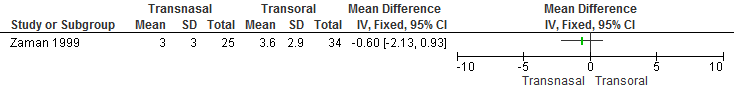


*Forest Plot 6.5: Anxiety during insertion*


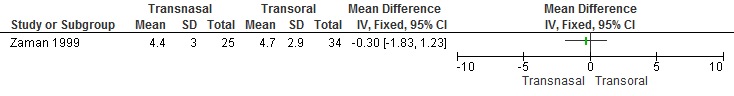


*Forest Plot 6.6: Anxiety during the procedure*


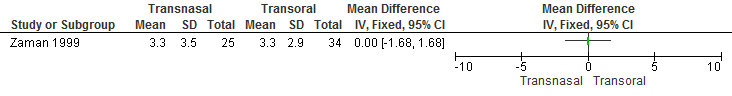


***Evidence Set 6 - GRADE evidence profile table***

**Setting**: Hospital

**Bibliography**: Zaman A, Hahn M, Hapke R, Knigge K, Fennerty MB, Katon RM. A randomized trial of peroral versus transnasal unsedated endoscopy using an ultrathin videoendoscope. *Gastrointestinal Endoscopy* 1999; 49(3):279-284. Mori A, Ohashi N, Yoshida A, Nozaki M, Tatcbe H, Okuno M, Hoshihara Y, Hongo M. Unsedated transnasal ultrathin esophagogastroduodenoscopy may provide better diagnostic performance in gastroesophageal reflux disease. *Disease of the Esophagus* 2011; 24:92-98.

| **Certainty assessment** | | | | | | | **№ of patients** | | **Effect** | | **Certainty** | **Importance** |
| --- | --- | --- | --- | --- | --- | --- | --- | --- | --- | --- | --- | --- |
| **№ of studies** | **Study design** | **Risk of bias** | **Inconsistency** | **Indirectness** | **Imprecision** | **Other considerations** | **TNE** | **Transoral EGD** | **Relative (95% CI)** | **Absolute (95% CI)** |  |  |
| Life threatening, severe, or medically significant consequences | | | | | | | | | | | | |
| 1 | randomised trials | serious ^a^ | not serious | serious ^b^ | serious ^c^ | none | 1/25 (4.0%) | 0/34 (0.0%) | not estimable |  | ⨁◯◯◯ VERY LOW | IMPORTANT |
| Incidence of suspected BE (RCT) | | | | | | | | | | | | |
| 1 | randomised trials | very serious ^a^ | not serious | serious ^b^ | serious ^c^ | none | 1/25 (4.0%) | 2/34 (5.9%) | **RR 0.68**  (0.07 to 7.09) | **19 fewer per 1,000**  (from 55 fewer to 358 more) | ⨁◯◯◯ VERY LOW | IMPORTANT |
| Incidence of suspected BE (grade 2 and 3) (obs) | | | | | | | | | | | | |
| 1 | observational studies | very serious ^d^ | not serious | serious ^e^ | serious ^c^ | none | 37/727 (5.1%) | 49/599 (8.2%) | **RR 0.62** (0.41 to 0.94) | **31 fewer per 1,000** (from 5 fewer to 48 fewer) | ⨁◯◯◯ VERY LOW | IMPORTANT |
| Anxiety prior to screening (Scale from: 0 to 10) | | | | | | | | | | | | |
| 1 | randomised trials | very serious ^a,f^ | not serious | serious ^b^ | serious ^c^ | none | 25 | 34 | - | MD **0.6 lower** (2.13 lower to 0.93 higher) | ⨁◯◯◯ VERY LOW | IMPORTANT |
| Anxiety during insertion (Scale from: 0 to 10) | | | | | | | | | | | | |
| 1 | randomised trials | very serious ^a,f^ | not serious | serious ^b^ | serious ^c^ | none | 25 | 34 | - | MD **0.3 lower** (1.83 lower to 1.23 higher) | ⨁◯◯◯ VERY LOW | IMPORTANT |
| Anxiety during the procedure (Scale from: 0 to 10) | | | | | | | | | | | | |
| 1 | randomised trials | very serious ^a,f^ | not serious | serious ^b^ | serious ^c^ | none | 25 | 34 | - | MD **0**  (1.68 lower to 1.68 higher) | ⨁◯◯◯ VERY LOW | IMPORTANT |

**CI:** Confidence interval; **EGD**: Esophagogastroduodenoscopy; **MD:** Mean Difference; **RR:** Risk ratio; **TNE**: Transnasal esophagoscopy

**Explanations**

a. There is no information provided on the method of randomization or allocation concealment. No protocol was found to determine if all outcomes were reported. There was no information provided on how the study was funded.

b. Participants were selected among those with upper GI symptoms, of which GERD was one reason. Other reasons included dyspepsia, abdominal pain, nausea/vomiting, anemia.

c. Too few participants.

d. A cohort who had received prior EGD who were allowed to select which screening modality they were exposed to. There is no description on how the outcome was assessed.

e. One of the main objectives of the study was to diagnose GERD. Patients who had previous EGD for screening upper intestinal tract disorders were enrolled in the present study.

f. Randomization to a particular method could cause different levels of anxiety prior to the procedure and during the procedure.

***Evidence Set 6 - Summary of Findings table***

| **TNE compared to Transoral EGD for screening for EAC and precancerous conditions (BE and dysplasia)** | | | | | | |
| --- | --- | --- | --- | --- | --- | --- |
| **Setting**: Hospital-based  **Intervention**: TNE  **Comparison**: Transoral EGD | | | | | | |
| Outcomes | **Anticipated absolute effects^*^** (95% CI) | | Relative effect (95% CI) | № of participants  (studies) | Certainty of the evidence (GRADE) | Comments |
|  | **Risk with Transoral EGD** | **Risk with TNE** |  |  |  |  |
| Life threatening, severe, or medically significant consequences | 0 per 1,000 | Not estimable due to zero count in comparison group | not estimable | 59 (1 RCT) | ⨁◯◯◯ VERY LOW ^a,b,c^ |  |
| Incidence of suspected BE (RCT) | 59 per 1,000 | **40 per 1,000** (4 to 417) | **RR 0.68**  (0.07 to 7.09) | 59 (1 RCT) | ⨁◯◯◯ VERY LOW ^a,b,c^ |  |
| Incidence of suspected BE(grade 2 and 3) (obs) | 82 per 1,000 | **51 per 1,000** (34 to 77) | **RR 0.62** (0.41 to 0.94) | 1326 (1 observational study) | ⨁◯◯◯ VERY LOW ^c,d,e^ |  |
| Anxiety prior to screening Scale from: 0 to 10 |  | The mean anxiety prior to screening in the intervention group was 0.6 lower (2.13 lower to 0.93 higher) | - | 59 (1 RCT) | ⨁◯◯◯ VERY LOW ^a,b,c,f^ |  |
| Anxiety during insertion Scale from: 0 to 10 |  | The mean anxiety during insertion in the intervention group was 0.3 lower (1.83 lower to 1.23 higher) | - | 59 (1 RCT) | ⨁◯◯◯ VERY LOW ^a,b,c,f^ |  |
| Anxiety during the procedure Scale from: 0 to 10 |  | The mean anxiety during the procedure in the intervention group was 0 (1.68 lower to 1.68 higher) | - | 59 (1 RCT) | ⨁◯◯◯ VERY LOW ^a,b,c,f^ |  |
| ***The risk in the intervention group** (and its 95% confidence interval) is based on the assumed risk in the comparison group and the **relative effect** of the intervention (and its 95% CI).  **CI:** Confidence interval; **EGD**: Esophagogastroduodenoscopy; **RR:** Risk ratio; **TNE**: Transnasal esophagoscopy | | | | | | |
| **GRADE Working Group grades of evidence** **High certainty:** We are very confident that the true effect lies close to that of the estimate of the effect **Moderate certainty:** We are moderately confident in the effect estimate: The true effect is likely to be close to the estimate of the effect, but there is a possibility that it is substantially different **Low certainty:** Our confidence in the effect estimate is limited: The true effect may be substantially different from the estimate of the effect **Very low certainty:** We have very little confidence in the effect estimate: The true effect is likely to be substantially different from the estimate of effect | | | | | | |

**Evidence Set 7: EGD with random biopsy versus Enhanced magnification-directed endoscopy (EME) biopsies**

***Evidence Set 7 - Results table***

| **Author Year**  **Study design** | **Outcome(s) description** | **Results** | | **ROB assessment** | **Notes** |
| --- | --- | --- | --- | --- | --- |
|  |  | **EGD with random biopsy** | **EME directed endoscopy biopsies** |  |  |
| **IMPORTANT OUTCOME 4: Incidence of EAC (by stage), BE, and low- and high-grade dysplasia** | | | | | |
| **Confirmed BE (histologically)** | | | | | |
| Ferguson 2006^57^ RCT | Specialized intestinal metaplasia (SIM) among those with endoscopically apparent BE, which was defined as any variation of >5mm between the lowest and highest point of the squamocolumnar junction from the gastroesophageal junction using only the findings on standard (or non-magnified) endoscopy. The first result includes only those with SIM patterns III/IV, while the second results include all SIM pattern types. | 12/20 (60%) | 11/18 (61%) | Moderate | EME tissue was classified using four pattern types [I round pits, II recticular, III villous, and IV ridged]. |
|  |  | p-value: 1.00 | |  |  |
|  |  | 12/20 (60%) | 19/36 (53%) |  |  |
|  |  | p-value: 0.78 | |  |  |

***Evidence Set 7 - Forest Plots***

*Forest Plot 7.1: Incidence of confirmed BE (specialized intestinal metaplasia pattern III and IV)*


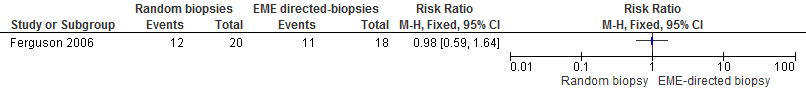


*Forest Plot 7.2: Incidence of confirmed BE (specialized intestinal metaplasia all patterns)*


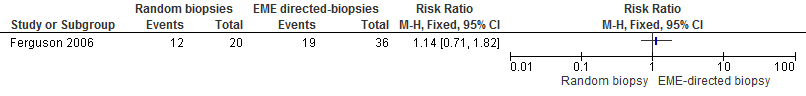


***Evidence Set 7 - GRADE evidence profile table***

| **Certainty assessment** | | | | | | | **№ of patients** | | **Effect** | | **Certainty** | **Importance** |
| --- | --- | --- | --- | --- | --- | --- | --- | --- | --- | --- | --- | --- |
| **№ of studies** | **Study design** | **Risk of bias** | **Inconsistency** | **Indirectness** | **Imprecision** | **Other considerations** | **EGD with random biopsy** | **EME-directed endoscopy biopsies** | **Relative (95% CI)** | **Absolute (95% CI)** |  |  |
| Incidence of confirmed BE (SIM pattern types III/IV) | | | | | | | | | | | | |
| 1 | randomized trials | serious ^a^ | not serious | serious ^b^ | serious ^c^ | none | 12/20 (60.0%) | 11/18 (61.1%) | **RR 0.98** (0.59 to 1.64) | **12 fewer per 1,000** (from 251 fewer to 391 more) | ⨁◯◯◯ VERY LOW | IMPORTANT |
| Incidence of confirmed BE (all SIM pattern types) | | | | | | | | | | | | |
| 1 | randomized trials | serious ^a^ | not serious | serious ^b^ | serious ^c^ | none | 12/20 (60.0%) | 19/36 (52.8%) | **RR 1.14** (0.71 to 1.82) | **74 more per 1,000** (from 153 fewer to 433 more) | ⨁◯◯◯ VERY LOW | IMPORTANT |

**CI:** Confidence interval; **RR:** Risk ratio

**Explanations**

a. No details on allocation concealment, no protocol found, and no details on funding.

b. GERD score determined with a validated questionnaire (Ofman J, et al. Identifying patients with gastroesophageal reflux disease: Validation of a practical screening tool. Dig Dis Sci 2002; 47:1863-9), with no other information provided

c. Too few participants in the study

***Evidence Set 7 - Summary of Findings Table***

| **EGD with random biopsy compared to EME-directed endoscopy biopsies for screening for EAC and precancerous conditions (BE and dysplasia)** | | | | | | |
| --- | --- | --- | --- | --- | --- | --- |
| **Setting**: Outpatient clinic  **Intervention**: EGD random biopsy  **Comparison**: EME-directed endoscopy biopsies | | | | | | |
| Outcomes | **Anticipated absolute effects^*^** (95% CI) | | Relative effect (95% CI) | № of participants  (studies) | Certainty of the evidence (GRADE) | Comments |
|  | **Risk with EME-directed endoscopy biopsies** | **Risk with EGD with random biopsy** |  |  |  |  |
| Incidence of confirmed BE (SIM pattern types III/IV) | 611 per 1,000 | **599 per 1,000** (361 to 1,000) | **RR 0.98** (0.59 to 1.64) | 38 (1 RCT) | ⨁◯◯◯ VERY LOW ^a,b,c^ |  |
| Incidence of confirmed BE (all SIM pattern types) | 528 per 1,000 | **602 per 1,000** (375 to 961) | **RR 1.14** (0.71 to 1.82) | 56 (1 RCT) | ⨁◯◯◯ VERY LOW ^a,b,c^ |  |
| ***The risk in the intervention group** (and its 95% confidence interval) is based on the assumed risk in the comparison group and the **relative effect** of the intervention (and its 95% CI).   **CI:** Confidence interval; **RR:** Risk ratio | | | | | | |
| **GRADE Working Group grades of evidence** **High certainty:** We are very confident that the true effect lies close to that of the estimate of the effect **Moderate certainty:** We are moderately confident in the effect estimate: The true effect is likely to be close to the estimate of the effect, but there is a possibility that it is substantially different **Low certainty:** Our confidence in the effect estimate is limited: The true effect may be substantially different from the estimate of the effect **Very low certainty:** We have very little confidence in the effect estimate: The true effect is likely to be substantially different from the estimate of effect | | | | | | |

**Explanations**

a. No details on allocation concealment, no protocol found, and no details on funding.

b. GERD score determined with a validated questionnaire (Ofman J, et al. Identifying patients with gastroesophageal reflux disease: Validation of a practical screening tool. Dig Dis Sci 2002; 47:1863-9), with no other information provided

c. Too few participants in the study

**Evidence Set 8: EGD with random biopsy versus chromoendoscopy**

***Evidence Set 8 - Results table***

| **Author Year**  **Study design** | **Outcome(s) description** | **Results** | | | **ROB assessment** | **Notes** |
| --- | --- | --- | --- | --- | --- | --- |
|  |  | **EGD with random biopsy** | | **Chromo-endoscopy** |  |  |
| **IMPORTANT OUTCOME 4: Incidence of EAC (by stage), BE, and low- and high-grade dysplasia** | | | | | | |
| **Confirmed BE (histologically)** | | | | | | |
| Wani 2014^58^  RCT | Patients were suspected of having BE if they had columnar lined epithelium that was reddish in color and velvety texture which could be distinguished easily from normal pale and glossy esophageal squamous epithelium. Specialized intestinal metaplasia was diagnosed if the intestinal goblet cells were present. | 5/33 (15.2%) | 4/23 (17.4%) | | Moderate | All patients (n=378) received EGD. Those with columnar lined epithelium were randomized to different biopsy methods (n=56). |
|  |  | p=0.55 | | |  |  |

***Evidence Set 8 - Forest Plot***

*Forest Plot 8.1: Incidence of confirmed BE*


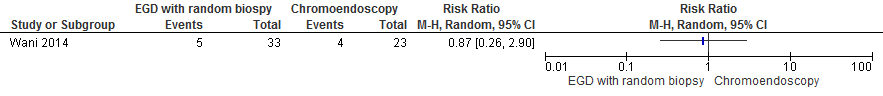


***Evidence Set 8 - GRADE evidence profile table***

| **Certainty assessment** | | | | | | | | **№ of patients** | | | **Effect** | | **Certainty** | **Importance** |
| --- | --- | --- | --- | --- | --- | --- | --- | --- | --- | --- | --- | --- | --- | --- |
| **№ of studies** | **Study design** | **Risk of bias** | **Inconsistency** | **Indirectness** | **Imprecision** | **Other considerations** | **EGD with random biopsy** | | **chromoendoscopy (methylene blue-directed biopsy)** | **Relative (95% CI)** | | **Absolute (95% CI)** |  |  |
| Incidence of confirmed BE | | | | | | | | | | | | | | |
| 1 ^a^ | randomised trials | serious ^b^ | not serious | serious ^c^ | serious ^d^ | none | 5/33 (15.2%) | | 4/23 (17.4%) | **RR 0.87** (0.26 to 2.90) | | **23 fewer per 1,000** (from 129 fewer to 330 more) | ⨁◯◯◯ VERY LOW | IMPORTANT |

**CI:** Confidence interval; **RR:** Risk ratio

**Explanations**

a. All participants were given EGD. If BE was suspected, patients were randomized to random biopsy or chromoendoscopy.

b. Method of allocation, and allocation concealment was not discussed. No protocol was found, there is no mention on how the study was funded, and there was no description of the baseline characteristics between the two study groups.

c. GERD was described as having “characteristics symptoms of GERD”.

d. Too few participants included.

***Evidence Set 8 - Summary of Findings Table***

| **EGD with random biopsy compared to chromoendoscopy (methylene blue-directed biopsy) for screening for EAC and precancerous conditions (BE and dysplasia)** | | | | | | |
| --- | --- | --- | --- | --- | --- | --- |
| **Setting**: Not reported  **Intervention**: EGD with random biopsy  **Comparison**: Chromoendoscopy (methylene blue-directed biopsy) | | | | | | |
| Outcomes | **Anticipated absolute effects^*^** (95% CI) | | Relative effect (95% CI) | № of participants  (studies) | Certainty of the evidence (GRADE) | Comments |
|  | **Risk with chromoendoscopy (methylene blue-directed biopsy)** | **Risk with EGD with random biopsy** |  |  |  |  |
| Incidence of confirmed BE | 174 per 1,000 | **151 per 1,000** (45 to 504) | **RR 0.87** (0.26 to 2.90) | 56 (1 RCT) ^a^ | ⨁◯◯◯ VERY LOW ^b,c,d^ |  |
| ***The risk in the intervention group** (and its 95% confidence interval) is based on the assumed risk in the comparison group and the **relative effect** of the intervention (and its 95% CI).   **CI:** Confidence interval; **RR:** Risk ratio | | | | | | |
| **GRADE Working Group grades of evidence** **High certainty:** We are very confident that the true effect lies close to that of the estimate of the effect **Moderate certainty:** We are moderately confident in the effect estimate: The true effect is likely to be close to the estimate of the effect, but there is a possibility that it is substantially different **Low certainty:** Our confidence in the effect estimate is limited: The true effect may be substantially different from the estimate of the effect **Very low certainty:** We have very little confidence in the effect estimate: The true effect is likely to be substantially different from the estimate of effect | | | | | | |

**Explanations**

a. All participants were given EGD. If BE was suspected, patients were randomized to random biopsy or chromoendoscopy.

b. Method of allocation, and allocation concealment was not discussed. No protocol was found, there is no mention on how the study was funded, and there was no description of the baseline characteristics between the two study groups.

c. GERD was described as having “characteristics symptoms of GERD”.

d. Too few participants included.

1. numbers estimated from Kaplan-Meier curve [↑](#footnote-ref-1)
2. numbers estimated from figure 2 [↑](#footnote-ref-2)
3. BE grade 1 (circumferential (C) or tongue (T) type, longest BE <1cm) [↑](#footnote-ref-3)
4. BE grade 2 (C type, 3cm > longest BE ≥1cm or T type, longest BE ≥1cm) [↑](#footnote-ref-4)
5. BE grade 3 (C type, shortest BE ≥3cm) [↑](#footnote-ref-5)
