# Supplementary material for: Screening for esophageal adenocarcinoma and precancerous conditions (dysplasia and Barrett’s esophagus) in patients with chronic gastroesophageal reflux disease with or without other risk factors: two systematic reviews and one overview of reviews to inform a guideline of the Canadian Task Force on Preventive Health Care (CTFPHC)
Source: Syst Rev. 2020 Jan 29;9:20. doi: 10.1186/s13643-020-1275-2 (PMC6990541; doi:10.1186/s13643-020-1275-2)
Supplement: Supplementary file 12 — Additional file 12: KQ3 evidence sets. [file 13643_2020_1275_MOESM12_ESM.docx]

**Additional file 12. KQ3 Results and GRADE domains tables**

**Contents**

Table 1. Outcomes and comparisons per systematic review (and primary study) 1

Evidence Set 1: Pharmacological therapy vs Placebo 3

*Evidence Set 1.1 Celecoxib vs Placebo: Results table* 3

*Evidence Set 1.1 Celecoxib vs Placebo: GRADE domains table* 4

Evidence Set 2: Pharmacological therapy vs Pharmacological therapy 5

*Evidence Set 2.1 Omeprazole vs H2RA: Results table* 5

*Evidence Set 2.1 Omeprazole vs H2RA: GRADE domains table* 6

Evidence Set 3: Chemical ablative technique combined with pharmacological therapy vs Pharmacological therapy alone 7

*Evidence Set 3.1 PDT + Omeprazole vs Omeprazole alone: Results table* 7

*Evidence Set 3.1 PDT + Omeprazole vs Omeprazole alone: GRADE domains table* 10

Evidence Set 4: Surgery combined with thermal ablative technique vs Surgery combined with surveillance 14

*Evidence Set 4.1 Anti-reflux surgery (Nissen Fundoplication) + APC vs Anti-reflux surgery (Nissen Fundoplication) + Surveillance (endoscopic): Results table* 14

*Evidence Set 4.1 Anti-reflux surgery (Nissen Fundoplication) + APC vs Anti-reflux surgery (Nissen Fundoplication) + Surveillance (endoscopic): GRADE domains table* 16

Evidence Set 5: Thermal ablative techniques combined with pharmacological therapy vs Pharmacological therapy 18

*Evidence Set 5.1 RFA + PPI vs PPI: Results table* 18

*Evidence Set 5.1 RFA + PPI vs PPI: GRADE domains table* 21

Evidence Set 6: Surgery vs Pharmacological therapy 24

*Evidence Set 6.1 Anti-reflux surgery vs H2 receptor antagonist/Omeprazole: Results table* 24

*Evidence Set 6.1 Anti-reflux surgery vs H2 receptor antagonist/Omeprazole: GRADE domains table* 26

Evidence Set 7: Chemical ablative technique vs Chemical ablative technique 28

*Evidence Set 7.1 PDT (5-ALA) vs PDT (Photofrin): Results table* 28

*Evidence Set 7.1 PDT (5-ALA) vs PDT (Photofrin): GRADE domains table* 29

*Evidence Set 7.2 PDT with different treatment parameters: Results table* 30

*Evidence Set 7.2 PDT with different treatment parameters: GRADE domains table* 31

Evidence Set 8: Thermal ablative technique vs Surveillance 32

*Evidence Set 8.1 RFA vs Surveillance (endoscopic): Results table* 32

*Evidence Set 8.1 RFA vs Surveillance (endoscopic): GRADE domains table* 34

Evidence Set 9: Thermal ablative technique combined with pharmacological therapy vs Thermal ablative technique combined with pharmacological therapy 36

*Evidence Set 9.1 APC + PPI vs MPEC + PPI: Results table* 36

*Evidence Set 9.1 APC + PPI vs MPEC +PPI: GRADE domains table* 37

*Evidence Set 9.2 MPEC + PPI vs APC + PPI: Results table* 38

*Evidence Set 9.2 MPEC vs APC: GRADE domains table* 39

Evidence Set 10: Thermal ablative technique vs Chemical ablative technique combined with pharmacological therapy 40

*Evidence Set 10.1 PDT vs APC + PPI: results table* 40

*Evidence Set 10.1 PDT vs APC + PPI: GRADE domains table* 44

Evidence Set 11: Mechanical ablative technique vs Thermal ablative technique 48

*Evidence Set 11.1 EMR vs RFA* 48

*Evidence Set 11.1 EMR vs RFA: GRADE domains table* 51

**Table 1. Outcomes and comparisons per systematic review (and primary study)**

|  | **Evidence Set 1.1** | **Evidence Set 2.1** | **Evidence Set 3.1** | **Evidence Set 4.1** | **Evidence Set 5.1** | **Evidence Set 6.1** | **Evidence Set 7.1** |
| --- | --- | --- | --- | --- | --- | --- | --- |
|  | **Celecoxib vs placebo** | **Omeprazole vs H2RA** | **PDT + Omeprazole vs Omeprazole** | **Anti-reflux surgery + APC vs Anti-reflux surgery +** **Surveillance** | **RFA + PPI vs PPI** | **Anti-reflux surgery vs Omeprazole / H2RA** | **PDT (ALA-5) vs PDT (Photofrin)** |
| **All-cause mortality** | Rees 2010^60^ (^61^) |  | Rees 2010^60^ (^66,67^) |  |  | Rees 2010^60^ (^76^) |  |
| **Progression to EAC** | Rees 2010^60^ (^61^) |  | Rees 2010^60^ (^67^)  Li 2008^69^ (^65^)  Fayter 2010^70^ (^65^) | Rees 2010^60^ (^71^) | Rees 2010^60^ (^75^)  Qumseya 2017^73^ (^75^) | Rees 2010^60^ (^76^)  Li 2008^69^ (^76^) |  |
| **Progression to HGD** |  |  |  | Li 2008^69^ (^71^) |  |  |  |
| **Progression to dysplasia** |  |  | Rees 2010^60^ (^66^) | Li 2008^69^ (^71^) | Rees 2010^60^ (^75^)  Qumseya 2017^73^ (^75^)  Pandey 2018^74^(^75^) | Rees 2010^60^ (^76^)  Li 2008^69^ (^76^) |  |
| **Eradication of neoplasia** |  |  |  |  |  |  |  |
| **Eradication of dysplasia** |  |  | Rees 2010^60^ (^66,67^)  Li 2008^69^ (^66,67^) |  |  | Rees 2010^60^ (^76^) |  |
| **Eradication of HGD** |  |  | Li 2008^69^ (^65^)  Fayter 2010^70^ (^65^)^b^ |  |  |  | Rees 2010^60^ (^76^)  Fayter 2010^70^ (^77^) |
| **Complete clearance of dysplasia** |  |  |  |  | Rees 2010^60^ (^75^)  Pandey 2018^74^ (^75^)^c^ |  |  |
| **Eradication of BE** |  |  | Rees 2010^60^ (^65^)  Li 2008^69^ (^65^) | Rees 2010^60^ (^71^)  Li 2008^69^ (^71^) | Rees 2010^60^ (^75^) | Rees 2010^60^ (^76^) |  |
| **Reduction/ regression in BE^a^** |  | Rees 2010^60^ (^62–64^)  Li 2008^69^ (^63^) | Rees 2010^60^ (^66^)  Li 2008^69^ (^66^)  Fayter 2010^70^ (^66^) |  |  |  |  |
| **Complete clearance of intestinal metaplasia** |  |  |  |  | Pandey 2018^74^ (^75^) |  |  |
| **Recurrence of EAC** |  |  |  |  |  |  |  |
| **Recurrence of intestinal metaplasia** |  |  |  |  |  |  |  |
| **Treatment failure (no ablation)** |  |  | De Souza 2014^68^ (^66,67^) | De Souza 2014^68^ (^72^) | De Souza 2014^68^ (^75^) |  |  |
| **Serious adverse reaction** |  |  |  |  |  |  |  |
| **Stricture formation** |  |  | Rees 2010^60^ (^67^)  Fayter 2010^70^ (^65^) |  | Rees 2010^6047^ (^75^) |  | Rees 2010^47^ (^68^) |
| **Bleeding** |  |  |  |  | Pandey 2018^74^ (^75^) |  |  |
| **Perforations** |  |  |  |  | Pandey 2018^74^ (^75^) |  |  |
| **Stenosis requiring treatment** |  |  |  |  |  |  |  |

APC: Argon Plasma Coagulation; BE: Barrett’s Esophagus; EAC: esophageal adenocarcinoma; H2RA: H2 Receptor Antagonists; PDT: Photodynamic Therapy; PPI: Proton Pump Inhibitor

a: could include reduction in length (cm), reduction in area (%) or regression; b: outcome evaluated was maintaining complete ablation; c: subset of patients

|  | **Evidence Set 7.2** | **Evidence Set 8.1** | **Evidence Set 9.1** | **Evidence Set 9.2** | **Evidence Set 10.1** | **Evidence Set 11.1** |
| --- | --- | --- | --- | --- | --- | --- |
|  | **PDT w/ different treatment parameters** | **RFA vs Surveillance** | **APC + PPI vs MPEC + PPI** | **MPEC + PPI vs**  **APC + PPI** | **PDT vs APC** | **EMR vs RFA** |
| **All-cause mortality** |  |  | Rees 2010^60^ (^82^) |  | Rees 2010^60^ (^86–88^) |  |
| **Progression to EAC** | Fayter 2010^70^ (^78,79^) | Qumseya 2017^73^ (^81^) |  |  | Almond 2014^84^ (^86,88,90,^) |  |
| **Progression to HGD** |  | Qumseya 2017^73^ (^81^)  Pandey 2018^74^ (^81^) |  |  | Almond 2014^84^ (^86,88^) |  |
| **Progression to dysplasia** |  |  |  |  |  |  |
| **Eradication of neoplasia** |  |  |  |  |  | Desai 2017^93^ (^95^) |
| **Eradication of dysplasia** |  | Pandey 2018^74^ (^81^) |  |  | Rees 2010^60^ (^88^)  Almond 2014^84^ (^86,88^)  Fayter 2010^70^ (^88^)  Li 2008^69^ (^88^) | Chadwick 2014^91^ (^95^) |
| **Eradication of BE^†^** |  |  |  |  | Rees 2010^60^ (^86–88^)  Fayter 2010^70^ (^87^) |  |
| **Complete ablation of BE** |  |  |  | Rees 2010^60^ (^82,83^)  Li 2008^69^ (^82,83^) | Li 2008^69^ (^85–87^)  Fayter 2010^70^ (^86^) |  |
| **Reduction/ regression in BE^a^** | Fayter 2010^70^ (^75^) |  |  |  | Rees 2010^60^ (^88^)  Li 2008^69^ (^88^)  Fayter 2010^70^ (^86,89^) |  |
| **Eradication of intestinal metaplasia** |  | Pandey 2018^74^ (^81^) |  |  | Almond 2014^84^ (^88^) | Desai 2017^93^ (^95^)  Chadwick 2014^91^ (^95^) |
| **Recurrence of EAC** |  |  |  |  |  | Fujii-Lau 2017^92^ (^95^) |
| **Recurrence of dysplasia** |  |  |  |  |  | Fujii-Lau 2017^92^ (^95^) |
| **Recurrence of IM** |  |  |  |  |  | Fujii-Lau 2017^92^ (^95^)  Desai 2017^93^ (^95^) |
| **Treatment failure (no ablation)** |  |  |  | De Souza 2014^68^ (^82,83^) | De Souza 2014^68^ (^86–88^) |  |
| **Stricture formation** | Fayter 2010^70^ (^78,80^) | Pandey 2018^74^ (^81^) | Rees 2010^60^ (^83^) |  | Rees 2010^60^ (^86–88^)  Almond 2014^84^ (^88^)  Fayter 2010^70^ (^87,88^) | Desai 2017^93^ (^95^) |
| **Bleeding** |  | Pandey 2018^74^ (^81^) |  |  |  | Chadwick 2014^91^ (^95^)  Desai 2017^93^ (^95^) |
| **Perforations** | Fayter 2010^70^ (^80^) | Pandey 2018^74^ (^81^) |  |  |  | Chadwick 2014^91^ (^95^)  Desai 2017^93^ (^95^) |
| **Stenosis requiring treatment** |  |  |  |  |  | Chadwick 2014^91^ (^95^) |

APC: Argon Plasma Coagulation; BE: Barrett’s Esophagus; EAC: esophageal adenocarcinoma; IM: Intestinal metaplasia; MPEC: Multipolar Electrocoagulation; PDT: Photodynamic Therapy; PPI: Proton Pump Inhibitor; RFA: Radiofrequency Ablation;

† eradication and complete ablation of BE are considered the same outcome, however a distinction has been made in Evidence Set 10.1 as the included studies and results differ between Rees 2010 and

**Evidence Set 1: Pharmacological therapy vs Placebo**

***Evidence Set 1.1 Celecoxib vs Placebo: Results table***

*Based on one primary study: Heath 2007, USA*^61^

| **1.1 Celecoxib vs Placebo** | | | | | | | | |
| --- | --- | --- | --- | --- | --- | --- | --- | --- |
| **Author Year** | **Outcome** | **Results: n/N** | | | **Effect estimate (95% CI)** | **Absolute**  **Risk Difference (ARD)** | **AMSTAR† & GRADE‡** | **Notes** |
|  |  | **Study, Country** | **Celecoxib** | **Placebo** |  |  |  |  |
| **All-cause mortality** | | | | | | | | |
| Rees 2010^60^ | All-cause mortality | Heath 2007^61^, USA | 3/49  See note | 3/51  See note | See note | See note | **AMSTAR:** Low  **Certainty**: Low | In the text two deaths are reported. We are unsure if the reported estimate from the forest plot belongs to Progression to EAC at one year (see next row). |
| **Progression to EAC** | | | | | | | | |
| Rees 2010^60^ | Progression to EAC at one year | Heath 2007^61^, USA | 3/49  (6.1%) | 3/51  (5.9%) | OR 1.04  (0.20, 5.44) | ARD with intervention: 2 more per 1,000 (from 46 fewer to 195 more);  Risk with control: 59 per 1,000 | **AMSTAR:** Low  **Certainty**: Very low | All six patients who progressed to EAC had a baseline diagnosis of HGD. |

**†** see Supplementary table 1 for further details on AMSTAR domain ratings

‡ see Evidence Set 1: GRADE domains table for further details on GRADE domain ratings

***Evidence Set 1.1 Celecoxib vs Placebo: GRADE domains table***

| **1.1 Celecoxib vs Placebo** | | | | | | | | |
| --- | --- | --- | --- | --- | --- | --- | --- | --- |
| **Review Studies** | **Outcome** | **Study Limitations** | **Indirectness** | **Inconsistency** | **Imprecision** | **Other considerations** | **Certainty** | **Importance** |
| Rees 2010^60^  Heath 2007^61^ | All-cause mortality | Serious  • some concerns due to assessments judged as unclear^1^ | No serious limitations  • it is unknown if patients had other GE conditions^2^ | No serious limitations  • one study | Serious  • small sample size (n=100)^3^ | No serious limitations  • SR did not assess for publication bias. Although small study, comprehensive search and included search for unpublished literature. | Low | Critical |
| Rees 2010^60^  Heath 2007^61^ | Progression to EAC at one-year | Serious  • some concerns due to assessments judged as unclear^1^ | No serious limitations  • it is unknown if patients had other GE conditions^2^ | No serious limitations  • one study | Very serious  • small sample size (n=100), wide CI including only six events^4^ | No serious limitations  • SR did not assess for publication bias. Although small studies, comprehensive search and included search for unpublished literature. | Very low | Critical |

1 Sequence generation, allocation concealment, and blinding all judged as unclear

2 The review does not report if the patients had other GE conditions, which was part of the exclusion criteria for this overview, but the effect on treatment is expected to be minimal

3 Based on few events/small study sample (sample size<rule of thumb of n=300 events)

4 Based on few events, CI for relative effects include both appreciable benefit and harm, and absolute CI reasonably includes appreciable benefit and harm

**Evidence Set 2: Pharmacological therapy vs Pharmacological therapy**

***Evidence Set 2.1 Omeprazole vs H2RA: Results table***

*Based on three primary studies: Caldwell 1996^61^; Peters 1999^62^; Weinstein 1996^63^*

| **2.1 Omeprazole vs Histamine Type 2 Receptor Antagonists (H2RA)** | | | | | | | | |
| --- | --- | --- | --- | --- | --- | --- | --- | --- |
| **Review Author Year** | **Outcome** | **Results: Mean (SD)** | | | **Effect estimate (95% CI)** | **Absolute**  **Risk Difference** | **AMSTAR† & GRADE‡** | **Notes** |
|  |  | **Study, Country** | **Omeprazole** | **H2RA** |  |  |  |  |
| **Reduction/regression of Barrett’s Esophagus (BE)** ϯ | | | | | | | | |
| Rees 2010^60^ | Reduction in length (cm) of BE at 12 months | Caldwell 1996^62^, NR | 0.7 (3.3) (n=10) | -1.7 (4.6)  (n=10) | Pooled MD -0.42  (-1.65, 0.82) | Mean difference 0.42 lower (1.65 lower to 0.82 higher);  Risk with control: The mean reduction in length (cm) ranged from -1.7 to 0.53 | **AMSTAR:** Low  **Certainty:** Very low | Weinstein and Peters compare different treatment regimens of Omeprazole to Ranitidine and Caldwell compares Omeprazole 20 mg once/day to Cimetidine^1^ 3x/day.  Weinstein: Omeprazole^2^ 2x/day for one year followed by 40 mg 1/day for one year compared to Ranitidine 150 mg 2/day for two years.  Peters: Omeprazole^3^ 2x/day compared to Ranitidine 150 mg 2x/day for two years. |
|  |  | Peters 1999^63^, NR | -5.6 (10.86)  (n=26) | 0.53 (13.86)  (n=27) |  |  |  |  |
|  |  | Weinstein 1996^64^, NR | -0.4 (3.32)  (n=50) | 0.2 (3.16)  (n=40) |  |  |  |  |
| Rees 2010^60^ | Reduction in length (cm) of BE at 12 months (subgroup analysis including higher doses of omeprazole) | Peters 1999^63^, NR | -5.6 (10.86)  (n=26) | 0.53 (13.86)  (n=27) | Pooled MD -0.81 (-2.13, 0.50) | Mean difference 0.81 lower (2.13 lower to 0.5 higher);  Risk with control: The mean reduction in length (cm) ranged from 0.2 to 0.53 | **AMSTAR:** Low  **Certainty:** Very low |  |
|  |  | Weinstein 1996^64^, NR | -0.4 (3.32)  (n=50) | 0.2 (3.16)  (n=40) |  |  |  |  |
| Rees 2010^60^ | Reduction in area (%) of BE at 12 months | Peters 1999^63^, NR | 5.2 (11.22)  (n=26) | 0.8 (11.22)  (n=27) | **Pooled MD** **4.06 (0.08, 8.04)** | Mean difference 4.06% higher (0.08% higher to 8.04% higher);  Risk with control: The mean reduction in area (%) ranged from 0.5 to 0.8. | **AMSTAR**: Low  **Certainty:** Very low to low | Li 2008 reports the area of regression for Peters 1999; concordance for these data. |
|  |  | Weinstein 1996^64^, NR | 4.3 (12.02)  (n=50) | 0.5 (13.28)  (n=40) |  |  |  |  |

Bolded effect estimates refer to statistically significant results. **†** see Supplementary table 1 for further details on AMSTAR domain ratings. ‡ see Evidence Set 2: GRADE domains table for further details on GRADE domain ratings. Ϯ post-hoc outcome

1 discrepant values reported: 400 mg in one instance and 300 mg in another

2 discrepant values reported: 40 mg in one instance and 80 mg in another

3 discrepant values reported: 40 mg in one instance and 20 mg in another

***Evidence Set 2.1 Omeprazole vs H2RA: GRADE domains table***

| **2.1 Omeprazole vs Histamine Type 2 Receptor Antagonists (H2RA)** | | | | | | | | | |
| --- | --- | --- | --- | --- | --- | --- | --- | --- | --- |
| **Review / Studies** | **Comparison** | **Outcome** | **Study Limitations** | **Indirectness** | **Inconsistency** | **Imprecision** | **Other considerations** | **Certainty** | **Importance** |
| Rees 2010^60^  Caldwell 1996^62^ (abstract)  Peters 1999^63^  Weinstein 1996^64^ | Omeprazole vs histamine type 2 receptor antagonists (ranitidine or cimetidine) | Reduction in length (cm) of BE at 12 months | Serious  • some concerns due to assessments judged as unclear^1^ | Unclear  • country of conduct not reported which may affect the delivery of care^3^  • it is unknown if patients had other GE conditions^4^ | Serious  • substantial heterogeneity (I^2^=63%, p=0.07) unaccounted for, little overlap of Cis, variation in direction of effect estimates | Serious  • small sample size (n=143)^5^ | No serious limitations  • SR did not assess for publication bias. Although small studies, comprehensive search and included search for unpublished literature. | Very Low | Critical; identified post hoc |
| Rees 2010^60^  Peters 1999^63^  Weinstein 1996^64^ | Omeprazole (40 mg) vs histamine type 2 receptor antagonists (ranitidine)  Subgroup including only higher dose omeprazole | Reduction in length (cm) of BE at 12 months | Serious  • some concerns due to assessments judged as unclear^2^ | Unclear  • country of conduct not reported which may affect the delivery of care^3^  • it is unknown if patients had other GE conditions^4^ | Serious  • moderate heterogeneity (I^2^=60%, p=0.11) unaccounted for, variations in magnitude of effect estimates | Serious  • small sample size (n=143)^5^ | No serious limitations  • SR did not assess for publication bias. Although small studies, comprehensive search and included search for unpublished literature. | Very Low | Critical; identified post hoc |
| Rees 2010^60^  Peters 1999^63^  Weinstein 1996^64^ | Omeprazole vs histamine type 2 receptor antagonists (ranitidine) | Reduction in area (%) of BE at 12 months | Serious  • some concerns due to assessments judged as unclear^2^ | Unclear  • country of conduct not reported which may affect the delivery of care^3^  • it is unknown if patients had other GE conditions^4^ | No serious limitations | Serious  • small sample sizes (n=143)^5^ | No serious limitations  • SR did not assess for publication bias. Although small studies, comprehensive search and included search for unpublished literature. | Very low to low | Critical; identified post hoc |

1 Majority of evidence coming from 2/3 studies (85% of evidence), which were unclear for allocation concealment and whether attrition-related concerns exist; one of the studies (>50% of evidence) additionally unclear for randomization sequence, any differences in care provided or sought, and blinding of outcomes assessors. Assessments from remaining study was not included, as was published in abstract form and accounted for 15% of pooled evidence.

2 Unclear for allocation concealment and whether attrition-related concerns exist in both studies; one of the studies (>60% of evidence) additionally unclear for randomization sequence, any differences in care provided or sought, and blinding of outcomes assessors.

3 Country of conduct may affect the delivery of care in light of potential contextual influences such as a change in the accessibility to the regimens

4 The review does not report if the patients had other GE conditions, which was part of the exclusion criteria for this overview, but the effect on treatment is expected to be minimal

5 Based on low study sample (sample size<rule of thumb of n=400 people)

**Evidence Set 3: Chemical ablative technique combined with pharmacological therapy vs Pharmacological therapy alone**

***Evidence Set 3.1 PDT + Omeprazole vs Omeprazole alone: Results table***

*Based on three primary studies (one an update of another): Ackroyd 2000*^66^*; Overholt 2005*^67^*; Overholt 2007 (update of 2005)*^65^

| **3.1** **Photodynamic therapy (PDT) + Omeprazole vs Omeprazole alone** | | | | | | | | | | | |
| --- | --- | --- | --- | --- | --- | --- | --- | --- | --- | --- | --- |
| **Author Year** | **Outcome** | **Results: n/N (%); Mean (SD)** | | | | | **Effect estimate (95%CI)** | | **Absolute Risk Difference (ARD)** | **AMSTAR† & GRADE‡** | **Notes** |
|  |  | **Study, Country** | **PDT + Omep.** | | **Omep.** | |  |  |  |  |  |
| **All-cause mortality** | | | | | | | | | | | |
| Rees 2010^60^ | All-cause mortality | Overholt 2005^67^, NR^§^ | 2/138  (1.4%) | | 1/70  (1.4%) | | OR 1.01  (0.09, 11.39) | | ARD with intervention: 0 fewer per 1,000 (from 13 fewer to 127 more);  Risk with control: 14 per 1,000 | **AMSTAR:** Low  **Certainty:** Very low | Overholt 2005 used PDT with 5-ALA and Ackroyd used PDT with porfimer sodium |
|  |  | Ackroyd 2000^66^, NR | 0/18 | | 0/18 | | OR not estimable | | n/a |  |  |
| **Progression to EAC** | | | | | | | | | | | |
| Rees 2010^60^ | Progression to cancer at latest possible time point (up to 2 years) | Overholt 2005^67^, NR^§^ | | 18/138  (13%) | | 20/70  (29%) | **OR 0.38**  **(0.18, 0.77)** | | ARD with intervention: 154 fewer per 1,000 (from 50 fewer to 219 fewer);  Risk with control: 286 per 1,000 | **AMSTAR:** Low  **Certainty:** Very low to low | Li 2008 reports the same results at 2 years. |
| Li 2008^69^ | Progression to cancer (at 5 years) | Overholt 2007^65^, NR^§^ | | 21/138 (15%) | | 20/70 (29%) | NR  ****RR 0.53 (0.31, 0.91)** | | ARD with intervention: 134 fewer per 1,000 (from 26 fewer to 197 fewer);  Risk with control: 286 per 1,000 | **AMSTAR:** Critically low  **Certainty**: Very low to low | Fayter 2010 reports that after 5 years of follow-up, the rate of patients who progressed to cancer in PDT+Omeprazole was significantly lower than in Omeprazole alone (*p* = 0.027). |
| **Progression from non-dysplastic BE to BE with dysplasia** | | | | | | | | | | | |
| Rees 2010^60^ | Progression from intestinal metaplasia to dysplasia | Ackroyd 2000^66^, NR | | 0/18 | | 12/18  (67%) | **OR 0.01**  **(0.00, 0.27)** | | ARD with intervention: 647 fewer per 1,000 (from --- to 316 fewer);  Risk with control: 667 per 1,000 | **AMSTAR:** Low  **Certainty**: Very low | Same results reported in Li 2008. |
| **Eradication** **of dysplasia** ϯ | | | | | | | | | | | |
| Rees 2010^60^ | Complete eradication of dysplasia at two-years | Ackroyd 2000^66^, NR | | *6/18  (33%) | | ***0/18** | **Pooled OR 9.13 (4.42, 18.86)** | | ARD with intervention: 426 more per 1,000 (from 248 more to 594 more);  Risk with control: 114 per 1,000 | **AMSTAR:** Low  **Certainty:** Very low to low | Ackroyd used PDT with 5-ALA and Overholt 2005 used PDT with porfimer sodium. |
|  |  | Overholt 2005^67^, NR^§^ | | 81/138  (59%) | | 10/70  (14%) |  |  |  |  |  |
| Li 2008^69^ | Dysplasia eradication | Ackroyd 2000^66^, NR | | *18/18 (100%) | | *6/18 (33.3%) | ****RR 2.85**  **(1.52, 5.33)** | | ARD with intervention: 617 more per 1,000 (from 173 more to 1,000 more);  Risk with control: 333 per, 1,000 | **AMSTAR:** Critically low  **Certainty**: Very low to low | Although data differences with what was reported in Rees 2010 for Ackroyd 2000, overall concordance between reviews. |
|  |  | Overholt 2005^67^, NR^§^ | | 81/138 (59%) | | 10/70 (14.3%) | ****RR 4.11**  **(2.28, 7.42)** | | ARD with intervention: 444 more per 1,000 (from 183 more to 917 more);  Risk with control: 143 per 1,000 |  |  |
| Li 2008^69^ | Eradication of High Grade Dysplasia | Overholt 2005^67^, NR^§^ | | 106/138 (77%) | | 27/70 (39%) | ****RR 1.99**  **(1.46, 2.71)** | | ARD with intervention: 382 more per 1,000 (from 177 more to 660 more);  Risk with control: 386 per 1,000 | **AMSTAR:** Critically low  **Certainty**: Very low to low | Fayter 2010 reported that the probability of maintaining complete ablation of HGD was 48% in PDT + Omep. compared to 4% in Omep. alone (p<0.0001) at the end of 5-year follow up period.  Same denominator as Li 2008 eradication of dysplasia, but unclear why there were more in the numerator. |
| **Eradication of Barrett’s Esophagus (BE)** ϯ | | | | | | | | | | | |
| Rees 2010^60^ | Complete eradication of BE over the course of the study (5 years) | Overholt 2007^65^, Overholt 2005^67^*,* NR^§^ | | 72/138  (52%) | | 5/70  (7.1%) | **OR 14.18**  **(5.38, 37.37)** | | ARD with intervention: 450 more per 1,000 (from 221 more to 670 more);  Risk with control: 71 per 1,000 | **AMSTAR:** Low  **Certainty**: Very low to low | Reported as complete ablation in Li 2008 but as complete eradication in Rees 2010. Results are concordant. |
| **Reduction/regression of Barrett’s Esophagus (BE)** ϯ | | | | | | | | | | | |
| Rees 2010^60^ | Reduction in length (cm) of BE at 12 months | Ackroyd 2000^66^, NR | | 1.11 (1.23)  (n=18) | | 0.11 (0.32)  (n=18) | **MD 1.00**  **(0.41, 1.59)** | | MD 1 cm higher (0.41 cm higher to 1.59 cm higher);  Risk with control: mean was 0.11 cm | **AMSTAR:** Low  **Certainty**: Very low |  |
| Rees 2010^60^ | Reduction in area (%) of BE at 12 months | Ackroyd 2000^66^, NR | | 31.11 (20.25)  (n=18) | | 1.11 (3.23)  (n=18) | **MD 30.00**  **(20.53, 39.47)** | | MD 30% higher (20.53% higher to 39.47% higher);  Risk with control: mean 1.11% | **AMSTAR:** Low  **Certainty**: Very low |  |
| Li 2008^69^ | Area of regression of BE | Ackroyd 2000^66^, NR | | Median (range): 30%  (0-60%) | | Median (range): 0%  (0-10%) | NR | | Not estimable | **AMSTAR:** Critically low  **Certainty:** Very low | SR did not refer what specific type of PPI was evaluated, however it includes the same primary studies as Rees 2010. |
| Fayter  2010^70^ | Evidence of regression | Ackroyd 2000^66^, NR | | 89% | | 11% | NR | | Not estimable | **AMSTAR:** Critically low  **Certainty:** Very low | Reduction is reported as 30% vs 0%, which corroborates with the median reported in Li 2008. |
| **Treatment failure** ϯ | | | | | | | | | | | |
| De Souza 2014^68^ | Treatment Failure (no ablation of BE) | Ackroyd 2000^66^, NR | | 4/18  (22%) | | 18/18  (100%) | **Pooled RD**  **-0.49**  **(-0.58, -0.39),**  I^2^ = 89%  ****RR 0.49**  **(0.41 to 0.59)** | | ARD with intervention: 487 fewer per 1,000 (391 fewer to 563 fewer)  Risk with control: 955 per 1,000 | **AMSTAR:** Critically low  **Certainty:** Very low | SR did not refer what specific type of PPI was evaluated, however it includes the same primary studies as Rees 2010.  Analysis uses fixed effects. Random effects would be RR 0.40 (0.17 to 0.92), I^2^=77%. |
|  |  | Overholt 2005^67^, NR^§^ | | 71/138  (51%) | | 66/70  (94%) |  |  |  |  |  |
| **Stricture formation** ϯ | | | | | | | | | | | |
| Rees 2010^60^ | Stricture formation | Overholt 2005^67^, NR^§^ | | 49/138  (36%) | | 0/70 | **OR 77.98**  **(4.73, 1286.52)** | | Not estimable | **AMSTAR:** Low  **Certainty**: Very low to low | Fayter 2010 reported (from Overholt 2007) that 36% of PDT patients developed oesophageal strictures, but that 94% of those with strictures were stricture free in the initial phase of the trial. |
| **†** see Supplementary table 1 for further details on AMSTAR domain ratings  ‡ see Evidence Set 3: GRADE domains table below for further details on GRADE domain ratings  ϯ post-hoc outcome  *discrepant data  **the effect estimate was not reported in the original SR but calculated by the overview team | | | | | | | | ^§^ Overholt 2005 has been an international trial but it is not known which countries were involved. Overholt 2007 was a 5 year follow up of the same patients.  Bolded effect estimates refer to statistically significant results.  Underlined first author name, publication year refers to a unique study included in more than one review. | | | |

***Evidence Set 3.1 PDT + Omeprazole vs Omeprazole alone: GRADE domains table***

| **3.1 Photodynamic therapy (PDT) + Omeprazole vs Omeprazole alone** | | | | | | | | |
| --- | --- | --- | --- | --- | --- | --- | --- | --- |
| **Review Studies** | **Outcome** | **Study Limitations** | **Indirectness** | **Inconsistency** | **Imprecision** | **Other considerations** | **Certainty** | **Importance** |
| Rees 2010^60^  Overholt 2005^67^  Ackroyd 2000^66^ | All-cause mortality | Serious  • some concerns due to assessments judged as unclear^1^ | Unclear  • country of conduct not reported which may affect the delivery of care^12^  • it is unknown if patients had other GE conditions^13^ | No serious limitations  • CI only 1 study; few events across studies | Very serious  • small sample sizes (n=244), wide CI including only three events in total^14^ | No serious limitations  • SR did not assess for publication bias. Although small studies, comprehensive search and included search for unpublished literature. | Very low | Critical |
| Rees 2010^60^    Overholt 2005^67^ | Progression to cancer at latest possible time point (up to 2 years) | Serious  • some concerns due to assessments judged as unclear^2^ | Unclear  • country of conduct not reported which may affect the delivery of care^12^  • it is unknown if patients had other GE conditions^13^ | No serious limitations  • one study | Serious  • small sample size (n=208)^15^ | No serious limitations  • SR did not assess for publication bias. Although small studies, comprehensive search and included search for unpublished literature. | Very low to low | Critical |
| Li 2008^69^  Overholt 2007^65^* | Progression to cancer (at 5 years) | Serious  • some concerns due to assessments judged as unclear^3^ | Unclear  • country of conduct not reported which may affect the delivery of care^12^  • it is unknown if patients had other GE conditions^13^ | No serious limitations  • one study | Serious  • small sample size (n=208)^15^ | No serious limitations  • SR did not assess for publication bias. Although small studies, comprehensive search and included search for unpublished literature. | Very low to low | Critical |
| Rees 2010^60^  Ackroyd 2000^66^ | Progression from intestinal metaplasia to dysplasia | Very serious  • some concerns due to assessments judged as unclear and high risk^4^ | Unclear  • country of conduct not reported which may affect the delivery of care^12^  • it is unknown if patients had other GE conditions^13^ | No serious limitations  • one study | Serious  • small sample size (n=36)^15^ | No serious limitations  • SR did not assess for publication bias. Although small studies, comprehensive search and included search for unpublished literature. | Very low | Critical |
| Rees 2010^60^  Ackroyd 2000^66^  Overholt 2005^67^ | Complete eradication of dysplasia at two-years | Serious  • some concerns due to assessments judged as unclear^5^ | Unclear  • country of conduct not reported which may affect the delivery of care^12^  • it is unknown if patients had other GE conditions^13^ | No serious limitations | Serious  • small sample size (n=244)^15^ | No serious limitations  • SR did not assess for publication bias. Although small studies, comprehensive search and included search for unpublished literature. | Very low to low | Critical; identified post hoc |
| Li 2008^69^  Overholt 2005^67^  Ackroyd 2000^66^ | Dysplasia eradication | Serious  • some concerns due to assessments judged as unclear^6^ | Unclear  • country of conduct not reported which may affect the delivery of care^12^  • it is unknown if patients had other GE conditions^13^ | No serious limitations | Serious  • small sample size (n=244)^15^ | No serious limitations  • SR did not assess for publication bias. Although small studies, comprehensive search and included search for unpublished literature. | Very low to low | Critical; identified post hoc |
| Li 2008^69^  Overholt 2005^67^ | Eradication of high-grade dysplasia | Serious  • some concerns due to assessments judged as unclear^7^ | Unclear  • country of conduct not reported which may affect the delivery of care^12^  • it is unknown if patients had other GE conditions^13^ | No serious limitations  • one study | Serious  • small sample size (n=208)^15^ | No serious limitations  • SR did not assess for publication bias. Although small studies, comprehensive search and included search for unpublished literature. | Very low to low | Critical; identified post hoc |
| Rees 2010^60^  Overholt 2007^65^  (update from Overholt 2005^67^*)* | Complete eradication of BE over the course of the study (5 years) | Serious  • some concerns due to assessments judged as unclear^8^ | Unclear  • country of conduct not reported which may affect the delivery of care^12^  • it is unknown if patients had other GE conditions^13^ | No serious limitations  • one study | Serious  • small sample sizes (n=208)^15^ | No serious limitations  • SR did not assess for publication bias. Although small studies, comprehensive search and included search for unpublished literature. | Very low to low | Critical; identified post hoc |
| Rees 2010^60^  Ackroyd 2000^66^ | Reduction in length (cm) of BE at 12 months | Very serious  • some concerns due to assessments judged as unclear and high-risk^4^ | Unclear  • country of conduct not reported which may affect the delivery of care^12^  • it is unknown if patients had other GE conditions^13^ | No serious limitations  • one study | Serious  • small sample size (n=36)^16^ | No serious limitations  • SR did not assess for publication bias. Although small studies, comprehensive search and included search for unpublished literature. | Very low | Critical; identified post hoc |
| Rees 2010^60^  Ackroyd 2000^66^ | Reduction in area (%) of BE at 12 months | Very serious  • some concerns due to assessments judged as unclear and high-risk^4^ | Unclear  • country of conduct not reported which may affect the delivery of care^12^  • it is unknown if patients had other GE conditions^13^ | No serious limitations  • one study | Serious  • small sample size (n=36)^16^ | No serious limitations  • SR did not assess for publication bias. Although small studies, comprehensive search and included search for unpublished literature. | Very low | Critical; identified post hoc |
| Li 2008^69^  Ackroyd 2000^66^ | Area of regression of BE | Very serious  • some concerns due to assessments judged as unclear and high-risk^9^ | Unclear  • country of conduct not reported which may affect the delivery of care^12^  • it is unknown if patients had other GE conditions^13^ | No serious limitations  • one study | Serious  • small sample size (n=36)^17^ | Serious  • SR did not assess for publication bias, and the review did not perform comprehensive or grey lit searches | Very low | Critical; identified post hoc |
| Fayter 2010^70^  Ackroyd 2000^66^ | Evidence of regression | Very serious  • some concerns due to assessments judged as unclear and high-risk^10^ | Unclear  • country of conduct not reported which may affect the delivery of care^12^  • it is unknown if patients had other GE conditions^13^ | No serious limitations  • one study; no quantitative sufficient data | Serious  • small sample size based on information from Rees 2010 (n=36)^18^ | No serious limitations  • SR did not assess for publication bias. Although small studies, comprehensive search and included search for unpublished literature. | Very low | Critical |
| De Souza 2014^68^  Overholt 2005^67^  Ackroyd 2000^66^ | Treatment failure | Serious  • some concerns due to assessments judged as unclear^11^ | Unclear  • country of conduct not reported which may affect the delivery of care^12^  • it is unknown if patients had other GE conditions^13^ | Serious  • considerable heterogeneity (I^2^=89%, p=0.003) | Serious  • small sample size (n=244)^15^ | Serious  • comprehensive search not undertaken and uncertain about grey literature search | Very low | Critical; identified post hoc |
| Rees 2010^60^  Overholt 2005^67^ | Stricture formation | Serious  • some concerns due to assessments judged as unclear^2^ | Unclear  • country of conduct not reported which may affect the delivery of care^12^  • it is unknown if patients had other GE conditions^13^ | No serious limitations  • one study | Serious  • small sample size (n=208)^15^ | No serious limitations  • SR did not assess for publication bias. Although small studies, comprehensive search and included search for unpublished literature. | Very low to low | Critical; identified post hoc |

1 Both studies were unclear in relation to adequate sequence generation and attrition. One study (85% of evidence) was additionally unclear for allocation concealment and blinding in relation to care provided or sought. (Rees 2010 assessments)

2 Unclear in relation to whether adequate sequence generation, concealment used, systematic differences in the care provided (providers) or sought (patients), blinding of outcome assessors, and if attrition-related concerns. (Rees 2010 assessments)

3 Review authors (Li 2008) used Jadad tool indicating that randomization methods and allocation concealment unclear and no blinding took place, which is likely if the same and additional domains were assessed as unclear by the review authors (Rees 2010) of the 2005 version of this study. Authors did not make Jadad assessments specific to this outcome, but is reasonable to infer there may be concerns as per the assessments for the 2005 version.

4 Review authors state at unclear risk of bias in relation to sequence generation and attrition and at high risk from outcomes in this study not being clearly outlined. Given description of concealment, this is likely also unclear. Presumably, the latter refers to inadequate description or definition of outcomes. (Rees 2010 assessments)

5 Both studies were unclear in relation to adequate sequence generation and attrition. One study (85% of evidence) was additionally unclear for allocation concealment and blinding in relation to care provided or sought; the other study was deemed at high risk due to selective reporting (outcomes not clearly outlined) but contributed a minority of the evidence. (Rees 2010 assessments)

6 Assessments for both studies (Li 2008 review) made using Jadad tool, thus at least unclear from sequence generation and attrition. This information is corroborated by the assessments made in the Rees 2010 review, with an additional unclear assessment for allocation concealment. May be reasonable to assume that unclear judgements made for blinding (performance and detection bias) in the other review (Rees 2010) would have been deemed the same for this outcome.

7 Assessments (Li 2008 review) made using Jadad tool, thus at least unclear from sequence generation, concealment, and attrition. May be reasonable to assume that unclear judgements made for blinding (performance and detection bias) for other outcomes in the study (Rees 2010 review) would pertain to this outcome.

8 Using assessments reported in two reviews (Li 2008 and Rees 2010), the risk of bias related to sequence generation, concealment, care provided or sought, assessment of outcomes, and attrition is unclear.

9 Assessments (Li 2008 review) made using Jadad tool, thus at least unclear from sequence generation and concealment. May be reasonable to assume assessments made for other outcomes in the Rees 2010 review would pertain to this outcome: unclear attrition, high risk of selective reporting from outcomes not being clearly outlined in the study.

10 Review authors (Fayter 2010) provide information for all studies as an aggregate. From Rees 2010, the study is unclear for sequence generation and likely also for concealment as per description. It is likely that if unclear attrition and high risk for selective reporting for other outcomes, these would pertain to this outcome.

11 Assessments made in De Souza 2014 review not adequately detailed. Using assessments made in Rees 2010, both studies were unclear for sequence generation. One study (85% of evidence) was additionally unclear for allocation concealment; may be reasonable to assume that unclear judgements made for blinding (performance and detection bias) for other outcomes in the study would pertain to this outcome.

12 Country of conduct may affect the delivery of care in light of potential contextual influences such as a change in the accessibility to the regimens (for pharmacological treatment), and differences in training or equipment (procedural treatment).

13 The reviews do not report if the patients had other GE conditions, which was part of the exclusion criteria for this overview, but the effect on treatment is expected to be minimal

14 Based on few events, CI for relative effects include both appreciable benefit and harm, and absolute CI reasonably includes appreciable benefit and harm.

15 Based on few events/ small study sample (sample size<rule of thumb of n=300 events)

16 Based on small study sample (less than rule of thumb of n=400 people)

17 Likely low study sample based on other outcomes for same study (sample size<rule of thumb of 400 people). Ranges overlap between groups, but extent of dispersion difficult to compare.

18 As likely low study sample based on other outcomes for same study (sample size<rule of thumb of n=400 people).

* Overholt 2005 is an international trial and Overholt 2007 presents the five-year follow up of the patients.

**Evidence Set 4: Surgery combined with thermal ablative technique vs Surgery combined with surveillance**

***Evidence Set 4.1 Anti-reflux surgery (Nissen Fundoplication) + APC vs Anti-reflux surgery (Nissen Fundoplication) + Surveillance (endoscopic): Results table***

*Based on two primary studies: Bright 2007 (update of Ackroyd 2004)*^71^*; Ackroyd 2004*^72^

| **4.1 Anti-reflux surgery (Nissen fundoplication) + Argon plasma coagulation (APC)**  **vs Anti-reflux surgery (Nissen fundoplication) + Surveillance (endoscopic)** | | | | | | | | |
| --- | --- | --- | --- | --- | --- | --- | --- | --- |
| **Author Year** | **Outcome** | **Results: n/N (%)** | | | **Effect estimate (95% CI)** | **Absolute**  **Risk Difference (ARD)** | **AMSTAR† & GRADE‡** | **Notes** |
|  |  | **Study, Country** | **Anti-reflux surgery + APC** | **Anti-reflux surgery + Surv.** |  |  |  |  |
| **Progression to EAC** | | | | | | | | |
| Rees 2010^60^ | Progression to EAC | Bright 2007^71^,NR | NR | NR | NR | Not estimable | **AMSTAR**: Low  **Certainty**: Very low | The review reports qualitatively that “none of the patients progressed  to adenocarcinoma”. |
| **Progression from low- to high-grade dysplasia** | | | | | | | | |
| Li 2008^69^ | Progression to HGD | Bright 2007^71^, NR | 0/20 (0%) | 2/20 (10%) | NR  **RR 0.20  (0.01, 3.92) | ARD with intervention: 80 fewer per 1,000 (from 99 fewer to 292 more);  Risk with control: 100 per 1,000 | **AMSTAR:** Critically low  **Certainty:** Very low |  |
| **Progression from IM to dysplasia** | | | | | | | | |
| Rees 2010^60^ | Progression to dysplasia at 5 years | Bright 2007^71^, NR | 0/19 | 2/20  (10%) | **RR 0.21  (0.01, 4.11) | ARD with intervention: 79 fewer per 1,000 (from 99 fewer to 311 more);  Risk with control: 100 per 1,000 | **AMSTAR**: Low  **Certainty**: Very low | Bright 2007 is an update of Ackroyd 2004 (data not presented). |
| **Eradication of Barrett’s Esophagus (BE)** | | | | | | | | |
| Rees 2010^60^ | Complete eradication of BE at 12 months | Bright 2007^71^, NR | 14/20  (70%) | 0/20 | **OR 91.46**  **(4.77, 1754.50)** | Not estimable | **AMSTAR**: Low  **Certainty**: Very low | The data presented in the forest plot differs from that in the text (APC: 11/19 vs Surv: 3/20). Although bannered as 12 months follow-up in the forest plot, data may represent five years, based on study description. |
| Li 2008^69^ | Complete ablation (among those with histological change) | Bright 2007^71^, NR | 8/20 (40%) | 3/20 (15%) | **RR 2.67  (0.82, 8.62) | ARD with intervention: 251 more per 1,000 (from 27 fewer to 1,000 more);  Risk with control: 150 per 1,000 | **AMSTAR:** Critically low  **Certainty:** Very low | Bright 2007 is an update of Ackroyd 2004 (data not presented). |
| **Treatment failure** ϯ | | | | | | | | |
| De Souza 2014^68^ | Treatment failure (no ablation of BE) at one year | Ackroyd 2004^72^, NR | 6/19  (32%) | 10/20  (50%) | ARR was not reported but only 95% CI (-0.119, 0.487), p>0.05  ** RR 0.63 (0.29,1.40) | ARD with intervention: 185 fewer per 1,000 (from 355 fewer to 200 more);  Risk with control:500 per 1,000 | **AMSTAR:** Critically low  **Certainty:** Very low |  |

**†** see Supplementary table 1 for further details on AMSTAR domain ratings

‡ see Evidence Set 4.1: GRADE domains table for further details on GRADE domain ratings

ϯ post-hoc outcome

**the effect estimate was not reported in the original SR but calculated by the overview team

*Note*: Comparison in Rees 2010 and Li 2008 is labelled as APC vs surveillance; however, in De Souza 2014 as APC vs PPI. The assumption has been made that as it refers to the same study and has the same study group sizes, that it is the same comparison.

It is assumed that surgery [(Anti-reflux surgery (Nissen Fundoplication)] was administered in the patients before randomization into APC vs. surveillance.

Bolded effect estimates refer to statistically significant results.

Underlined first author name, publication year refers to a unique study included in more than one review.

***Evidence Set 4.1 Anti-reflux surgery (Nissen Fundoplication) + APC vs Anti-reflux surgery (Nissen Fundoplication) + Surveillance (endoscopic): GRADE domains table***

| **4.1 Anti-reflux surgery (Nissen Fundoplication) + Argon plasma coagulation (APC)**  **vs Anti-reflux surgery (Nissen Fundoplication) + Surveillance (endoscopic)** | | | | | | | | | |
| --- | --- | --- | --- | --- | --- | --- | --- | --- | --- |
| **Review Studies** | **Outcome** | **Study Limitations** | **Indirectness** | **Inconsistency** | **Imprecision** | **Other considerations** | **Certainty** | **Importance** |  |
| Rees 2010^60^  Bright 2007^71^ | Progression to EAC | Very serious  • some concerns due to assessments judged as unclear and high-risk^1^ | Unclear  • country of conduct not reported which may affect the delivery of care^4^  • it is unknown if patients had other GE conditions^5^ | No serious limitation  • one study | Serious  • small sample size (n=40)^6^ | No serious limitations  • SR did not assess for publication bias. Although small studies, comprehensive search and included search for unpublished literature. | Very low | Critical |  |
| Li 2008^69^  Bright 2007^71^ | Progression to HGD | Very serious  • some concerns due to assessments judged as unclear and high-risk^2^ | Unclear  • country of conduct not reported which may affect the delivery of care^4^  • it is unknown if patients had other GE conditions^5^ | No serious limitations  • one study | Very serious  • few events, small sample size (n=40)^7^ | Serious  • SR did not assess for publication bias, and the review did not perform comprehensive or grey lit searches | Very low | Critical |  |
| Rees 2010^60^  Bright 2007^71^ | Progression from IM to dysplasia | Very serious  • some concerns due to assessments judged as unclear and high-risk^1^ | Unclear  • country of conduct not reported which may affect the delivery of care^4^  • it is unknown if patients had other GE conditions^5^ | No serious limitations  • one study | Very serious  • sparse events, small sample size (n=40)^7^ | No serious limitations  • SR did not assess for publication bias. Although small studies, comprehensive search and included search for unpublished literature | Very low | Critical |  |
| Rees 2010^60^  Bright 2007^71^ | Complete eradication of BE at 12 months | Very serious  • some concerns due to assessments judged as unclear and high-risk^1^ | Unclear  • country of conduct not reported which may affect the delivery of care^4^  • it is unknown if patients had other GE conditions^5^ | No serious limitations  • one study | Serious  • small sample sizes (n=40)^8^ | No serious limitations  • SR did not assess for publication bias. Although small studies, comprehensive search and included search for unpublished literature. | Very low | Critical; identified post hoc |  |
| Li 2008^69^  Bright 2007^71^ | Complete ablation (among those with histological change) | Very serious  • some concerns due to assessments judged as unclear and high-risk^2^ | Unclear  • country of conduct not reported which may affect the delivery of care^4^  • it is unknown if patients had other GE conditions^5^ | No serious limitations  • one study | Serious  • small sample size (n=40)^9^ | No serious limitations  • SR did not assess for publication bias. Although small studies, comprehensive search and included search for unpublished literature. | Very low | Critical; identified post hoc |  |
| De Souza 2014^68^  Ackroyd 2004^72^ | Treatment failure at one year | Very serious  • some concerns due to assessments judged as unclear and high-risk^3^ | Unclear  • country of conduct not reported which may affect the delivery of care^4^  • it is unknown if patients had other GE conditions^5^ | No serious limitations  • one study | Very serious  • small sample size (n=40)^10^ | Serious  • comprehensive search not undertaken and uncertain about grey literature search | Very low | Critical; identified post hoc |  |

1 High risk of bias for performance bias, detection bias, and selective reporting (outcomes not clearly outlined in methodology); unclear sequence generation and potentially allocation concealment as per authors’ description. (Rees 2010 assessment).

2 Assessments (Li 2008) made using Jadad tool so at least unclear for randomization method and concealment; this information is corroborated by the assessments made in the Rees 2010 review. May be reasonable to assume that the high risk of performance and detection biases for the outcomes addressed in the Rees 2010 assessments would pertain here and corroborate the Jadad assessment of no blinding. Rees 2010 also identified selective reporting (see footnote 1) issues that could reasonably apply to this outcome.

3 Assessments in De Souza 2014 review not adequately detailed. Using assessments from Rees 2010 and Li 2008, sequence generation is unclear, likely also for concealment; high risk for performance and detection bias and selective reporting could apply to this outcome.

4 Country of conduct may affect the delivery of care in light of potential contextual influences such as differences in training or equipment.

5 The reviews do not report if the patients had other GE conditions, which were part of the exclusion criteria for this overview, but the effect on treatment is expected to be minimal

6 Likely low study sample based on other outcomes for the same study (sample size<rule of thumb of n=300 events)

7 Based on few events, CI for relative effects include both appreciable benefit and harm, and absolute CI reasonably includes appreciable benefit and harm

8 Based on low study sample (sample size<rule of thumb of n=300 events)

9 Based on low study sample (sample size<rule of thumb of n=300 events) and including little to no absolute effect and appreciable benefit

10 Based on few events, CI for relative effects include both appreciable benefit and harm, and absolute CI reasonably includes appreciable benefit and harm

*Bright 2007 is a follow-up to Ackroyd 2004

**Evidence Set 5: Thermal ablative techniques combined with pharmacological therapy vs Pharmacological therapy**

***Evidence Set 5.1 RFA + PPI vs PPI: Results table***

*Based on one primary study: Shaheen 2009, USA*^75^

| **5.1 Radiofrequency ablation (RFA) + Proton Pump Inhibitor (PPI) vs Proton Pump Inhibitor (PPI)** | | | | | | | | | |
| --- | --- | --- | --- | --- | --- | --- | --- | --- | --- |
| **Author Year** | **Outcome** | **Results: n/N (%)** | | | **Effect estimate (95% CI)** | **Absolute**  **Risk Difference (ARD)** | **AMSTAR† & GRADE‡** | **Notes** | |
|  |  | **Study, Country** | **RFA + PPI** | **PPI** |  |  |  |  |  |
| **Progression to EAC** | | | | | | | | | |
| Rees 2010^60^ | Progression to cancer at five years or latest time point | Shaheen 2009^75^, USA | 1/84  (1.2%) | 4/43  (9.3%) | OR 0.12  (0.01, 1.09) | ARD with intervention: 81 fewer per 1,000 (from 92 fewer to 8 more);  Risk with control: 93 per 1,000 | **AMSTAR**: Low  **Certainty**: Low | | Includes both low- and high-grade dysplasia. |
| Qumseya 2017^73^ | Cumulative progression to EAC over follow up | Shaheen 2009^75^, USA | 0/42 | 0/21 | Not reported^¥^ | Not estimable | **AMSTAR**: Low  **Certainty**: Very low | | Comparison labelled as RFA vs surveillance in SR, but assumption that it is the same study, but only reporting on those with LGD, as stated in review. |
| **Progression from non-dysplastic BE to BE with dysplasia** | | | | | | | | | |
| Rees 2010^60^ | Progression to higher grades of dysplasia | Shaheen 2009^75^, USA | 3/84  (3.6%) | 7/43  (16%) | **OR 0.19**  **(0.05, 0.78)** | ARD with intervention: 127 fewer per 1,000 (from 31 fewer to 153 fewer);  Risk with control: 163 per 1,000 | **AMSTAR**: Low  **Certainty**: Low | | The text reports that there was no data for those progressing from IM to dysplasia, however the forest plot is titled “number progressing from dysplasia from IM”. |
| Qumseya 2017^73^ | Progression to high-grade dysplasia | Shaheen 2009^75^, USA | 2/42  (4.8%) | 3/21*  (14.3%) | **RR 0.33  (0.06, 1.84)  Event rates^¥¥^ | ARD with intervention: 91 fewer per 1,000 (from 133 fewer to 92 more);  Risk with control: 143 per 1,000 | **AMSTAR**: Low  **Certainty**: Very low | | Comparison labelled as RFP vs surveillance in SR, but assumption that it is the same study, but only reporting on those with LGD, as stated in review. |
| Qumseya 2017^73^ | Progression to high-grade dysplasia (per patient-year) (among those with LGD) | Shaheen 2009^75^, USA | 0.0238 | 0.1429 | Not reported^¥¥¥^ | Not estimable | **AMSTAR**: Low  **Certainty**: Very low | | Comparison labelled as RFA vs surveillance in SR, but assumption that it is the same study, but only reporting on those with LGD, as stated in review. |
| Pandey 2018^74^ | Progression to high-grade dysplasia | Shaheen 2009^75^, USA | 2/42  (4.8%) | 3/22*  (13.6%) | OR 0.32  (0.05, 2.06) | ARD with intervention: 93 fewer per 1,000 (from 130 fewer to 145 more);  Risk with control: 136 per 1,000 | **AMSTAR:** Critically Low  **Certainty:** Very low | | The forest plot (figure 5) of Pandey et al., 2018 refers to progression to high grade dysplasia (HGD) or cancer; however, the corresponding numbers in Table 2 of the review labelled as progression to HGD only.  Discrepant denominator compared to Qumseya 2017. |
| **Complete clearance of dysplasia** ϯ | | | | | | | | | |
| Rees 2010^60^ | Complete eradication of dysplasia at 12 months | Shaheen 2009^75^, USA | 72/84 (86%) | 9/43 (21%) | **OR 22.67**  **(8.72, 58.94)** | ARD with intervention: 648 more per 1,000 (from 488 more to 730 more);  Risk with control: 209 per 1,000 | **AMSTAR:** Low  **Certainty:** Low | | RR 4.10 (3.33, 4.49), calculated from the OR |
| Pandey 2018^74^ | Complete eradication of dysplasia | Shaheen 2009^75^, USA | 38/42  (90%) | 5/22  (23%) | ****RR 3.98**  **(1.83 to 8.66)** | ARD with intervention: 677 more per 1,000 (from 189 more to 1,000 more);  Risk with control: 227 per 1,000 | **AMSTAR:** Critically Low  **Certainty:**  Very low | | Standardized Mean Difference=90.50; 95%CI=90.40, 90.60; SE=0.05  Evaluated only LGD patients. |
| **Complete eradication of BE at 12 months** ϯ | | | | | | | | | |
| Rees 2010^60^ | Complete eradication of BE at 12 months | Shaheen 2009^75^, USA | 65/84  (77%) | 1/43  (2.3%) | **OR 143.68**  **(18.53, 1113.87)** | ARD with intervention: 751 more per 1,000 (from 283 more to 940 more);  Risk with control: 23 per 1,000 | **AMSTAR**: Low  **Certainty**: Low | | Result for PPI group in text differs from forest plot (0/43).  RR 33.27 (13.16, 41.44), calculated from the OR |
| **Complete clearance of intestinal metaplasia** | | | | | | | | | |
| Pandey 2018^74^ | Complete eradication of intestinal metaplasia | Shaheen 2009^75^, USA | 34/42  (81.0%) | 1/22  (4.6%) | ****RR 17.81**  **(2.61, 121.54)** | ARD with intervention: 764 more per 1,000 (from 73 more to 1,000 more);  Risk with control: 45 per 1,000 | **AMSTAR:** Critically Low  **Certainty:**  Very low | | Standardized Mean Difference=81.00; 95%CI=80.88, 81.12; SE=0.06 |
| **Treatment failure** ϯ | | | | | | | | | |
| De Souza 2014^68^ | Treatment Failure (no ablation of BE) at one year | Shaheen 2009^75^, USA | 19/84  (23%) | 42/43  (98%) | **ARI 0.751**  **(0.651, 0.851)**  ****RR 0.23**  **(0.16, 0.34)** | ARD with intervention: 752 fewer per 1,000 (645 fewer to 820 fewer)  Risk with control: 977 per 1,000 | **AMSTAR:** Critically low  **Certainty:** Very low | Labelled as RFA vs PPI in review, but assumption has been made that it is the same comparator as in Rees 2010. | |
| **Stricture formation** ϯ | | | | | | | | | |
| Rees 2010^60^ | Stricture formation | Shaheen 2009^75^, USA | 5/84  (6.0%) | 0/43 | OR 6.02  (0.33, 111.44) | Not estimable | **AMSTAR:** Low  **Certainty**: Very low | |  |
| **Perforations** | | | | | | | | | |
| Pandey 2018^74^ | Perforations | Shaheen 2009^75^, USA | No instances of perforation were reported in total 84 patients. | | | | **AMSTAR:** Critically Low  **Certainty:** Very low | |  |
| **Bleeding** | | | | | | | | | |
| Pandey 2018^74^ | Bleeding | Shaheen 2009^75^, USA | One event (1/84) was reported, but data was not presented per arm. | | | | **AMSTAR:** Critically Low  **Certainty:** Very low | |  |

**†** see Supplementary table 1 for further details on AMSTAR domain ratings

‡ see Evidence Set 4.2: GRADE domains table for further details on GRADE domain ratings

ϯ post-hoc outcome

**the effect estimate was not reported in the original SR, but calculated by the overview team

^¥^ Between groups comparison was not reported but only event rate per arm was provided: RFA: 0.002, 95% CI -0.012, 0.017, p-value 0.752. Surveillance: 0.005, 95% CI –0.025, 0.034, p-value 0.752

^¥¥^ Between groups comparison was not reported but only event rate per arm was provided: RFA: 0.048, 95% CI 0.012,0.171, p-value 0.000 Surveillance:  0.143, 95% CI 0.047, 0.361, p-value 0.004

^¥¥¥^ Between groups comparison was not reported but only event rate per arm was provided: RFA: 0.048, 95% CI -0.018, 0.114, p-value 0.157. Surveillance: 0.143, 95% CI -0.019, 0.305, p-value 0.083

*Note*: Shaheen 2009 is cited as Shaheen 2008 in Rees 2010, but refers to the same study as that in De Souza 2014. In Rees 2010, the comparison is labelled as RFA vs sham in the text description, but in the tables of included studies, it states that all participants were given PPI.

Bolded effect estimates refer to statistically significant results.

Underlined first author name, publication year refers to a unique study included in more than one review.

***Evidence Set 5.1 RFA + PPI vs PPI: GRADE domains table***

| **5.1 Radiofrequency ablation (RFA) + Proton Pump Inhibitor (PPI) vs Proton Pump Inhibitor (PPI)** | | | | | | | | |
| --- | --- | --- | --- | --- | --- | --- | --- | --- |
| **Review**  **Study (ies)** | **Outcome** | **Study Limitations** | **Indirectness** | **Inconsistency** | **Imprecision** | **Other considerations** | **Certainty** | **Importance** |
| Rees 2010^60^  Shaheen 2009^75^ | Progression to EAC at five years or latest time point | Serious  • some concerns due to assessments judged as unclear^1^ | No serious limitations  • it is unknown if patients had other GE conditions^5^ | No serious limitations  • one study | Serious  • small sample size (n=117)^6^ | No serious limitations  • SR did not assess for publication bias. Although small studies, comprehensive search and included search for unpublished literature. | Low | Critical |
| Qumseya 2017^73^  Shaheen 2009^75^ | Cumulative progression to EAC over follow up (among those with LGD) | Serious  • some concerns due to assessments judged as unclear^2^ | No serious limitations  • it is unknown if patients had other GE conditions^5^ | No serious limitations  • one study | Serious  • sparse number of events, small sample size (n=63)^7^ | Serious  • publication bias detected | Very low | Critical |
| Rees 2010^60^  Shaheen 2009^75^ | Progression to higher grades of dysplasia | Serious  • some concerns due to assessments judged as unclear^1^ | No serious limitations  • it is unknown if patients had other GE conditions^5^ | No serious limitations  • one study | Serious  • small sample size (n=117)^7^ | No serious limitations  • SR did not assess for publication bias. Although small studies, comprehensive search and included search for unpublished literature | Low | Critical |
| Qumseya 2017^73^  Shaheen 2009^75^ | Progression to high-grade dysplasia | Serious  • some concerns due to assessments judged as unclear^2^ | No serious limitations  • it is unknown if patients had other GE conditions^5^ | No serious limitations  • one study | Very serious  • small sample size (n=63)^8^ | Serious  • publication bias detected | Very low | Critical |
| Qumseya 2017^73^  Shaheen 2009^75^ | Progression to high-grade dysplasia (per person/year) | Serious  • some concerns due to assessments judged as unclear^2^ | No serious limitations  • it is unknown if patients had other GE conditions^5^ | No serious limitations  • one study | Serious  • small sample size (n=63), unclear number of events^9^ | Serious  • publication bias detected | Very low | Critical |
| Pandey 2018^74^  Shaheen 2009^75^ | Progression to high-grade dysplasia | Serious  • some concerns due to assessments judged as unclear^3^ | No serious limitations  • it is unknown if patients had other GE conditions^5^ | No serious limitations  • one study | Very serious  • fewer events, small sample size (n=64)^8^ | Serious  • publication bias was assessed but for a composite outcome (progression to HGD or cancer) and included only two RCTs and one observational study not relevant to overview. Small studies; search for unpublished research was not reported in the review. | Very low | Critical |
| Pandey 2018^74^  Shaheen 2009^75^ | Complete eradication of intestinal metaplasia | Serious  • some concerns due to assessments judged as unclear^3^ | No serious limitations  • it is unknown if patients had other GE conditions^5^ | No serious limitations  • one study | Serious  • small sample size (n=64)^7^ | Serious  • publication bias was assessed but included only two RCTs and four observational studies not relevant to overview; Small studies; search for unpublished research was not reported in the review. | Very low | Critical |
| Rees 2010^60^  Shaheen 2009^75^ | Complete eradication of dysplasia at 12 months | Serious  • some concerns due to assessments judged as unclear^1^ | No serious limitations  • it is unknown if patients had other GE conditions^5^ | No serious limitations  • one study | Serious  • small sample size (n=117)^7^ | No serious limitations  • SR did not assess for publication bias. Although small studies, comprehensive search and included search for unpublished literature. | Low | Critical; identified post hoc |
| Pandey 2018^74^  Shaheen 2009^75^ | Complete eradication of dysplasia | Serious  • some concerns due to assessments judged as unclear^3^ | No serious limitations  • it is unknown if patients had other GE conditions^5^ | No serious limitations  • one study | Serious  • small sample size (n=64)^7^ | Serious  • publication bias was assessed but included only two RCTs and four observational studies not relevant to overview; Small studies; search for unpublished research was not reported in the review. | Very low | Critical |
| Rees 2010^60^  Shaheen 2009^75^ | Complete eradication of BE at 12 months | Serious  • some concerns due to assessments judged as unclear^1^ | No serious limitations  • it is unknown if patients had other GE conditions^5^ | No serious limitations  • one study | Serious  • small sample sizes (n=117)^7^ | No serious limitations  • SR did not assess for publication bias. Although small studies, comprehensive search and included search for unpublished literature. | Low | Critical; identified post hoc |
| De Souza 2014^68^  Shaheen 2009^75^ | Treatment failure at one year | Serious  • some concerns due to assessments judged as unclear^4^ | No serious limitations  • it is unknown if patients had other GE conditions^5^ | No serious limitations  • one study | Serious  • small sample size (n=117)^7^ | Serious  • comprehensive search not undertaken and uncertain about grey literature search | Very low | Critical; identified post hoc |
| Rees 2010^60^  Shaheen 2009^75^ | Stricture formation | Serious  • some concerns due to assessments judged as unclear^1^ | No serious limitations  • it is unknown if patients had other GE conditions^5^ | No serious limitations  • one study | Very serious  • small sample size (n=117) and very wide CI due to zero events in one group^10^ | No serious limitations  • SR did not assess for publication bias. Although small studies, comprehensive search and included search for unpublished literature. | Very low | Critical; identified post hoc |
| Pandey 2018^74^  Shaheen 2009^75^ | Perforations | Serious  • some concerns due to assessments judged as unclear^3^ | No serious limitations  • it is unknown if patients had other GE conditions^5^ | No serious limitations  • one study | Serious  • small sample size (n=64)^7^ | Serious  • publication bias was not assessed for this outcome. Search for unpublished research was not stated in search strategy. | Very low | Critical |
| Pandey 2018^74^  Shaheen 2009^75^ | Bleeding | Serious  • some concerns due to assessments judged as unclear^3^ | No serious limitations  • it is unknown if patients had other GE conditions^5^ | No serious limitations  • one study | Serious  • small sample size (n=64)^7^ | Serious  • publication bias was not assessed for this outcome. Search for unpublished research was not stated in search strategy | Very low | Critical |

1 Allocation concealment unclear. (Rees 2010 assessment)

2 Assessments in Qumseya 2017 review do not map well to study limitations criteria. Using study-level assessments made in the Rees 2010 review, allocation concealment is at an unclear risk of bias.

3 Assessments as performed or reported in Pandey 2018 are of limited application to the study limitations domain. Using study-level assessment made in the Rees 2010 review, allocation concealment is at an unclear risk of bias.

4 Although the De Souza 2017 assessment yielded a Jadad score of 5, the Rees 2010 review assessment reported this study at an unclear risk of bias for allocation concealment.

5 The reviews do not report if the patients had other GE conditions, which was part of the exclusion criteria for this overview, but the effect on treatment is expected to be minimal

6 Based on low study sample (sample size<rule of thumb of n=300 events) and including little to no absolute effect and appreciable benefit

7 Based on few events/small study sample (sample size<rule of thumb of n=300 events)

8 Based on few events, CI for relative effects include both appreciable benefit and harm, and absolute CI reasonably includes appreciable benefit and harm

9 Based on low study sample (sample size<rule of thumb of n=400 people)

10 Based on few events and CI for relative effects include both appreciable benefit and harm. Absolute CI unknown.

**Evidence Set 6: Surgery vs Pharmacological therapy**

***Evidence Set 6.1 Anti-reflux surgery vs H2 receptor antagonist/Omeprazole: Results table***

*Based on one primary study: §Parrilla 2003*^76^

| **6.1 Anti-reflux surgery (Nissen Fundoplication) vs H2 receptor agonist/Omeprazole^a^** | | | | | | | | | | |
| --- | --- | --- | --- | --- | --- | --- | --- | --- | --- | --- |
| **Author Year** | **Outcome** | | **Results: n/N (%)** | | | **Effect estimate (95% CI)** | | **Absolute**  **Risk Difference (ARD)** | **AMSTAR† & GRADE‡** | **Notes** |
|  |  |  | **Study, Country** | **Surgery** | **H2RA/ Omeprazole** |  |  |  |  |  |
| **All-cause mortality** | | | | | | | | | | |
| Rees 2010^60^ | | Mortality | Parrilla 2003^76^, NR | 0 | 0 | Not estimable | | Not estimable | **AMSTAR:** Low  **Certainty**: Very low |  |
| **Progression to EAC** | | | | | | | | | | |
| Rees 2010^60^ | Progression to cancer | | Parrilla 2003^76^, NR | 2/53  (3.8%) | 2/40  (5%) | OR 0.75  (0.10, 5.53) | | ARD with intervention: 12 fewer per 1,000 (from 45 fewer to 175 more);  Risk with control: 50 per 1,000 | **AMSTAR:** Low  **Certainty**: Very low | Li 2008 provided similar results, with slight difference in the total N, but this did not change the overall effect estimate or ARD. |
| **Progression from non-dysplastic BE to BE with dysplasia** | | | | | | | | | | |
| Rees 2010^60^ | Progression to dysplasia from intestinal metaplasia | | Parrilla 2003^76^, NR | 1/44*  (2.3%) | 8/40  (20%) | **OR 0.09**  **(0.01, 0.78)** | | ARD with intervention: 178 fewer per 1,000 (from 37 fewer to 198 fewer);  Risk with control: 200 per 1,000 | **AMSTAR:** Low  **Certainty**: Very low | De novo dysplasia 3/58 in the surgery group and 8/43 in omeprazole group. |
| Li 2008^69^ | Progression from non-dysplastic BE to BE with dysplasia | | Parrilla 2003^76^, NR | 3/53*  (5.7%) | 8/40  (20%) | NR  **RR 0.28 (0.08, 1.00) | | ARD with intervention: 144 fewer per 1,000 (from 0 fewer to 184 fewer);  Risk with control: 200 per 1,000 | **AMSTAR:** Critically low  **Certainty:** Very low | 1/49 de novo dysplasia among those with successful surgery, 8/40 among the omeprazole group. The difference was statistically significant. |
| **Complete eradication of dysplasia** ϯ | | | | | | | | | | |
| Rees 2010^60^ | Complete eradication of dysplasia at 5-years | | Parrilla 2003^76^, NR | 5/58  (8.6%) | 3/43  (7.0%) | OR 1.26  (0.28, 5.58) | | ARD with intervention: 17 more per 1,000 (from 49 fewer to 225 more);  Risk with control: 70 per 1,000 | **AMSTAR:** Low  **Certainty**: Very low | 49 of the 58 patients were considered to have successful surgery |
| **Complete eradication of Barrett’s Esophagus (BE)** ϯ | | | | | | | | | | |
| Rees 2010^60^ | Complete eradication of BE at 5 years | | Parrilla 2003^76^, NR | 0/53 | 0/40 | Not estimable | | Not estimable | **AMSTAR:** Low  **Certainty**: Very low |  |
| **†** see Supplementary table 1 for further details on AMSTAR domain ratings  ‡ see Evidence Set 5: GRADE domain table for further details on GRADE domain ratings  ϯ post-hoc outcome  **the effect estimate was not reported in the original SR but calculated by the overview team  a patients prior to 1992 were given H2RA (ranitidine) and then converted to omeprazole | | | | | | | **Bolded** effect estimates refer to statistically significant results.  Underlined first author name, publication year refers to a unique study included in more than one review.  *§*the median (range) age was reported as 50(12-78) in medical arm, and 43(10-71) in surgical group in Li 2008 but according to Rees 2010’s only adults were eligible in the review.  *discrepant data | | | |

***Evidence Set 6.1 Anti-reflux surgery vs H2 receptor antagonist/Omeprazole: GRADE domains table***

| **6.1 Anti-reflux surgery (Nissen Fundoplication) vs H2 receptor antagonist/ Omeprazole^a^** | | | | | | | | |
| --- | --- | --- | --- | --- | --- | --- | --- | --- |
| **Review Studies** | **Outcome** | **Study Limitations** | **Indirectness** | **Inconsistency** | **Imprecision** | **Other considerations** | **Certainty** | **Importance** |
| Rees 2010^60^  Parrilla 2003^76^ | All-cause mortality | Very serious  • some concerns due to assessments judged as unclear and high risk^1^ | Unclear  • country of conduct not reported which may affect the delivery of care^3^  • it is unknown if patients had other GE conditions^4^ | No serious limitations  • one study | Serious  • small sample size (although sample is not mentioned for this outcome, but it is apparent from another outcome in this study (n=101))^5^ | No serious limitations  • SR did not assess for publication bias. Although small studies, comprehensive search and included search for unpublished literature. | Very low | Critical |
| Rees 2010^60^  Parrilla 2003^76^ | Progression to cancer | Very serious  • some concerns due to assessments judged as unclear and high-risk^1^ | Unclear  • country of conduct not reported which may affect the delivery of care^3^  • it is unknown if patients had other GE conditions^4^ | No serious limitations  • one study | Very serious  • small sample size (n=101), wide CI including only four events in total^6^ | No serious limitations  • SR did not assess for publication bias. Although small studies, comprehensive search and included search for unpublished literature. | Very low | Critical |
| Rees 2010^60^  Parrilla 2003^76^ | Progression from non-dysplastic BE to BE with dysplasia | Very serious  • some concerns due to assessments judged as unclear and high-risk^1^ | Unclear  • country of conduct not reported which may affect the delivery of care^3^  • it is unknown if patients had other GE conditions^4^ | No serious limitations  • one study | Serious  • small sample size (n=101)^7^ | No serious limitations  • SR did not assess for publication bias. Although small studies, comprehensive search and included search for unpublished literature. | Very low | Critical |
| Li 2008^69^  Parrilla 2003^76^ | Progression from non-dysplastic BE to BE with dysplasia | Very serious  • some concerns due to assessments judged as unclear and high-risk^2^ | Unclear  • country of conduct not reported which may affect the delivery of care^3^  • it is unknown if patients had other GE conditions^4^ | No serious limitations  • one study | Serious  • small sample size (n=101)^8^ | Serious  • SR did not assess for publication bias, and the review did not perform comprehensive or grey lit searches | Very low | Critical |
| Rees 2010^60^  Parrilla 2003^76^ | Complete eradication of dysplasia at 5 years | Very serious  • some concerns due to assessments judged as unclear and high-risk^1^ | Unclear  • country of conduct not reported which may affect the delivery of care^3^  • it is unknown if patients had other GE conditions^4^ | No serious limitations  • one study | Very serious  • small sample size (n=101), wide CI with only eight events in total^6^ | No serious limitations  • SR did not assess for publication bias. Although small studies, comprehensive search and included search for unpublished literature. | Very low | Critical; identified post hoc |
| Rees 2010^60^  Parrilla 2003^76^ | Complete eradication of BE at 5 years | Very serious  • some concerns due to assessments judged as unclear and high-risk^1^ | Unclear  • country of conduct not reported which may affect the delivery of care^3^  • it is unknown if patients had other GE conditions^4^ | No serious limitations  • one study | Serious  • few events, small sample size (n=101)^7^ | No serious limitations  • SR did not assess for publication bias. Although small studies, comprehensive search and included search for unpublished literature. | Very low | Critical; identified post hoc |

1 Authors (Rees 2010) report that due to the nature of the study blinding (performance bias) was impossible and deemed it as unclear risk. However, two other studies in this review with the same explanation were deemed as high risk. This is corroborated by the no blinding assessment (Jadad) in the Li 2008 review; with unclear risk of bias for attrition (Rees 2010).

2 High risk for lack of blinding in Jadad assessment (Li 2008 review) reasonably corroborated by high risk of performance and detection biases (Rees 2010 review) for other outcomes in this study. The unclear risk of bias for attrition for other outcomes in this study may also pertain to this outcome.

3 Country of conduct may affect the delivery of care in light of potential contextual influences such as a change in the accessibility to the regimens (for pharmacological treatment), and differences in training or equipment (surgical treatment).

4 The reviews do not report if the patients had other GE conditions, which was part of the exclusion criteria for this overview, but the effect on treatment is expected to be minimal

5 Likely low study sample based on other outcomes for the same study (sample size<rule of thumb of n=300 events)

6 Based on few events, CI for relative effects include both appreciable benefit and harm, and absolute CI reasonably includes appreciable benefit and harm

7 Based on few events/ small study sample (sample size<rule of thumb of n=300 events)

8 Based on few events/ small study sample (sample size<rule of thumb of n=300 events). Relative and absolute CIs include the possibility of little to no effect.

**Evidence Set 7: Chemical ablative technique vs Chemical ablative technique**

***Evidence Set 7.1 PDT (5-ALA) vs PDT (Photofrin): Results table***

*Based on one primary study in abstract: Mackenzie 2008*^77^

| **7.1 Photodynamic therapy (PDT) using 5-ALA vs Photodynamic therapy (PDT) using Photofrin** | | | | | | | | | |
| --- | --- | --- | --- | --- | --- | --- | --- | --- | --- |
| **Author Year** | | **Outcome** | **Results: n/N** | | | **Effect estimate (95% CI)** | **Absolute**  **Risk Difference (ARD)** | **AMSTAR† & GRADE‡** | **Notes** |
|  |  |  | **Study** | **5-ALA** | **Photofrin** |  |  |  |  |
| **Eradication of high-grade dysphagia** ϯ | | | | | | | | | |
| Rees 2010^60^ | Eradication of high-grade dysphagia | | Mackenzie 2008 (abstract)^77^, NR | 14/14  (100%) | 9/14  (64%) | OR 16.79  (0.83, 340.08) | ARD not calculated, as the data were from an abstract. | **AMSTAR:** Low  **Certainty:** Very low | The trial reported preliminary data only, as recruitment is not yet complete. Reported as remission in Fayter 2010.  RR 1.51 (0.93, 1.55), calculated from the OR |
| **Stricture formation** ϯ | | | | | | | | | |
| Rees 2010^60^ | Stricture formation | | Mackenzie 2008 (abstract)^77^, NR | 1/16  (6.3%) | 6/16  (28%) | OR 0.11  (0.01, 1.07) | ARD not calculated, as the data were from an abstract. | **AMSTAR:** Low  **Certainty:** Very low |  |

**†** see Supplementary table 1 for further details on AMSTAR domain ratings

‡ see Evidence Set 7.1: GRADE domains table for further details on GRADE domain ratings

ϯ post-hoc outcome

***Evidence Set 7.1 PDT (5-ALA) vs PDT (Photofrin): GRADE domains table***

| **7.1 Photodynamic therapy (PDT) using 5-ALA vs Photodynamic therapy (PDT) using Photofrin** | | | | | | | | |
| --- | --- | --- | --- | --- | --- | --- | --- | --- |
| **Review Studies** | **Outcome** | **Study Limitations** | **Indirectness** | **Inconsistency** | **Imprecision** | **Other considerations** | **Certainty** | **Importance** |
| Rees 2010^60^  Mackenzie 2008 (abstract)^77^ | Eradication of high-grade dysplasia | Very serious  • abstract^1^ | Unclear  • country of conduct not reported which may affect the delivery of care^2^  • it is unknown if patients had other GE conditions^3^ | No serious limitations  • one study | Serious  • small sample sizes (n=32) and very wide CI^4^ | No serious limitations  • SR did not assess for publication bias. Although small studies, comprehensive search and included search for unpublished literature. | Very low | Critical; identified post hoc |
| Rees 2010^60^  Mackenzie 2008 (abstract)^77^ | Stricture formation | Very serious  • abstract^1^ | Unclear  • country of conduct not reported which may affect the delivery of care^2^  • it is unknown if patients had other GE conditions^3^ | No serious limitations  • one study | Serious  • small sample size (n=32)^4^ | No serious limitations  • SR did not assess for publication bias. Although small studies, comprehensive search and included search for unpublished literature. | Very low | Critical; identified post hoc |

1 Authors identify high risk in ‘other bias’ domain because in abstract form.

2 Country of conduct may affect the delivery of care in light of potential contextual influences such as differences in training or equipment

3 The reviews do not report if the patients had other GE conditions, which was part of the exclusion criteria for this overview, but the effect on treatment is expected to be minimal

4 Based on few events/ small study sample (sample size<rule of thumb of n=300 events). CI includes little to no effect and appreciable benefit.

***Evidence Set 7.2 PDT with different treatment parameters: Results table***

*Based on three primary studies: Kelty 2004b*^73^*; Mackenzie 2007(abstract)*^78^*; Mackenzie 2009*^79^

| **7.2 PDT with different treatment parameters** | | | | | |
| --- | --- | --- | --- | --- | --- |
| **Author Year** | **Outcome** | **Results** | | **AMSTAR† & GRADE‡** | **Notes** |
|  |  | **Study** | **Narrative results** |  |  |
| **Multiple outcomes reported narratively** ϯ | | | | | |
| Fayter  2010^70^ | Cancer risk at 36 months follow-up | Mackenzie 2007 (abstract)^78^ | Patients with HGD receiving high-dose ALA–PDT (60 mg/kg) and high-dose red light (1000 J/cm) had a significant decrease in cancer risk compared with treatment groups with lower doses of photosensitiser and/or lower light doses (3% risk vs 24% risk). | **AMSTAR:** Critically low  **Certainty:** Very low | ALA–PDT with varying doses of light and comparing red or green light (abstract only)^78^ |
| Fayter  2010^70^ | Lower rates of adenocarcinoma | Mackenzie 2007 (abstract)^78^  Mackenzie 2009^79^ | ALA red light was associated with lower rates of adenocarcinoma than green light (8% vs 45%, p < 0.05).^78^  60-mg ALA red light was statistically significantly more successful than 30-mg ALA red light (p=0.03) and 30-mg ALA green light (p=0.005).^79^ | **AMSTAR:** Critically low  **Certainty:** Very low to low | ALA–PDT with varying doses of light and comparing red or green light (abstract only)^78^  Discrepancies in what comparisons are: the description compares ALA–PDT with red light vs ALA with green  light at 30 or 60 mg/kg, however the results compare 60-mg red light to 30-mg redlight and 60-mg red light to 30-mg green light^79^ |
| Fayter  2010^70^ | Reductions in BE | Kelty 2004b^80^ | Among patients with no dysplasia (5 patients per group), 30-mg/kg and fractionated groups showed the greatest reductions in Barrett’s epithelium (results not statistically significant). | **AMSTAR:** Critically low  **Certainty:** Very low to low | ALA–PDT at 30 mg/kg or 60 mg/kg at 4- or 6-hour incubation times or with fractionated illumination^80^ |
| Fayter  2010^70^ | Stricture | Kelty 2004b^80^  Mackenzie 2007 (abstract)^78^ | No patients developed strictures. | **AMSTAR:** Critically low  **Certainty:** Very low to low | ALA–PDT with varying doses of light and comparing red or green light (abstract only)^78^ |
| Fayter  2010^70^ | Perforation | Kelty 2004b^80^ | Reported no major side effects in terms of perforations. | **AMSTAR:** Critically low  **Certainty:** Very low to low | ALA–PDT at 30 mg/kg or 60 mg/kg at 4- or 6-hour incubation times or with fractionated illumination^80^ |

**†** see Supplementary table 1 for further details on AMSTAR domain ratings

‡ see Evidence Set 7.2: GRADE domains table for further details on GRADE domain ratings

ϯ post-hoc outcome

***Evidence Set 7.2 PDT with different treatment parameters: GRADE domains table***

| **7.2 PDT with different treatment parameters** | | | | | | | | | |
| --- | --- | --- | --- | --- | --- | --- | --- | --- | --- |
| **Review Studies** | **Outcome** | **Study Limitations** | **Indirectness** | **Inconsistency** | **Imprecision** | **Other considerations** | **Certainty** | **Importance** |  |
| Fayter  2010^70^  Mackenzie 2007^78^  (abstract) | Cancer risk | Very serious  • abstract^1^ | Unclear  • country of conduct not reported which may affect the delivery of care^3^  • it is unknown if patients had other GE conditions^4^ | No serious limitations  • one study | Serious  • small sample size (n=72)^5^ | No serious limitations  • SR did not assess for publication bias. Although small studies, comprehensive search and included search for unpublished literature. | Very low | Critical |  |
| Fayter  2010^70^  Mackenzie 2007^78^  (abstract) Mackenzie 2009^79^ | Lower rates of adenocarcinoma | Very serious to serious^1,2^ | Unclear  • country of conduct not reported which may affect the delivery of care  • it is unknown if patients had other GE conditions^4^ | No serious limitations | Serious  • small sample size (n=101)^5^ | No serious limitations  • SR did not assess for publication bias. Although small studies, comprehensive search and included search for unpublished literature. | Very low to low | Critical |  |
| Fayter  2010^70^  Kelty 2004b^80^ | Reductions in BE | Serious^2^ | Unclear  • country of conduct not reported which may affect the delivery of care^3^  • it is unknown if patients had other GE conditions^4^ | No serious limitations  • one study | Serious  • small sample size (n=10)^5^ | No serious limitations  • SR did not assess for publication bias. Although small studies, comprehensive search and included search for unpublished literature. | Very low to low | Critical; post hoc |  |
| Fayter  2010^70^  Kelty 2004b^80^  Mackenzie 2007^78^  (abstract) | Strictures | Very serious to serious^1,2^ | Unclear  • country of conduct not reported which may affect the delivery of care^3^  • it is unknown if patients had other GE conditions^4^ | No serious limitations | Serious  • small sample size (n=29)^5^ | No serious limitations  • SR did not assess for publication bias. Although small studies, comprehensive search and included search for unpublished literature. | Very low to low | Critical |  |
| Fayter  2010^70^  Kelty 2004b^80^ | Perforation | Serious^2^ | Unclear  • country of conduct not reported which may affect the delivery of care^3^  • it is unknown if patients had other GE conditions^4^ | No serious limitations  • one study | Serious  • small sample size (n=25)^5^ | No serious limitations  • SR did not assess for publication bias. Although small studies, comprehensive search and included search for unpublished literature. | Very low to low | Critical |  |

1 Information provided was only in aggregate; assessments made on a study available only in abstract form and as per Rees 2010 assessment, can be indicated at high risk for ‘other bias’ domain

2 Information provided was only in aggregate with sequence generation unclear for 80% of the included studies, concealment allocation unclear for 90% of the included studies, and blinding was unclear in 62% of the included studies

3 Country of conduct may affect the delivery of care in light of potential contextual influences such as differences in training or equipment

4 The review does not report if the patients had other GE conditions, which was part of the exclusion criteria for this overview

5 Based on few events/ small study sample (sample size<rule of thumb of n=300 events). Insufficient reporting of information to judge extent of imprecision of data.

**Evidence Set 8: Thermal ablative technique vs Surveillance**

***Evidence Set 8.1 RFA vs Surveillance (endoscopic): Results table***

*Based on one primary study: Phoa 2014*^81^

| **8.1 Radiofrequency ablation (RFA) vs Surveillance (endoscopic)** | | | | | | | | | | |
| --- | --- | --- | --- | --- | --- | --- | --- | --- | --- | --- |
| **Author Year** | **Outcome** | **Results: n/N; n/year** | | | | **Effect estimate (95% CI)** | **Absolute**  **Risk Difference** | **AMSTAR† & GRADE‡** | **Notes** |  |
|  |  | **Study, Country** | **RFA** | | **Surv.** |  |  |  |  |  |
| **Progression to EAC** | | | | | | | | | | |
| Qumseya 2017^73^ | Progression to EAC: Cumulative progression over the follow up period | Phoa 2014^81^, Netherlands | 1/68  (1.5%) | 6/68  (8.8%) | | **RR 0.17  (0.02, 1.35) | ARD with intervention: 73 fewer per 1,000 (from 86 fewer to 31 more);  Risk with control: 88 per 1,000 | **AMSTAR:** Low  **Certainty:** Very low | Cumulative disease progression rates to EAC reported |  |
| Qumseya 2017^73^ | Progression to EAC: progression per patient-year | Phoa 2014^81^, Netherlands | 0.00501 | 0.03852 | | Not reported^¥^ | Not estimable | **AMSTAR:** Low  **Certainty:** Very low to low | Incidence rate of disease progression to EAC reported |  |
| **Progression from low-grade to high-grade dysplasia (HGD)** | | | | | | | | | | |
| Qumseya 2017^73^ | Progression to high-grade dysplasia | Phoa 2014^81^, Netherlands | 0/68 | 12/68  (18%) | | **RR 0.04  (0.00 to 0.66)  Event rate^¥¥^ | ARD with intervention: 169 fewer per 1,000 (60 fewer to ---)  Risk with control: 176 per 1,000 | **AMSTAR:** Low  **Certainty:** Very low | Cumulative disease progression rates to HGD reported in review but do not provide a difference between groups |  |
| Pandey 2018^74^ | Progression to high-grade dysplasia | Phoa 2014^81^, Netherlands | 0/68 | 18/68  (26%) | | ****RR 0.03**  **(0.00, 0.44)** | ARD with intervention: 257 fewer per 1,000 (from 148 fewer to ---);  Risk with control: 265 per 1,000 | **AMSTAR:** Critically Low  **Certainty:** Very low to low | The forest plot (figure 5) of Pandey et al., 2018 refers to progression to HGD or cancer; however, Table 2 documents only 1 EAC event in RFA group only. |  |
| Qumseya 2017^73^ | Progression to high-grade dysplasia (per patient-year) | Phoa 2014^81^, Netherlands | 0 | 0.07704 | | Not reported  Not reported^¥¥¥^ | Not estimable | **AMSTAR:** Low  **Certainty:** Very low to low | Incidence rate of disease progression to HGD reported but do not provide a difference between groups |  |
| **Eradication of dysplasia** | | | | | | | | | | |
| Pandey 2018^74^ | Complete eradication of dysplasia | Phoa 2014^81^, Netherlands | 62/63  (98%) | 19/68  (28%) | | ****RR 3.52**  **(2.40, 5.17)** | ARD with intervention: 704 more per 1,000 (391 more to 1,000 more);  Risk with control: 279 per 1,000 | **AMSTAR:** Critically Low  **Certainty:** Very low to low | Standardized Mean Difference=98.4; 95%CI=98.63, 98.44); SE=0.02 |  |
| **Eradication of intestinal metaplasia** | | | | | | | | | | |
| Pandey 2018^74^ | Complete eradication of intestinal metaplasia | Phoa 2014^81^, Netherlands | 54/60  (90%) | 0/68 | | ****RR 123.30 (7.78, 1954.10)** | Not estimable | **AMSTAR:** Critically Low  **Certainty:** Very low to low | Standardized Mean Difference=90.00; 95%CI=89.92, 90.08); SE=0.04 |  |
| **Stricture formation** | | | | | | | | | | |
| Pandey 2018^74^ | Stricture formation | Phoa 2014^81^, Netherlands | 8 events were reported, but data was not presented per arm. | | | | | **AMSTAR:** Critically Low  **Certainty:** Very low to low |  |  |
| **Perforations** | | | | | | | | | | |
| Pandey 2018^74^ | Perforations | Phoa 2014^81^, Netherlands | No instances of perforation were reported among the 68 patients. | | | | | **AMSTAR:** Critically Low  **Certainty:** Very low to low |  |  |
| **Bleeding** | | | | | | | | | | |
| Pandey 2018^74^ | Bleeding | Phoa 2014^81^, Netherlands | One event in total (1/68) was reported, but data was not presented per arm. | | | | | **AMSTAR:** Critically Low  **Certainty:** Very low to low |  |  |

**†** see Supplementary table 1 for further details on AMSTAR domain ratings

‡ see Evidence Set 8.1: GRADE domains table for further details on GRADE domain ratings

ϯ post-hoc outcome

**the effect estimate was not reported in the original SR but calculated by the overview team

^¥^ Between groups comparison was not reported but only event rate per arm was provided: RFA: 0.015, 95%CI 0.002, 0.097, p-value 0.000, Surveillance: 0.088, 95%CI 0.040, 0.183, p-value 0.000

^¥¥^ RFA: 0.007, 95% CI 0.00,0.105, p-value 0.001, Surveillance: 0.176, 95% CI 0.103,0.296, p-value 0.000

^¥¥¥^ Between groups comparison was not reported but only event rate per arm was provided: RFA: 0.003, 95% CI -0.004, 0.009, p-value 0.480, Surveillance: 0.077, 95% CI 0.033, 0.121, p-value 0.001Bolded effect estimates refer to statistically significant results.

Underlined first author name, publication year refers to a unique study included in more than one review.

***Evidence Set 8.1 RFA vs Surveillance (endoscopic): GRADE domains table***

| **8.1 Radiofrequency ablation (RFA) vs Surveillance (endoscopic)** | | | | | | | | | |
| --- | --- | --- | --- | --- | --- | --- | --- | --- | --- |
| **Review Studies** | **Outcome** | **Study Limitations** | **Indirectness** | **Inconsistency** | **Imprecision** | **Other considerations** | **Certainty** | **Importance** |  |
| Qumseya 2017^73^  Phoa 2014^81^ | Progression to EAC: cumulative progression over the follow-up period | Unclear  • insufficient information to judge^1^ | No serious limitations  • it is unknown if patients had other GE conditions^3^ | No serious limitations  • one study | Very serious  • small sample size (n=136) with only seven events in total^4^ | Serious  • publication bias detected | Very low | Critical |  |
| Qumseya 2017^73^  Phoa 2014^81^ | Progression to EAC: progression per patient per year | Unclear  • insufficient information to judge^1^ | No serious limitations  • it is unknown if patients had other GE conditions^3^ | No serious limitations  • one study | Serious  • zero/ unclear number of events (yet incidence rates generated), small sample size (n=136)^5^ | Serious  • publication bias detected | Very low to low | Critical |  |
| Qumseya 2017^73^  Phoa 2014^81^ | Progression to high-grade dysplasia | Unclear  • insufficient information to judge^1^ | No serious limitations  • it is unknown if patients had other GE conditions^3^ | Serious  • considerable heterogeneity (I^2^=79%, p<0.001). No overlap of CI. No detailed information provided to account for heterogeneity | Serious  • small sample size (n=136)^6^ | Serious  • publication bias detected | Very low | Critical |  |
| Pandey 2018^74^  Phoa 2014^81^ | Progression to high-grade dysplasia | Unclear  • insufficient information to judge^2^ | No serious limitations  • it is unknown if patients had other GE conditions^3^ | No serious limitations  • one study | Serious  • small sample size (n=136)^6^ | Serious  • publication bias was assessed for a composite outcome (progression to HGD or cancer) and included only two RCTs and one observational study not relevant to overview. Small studies; search for unpublished research was not reported in the review. | Very low to low | Critical |  |
| Qumseya 2017^73^  Phoa 2014^81^ | Progression to high-grade dysplasia: progression per patient per year | Unclear  • insufficient information to judge^1^ | No serious limitations  • it is unknown if patients had other GE conditions^3^ | No serious limitations  • one study | Serious  • small sample size (n=136)^7^ | Serious  • publication bias detected | Very low to low | Critical |  |
| Pandey 2018^74^  Phoa 2014^81^ | Complete eradication of dysplasia | Unclear  • insufficient information to judge^2^ | No serious limitations  • it is unknown if patients had other GE conditions^3^ | No serious limitations  • one study | Serious  • small sample size (n=136)^6^ | Serious  • publication bias assessed that included only two RCTs and four observational studies not relevant to overview;  Small studies; search for unpublished research was not reported in the review. | Very low to low | Critical |  |
| Pandey 2018^74^  Phoa 2014^81^ | Complete eradication of intestinal metaplasia | Unclear  • insufficient information to judge^2^ | No serious limitations  • it is unknown if patients had other GE conditions^3^ | No serious limitations  • one study | Serious  • small sample size (n=136)^6^ | Serious  • publication bias assessed but included only two RCTs and with 4 observational studies not relevant to overview; Small studies; search for unpublished research was not reported in the review. | Very low to low | Critical |  |
| Pandey 2018^74^  Phoa 2014^81^ | Stricture formation | Unclear  • insufficient information to judge^2^ | No serious limitations  • it is unknown if patients had other GE conditions^3^ | No serious limitations  • one study | Serious  • small sample size (n=136) with only eight events in total^6^ | Serious  • publication bias was not assessed for this outcome. Search for unpublished research was not reported. | Very low to low | Critical |  |
| Pandey 2018^74^  Phoa 2014^81^ | Perforations | Unclear  • insufficient information to judge^2^ | No serious limitations  • it is unknown if patients had other GE conditions^3^ | No serious limitations  • one study | Serious  • small sample size (n=136) with zero events in total^6^ | Serious  • publication bias was not assessed for this outcome. Search for unpublished research was not reported. | Very low to low | Critical |  |
| Pandey 2018^74^  Phoa 2014^81^ | Bleeding | Unclear  • insufficient information to judge^2^ | No serious limitations  • it is unknown if patients had other GE conditions^3^ | No serious limitations  • one study | Serious  • small sample size (n=136) with only one event in total^6^ | Serious  • publication bias was not assessed for this outcome. Search for unpublished research was not reported. | Very low to low | Critical |  |

1 Presentation of Downs and Black does not map well to risk of bias criteria. No additional information available for use.

2 Unclear if the Cochrane risk of bias tool and the Critical Appraisal Skills Programme checklist were actually used, and how 1-4 ranking was determined. No additional information available for use.

3 The reviews do not report if the patients had other GE conditions, which was part of the exclusion criteria for this overview, but the effect on treatment is expected to be minimal

4 Based on few events, CI for relative effects include both appreciable benefit and harm, and absolute CI reasonably includes appreciable benefit and harm.

5 Based on low study sample (sample size<rule of thumb of n=400 people)

6 Based on few events/ small study sample (sample size<rule of thumb of n=300 events)

7 Likely low study sample based on other outcomes for the same study (sample size<rule of thumb of n=400 people)

**Evidence Set 9: Thermal ablative technique combined with pharmacological therapy vs Thermal ablative technique combined with pharmacological therapy**

***Evidence Set 9.1 APC + PPI vs MPEC + PPI: Results table***

*Based on two primary studies: Dulai 2005*^82^*; Sharma 2006*^83^

| **9.1 Argon plasma coagulation (APC) + Proton Pump Inhibitor (PPI) vs Multipolar electrocoagulation (MPEC) + PPI** | | | | | | | | | |
| --- | --- | --- | --- | --- | --- | --- | --- | --- | --- |
| **Author Year** | **Outcome** | **Results: n/N** | | | | **Effect estimate (95% CI)** | **Absolute**  **Risk Difference (ARD)** | **AMSTAR† & GRADE‡** | **Notes** |
|  |  | **Study, Country** | **APC** | | **MPEC** |  |  |  |  |
| **All-cause mortality** | | | | | | | | | |
| Rees 2010^60^ | All-cause mortality | Dulai 2005^82^, NR | 0/24 | 0/24 | | OR not estimable | Not estimable | **AMSTAR:** Low  **Certainty:** Very low to low |  |
| **Stricture formation** ϯ | | | | | | | | | |
| Rees 2010^60^ | Stricture formation | Sharma 2006^83^, NR | 1/19  (5.3%) | 0/12 | | OR 2.03  (0.08, 53.87) | Not estimable | **AMSTAR:** Low  **Certainty:** Very low |  |

**†** see Supplementary table 1 for further details on AMSTAR domain ratings

‡ see Evidence Set 9.1: GRADE domains table for further details on GRADE domain ratings

ϯ post-hoc outcome

***Evidence Set 9.1 APC + PPI vs MPEC +PPI: GRADE domains table***

| **9.1 Argon plasma coagulation (APC) + PPI vs Multipolar electrocoagulation (MPEC) + PPI** | | | | | | | | | |
| --- | --- | --- | --- | --- | --- | --- | --- | --- | --- |
| **Review Studies** | **Outcome** | **Study Limitations** | **Indirectness** | **Inconsistency** | **Imprecision** | **Other considerations** | **Certainty** | **Importance** |  |
| Rees 2010^60^  Dulai 2005^82^ | All-cause mortality | Serious  • concern due to unclear assessment^1^ | Unclear  • country of conduct not reported which may affect the delivery of care^3^  • it is unknown if patients had other GE conditions^4^ | No serious limitations  • one study | Serious  • small sample size (n=52), CI not estimable, but likely wide^5^ | No serious limitations  • SR did not assess for publication bias. Although small study, comprehensive search and included search for unpublished literature. | Very low to low | Critical |  |
| Rees 2010^60^  Sharma 2006^83^ | Stricture formation | Serious  • some concerns due to assessments judged as unclear^2^ | Unclear  • country of conduct not reported which may affect the delivery of care^3^  • it is unknown if patients had other GE conditions^4^ | No serious limitations  • one study | Very serious  • small sample size (n=35) and very wide CI with only one event in total^6^ | No serious limitations  • SR did not assess for publication bias. Although small studies, comprehensive search and included search for unpublished literature. | Very Low | Critical; identified post hoc |  |

1 Although review authors rated this study at a low risk of bias, the support for judgement provided for allocation concealment did not meet the Cochrane criterion and should be deemed unclear. (Rees 2010 review)

2 Sequence generation, blinding (performance and detection bias), and attrition unclear (Rees 2010 review)

3 Country of conduct may affect the delivery of care in light of potential contextual influences such as a change in the accessibility to the regimens (for pharmacological treatment), and differences in training or equipment (procedural treatment).

4 The review does not report if the patients had other GE conditions, which was part of the exclusion criteria for this overview, but the effect on treatment is expected to be minimal

5 Based on few events/ small study sample (sample size<rule of thumb of n=300 events)

6 Based on few events and CI for relative effects include both appreciable benefit and harm. Absolute CI unknown.

***Evidence Set 9.2 MPEC + PPI vs APC + PPI: Results table***

*Based on two primary studies: Dulai 2005*^82^*; Sharma 2006*^83^

| **9.2 Multipolar electrocoagulation (MPEC) vs Argon plasma coagulation (APC)** | | | | | | | | | |
| --- | --- | --- | --- | --- | --- | --- | --- | --- | --- |
| **Author Year** | **Outcome** | **Results: n/N** | | | | **Effect estimate (95% CI)** | **Absolute**  **Risk Difference (ARD)** | **AMSTAR† & GRADE‡** | **Notes** |
|  |  | **Study, Country** | **MPEC + PPI** | | **APC + PPI** |  |  |  |  |
| **Complete ablation of Barrett’s esophagus** | | | | | | | | | |
| Li 2008^69^ | Histological complete ablation of BE | Dulai 2005^82^, NR | 21/26 (81%) | 17/26 (65%) | | Pooled OR 2.01 (0.77, 5.23) | ARD with intervention: 140 more per 1,000 (from 62 fewer to 260 more);  Risk with control: 644 per 1,000 | **AMSTAR:** Critically low  **Certainty:** Very low | Rees 2010 reports as complete eradication and provides percentage data only and does not clearly provide follow-up time, with discordant results for Dulai 2015. |
|  |  | Sharma 2006^83^, NR | 12/16 (75%) | 12/19 (63%) | |  |  |  |  |
| **Treatment failure** ϯ | | | | | | | | | |
| De Souza 2014^68^ | Treatment Failure (no ablation of BE) | Dulai 2005^82^, NR | 5/26  (19%) | 9/26  (35%) | | Pooled RD -0.14 (-0.33, 0.05),  I^2^: 0%  **RR 0.61  (0.30, 1.22) | ARD with intervention: 139 more per 1,000 (249 fewer to 78 more)  Risk with control: 356 per 1,000 | **AMSTAR:** Critically low  **Certainty:** Very low |  |
|  |  | Sharma 2006^83^, NR | 4/16  (25%) | 7/19  (37%) | |  |  |  |  |

**†** see Supplementary table 1 for further details on AMSTAR domain ratings

‡ see Evidence Set 9.2: GRADE domains table for further details on GRADE domain ratings

ϯ post-hoc outcome

Underlined first author name, publication year refers to a unique study included in more than one review.

***Evidence Set 9.2 MPEC vs APC: GRADE domains table***

| **Review Studies** | **Outcome** | **Study Limitations** | **Indirectness** | **Inconsistency** | **Imprecision** | **Other considerations** | **Certainty** | **Importance** |
| --- | --- | --- | --- | --- | --- | --- | --- | --- |
| Li 2008^69^  Dulai 2005^82^  Sharma 2006^83^ | Ablation | Serious  • some concerns due to assessments judged as unclear^1^ | Unclear  • country of conduct not reported which may affect the delivery of care^3^  • it is unknown if patients had other GE conditions^4^ | No serious limitations  • overlap of CIs, I^2^=0% | Serious  • small sample size (n=87)^5^ | Serious  • SR did not assess for publication bias, and the review did not perform comprehensive or grey lit searches | Very Low | Critical; identified post hoc |
| De Souza 2014^68^  Dulai 2005^82^  Sharma 2006^83^ | Treatment failure | Serious  • some concerns due to assessments judged as unclear^2^ | Unclear  • country of conduct not reported which may affect the delivery of care^3^  • it is unknown if patients had other GE conditions^4^ | No serious limitation  • I2=0%, overlap of CIs | Serious  • small sample size (n=87)^5^ | Serious  • comprehensive search not undertaken and uncertain about grey literature search | Very Low | Critical; identified post hoc |

1 Unclear assessment between the two studies in relation to sequence generation (Sharma 2006) and allocation concealment (Dulai 2006; based on authors’ supporting text in Rees 2010). It is unclear whether the lack of blinding detected in both studies using the Jadad tool corresponds directly to assessments of performance and detection biases.

2 Assessments made in De Souza 2014 review not adequately detailed. It is reasonable to consider that judgements made in footnote 1 pertain to this outcome.

3 Country of conduct may affect the delivery of care in light of potential contextual influences such as differences in training or equipment

4 The review does not report if the patients had other GE conditions, which was part of the exclusion criteria for this overview, but the effect on treatment is expected to be minimal

5 Based on few events/ small study sample (sample size<rule of thumb of n=300 events). CI includes little to no effect and appreciable benefit.

**Evidence Set 10: Thermal ablative technique vs Chemical ablative technique combined with pharmacological therapy**

***Evidence Set 10.1 PDT vs APC + PPI: results table***

*Based on five primary studies: Hage 2004*^86^*; Hage 2005*^85^*; Ragunath 2005*^88^*; Zoepf 2003*^89^ *(abstract); Zopf 2001*^90^

| **10.1 Photodynamic therapy (PDT) vs Argon plasma coagulation (APC) + Proton Pump Inhibitor (PPI)** | | | | | | | | | | | |
| --- | --- | --- | --- | --- | --- | --- | --- | --- | --- | --- | --- |
| **Author Year** | **Outcome** | **Results: n/N; Mean (SD)** | | | | **Effect estimate (95% CI)** | **Absolute**  **Risk Difference (ARD)** | | **AMSTAR† & GRADE‡** | **Notes** |  |
|  |  | **Study, Country** | **PDT** | **APC + PPI** | |  |  |  |  |  |  |
| **All-cause mortality** | | | | | | | | | | | |
| Rees 2010^60^ | All-cause mortality | Hage 2004^86^, NR | 1/26  (3.8%) | 0/14 | | OR 1.71  (0.07, 44.65) | Not estimable | | **AMSTAR:** Low  **Certainty:** Very low | Hage 2005 and Kelty 2004 use 5-ALA PDT and Ragunath 2005 uses porfimer sodium. |  |
|  |  | Kelty 2004^87^, NR | 0/34 | 0/34 | | OR not estimable |  |  |  |  |  |
|  |  | Ragunath 2005^88^, UK | 0/13 | 0/13 | | OR not estimable |  |  |  |  |  |
| **Progression to EAC** | | | | | | | | | | | |
| Almond 2014^84^ | Cancer incidence | Zopf 2001^90^, NR | 0/4 | 0/5 | | Not estimable | Not estimable | | **AMSTAR:** Critically low  **Certainty:** Very low |  |  |
|  |  | Hage 2004^86^, NR | 0/5 | 0/3 | | Not estimable |  |  |  |  |  |
|  |  | Ragunath 2005^88^, UK | 1/11  (9%) | 0/9 | | **RR 2.50 (0.11, 54.87) |  |  |  |  |  |
| **Progression to high-grade dysplasia** | | | | | | | | | | | |
| Almond 2014^84^ | Progression to high-grade dysplasia | Hage 2004^86^, NR | 0/4 | 0/5 | | Not estimable | Not estimable | | **AMSTAR:** Critically low  **Certainty:** Very low |  |  |
|  |  | Ragunath 2005^88^, UK | 0/5 | 0/3 | |  |  |  |  |  |  |
| **Eradication of dysplasia** ϯ | | | | | | | | | | | |
| Rees 2010^60^ | Complete eradication of dysplasia at 12 months | Ragunath 2005^88^, UK | *10/13  (77%) | 6/9  (67%) | | OR 1.67  (0.25, 11.07) | ARD with intervention: 103 more per 1,000 (from 333 fewer to 290 more);  Risk with control: 667 per 1,000 | | **AMSTAR:** Low  **Certainty:** Very low | Fayter 2010 reports that dysplasia eradication was statistically significantly better at 4 months, but not at 12 months, with PDT.  Li 2008 provides concordant data. |  |
| Almond 2014^84^ | Complete eradication of dysplasia at 12 months | Hage 2004^86^, NR | 5/5  (100%) | 3/3  (100%) | | NR  **RR 1.00  (0.64, 1.56) | ARD with intervention: 0 fewer per 1,000 (from 360 fewer to 560 more);  Risk with control: 1,000 per 1,000 | | **AMSTAR:** Critically low  **Certainty:** Very low |  |  |
|  |  | Ragunath 2005^88^, UK | *8/11  (73%) | 6/9  (67%) | | NR  **RR 1.09  (0.61, 1.96) | ARD with intervention: 60 more per 1,000 (from 260 fewer to 640 more);  Risk with control: 667 per 1,000 | | **AMSTAR:** Critically low  **Certainty:** Very low |  |  |
| **Complete ablation of Barrett’s Esophagus (BE)** | | | | | | | | | | | |
| Li 2008^69^ | Histologically complete ablation of BE | Kelty 2004^87^, NR | 13/35  (37%) | | 26/37  (70%) | ****RR 0.51 (0.34, 0.77)** | | ARD with intervention: 289 fewer per 1,000 (from 136 fewer to 390 fewer);  Risk with control: 590 per 1,000 | **AMSTAR:** Critically low  **Certainty:** Very low | Results in review are presented as APC+PPI vs PDT but restructured here to align with this table’s presentation. OR favours APC+PPI treatment.  The comparator is labelled as ALA-PDT and the intervention is labelled as APC alone in Li 2008.  Fayter 2010 reports no significant difference in rates of complete ablation between groups (Hage 2004). |  |
|  |  | Hage 2004^86^, NR | 5/26  (19%) | | 5/14  (36%) |  |  |  |  |  |  |
|  |  | Hage 2005^85^, NR | 4/19  (21%) | | 5/10  (50%) |  |  |  |  |  |  |
| **Eradication of Barrett’s Esophagus (BE)** | | | | | | | | | | | |
| Rees 2010^60^ | Complete eradication of BE at 12 months | Hage 2004^86^, NR | 18/21  (86%) | 8/12  ***(67%) | | Pooled OR 0.31  (0.00, 32.60), I^2^=91.5% | Moderate baseline risk:  ***ARD with intervention: 284 fewer per 1,000 (from --- to 315 more)  Risk with control: 670 per 1,000  High baseline risk:  ***ARD with intervention: 61 fewer per 1,000 (from --- to 29 more)  Risk with control: 970 per 1,000 | | **AMSTAR:** Low  **Certainty:** Very low | Fayter 2010 reports that treatment led to complete reversal of the columnar segment to squamous epithelium in 50% of patients receiving ALA–PDT and 97% of patients receiving APC (*p* < 0.0001). |  |
|  |  | Kelty 2004^87^, NR | 17/34  (50%) | 33/34  ***(97%) | |  |  |  |  |  |  |
|  |  | Ragunath 2005^88^, UK | *0/13 | *0/13 | |  |  |  |  |  |  |
| **Reduction of Barrett’s Esophagus (BE)** ϯ | | | | | | | | | | | |
| Rees 2010^60^ | Reduction in length (cm) of BE at 12  months | Ragunath 2005^88^, UK | 2.31 (1.75) (n=13) | 3.22 (1.3) (n=13) | | MD -0.91  (-2.10, 0.28) | MD 0.91cm lower (2.1cm lower to 0.28cm higher);  Risk with control: The mean reduction in length (cm) of BE at 12 months was 3.22cm | | **AMSTAR:** Low  **Certainty:** Low |  |  |
| Fayter 2010^70^ | BE surface reduction | Hage 2004^86^, NR | Both the group receiving fractionated-dose PDT with ALA and the group receiving APC had statistically significantly better results in terms of Barrett’s oesophagus surface reduction than the group receiving single-dose PDT. Differences between fractionated-dose PDT and APC were not significant. | | | | | | **AMSTAR:** Critically low  **Certainty:** Very low | Compares two different PDT doses to APC. |  |
| Li 2008^69^ | Length of regression (median) (endoscopic change) | Ragunath 2005^88^, UK | 57%  (4 month)  60%  (12 months) | 65%  (4 months)  56%  (12 months) | | Not estimable | Not estimable | | **AMSTAR:** Critically low  **Certainty:** Very low | Data provided by authors was the median percentage regression of BE without additional information. |  |
| Fayter 2010^70^ | Reduction in length | Zoepf 2003^89^, NR | 90% reduction for those undergoing ALA–PDT treatment than those receiving APC but fewer treatments were used for APC. | | | | | | **AMSTAR:** Critically low  **Certainty:** Very low to low | Zoepf 2003 abstract only. |  |
| **Eradication of intestinal metaplasia** | | | | | | | | | | |  |
| Almond 2014^84^ | Complete eradication of intestinal metaplasia | Ragunath 2005^88^, UK | 2/11 | 2/9 | | NR  **RR 0.82  (0.14, 4.71) | ARD with intervention: 40 fewer per 1,000 (from 191 fewer to 824 more);  Risk with control: 222 per 1,000 | | **AMSTAR:** Critically low  **Certainty:** Very low |  |  |
| **Treatment failure (no ablation)** ϯ | | | | | | | | | | | |
| De Souza 2014^45^ | Treatment Failure (no ablation of BE) | Hage 2004^86^, NR | 1/13  (7.8%) | 3/14  (21%) | | **Pooled RD 0.14 (0.02, 0.27),** I^2^=82%^Ϯ^  ****RR 1.72 (1.13, 2.61)** | Low baseline risk:  ARD with intervention: 79 more per 1,000 (14 more to 174 more)  Risk with control: 110 per 1,000  High baseline risk:  ARD with intervention: 612 more per 1,000 (from 110 more to 1,000 more)  Risk with control: 850 per 1,000 | | **AMSTAR:** Critically low  **Certainty:** Very low | Opposite of complete eradication of BE (reported above by Rees 2010), with the same three primary studies, with some discordance in group size.  The review labelled the comparator as APC alone.  PDT in Hage and Kelty were ALA-PDT but porfimer sodium PDT in Ragunath 2005. |  |
|  |  | Kelty 2004^87^, NR | 18/35  (51%) | 4/37  (11%) | |  |  |  |  |  |  |
|  |  | Ragunath 2005^88^, UK | 11/13  (85%) | 11/13  (85%) | |  |  |  |  |  |  |
| **Stricture formation** ϯ | | | | | | | | | | | |
| Rees 2010^60^ | Stricture formation | Hage 2004 ^86^, NR | 0/26 | 1/14  (7.1%) | | Pooled OR 0.51 (0.11, 2.44) | ARD with intervention: 31 fewer per 1,000 (from 58 fewer to 81 more);  Risk with control: 66 per 1,000 | | **AMSTAR:** Low  **Certainty:** Very low | Fayter 2010 reported that no major side effects in terms of perforations or strictures occurred in the trials (from Kelty 2004 only). |  |
|  |  | Kelty 2004^87^, NR | 0/34 | 1/34  (2.9%) | |  |  |  |  |  |  |
|  |  | Ragunath 2005^88^, UK | *2/13  (15%) | *2/13  (15%) | |  |  |  |  |  |  |
| Almond 2014^84^ | Stricture | Ragunath 2005^88^, UK | *2/11  (18%) | *1/9  (11%) | | NR  ** RR 1.64 (0.18, 15.26) | ARD with intervention: 71 more per 1,000 (from 91 fewer to 1,000 more);  Risk with control: 111 per 1,000 | | **AMSTAR:** Critically low  **Certainty:** Very low | Almond 2014 included only those with LGD. |  |

**†** see Supplementary table 1 for further details on AMSTAR domain ratings

‡ see Evidence Set 10.1: GRADE domains table for further details on GRADE domain ratings

ϯ post-hoc outcome

*discrepant data

Ϯ SR authors seem to have double counted the Hage 2004 data, therefore the MD and I^2^ may be different from what is presented. The ARD is calculated based on the RR (based on the three studies).

**the effect estimate was not reported in the original SR but calculated by the overview team

*** The ARD was not estimable for the pooled estimate because the lower 95% CI is 0.00. The calculated ARDs are, therefore, shown according to moderate and high baseline control group rates.

Bolded effect estimates refer to statistically significant results.

Underlined first author name, publication year refers to a unique study included in more than one review.

***Evidence Set 10.1 PDT vs APC + PPI: GRADE domains table***

| **10.1 Photodynamic therapy (PDT) vs Argon plasma coagulation (APC) + Proton Pump Inhibitor (PPI)** | | | | | | | | | |
| --- | --- | --- | --- | --- | --- | --- | --- | --- | --- |
| **Review Studies** | **Outcome** | **Study Limitations** | **Indirectness** | **Inconsistency** | **Imprecision** | **Other considerations** | **Certainty** | **Importance** |  |
| Rees 2010^60^  Hage 2004^86^  Kelty 2004^87^  Ragunath 2005^88^ | All-cause mortality | Serious  • some concern due to assessments judged as unclear^1^ | Unclear  • country of conduct not reported in two RCTs which may affect the delivery of care^13^  • it is unknown if patients had other GE conditions^14^ | No serious limitations  • CI only 1 study; few events across studies | Very serious  • small sample size (n=134), very wide CI with only one event in total^15^ | No serious limitations  • SR did not assess for publication bias. Although small studies, comprehensive search and included search for unpublished literature. | Very low | Critical |  |
| Almond 2014^84^  Zӧpf 2001^90^  Hage 2004^86^  Ragunath 2005^88^ | Cancer incidence | Serious  • some concern due to assessments judged as unclear^2^ | Unclear  • country of conduct not reported in two RCTs which may affect the delivery of care^13^  • it is unknown if patients had other GE conditions^14^ | No serious limitations  • CI not estimable because of zero events but is likely to overlap among studies. | Serious  • small sample size (n=37)^16^ | Serious  • SR did not assess for publication bias, and the review did not perform comprehensive or grey lit searches | Very low | Critical |  |
| Almond 2014^84^  Hage 2004^86^  Ragunath 2005^88^ | Progression to HGD | Serious  • some concern due to assessments judged as unclear^3^ | Unclear  • country of conduct not reported in one RCT which may affect the delivery of care^13^  • it is unknown if patients had other GE conditions^14^ | No serious limitations  • CI not estimable because of zero events but is likely to overlap between studies. | Serious  • small sample sizes (n=17). No events.^16^ | Serious  • SR did not assess for publication bias, and the review did not perform comprehensive or grey lit searches | Very low | Critical |  |
| Rees 2010^60^  Ragunath 2005^88^ | Complete eradication of dysplasia at 12 months | Serious  • some concerns due to assessments judged as unclear^4^ | Unclear  • country of conduct not reported which may affect the delivery of care^13^  • it is unknown if patients had other GE conditions^14^ | No serious limitations  • one study | Very serious  • small sample size (n=22), wide CI^17^ | No serious limitations  • SR did not assess for publication bias. Although small studies, comprehensive search and included search for unpublished literature. | Very low | Critical; identified post hoc |  |
| Almond 2014^84^    Hage 2004^86^ | Complete eradication of dysplasia at 12 months | Very serious  • concerns due to assessments judged as unclear and high risk^5,6^ | Unclear  • country of conduct not reported which may affect the delivery of care^13^  • it is unknown if patients had other GE conditions^14^ | No serious limitations  • one study, insufficient data | Very serious  • small sample (n=28)^18^ | Serious  • SR did not assess for publication bias, and the review did not perform comprehensive or grey lit searches | Very low | Critical |  |
| Almond 2014^84^    Ragunath 2005^88^ | Complete eradication of dysplasia at 12 months | Serious  • some concerns due to assessments judged as unclear^6^ | Unclear  • country of conduct not reported which may affect the delivery of care^13^  • it is unknown if patients had other GE conditions^14^ | No serious limitations  • one study | Very serious  • small sample (n=26)^17^ | Serious  • SR did not assess for publication bias, and the review did not perform comprehensive or grey lit searches | Very low | Critical |  |
| Li 2008^69^  Kelty 2004^87^  Hage 2004^86^  Hage 2005^85^ | Histologically complete ablation of BE | Very serious  • some concerns due to assessments judged as unclear and high risk^7^ | Unclear  • country of conduct not reported which may affect the delivery of care^13^  • it is unknown if patients had other GE conditions^14^ | No serious limitations  • overlap of CIs, I^2^=0% | Serious  • small sample size (n=141)^16^ | Serious  • SR did not assess for publication bias, and the review did not perform comprehensive or grey lit searches | Very low | Critical |  |
| Rees 2010^60^  Hage 2004^86^  Kelty 2004^87^  Ragunath 2005^88^ | Complete eradication of BE at 12 months | Serious  • some concerns due to assessments judged as unclear^8^ | Unclear  • country of conduct not reported in one RCT which may affect the delivery of care^13^  • it is unknown if patients had other GE conditions^14^ | Serious  • considerable heterogeneity (I^2^=91%, p=0.00061) unaccounted for, no overlap of CIs, variation in effect estimates | Very serious  • small sample size (n=134) and very wide CI^17^ | No serious limitations  • SR did not assess for publication bias. Although small studies, comprehensive search and included search for unpublished literature. | Very low | Critical; identified post hoc |  |
| Almond 2014^84^  Ragunath 2005^88^ | Complete Eradication-Intestinal Metaplasia | Serious  • some concerns due to assessments judged as unclear^6^ | No serious limitations  • it is unknown if patients had other GE conditions^14^ | No serious limitations  • one study | Very serious  • small sample size (n=20) only four events in total^17^ | Serious  • SR did not assess for publication bias, and the review did not perform comprehensive or grey lit searches | Very low | Critical |  |
| Rees 2010^60^  Ragunath 2005^88^ | Reduction in length (cm) of BE at 12 months | Serious  • some concerns due to assessments judged as unclear^4^ | No serious limitations  • it is unknown if patients had other GE conditions^14^ | No serious limitations  • one study | Serious  • small sample size (n=26), wide CI^19^ | No serious limitations  • SR did not assess for publication bias. Although small studies, comprehensive search and included search for unpublished literature. | Low | Critical; identified post hoc |  |
| Fayter 2010^70^  Hage 2004^86^ | BE surface reduction | Very serious  • some concerns due to assessments judged as unclear and high risk^9^ | Unclear  • country of conduct not reported which may affect the delivery of care^13^  • it is unknown if patients had other GE conditions^14^ | No serious limitations  • one study, insufficient data | Serious  • small sample size (n=40)^16^ | No serious limitations  • SR did not assess for publication bias. Although small studies, comprehensive search and included search for unpublished literature. | Very low | Critical |  |
| Li 2008^69^  Ragunath 2005^88^ | Length of regression (median) (endoscopic change) | Serious  • some concern due to assessments judged as unclear^10^ | No serious limitations  • it is unknown if patients had other GE conditions^14^ | No serious limitations  • one study | Serious  • small sample size (n=26)^20^ | Serious  • SR did not assess for publication bias, and the review did not perform comprehensive or grey lit searches | Very low | Critical  identified post hoc |  |
| Fayter 2010^70^  Zoepf 2003^89^  (abstract) | Reduction in length | Very serious to serious  • abstract^11^ | Unclear  • country of conduct not reported which may affect the delivery of care^13^  • it is unknown if patients had other GE conditions^14^ | No serious limitations  • one study | Serious  • small sample size (n=20)^20^ | No serious limitations  • SR did not assess for publication bias. Although small studies, comprehensive search and included search for unpublished literature. | Very low to low | Critical |  |
| De Souza 2014^45^  Hage 2004^86^  Kelty 2004^87^  Ragunath 2005^88^ | Treatment failure (no ablation of BE) | Serious  • some concerns due to assessments judged as unclear^12^ | Unclear  • country of conduct not reported in two RCTs which may affect the delivery of care^13^  • it is unknown if patients had other GE conditions^14^ | Serious  • considerable heterogeneity (I^2^=82%, p=0.0007) and some variation in overlap of CI | Serious  • small sample size (n=125)^16^ | Serious  • comprehensive search not undertaken and uncertain about grey literature search | Very low | Critical; identified post hoc |  |
| Rees 2010^60^  Hage 2004^86^  Kelty 2004^87^  Ragunath 2005^88^ | Stricture formation | Serious  • some concerns due to assessments judged as unclear^8^ | Unclear  • country of conduct not reported in two RCTs which may affect the delivery of care^13^  • it is unknown if patients had other GE conditions^14^ | No serious limitations | Very serious  • small sample size (n=134)^17^ | No serious limitations  • SR did not assess for publication bias. Although small studies, comprehensive search and included search for unpublished literature. | Very low | Critical; identified post hoc |  |
| Almond 2014^84^  Ragunath 2005^88^ | Stricture formation | Serious  • some concerns due to assessments judged as unclear^6^ | No serious limitations  • it is unknown if patients had other GE conditions^14^ | No serious limitations  • one study | Very serious  • few events, small sample size (n=20)^17^ | Serious  • SR did not assess for publication bias, and the review did not perform comprehensive or grey lit searches | Very low | Critical; identified post hoc |  |

1 80% of evidence from studies at unclear risk of bias: allocation concealment, performance bias, and attrition common between studies; remaining study at high risk of bias because of blinding (performance bias). We did not consider detection bias at risk because of the objective nature of the outcome. (Rees 2010 assessment)

2 Assessments made in the Almond 2014 review not adequately described. Using assessments made in Rees 2010, 75% of evidence (two studies) is at unclear risk for allocation concealment, and assessment made for unclear risk of attrition bias for other outcomes may apply to this outcome. Additionally, 22% of evidence (one study) was unclear for sequence generation and high risk for performance and detection bias.

3 Assessments made in the Almond 2014 not adequately described. Using assessments made in Rees 2010, evidence at unclear risk for allocation concealment, and assessment made for unclear risk of attrition bias for other outcomes may apply to this outcome. Additionally, around half of evidence was unclear for sequence generation and high risk for performance and detection bias.

4 Allocation concealment, blinding and attrition unclear (Rees 2010 assessment)

5 High risk for performance and detection bias, unclear remaining domains (Rees 2010 assessment)

6 Assessments made in the Almond 2014 review not adequately described. Using assessments made in Rees 2010, unclear risk for allocation concealment; unclear assessments for performance and detection bias and attrition may be applicable to this outcome.

7 As the primary assessments are made with the Jadad tool (Li 2008 review), information from the Cochrane assessment (Rees 2010 review) was also used for context. Information on Hage 2005 is limited with only Jadad assessment available; it might reasonable to infer study conduct was similar as Hage 2004. High risk of performance and detection bias in 49% of the evidence, with high or unclear in the remainder. Allocation concealment is unclear across the evidence base, and the randomization method is unclear in 49% of the evidence.

8 80% of evidence from studies at unclear risk of bias: allocation concealment, performance and detection bias, and attrition common between studies; remaining study at high risk of bias because of blinding (performance and detection bias). (Rees 2010 assessment).

9 Assessments made in the Fayter 2010 review were provided in aggregate. Using assessments from Rees 2010, high risk of performance bias and detection bias seem reasonable to consider for this outcome. Remaining domains were at unclear risk.

10 Based on both Jadad (Li 2008) and Cochrane (Rees 2010) assessments, unclear risk for allocation concealment and performance and detection biases.

11 Assessments made on a study available only in abstract form; the risk of bias could be serious or very serious but unlikely to be at low risk; information provided was only in aggregate.

12 Assessments made in the De Souza review not adequately described. Using assessments made in the Rees 2010 review, 80% of evidence from studies at unclear risk of bias: allocation concealment, performance and detection bias, and attrition common between studies; remaining study at high risk of bias because of blinding (performance and detection bias).

13 Country of conduct may affect the delivery of care in light of potential contextual influences such as a change in the accessibility to the regimens (for pharmacological treatment), and differences in training or equipment (procedural treatment).

14 The reviews do not report if the patients had other GE conditions, which was part of the exclusion criteria for this overview, but the effect on treatment is expected to be minimal

15 Based on few events and CI for relative effects include both appreciable benefit and harm. Absolute CI unknown.

16 Based on few events/ small study sample (sample size<rule of thumb of n=300 events)

17 Based on few events, CI for relative effects include both appreciable benefit and harm, and absolute CI reasonably includes appreciable benefit and harm.

18 Based on few events/ small study sample (sample size<rule of thumb of n=300 events). CI includes little to no effect and appreciable benefit.

19 Based on low study sample (sample size<rule of thumb of n=400 people).

20 Based on few events/ small study sample (sample size<rule of thumb of n=300 events). Insufficient reporting of information to judge extent of imprecision of data.

**Evidence Set 11: Mechanical ablative technique vs Thermal ablative technique**

***Evidence Set 11.1 EMR vs RFA***

*Based on one primary study in abstract: van Vilsteren 2011*^95^

| **11.1 Endoscopic mucosal resection (EMR) vs Radiofrequency ablation (RFA)** | | | | | | | | |
| --- | --- | --- | --- | --- | --- | --- | --- | --- |
| **Author Year** | **Outcome** | **Results: n/N (%)** | | | **Effect estimate (95% CI)** | **Absolute**  **Risk Difference (ARD)** | **AMSTAR† & GRADE‡** | **Notes** |
|  |  | **Study, Country** | **EMR** | **RFA** |  |  |  |  |
| **Eradication of cancer** ϯ | | | | | | | | |
| Desai 2017^93^ | Complete eradication of neoplasia | van Vilsteren 2011^95^, The Netherlands/Germany | 25/25  (100%) | NR/22 (96%) | NR | Not estimable | **AMSTAR:** Critically low  **Certainty**: Very low | Comparisons labelled as stepwise (complete) EMR compared to focal-EMR + RFA. Same primary study as in Chadwick 2014 and Fujii-Lau 2017. |
| **Eradication of dysplasia** ϯ | | | | | | | | |
| Chadwick 2014^91^ | Complete eradication of dysplasia (end of treatment) | van Vilsteren 2011^95^, The Netherlands/Germany | 25/25 (100%) | 21/22 (95%) | NR  **RR 1.05  (0.93, 1.18) | ARD with intervention: 48 more per 1,000 (from 67 fewer to 172 more);  Risk with control: 955 per 1,000 | **AMSTAR:** Critically low  **Certainty**: Very low | Intervention labelled as EMR and comparator as RFA. |
| Chadwick 2014^91^ | Complete eradication of dysplasia with no recurrence at follow-up | van Vilsteren 2011^95^, The Netherlands/Germany | 25/25 (100%) | 21/22 (95%) | NR  **RR 1.05  (0.93, 1.18) | ARD with intervention: 48 more per 1,000 (from 67 fewer to 172 more);  Risk with control: 955 per 1,000 | **AMSTAR:** Critically low  **Certainty**: Very low |  |
| **Eradication of intestinal metaplasia (IM)** ϯ | | | | | | | | |
| Desai 2017^93^ | Complete eradication of IM | van Vilsteren 2011^95^, The Netherlands/Germany | *NR/25  (92%) | *NR/22 (92%) | NR | Not estimable | **AMSTAR:** Critically low  **Certainty**: Very low | Text and table differ on CE-IM rates for s-EMR group (20/25 in text). |
| Chadwick 2014^91^ | Complete eradication of IM (end of treatment) | van Vilsteren 2011^95^, The Netherlands/Germany | *24/25 (96%) | *21/22 (95%) | NR  **RR 1.01  (0.89, 1.14) | ARD with intervention: 10 more per 1,000 (from 105 fewer to 134 more);  Risk with control: 955 per 1,000 | **AMSTAR:** Critically low  **Certainty**: Very low | *Percentages are discrepant between reviews but results overall are concordant. |
| Chadwick 2014^91^ | Complete eradication of IM with no recurrence at follow- up | van Vilsteren 2011^95^, The Netherlands/Germany | 24/25 (96%) | 21/22 (95%) | NR  **RR 1.01  (0.89, 1.14) | ARD with intervention: 10 more per 1,000 (from 105 fewer to 134 more);  Risk with control: 955 per 1,000 | **AMSTAR:** Critically low  **Certainty**: Very low |  |
| **Recurrence of EAC** | | | | | | | | |
| Fujii-Lau 2017^92^ | Early neoplasia recurrence after complete eradication | van Vilsteren 2011^95^, The Netherlands/Germany | 1/25 | 0/22 | Not estimable  **RR 2.65  (0.11, 62.00) | Not estimable | **AMSTAR:** Critically low  **Certainty:** Very low | Intervention labelled as stepwise complete endoscopic resection (s-EMR) in this review. |
| **Recurrence of dysplasia** ϯ | | | | | | | | |
| Fujii-Lau 2017^92^ | Dysplasia recurrence after achieving complete eradication | van Vilsteren 2011^95^, The Netherlands/Germany | 0/25 | 0/22 | Not estimable^¥^ | Not estimable | **AMSTAR:** Critically low  **Certainty**: Low | Recurrence after achieving complete eradication of IM following endoscopic eradication therapy |
| **Recurrence of intestinal metaplasia (IM)** ϯ | | | | | | | | |
| Desai 2017^93^ | Recurrence of IM (follow up) | van Vilsteren 2011^95^, The Netherlands/Germany | 3/25  (12%) | NR/22 | NR | Not estimable | **AMSTAR:** Critically low  **Certainty**: Very low |  |
| Fujii-Lau 2017^92^ | IM recurrence | van Vilsteren 2011^95^, The Netherlands/Germany | 2/25 | 2/22 | **RR 0.88  (0.14, 5.73)  Incidence rate^¥¥^ | ARD with intervention: 11 fewer per 1,000 (from 78 fewer to 430 more)  Risk with control: 91 per 1,000 | **AMSTAR:** Critically low  **Certainty**: Very low | Recurrence after achieving complete eradication of IM following endoscopic  eradication therapy |
| **Bleeding** ϯ | | | | | | | | |
| Chadwick 2014^91^ | Acute bleeding endoscopically treated | van Vilsteren 2011^95^, The Netherlands/Germany | 5/25 (20%)* | 2/22 (9.1%)* | NR  **RR 2.20  (0.47, 10.23) | ARD with intervention: 109 more per 1,000 (from 48 fewer to 839 more);  Risk with control: 91 per 1,000 | **AMSTAR:** Critically low  **Certainty:** Very low |  |
| Desai 2017^93^ | Bleeding | van Vilsteren 2011^95^, The Netherlands/Germany | 6/25 (24%)* | 3/22 (13.6%)* | NR  ** RR 1.76  (0.50, 6.22) | ARD with intervention: 104 more per 1,000 (from 68 fewer to 712 more);  Risk with control: 136 per 1,000 | **AMSTAR:** Critically low  **Certainty:** Very low | *Discrepant data between van Vilsteren 2011 in Chadwick 2014 and Desai 2017 but results overall are concordant. |
| **Perforations** ϯ | | | | | | | | |
| Chadwick 2014^91^ | Number of perforations | van Vilsteren 2011^95^, The Netherlands/Germany | 1/25 (4%) | 0/22 | Not estimable  **RR 2.65  (0.11, 62.00) | Not estimable | **AMSTAR:** Critically low  **Certainty:** Very low | Desai 2017 reports the same results. |
| **Stricture** ϯ | | | | | | | | |
| Desai 2017^93^ | Stricture | van Vilsteren 2011^95^, The Netherlands/Germany | 22/25 (88%) | 3/22 (13.6%) | NR  ****RR 6.45**  **(2.23, 18.66)** | ARD with intervention: 743 more per 1,000 (from 168 more to 1,000 more);  Risk with control: 136 per 1,000 | **AMSTAR:** Critically low  **Certainty:** Very low |  |
| **Stenosis requiring treatment** ϯ | | | | | | | | |
| Chadwick 2014^91^ | Stenosis requiring treatment (with a median of 3 dilatations; all had large ERs before RFA) | van Vilsteren 2011^95^, The Netherlands/Germany | 22/25 (88%) | 3/21 (14%) | NR  **** RR 6.16**  **(2.14, 17.74)** | 737 more per 1,000 (from 163 more to 1,000 more);  Risk with control:143 per 1,000 | **AMSTAR:** Critically low  **Certainty:** Very low |  |

**†** see Supplementary table 1 for further details on AMSTAR domain ratings

‡ see Evidence Set 11.1: GRADE domains tables further details on GRADE domain ratings

ϯ post-hoc outcome

* discordance between reviews

**the effect estimate was not reported in the original SR but calculated by the overview team

^¥^ Between groups comparison was not reported but only per arm data reported: Incidence of recurrence (95% CI) per 100 person-year: s-EMR: 1.9 (0.0, 5.6), p-value 0.32. RFA: 1.3 (0.0, 4.9), p-value 0.48

^¥^ Between groups comparison was not reported but only per arm data reported: Incidence of recurrence (95% CI) per 100 py: after EMR: 3.8, 95% CI (0.0, 9.1) p-value 0.16 after RFA: 5.3, 95% CI (0.0, 12.6), p-value 0.16Bolded effect estimates refer to statistically significant results.

Underlined first author name, publication year refers to a unique study included in more than one review.

***Evidence Set 11.1 EMR vs RFA: GRADE domains table***

| **11.1 Endoscopic mucosal resection (EMR) vs Radiofrequency ablation (RFA)** | | | | | | | | | |
| --- | --- | --- | --- | --- | --- | --- | --- | --- | --- |
| **Review**  **Studies** | **Outcome** | **Study Limitations** | **Indirectness** | **Inconsistency** | **Imprecision** | **Other considerations** | **Certainty** | **Importance** |  |
| Desai 2017^93^  van Vilsteren 2011^95^ | Complete eradication of neoplasia | Serious  • mix of risk of bias across outcomes, some information missing^1^ | No serious limitations  • it is unknown if patients had other GE conditions^3^ | No serious limitations  • one study | Serious  • small sample size (n=47)^4^ | Serious  • although review performed grey literature searches, a comprehensive search was not performed | Very low | Critical |  |
| Chadwick  2014^91^  van Vilsteren 2011^95^ | Complete eradication of dysplasia (end of treatment) | Serious  • mix of risk of bias across outcomes, some information missing^2^ | No serious limitations  • it is unknown if patients had other GE conditions^3^ | No serious limitations  • one study | Serious  • small sample size (n=47)^5^ | Serious  • SR did not assess for publication bias, and although the review performed grey literature searches, a comprehensive search was not performed | Very low | Critical |  |
| Chadwick  2014^91^  van Vilsteren 2011^95^ | Complete eradication of dysplasia with no recurrence at follow-up | Serious  • mix of risk of bias across outcomes, some information missing^2^ | No serious limitations  • it is unknown if patients had other GE conditions^3^ | No serious limitations  • one study | Serious  • small sample size (n=47)^5^ | Serious  • SR did not assess for publication bias, and although the review performed grey literature searches, a comprehensive search was not performed | Very low | Critical |  |
| Desai 2017^93^  van Vilsteren 2011^95^ | Complete eradication of intestinal metaplasia | Serious  • mix of risk of bias across outcomes, some information missing^1^ | No serious limitations  • it is unknown if patients had other GE conditions^3^ | No serious limitations  • one study | Serious  • small sample size (n=47)^4^ | Serious  • although review performed grey literature searches, a comprehensive search was not performed | Very low | Critical |  |
| Chadwick 2014^91^  van Vilsteren 2011^95^ | Complete eradication of intestinal metaplasia (end of treatment) | Serious  • mix of risk of bias across outcomes, some information missing^2^ | No serious limitations  • it is unknown if patients had other GE conditions^3^ | No serious limitations  • one study | Serious  • small sample size (n=47)^5^ | Serious  • SR did not assess for publication bias, and although the review performed grey literature searches, a comprehensive search was not performed | Very low | Critical |  |
| Chadwick 2014^91^  van Vilsteren 2011^95^ | Complete eradication of intestinal metaplasia with no recurrence at follow-up | Serious  • mix of risk of bias across outcomes, some information missing^2^ | No serious limitations  • it is unknown if patients had other GE conditions^3^ | No serious limitations  • one study | Serious  • small sample size (n=47)^5^ | Serious  • SR did not assess for publication bias, and although the review performed grey literature searches, a comprehensive search was not performed | Very low | Critical |  |
| Fujii-Lau 2017^92^  van Vilsteren 2011^95^ | Early neoplasia recurrence after complete eradication | Serious  • mix of risk of bias across outcomes, some information missing^2^ | No serious limitations  • it is unknown if patients had other GE conditions^3^ | No serious limitations  • one study | Serious  • small sample size (n=47) with only one event in total^4^ | Serious  • publication bias detected | Very low | Critical; post hoc |  |
| Fujii-Lau 2017^92^  van Vilsteren 2011^95^ | Dysplasia recurrence after achieving complete eradication | Serious  • mix of risk of bias across outcomes, some information missing^2^ | No serious limitations  • it is unknown if patients had other GE conditions^3^ | No serious limitations  • one study | Serious  • small sample size (n=47) with no events^4^ | No serious limitations  • not detected by review authors | Low | Critical; post hoc |  |
| Desai 2017^93^  van Vilsteren 2011^95^ | Recurrence of IM (follow-up) | Serious  • mix of risk of bias across outcomes, some information missing^1^ | No serious limitations  • it is unknown if patients had other GE conditions^3^ | No serious limitations  • one study | Serious  • small sample size (n=47)^4^ | Serious  • although review performed grey literature searches, a comprehensive search was not performed | Very low | Critical; post hoc |  |
| Fujii-Lau 2017^92^  van Vilsteren 2011^95^ | Intestinal Metaplasia recurrence | Serious  • mix of risk of bias across outcomes, some information missing^2^ | No serious limitations  • it is unknown if patients had other GE conditions^3^ | No serious limitations  • one study | Very serious  • small sample size (n=47) with only four events in total^6^ | No serious limitations  • not detected by review authors | Very low | Critical; post hoc |  |
| Chadwick 2014^91^  van Vilsteren 2011^95^ | Acute bleeding endoscopically treated | Serious  • mix of risk of bias across outcomes, some information missing^2^ | No serious limitations  • it is unknown if patients had other GE conditions^3^ | No serious limitations  • one study | Very serious  • small sample size (n=47) with only seven events in total^6^ | Serious  • SR did not assess for publication bias, and although the review performed grey literature searches, a comprehensive search was not performed | Very Low | Critical |  |
| Desai 2017^93^  van Vilsteren 2011^95^ | Bleeding | Serious  • mix of risk of bias across outcomes, some information missing^1^ | No serious limitations  • it is unknown if patients had other GE conditions^3^ | No serious limitations  • one study | Very serious  • small sample size (n=47) with only nine events in total^6^ | Serious  • although review performed grey literature searches, a comprehensive search was not performed | Very Low | Critical |  |
| Chadwick 2014^91^  van Vilsteren 2011^95^ | Number of perforations | Serious  • mix of risk of bias across outcomes, some information missing^2^ | No serious limitations  • it is unknown if patients had other GE conditions^3^ | No serious limitations  • one study | Serious  • small sample size (n=47) with only one event in total^4^ | Serious  • SR did not assess for publication bias, and although the review performed grey literature searches, a comprehensive search was not performed | Very Low | Critical |  |
| Desai 2017^93^  van Vilsteren 2011^95^ | Stricture | Serious  • mix of risk of bias across outcomes, some information missing^1^ | No serious limitations  • it is unknown if patients had other GE conditions^3^ | No serious limitations  • one study | Serious  • small sample size (n=47)^5^ | Serious  • although review performed grey literature searches, a comprehensive search was not performed | Very Low | Critical; post hoc |  |
| Chadwick 2014^91^  van Vilsteren 2011^95^ | Stenosis requiring treatment | Serious  • mix of risk of bias across outcomes, some information missing^2^ | No serious limitations  • it is unknown if patients had other GE conditions^3^ | No serious limitations  • one study | Serious  • small sample size (n=47)^5^ | Serious  • SR did not assess for publication bias, and although the review performed grey literature searches, a comprehensive search was not performed | Very Low | Critical |  |

1 Information provided in Desai 2017 review not adequately detailed and information only given for the s-EMR group Tools other than Cochrane used in other reviews for this study; information from across reviews considered together. Unclear sequence generation (Chadwick 2014); concealment and blinding of patients (performance bias) could be at either high or unclear risk based on reporting (Chadwick 2014, Fujii-Lau 2017).

2 No reviews evaluating this study used the Cochrane risk of bias tool; therefore, information was considered from across reviews together. Unclear randomization method (Chadwick 2014); concealment and blinding of patients (performance bias) could be at either high or unclear risk based on reporting (Fujii-Lau 2017; Chadwick 2014).

3 The reviews do not report if the patients had other GE conditions, which was part of the exclusion criteria for this overview, but the effect on treatment is expected to be minimal

4 Based on few events/ small study sample (sample size<rule of thumb of n=300 events). Insufficient reporting of information to judge extent of imprecision of data.

5 Based on few events/ small study sample (sample size<rule of thumb of n=300 events).

6 Based on few events, CI for relative effects include both appreciable benefit and harm, and absolute CI reasonably includes appreciable benefit and harm.
