# Supplementary material for: Screening for esophageal adenocarcinoma and precancerous conditions (dysplasia and Barrett’s esophagus) in patients with chronic gastroesophageal reflux disease with or without other risk factors: two systematic reviews and one overview of reviews to inform a guideline of the Canadian Task Force on Preventive Health Care (CTFPHC)
Source: Syst Rev. 2020 Jan 29;9:20. doi: 10.1186/s13643-020-1275-2 (PMC6990541; doi:10.1186/s13643-020-1275-2)
Supplement: Supplementary file 13 — Additional file 13: GRADE considerations and decisions for the overview of reviews (KQ3). [file 13643_2020_1275_MOESM13_ESM.docx]

# Additional file 13. GRADE considerations and decisions for the overview of reviews (KQ3)

## Study limitations domain

The GRADE Handbook outlines several criteria that are likely to result in biased results in randomized trials: randomization/concealment; blinding; attrition; selective reporting; and other limitations, such as the use of unvalidated outcome measures for patient-reported outcomes [1].

Different critical appraisal criteria were used across reviews, including the Cochrane RoB tool. Unlike other tools, the Cochrane RoB tool addresses the GRADE criteria directly. Since the Cochrane RoB criteria correspond perfectly to the GRADE criteria, we have elected to present all critical appraisal information across reviews according to those criteria, to facilitate judgements for the study limitations domain.

To optimize the use of relevant information for a given study, we considered available Cochrane RoB information, either as a primary source or together with assessments made with another tool, to inform a judgement. We regarded study-level Cochrane RoB assessments as relevant to any reporting of a study. Details on how this information was considered is provided by outcome in the footnotes of the KQ3: GRADE evidence sets 1-11.

In cases where information relevant to the study limitations criteria was not available or not reported in a way to enable its use, the study limitations domain was labelled as ‘unclear’, and no judgement was made on whether to down-rate (see final GRADE rating for further details).

In a few cases where the body of evidence was a mix of abstract and full report information (e.g., KQ3: Evidence Set 7.2), we provide a range of potential assessments, reflective of the uncertainty in the collective risk of bias information. Aligning with assessments made in an included Cochrane review, conference abstracts were deemed to possess very serious limitations due to their preliminary nature (also used for abstract data alone), and the RoB information for the full reports was provided in aggregate in these cases, making it uncertain to know the contribution of an individual study in the analysis.

## Indirectness domain

Evaluating directness was more difficult, owing to our reliance on review authors’ reporting of study information. When evaluating indirectness, two factors were considered:

- Country of conduct: Of particular importance, as the delivery of care in some jurisdictions may not be directly applicable to the Canadian context and, therefore, may impact the understanding of the applicability of treatment effectiveness. This could impact pharmacological treatment as it may impact accessibility to the regimens. It may also impact procedural and surgical treatment, as there may be differences in training or equipment used. Therefore, when the country of conduct was included it was assessed against the Canadian context to determine if down-rating was necessary. For example, if a trial was conducted in the USA, care delivery for these interventions was thought not to differ from the Canadian context, so down-rating did not occur. If information on the country of conduct was missing, indirectness was not rated, but labelled as ‘unclear’.
- Other gastroesophageal conditions (GE): None of the included SRs provided any information as to whether the participants had other GE conditions, an *a priori* determined exclusion criterion. Although important to note in the GRADE tables as having been considered, it was judged to have minimal effect, and indirectness was not down-rated.

## Imprecision

Imprecision was judged based on GRADE default thresholds for optimal information size (300 events for dichotomous outcomes and 400 patients for continuous outcomes) and interpretation of confidence intervals according to whether results include no effect, appreciable benefit, and/or appreciable harm (benefit/harm threshold RR<0.75 and RR>1.25, along with consideration of the absolute confidence interval). Clinical significance of estimates was difficult to determine for many outcomes and addressed in the Discussion section.

## Final GRADE rating

As all primary studies in the reviews were RCTs, each outcome started with a high level of certainty. If there was sufficient down-rating to very low certainty (i.e., three levels of down-rating) among domains with sufficient information for assessment, any unclear domain(s) would be inconsequential as no further rating changes are possible. However, if the certainty of the evidence was low, moderate, or high after rating the domains with sufficient information, an unclear domain may impact the certainty of the evidence. For example, if the rating (based on GRADE domains with known evidence) was a low level of certainty, and there was one domain that was unclear, having sufficient information to rate the domain could result in one of two situations: 1. A rating of no serious limitations would result in a final level of certainty of low (no change); or 2. A rating of serious or very serious would result in a final level of certainty of very low (one additional down-rating). To reflect this uncertainty, we have provided the range of possible certainty rating (i.e., very low to low).
