# Supplementary material for: Screening for esophageal adenocarcinoma and precancerous conditions (dysplasia and Barrett’s esophagus) in patients with chronic gastroesophageal reflux disease with or without other risk factors: two systematic reviews and one overview of reviews to inform a guideline of the Canadian Task Force on Preventive Health Care (CTFPHC)
Source: Syst Rev. 2020 Jan 29;9:20. doi: 10.1186/s13643-020-1275-2 (PMC6990541; doi:10.1186/s13643-020-1275-2)
Supplement: Supplementary file 14 — Additional file 14: Characteristics tables [file 13643_2020_1275_MOESM14_ESM.docx]

**Additional file 14. Table of characteristics of included studies and reviews**

**Table 1. KQ 1: Table of characteristics of included studies**

| **Study year Country** | **Study design (total sample)** | **Data collection** | **Intervention & Comparator(s)**  **(n allocated)**  **Setting** | **Population** | | | | **Outcome(s)** | |
| --- | --- | --- | --- | --- | --- | --- | --- | --- | --- |
|  |  |  |  | **GERD**^^[[1]](#footnote-1)^^ **definition** | **Inclusion/Exclusion criteria** | **Patient characteristics** |  | |  |
| Chak 2014^55^ USA | Randomized Controlled Trial (n=184) | Not reported  **Follow-up:** one time test | Transnasal esophagoscopy (TNE)^^[[2]](#footnote-2)^^ (n=92)  Video capsule esophagoscopy (VCE)^^[[3]](#footnote-3)^^ (n=92)  Outpatient clinic | Symptoms of GERD (from questionnaire^^[[4]](#footnote-4)^^) or use of acid suppression medicine (within 7 days of screening) | **Inclusion**: Veterans aged 45-85 years, without a prior EGD in the past 10 years and with no contraindications to VCE or TNE (history of recurrent epistaxis), with or without GERD.  **Exclusion**: Altered nasopharyngeal anatomy, allergy to lidocaine derivatives, bleeding diathesis, prolonged prothrombin time, known swallowing disorders, having a cardiac pacemaker or other eletromechanical implants, suspected gastrointestinal obstruction, strictures, or fistulas based on clinical picture. | Mean (SD) age: 59 (8) years  Males: 96%  White ethnicity: 41%  PPI therapy: 46%  Smokers: 80%  Mean (SD) BMI:  TNE: 31.25 (7.66)  ECE: 31.38 (6.98) | Endoscopically suspected BE  Histologically confirmed BE  Anxiety, nervousness, or worry before and during the procedure | |  |
| Chang 2011^51^  USA | Randomized Controlled Trial (n=60) | Feb 2009 - May 2010  **Follow-up:** one-time test | Transnasal esophagoscopy T(NE)^^[[5]](#footnote-5)^^ (n=20)  Video capsule esophagoscopy (VCE)^2^ (n=20)  EGD^^[[6]](#footnote-6)^,^[[7]](#footnote-7)^^ (n=20)  Clinical research centre | Symptoms obtained through three validated questionnaires^,4,^[[8]](#footnote-8)^,^[[9]](#footnote-9)^^ | **Inclusion**: Patients over 50 years old from the Olmsted Country cohort, who had not undergone sedated EGD in the past 5 years  **Exclusion**: History of known BE, upper endoscopy within past 5 years, history of dysphagia, Zenker diverticulum, epiphrenic diverticulum, known or suspected intestinal obstruction, cardiac pacemaker, any implanted electromedical device, pregnancy, any MRI anticipated within 7 days, any abdominal surgery within previous 6 months (except cholecystectomy), history of recurrent epistaxis, no longer an Olmsted Country, Minnesota resident, deceased, any serious illness that may impair ability to complete a questionnaire, any contraindication to esophageal biopsy | Age: NR  Males: 55%  White ethnicity: NR  PPI therapy: NR  Smokers: NR  Mean (SD) BMI: NR | Endoscopically suspected BE  Histologically confirmed BE  Dysplasia | |  |
| Ferguson 2006^57^ USA | Randomized Controlled Trial (n=137) | Not reported  **Follow-up:** one time test | EGD + biopsy^^[[10]](#footnote-10)^^ (n=69)  Enhanced magnification-directed endoscopy^^[[11]](#footnote-11)^^ biopsies (with acetic acid) (n=68)  Outpatient clinic | Validated questionnaire,^^[[12]](#footnote-12)^^ GERD score | **Inclusion**: Patients presenting for EGD with a history of GERD  **Exclusion**: Patients with a known history of esophageal cancer, BE with dysplasia, esophagectomy, previous endoscopic ablation therapy, contraindication to procurement of biopsies, or those with an allergy to acetic acid | Age:  EGD: 62.8 y (15.0)  EME: 62.2 y (15.6)  Male: 42%  White ethnicity: NR  PPI therapy: NR  Smokers: NR  Mean (SD) BMI: NR | Specialized Intestinal Metaplasia | |  |
| Sami 2015^52^ USA | Randomized Controlled Trial (n=209) | Apr 2011 - Oct 2013  **Follow-up:** one time test | EGD^^[[13]](#footnote-13)^^ (n=61)  Hospital-based transnasal esophagoscopy^1^ (n=72) Hospital outpatient endoscopy suite  Mobile-based transnasal esophagoscopy^1^ (n=76)  Mobile research vehicle | GERQ questionnaire^4^ and defined as “heartburn or acid regurgitation ≥1 week, <1 week, or none” | **Inclusion**: Subjects ≥50 years of age from the Olmsted Country cohort, who previously completed validated gastrointestinal symptom questionnaires from 1988 to 2009  **Exclusion**: History of progressive dysphagia or recurrent epistaxis, known Zenker’s or epiphrenic diverticulum, moved out of Olmsted County or deceased, significant illness that may impair ability to complete questionnaires, and coagulopathy. | Mean (SD) age: 65 y (9)  Males: 46%  White ethnicity: 99%  PPI therapy: 17%  Smokers: NR  BMI mean (SD): EGD: 28.8 (5.8)  hTNE: 30.5 (13.9)  mTNE: 29.0 (5.6) | Endoscopically suspected BE  Histologically confirmed BE  Anxiety experienced during the procedure  Serious adverse events | |  |
| Wani 2014^58^ India | Randomized Controlled Trial (n=378) | Jan 2010-Feb 2012  **Follow-up:** one time test | EGD^^[[14]](#footnote-14)^^ + biopsy^10^ (n=33)  EGD^9^ + chromo-endoscopy^^[[15]](#footnote-15)^^ (n=23)  Setting not reported | Described as “characteristics symptoms of GERD”. | **Inclusion**: Patients with GERD from five northern states of India  **Exclusion**: Not reported | Mean (SD) age: 48.15 y (10.9)  Males: 66.7%  White ethnicity: NR  PPI therapy: NR  Smokers: NR  Mean (SD) BMI: NR | Histologically confirmed BE (Specialized Intestinal Metaplasia) | |  |
| Zaman 1999^56^ USA | Randomized Controlled Trial (n=105) | Not reported  **Follow-up:** one time test | Unsedated transnasal endoscopy (T-EGD)^^[[16]](#footnote-16)^^  (n=29)^^[[17]](#footnote-17)^^  Unsedated peroral endoscopy (P-EGD)^16^  (n=31)^17^  Hospital gastroenterology clinics and endoscopy unit | not defined | **Inclusion**: Upper gastrointestinal symptoms.  **Exclusion**: Acute gastrointestinal hemorrhage, history of sinus surgery or recurrent sinusitis, any current anticoagulation therapy, dysphagia possibly requiring esophageal dilation, a need for sclerotherapy or band ligation of varices, or surveillance for Barrett’s esophagus. | Mean age (range): 46 y (21-84)  Males: 58%  White ethnicity: NR  PPI therapy: NR  Smokers: NR  Mean (SD) BMI: NR | Life threatening, severe, or medically significant consequence  Endoscopically suspected BE  Anxiety before procedure, during insertion, and during procedure | |  |
| Jobe 2006^53^ USA | Randomized crossover study  (n=134) | Mar 2004-Mar 2005  **Follow-up:** one time test for each modality | EGD + biopsy^10^ then  transnasal esophagoscopy^^[[18]](#footnote-18)^^ (n=62)  Transnasal esophagoscopy then EGD + biopsy (n=72)  Office-based screening performed in randomized order 1 week to 1 month later in all patients. | Heartburn, regurgitation or dysphagia | **Inclusion**: Outpatients ≥18 years who were scheduled for endoscopic screening for symptoms of chronic GERD and all patients ≥18 years of age with histologically proven BE who were enrolled in endoscopic surveillance  **Exclusion**: Patients with a history of prior antireflux surgery, endoscopic antireflux procedure, Zenker’s diverticulum, epiphrenic diverticulum, pregnancy, anticoagulation therapy, esophageal varices, history of recurrent epistaxis, or head or neck malignancy | Median age (IQR): 59 y (51-71)  Males: 80%  White ethnicity: 95%  Antisecretory medication: 78%  Smokers: 73%  Mean (SD) BMI: 30.1 (5.9) | Histologically confirmed BE  Dysplasia (low- and high-grade)  Esophageal adenocarcinoma  Anxiety before procedure, during insertion, and during procedure | |  |
| Mori 2010^54^ Japan | Prospective cohort  (n=1580) | Not reported  **Follow-up:** one time test | EGD^^[[19]](#footnote-19)^^ (n=254)  Ultrathin nasal EGD^5^ (n=727)^^[[20]](#footnote-20)^^  Ultrathin oral EGD^5^ (n=599)  Hospital setting | Not defined | **Inclusion**: Consecutive outpatients who underwent EGD for screening upper intestinal tract disorders in Inuyama Chuo Hospital and Ichinomiya Nishi Hospital  **Exclusion**: Patients with esophagogastric cancers, gastroduodenal ulcers, and histories of esophagogastrointestinal surgery as well as those taking proton-pump-inhibitors or histamine-2 receptor antagonists. | Mean (SD) age: 60 y (16)  Males: 50%  White ethnicity: NR  PPI therapy: NR  Smokers: NR  Mean (SD) BMI: NR | Endoscopically suspected BE | |  |
| Rubenstein 2008^49^  USA | Retrospective controlled cohort study (n=155) | 1995-2003  **Follow-up**: none or prior EGD in the past 5 years, depending on patient | EGD (n=25)  no prior EGD (n=130) | GER identified by International Classification of Diseases codes 530.10-530.12, 530.81, or 787.1 | **Inclusion**: Veterans diagnosed with adenocarcinoma of the distal third of the esophagus or of the gastric cardia from 1995 through 2003, and who had gastroesophageal reflux diagnosed before the diagnosis of cancer  **Exclusion**: Subjects who did not have at least one admission or outpatient encounter in each of the 5 years before the cancer diagnosis, those with dysplasia but with no evidence of EAC, and subjects without EAC (such as gastric cardia adenocarcinoma, which shares the same ICD code as EAC) (based on the review of the electronic medical records). | Age: NR  Males: 99%  White ethnicity: 84%  PPI therapy: NR  Smokers: NR  Mean (SD) BMI: NR | Esophageal adenocarcinoma stage at diagnosis  Long-term survival | |  |
| Hammad 2018^50^  USA | Retrospective cohort study (n=153*)  *excludes 29 patients under surveillance for BE | Feb 2005- Sept 2017  **Follow-up**: none or prior EGD in the past 5 years, depending on patient | No EGD (n=152)  EGD <5 years ago (n=1) | Not defined | **Inclusion**: Patients diagnosed with EAC between February 2005 to September 2017.  **Exclusion**: Not reported | Mean age: 67.5 y (SD 9.3)  Males: 99.3%  White ethnicity: 84.3%  PPI therapy: 47.7%  Any anti-acid: 58.8%  Active smokers: 43.1%  Ex-smoker: 41.2%  Mean (SD) BMI: 30 (6.5) | Esophageal adenocarcinoma stage at diagnosis | |  |

**Table 2. KQ 2: Table of characteristics of included studies**

| **Study year Country**  **Funding** | **Setting** | **Patient flow and timing of outcome data collection** | **Population** | | | **Intervention & Comparator(s)** |
| --- | --- | --- | --- | --- | --- | --- |
|  |  |  | **GERD^^[[21]](#footnote-21)^^** **definition** | **Inclusion/Exclusion criteria** | **Patient characteristics providing outcome data^^[[22]](#footnote-22)^^** |  |
| Chak 2014^55^  USA  U.S. Public Health Service | Outpatient clinic | 1210 patients were asked to participate in a RCT. 1026 patients did not participate prior to randomization. The 184 who agreed to participate were given the option to withdraw after randomization. Seven patients withdrew - these patients were asked to fill out a non-completion questionnaire to ascertain reasons for non-completion. | Symptoms of GERD (from questionnaire^^[[23]](#footnote-23)^^) or use of acid suppression medicine (within 7 days of screening) | **Inclusion**: Veterans aged 45-85 years, without a prior EGD in the past 10 years and with no contraindications to VCE or transnasal esophagoscopy (history of recurrent epistaxis), with or without GERD.  **Exclusion**: Altered nasopharyngeal anatomy, allergy to lidocaine derivatives, bleeding diathesis, prolonged prothrombin time, known swallowing disorders, having a cardiac pacemaker or other eletromechanical implants, suspected gastrointestinal obstruction, strictures, or fistulas based on clinical picture. | Not reported | Transnasal esophagoscopy^^[[24]](#footnote-24)^^  Video capsule esophagoscopy^^[[25]](#footnote-25)^^ |
| Zaman 1999^56^  USA  NR | Hospital gastroenterology clinics and endoscopy unit over a 12- month period | 105 consecutive patients were asked to participate in a RCT. 45 patients refused participation and provided reasons why. | Not defined | **Inclusion**: Upper gastrointestinal symptoms.  **Exclusion**: Acute gastrointestinal hemorrhage, history of sinus surgery or recurrent sinusitis, any current anticoagulation therapy, dysphagia possibly requiring esophageal dilation, a need for sclerotherapy or band ligation of varices, or surveillance for Barrett’s esophagus. | Not reported | Unsedated transnasal endoscopy^^[[26]](#footnote-26)^^  Unsedated peroral endoscopy^^[[27]](#footnote-27)^^ |
| Zaman 1998^59^  USA  NR | Hospital gastroenterology clinics and endoscopy units over a 6-month period | 62 patients were asked to participate in a study where they would receive peroral endoscopy followed by standard sedated EGD. Those who refused participation were asked to provide their reasons. | Not defined | **Inclusion**: Patients being evaluated for upper gastrointestinal symptoms  **Exclusion**: Evidence of acute gastrointestinal hemorrhage | Not reported | Unsedated peroral endoscopy^7^  Standard sedated EGD^^[[28]](#footnote-28)^^ |

**Table 3. KQ 3: Table of characteristics of included systematic reviews**

| **Author Year, Country**  **Funding**  **COI** | **Date of last search; Databases searched**  **Included studies** | **Total population of SR** | **Primary Studies†** | **Comparisons**  **(number of trials)** | **Outcomes** | **AMSTAR Rating** |
| --- | --- | --- | --- | --- | --- | --- |
| Pandey  2018^74^, UK  Funding: NR  COI: None | May 2017;  Ovid MEDLINE, EMBASE, and Web of Science  2 RCTs, 6 observational cohort studies (3 prospective design) | 619 Adult patients diagnosed with low grade  Dysplasia (Barrett’s esophagus-associated low grade dysplasia receiving RFA) | *Phoa 2014, Shaheen 2009* | - RFA vs surveillance (n=2) | - Progression to high grade dysplasia - Complete eradication of intestinal metaplasia - Complete eradication of dysplasia - Stricture formation - Perforation | Critically Low |
| Codipilly 2018^104^, USA  Funding: Public Health Service award, NIH award, and NIH grant  COI: Yes, Declared | September 2017;  MEDLINE,  Cochrane CENTRAL, SCOPUS, Web of Science, PubMed, and  Ovid EMBASE  1 ongoing RCT, 1 Case-control, 17 cohort studies (included in the quantitative synthesis + additional 3 cohort excluded from the quantitative analysis) | 3,400 BE patients (1700 in each group) in one ongoing RCT, the Barrett’s Oesophagus Surveillance Study (BOSS), in BE patients | *BOSS trial* | - Surveillance versus No Surveillance (n=1 ongoing RCT) | Not applicable as it included an ongoing RCT with no results available | Critically Low |
| Almond 2014^84^, UK  Funding: NR  COI: None declared | January 2013; MEDLINE, Embase  6 RCTs (37* studies: cohort, case series): 3 RCTs providing data | 90 patients with a diagnosis of low-grade dysplasia using any form of endoscopic therapy. Of these, 36 patients provided comparative data. | *Bright 2007*, *Dulai 2005*, Hage 2004, Ragunath 2005, *Shaheen 2011*, Zopf 2001 | - PDT vs APC (n=3) - MPEC vs NR (n=1) - APC vs NR (n=1) - RFA vs NR (n=1) | - Incident cancers - Progression to HGD | Critically low |
| Chadwick 2014^91^, UK  Funding: NR  COI: None declared | January 2013; PubMed, Embase, Cochrane Library  3 RCTs (22 total studies: cohort) | 47 adults with Barrett’s esophagus with HGD or intramucosal cancer  n=42 in Shaheen 2009 and n=61 in Shaheen 2011 (update) | *Shaheen 2011* (*follow-up of Shaheen 2009*), van Vilsteren 2011  Only RFA group data is presented in Shaheen 2009 and 2011 | - Complete EMR + Triple therapy vs RFA + Triple therapy (n=1) - *RFA + PPI vs sham + PPI (n=2)* | - Recurrence of intramucosal cancer - Complete eradication of: dysplasia, intestinal metaplasia with no recurrence | Critically low |
| De Souza 2014^68^, Brazil  Funding: NR  COI: NR | NR; Pubmed, Embase, LILACS, Cochrane Library  9 RCTs | 649 adults with Barrett’s esophagus comparing various modalities of endoscopic therapy for BE or endoscopic ablation treatment vs PPI. | Ackroyd 2000, Ackroyd 2004, Dulai 2005, Hage 2004, Kelty 2004, Overholt 2005, Ragunath 2005, Shaheen 2009, Sharma 2006 | - PDT vs APC (n=3) - MPEC vs APC (n=2) - PDT vs PPI (n=2) - APC vs PPI (n=1) - RFA vs PPI (n=1) | - Treatment failure | Critically low |
| Desai 2017^93^, USA  Funding: NR  COI: None declared | June 2016; PubMed, Embase, Cochrane Library, Web of Science  1 RCT (20 studies: cohorts) | 47 patients with Barrett’s esophagus related neoplasia (HGD/EAC) who underwent either f-EMR + RFA or stepwise (or complete) EMR with intent of complete eradication of BE related neoplasia. | van Vilsteren 2011 | - Stepwise (complete) EMR vs focal-EMR + RFA (n=1) | - Recurrence of: EAC, dysplasia, intestinal metaplasia - Complete eradication of: neoplasia, intestinal metaplasia | Critically low |
| Fayter 2010^70^,  UK  Funding: NIHR  COI: NR | October 2008; MEDLINE, Embase, CINAHL, PASCAL, LILACS, Cochrane Library  11 RCTs | 594 adults with Barrett’s esophagus, adenocarcinoma (no data of interest on EAC population) | *Ackroyd 1996*, Ackroyd 2000, Hage 2004, Kelty 2004, Kelty 2004b, Mackenzie 2007, Mackenzie 2008, Mackenzie 2009, Overholt 2007, Ragunath 2005, Zoepf 2003 | - ALA-PDT vs placebo PDT (n=2) - ALA-PDT vs APC (n=3) - PDT with porfimer sodium vs APC (n=1) - PDT with porfimer sodium + PPI vs PPI alone (n=1) - PDT delivery comparisons (n=4) | - All-cause mortality - Eradication of dysplasia - Complete ablation/remission of: dysplasia, BE - Reduction/regression of: BE - Progression to cancer - Many outcomes were reported narratively | Critically low |
| Fujii-Lau 2017^92^, USA  Funding: NR  COI: (1) | May 2016; PubMed, Embase, Web of Science  2 RCTs (39 studies: cohort, case series) | 22 patients who achieved complete eradication of intestinal metaplasia after treatment with endoscopic eradication therapies (EMR, RFA or a combination of both) | *Shaheen 2011*, van Vilsteren 2011  Only RFA group data is presented in Shaheen 2011 | - Stepwise complete EMR vs RFA (n=1) - RFA vs sham (n=1) | - Recurrence of esophageal cancer - Complete eradication of: dysplasia, intestinal metaplasia with no recurrence | Critically low |
| Li 2008^69^, China  Funding: NR  COI: NR | Date NR; Pubmed, Embase, Cochrane Library  13 RCTs; however: 12 of them are providing data | 747 patients who had BE validated by pathology review who were treated with therapeutic treatment modalities. | Ackroyd 2000, Bright 2007 (*update of Ackroyd 2004*), Dulai 2005, Hage 2004, Hage 2005, Kelty 2004, Peters 1999, Overholt 2007 (update of Overholt 2005), Parrilla 2003, Ragunath 2005, Sharma 2006 | - Anti-reflux surgery vs Omeprazole (n=1) - PPI vs H2 Receptor Antagonists (n=1) - PDT vs PPI (n=3) - Anti-reflux surgery +APC vs Anti-reflux surgery + surveillance (n=2) - APC vs PDT (n=4) - APC vs MPEC (n=2) | - Progression to: cancer, dysplasia, HGD - Eradication of: dysplasia, HGD - Complete ablation of BE - Regression of BE (length, area) | Critically low |
| Qumseya 2017^73^, USA  Funding: No financial support  COI: (2) | December 2015; Medline, Embase, Cochrane Library  2 RCTs (19 studies: prospective studies, four national registries, and retrospective analyses) | 199 patients with Barrett’s esophagus with LGD treated with RFA (with or without EMR) or surveillance. | Phoa 2014, Shaheen 2009 | - RFA vs surveillance (n=2) | - Progression to cancer - Progression to HGD | Low |
| Rees 2010^60^, UK  Medical Research Council  COI: (3) | June 2008; MEDLINE, Embase, Cochrane Library  16 RCTs: 15 providing data  Overholt 2007 was used to supplement Overholt 2005. | 1074 adults whom the diagnosis of BE has been established both endoscopically and confirmed histologically, regardless of the status of dysplasia. | Ackroyd 2000, Bright 2007, Caldwell 1996, Dulai 2005, Hage 2004, Heath 2007, *Luman 1996*, Kelty 2004, Overholt 2005, Mackenzie 2008, Parrilla 2003, Peters 1999, Ragunath 2005, Shaheen 2008, Sharma 2006, Weinstein 1996 | - PPI vs H2RA (n=3) - Celecoxib vs placebo (n=1) - Surgery vs PPI/ H2RA (n=1) - APC vs surveillance (n=1) - APC w/ PPI vs MPEC w/ PPI (n=2) - APC w/ PPI vs PDT (n=3) - PDT w/ PPI vs PPI (n=2) - PDT (5-ALA) vs PDT (Porfimer sodium) (n=1) - RFA w/PPI vs PPI (n=1) | - All-cause mortality - Progression to: cancer, dysplasia - Complete eradication of: dysplasia, BE - Reduction/regression of: BE (length, area) | Low |

† italicized studies do not provide any data in the results of this overview of reviews.

(1) Authors have received funding from CSA Medical, Covidien, C2Therapeutic, CDx Medical, and Interpace Diagnostics

(2) Authors have received funding from Olympus, Ninepoint Medical, Medtronic, Cook Inc, Boston Scientific, Medtronic, C2Therapeutics, Erbe Medical

(3) Authors have received funding from Medical Research Council, Royal College of Surgeons of Edinburgh, Cancer Research UK, Astra Zeneca. Past collaborations with Merck and GlaxoSmithKline

* SR authors state that four studies were identified from a single publication and the original references could not be obtained.

**Table 4. KQ 3: Characteristics of primary studies in included reviews**

| **Author Year, Country** | **Intervention & Comparator** | **Participant characteristics** | | | | |
| --- | --- | --- | --- | --- | --- | --- |
|  |  | **Participants** | **Sex (m/f)** | **Age** | **Race** | **GE** |
| Ackroyd 2000^66^, NR | **Intervention (n=18)**: Photodynamic therapy with 5-Aminolevulinic acid + Proton pump inhibitor (Omeprazole 20 mg od and laser (green light 514 nm) per 3 cm)  **Comparator (n=18)**: Proton pump inhibitor (Omeprazole 20 mg od) | 36 individuals with BE and confirmed low grade dysplasia | PDT+PPI: 15/3  PPI: 15/3 | **median (range) in years**  PDT+PPI: 56 (30–71)  PPI: 54 (42–68) | NR | NR |
| Ackroyd 2004^72^, NR | **Intervention**: Argon plasma coagulation  **Comparator**: Endoscopic surveillance + PPI | 40 individuals with BE (2 with LGD) who had undergone antireflux surgery | APC: 15/5  Surveillance: 17/3 | **median (range) in years**  APC: 47 (41–57) Surveillance: 51 (38–59) | NR | NR |
| Bright 2007^71^, NR  *(long-term follow-up of patients in Ackroyd 2004)* | **Intervention (n=20)**: Post-surgery  Argon plasma coagulation (up to 6 treatments)  **Comparator (n=20)**: Surveillance with PPI | 40 individuals with BE (one with low grade dysplasia)  *(Almond 2014 includes only LGD)* | APC: 15/5 Surveillance: 17/3 | **median (range)** **in years**  APC: 56.5 (43–67) Surveillance: 58.3 (42-79) | NR | NR |
| Caldwell 1996^62^, NR  *(published in abstract)* | **Intervention**: Omeprazole 20 mg od  **Comparator**: Cimetidine 300 mg tds | 20 individuals (28 entered the study) | NR | NR | NR | NR |
| Dulai 2005^82^, NR | **Intervention (n=26)**: Argon plasma coagulation + pantoprazole 40 mg bd  **Comparator (n=26)**: Multipolar electrocoagulation + pantoprazole 40 mg bd pantoprazole inc. if symptomatic or persistent oesophagitis | 52 individuals with BE (one with LGD)  *(Almond 2014 includes only LGD)* | APC: 21/5  MPEC: 18/8 or 23/3 *(differs in Li and Rees)* | **mean (SD) in years**  APC: 58 (11)  MPEC: 56 (11) | NR | NR |
| Hage 2004^86^, NR | **Intervention (n=14)**: Argon plasma coagulation (65 w)  **Comparator (n=26)**: 5-Aminolevulinic acid Photodynamic therapy 60 mg/kg (100 J/cm2) or 5-ALA PDT 60 mg/kg (high dose 100 + 20 J/cm2 divided) administration regime) | 40 individuals: 32 with BE and eight with low grade dysplasia  *(Almond 2014 includes only LGD)* | APC: 11/3  PDT: 20/6 | **median (range)** **in years**  APC: 60 (41–69)  PDT: unknown (52–72) | NR | NR |
| Hage 2005^85^, NR | **Intervention (n=10)**: Argon plasma coagulation  **Comparator (n=19)**: Photodynamic therapy | 29 individuals: 16 with IM, five with LGD and eight with HDG | APC: 7/3  PDT: 16/3 | **median (range)** **in years**  APC: 54.5 (37–74)  PDT: 59 (44–79) | NR | NR |
| Heath 2007^61^, USA | **Intervention (n=49)**: Celecoxib 200 mg twice daily or placebo twice daily for at least a year and a maximum of 2 years  **Comparator (n=51)**: Placebo | 100 individuals | NR | NR | NR | NR |
| Kelty 2004a^87^, NR | **Intervention (n=37)**: Argon plasma coagulation (65 W) + Proton pump inhibitor  **Comparator (n=35)**: Aminolevulinic acid-Photodynamic therapy (85 J/cm2) + Proton pump inhibitor | 68 individuals with BE with dysplasia (72 entered the study) | APC + PPI: 30/7  PDT + PPI: 28/7 | **median (range) in years**  APC + PPI: 59 (28–79)  PDT + PPI: 61 (33–83) | NR | NR |
| Kelty 2004b^80^, NR  *(might be a subgroup of Kelty 2004a)* | **Intervention & Comparator**: Aminolevulinic acid- Photodynamic therapy at 30 mg/kg or 60 mg/kg at 4- or 6-hour incubation times or with  fractionated illumination | 25 individuals without dysplasia | 58/14 | NR | NR | NR |
| Mackenzie 2007^78^, NR  *(published in abstract)* | **Intervention & Comparator**: Aminolevulinic acid-Photodynamic therapy with varying doses of light and comparing red or green light | 72 individuals with HGD | NR | NR | NR | NR |
| Mackenzie 2008^77^, NR  *(published in abstract)* | **Intervention (n=16)**: Aminolevulinic acid-Photodynamic therapy 60 mg/kg, activated by 1178 J/cm of red laser light  **Comparator (n=16)**: Photodynamic therapy with Porfimer sodium (standard protocol (no more details))  Follow up with quadrantic biopsies every 2 cm at 6 weeks, 4 months and 1 year post-therapy | 32 (40 recruited) individuals with HGD | NR | NR | NR | NR |
| Mackenzie 2009^79^, NR  *(full publication of Mackenzie 2007)* | **Intervention**: Aminolevulinic acid- Photodynamic therapy with red light at 30 or 60 mg/kg  **Comparator**: Aminolevulinic acid- Photodynamic therapy with green light at 30 or 60 mg/kg | 27 individuals with HGD | NR | NR | NR | NR |
| Overholt 2005^67^, NR | **Intervention (n=138)**: Photodynamic therapy (130 J/cm2) after 2 mg/kg porfimer sodium, using diffuser with centring balloon. Focal nodules pretreated with 50 J/cm2 PDT with bare fibre with omeprazole 20 mg bd  **Comparator (n=70)**: Proton pump inhibitor (Omeprazole 20 mg bd) | 208 individuals with high grade dysplasia | PDT + PPI: 117/21  PPI: 59/11 | **mean (SD) in years**  PDT + PPI: 66.1 (10.7)  PPI: 67.3 (11.0) | NR | NR |
| Overholt 2007^65^, NR  *(combined with Overholt 2005 in Li 2008 as it presents the 5-year follow-up)* | As above | As above | As above | As above | NR | NR |
| Parrilla 2003^76^, NR | **Intervention**: Surgery (Short Nissen 56 or Collis Nissen 2) with no acid suppression  **Comparator**: Acid suppression (ranitidine 1982 to 1992 omeprazole 20 mg 1992 to 2000) | 101 individuals (113 entered the study): 93 with intestinal metaplasia and eight with low grade dysplasia | Surgery: 39/19  Acid suppression: 33/10 | **median (range) in years**  Surgery: 43 (10–71)  Acid suppression: 50 (12–78) | NR | NR |
| Peters 1999^63^, NR | **Intervention**: Ranitidine 150 mg bd  **Comparator**: Omeprazole 20 mg bd | 61 individuals with BE; 53 completed the study | Ranitidine: 20/10  Omeprazole: 23/8 | **median (range) in years**  Ranitidine: 56 (51-60.5)  Omeprazole: 58 (53.5-62) | NR | NR |
| Phoa 2014^81^, The Netherlands | **Intervention (n=68)**: Radio frequency ablation  **Comparator (n=68)**: Endoscopic surveillance | 136 individuals with LGD | RFA: 55/13  Surveillance: 61/7 | **mean (SD) in years**  RFA: 63 (10)  Surveillance: 63 (9) | NR | NR |
| Ragunath 2005^88^, USA | **Intervention (n=13)**: Argon Plasma Coagulation at a power setting of 65 W and argon gas flow at 1.8 l/min in 1 to 6 sessions (mean 5)  **Comparator (n=13)**: Photodynamic therapy performed 48 hours after intravenous injection of porfimer sodium 2 mg/kg with a 630 nm red laser light, 200 J/cm through a PDT balloon in 1 session | 26 individuals: 23 with LGD and 3 with HGD  *(Almond 2014 includes only LGD)* | APC: 13/0  PDT: 11/2 | **median (range) in years**  APC: 55 (35–79)  PDT: 64 (41–86) | NR | NR |
| Shaheen 2009^75^, USA | **Intervention (n=78)**: Radio frequency ablation 40 W/cm2 and 12 J/cm2; repeat RFA at 2, 4, 9 months if residual BE) + high-dose Proton pump inhibitor (40 mg bd)  **Comparator (n=39)**: Sham + high-dose Proton pump inhibitor (40 mg bd) | 117 individuals (59 LGD and 58 HGD) (127 enrolled)  *(Pandey 2018 and Qumseya 2017 only include patients with LGD (n=64 and n=63))* | RFA + PPI: 33/9  Sham + PPI: 18/3  *(based on 63 LGD patients in Qumseya 2017)* | **mean (SD) in years**  RFA + PPI: 65.9 (1.4)  Sham + PPI 64.6 (1.9)  *(based on 63 LGD patients in Qumseya 2017)* | NR | NR |
| Sharma 2006^83^, NR | **Intervention (n=16)**: Multipolar electrocoagulation 20 W continuous power  **Comparator (n=19)**: Argon plasma coagulation (60W gas flow 1.4 to 1.8 L/min) | 35 individuals (3 with LGD) | 34 male  1 female | **median (range) in years**  MPEC: 60 (42–68)  APC: 65 (32–84) | NR | NR |
| van Vilsteren 2011^95^, The Netherlands/ Germany | **Intervention (n=22)**: focal endoscopic mucosal resection + Radiofrequency ablation  **Comparator (n=25)**: stepwise endoscopic mucosal resection | 47 individuals with HGD and EAC | f-EMR + RFA: 19/3  s-EMR: 21/4 | **median (range or IQR*) in years**  f-EMR + RFA: 69 (55-73)  s-EMR: 68 (45-88) | NR | NR |
| Weinstein 1996^64^, NR | **Intervention**: Acid suppression with ranitidine (150 mg bd) for 2 years  **Comparator**: Omeprazole 80 mg for 1 year, then 40 mg in second year | 106 individuals with Barrett’s esophagus | NR | NR | NR | NR |
| Zoepf 2003^89^, NR  *(published in abstract)* | **Intervention**: Aminolevulinic acid-Photodynamic therapy  **Comparator**: Argon plasma coagulation | 20 individuals with mixed levels of dysplasia | NR | NR | NR | NR |
| Zӧpf 2001^90^, NR | **Intervention (n=4)**: Photodynamic therapy  **Comparator (n=5)**: Argon plasma coagulation | 9 individuals with LGD | NR | NR | NR | NR |

* it is unclear if this was reported as the range or the IQR

ALA: Aminolevulinic acid; APC: Argon plasma coagulation; BE: Barrett’s esophagus; EMR: Endoscopic Mucosal Resection; GE: Other gastro-esophageal conditions; MPEC: Multipolar electrocoagulation; NR: not reported; PDT: Photodynamic Therapy; PPI: Proton Pump Inhibitors; RFA: Radiofrequency Ablation

1. Gastro-esophageal Reflux Disease [↑](#footnote-ref-1)
2. Performed with Vision Sciences disposable sheath TNE-5000 digital esophagoscope [↑](#footnote-ref-2)
3. Performed with the Given Imaging PillCam ESO 2 capsule endoscopy [↑](#footnote-ref-3)
4. Locke GR, Talley NJ, Weaver AL, et al. A new questionnaire for gastroesophageal reflux disease. Mayo Clin Proc. 1994; 69:539–47 [↑](#footnote-ref-4)
5. Performed with Fujinon EG-530N endoscope [↑](#footnote-ref-5)
6. Esophagogastroduodenoscopy [↑](#footnote-ref-6)
7. Performed with PENTAX Medicalvideo endoscope [↑](#footnote-ref-7)
8. Talley NJ, Phillips SF, Melton J III, Wiltgen C, Zinsmeister AR. A patient questionnaire to identify bowel disease. Ann Intern Med 1989; 111 :671-674. [↑](#footnote-ref-8)
9. Talley NJ, Phillips SF, Wiltgen CM, Zinmeister AR, Melton LJ III. Assessment of functional gastrointestinal disease: the bowel disease questionnaire. Mayo Clin Proc 1990; 65: 1456-1479. [↑](#footnote-ref-9)
10. Conventional four-quadrant random biopsies taken every 2 cm [↑](#footnote-ref-10)
11. Performed with the Olympus GIF-Q160-Z endoscope [↑](#footnote-ref-11)
12. Ofman J, Shaw M, Sadik K, et al. Identifying patients with gastroesophageal reflux disease: Validation of a practical screening tool. Dig Dis Sci 2002; 47: 1863-9. [↑](#footnote-ref-12)
13. Performed with the Olympus GIF-180 high definition endoscope [↑](#footnote-ref-13)
14. Performed with Olympus GIF-Q180 video endoscope [↑](#footnote-ref-14)
15. Done with methylene blue directed biopsies [↑](#footnote-ref-15)
16. Olympus N200 and N230 ultrathin endoscopes [↑](#footnote-ref-16)
17. 4 patients randomized to transnasal required crossover to the peroral group. One crossover also could not complete the peroral screening and received endoscopy under general anesthesia. [↑](#footnote-ref-17)
18. Transnasal esophagoscopy performed with Olympus 5.1 mm diameter flexible endoscope. Sedated EGD performed with Olympus 9.8 mm diameter flexible endoscope [↑](#footnote-ref-18)
19. Performed with Olympus GIF-XQ240 or XQ260. Appears to be unsedated. [↑](#footnote-ref-19)
20. 25 patients chose N-EGD but had failed transnasal intubation and were converted to transoral EGD [↑](#footnote-ref-20)
21. Gastro-esophageal Reflux Disease [↑](#footnote-ref-21)
22. We only collected data on the population that is providing relevant outcome information (e.g., reasons for not participating in screening) [↑](#footnote-ref-22)
23. Locke GR, Talley NJ, Weaver AL, et al. A new questionnaire for gastroesophageal reflux disease. Mayo Clin Proc. 1994; 69:539–47 [↑](#footnote-ref-23)
24. Performed with Vision Sciences disposable sheath TNE-5000 digital esophagoscope [↑](#footnote-ref-24)
25. Performed with the Given Imaging PillCam ESO 2 capsule endoscopy [↑](#footnote-ref-25)
26. Performed with the Olympus N200 or N230 ultrathin endoscopes (N200 is the original name, and the later model is N230). A 6-mm diameter upper endoscope [↑](#footnote-ref-26)
27. Performed with the Olympus XGIF-N200H. A 6-mm ultrathin (UT) video endoscope [↑](#footnote-ref-27)
28. Performed with the Olympus GIF-100. A 9.5 mm diameter upper endoscope [↑](#footnote-ref-28)
