# Supplementary material for: Screening for esophageal adenocarcinoma and precancerous conditions (dysplasia and Barrett’s esophagus) in patients with chronic gastroesophageal reflux disease with or without other risk factors: two systematic reviews and one overview of reviews to inform a guideline of the Canadian Task Force on Preventive Health Care (CTFPHC)
Source: Syst Rev. 2020 Jan 29;9:20. doi: 10.1186/s13643-020-1275-2 (PMC6990541; doi:10.1186/s13643-020-1275-2)
Supplement: Supplementary file 15 — Additional file 15: ROB and quality appraisal results [file 13643_2020_1275_MOESM15_ESM.docx]

**Additional file 15. RoB and quality appraisal results**

**Table 1. KQ1: Cochrane ROB for RCTs**

| **Author/Year** | **Sequence generation** | **Allocation Concealment** | **Blinding of Participants/ Personnel** | **Blinding of Outcome Assessors** | **Incomplete Outcome Data** | **Selective Outcome Reporting** | **Other** | **Overall ROB** |
| --- | --- | --- | --- | --- | --- | --- | --- | --- |
| **Critical Outcome 1: Mortality** | | | | | | | | |
| ***All-cause mortality*** | | | | | | | | |
| Not reported | | | | | | | | |
| ***Cancer-related mortality*** | | | | | | | | |
| Not reported | | | | | | | | |
| **Critical Outcome 2 Survival** | | | | | | | | |
| Not reported | | | | | | | | |
| **Critical Outcome 3: Life threatening, severe, or medically significant consequences** | | | | | | | | |
| Sami 2015^52^ | + | - | - | - | + | - | ? | High |
| Zaman 1999^56^ | - | - | + | ? | + | ? | ? | Moderate |
| **Important Outcome 4: Incidence of EAC, BE, and low- and high-grade dysplasia** | | | | | | | | |
| ***Incidence of EAC*** | | | | | | | | |
| Jobe 2006^53^ | + | + | + | ? | + | ? | + | Moderate |
| ***Incidence of endoscopically suspected BE*** | | | | | | | | |
| Chak 2014^55^ | + | + | - | - | + | ? | + | High |
| Chang 2011^51^ | ? | ? | - | - | - | - | ? | High |
| Sami 2015^52^ | + | - | - | - | + | - | ? | High |
| Zaman 1999^56^ | ? | ? | - | - | + | ? | ? | High |
| ***Incidence of histologically confirmed BE*** | | | | | | | | |
| Chak 2014^55^ | + | + | + | + | + | ? | + | Low |
| Chang 2011^51^ | ? | ? | + | - | - | - | ? | High |
| Ferguson 2006^57^ | + | ? | + | + | + | ? | ? | Moderate |
| Sami 2015^52^ | + | - | + | + | + | - | ? | High |
| Wani 2014^58^ | ? | ? | + | + | + | ? | ? | Moderate |
| Jobe 2006^53^ | + | + | + | + | + | ? | + | Low |
| ***Incidence of low- and high-grade dysplasia*** | | | | | | | | |
| Chang 2011^51^ | ? | ? | ? | ? | ? | - | ? | Moderate |
| Jobe 2006^53^ | + | + | ? | ? | + | ? | + | Moderate |
| **Important Outcome 5: Quality of Life** | | | | | | | | |
| Not reported | | | | | | | | |
| **Important Outcome 6: Psychological effects** | | | | | | | | |
| Chak 2014^55^ | + | + | - | - | + | ? | + | High |
| Sami 2015^52^ | + | - | - | - | + | - | ? | High |
| Jobe 2006^53^ | + | + | - | - | + | ? | + | High |
| Zaman 1999^56^ | ? | ? | - | - | + | ? | ? | High |
| **Important Outcome 7: Major or minor medical procedures** | | | | | | | | |
| Not reported | | | | | | | | |
| **Important Outcome 8: Overdiagnosis** | | | | | | | | |
| Not reported | | | | | | | | |

(+) low risk; (?) unclear risk; (-) high risk

**Table 2. KQ1: Newcastle-Ottawa Scale (NOS) for Cohort studies**

| **Author/Year** | **SELECTION** | | | | **COMPARABILITY** | **OUTCOME** | | | **Overall ROB** |
| --- | --- | --- | --- | --- | --- | --- | --- | --- | --- |
|  | **Representativeness of the Exposed Cohort** | **Selection of the Non-Exposed Cohort** | **Ascertainment of Exposure** | **Demonstration That Outcome of Interest Was Not Present at Start of Study** | **Comparability of Cohorts on the Basis of the Design or Analysis** | **Assessment of Outcome** | **Was Follow-Up Long Enough for Outcomes to Occur** | **Adequacy of Follow Up of Cohorts** |  |
| **CRITICAL OUTCOME 1: Mortality** | | | | | | | | | |
| ***All-cause mortality*** | | | | | | | | | |
| Not reported | | | | | | | | | |
| ***Cancer-related mortality*** | | | | | | | | | |
| Not reported | | | | | | | | | |
| **CRITICAL OUTCOME 2: Survival** | | | | | | | | | |
| Rubenstein 2008^49^ | - | * | * | n/a | - | * | * | * | Moderate |
| **CRITICAL OUTCOME 3: Life threating, severe, or medically significant consequences** | | | | | | | | | |
| Not reported | | | | | | | | | |
| **IMPORTANT OUTCOME 4: Incidence of EAC (by stage), BE, and low- and high-grade dysplasia** | | | | | | | | | |
| ***Incidence of EAC*** | | | | | | | | | |
| Rubenstein 2008^49^ | - | * | * | n/a | - | * | * | * | Moderate |
| Hammad 2019^50^ | - | * | * | n/a | - | * | * | * | Moderate |
| ***Incidence of endoscopically suspected BE*** | | | | | | | | | |
| Mori 2010^54^ | - | * | * | - | * | - | n/a | n/a | High |
| ***Incidence of histologically confirmed BE*** | | | | | | | | | |
| Not reported | | | | | | | | | |
| ***Incidence of dysplasia*** | | | | | | | | | |
| Not reported | | | | | | | | | |
| **IMPORTANT OUTCOME 5: Quality of Life** | | | | | | | | | |
| Not reported | | | | | | | | | |
| **IMPORTANT OUTCOME 6: Psychological effects** | | | | | | | | | |
| Not reported | | | | | | | | | |
| **IMPORTANT OUTCOME 7:** **Major or minor medical procedures** | | | | | | | | | |
| Not reported | | | | | | | | | |
| **IMPORTANT OUTCOME 8: Overdiagnosis** | | | | | | | | | |
| Not reported | | | | | | | | | |

(*) low risk; (-) high risk; (n/a) not applicable

**Table 3. KQ2: Cochrane Risk of Bias for RCTs**

| **Author/Year** | **Sequence generation** | **Allocation Concealment** | **Blinding of Participants/ Personnel** | **Blinding of Outcome Assessors** | **Incomplete Outcome Data** | **Selective Outcome Reporting** | **Other** | **Overall ROB** |
| --- | --- | --- | --- | --- | --- | --- | --- | --- |
| **Chak 2014**^55^ | Low | Low | High | High | Low | Unclear | Unclear | High |

**Table 4. KQ3:** **AMSTAR ratings for included systematic reviews**

|  | **Fujii-Lau 2017**^92^ | **Desai 2017**^93^ | **Qumseya 2017**^73^ | **De Souza 2014**^68^ | **Almond 2014**^84^ | **Chadwick 2014**^91^ | **Fayter 2010**^70^ | **Rees 2010**^60^ | **Li 2008**^69^ | **Pandey 2018**^74^ | **Codipilly 2018**^104^ |
| --- | --- | --- | --- | --- | --- | --- | --- | --- | --- | --- | --- |
| **Confidence** | **Critically low**  (total score 7; 2 critical domains) | **Critically low**  (total score 4; 3 critical domains) | **Low**  (total score 8; 1 critical domain) | **Critically low**  (total score  2; 4 critical domains) | **Critically low**  (total score 2; 4 critical domains) | **Critically low**  (total score 4; 4 critical domains) | **Critically low**  (total score 6; 2 critical domains) | **Low**  (total score 8; 1 critical domain) | **Critically low** (total score 3; 4 critical domains) | **Critically Low**  (total score 3; 1 critical domain) | **Critically low**  (total score 6; 2 critical domains) |
| 1.Was an 'a priori' design provided | No | No | Yes | No | No | No | Yes | Yes | No | No | No |
| 2. Was there duplicate study selection and data extraction? | Yes | No | Yes | No | No | Yes | Yes | Yes | No | Can’t tell | Yes |
| 3. Was a comprehensive literature search performed? | Yes | No | Yes | No | No | No | Yes | Yes | No | Can’t tell | Yes |
| 4. Was the status of publication (i.e., grey literature) used as an inclusion criterion? | Yes | Yes | Can’t answer | Can’t answer | No | Yes | Yes | Yes | No | Can’t tell | Can’t tell |
| 5. Was a list of studies (included and excluded) provided? | No | No | No | No | No | No | No | Yes | No | No | No |
| 6. Were the characteristics of the included studies provided? | Yes | Yes | Yes | Yes | Yes | Yes | Yes | Yes | Yes | Yes | Yes |
| 7. Was the scientific quality of the included studies assessed and documented? | Yes | Yes | Yes | Yes | Yes | Yes | Yes | Yes | Yes | Yes | Yes |
| 8. Was the scientific quality of the included studies used appropriately in formulating conclusions? | No | No | Yes | No | Can’t answer | No | No | No | No | No | Yes |
| 9. Were the methods used to combine the findings of the studies appropriate? | Yes | No | Yes | No | Can’t answer | Can’t answer | Not applicable | Yes | Yes | No | Yes |
| 10. Was the likelihood of publication bias assessed? | Yes | Yes | Yes | No | No | No | No | No | No | Yes | N/A |
| 11. Was the conflict of interest included? | Can’t answer | No | Can’t answer | No | No | No | No | Can’t answer | Can’t answer | No | No |

Note: Highlighted texts demonstrate the critical domains

**Table 5. KQ3: Risk of bias/Methodological Assessments of Primary Studies**

| **Study (Review)** | **Notes** | **Outcome** | **Sequence generation/ randomization method** | **Allocation concealment** | **Blinding*†** | **Attrition†** | **Selective reporting** | **Other** |
| --- | --- | --- | --- | --- | --- | --- | --- | --- |
| Ackroyd 2000^66^  (Rees 2010)^60^ | Cochrane RoB tool used. Outcome-specific assessments were reported as pertaining to all outcomes. | All-cause mortality; Progression from IM to dysplasia; Complete eradication of dysplasia at two years; Reduction in length (cm) of BE at 12 months; Reduction in area (%) of BE at 12 months | Unclear | Low | Low | Unclear | High | Low |
| Ackroyd 2000^66^  (Li 2008)^69^ | Jadad score = 4/5. No specification on who was blinded (e.g., patients, physicians, outcome assessors, statisticians). Explanation provided for withdrawals and dropouts, but no information on the total number of withdrawals/ dropouts and relative number between arms. | Dysplasia eradication; area of regression of BE | Unclear | Sealed envelops | Double blind | Yes | n/a | n/a |
| Ackroyd 2000^66^  (De Souza 2014)^68^ | Jadad score = 3/5, with no details on specific items. | Treatment failure | n/a | n/a | n/a | n/a | n/a | n/a |
| Ackroyd 2000^66^  (Fayter 2010)^70^ | Use of an adopted checklist (not specified). Neither the outcome specific nor study specific assessments was reported (i.e., provided in aggregate among all included studies). | Evidence of regression | Unclear for almost 80% of the studies | Unclear for almost 90% of the studies | Unclear for almost 62% of the studies | Not carried out in almost 10% of the studies and unclear for almost 20% of the studies | n/a | n/a |
| Ackroyd 2004^72^  (Li 2008)^69^ | Jadad score = 2/5. Explanation provided for withdrawals and dropouts, but no information on the total number of withdrawals/ dropouts and relative number between arms. | Area of regression of BE | Unclear | Sealed envelops | None | Yes | n/a | n/a |
| Ackroyd 2004^72^  (De Souza 2014)^68^ | Jadad score = 2/5, with no details on specific items. | Treatment failure at one year | n/a | n/a | n/a | n/a | n/a | n/a |
| Bright 2007^71^  (Rees 2010)^60^ | Cochrane RoB tool used. Outcome-specific assessments were reported as pertaining to all outcomes. | Progression to EAC; Progression to dysplasia at 5 years; Complete eradication of BE at 12 months | Unclear | Low (sealed opaque envelops) | High | Low | High | Low |
| Bright 2007^71^  (Li 2008)^69^ | Jadad score = 2/5. Explanation provided for withdrawals and dropouts, but no information on the total number of withdrawals/ dropouts and relative number between arms. | Progression to HDG; Complete ablation (among those with histological change) | Unclear | Unclear | None | Yes | n/a | n/a |
| Caldwell 1996^62^ (abstract)  (Rees 2010)^60^ | Cochrane RoB tool used. Outcome-specific assessments were reported as pertaining to all outcomes. | Reduction in length (cm) of BE at 12 months | Unclear | Unclear | Unclear | Unclear | Unclear | High |
| Dulai 2005^82^  (Rees 2010)^60^ | Cochrane RoB tool used. Outcome-specific assessments were reported as pertaining to all outcomes. | All-cause mortality | Low | Low (sealed opaque envelopes) | Low | Low | Low | Low |
| Dulai 2005^82^  (Li 2008)^69^ | Jadad score = 2/5. Explanation provided for withdrawals and dropouts, but no information on the total number of withdrawals/ dropouts and relative number between arms. | Histological complete ablation | Unclear | Unclear | None | Yes | n/a | n/a |
| Dulai 2005^82^  (De Souza 2014)^68^ | Jadad score = 2/5, with no details on specific items. | Treatment failure | n/a | n/a | n/a | n/a | n/a | n/a |
| Hage 2004^86^  (Rees 2010)^60^ | Cochrane RoB tool used. Outcome-specific assessments were reported as pertaining to all outcomes. | All-cause mortality; Complete eradication of BE at 12 months; Stricture formation | Unclear | Unclear | High | Unclear | Unclear | Unclear |
| Hage 2004^86^  (Li 2008)^69^ | Jadad score = 2/5. Explanation provided for withdrawals and dropouts, but no information on the total number of withdrawals/ dropouts and relative number between arms. | Histologically complete ablation of BE | Unclear | Unclear | None | Yes | n/a | n/a |
| Hage 2004^86^  (De Souza 2014)^68^ | Jadad score = 2/5, with no details on specific items. | Treatment failure (no ablation of BE) | n/a | n/a | n/a | n/a | n/a | n/a |
| Hage 2004^86^  (Almond 2014)^84^ | Jadad score = 1/5 , with no details on specific items. | Cancer incidence; Progression to HGD; Complete eradication of dysplasia at 12 months | n/a | n/a | n/a | n/a | n/a | n/a |
| Hage 2004^86^  (Fayter 2010)^70^ | Use of an adopted checklist (not specified). Neither the outcome specific nor study specific assessments was reported (i.e., provided in aggregate among all included studies). | BE surface reduction | Unclear for almost 80% of the studies | Unclear for almost 90% of the studies | Unclear for almost 62% of the studies | Not carried out in almost 10% of the studies and unclear for almost 20% of the studies | n/a | n/a |
| Hage 2005^85^  (Li 2008)^69^ | Jadad score = 2/5. Explanation provided for withdrawals and dropouts, but no information on the total number of withdrawals/ dropouts and relative number between arms. | Histologically complete ablation of BE | Unclear | Unclear | None | Yes | n/a | n/a |
| Heath 2007^61^  (Rees 2010)^60^ | Cochrane RoB tool used. Outcome-specific assessments were reported as pertaining to all outcomes. | All-cause mortality; Progression to EAC at one year | Unclear | Unclear | Unclear | Low | Low | Low |
| Kelty 2004^87^  (Rees 2010)^60^ | Cochrane RoB tool used. Outcome-specific assessments were reported as pertaining to all outcomes. | All-cause mortality; Complete eradication of BE at 12 months; Stricture formation | Low | Unclear | Unclear | Unclear | Unclear | Unclear |
| Kelty 2004^87^  (Li 2008)^69^ | Jadad score = 3/5. Explanation provided for withdrawals and dropouts, but no information on the total number of withdrawals/ dropouts and relative number between arms. | Histologically complete ablation of BE | Low | Unclear | None | Yes | n/a | n/a |
| Kelty 2004^87^  (De Souza 2014)^68^ | Jadad score = 3/5, with no details on specific items. | Treatment failure (no ablation of BE) | n/a | n/a | n/a | n/a | n/a | n/a |
| Kelty 2004b^73^  (Fayter 2010)^70^ | Use of an adopted checklist (not specified). Neither the outcome specific nor study specific assessments was reported (i.e., provided in aggregate among all included studies). | Reductions in BE; Perforations or strictures | Unclear for almost 80% of the studies | Unclear for almost 90% of the studies | Unclear for almost 62% of the studies | Not carried out in almost 10% of the studies and unclear for almost 20% of the studies | n/a | n/a |
| Mackenzie 2007^78^  (abstract)  (Fayter 2010)^70^ | Use of an adopted checklist (not specified). Neither the outcome specific nor study specific assessments was reported (i.e., provided in aggregate among all included studies). | Cancer risk; Lower rates of adenocarcinoma; Stricture | Unclear for almost 80% of the studies | Unclear for almost 90% of the studies | Unclear for almost 62% of the studies | Not carried out in almost 10% of the studies and unclear for almost 20% of the studies | n/a | n/a |
| Mackenzie 2008^77^  (abstract)  (Rees 2010)^60^ | Cochrane RoB tool used. Outcome-specific assessments were reported as pertaining to all outcomes. It is unclear why there was a high-risk rating under the “other” domain. | Eradication of HGD; Stricture formation | Unclear | Unclear | Unclear | Unclear | Unclear | High (published in abstract) |
| Mackenzie 2009^79^  (Fayter 2010)^70^ | Use of an adopted checklist (not specified). Neither the outcome specific nor study specific assessments was reported (i.e., provided in aggregate among all included studies). | Lower rates of adenocarcinoma | Unclear for almost 80% of the studies | Unclear for almost 90% of the studies | Unclear for almost 62% of the studies | Not carried out in almost 10% of the studies and unclear for almost 20% of the studies | n/a | n/a |
| Overholt 2005^67^  (Rees 2010)^60^ | Cochrane RoB tool used. Outcome-specific assessments were reported as pertaining to all outcomes. Support for judgement points to another study. | All-cause mortality; Progression to cancer at latest possible time point; Complete eradication of dysplasia at two years; Stricture formation; Complete eradication of BE over the course of the study (5 years) | Unclear | Unclear | Unclear | Unclear | Unclear | Unclear |
| Overholt 2005^67^  (Li 2008)^69^ | Jadad score = 2/5. Explanation provided for withdrawals and dropouts, but no information on the total number of withdrawals/ dropouts and relative number between arms. | Dysplasia eradication; Eradication of HGD | Unclear | Unclear | None | Yes | n/a | n/a |
| Overholt 2005^67^  (De Souza 2014)^68^ | Jadad score = 2/5, with no details on specific items. | Treatment failure | n/a | n/a | n/a | n/a | n/a | n/a |
| Overholt 2007^65^  (Li 2008)^69^ | Jadad score = 2/5. Explanation provided for withdrawals and dropouts, but no information on the total number of withdrawals/ dropouts and relative number between arms | Progression to cancer at 5 years | Unclear | Unclear | None | Yes | n/a | n/a |
| Parrilla 2003^76^  (Rees 2010)^60^ | Cochrane RoB tool used. Outcome-specific assessments were reported as pertaining to all outcomes. | Mortality; Progression to cancer; Progression to dysplasia from intestinal metaplasia; Complete eradication of dysplasia at 5 years; Complete eradication of BE at 5 years | Low | Low | Unclear  (nature of study made blinding impossible; interpreted as high risk) | Unclear | Low | Low |
| Parrilla 2003^76^  (Li 2008)^69^ | Jadad score = 3/5. Explanation provided for withdrawals and dropouts, but no information on the total number of withdrawals/ dropouts and relative number between arms. | Progression from non-dysplastic BE to BE with dysplasia | Low | Sealed envelopes | None | Yes | n/a | n/a |
| Peters 1999^63^  (Rees 2010)^60^ | Cochrane RoB tool used. Outcome-specific assessments were reported as pertaining to all outcomes. | Reduction in length (cm) of BE at 12 months; Reduction in area (%) of BE at 12 months | Low | Unclear | Low | Unclear | Low | Unclear |
| Phoa 2014^81^  (Qumseya 2017)^73^ | Downs & Black 23 (Good). Based on sums, the tool may have been modified. Items relevant to risk of bias tool cannot be distinguished due to reporting.  (Poor quality if the score was <15, fair quality if the score was 15–19, and good quality if the score was >20.) | Progression to EAC: cumulative progression over the follow-up period; Progression to EAC: progression/patient-year; Progression to HGD; Progression to HDG: progression/patient-year | n/a | n/a | n/a | n/a | n/a | n/a |
|  |  |  | Black & Downs:  Reporting: 11  External validity: 3  Interval validity, bias: 4  Internal validity, confounding: 5  Power: 0 | | | | | |
| Phoa 2014^81^  (Pandey 2018)^74^ | Quality assessment was guided by the Cochrane RoB tool and Critical Appraisal Skills Programme (CASP) checklist. They quality was ranked from 1 to 4. This RCT was ranked as 1 (highest quality). Per outcome assessment was not provided. | Progression to HDG; Complete eradication of IM; Complete eradication of dysplasia; Stricture formation; Perforations; Bleeding | NR | NR | NR | NR | NR | NR |
| Ragunath 2005^88^  (Rees 2010)^60^ | Cochrane RoB tool used. Outcome-specific assessments were reported as pertaining to all outcomes. | All-cause mortality; Complete eradication of dysplasia at 12 months; Complete eradication of BE at 12 months; Reduction in length (cm) of BE at 12 months; Stricture formation | Low | Unclear | Unclear | Unclear | Low | Low |
| Ragunath 2005  (Li 2008) | Jadad score = 3/5. Explanation provided for withdrawals and dropouts, but no information on the total number of withdrawals/ dropouts and relative number between arms. | Length of regression (median) (endoscopic change) | Low | Unclear | None | Yes | n/a | n/a |
| Ragunath 2005^88^  (De Souza 2014) ^68^ | Jadad score = 3/5, with no details on specific items. | Treatment failure (no ablation of BE) | n/a | n/a | n/a | n/a | n/a | n/a |
| Ragunath 2005^88^  (Almond 2014)^84^ | Jadad score = 3/5, with no details on specific items. | Cancer incidence; Progression to HGD; Complete eradication of dysplasia at 12 months; Complete eradication of IM; Stricture | n/a | n/a | n/a | n/a | n/a | n/a |
| Shaheen 2009^75^  (Rees 2010)^47^ | Cochrane RoB tool used. Outcome-specific assessments were reported as pertaining to all outcomes. | Progression to EAC at 5-years (or latest time point); Progression to higher grades of dysplasia; Complete eradication of dysplasia at 12 months; Complete eradication of BE at 12 months; Stricture formation | Low | Unclear | Low | Low | Low | Low |
| Shaheen 2009^75^  (De Souza 2014)^68^ | Jadad score = 5/5, with no details on specific items. | Treatment failure at one year | n/a | n/a | n/a | n/a | n/a | n/a |
| Shaheen 2009^75^  (Qumseya 2017)^73^ | Downs & Black 27. Based on sums, the tool may have been modified. Items relevant to risk of bias tool cannot be distinguished due to reporting.  (Poor quality if the score was <15, fair quality if the score was 15–19, and good quality if the score was >20.) | Cumulative progression to EAC over follow-up (among those with LGD); Progression to HGD | n/a | n/a | n/a | n/a | n/a | n/a |
|  |  |  | Black & Downs:  Reporting: 12  External validity: 3  Interval validity, bias: 5  Internal validity, confounding: 6  Power: 1 | | | | | |
| Shaheen 2009^75^  (Pandey 2018)^74^ | Quality assessment was guided by the Cochrane RoB tool and Critical Appraisal Skills Programme (CASP) checklist. They ranked quality from 1 to 4. This study was ranked as 1 (highest quality). Per outcome assessment was not provided. | Progression to HGD; Complete; Eradication of intestinal metaplasia; Complete eradication of dysplasia; Perforations; Bleeding | NR | NR | NR | NR | NR | NR |
| Sharma 2006^83^  (Rees 2010) ^60^ | Cochrane RoB tool used. Outcome-specific assessments were reported as pertaining to all outcomes. | Stricture formation | Unclear | Low | Unclear | Unclear | Low | Low |
| Sharma 2006^83^  (Li 2008)^69^ | Jadad score = 2/5. Explanation provided for withdrawals and dropouts, but no information on the total number of withdrawals/ dropouts and relative number between arms. | Histological complete ablation | Unclear (“according to BE length”) | Sealed Envelope | None | Yes | n/a | n/a |
| Sharma 2006^83^  (De Souza 2014) ^68^ | Jadad score = 1/5, with no details on specific items. | Treatment failure | n/a | n/a | n/a | n/a | n/a | n/a |
| van Vilsteren 2011^95^  (Chadwick 2014)^91^ | Newcastle-Ottawa scale. No final score, information provided by domain. No indication that outcome-specific items were addressed by item – one assessment provided. | Complete eradication of dysplasia (end of treatment); Complete eradication of dysplasia with no recurrence at follow-up; Complete eradication of IM (end of treatment); Complete eradication of IM with no recurrence at follow-up; Acute bleeding endoscopically treated; Number of perforations; Stenosis requiring treatment | Authors state randomized but not method | n/a | Performance: unknown  Detection: low risk | Adequate follow-up rate | n/a | Groups comparable.  Other considerations? |
|  |  |  | NOS:  Representativeness of cohort: 1  Selection of non-exposed cohort: 1  Ascertainment of exposure: 1  Demonstration outcome of interest not present at start: 1  Comparability of cohorts on the basis of the design or analysis: 1  Assessment of outcome: 1  Was the follow-up long enough for outcomes to occur: 1  Adequacy of follow-up cohorts: 1 | | | | | |
|  |  |  | Reporting: 11  External validity: 3  Interval validity, bias: 4  Internal validity, confounding: 5  Power: 0 | | | | | |
| van Vilsteren 2011^95^  (Desai 2017)^93^ | Newcastle-Ottawa scale. Score = 7. No indication that outcome-specific items were addressed by item – one assessment provided. | Complete eradication of neoplasia; Complete eradication of IM; Recurrence of IM (follow up); Bleeding; Stricture | n/a | n/a | n/a | n/a | n/a | n/a |
|  |  |  | NOS:  Representativeness of cohort: 1  Selection of non-exposed cohort: not reported  Ascertainment of exposure: 1  Demonstration outcome of interest not present at start: 1  Comparability of cohorts on the basis of the design or analysis: 1  Assessment of outcome: 1  Was the follow-up long enough for outcomes to occur: 1  Adequacy of follow-up cohorts: 1 | | | | | |
| van Vilsteren^95^  (Fujii-Lau 2017)^92^ | Downs and Black. Rating for individual items reported. Those pertaining to risk of bias assessments are provided here. | Early neoplasia recurrence after complete eradication; Dysplasia recurrence after achieving complete eradication; IM recurrence | Score = 1 for random method | Score = 0 for concealment | Score = 0 for patient blinding  Score = 0 for blinding of outcome assessors | Score = 1 for loss to follow-up | n/a | n/a |
| Weinstein 1996^64^  (Rees 2010) ^60^ | Cochrane RoB tool used. Outcome-specific assessments were reported as pertaining to all outcomes. | Reduction in length (cm) of BE at 12 months; Reduction in area (%) of BE at 12 months | Unclear | Unclear | Unclear | Unclear | Unclear | Unclear |
| Zopf 2001^90^  (Almond 2014)^84^ | Jadad score = 1/5, with no details on specific items. | Cancer incidence | n/a | n/a | n/a | n/a | n/a | n/a |
| Zoepf 2003^89^  (Fayter 2010)^70^ | Use of an adopted checklist (not specified). Neither the outcome specific nor study specific assessments was reported (i.e., provided in aggregate among all included studies). | Reduction in length | Unclear for almost 80% of the studies | Unclear for almost 90% of the studies | Unclear for almost 62% of the studies | Not carried out in almost 10% of the studies and unclear for almost 20% of the studies | n/a | n/a |

Abbreviation: n/a=not available.

*Performance and detection bias

†Outcome-specific assessments.

** Rees 2010 used data from Overholt 2007 to supplement data in Overholt 2005.
