# Supplementary material for: Screening for esophageal adenocarcinoma and precancerous conditions (dysplasia and Barrett’s esophagus) in patients with chronic gastroesophageal reflux disease with or without other risk factors: two systematic reviews and one overview of reviews to inform a guideline of the Canadian Task Force on Preventive Health Care (CTFPHC)
Source: Syst Rev. 2020 Jan 29;9:20. doi: 10.1186/s13643-020-1275-2 (PMC6990541; doi:10.1186/s13643-020-1275-2)
Supplement: Supplementary file 16 — Additional file 16: KQ2 results. [file 13643_2020_1275_MOESM16_ESM.docx]

**Additional file 16. KQ2 Results table**

| **Author Year**  **Study design** | **Intervention & Comparator(s)** | **Results** |
| --- | --- | --- |
| Chak 2014^55^  RCT | Transnasal esophagoscopy vs. Video capsule esophagoscopy | 1210 eligible participants were invited, and 184 agreed.  **Unwillingness to participate**  1026 invited participants did not participate for the following reasons:   - Did not return phone call/did not respond to letter (n=627) - *Declined to participate (n=385) [with no reasons provided]* - EGD in past 10 years (n=2) - Did not meet inclusion criteria (n=10) - *Difficulty getting to the hospital (n=2)*   **Uptake of screening**  7 individuals declined post-randomization. In the TNE group, 5 wanted capsule instead. In the ECE group, 2 were worried about capsule getting stuck (p=0.25). |
| Zaman 1999^56^  RCT | T-EGD vs. P-EGD | 105 consecutive outpatients undergoing upper endoscopy because of GI symptoms were asked to participate and 45 patients refused participation (43%).  **Unwillingness to participate**  Reasons given included anxiety (18/105, 17%), a fear of gagging (10/105, 10%), not being interested in the study (10/105, 10%), or not wishing to undergo a transnasal procedure (7/45, 7%).    Many of these patients were expecting sedation when initially approached about the study. |
| Zaman 1998^59^  Cohort | P-EGD and sedated EGD | 62 patients undergoing outpatient endoscopy for gastrointestinal symptoms were asked to participate, of whom 19 refused participation (31%).  **Unwillingness to participate**  Reasons for nonparticipation included anxiety (12/62, 19%), fear of gagging (3/62, 5%), and unwillingness to be study subjects (4/62, 6%). |
