# Supplementary material for: Screening for esophageal adenocarcinoma and precancerous conditions (dysplasia and Barrett’s esophagus) in patients with chronic gastroesophageal reflux disease with or without other risk factors: two systematic reviews and one overview of reviews to inform a guideline of the Canadian Task Force on Preventive Health Care (CTFPHC)
Source: Syst Rev. 2020 Jan 29;9:20. doi: 10.1186/s13643-020-1275-2 (PMC6990541; doi:10.1186/s13643-020-1275-2)
Supplement: Supplementary file 17 — Additional file 17: List of potentially relevant ongoing studies. [file 13643_2020_1275_MOESM17_ESM.docx]

# Additional file 17. List of potentially relevant ongoing studies

## KQ1

| **Trial Identifier** | **Title** | **Estimated Study Completion Date** |
| --- | --- | --- |
| NCT02883621 | Comparison Between Standard Endoscopy and Cap Assisted Endoscopy for Diagnostic Yield in Esophagus | May 2017 |
| NCT02729948 | Use of a Tethered Capsule Endoscope in Screening for Barrett's Esophagus | August 2017 |
| NCT02852161 | The Accuracy and Acceptability of Magnet Assisted Capsule Endoscopy in the Diagnosis of Esophageal Pathology: a Pilot Study | October 2017 |
| NCT03009383 | A Bedside Portable Endoscopy for the Esophageal Foreign Body | December 2017 |
| NCT01438385 | Endoscopic Retrograde Cholangiopancreatography, Endoscopic Ultrasound and Interventional Endoscopy in Pancreatico-biliary, Gastrointestinal and Esophageal Disorders | December 2017 |
| NCT02395471 | Assessment of a Minimally Invasive Esophageal Cytology Collection System in Patients With Barrett's Esophagus or GERD Symptoms | June 2018 |
| NCT02685150 | The Role of Endoscopic Tri-Modal Imaging in Distinguishing Functional Dyspepsia From Reflux Disease | June 2018 |
| NCT02445014 | Pilot Study for Imaging of Barrett's Esophagus Using an Spectrally Encoded Confocal Microscopy Tethered Endoscopic Capsule | December 2018 |
| NCT01585103 | Cytosponge Protocol | September 2019 |
| ISRCTN68382401 | Barrett’s ESophagus Trial 3 (BEST3): Cluster randomised controlled trial comparing the Cytosponge-TFF3 test with usual care to facilitate the diagnosis of oesophageal pre-cancer in primary care. | September 2019 |
| ISRCTN76017289 | Quality of life measures in Barrett's Oesophagus care pathways | October 2019 |
| NCT02560623 | Minimally-Invasive Detection of Barrett's Esophagus and Barrett's Esophagus Related Dysplasia/Carcinoma by a Sponge on String Device | December 2019 |
| ISRCTN54190466 | Randomised controlled trial of surveillance and no surveillance for patients with Barrett's oesophagus: BOSS (Barrett's Oesophagus Surveillance Study) | June 2022 |
| NCT00987857 | Endoscopy Every 2 Years or Only as Needed in Monitoring Patients With Barrett Esophagus | May 2022 |
| NCT03596476 | Diagnostic Yield of Post PRandial Esophageal High Resolution Impedance Manometry in Patients With Gastro-Esophageal Reflux Disease Symptoms Resistant to Proton Pump Inhibitor Therapy (PRIMER) | January 2022 |
| NCT03596411 | The Detection of Barrett's Esophagus by Gastrointestinal Endoscopy Prevents Esophageal Carcinoma in Morbid Obese After Sleeve Gastrectomy (Refleeve) | November 2023 |
| NCT01688908 | Efficacy of Endoscopy Screening on Esophageal Cancer in a High Risk Region of Rural China: a Randomized Controlled Trial | December 2027 |
| NCT00903136 | Tethered Capsule Endoscope in Screening Participants for Barrett Esophagus | Unknown |
| NCT00341523 | Early Detection of Esophageal Cancer | Unknown |

## KQ2

| **Trial Identifier** | **Title** | **Estimated Study Completion Date** |
| --- | --- | --- |
| ISRCTN35624133 | Walk in nasal endoscopy (WINES) study: a pilot evaluation of the safety and feasibility, and cost savings of introducing a radically new approach to upper gastrointestinal (GI) endoscopy | December 2004 |
| NCT02852161 | The Accuracy and Acceptability of Magnet Assisted Capsule Endoscopy in the Diagnosis of Esophageal Pathology: a Pilot Study (MACE) | October 2017 |
| NCT02729948 | Use of a Tethered Capsule Endoscope in Screening for Barrett's Esophagus | August 2017 |
| NCT02395471 | Assessment of a Minimally Invasive Esophageal Cytology Collection System in Patients With Barrett's Esophagus or GERD Symptoms | June 2018 |
| ISRCTN68382401 | Barrett’s Esophagus Trial 3 (BEST3): Cluster randomised controlled trial comparing the Cytosponge-TFF3 test with usual care to facilitate the diagnosis of oesophageal pre-cancer in primary care. | September 2019 |
| NCT02445014 | Pilot Study for Imaging of Barrett's Esophagus Using an Spectrally Encoded Confocal Microscopy Tethered Endoscopic Capsule | December 2018 |

## KQ3

A list of ongoing Barrett’s Oesophagus studies referenced by Fayter 2010^50^:

| **S#** | **Investigator** | **Start date** | **Interventions** | **Status** |
| --- | --- | --- | --- | --- |
| 1 | Lovat L | February 2006 | ALA–PDT vs Ps PDT to study the side effect profile and to establish measures of efficacy in the eradication of dysplasia in Barrett’s oesophagus | Expected end February 2009 – but authors stated that the trial was ongoing; 55 out of 66 patients were recruited by January 2009 |
| 2 | Nava H | February 2004 | PDT in two light regimes for HGD and early cancer | Suspended, no reply to e-mail |
| 3 | Reed M | April 1995 | ALA–PDT (green light) vs placebo (all patients to take omeprazole) | Finished March 1996, no reply to e-mail |
| 4 | Wang K | September 2005 | Mucosal resection vs resection+PDT | Recruiting, no reply to e-mail |

List of Eligible SRs that should be tracked for emerging trials and results in the future:

Codipilly et al., 2018,^57^ assessed effect of endoscopic surveillance in patients with Barrett’s Esophagus. In addition to observational studies, it included one ongoing randomized clinical trial, the Barrett’s Oesophagus Surveillance Study (BOSS), being conducted in more than 100 hospitals and randomizes 3400 BE patients (1700 in each group: surveillance versus no surveillance). No outcome data was reported from this ongoing trial in this review.

Boghossian et al., 2017^87^ was a Cochrane review identified through an excluded clinical practice guideline (CPG).^88^ The CPG was excluded because it clearly stated that its recommendations does not apply to those with Barrett’s esophagus. The referenced SR was on deprescribing versus continuation of chronic proton pump inhibitor use in adults and one of the populations of interest was patients with Barrett’s esophagitis. However, it did not identify any study in this population. As such, no results pertaining the BE patients was provided. The review was excluded but it would need to be tracked in the future for any emerging trials.

Additional note: one CPG, Wani et al., 2018^89^, did not qualify as a systematic review and was excluded. Although the conduct of the evidence based does not meet the inclusion criteria, it addresses endoscopic eradication.
