# Supplementary material for: Screening for esophageal adenocarcinoma and precancerous conditions (dysplasia and Barrett’s esophagus) in patients with chronic gastroesophageal reflux disease with or without other risk factors: two systematic reviews and one overview of reviews to inform a guideline of the Canadian Task Force on Preventive Health Care (CTFPHC)
Source: Syst Rev. 2020 Jan 29;9:20. doi: 10.1186/s13643-020-1275-2 (PMC6990541; doi:10.1186/s13643-020-1275-2)
Supplement: Supplementary file 18 — Additional file 18: Overlap and concordance. [file 13643_2020_1275_MOESM18_ESM.docx]

# Additional file 18. Evaluation of overlap of studies and concordance of results among reviews

| **Evidence Set** | **Outcome(s)** | **Total publications^†^** | **# index publications** | **# reviews** | **CCA** | **Concordance** |
| --- | --- | --- | --- | --- | --- | --- |
| 2.1 | Reduction in area (%) of BE at 12 months | 3 | 2 | 2 | 0.5 | Yes |
| 3.1 | Progression to cancer at latest possible time point (up to 2 years) | 2 | 1 | 2 | 1 | Yes |
| 3.1 | Progression to cancer (at 5 years) | 2 | 1 | 2 | 1 | Yes |
| 3.1 | Progression from IM to dysplasia | 2 | 1 | 2 | 1 | Yes |
| 3.1 | Dysplasia eradication | 4 | 2 | 2 | 1 | Yes, reasonable overlap across reported data. |
| 3.1 | Complete eradication of BE over the course of the study (5 years) | 4 | 2 | 2 | 1 | Yes |
| 3.1 | Reduction in length (cm) of BE at 12 months; Reduction in area (%) of BE at 12 months; Area of regression; Evidence of regression | 3 | 1 | 3 | 1 | Outcomes measured differently across reviews; Li 2008 and Fayter 2010 are concordant for area of regression. |
| 5.1 | Progression to cancer at five years or latest time point; Cumulative progression to EAC over follow up | 2 | 1 | 2 | 1 | n/a: one review reports on patient subset |
| 5.1 | Progression to higher grades of dysplasia; Progression to high-grade dysplasia (Qumseya 2017); Progression to high-grade dysplasia (per patient-year) (among those with LGD); Progression to high-grade dysplasia (Pandey 2018) | 3 | 1 | 3 | 1 | Outcomes measured differently across reviews but the two reviews that report data in the same way are concordant. |
| 5.1 | Complete eradication of dysplasia at 12 months; Complete eradication of dysplasia | 2 | 1 | 2 | 1 | n/a: one reviews reports on patient subset |
| 6.1 | Progression to cancer | 2 | 1 | 2 | 1 | Yes |
| 6.1 | Progression to dysplasia from IM; Progression from non-dysplastic BE to BE with dysplasia | 2 | 1 | 2 | 1 | Different data reported for intervention group, led to discordant results. |
| 7.1 | Eradication of high-grade dysplasia | 2 | 1 | 2 | 1 | Yes |
| 8.1 | Progression to high-grade dysplasia | 2 | 1 | 2 | 1 | Yes |
| 9.2 | Histological complete ablation of BE; Treatment failure (no ablation of BE) | 6 | 2 | 3 | 1 | Yes, likely but some reporting issues. |
| 10.1 | Complete eradication of dysplasia at 12 months (see notes column of the evidence set 10.1 for this outcome that comments on 4 month data) |  |  |  | n/a | 1 review |
| 10.1 | Complete eradication of dysplasia at 12 months | 5 | 2 | 4 | 0.5 | Concordance not relevant; patient subset in Almond |
| 10.1 | Complete eradication of BE at 12 months; Complete eradication of IM | 5 | 3 | 3 | 0.3333333 | Differences in how information reported makes concordance assessment difficult across all three reviews, but Rees 2010 and Almond 2014 results overlap. |
| 10.1 | Reduction in length (cm) of BE at 12 months; BE surface reduction; Length of regression (median) (endoscopic change); Reduction in length | 4 | 3 | 3 | 0.1666667 | n/a: differences in measurement and reporting preclude assessment |
| 10.1 | Stricture formation; Stricture | 6 | 3 | 3 | 0.5 | n/a: Almond 2014 focused on patient subset (LGD), precluding concordance comparison; unclear if this explains inclusion of one trial only. Other two reviews reported information differently. |
| 11.1 | Complete eradication of IM; Complete eradication of IM (end of treatment) | 2 | 1 | 2 | 1 | Yes (although effect estimates not provided in one study, results are similar) |
| 11.1 | Complete eradication of IM with no recurrence at follow- up | 2 | 1 | 2 | 1 | Yes, although effect estimate available for one, available information similar |
| 11.1 | Acute bleeding endoscopically treated; Bleeding | 2 | 1 | 2 | 1 | Yes |

† including double counting

BE: Barrett’s esophagus; CCA: corrected covered area; IM: Intestinal metaplasia; LGD: Low-grade dysplasia
